# Chemodivergent manganese-catalyzed C–H activation: modular synthesis of fluorogenic probes

**DOI:** 10.1038/s41467-021-23462-9

**Published:** 2021-06-07

**Authors:** Nikolaos Kaplaneris, Jongwoo Son, Lorena Mendive-Tapia, Adelina Kopp, Nicole D. Barth, Isaac Maksso, Marc Vendrell, Lutz Ackermann

**Affiliations:** 1 Institut für Organische und Biomolekulare Chemie, Georg-August-Universität Göttingen, Göttingen, Germany; 2 Centre for Inflammation Research, The University of Edinburgh, Edinburgh, UK; 3 German Centre for Cardiovascular Research (DZHK), Berlin, Germany

**Keywords:** Homogeneous catalysis, Synthetic chemistry methodology

## Abstract

Bioorthogonal late-stage diversification of amino acids and peptides bears enormous potential for drug discovery and molecular imaging. Despite major accomplishments, these strategies largely rely on traditional, lengthy prefunctionalization methods, heavily involving precious transition-metal catalysis. Herein, we report on a resource-economical manganese(I)-catalyzed C–H fluorescent labeling of structurally complex peptides ensured by direct alkynylation and alkenylation manifolds. This modular strategy sets the stage for unraveling structure-activity relationships between structurally discrete fluorophores towards the rational design of BODIPY fluorogenic probes for real-time analysis of immune cell function.

## Abstract (web summary)

Bioorthogonal diversification of peptides is generally dependent on impractical prefunctionalization methods. Here, the authors develop a manganese(I)-catalyzed C–H fluorescent labeling with BODIPY probes, which enables the development of activatable fluorophores to image cell function.

## Introduction

The fluidity of cell membranes is an essential microenvironmental feature associated with many biological processes, including the transport of biomolecules, activity of membrane-bound proteins, protein–protein interactions, and metabolic reactions^1^. It is not surprising, therefore, that the dysregulation of membrane fluidity has been linked to cellular abnormalities in a variety of diseases^2,3^. In this regard, imaging studies to reveal how the composition of cell membranes changes in real-time are of key importance, both in biomedical studies as well as in the translation of new therapeutics. Fluorogenic molecular rotors have been recognized as attractive tools for sensing changes in the viscosity and polarity of live cells^4^. In the past few years, environmentally-sensitive fluorophores have been developed to image in vivo cell-specific events associated with infection, inflammation, and cancer^5–10^. However, this approach largely involves elements of substrate prefunctionalization. As a consequence, new approaches for late-stage fluorogenic labeling of biomolecules are required to assemble molecular probes that enable real-time imaging of membrane fluidity as direct reporters of cell function. The position- and chemo-selective modification of biologically relevant compounds is in high demand to program late-stage diversification of biomolecules with new spectral and biological capabilities^11–13^. This linch-pin approach allows to stitch different chemical moieties together to access highly functional entities with unique properties^14^. A major challenge in peptide functionalization is represented by the need for predictable, robust, and mild methods. Until recently, the modification of amino acids and peptides has largely relied on classical condensations, (cyclo)additions, or metal-catalyzed cross-couplings with two prefunctionalized substrates^15–18^. During the last decade, C–H activation has become an increasingly viable tool for molecular syntheses^19–23^. Enormous efforts have been devoted to establishing C–H functionalizations of amino acids with the toxic 4d transition metals palladium and ruthenium^24–29^, whilst Earth-abundant 3d transition metal catalysis continues to be scarce^30–33^. Manganese complexes typically demonstrate low toxicity, thus its utilization in C–H functionalization is highly desirable. While the viability of manganese(I)-catalysis for C–H activation has been documented^34–37^, late-stage diversifications of structurally complex peptides towards molecular imaging have thus far unfortunately proven elusive. CD8^+^ T cells are an effector subset of lymphocytes that represent the main line of defense of the adaptative immune system against infections and cancer^38–40^. Importantly, recent studies showed evidence that the composition of the plasma membrane of CD8^+^ T cells is directly related to their cytotoxic capability^41,42^. Since the biological activity of T cells can be indicative of how the host immune system is responding to disease, we focussed our design on the construction of functional BODIPY reporters of CD8^+^ T cell function.

Thus, we devise a manganese-catalyzed linch-pin strategy to stitch peptides with BODIPY fluorophores featuring excellent photophysical properties. Notably, the nature of the molecular linkage is identified as a key design element to modulate the fluorescent properties. This approach sets the stage for facilitating structure-activity relationship studies of BODIPY fluorogenic probes as well as the construction of unique functional reporters of cell activity. Herein, we exploit the environmentally-sensitive fluorogenicity of BODIPY chemotypes to image changes in the composition of the cell membrane of CD8^+^ T cells as direct indicators of their functional state.

## Results and discussion

**Fig. 1:**
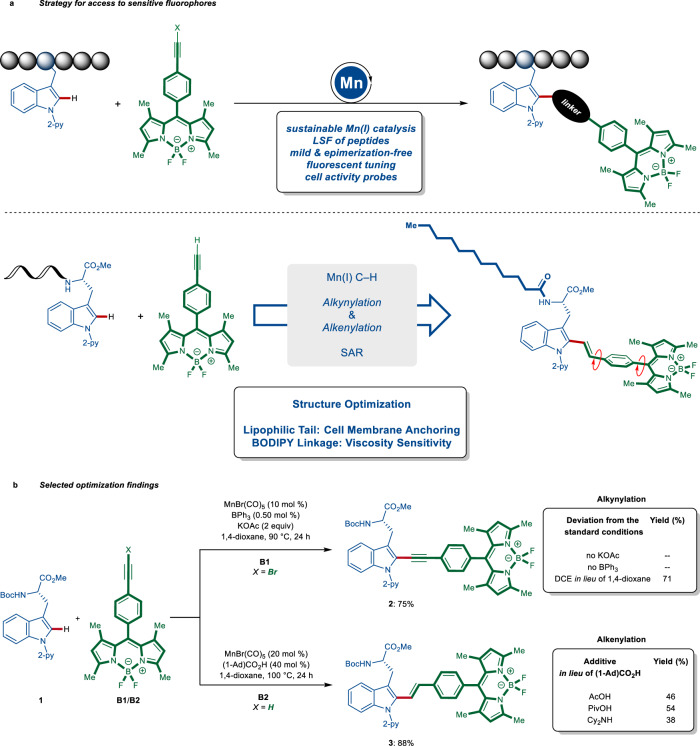
Divergent and modular assembly of fluorogenic probes via Earth-abundant manganese(I)-catalyzed C–H activation. **a** A versatile approach towards diverse fluorogenic probes. **b** Selected optimization findings for the utilization of bromoalkyne **B1** and terminal alkyne **B2** to furnish linear alkynylated and bent alkenylated BODIPY amino acid imaging probes. 2-py, 2-pyridyl; SAR, structure–activity relationship; (1-Ad)CO_2_H, 1-adamantanecarboxylic acid; DCE, 1,2-dichloroethane; PivOH, pivalic acid.

Our strategy for a divergent assembly point for the preparation of fluorescent peptides with tunable optical properties identified internal bromoalkynes^35^ and terminal alkynes^36^ as the substrates. We envisioned that, by employing these architectures, we could gain access to fluorescent imaging probes, in which the phenyl-BODIPY fluorescent core would be stitched to the indole-moiety of the tryptophan amino acid via a linear alkynyl or a bent alkenyl spacer, while at the same time extending the conjugate π-system to fine-tune the fluorogenic behavior. We envisioned that this linkage would not only alter the fluorescent properties of the peptide but also render fluorescent molecular rotors (Fig. 1a). To reduce our hypothesis into practice, we exploited an insertion/*β*-elimination manifold, via the combination of KOAc and BPh_3_, that enabled, under exceedingly mild, racemization-free reaction conditions, efficient alkynylation to selectively deliver BODIPY-labeled amino acid **2** with a linear linkage (Fig. 1b and Supplementary Tables 1 and 2). The beneficial assets of the Lewis-acidic BPh_3_ are attributed to ensuring an efficient *β*-bromide elimination^35^. In sharp contrast, (1-Ad)CO_2_H as additive facilitated an insertion/protodemetalation manifold with terminal BODIPY-alkyne **B2**, providing the bent spacer (Fig. 1b). The acid additive is proposed to enable efficient carboxylate-assisted C–H activation while accelerating the protodemetalation^43–45^.

**Fig. 2:**
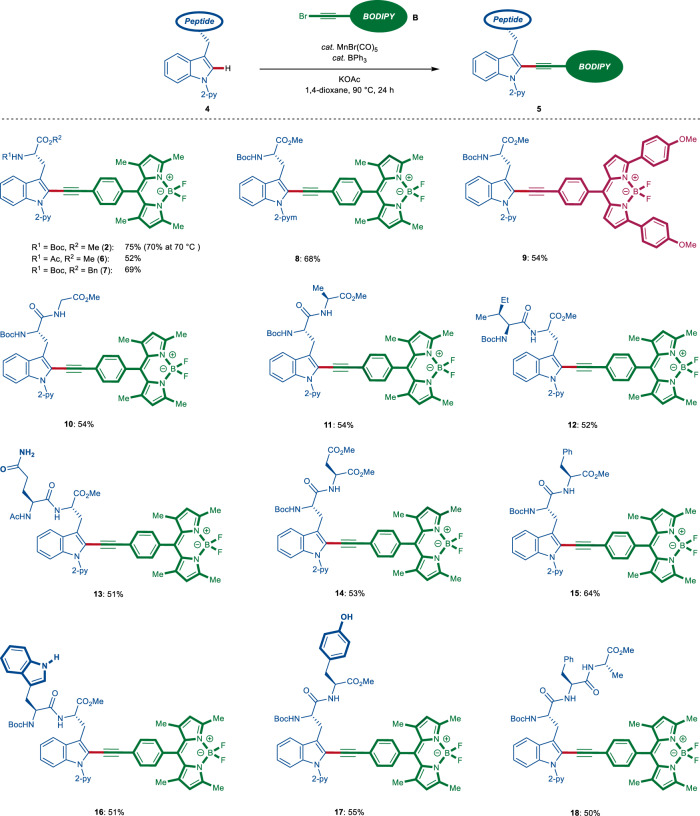
Manganese-catalyzed fluorescent labeling of small peptides via late-stage C–H stitching. Synthesis of peptides **6**–**18** featuring a linear linker between the BODIPY fluorophore and the tryptophan amino acid via C–H alkynylation.

With the optimal reaction conditions for late-stage fluorescent-labeling in hand, we explored the generality of our alkynylation regime in a broad range of small peptides (Fig. 2). Gratifyingly, the reaction conditions tolerated various tryptophan derivatives, delivering the desired fluorogenic amino acid derivatives **2**, **6**, and **7** with remarkable efficacy. Moreover, our approach proved viable to BODIPY-alkyne **B3** decorated with aryl groups at the alpha pyrrole positions to bathochromically shift the emission wavelength of the thus-obtained amino acid **9**. The robustness of our strategy is asserted with the preparation of various dipeptides which were selectively labeled without epimerization.

**Fig. 3:**
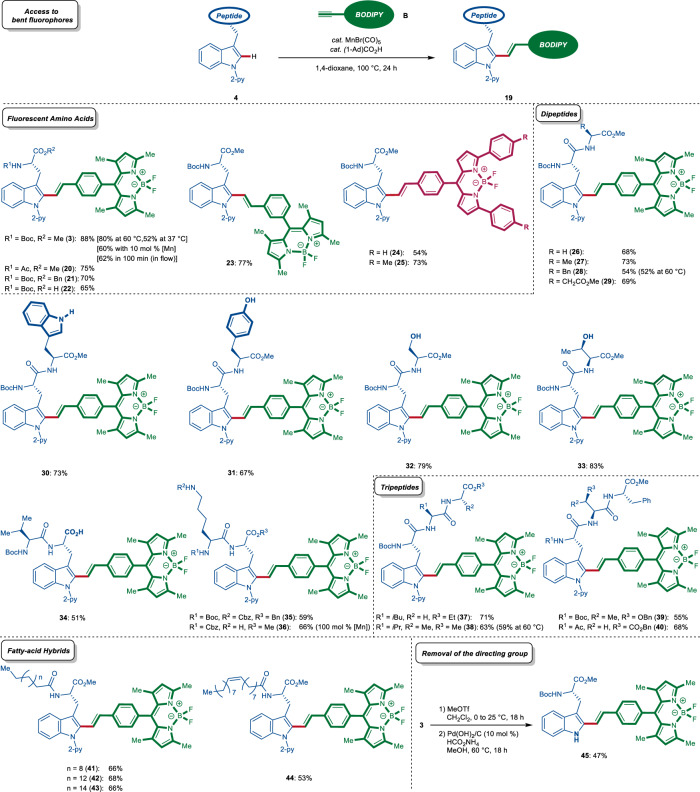
Manganese-catalyzed fluorescent labeling of small peptides via late-stage C–H stitching. Synthesis of peptides **20**–**45** featuring a bent linker between the BODIPY fluorophore and the tryptophan amino acid via late-stage C–H alkenylation.

Encouraged by the versatility of the manganese(I)-catalysis, we next investigated the late-stage diversification of complex peptides under the alkenylation manifold (Fig. 3). In this regard, various terminal BODIPY-alkynes (**B**) bearing substituents on the fluorescent core were efficiently converted to the desired labelled amino acid derivatives **3**, and **20**–**25** with complete *E*-selectivity. Importantly, the manganese(I)-catalyzed C–H activation even occurred at physiological temperature of 37 °C. Furthermore, the reaction time could be significantly reduced to only 100 min, when using a flow setup connected to a cartridge filled with the metal scavenger, QuadraPure™ IDA. Thereby, the residual manganese level was reduced to 6.1 ppm, as determined by inductively coupled plasma mass spectrometry (ICP-MS) analysis. Likewise, we provided access to the free fluorescent amino acid **22** as a building block for the synthesis of larger fluorogenic peptides. Various di- and tripeptides with unprotected, -OH, -CO_2_H, and -NH_2_-free amino acids, such as tryptophan, tyrosine, serine, threonine, and lysine, were converted in a site- and chemoselective fashion. Also, fluorescent free-C-terminus dipeptide **34** was obtained in a positional selective fashion. Triggered by the robustness of our alkenylation regime, we examined whether more complex peptides would be amenable to this site-selective labeling process. To this end, tripeptides **4** were site-, chemo-, and diastereo-*E*-selectively converted. The robustness of our alkenylation regime was demonstrated by the efficient synthesis of the fluorescent dipeptide **28** and tripeptide **38** at 60 °C. Tryptophan derivatives containing lipophilic tails at the N-terminus where also subjected to the labeling process to afford modulated lipophilic rotors that could be used as membrane fluidity probes (vide infra). Thus, the linch-pin approach stitched lauric, palmitic, stearic, and *cis*-oleic acid together onto tryptophans, leading to *E*-bent fluorogenic probes **41**–**44**. Moreover, the traceless removal of the N-pyridyl group was accomplished by a selective methylation/ hydrogenation protocol giving rise to NH-free tryptophan-containing fluorogenic amino acid **45**.

**Fig. 4:**
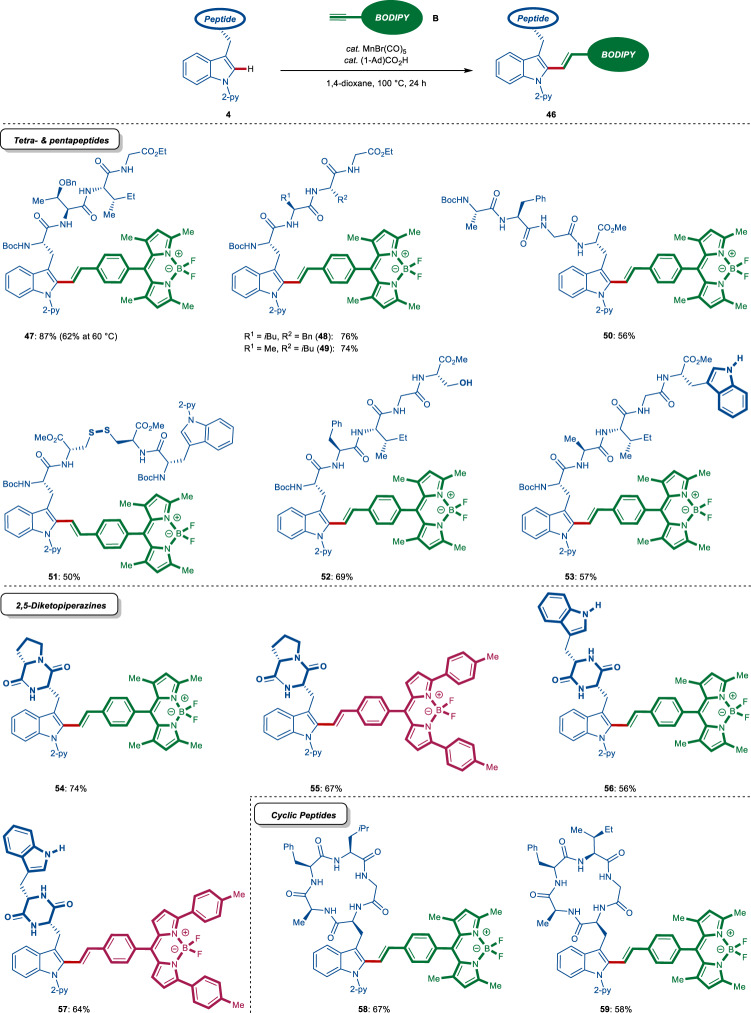
Manganese-catalyzed fluorescent labeling of complex peptides via late-stage C–H stitching. Synthesis of peptides **47**–**53** featuring a bent linker between the BODIPY fluorophore and the tryptophan via C–H stitching, late-stage diversification of natural product derivatives featuring the 2,5-diketopiperazine core (**54**–**57**) and synthesis of fluorescent cyclic peptides via C–H alkenylation.

Inspired by the remarkable versatility of our manganese(I)-catalyzed alkenylation, we became attracted to manganese(I) catalysis for the labeling of more challenging peptidic structures. To our delight, the linch-pin approach proved robust for the selective and efficient labeling of tetra- and pentapeptides **47**–**53**, featuring sensitive cystine and unprotected tryptophan and OH-free serine (Fig. 4). Likewise, the late-stage diversification of conformationally rigid cyclic peptides was realized, enabling access to brevianamide F (cyclo[Trp^py^-Pro]) and fellutanine A (cyclo[Trp^py^-Trp]) diketopiperazine fluorescent analogs **54**–**57** (Fig. 4). Cyclic pentapeptides were also selectively labeled, giving access to fluorescent peptides **58** and **59** with potentially improved resistance to proteolytic degradation (Fig. 4). These findings assert the utility of our manganese(I) catalysis to directly access proteolytically-stable fluorescent peptides for bioimaging studies, without relying on lengthy de novo syntheses based on pre-functionalized building blocks.

CD8^+^ T cells are key mediators of the immune response against pathogens and cancer cells. Once T cells are activated, they exert their killing activity through the production and release of cytotoxic cytokines and enzymes. Within the last decades, immunotherapies have emerged as an effective treatment to invigorate the activity of CD8^+^ T cells against tumors^46^. However, immunotherapies show limited success and high variability between patients, which highlights the need for chemical tools that can provide direct readouts of immune response to accelerate the design of more efficient treatments^47^. The cytotoxic ability of CD8^+^ T cells has been recently linked to lipid metabolism^41,42^. Different studies report that increases of cholesterol—an essential component of lipid bilayer fluidity—in the plasma membrane are directly associated with rapid cell proliferation and enhanced effector function. Given the suitability of our synthetic platform for the rapid optimization of fluorophores, we decided to explore whether BODIPY fluorogenic probes would monitor changes in the membrane fluidity of CD8^+^ T cells and behave as fluorescent reporters of cellular activity.

**Fig. 5:**
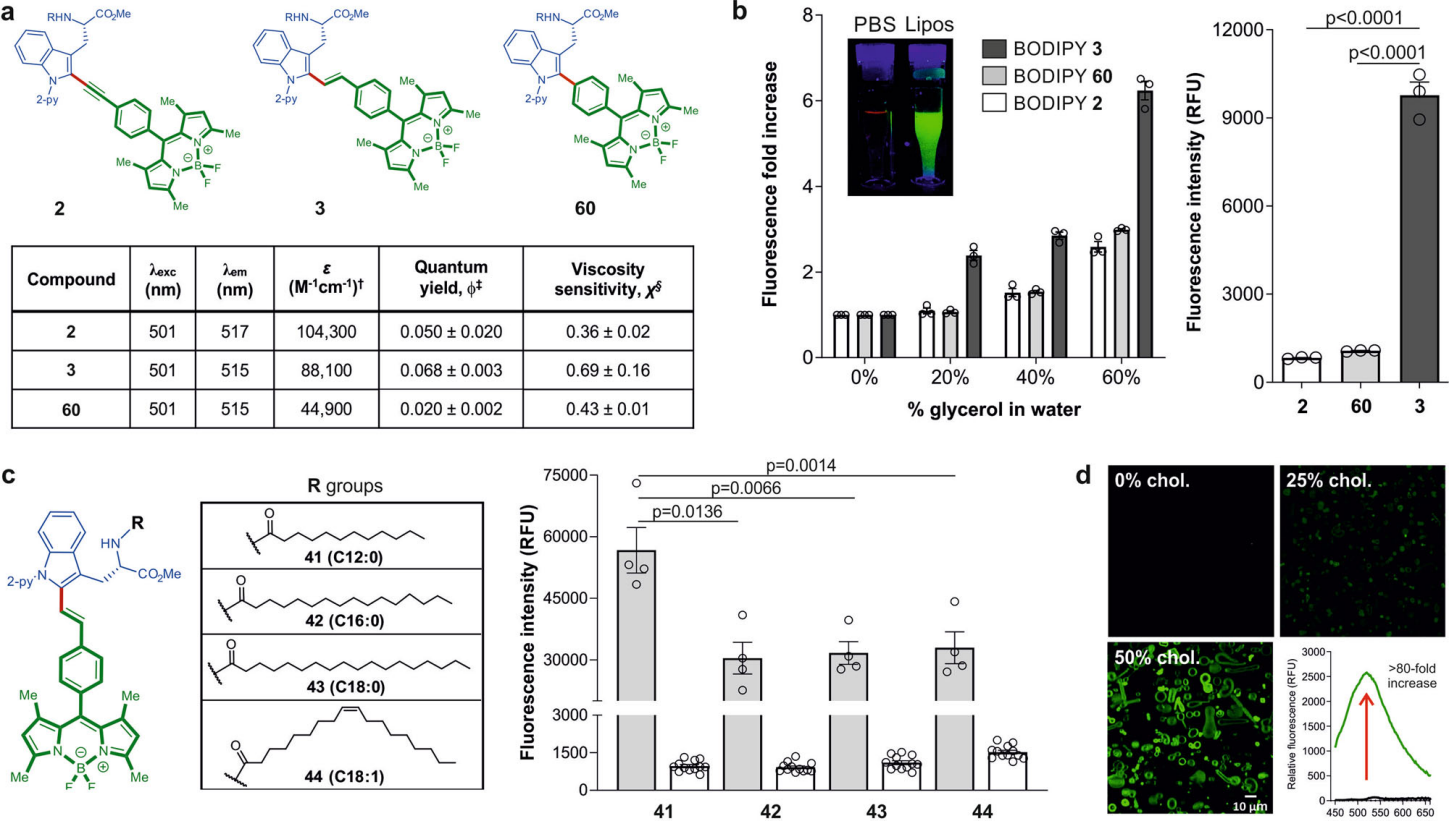
Evaluation of fluidity-sensitive fluorogenic BODIPY probes. **a** Spectral properties of the BODIPY amino acids **2, 3**, and **60**. *λ*_*exc*_: 450 nm. [^†^] Measured in EtOH, [^‡^] measured in glycerol:H_2_O (6:4) with fluorescein in basic EtOH as a standard, [^§^] measured in glycerol:H_2_O mixtures with increasing viscosity. **b** Fluorescence bar plots displaying the fluorescence fold increase in 0–60% glycerol in water (left) and fluorescence emission in 60% glycerol-water (right) of amino acids **2, 3**, and **60** (25 µM), *λ*_*exc*_: 450 nm. Inset) Pictograms of compound **3** under UV excitation in PBS (left) and in liposome suspensions (right). **c** Fluorescence emission bar plot of lipid-fluorophore conjugates **41**–**44** (1 µM) after incubation in phosphatidylcholine (PC): cholesterol liposome suspensions (gray) and in PBS (white), *λ*_*exc*_: 450 nm. **d** Fluorescence confocal microscopy images of compound **41** (1 µM, green) after incubation with DMPC-based liposomes containing increasing amounts of cholesterol. Scale bar: 10 µm. In all panels, data presented as means ± SEM from experiments performed at least in triplicate. Fluorescence spectra of compound **41** (1 µM) in cholesterol-containing liposomes (green) and in PBS (black). Data presented as means ± SEM (*n* = 4) for emission in presence of liposomes and (*n* = 12) for emission in PBS. *P* values obtained from ONE-ANOVA tests with multiple comparisons. *ε*, molar extinction coefficient; lipos., liposomes; chol., cholesterol.

First, we examined the Trp(BODIPY) analogs **2**, **3**, and **60** as membrane viscosity sensors (Fig. 5a). All three analogs exhibited similar excitation (~501 nm) and green emission (~515 nm) maxima wavelengths (Supplementary Figs. 4–6). To examine their response to changes in fluidity, we first measured their fluorescence emission in water-glycerol mixtures, where the derivative **3**, including a π-conjugated double bond bridge that could favor the charge transfer between the indole and phenyl-BODIPY groups, showed the best fluorogenic character among all the derivatives (Fig. 5b). The weakest response was observed for derivative **2**, which features a triple bond linkage that could lead to increased system rigidification and a reduction of intramolecular charge transfer. We quantified the viscosity sensitivity coefficients for all compounds and observed that compound **3** displayed values around 0.7, being among the most sensitive fluorescent rotors reported to date^48,49^. Next, to facilitate the anchoring of the BODIPY reporter to the plasma membrane of live cells, we conjugated compound **3** to fatty acids of different lengths and saturation degrees (Fig. 5c). We examined their fluorescence emission in liposomes containing phosphatidylcholine and cholesterol, mimicking the cell membrane microenvironments. Interestingly, the lauric acid derivative **41**, which had the shortest fatty acid chain (i.e., 12 carbon atoms) exhibited the strongest fluorogenic behavior with over 80-fold increase in emission (Fig. 5c). In view of these results, we performed confocal microscopy experiments to compare the staining of compound **41** in liposomes containing different amounts of cholesterol. As shown in Fig. 5d, compound **41** exhibited turn-on fluorescence emission in response to the cholesterol content.

**Fig. 6:**
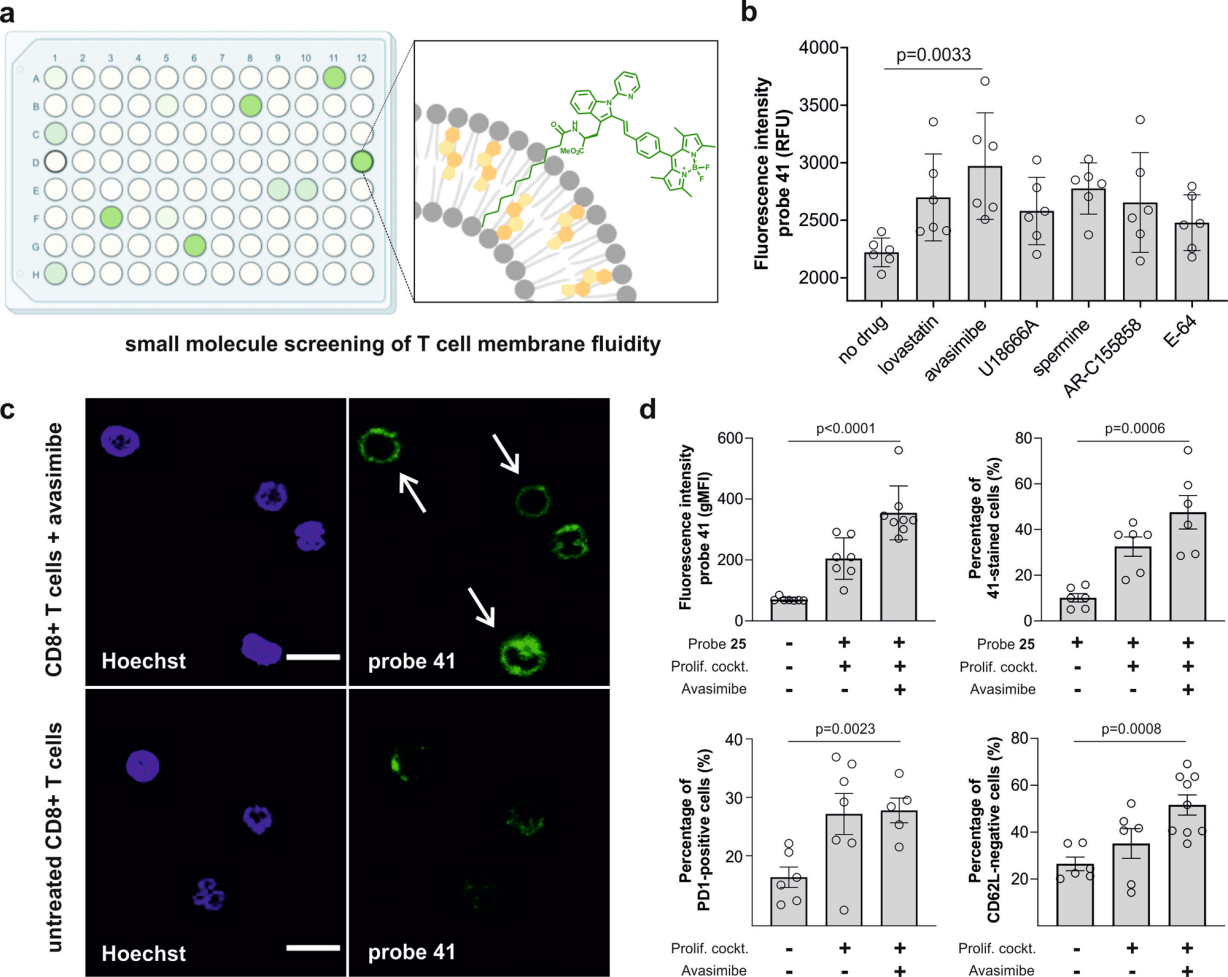
Probe **41** enables fluorescence-based screening of small molecule modulators of CD8^+^ T cells. **a** Schematic illustration of our fluorescence-based screening to detect cholesterol fluctuations in T cells using compound **41**. **b** Quantification of the fluorescence emission of Jurkat T cells after incubation with different small molecules and staining with compound **41** (1 µM, *λ*_*exc*_/_*em*_: 488/525 nm). Data presented as means + SEM (*n* = 6). **c** Fluorescence confocal microscopy images of activated CD8^+^ T cells with avasimibe (30 µM) or without avasimibe. Cells were stained with compound **41** (1 µM, green) and Hoechst 33342 (blue, 7 µM) (*λ*_*exc*_/_*em*_: 405/450 nm (Hoechst), 488/525 nm (**4**)). White arrows point at the plasma membrane localization of probe **41**. Scale bar: 10 µm. **d** Quantification of the fluorescence intensity (*n* = 7) and the percentage (*n* = 6) of **41**-stained CD8^+^ T cells under different activation conditions. Flow cytometry analysis of the CD8^+^ T cells markers PD-1 (no avasimibe: *n* = 6, IL-2 activation: *n* = 7, IL-2 activation + avasimibe: *n* = 5) and CD62L (no avasimibe/IL2-activation: *n* = 6, IL-2 activation+avasimibe: *n* = 9) before and after treatment with avasimibe (30 µM). In all panels, data presented as means ± SEM from experiments performed at least in triplicate. *P* values obtained from unpaired two-tailed *t* tests.

Encouraged by the sensitivity of compound **41** to monitor changes in membrane fluidity, we next evaluated its utility as a fluorescent probe for identifying small molecule drugs that activate T cells and regulate the cholesterol content in their membranes. We employed a flow cytometry platform to screen a collection of commercially available regulators of lipid metabolism (Fig. 6a). Jurkat T cells were cultured using a cell proliferation cocktail (i.e., anti-CD3, anti-CD28, and IL-2) and treated with drugs or vehicle for 12 h before imaging with probe **41**. Among all drugs tested, cells treated with the Acyl-CoA:cholesterol acyltransferase inhibitor avasimibe showed the highest fluorescence emission (Fig. 6b). Notably, avasimibe has been reported in combination with anti-PD1 immunotherapies for the treatment of cancer. We also examined whether compound **41** was able to detect changes in the activity of CD8^+^ T cells that were freshly isolated from human peripheral blood. Fluorescence microscopy images of compound **41** in human CD8^+^ T cells exhibited bright staining of the cell membrane in avasimibe-treated cells but not in untreated CD8^+^ T cells (Fig. 6c). Moreover, we performed a detailed live-cell analysis by flow cytometry to observe that the fluorescence signal of compound **41** was significantly higher in avasimibe-treated activated CD8^+^ T cells than in naïve CD8^+^ T cells (Fig. 6d and Supplementary Fig. 8). Finally, we confirmed the functional state of CD8^+^ T cells by measuring the expression of receptor markers that are directly associated with T cell activity and assessed the response of human CD8^+^ T cells using antibody-based readouts (Supplementary Fig. 7). Specifically, avasimibe-treated cells showed a slight increase in the levels of PD-1 and a significant decrease in the levels CD62L, which are both indicative of CD8^+^ T cell activation state (Fig. 6d and Supplementary Fig. 9). Altogether, these results highlight the utility of compound **41** as a simple and cost-effective turn-on probe to detect subtle changes in the cholesterol content and activation state of live human CD8^+^ T cells under physiological conditions.

In summary, we have developed a modular linch-pin strategy for the stereo-divergent manganese(I)-catalyzed C–H labeling of structurally complex peptides with BODIPY probes. The modular and robust nature of our approach sets the stage for expedient structure–activity relationship studies of fluorogenic peptides. We validated the Earth-abundant manganese(I) catalysis by providing access to highly sensitive molecular rotors with the ability to sense changes in the composition of immune cell membranes. We identified a fluidity-sensitive probe, which emits bright fluorescence in response to cholesterol found in cell membranes, and utilized it to establish a rapid fluorescence-based screening platform for the identification of small molecule modulators of CD8^+^ T cells. Overall, our modular and divergent C–H activation strategy will establish expedient avenues in the development of activatable fluorophores to image cell function in real-time and physiological conditions.

## Methods

### General procedure A: manganese-catalyzed C–H alkynylation of peptides

A suspension of peptide **4** (0.10 mmol, 1.00 equiv), BODIPY-bromoalkyne **B** (0.11 mmol, 1.1 equiv), MnBr(CO)_5_ (2.7 mg, 10 mol %), KOAc (19.6 mg, 2.0 equiv), and BPh_3_ (50 µL, 0.01 m in 1,4-dioxane, 0.5 mol %) in 1,4-dioxane (1.0 mL) was stirred at 90 °C for 24 h. After cooling to ambient temperature, CH_2_Cl_2_ (10 mL) was added and the mixture was concentrated in vacuo. Purification by column chromatography on silica gel afforded the desired products.

### General procedure B: manganese-catalyzed C–H hydroarylation of peptides

A suspension of peptide **4** (0.10 mmol, 1.0 equiv), BODIPY-alkyne **B** (0.11-0.20 mmol, 1.1 -2.0 equiv), MnBr(CO)_5_ (5.5 mg, 20 mol %), and (1-Ad)CO_2_H (7.2 mg, 40 mol %) in 1,4-dioxane (1.0 mL) was stirred at 100 °C for 24 h. After cooling to ambient temperature, CH_2_Cl_2_ (10 mL) was added and the mixture was concentrated in vacuo. Purification by column chromatography on silica gel afforded the desired products.

### Flow cytometry analysis

Dyes were reconstituted at 10 mM in DMSO. 10^5^ cells were stained with compound **41** (1 μM) in HEPES-NaCl containing 0.1% BSA for 1 h at r.t. prior to staining with antibodies. Cells were washed with HEPES-NaCl followed by staining with antihuman PD-1-PE (1:100, 10 μg mL^−1^), antihuman CD62L-PE/Cy7 (1:200, 10 μg mL^−1^), antihuman CD39-APC (1:200, 5 μg mL^−1^), and antihuman CD8-PerCP/Cy5.5 (1:200, 0.4 μg mL^−1^) in HEPES-NaCl containing 0.1% BSA and 1% FcR block for 20 min on ice. Cells were washed at 300 *g*, 5 min in HEPES-NaCl prior to staining with 25 nM Annexin V-PB in HEPES-NaCl containing 0.1% BSA and 2 mM CaCl_2_. Fluorescence emission was measured on a 5 L LSR flow cytometer. Excitation sources/emission filters used: Annexin V-PB (355 nm, 450/50 nm), **41** (488 nm, 510/20 nm), PD1-PE (561 nm, 582/15 nm), CD62L-PE/Cy7 (561 nm, 780/60 nm), CD8-PerCP/Cy5.5 (488 nm, 710/50 nm), CD39-APC (647 nm, 670/14 nm).

### Fluorescence confocal microscopy

Confocal imaging was performed in a TCS Leica SP8 confocal laser scanning microscope. Prior to imaging, cells were stained with compound **41** (1 μM) for 1 h at r.t. in HEPES-NaCl containing 0.1% BSA. Cells were further counterstained with the nuclear counterstain Hoechst 33342 (3 μM) for 10 min at r.t. Excitation/emission wavelengths: Hoechst 33342 (355 nm, 450/50 nm), **41** (488 nm, 520/40 nm). Images were processed with ImageJ.

### Statistics and reproducibility

Statistical differences were analyzed two-sided unpaired *t* tests using Graphpad software. Independent experiments were performed at least three times, unless otherwise mentioned.

### Reporting summary

Further information on research design is available in the Nature Research Reporting Summary linked to this article.

## Supplementary information

Supplementary Information

Reporting Summary

## Data Availability

The authors declare that the data supporting the findings of this study are available within the paper and its Supplementary Information files. Source data are provided with this paper. All other requests for materials and information should be addressed to the corresponding authors. Source data are provided with this paper.

## Source data

Source Data

## References

[1] Bernardino de la Serna J, Schütz GJ, Eggeling C, Cebecauer M (2016). There is no simple model of the plasma membrane organization. Front. Cell Dev. Biol.

[2] Kuter K (2016). Adaptation within mitochondrial oxidative phosphorylation supercomplexes and membrane viscosity during degeneration of dopaminergic neurons in an animal model of early Parkinson’s disease. Biochim. Biophys. Acta, Mol. Basis Dis..

[3] Martín V (2010). Lipid alterations in lipid rafts from Alzheimer’s Disease human brain cortex. J. Alzheimer.

[4] Yang Z (2014). Macro-/micro-environment-sensitive chemosensing and biological imaging. Chem. Soc. Rev..

[5] Subiros-Funosas R (2020). Fluorogenic Trp(redBODIPY) cyclopeptide targeting keratin 1 for imaging of aggressive carcinomas. Chem. Sci..

[6] Fernandez A, Thompson EJ, Pollard JW, Kitamura T, Vendrell M (2019). A fluorescent activatable AND-gate chemokine CCL2 enables in vivo detection of metastasis-associated macrophages. Angew. Chem. Int. Ed..

[7] Benson S (2019). SCOTfluors: small, conjugatable, orthogonal, and tunable fluorophores for in vivo imaging of cell metabolism. Angew. Chem. Int. Ed..

[8] Patalag LJ, Ho LP, Jones PG, Werz DB (2017). Ethylene-bridged Oligo-BODIPYs: access to intramolecular J-aggregates and superfluorophores. J. Am. Chem. Soc..

[9] Fernandez A (2017). Chemical modulation of in vivo macrophage function with subpopulation-specific fluorescent prodrug conjugates. ACS Cent. Sci..

[10] Mendive-Tapia L (2016). Spacer-free BODIPY fluorogens in antimicrobial peptides for direct imaging of fungal infection in human tissue. Nat. Commun..

[11] Tong H-R, Li B, Li G, He G, Chen G (2020). Postassembly modifications of peptides via metal-catalyzed C−H functionalization. CCS Chem..

[12] Wang W, Lorion MM, Shah J, Kapdi AR, Ackermann L (2018). Late-stage peptide diversification by position-selective C−H activation. Angew. Chem. Int. Ed..

[13] Noisier AFM, Brimble MA (2014). C–H functionalization in the synthesis of amino acids and peptides. Chem. Rev..

[14] Ohata J, Martin SC, Ball ZT (2019). Metal-mediated functionalization of natural peptides and proteins: panning for bioconjugation gold. Angew. Chem. Int. Ed..

[15] Zhang C, Vinogradova EV, Spokoyny AM, Buchwald SL, Pentelute BL (2019). Arylation chemistry for bioconjugation. Angew. Chem. Int. Ed..

[16] Hoyt EA, Cal PMSD, Oliveira BL, Bernardes GJL (2019). Contemporary approaches to site-selective protein modification. Nat. Rev. Chem..

[17] Jbara M, Maity SK, Brik A (2017). Palladium in the chemical synthesis and modification of proteins. Angew. Chem. Int. Ed..

[18] deGruyter JN, Malins LR, Baran PS (2017). Residue-specific peptide modification: a chemist’s guide. Biochemistry.

[19] Rej S, Ano Y, Chatani N (2020). Bidentate directing groups: an efficient tool in c–h bond functionalization chemistry for the expedient construction of C–C bonds. Chem. Rev..

[20] Meng G (2020). Achieving site-selectivity for C–H activation processes based on distance and geometry: a carpenter’s approach. J. Am. Chem. Soc..

[21] Blakemore DC (2018). Organic synthesis provides opportunities to transform drug discovery. Nat. Chem..

[22] Park Y, Kim Y, Chang S (2017). Transition metal-catalyzed C–H amination: scope, mechanism, and applications. Chem. Rev..

[23] Gensch T, Hopkinson MN, Glorius F, Wencel-Delord J (2016). Mild metal-catalyzed C–H activation: examples and concepts. Chem. Soc. Rev..

[24] Zhang X (2018). A general strategy for synthesis of cyclophane-braced peptide macrocycles via palladium-catalysed intramolecular *sp*^*3*^ C−H arylation. Nat. Chem..

[25] Tang J (2018). Peptide-guided functionalization and macrocyclization of bioactive peptidosulfonamides by Pd(II)-catalyzed late-stage C–H activation. Nat. Commun..

[26] Schischko A, Ren H, Kaplaneris N, Ackermann L (2017). Bioorthogonal diversification of peptides through selective ruthenium(II)-catalyzed C–H activation. Angew. Chem. Int. Ed..

[27] Liu T, Qiao JX, Poss MA, Yu J-Q (2017). Palladium(II)-catalyzed site-selective C(sp^3^)−H alkynylation of oligopeptides: a linchpin approach for oligopeptide–drug Conjugation. Angew. Chem. Int. Ed..

[28] Liu Y-J (2016). Divergent and stereoselective synthesis of β-silyl-α-amino acids through palladium-catalyzed intermolecular silylation of unactivated primary and secondary C−H bonds. Angew. Chem. Int. Ed..

[29] Chen G (2015). Ligand-Enabled β-C–H arylation of α-amino acids using a simple and practical auxiliary. J. Am. Chem. Soc..

[30] Scamp RJ, deRamon E, Paulson EK, Miller SJ, Ellman JA (2020). Cobalt(III)-catalyzed C−H amidation of dehydroalanine for the site-selective structural diversification of thiostrepton. Angew. Chem. Int. Ed..

[31] Lorion MM, Kaplaneris N, Son J, Kuniyil R, Ackermann L (2019). Late-stage peptide diversification through cobalt-catalyzed C−H activation: sequential multicatalysis for stapled peptides. Angew. Chem. Int. Ed..

[32] Kaplaneris N (2019). Late-stage diversification through manganese-catalyzed C−H activation: access to acyclic, hybrid, and stapled peptides. Angew. Chem. Int. Ed..

[33] Gandeepan P (2019). 3d Transition metals for C–H activation. Chem. Rev..

[34] Hu Y, Zhou B, Wang C (2018). Inert C–H bond transformations enabled by organometallic manganese catalysis. Acc. Chem. Res..

[35] Ruan Z, Sauermann N, Manoni E, Ackermann L (2017). Manganese-catalyzed C−H alkynylation: expedient peptide synthesis and modification. Angew. Chem. Int. Ed..

[36] Shi L, Zhong X, She H, Lei Z, Li F (2015). Manganese catalyzed C–H functionalization of indoles with alkynes to synthesize bis/trisubstituted indolylalkenes and carbazoles: the acid is the key to control selectivity. Chem. Commun..

[37] Kuninobu Y, Nishina Y, Takeuchi T, Takai K (2007). Manganese-catalyzed insertion of aldehydes into a C-H bond. Angew. Chem. Int. Ed..

[38] van der Leun AM, Thommen DS, Schumacher TN (2020). CD8+ T cell states in human cancer: insights from single-cell analysis. Nat. Rev. Cancer.

[39] Farhood B, Najafi M, Mortezaee K (2019). CD8+ cytotoxic T lymphocytes in cancer immunotherapy: a review. J. Cell. Physiol..

[40] Zhang N, Bevan MJ (2011). CD8+ T cells: foot soldiers of the immune system. Immunity.

[41] Yin Z (2019). Targeting T cell metabolism in the tumor microenvironment: an anti-cancer therapeutic strategy. J. Exp. Clin. Cancer Res.

[42] Yang W (2016). Potentiating the antitumour response of CD8+ T cells by modulating cholesterol metabolism. Nature.

[43] Hammarback LA, Robinson A, Lynam JM, Fairlamb IJS (2019). Mechanistic insight into catalytic redox-neutral C–H bond activation involving manganese(I) carbonyls: catalyst activation, turnover, and deactivation pathways reveal an intricate network of steps. J. Am. Chem. Soc..

[44] Hammarback LA, Robinson A, Lynam JM, Fairlamb IJS (2019). Delineating the critical role of acid additives in Mn-catalysed C–H bond functionalisation processes. Chem. Commun..

[45] Ackermann L (2011). Carboxylate-assisted transition-metal-catalyzed C−H bond functionalizations: mechanism and scope. Chem. Rev..

[46] Ribas A, Wolchok JD (2018). Cancer immunotherapy using checkpoint blockade. Science.

[47] Binnewies M (2018). Understanding the tumor immune microenvironment (TIME) for effective therapy. Nat. Med..

[48] Zhou F (2011). Molecular rotors as fluorescent viscosity sensors: molecular design, polarity sensitivity, dipole moments changes, screening solvents, and deactivation channel of the excited states. Eur. J. Org. Chem..

[49] Hsu Y-P (2019). Fluorogenic D-amino acids enable real-time monitoring of peptidoglycan biosynthesis and high-throughput transpeptidation assays. Nat. Chem..

