# Supplementary material for: Chemodivergent manganese-catalyzed C–H activation: modular synthesis of fluorogenic probes
Source: Nat Commun. 2021 Jun 7;12:3389. doi: 10.1038/s41467-021-23462-9 (PMC8185085; doi:10.1038/s41467-021-23462-9)
Supplement: Supplementary file 1 — Supplementary Information [file 41467_2021_23462_MOESM1_ESM.pdf]

Supplementary Information

Chemodivergent Manganese-Catalyzed C–H Activation: Modular Synthesis of Fluorogenic Probes

Nikolaos Kaplaneris,[1, 4] Jongwoo Son,[1, 4] Lorena Mendive-Tapia,[2, 4] Adelina Kopp,[1] Nicole D. Barth,[2] Isaac Maksso,[1] Marc Vendrell,*[2] Lutz Ackermann*[1, 3]

[1] Institut für Organische und Biomolekulare Chemie,
Georg-August-Universität Göttingen,
Tammannstraße 2, 37077, Göttingen, Germany,

[2] Centre for Inflammation Research,
The University of Edinburgh,
Little France Crescent 47, EH16 4TJ, Edinburgh, UK

[3] German Centre for Cardiovascular Research (DZHK).
Potsdamer Straße 58, 10785, Berlin, Germany.

[4] These authors contributed equally to this work

E-mail: Marc.Vendrell@ed.ac.uk
E-mail: Lutz.Ackermann@chemie.uni-goettingen.de
Homepage: http://www.ackermann.chemie.uni-goettingen.de

Contents

General Remarks **S1**
Synthesis of BODIPY-alkynes **S2**
Optimization Studies for the Manganese-Catalyzed C–H Labelling **S4**
General Procedure C: Manganese-Catalyzed C–H Alkynylation of Peptides **S6**
General Procedure D: Manganese-Catalyzed C–H Hydroarylation of Peptides **S6**
ICP-MS Analysis **S6**
Manganese-Catalyzed C–H Hydroarylation of 1 in Flow: In Line Manganese Scavenging **S6**
In vitro Spectral Measurements **S7**
Liposome Preparation **S7**
Cell Culture **S8**
Flow Cytometry Analysis **S8**
Fluorescence Confocal Microscopy **S9**
Characterization Data **S10**
Studies on Potential Racemization **S77**
Supplementary Figures and Table **S79**
NMR Spectra **S84**
Supplementary References **S210**

General Remarks

Catalytic reactions were carried out in Schlenk tubes under N_2. 1,4-Dioxane and PhMe were dried over Na and freshly distilled under N_2. DCE was dried over CaH_2 and freshly distilled under N_2. The following substrate Boc-Trp^{py}-OH was synthesized according to previously described methods.[1] Peptides were synthesized under standard solution phase protocols (EDCI/HOBt). Other chemicals were obtained from commercial sources and were used without further purification. Yields refer to isolated compounds, estimated to be >95% pure as determined by ^{1}H NMR. Chromatography: Merck silica gel 60 (40-63 µm). NMR: Spectra were recorded on Bruker Avance 300, Avance III 400, Avenace III HD 400, Varian Unity 300, Inova 500 or Inova 600 in the solvent indicated; chemical shifts (δ) are given in ppm. All IR spectra were recorded on a Bruker FT-IR Alpha device. MS: EI-MS-was recorded on Jeol AccuTOF at 70 eV and ESI-MS was recorded on Bruker microTOF and maXis. M.p.: Stuart melting point apparatus SMP3, Barloworld Scientific, values are uncorrected. Flow: Vapourtec E-Series User Manual-Rev 3.4. ICP-MS: Thermo Scientific™ iCAP™ RQ ICP-MS.

Synthesis of BODIPY-alkynes

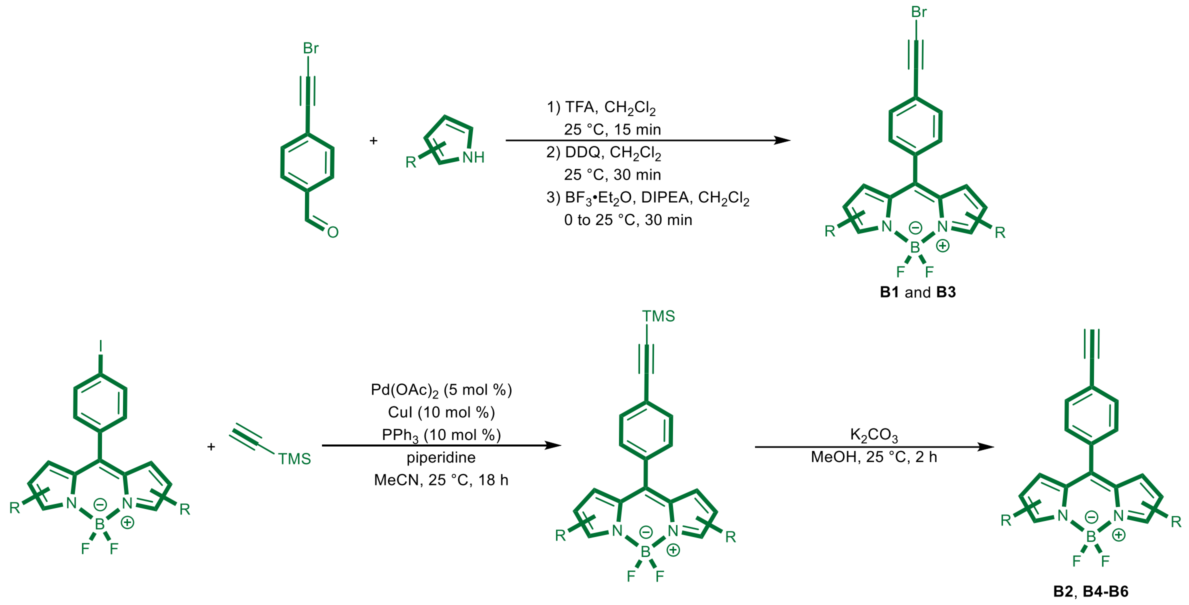

Supplementary Figure 1. Synthetic route for BODIPY-alkynes **B**.

General Procedure A: Synthesis of BODIPY-Bromoalkynes B1, B3

Pyrrole derivative (2.0 equiv) and 4-(bromoethynyl)benzaldehyde (1.0 equiv) were dissolved in CH_2Cl_2 (0,1 M with respect to the aldehyde) and stirred for 5 min at 25 °C, under nitrogen atmosphere. Then, trifluoroacetic acid (0.05 mol %) and the mixture is stirred for 5 min. The solution was diluted with CH_2Cl_2 and washed with H_2O (3 times), dried over Na_2SO_4 and the solution was concentrated *in vacuo* (The product was used without further purification).

The obtainted product was dissolved dissolved in CH_2Cl_2 (0,1 M) under nitrogen atmosphere. Then, dichloro-5,6-dicyano-*p*-benzoquinone (1.1 equiv) was added and the mixture was stirred for 30 min at at 25 °C. Then, the mixture was cooled to 0 °C with an ice-bath. Then, N,N-diisopropylethylamine (10 equiv) was added and the mixture was stirred at 0 °C for 30 min. Then, BF_3•Et_2O (12 equiv) was added and the mixture was stirred for 0 °C for 5 min, at which point the ice-bath was removed the mixture was stirred at 25 °C for 30 min. Then, the mixture was diluted with CH_2Cl_2 and washed with *sat.* $NaHCO_3$ (3 times) and *sat.* NaCl, dried over Na_2SO_4 and the solution was concentrated *in vacuo.* Purification by column chromatography on silica gel afforded the desired products **B**.

General Procedure B: Synthesis of BODIPY-Alkynes B2, B4-B6

A suspension of iodo-BODIPY (1.0 equiv), trimethylsilylacetylene (1.5 equiv), $Pd(OAc)_2$ (5.0 mol %), CuI (10 mol %), PPh_3 (10 mol %), and piperidine (3 equiv, degassed) in MeCN (0.1 M, degassed) was stirred at 25 °C for 18 h. After, CH_2Cl_2 was added and the mixture was concentrated *in vacuo*. Purification by column chromatography on silica gel afforded the desired TMS-BODIPY-alkynes.

A suspension of TMS-BODIPY-alkyne and K_2CO_3 (10 mol %) in MeOH (0,1 M) was stirred at 25 °C for 2-24 h. Then, the solvent was concentrated *in vacuo*, the mixture was redissolved in CH_2Cl_2 and washed with *sat.* $NaHCO_3$ (3 times) and *sat.* NaCl, dried over Na_2SO_4 and the solution was concentrated *in vacuo* Purification by column chromatography on silica gel afforded the desired products **B**.

Optimization Studies for the Manganese-Catalyzed C–H Labelling

Supplementary Table 1. Optimization of the Manganese(I)-Catalyzed C–H Alkynylation of Tryptophan **1**.[a]

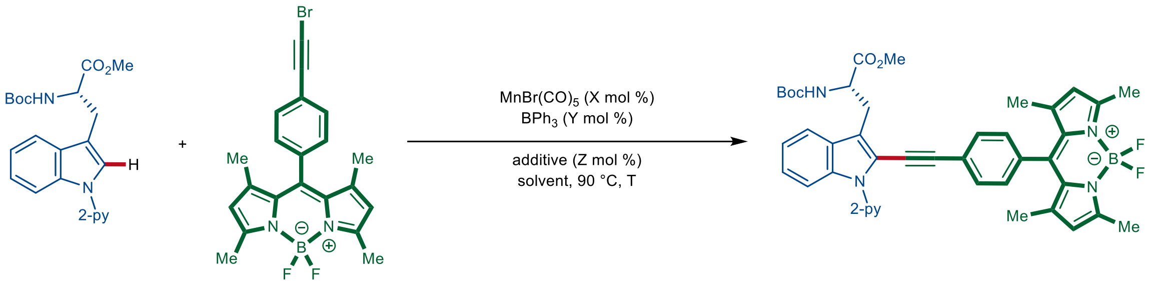

1 **B1** **2**

Entry	X	Solvent	Y	Additive (Z)	Time	Yield [%][b]
1	5	DCE	0.05	Cy_2NH (1.0)	18 h	33
2	10	DCE	5.0	Cy_2NH (1.0)	18 h	51
3	10	DCE	5.0	KOAc (2.0)	18 h	67
4	10	1,4-dioxane	5.0	KOAc (2.0)	18 h	71
**5**	**10**	**1,4-dioxane**	**0.50**	**KOAc (2.0)**	**24 h**	**75**
6	10	1,4-dioxane	0.50	KOAc (2.0)	24 h	70[c]
7	10	1,4-dioxane	-	KOAc (2.0)	24 h	---
8	10	1,4-dioxane	0.05	KOAc (2.0)	18 h	47
9	10	DCE	0.5	KOAc (2.0)	24 h	61
10	10	1,4-dioxane	0.5	-	24 h	---
11	10	1,4-dioxane	5.0	KOAc (2.0)	3 h	59

[a] Reaction conditions: **1** (0.10 mmol), **B1** (0.11 mmol), $MnBr(CO)_5$, additive, BPh_3, solvent (1.0 mL). [b] Yields of isolated product. [c] Reaction at 70 °C.

Supplementary Table 2. Optimization of the Manganese(I)-Catalyzed C–H Hydroarylation of Tryptophan **1**.[a]

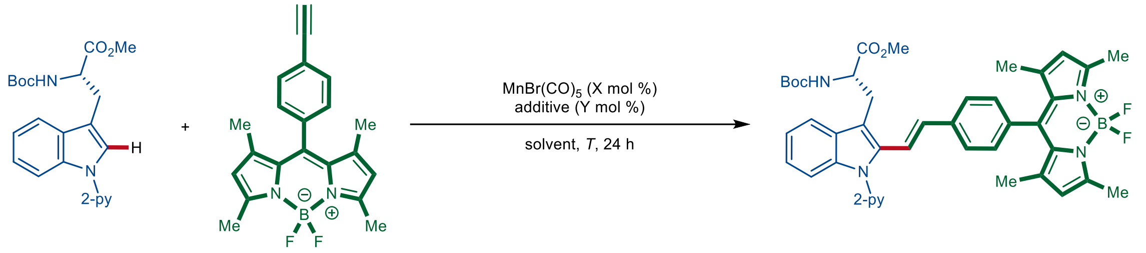

1 **B2** **3**

Entry	X	Solvent	Additive (Y mol %)	*T* []	Yield [%][b]
1	10	Et_2O	DIPEA (20)<br>$PhCO_2H$ (20)	80	---
2	20	1,4-dioxane	AcOH (40)	100	46
3	20	1,4-dioxane	PivOH (40)	100	54
4	20	1,4-dioxane	Cy_2NH (40)	100	38
**5**	**20**	**1,4-dioxane**	**(1-Ad)CO_2H (40)**	**100**	**88**
6	20	1,4-dioxane	(1-Ad)CO_2H (40)	80	83[c]
7	20	1,4-dioxane	(1-Ad)CO_2H (40)	60	80[c]
8	20	1,4-dioxane	(1-Ad)CO_2H (40)	37	52[c]
9	10	1,4-dioxane	(1-Ad)CO_2H (40)	100	60[c]
10	20	1,4-dioxane:H_2O (7/3)	(1-Ad)CO_2H (40)	100	85
11	20	1,4-dioxane:H_2O (7/3)	(1-Ad)CO_2H (40)	80	77

[a] Reaction conditions: **1** (0.10 mmol), **B2** (0.10 mmol), $MnBr(CO)_5$, additive, solvent (1.0 mL), 24 h. [b] Yields of isolated product. [c] **B2** (0.11 mmol).

General Procedure C: Manganese-Catalyzed C–H Alkynylation of Peptides

A suspension of peptide **4** (0.10 mmol, 1.00 equiv), BODIPY-bromoalkyne **B** (0.11 mmol, 1.1 equiv), $MnBr(CO)_5$ (2.7 mg, 10 mol %), KOAc (19.6 mg, 2.0 equiv) and BPh_3 (50 µL, 0.01 M in 1,4-dioxane, 0.5 mol %) in 1,4-dioxane (1.0 mL) was stirred at 90 °C for 24 h. After cooling to ambient temperature, CH_2Cl_2 (10 mL) was added and the mixture was concentrated *in vacuo*. Purification by column chromatography on silica gel afforded the desired products **2**, **6-18**.

General Procedure D: Manganese-Catalyzed C–H Hydroarylation of Peptides

A suspension of peptide **4** (0.10 mmol, 1.0 equiv), BODIPY-alkyne **B** (0.11-0.20 mmol, 1.1 -2.0 equiv), $MnBr(CO)_5$ (5.5 mg, 20 mol %) and (1-Ad)CO_2H (7.2 mg, 40 mol %) in 1,4-dioxane (1.0 mL) was stirred at 100 °C for 24 h. After cooling to ambient temperature, CH_2Cl_2 (10 mL) was added and the mixture was concentrated *in vacuo*. Purification by column chromatography on silica gel afforded the desired products **3**, **20-44** and **47-59**.

ICP-MS Analysis

Amino acid **3** (5.0 – 15.0 mg) was mixed with HNO_3/HCl (1/3, 1.2 mL, trace metal grade) and sonicated at 60 ºC for 3 h. Then distilled H_2O (8.8 mL) was added and the solution was sonicated for 10 min. The solution was centrifuged (20 min, 11180 g) and then analyzed with Thermo Scientific™ iCAP™ RQ.

Manganese-Catalyzed C–H Hydroarylation of 1 in Flow: In Line Manganese Scavenging

A 5 mL oven-dried pear-shaped flask was charged with methyl N_a-(*tert*-butoxycarbonyl)-1-(pyridin-2-yl)-*L*-tryptophanate (**1**) (39.5 mg, 0.10 mmol), 10-(4-ethynylphenyl)-5,5-difluoro-1,3,7,9-tetramethyl-5*H*-4λ_4,5λ_4-dipyrrolo[1,2-*c*:2',1'-*f*][1,3,2]diazaborinine (**B2**) (38.3 mg, 0.11 mmol), $MnBr(CO)_5$ (5.5 mg, 20 mol %) and (1-Ad)CO_2H (7.2 mg, 40 mol %) and 1,4-dioxane (2.0 mL) under air. Subsequently, the reaction media was connected to the inlet of the 10 mL standard heated reactor connected with a column reactor (Ø = 8 mm) packed with 250 mg of QuadraPure™ IDA over 2 g of sand. The syringe pump was operated at a flow rate of 100µL / min

(100 min residence time). The temperature of the standard heated reactor was set at 100 °C. By using the Flow Wizard system, the solution was collected automatically. To ensure full transfer of the crude mixture to the collection flask EtOAc (20 mL) was pumped through the reactor at ambient temperature. Next, the mixture was concentrated *in vacuo*. Purification by column chromatography on silica gel (CH_2Cl_2/MeOH: 100/0.2) yielded **3** (46.0 mg, 62%) as an orange solid. The obtained solid was analyzed by inductively coupled plasma mass spectrometry (ICP-MS) analysis, showing 6.1 ppm of residual manganese.

In vitro Spectral Measurements

Spectral properties were recorded in 96-well black plates on a Synergy HT spectrophotometer. Compounds were dissolved at the indicated concentrations and spectra were recorded at r.t. For extinction coefficient measurements, the absorbance of each sample at the maximum excitation wavelength was recorded and the extinction coefficient was then determined by fitting the data to Beer's law. To measure viscosity sensitivity, amino acids were diluted in glycerol-water solutions (0%, 20%, 40%, 50% and 60% glycerol, v/v) to a final concentration of 25 µM. Their fluorescence intensity was measured at the maximum emission wavelength with excitation at the maximum excitation wavelength. The viscosity sensitivity was then calculated using the Förster–Hoffmann equation ($\log I = C + x \log \eta$), where η is the dynamic viscosity of the solvent mixture, I is the emission intensity of the amino acid, c is a constant depending on the temperature, and x is the viscosity sensitivity of the dye. The dynamic viscosities of glycerol-water solutions were calculated based on the previous reports.[2] Quantum yields were referenced to fluorescein in basic EtOH.[3]

Liposome Preparation

2 mM lipid stock solutions of pure DMPC or cholesterol were diluted in chloroform: methanol in 2:1 ratio. The appropriate volume of each lipid stock was transferred into a dried round-bottom flask, which was then set on a rotary evaporator at 40 ºC (above the phase transition temperature for DMPC) to concentrate to dryness. As the solvent started to evaporate, a thin film of dry lipid was formed on the interior walls of the flask. Once the solvent was completely evaporated, the film was processed with N_2 so that any excessive solvent residues were removed. The resulting

film was hydrated with DPBS buffer solution (previously warmed at 40 ºC) and kept for annealing in a 40 ºC water bath for 1 h with constant rotation.

Cell Culture

Ethical approval was received by the Accredited Medical Regional Ethics Committee (AMREC, reference number 20-HV-069). Work with human CD8+ T cells complied with all relevant ethical regulations and informed consent was obtained before study commenced. Primary CD8+ T cells were freshly isolated from human whole blood from healthy donors by immunomagnetic negative selection (EasySep™ Direct Human CD8+ T Cell Isolation Cocktail, STEMCELL Technologies) and were incubated using Dulbecco's Modified Eagle Medium (DMEM) supplemented with 20% fetal bovine serum (FBS), antibiotics (100 U mL^{-1} penicillin and 100 mg mL^{-1} streptomycin), 1 % non-essential amino acids, 1 mM sodium pyruvate, 50 nM 2-mercaptoethanol and 2 mM L-glutamine. The day of the experiment, 10^5 CD8+T cells/well were seeded in a 96 well-plate in DMEM media containing recombinant human IL-2 (80 U mL^{-1}), human anti-CD3 (2 μg mL^{-1}) and human anti-CD28 (5 μg mL^{-1}). After two days of incubation at 37 ºC with 5% CO_2, IL-2 was added to the wells (1000 U mL^{-1}) which were further incubated for 7 h prior flow cytometry analysis. When indicated, avasimibe was added 12 h before addition of IL-2 (1000 U mL^{-1}) at the indicated concentrations. Jurkat cells obtained from ATCC were cultured in RPMI-1640 media in a humidified atmosphere at 37 ºC with 5% CO_2 and were regularly passaged in T-25 cell culture flasks. The day of the experiment, 1.5×10^5 Jurkat cells/well were seeded in 96 well-plates and incubated with the drugs at the indicated concentrations. 12 h after the drug addition, cells were incubated with recombinant human IL-2 (1000 U mL^{-1}), human anti-CD3 (2 μg mL^{-1}) and human anti-CD28 (4 μg mL^{-1}) for 7 h prior flow cytometry analysis. For analysis of intracellular cytokines, Protein Transport Inhibitor (containing Brefeldin A) (BD GolgiPlugTM) was added at 1:1000 simultaneously with stimuli.

Flow Cytometry Analysis

Dyes were reconstituted at 10 mM in DMSO. 10^5 cells were stained with compound **41** (1 μM) in HEPES-NaCl containing 0.1% BSA for 1 h at r.t. prior to staining with antibodies. Cells were washed with HEPES-NaCl followed by staining with anti-human PD-1-PE (10 μg mL^{-1}), anti-

human CD62L-PE/Cy7 (10 μg mL^{-1}) and anti-human CD8-PerCP/Cy5.5 (5 μg mL^{-1}) in HEPES-NaCl containing 0.1% BSA and 1% FcR block for 20 min on ice. Cells were washed at 300 g, 5 min in HEPES-NaCl prior to staining with 25 nM Annexin V-PB in HEPES-NaCl containing 0.1% BSA and 2 mM $CaCl_2$. Dyes were reconstituted at 10 mM in DMSO. 10^5 cells were stained with compound **41** (1 μM) in HEPES-NaCl containing 0.1% BSA for 1 h at r.t. prior to staining with antibodies. Cells were washed with HEPES-NaCl followed by staining with anti-human PD-1-PE (1:100, 10 μg mL^{-1}), anti-human CD62L-PE/Cy7 (1:200, 10 μg mL^{-1}), CD39-APC (1:200, 5 μg mL^{-1}), CD3-PE-CF594 (1:200, 5 μg mL^{-1}), CD86-ef450 (1:200, 10 μg mL^{-1}), and anti-human CD8-PerCP/Cy5.5 (1:200, 0.4 μg mL^{-1}) in HEPES-NaCl containing 0.1% BSA and 1% FcR block for 20 min on ice. Cells were washed at 300 g, 5 min in HEPES-NaCl prior to staining with 25 nM Annexin V-PB in HEPES-NaCl containing 0.1% BSA and 2 mM CaCl2. For intracellular cytokine staining, cells were washed once in HEPES-NaCl prior to permeabilization with BD Cytofix/Cytoperm™ according to the manufacturer for 20 min on ice. Followed by staining with Granzyme B-APC (1:100, 1.5 μg mL^{-1}), Granzyme A-AF488 (1:100, 1 μg mL^{-1}) and IFNγ-APC/Cy7 (1:100, 1.26 μg mL^{-1}) in Perm/Wash Buffer for 20 min on ice. Stained cells were washed once in Perm/Wash Buffer prior to resuspension in Perm/Wash Buffer for flow cytometric assessment. Fluorescence emission was measured on a 5L LSR flow cytometer. Excitation sources/emission filters used: Annexin V-PB (355 nm, 450/50 nm), **41** (488 nm, 510/20 nm), PD1-PE (561 nm, 582/15 nm), CD62L-PE/Cy7 (561 nm, 780/60 nm), CD8-PerCP/Cy5.5 (488 nm, 710/50 nm), Granzyme B-APC (647 nm, 670/14 nm), CD39-APC, (647 nm, 6701/14 nm), CD3-PE-CF594 (561 nm, 610/20 nm), IFNγ-APC/Cy7 (647 nm, 780/60 nm), Granzyme A-AF488 (488 nm, 510/20 nm), CD86-ef450 (405 nm, 450/50 nm).

Fluorescence Confocal Microscopy

Confocal imaging was performed in a TCS Leica SP8 confocal laser scanning microscope. Prior to imaging, cells were stained with compound **41** (1 μM) for 1 h at r.t. in HEPES-NaCl containing 0.1% BSA. Cells were further counterstained with the nuclear counterstain Hoechst 33342 (3 μM) for 10 min at r.t.. Excitation/emission wavelengths: Hoechst 33342 (355 nm, 450/50 nm), **41** (488 nm, 520/40 nm). Images were processed with ImageJ.

Characterization Data

10-[4-(Bromoethynyl)phenyl-5,5-difluoro-1,3,7,9-tetramethyl-5*H*-4λ_4,5λ_4-dipyrrolo[1,2-*c*:2',1'-*f*][1,3,2]diazaborinine (B1)

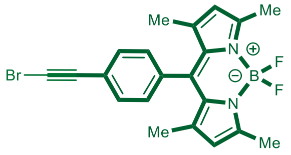

Following the general procedure **A**, on 5.0 mmol scale, **B1** was obtained as a red solid (44%, over 3 steps).

M. p.: 236 – 239 ºC.

^{1}H NMR (400 MHz, $CDCl_3$): δ 7.61 (d, J = 7.7 Hz, 2H), 7.28 (d, J = 7.7 Hz, 2H), 6.01 (s, 2H), 2.58 (s, 6H), 1.42 (s, 6H).

^{13}C NMR (101 MHz, $CDCl_3$): δ 155.8 (C_q), 142.9 (C_q), 140.5 (C_q), 135.5 (C_q), 132.7 (CH), 131.1 (C_q)128.3 (CH), 123.6 (C_q), 121.4 (CH), 79.4 (C_q), 51.6 (C_q), 14.6 (CH_3), 14.6 (CH_3).

^{19}F NMR (377 MHz, $CDCl_3$): δ – 146.3 (q, $^{1}J_{B\text{-}F}$ = 32.8 Hz).

IR (ATR): 2926, 1544, 1510, 1467, 1410, 1310, 1201, 1087, 970 cm^{-1}.

MS (ESI) m/z (relative intensity): 449 (90 $[M+Na]^+$, 427 (100) $[M+H]^+$.

HR-MS (ESI) m/z calcd for $C_{21}H_{19}B^{79}BrF_2N_2$ $[M+H]^+$:427.0791, found: 427.0789.

10-(4-Ethynylphenyl)-5,5-difluoro-1,3,7,9-tetramethyl-5*H*-4λ_4,5λ_4-dipyrrolo[1,2-*c*:2',1'-*f*][1,3,2]diazaborinine (B2)

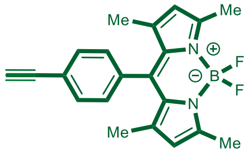

Following the general procedure **B**, on 5.0 mmol scale, **B2** was obtained as a red solid (54%, over 2 steps). The data are in agreement with the literature.[4]

M. p.: 223 – 227 ºC.

^{1}H NMR (400 MHz, $CDCl_3$): δ 7.65 (d, J = 7.9 Hz, 2H), 7.29 (d, J = 7.9 Hz, 2H), 6.01 (s, 2H), 3.21 (s, 1H), 2.58 (s, 6H), 1.42 (s, 6H).

^{13}C NMR (101 MHz, $CDCl_3$): δ 155.8 (C_q), 142.9 (C_q), 140.5 (C_q), 135.5 (C_q), 132.8 (CH), 131.1 (C_q), 128.1 (CH), 122.9 (C_q), 121.3 (CH), 82.8 (C_q), 78.5 (CH), 14.5 (CH_3), 14.5 (CH_3).

^{19}**F NMR** (377 MHz, $CDCl_3$): δ – 146.3 (q, $^1J_{B-F}$ = 32.2 Hz).
IR (ATR): 3256, 2922, 1554, 1510, 1412, 1202, 1155, 1039, 971 cm^{-1}.
MS (ESI) m/z (relative intensity): 371 (100) $[M+Na]^+$, 349 (60) $[M+H]^+$.
HR-MS (ESI) m/z calcd for $C_{21}H_{20}BF_2N_2$ $[M+H]^+$:349.1686, found: 349.1686.

10-[4-(Bromoethynyl)phenyl]-5,5-difluoro-3,7-bis(4-methoxyphenyl)-5*H*-4λ_4,5λ_4-dipyrrolo[1,2-*c*:2',1'-*f*][1,3,2]diazaborinine (B3)

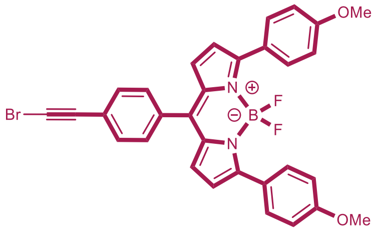

Following the general procedure **A**, on 5.0 mmol scale, **B3** was obtained as a purple solid (50%, over 3 steps).
M. p.: 223 – 225 ºC.
^{1}**H NMR** (400 MHz, $CDCl_3$): δ 7.91 (d, J = 8.5 Hz, 4H), 7.62 (d, J = 8.0 Hz, 2H), 7.55 (d, J = 8.0 Hz, 2H), 6.99 (d, J = 8.5 Hz, 4H), 6.83 (d, J = 4.3 Hz, 2H), 6.64 (d, J = 4.3 Hz, 2H), 3.88 (s, 6H).
^{13}**C NMR** (101 MHz, $CDCl_3$): δ 160.8 (C_q), 158.6 (C_q), 141.1 (C_q), 136.0 (C_q), 134.9 (C_q), 131.8 (CH), 131.2 (CH), 131.2 (CH), 131.1 (CH), 130.6 (CH), 130.1 (CH), 125.1 (C_q), 124.5 (C_q), 120.7 (CH), 113.8 (CH), 79.4 (C_q), 55.3 (CH_3), 52.4 (C_q).
^{19}**F NMR** (377 MHz, $CDCl_3$): δ – 132.9 (q, $^1J_{B-F}$ = 32.3 Hz).
IR (ATR): 1602, 1555, 1518, 1462, 1253, 1133, 1051, 963, 787 cm^{-1}.
MS (ESI) m/z (relative intensity): 583 (40) $[M+H]^+$, 563 (100) $[MF]^+$.
HR-MS (ESI) m/z calcd for $C_{31}H_{23}B^{79}BrF_2N_2O_2$ $[M+H]^+$:583.1004, found: 583.0998.

10-(3-Ethynylphenyl)-5,5-difluoro-1,3,7,9-tetramethyl-5*H*-4λ_4,5λ_4-dipyrrolo[1,2-*c*:2',1'-*f*][1,3,2]diazaborinine (B4)

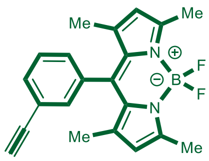

Following the general procedure **B**, on 5.0 mmol scale, **B4** was obtained as a red solid (21%, over 2 steps).

M. p.: 112 – 114 ºC.

^{1}H NMR (300 MHz, $CDCl_3$): δ 7.62 (ddd, J = 7.7, 1.5, 1,5 Hz, 1H), 7.53 – 7.43 (m, 2H), 7.30 (ddd, J = 7.7 1.5, 1,5 Hz, 1H), 6.01 (s, 2H), 3.16 (s, 1H), 2.57 (s, 6H), 1.43 (s, 6H).

^{13}C NMR (75 MHz, $CDCl_3$): δ 155.8 (C_q), 143.0 (C_q), 140.1 (C_q), 135.3 (C_q), 132.6 (CH), 131.6 (CH), 131.2 (C_q), 129.3 (CH), 128.4 (CH), 123.3 (C_q), 121.4 (CH), 82.6 (C_q), 78.6 (CH), 14.6 (CH_3), 14.6 (CH_3).

^{19}F NMR (282 MHz, $CDCl_3$): δ – 145.9 - –146.5 (m).

IR (ATR): 1661, 1588, 1487, 1236, 1136, 976, 838, 711, 652 cm^{-1}.

MS (ESI) m/z (relative intensity): 371 (100) $[M+Na]^+$, 349 (40) $[M+H]^+$.

HR-MS (ESI) m/z calcd for $C_{21}H_{20}BF_2N_2$ $[M+H]^+$:349.1686, found: 349.1685.

10-(4-Ethynylphenyl)-5,5-difluoro-3,7-diphenyl-5*H*-4λ_4,5λ_4-dipyrrolo[1,2-*c*:2',1'-*f*][1,3,2]diazaborinine (B5)

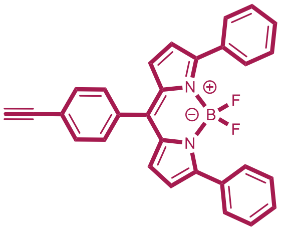

Following the general procedure **B**, on 5.0 mmol scale, **B5** was obtained as a purple solid (34%, over 2 steps).

M. p.: 169 – 171 ºC.

^{1}H NMR (400 MHz, $CDCl_3$): δ 7.98 – 7.85 (m, 4H), 7.69 (d, J = 7.9 Hz, 2H), 7.59 (d, J = 7.8 Hz, 2H), 7.50 – 7.41 (m, 6H), 6.90 (d, J = 4.2 Hz, 2H), 6.67 (d, J = 4.2 Hz, 2H), 3.28 (s, 1H).

^{13}C NMR (101 MHz, $CDCl_3$): δ 159.3 (C_q), 142.9 (C_q), 142.9 (C_q), 136.1 (C_q), 134.7 (C_q), 132.5 (CH), 132.0 (CH), 130.6 (CH), 130.6 (CH), 129.6 (CH), 129.5 (CH), 129.5 (CH), 129.4 (CH), 128.3 (CH), 124.2 (C_q), 121.2 (CH), 121.1 (CH), 82.7 (C_q), 79.5 (CH).

^{19}F NMR (282 MHz, $CDCl_3$): δ – 132.5 (q, $^1J_{B\text{-}F}$ = 31.7 Hz).

IR (ATR): 3371, 1557, 1525, 1448, 1285, 1261, 1148, 1132, 1049, 978, 754 cm^{-1}.

MS (ESI) m/z (relative intensity): 467 (100) $[M+Na]^+$, 445 (10) $[M+H]^+$.

HR-MS (ESI) m/z calcd for $C_{29}H_{20}BF_2N_2$ $[M+H]^+$:445.1687, found: 445.1680.

10-(4-Ethynylphenyl)-5,5-difluoro-3,7-di-*p*-tolyl-5*H*-4λ_4,5λ_4-dipyrrolo[1,2-*c*:2',1'-*f*][1,3,2]diazaborinine (B6)

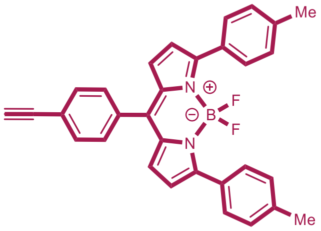

Following the general procedure **B**, on 5.0 mmol scale, **B6** was obtained as a purple solid (69%, over 2 steps).

M. p.: 125 – 126 ºC.

^{1}H NMR (400 MHz, $CDCl_3$): δ 7.82 (d, J = 8.0 Hz, 4H), 7.68 (d, J = 8.0 Hz, 2H), 7.58 (d, J = 8.0 Hz, 2H), 7.32 – 7.13 (m, 4H), 6.86 (d, J = 4.3 Hz, 2H), 6.65 (d, J = 4.3 Hz, 2H), 3.27 (s, 1H), 2.42 (s, 6H).

^{13}C NMR (101 MHz, $CDCl_3$): δ 159.2 (C_q), 142.1 (C_q), 139.8 (C_q), 136.1 (C_q), 134.9 (C_q), 132.0 (CH), 130.5 (CH), 130.4 (CH), 129.7 (C_q), 129.5 (CH), 129.4 (CH), 129.4 (CH), 129.1 (CH), 124.0 (C_q), 121.0 (CH), 82.8 (C_q), 79.3 (CH), 21.5 (CH_3).

^{19}F NMR (377 MHz, $CDCl_3$): δ – 132.7 (q, $^1J_{B\text{-}F}$ = 31.9 Hz).

IR (ATR): 3280, 1558, 1540, 1466, 1286, 1260, 1125, 1051, 962, 707 cm^{-1}.

MS (ESI) m/z (relative intensity): 495 (100) $[M+Na]^+$, 473 (20) $[M+H]^+$.

HR-MS (ESI) m/z calcd for $C_{31}H_{24}BF_2N_2$ $[M+H]^+$:473.2000, found: 473.1993.

***N$_a$*-[(*tert*-Butoxycarbonyl)-*L*-valyl]-1-(pyridin-2-yl)-*L*-tryptophan (4p)**

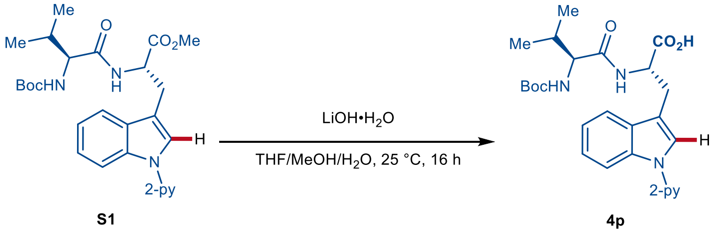

To a stirred solution of methyl *N$_a$*-[(*tert*-butoxycarbonyl)-*L*-valyl]-1-(pyridin-2-yl)-*L*-tryptophanate **S1** (494 mg, 1.0 mmol) in THF/MeOH/H_2O (5.0 mL, 2/2/1) LiOH•H_2O (84 mg, 2.0 mmol) was added and the mixture was stirred at 25 °C for 16 h. Then, EtOAc (50 mL) was added

and the solution was washed with aq. HCl (20 mL, 1 M, 3 times) and brine. The organic layer was dried over Na_2SO_4 and concentrated *in vacuo* providing **4p** (95%, 455 mg) as a white solid.

M. p.: 128 – 131 ºC.

^{1}H NMR (400 MHz, DMSO-d_6): δ 12.74 (brs, 1H), 8.61 – 8.50 (m, 1H), 8.41 (d, J = 8.2 Hz, 1H), 8.25 – 8.10 (m, 1H), 8.01 – 7.91 (m, 1H), 7.88 (s, 1H), 7.76 – 7.60 (m, 2H), 7.31 – 7.11 (m, 3H), 6.68 (d, J = 7.2 Hz, 1H), 4.71 – 4.55 (m, 1H), 3.84 (dd, J = 8.3, 7.2 Hz, 1H), 3.25 (d, J = 14.9 Hz, 1H), 3.15 (dd, J = 14.9, 8.6 Hz, 1H), 1.96 – 1.83 (m, 1H), 1.32 (s, 9H), 0.93 – 0.64 (m, 6H).

^{13}C NMR (101 MHz, DMSO-d_6): δ 173.0 (C_q), 171.5 (C_q), 155.4 (C_q), 152.2 (C_q), 148.4 (CH), 139.0 (CH), 134.8 (C_q), 129.8 (C_q), 124.6 (CH), 123.0 (CH), 121.0 (CH), 119.9 (CH), 118.8 (CH), 114.1 (CH), 114.1 (C_q), 113.8 (CH), 78.0 (C_q), 59.7 (CH), 52.0 (CH), 30.4 (CH), 28.1 (CH_3), 26.8 (CH_2), 19.1 (CH_3), 18.3 (CH_3).

IR (ATR): 3331, 2964, 1687, 1643, 1483, 1438, 1243, 1213, 1169, 1155, 735 cm^{-1}.

MS (ESI) m/z (relative intensity): 503 (60) $[M+Na]^+$, 481 (100) $[M+H]^+$.

HR-MS (ESI) m/z calcd for $C_{26}H_{33}N_4O_5$ $[M+H]^+$:481.2445, found: 481.2443.

Methyl *N*$_{\alpha}$-{[(benzyloxy)carbonyl]-*L*-lysyl}-1-(pyridin-2-yl)-*L*-tryptophanate (4r)

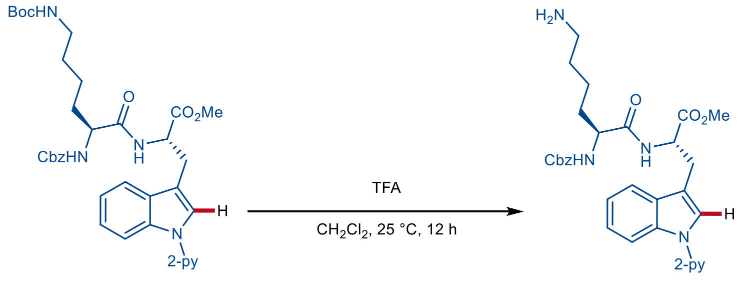

To a stirred solution of methyl *N*$_{\alpha}$-{*N*$_2$-[(benzyloxy)carbonyl]-*N*$_6$-[*tert*-butoxycarbonyl]-*L*-lysyl}-1-(pyridin-2-yl)-*L*-tryptophanate **S2** (657 mg, 1.0 mmol) in CH_2Cl_2 (3.0 mL) trifluoroacetic acid (1.0 mL) was added. The solution was stirred at 25 °C for 12 h. Then, the solution was concentrated *in vacuo* and the residue was dissolved in EtOAc (50 mL). The solution was washed with sat $NaHCO_3$ (20 mL, 3 times) and brine. The organic layer was dried over Na_2SO_4 and concentrated *in vacuo*. Purification by column chromatography on silica gel (CH_2Cl_2/MeOH: 100/0 →85/15, NEt_3 2%) yielded **4r** (475 mg, 85%) as a white solid.

M. p.: 114 – 116 ºC.

^{1}H NMR (400 MHz, DMSO-d_6): δ 8.61 – 8.53 (m, 1H), 8.50 – 8.38 (m, 2H), 7.93 (dd, J = 8.0, 8.0 Hz, 1H), 7.86 (s, 1H), 7.72 (d, J = 8.4 Hz, 1H), 7.60 (d, J = 7.8 Hz, 1H), 7.43 (d, J = 7.3 Hz, 1H), 7.37 – 7.29 (m, 4H), 7.29 – 7.23 (m, 2H), 7.23 – 7.17 (m, 2H), 4.99 (d, J = 12.7 Hz, 1H), 4.92 (d, J = 12.7 Hz, 1H), 4.66 (q, J = 6.9 Hz, 1H), 4.05 (q, J = 7.5 Hz, 1H), 3.61 (s, 3H), 3.47 – 3.08 (m, 4H), 2.76 – 2.61 (m, 2H), 1.75 – 1.18 (m, 6H).
^{13}C NMR (101 MHz, DMSO-d_6): δ 172.7 (C_q), 172.4 (C_q), 156.4 (C_q), 152.6 (C_q), 148.9 (CH), 139.5 (CH), 137.4 (C_q), 135.2 (C_q), 130.1 (C_q), 128.8 (CH), 128.3 (CH), 128.2 (CH), 125.1 (CH), 123.6 (CH), 121.5 (CH), 120.5 (CH), 119.0 (CH), 114.7 (CH), 114.4 (CH), 114.2 (C_q), 65.8 (CH_2), 54.8 (CH), 52.8 (CH), 52.4 (CH_3), 46.2 (CH_2), 31.7 (CH_2), 27.9 (CH_2), 27.0 (CH_2), 22.8 (CH_2).
IR (ATR): 3312, 1689, 1670, 1642, 1525, 1473, 1436, 1245, 1201, 1124, 740 cm^{-1}.
MS (ESI) m/z (relative intensity): 1115 (10) $[2M+H]^+$, 558 (100) $[M+H]^+$.
HR-MS (ESI) m/z calcd for $C_{31}H_{36}N_5O_5$ $[M+H]^+$:558.2711, found: 558.2713.

(3*S*,6*S*,9*S*,12*S*)-6-Benzyl-3-isobutyl-9-methyl-12-{[1-(pyridin-2-yl)-1*H*-indol-3-yl]methyl}-1,4,7,10,13-pentaazacyclopentadecane-2,5,8,11,14-pentaone (4al)

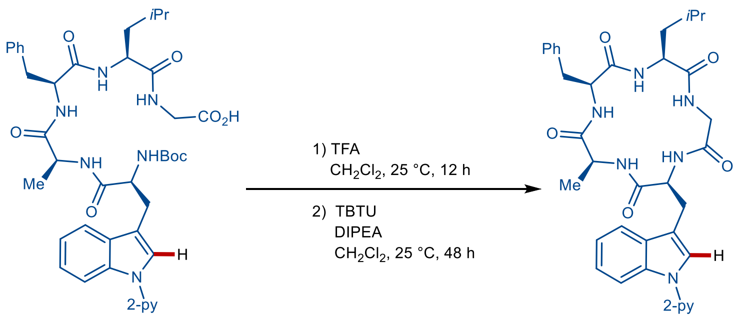

To a stirred solution of N_a-(*tert*-butoxycarbonyl)-1-(pyridin-2-yl)-*L*-tryptophyl-*L*-alanyl-*L*-phenylalanyl-*L*-leucylglycine **S3** (769 mg, 1.0 mmol) in CH_2Cl_2 (3.0 mL) trifluoroacetic acid (1.0 mL) was added. The solution was stirred at 25 °C for 12 h. Then, the solution was concentrated *in vacuo* and residual trifluoroacetic acid was azeotropically removed by co-evaporation with toluene (2 mL, 3 times,). The obtained solid was dissolved in DMF (30 mL) and added dropwise to a solution of TBTU (963 mg, 3.0 mmol) and DIPEA (775 mg,6 mmol) in CH_2Cl_2/DMF (40/1, 1000 mL) over the course of 30 min at 25 °C, and allowed to stir for 48 h. Then, the solvent was evaporated *in vacuo* and the residue was dissolved in EtOAc (500 mL). The solution was washed with aq. HCl (, 100 mL, 1 M,3 times), aq. sat. $NaHCO_3$ (100 mL, 1 times) and brine. The organic

layer was dried over Na_2SO_4 and concentrated *in vacuo*. Purification by column chromatography on silica gel (CH_2Cl_2/MeOH: 100/0 →85/15) yielded **4al** (164 mg, 25%) as a white solid.

M. p.: 263 ºC (decomposition).

^{1}H NMR (400 MHz, DMSO-d_6): δ 8.74 – 8.65 (m, 1H), 8.63 – 8.50 (m, 1H), 8.47 – 8.27 (m, 2H), 8.18 (d, J = 7.6 Hz, 1H), 8.06 – 7.92 (m, 2H), 7.88 (s, 1H), 7.81 (d, J = 7.9 Hz, 1H), 7.78 – 7.56 (m, 2H), 7.35 – 7.11 (m, 8H), 4.53 – 4.41 (m, 1H), 4.32 – 4.14 (m, 2H), 4.13 – 4.03 (m, 1H), 4.00 – 3.85 (m, 1H), 3.34 – 3.19 (m, 2H), 3.21 – 2.97 (m, 3H), 1.62 – 1.33 (m, 2H), 1.31 – 1.06 (m, 4H), 1.10 – 0.48 (m, 6H).

^{13}C NMR (101 MHz, DMSO-d_6): δ 172.9 (C_q), 171.6 (C_q), 171.1 (C_q), 170.5 (C_q), 169.5 (C_q), 152.0 (C_q), 148.5 (CH), 139.2 (CH), 137.7 (C_q), 134.8 (C_q), 129.7 (C_q), 129.0 (CH), 128.2 (CH), 126.4 (CH), 124.5 (CH), 123.1 (CH), 121.0 (CH), 120.1 (CH), 118.9 (CH), 114.4 (C_q), 114.0 (CH), 113.9 (CH), 57.7 (CH), 54.3 (CH), 51.1 (CH), 49.3 (CH), 43.5 (CH_2), 40.6 (CH_2), 35.9 (CH_2), 26.7 (CH_2), 24.4 (CH), 23.0 (CH_3), 21.7 (CH_3), 17.1 (CH_3).

IR (ATR): 3323, 2926, 1672, 1656, 1632, 1522, 1471, 1453, 1436, 733 cm^{-1}.

MS (ESI) m/z (relative intensity): 674 (80) $[M+Na]^+$, 652 (100) $[M+H]^+$.

HR-MS (ESI) m/z calcd for $C_{36}H_{42}N_7O_5$ $[M+H]^+$:652.3242, found: 652.3242.

(3*S*,6*S*,9*S*,12*S*)-6-benzyl-3-((*S*)-*sec*-butyl)-9-methyl-12-{[1-(pyridin-2-yl)-1*H*-indol-3-yl]methyl}-1,4,7,10,13-pentaazacyclopentadecane-2,5,8,11,14-pentaone (4am)

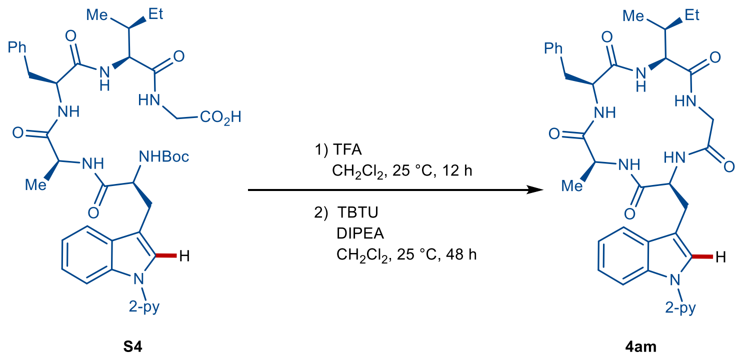

To a stirred solution of N_{α}-(*tert*-butoxycarbonyl)-1-(pyridin-2-yl)-*L*-tryptophyl-*L*-alanyl-*L*-phenylalanyl-*L*-isoleucylglycine **S4** (769 mg, 1.0 mmol) in CH_2Cl_2 (3.0 mL) trifluoroacetic acid (1.0 mL) was added. The solution was stirred at 25 °C for 12 h. Then, the solution was concentrated *in vacuo* and residual trifluoroacetic acid was azeotropically removed by co-evaporation with

toluene (2 mL, 3 times,). The obtained solid was dissolved in DMF (30 mL) and added dropwise to a solution of TBTU (963 mg, 3.0 mmol) and DIPEA (775 mg,6 mmol) in CH_2Cl_2/DMF (20/1, 1000 mL) over the course of 30 min at 25 °C, and allowed to stir for 48 h. Then, the solvent was evaporated *in vacuo* and the residue was dissolved in EtOAc (500 mL). The solution was washed with aq. HCl (, 100 mL, 1 M,3 times), aq. sat. $NaHCO_3$ (100 mL, 1 times) and brine. The organic layer was dried over Na_2SO_4 and concentrated *in vacuo*. Purification by column chromatography on silica gel (CH_2Cl_2/MeOH: 100/0 →85/15) yielded **4am** (213 mg, 33%) as a white solid.

M. p.: 255 ºC (decomposition).

^{1}H NMR (400 MHz, DMSO-d_6): δ 8.97 (brs, 1H), 8.63 (d, J = 8.2 Hz, 1H), 8.57 (d, J = 4.8 Hz, 1H), 8.42 – 8.28 (m, 2H), 8.07 – 7.95 (m, 1H), 7.88 (s, 1H), 7.83 (d, J = 9.1 Hz, 1H), 7.70 – 7.60 (m, 3H), 7.36 – 7.12 (m, 8H), 4.49 – 4.41 (m, 1H), 4.27 (t, J = 7.4 Hz, 1H), 4.12 (t, J = 8.4 Hz, 1H), 3.97 (d, J = 8.0 Hz, 1H), 3.89 (dd, J = 13.9, 5.4 Hz, 1H), 3.33 – 3.17 (m, 3H), 3.16 – 3.04 (m, 2H), 1.74 – 1.61 (m, 1H), 1.43 (ddd, J = 12.4, 7.7, 3.7 Hz, 1H), 1.21 (d, J = 7.1 Hz, 3H), 1.14 – 0.97 (m, 1H), 0.91 – 0.75 (m, 6H).

^{13}C NMR (101 MHz, DMSO-d_6): δ 173.2 (C_q), 172.0 (C_q), 171.8 (C_q), 170.8 (C_q), 169.9 (C_q), 152.5 (C_q), 149.0 (CH), 139.7 (CH), 138.2 (C_q), 135.3 (C_q), 130.1 (C_q), 129.4 (CH), 128.7 (CH), 126.9 (CH), 125.1 (CH), 123.6 (CH), 121.4 (CH), 120.5 (CH), 119.3 (CH), 115.1 (C_q), 114.4 (CH), 114.3 (CH), 59.4 (CH), 57.1 (CH), 54.8 (CH), 49.4 (CH), 44.2 (CH_2), 37.5 (CH), 36.2 (CH_2), 27.2 (CH_2), 24.9 (CH_2), 17.5 (CH_3), 16.0 (CH_3), 11.4 (CH_3).

IR (ATR): 3328, 2964, 2926, 1672, 1655, 1632, 1529, 1471, 1453, 732 cm^{-1}.

MS (ESI) m/z (relative intensity): 674 (90) $[M+Na]^+$, 652 (100) $[M+H]^+$.

HR-MS (ESI) m/z calcd for $C_{36}H_{42}N_7O_5$ $[M+H]^+$:652.3242, found: 652.3238.

Methyl (*S*)-2-[(*tert*-butoxycarbonyl)amino]-3-(2-{[4-(5,5-difluoro-1,3,7,9-tetramethyl-5*H*-4λ_4,5λ_4-dipyrrolo[1,2-*c*:2',1'-*f*][1,3,2]diazaborinin-10-yl)phenyl]ethynyl}-1-(pyridin-2-yl)-1*H*-indol-3-yl)propanoate (2)

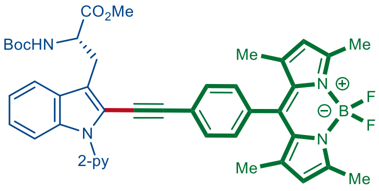

The general procedure **C** was followed using methyl N_a-(*tert*-butoxycarbonyl)-1-(pyridin-2-yl)-*L*-

tryptophanate (**1**) (39.5 mg, 0.10 mmol), 10-[4-(bromoethynyl)phenyl]-5,5-difluoro-1,3,7,9-tetramethyl-5*H*-4λ_4,5λ_4-dipyrrolo[1,2-*c*:2',1'-*f*][1,3,2]diazaborinine (**B1**) (47.0 mg, 0.11 mmol), $MnBr(CO)_5$ (2.7 mg, 10 mol %), KOAc (19.6 mg, 2.0 equiv) and BPh_3 (50 µL, 0.01 M in 1,4-dioxane, 0.5 mol %) in 1,4-dioxane (1.0 mL). Purification by column chromatography on silica gel (CH_2Cl_2/MeOH: 100/0.4) yielded **2** (55.6 mg, 75%) as an orange solid.

M. p.: 130 – 133 ºC.

^{1}H NMR (400 MHz, $CDCl_3$): δ 8.73 (dd, J = 4.8, 0.8 Hz, 1H), 7.96 (ddd, J = 8.1, 7.9, 0.8 Hz, 1H), 7.82 (d, J = 8.1 Hz, 1H), 7.75 (d, J = 8.1 Hz, 1H), 7.65 (d, J = 7.9 Hz, 1H), 7.61 (d, J = 8.1 Hz, 2H), 7.37 (dd, J = 7.9, 4.8 Hz, 1H), 7.34 (dd, J = 7.9, 7.9 Hz, 1H), 7.29 (d, J = 8.1 Hz, 2H), 7.28 – 7.21 (m, 1H), 6.01 (s, 2H), 5.32 (brd, J = 6.1 Hz, 1H), 4.80 (ddd, J = 6.1, 6.0, 6.0 Hz, 1H), 3.66 (s, 3H), 3.54 (d, J = 6.0 Hz, 2H), 2.58 (s, 6H), 1.44 (s, 6H), 1.41 (s, 9H).

^{13}C NMR (126 MHz, $CDCl_3$): δ 172.5 (C_q), 155.7 (C_q), 155.0 (C_q), 150.9 (C_q), 148.9 (CH), 142.8 (C_q), 140.5 (C_q), 137.8 (CH), 136.5 (C_q), 135.1 (C_q), 131.6 (CH), 131.0 (C_q), 128.2 (CH), 127.8 (C_q), 124.9 (CH), 123.4 (C_q), 121.8 (CH), 121.6 (CH), 121.3 (CH), 120.0 (C_q), 119.9 (CH), 119.2 (CH), 119.2 (C_q), 112.0 (CH), 97.8 (C_q), 81.9 (C_q), 79.7 (C_q), 54.0 (CH), 52.3 (CH_3), 28.5 (CH_2), 28.2 (CH_3), 14.6 (CH_3), 14.5 (CH_3).

^{19}F NMR (283 MHz, $CDCl_3$): δ –146.3 (q, $^1J_{B-F}$ = 32.0 Hz).

IR (ATR): 3425, 3052, 2973, 2926, 1743, 1714, 1588, 1543, 1509, 1469 cm^{-1}.

MS (ESI) m/z (relative intensity): 764 (95) $[M+Na]^+$, 742 (100) $[M+H]^+$.

HR-MS (ESI) m/z calcd for $C_{43}H_{42}BF_2N_5O_4Na$ $[M+Na]^+$:764.3197, found: 764.3187.

Methyl (*S*,*E*)-2-[(*tert*-butoxycarbonyl)amino]-3-{2-[4-(5,5-difluoro-1,3,7,9-tetramethyl-5*H*-4λ_4,5λ_4-dipyrrolo[1,2-*c*:2',1'-*f*][1,3,2]diazaborinin-10-yl)styryl]-1-(pyridin-2-yl)-1*H*-indol-3-yl}propanoate (3)

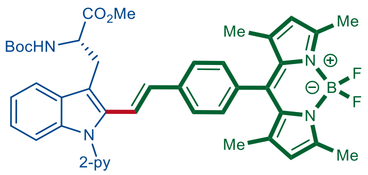

The general procedure **D** was followed using methyl N_a-(*tert*-butoxycarbonyl)-1-(pyridin-2-yl)-*L*-tryptophanate (**1**) (39.5 mg, 0.10 mmol), 10-(4-ethynylphenyl)-5,5-difluoro-1,3,7,9-tetramethyl-5*H*-4λ_4,5λ_4-dipyrrolo[1,2-*c*:2',1'-*f*][1,3,2]diazaborinine (**B2**) (38.3 mg, 0.11 mmol), $MnBr(CO)_5$

(5.5 mg, 20 mol %) and (1-Ad)CO_2H (7.2 mg, 40 mol %) in 1,4-dioxane (1.0 mL). Purification by column chromatography on silica gel (CH_2Cl_2/MeOH: 100/0.2) yielded **3** (65.4 mg, 88%) as an orange solid. The obtained solid was analyzed by inductively coupled plasma mass spectrometry (ICP-MS) analysis, showing 24.5 ppm of residual manganese.

M. p.: 149 – 152 ºC.

^{1}H NMR (300 MHz, $CDCl_3$): δ 8.76 (d, J = 3.7 Hz, 1H), 7.93 (dd, J = 7.2, 7.1 Hz, 1H), 7.62 (d, J = 7.1 Hz, 1H), 7.49 (d, J = 7.7 Hz, 2H), 7.50 – 7.47 (m, 2H), 7.35 (d, J = 16.7 Hz, 1H), 7.28 – 7.21 (m, 5H), 6.54 (d, J = 16.7 Hz, 1H), 6.00 (s, 2H), 5.26 (d, J = 7.6 Hz, 1H), 4.78 (ddd, J = 7.6, 5.4, 5.3 Hz, 1H), 3.57 – 3.54 (m, 5H), 2.58 (s, 6H), 1.44 (s, 9H), 1.43 (s, 6H).

^{13}C NMR (76 MHz, $CDCl_3$): δ 172.7 (C_q), 155.4 (C_q), 155.0 (C_q), 152.0 (C_q), 149.7 (CH), 143.0 (C_q), 141.4 (C_q), 138.4 (CH), 138.2 (C_q), 137.9 (C_q), 134.2 (C_q), 134.1 (C_q), 131.3 (C_q), 130.5 (CH), 129.1 (C_q), 128.3 (CH), 126.9 (CH), 123.9 (CH), 122.4 (CH), 122.3 (CH), 121.2 (CH), 119.0 (CH), 118.0 (CH), 113.5 (C_q), 110.7 (CH), 79.9 (C_q), 54.3 (CH), 52.3 (CH_3), 28.6 (CH_2), 28.3 (CH_3), 14.7 (CH_3), 14.6 (CH_3). (One aromatic CH is missing due to overlap, the overlap was verified by HSQC analysis, showing that the peak at 121.2 corresponds to two carbons).

^{19}F NMR (283 MHz, $CDCl_3$): δ –146.2 (q, $^1J_{B\text{-}F}$ = 31.9 Hz).

IR (ATR): 3425, 3054, 2975, 2928, 2864, 1741, 1710, 1541, 1508, 1455 cm^{-1}.

MS (ESI) m/z (relative intensity): 766 (55) $[M+Na]^+$, 744 (100) $[M+H]^+$.

HR-MS (ESI) m/z calcd for $C_{43}H_{44}BF_2N_5O_4Na$ $[M+Na]^+$:766.3354, found: 766.3343.

Methyl (*S*)-2-acetamido-3-(2-{[4-(5,5-difluoro-1,3,7,9-tetramethyl-5*H*-4λ_4,5λ_4-dipyrrolo[1,2-*c*:2',1'-*f*][1,3,2]diazaborinin-10-yl)phenyl]ethynyl}-1-(pyridin-2-yl)-1*H*-indol-3-yl)propanoate (6)

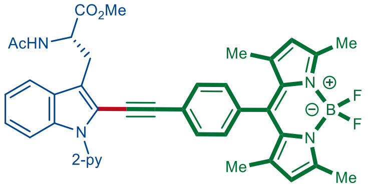

The general procedure **C** was followed using methyl N_α-acetyl-1-(pyridin-2-yl)-*L*-tryptophanate (**4a**) (33.7 mg, 0.10 mmol), 10-[4-(bromoethynyl)phenyl]-5,5-difluoro-1,3,7,9-tetramethyl-5*H*-4λ_4,5λ_4-dipyrrolo[1,2-*c*:2',1'-*f*][1,3,2]diazaborinine (**B1**) (47.0 mg, 0.11 mmol), $MnBr(CO)_5$ (2.7 mg, 10 mol %), KOAc (19.6 mg, 2.0 equiv) and BPh_3 (50 µL, 0.01 M in 1,4-dioxane, 0.5 mol %)

in 1,4-dioxane (1.0 mL). Purification by column chromatography on silica gel (CH_2Cl_2/MeOH: 100/0.4) yielded **6** (35.7 mg, 52%) as an orange solid.

M. p.: 111 – 114 °C.

^{1}H NMR (400 MHz, $CDCl_3$): δ 8.73 (dd, J = 4.9, 1.9 Hz, 1H), 7.99 (ddd, J = 7.8, 7.7, 1.9 Hz, 1H), 7.80 (d, J = 7.8 Hz, 1H), 7.75 (d, J = 8.1 Hz, 1H), 7.62 – 7.58 (m, 3H), 7.40 (dd, J = 7.7, 4.9 Hz, 1H), 7.36 – 7.29 (m, 3H), 7.25 (t, J = 7.5 Hz, 1H), 6.22 (brd, J = 7.6 Hz, 1H), 6.01 (s, 2H), 5.07 (ddd, J = 7.6, 5.4, 5.4 Hz, 1H), 3.68 (s, 3H), 3.63 – 3.55 (m, 2H), 2.58 (s, 6H), 1.92 (s, 3H), 1.43 (s, 6H).

^{13}C NMR (101 MHz, $CDCl_3$): δ 172.0 (C_q), 169.6 (C_q), 155.8 (C_q), 151.0 (C_q), 149.1 (CH), 142.8 (C_q), 140.5 (C_q), 137.9 (CH), 136.7 (C_q), 135.3 (C_q), 131.6 (CH), 131.1 (C_q), 128.4 (CH), 127.8 (C_q), 125.1 (CH), 123.3 (C_q), 122.0 (CH), 121.7 (CH), 121.4 (CH), 120.1 (CH), 119.7 (C_q), 119.6 (C_q), 119.1 (CH), 112.2 (CH), 97.5 (C_q), 82.2 (C_q), 52.6 (CH), 52.5 (CH_3), 27.9 (CH_2), 23.2 (CH_3), 14.6 (CH_3), 14.5 (CH_3).

^{19}F NMR (377 MHz, $CDCl_3$): δ –146.2 (q, $^1J_{B\text{-}F}$ = 32.4 Hz).

IR (ATR): 3300, 3057, 2955, 2924, 2853, 1741, 1661, 1542, 1509, 1469 cm^{-1}.

MS (ESI) m/z (relative intensity): 706 (65) $[M+Na]^+$, 684.3 (100) $[M+H]^+$.

HR-MS (ESI) m/z calcd for $C_{40}H_{36}BF_2N_5O_3Na$ $[M+Na]^+$:706.2778, found: 706.2769.

Benzyl (*S*)-2-[(*tert*-butoxycarbonyl)amino]-3-(2-{[4-(5,5-difluoro-1,3,7,9-tetramethyl-5*H*-4λ_4,5λ_4-dipyrrolo[1,2-*c*:2',1'-*f*][1,3,2]diazaborinin-10-yl)phenyl]ethynyl}-1-(pyridin-2-yl)-1*H*-indol-3-yl)propanoate (7)

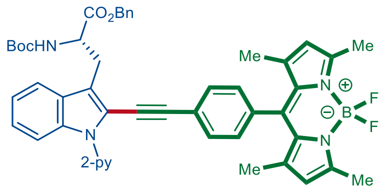

The general procedure **C** was followed using benzyl N_a-(*tert*-butoxycarbonyl)-1-(pyridin-2-yl)-*L*-tryptophanate (**4b**) (47.1 mg, 0.10 mmol), 10-[4-(bromoethynyl)phenyl]-5,5-difluoro-1,3,7,9-tetramethyl-5*H*-4λ_4,5λ_4-dipyrrolo[1,2-*c*:2',1'-*f*][1,3,2]diazaborinine (**B1**) (47.0 mg, 0.11 mmol), $MnBr(CO)_5$ (2.7 mg, 10 mol %), KOAc (19.6 mg, 2.0 equiv) and BPh_3 (50 µL, 0.01 M in 1,4-dioxane, 0.5 mol %) in 1,4-dioxane (1.0 mL). Purification by column chromatography on silica gel (CH_2Cl_2/MeOH: 100/0.4) yielded **7** (56.3 mg, 69%) as an orange solid.

M. p.: 119 – 122 ºC.

^{1}H NMR (300 MHz, $CDCl_3$): δ 8.73 (d, J = 4.8 Hz, 1H), 7.95 (t, J = 7.6 Hz, 1H), 7.82 (d, J = 7.6 Hz, 1H), 7.68 (d, J = 7.1 Hz, 1H), 7.66 (d, J = 6.8 Hz, 1H), 7.56 (d, J = 7.5 Hz, 2H), 7.43 – 7.33 (m, 2H), 7.32 – 7.23 (m, 6H), 7.23 – 7.12 (m, 2H), 6.01 (s, 2H), 5.31 (brd, J = 7.7 Hz, 1H), 5.14 (d, J = 12.4 Hz, 1H), 5.04 (d, J = 12.4 Hz, 1H), 4.84 (ddd, J = 7.7, 6.2, 5.7 Hz, 1H), 3.55 – 3.53 (m, 2H), 2.58 (s, 6H), 1.43 (s, 6H), 1.39 (s, 9H).

^{13}C NMR (101 MHz, $CDCl_3$): δ 172.1 (C_q), 155.8 (C_q), 155.0 (C_q), 151.0 (C_q), 149.0 (CH), 142.9 (C_q), 140.6 (C_q), 137.8 (CH), 136.7 (C_q), 135.2 (C_q), 135.1 (C_q), 131.7 (CH), 131.1 (C_q), 128.5 (CH), 128.3 (CH), 128.2 (CH), 128.1 (CH), 127.9 (C_q), 125.0 (CH), 123.4 (C_q), 121.9 (CH), 121.8 (CH), 121.4 (CH), 120.2 (C_q), 120.0 (CH), 119.3 (C_q), 119.2 (CH), 112.2 (CH), 98.1 (C_q), 82.0 (C_q), 79.8 (C_q), 67.3 (CH_2), 54.3 (CH), 28.7 (CH_2), 28.3 (CH_3), 14.7 (CH_3), 14.6 (CH_3).

^{19}F NMR (377 MHz, $CDCl_3$): δ –146.2 (q, $^1J_{B-F}$ = 32.3 Hz).

IR (ATR): 3423, 3061, 2975, 2926, 2825, 1714, 1543, 1509, 1469, 1437 cm^{-1}.

MS (ESI) m/z (relative intensity): 840 (80) $[M+Na]^+$, 818 (100) $[M+H]^+$.

HR-MS (ESI) m/z calcd for $C_{49}H_{46}BF_2N_5O_4Na$ $[M+Na]^+$:840.3511, found: 840.3505.

Methyl (*S*)-2-[(*tert*-butoxycarbonyl)amino]-3-(2-{[4-(5,5-difluoro-1,3,7,9-tetramethyl-5*H*-4λ_4,5λ_4-dipyrrolo[1,2-*c*:2',1'-*f*][1,3,2]diazaborinin-10-yl)phenyl]ethynyl}-1-(pyrimidin-2-yl)-1*H*-indol-3-yl)propanoate (8)

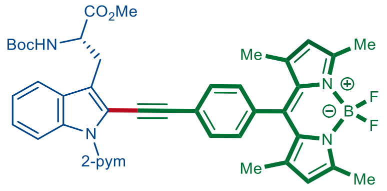

The general procedure **C** was followed using methyl N_a-(*tert*-butoxycarbonyl)-1-(pyrimidin-2-yl)-*L*-tryptophanate (**4c**) (39.6 mg, 0.10 mmol), 10-[4-(bromoethynyl)phenyl]-5,5-difluoro-1,3,7,9-tetramethyl-5*H*-4λ_4,5λ_4-dipyrrolo[1,2-*c*:2',1'-*f*][1,3,2]diazaborinine (**B1**) (47.0 mg, 0.11 mmol), $MnBr(CO)_5$ (2.7 mg, 10 mol %), KOAc (19.6 mg, 2.0 equiv) and BPh_3 (50 µL, 0.01 M in 1,4-dioxane, 0.5 mol %) in 1,4-dioxane (1.0 mL). Purification by column chromatography on silica gel (*n*-hexane/EtOAc: 3/1) yielded **8** (50.7 mg, 68%) as an orange solid.

M. p.: 146 – 149 ºC.

^{1}H NMR (400 MHz, $CDCl_3$): δ 8.90 (d, J = 5.0 Hz, 2H), 8.42 (d, J = 8.4 Hz, 1H), 7.73 (d, J = 7.8 Hz, 2H), 7.66 (d, J = 7.9 Hz, 1H), 7.41 (t, J = 7.9 Hz, 1H), 7.32 (d, J = 7.8 Hz, 2H), 7.30 – 7.29 (m, 1H), 7.26 (t, J = 5.0 Hz, 1H), 6.02 (s, 2H), 5.35 (brd, J = 7.2 Hz, 1H), 4.79 (ddd, J = 7.2, 6.9, 6.9 Hz, 1H), 3.65 (s, 3H), 3.53 (d, J = 6.9 Hz, 2H), 2.59 (s, 6H), 1.46 (s, 6H), 1.40 (s, 9H).
^{13}C NMR (100 MHz, $CDCl_3$): δ 172.6 (C_q), 158.1 (CH), 157.3 (C_q), 155.8 (C_q), 155.0 (C_q), 142.9 (C_q), 140.7 (C_q), 136.2 (C_q), 135.0 (C_q), 131.9 (CH), 131.2 (C_q), 128.8 (C_q), 128.2 (CH), 125.5 (CH), 124.1 (C_q), 122.8 (C_q), 122.6 (CH), 121.3 (CH), 119.4 (C_q), 119.2 (CH), 117.6 (CH), 114.5 (CH), 97.6 (C_q), 82.9 (C_q), 79.8 (C_q), 53.9 (CH), 52.4 (CH_3), 28.6 (CH_2), 28.3 (CH_3), 14.7 (CH_3), 14.6 (CH_3).
^{19}F NMR (377 MHz, $CDCl_3$): δ –146.2 (q, $^{1}J_{B\text{-}F}$ = 32.2 Hz).
IR (ATR): 3428, 3049, 2975, 2927, 2865, 1742, 1711, 1542, 1508, 1452 cm^{-1}.
MS (ESI) m/z (relative intensity): 765 (100) $[M+Na]^+$, 743 (35) $[M+H]^+$.
HR-MS (ESI) m/z calcd for $C_{42}H_{41}BF_2N_6O_4Na$ $[M+Na]^+$:765.3150, found: 765.3139.

Methyl (*S*)-2-[(*tert*-butoxycarbonyl)amino]-3-(2-{[4-(5,5-difluoro-3,7-bis(4-methoxyphenyl)-5*H*-4λ_4,5λ_4-dipyrrolo[1,2-*c*:2',1'-*f*][1,3,2]diazaborinin-10-yl)phenyl]ethynyl}-1-(pyridin-2-yl)-1*H*-indol-3-yl)propanoate (9)

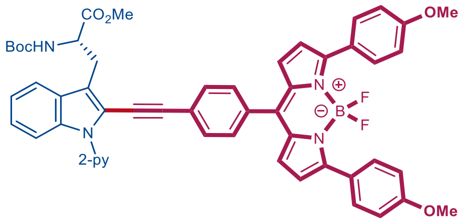

The general procedure **C** was followed using methyl N_a-(*tert*-butoxycarbonyl)-1-(pyridin-2-yl)-*L*-tryptophanate (**1**) (39.5 mg, 0.10 mmol), 10-[4-(bromoethynyl)phenyl]-5,5-difluoro-3,7-bis(4-methoxyphenyl)-5*H*-4λ_4,5λ_4-dipyrrolo[1,2-*c*:2',1'-*f*][1,3,2]diazaborinine (**B3**) (64.1 mg, 0.11 mmol), $MnBr(CO)_5$ (2.7 mg, 10 mol %), KOAc (19.6 mg, 2.0 equiv) and BPh_3 (50 µL, 0.01 M in 1,4-dioxane, 0.5 mol %) in 1,4-dioxane (1.0 mL). Purification by column chromatography on silica gel (*n*-hexane/EtOAc: 3/1) yielded **9** (48.4 mg, 54%) as a purple solid.
M. p.: 112 – 117 ºC.
^{1}H NMR (400 MHz, $CDCl_3$): δ 8.74 (dd, J = 4.0, 1.7 Hz, 1H), 7.98 (ddd, J = 8.1, 8.1, 1.7 Hz, 1H), 7.91 (d, J = 9.0 Hz, 4H), 7.84 (d, J = 8.1 Hz, 1H), 7.76 (d, J = 8.5 Hz, 1H), 7.68 (d, J = 7.9 Hz, 1H), 7.63 (d, J = 8.2 Hz, 2H), 7.58 (d, J = 8.2 Hz, 2H), 7.39 (dd, J = 8.1, 4.0, 1H), 7.36 – 7.34 (m,

1H), 7.27 (t, J = 7.9 Hz, 1H), 6.99 (d, J = 9.0 Hz, 4H), 6.85 (d, J = 4.2 Hz, 2H), 6.65 (d, J = 4.2 Hz, 2H), 5.35 (d, J = 8.1 Hz, 1H), 4.81 (dt, J = 8.1, 6.2 Hz, 1H), 3.88 (s, 6H), 3.70 (s, 3H), 3.60 – 3.51 (m, 2H), 1.42 (s, 9H).

^{13}C NMR (100 MHz, $CDCl_3$): δ 172.6 (C_q), 160.8 (C_q), 158.5 (C_q), 155.1 (C_q), 151.1 (C_q), 149.0 (CH), 141.1 (C_q), 137.9 (CH), 136.7 (C_q), 136.0 (C_q), 134.7 (C_q), 131.1 (CH), 130.9 (CH), 130.7 (CH), 130.0 (CH), 127.9 (C_q), 125.1 (CH), 125.0 (C_q), 124.4 (C_q), 121.9 (CH), 121.7 (CH), 120.7 (CH), 120.3 (C_q), 120.0 (CH), 119.3 (CH), 119.2 (C_q), 113.8 (CH), 112.2 (CH), 97.9 (C_q), 82.8 (C_q), 79.8 (C_q), 55.3 (CH_3), 54.1 (CH), 52.5 (CH_3), 28.7 (CH_2), 28.3 (CH_3).

^{19}F NMR (377 MHz, $CDCl_3$): δ –132.9 (q, $^{1}J_{B\text{-}F}$ = 32.3 Hz).

IR (ATR): 3426, 3053, 2955, 2926, 2853, 1742, 1713, 1605, 1560, 1536 cm^{-1}.

MS (ESI) m/z (relative intensity): 920 (70) $[M+Na]^+$, 898 (100) $[M+H]^+$.

HR-MS (ESI) m/z calcd for $C_{53}H_{46}BF_2N_5O_6Na$ $[M+Na]^+$:920.3410, found: 920.3395**.**

Methyl (*S*)-[2-({*tert*-butoxycarbonyl}amino)-3-(2-{[4-(5,5-difluoro-1,3,7,9-tetramethyl-5*H*-4λ_4,5λ_4-dipyrrolo[1,2-*c*:2',1'-*f*][1,3,2]diazaborinin-10-yl)phenyl]ethynyl}-1-{pyridin-2-yl}-1*H*-indol-3-yl)propanoyl]glycinate (10)

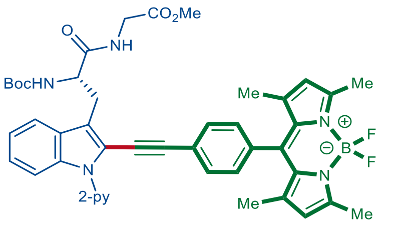

The general procedure **C** was followed using methyl N_{α}-(*tert*-butoxycarbonyl)-1-(pyridin-2-yl)-*L*-tryptophylglycinate (**4d**) (45.3 mg, 0.10 mmol), 10-[4-(bromoethynyl)phenyl]-5,5-difluoro-1,3,7,9-tetramethyl-5*H*-4λ^4,5λ^4-dipyrrolo[1,2-*c*:2',1'-*f*][1,3,2]diazaborinine (**B1**) (47.0 mg, 0.11 mmol), $MnBr(CO)_5$ (2.7 mg, 10 mol %), KOAc (19.6 mg, 2.0 equiv) and BPh_3 (50 µL, 0.01 M in 1,4-dioxane, 0.5 mol %) in 1,4-dioxane (1.0 mL). Purification by column chromatography on silica gel (CH_2Cl_2/MeOH: 100/0.3) yielded **10** (43.2 mg, 54%) as an orange solid.

M. p.: 146 – 149 ºC.

^{1}H NMR (400 MHz, $CDCl_3$): δ 8.69 (dd, J = 4.9, 1.9 Hz, 1H), 7.94 (ddd, J = 8.1, 8.1, 1.9 Hz, 1H), 7.77 (dd, J = 8.1, 1.0 Hz, 1H), 7.73 (d, J = 8.1 Hz, 1H), 7.69 (d, J = 7.9 Hz, 1H), 7.60 (d, J = 8.2 Hz, 2H), 7.35 (ddd, J = 7.5, 4.9, 1.0 Hz, 1H), 7.30 (ddd, J = 8.1, 4.9, 1.0 Hz, 1H), 7.26 (d, J = 8.2 Hz, 2H), 7.21 (ddd, J = 8.1, 7.1, 1.0 Hz, 1H), 6.30 (brs, 1H), 5.97 (s, 2H), 5.38 (brs, 1H), 4.61 –

4.56 (m, 1H), 3.93 (dd, J = 18.2, 5.2 Hz, 1H), 3.82 (d, J = 18.2 Hz, 1H), 3.60 (s, 3H), 3.52 – 3.46 (m, 2H), 2.54 (s, 6H), 1.40 (s, 6H), 1.36 (s, 9H).

^{13}C NMR (100 MHz, $CDCl_3$): δ 171.5 (C_q), 171.5 (C_q), 169.5 (C_q), 155.8 (C_q), 151.0 (C_q), 149.0 (CH), 142.9 (C_q), 140.6 (C_q), 137.8 (CH), 136.6 (C_q), 135.2 (C_q), 131.7 (CH), 131.1 (C_q), 128.3 (CH), 127.7 (C_q), 125.1 (CH), 123.3 (C_q), 121.9 (CH), 121.8 (CH), 121.3 (CH), 120.4 (C_q), 120.0 (CH), 119.4 (CH), 119.3 (C_q), 112.1 (CH), 98.3 (C_q), 81.7 (C_q), 80.1 (C_q), 55.2 (CH), 52.2 (CH_3), 41.3 (CH_2), 28.6 (CH_2), 28.2 (CH_3), 14.6 (CH_3), 14.5 (CH_3).

^{19}F NMR (283 MHz, $CDCl_3$): δ –146.2 (q, $^1J_{B\text{-}F}$ = 32.4 Hz).

IR (ATR): 3408, 3328, 3052, 2974, 2928, 1748, 1712, 1677, 1588, 1543 cm^{-1}.

MS (ESI) m/z (relative intensity): 821 (100) $[M+Na]^+$, 799 (45) $[M+H]^+$.

HR-MS (ESI) m/z calcd for $C_{45}H_{45}BF_2N_6O_5Na$ $[M+Na]^+$:821.3412, found: 821.3412.

Methyl {(*S*)-2-[(*tert*-butoxycarbonyl)amino]-3-(2-{[4-(5,5-difluoro-1,3,7,9-tetramethyl-5*H*-4λ_4,5λ_4-dipyrrolo[1,2-*c*:2',1'-*f*][1,3,2]diazaborinin-10-yl)phenyl]ethynyl}-1-(pyridin-2-yl)-1*H*-indol-3-yl)propanoyl}-*L*-alaninate (11)

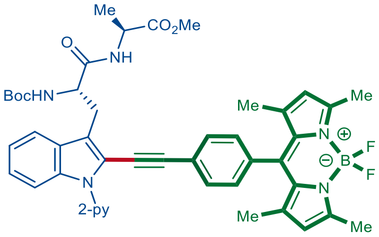

The general procedure **C** was followed using methyl N_a-(*tert*-butoxycarbonyl)-1-(pyridin-2-yl)-*L*-tryptophyl-*L*-alaninate (**4e**) (46.6 mg, 0.10 mmol), 10-[4-(bromoethynyl)phenyl]-5,5-difluoro-1,3,7,9-tetramethyl-5*H*-4λ_4,5λ_4-dipyrrolo[1,2-*c*:2',1'-*f*][1,3,2]diazaborinine (**B1**) (47.0 mg, 0.11 mmol), $MnBr(CO)_5$ (5.4 mg, 20 mol %), KOAc (19.6 mg, 2.0 equiv) and BPh_3 (50 µL, 0.01 M in 1,4-dioxane, 0.5 mol %) in 1,4-dioxane (1.0 mL). Purification by column chromatography on silica gel (CH_2Cl_2/MeOH: 100/0.6) yielded **11** (43.9 mg, 54%) as an orange solid.

M. p.: 215 – 219 ºC.

^{1}H NMR (400 MHz, $CDCl_3$): δ 8.74 (dd, J = 4.7, 1.8 Hz, 1H), 7.98 (ddd, J = 7.9, 7.9, 1.8 Hz, 1H), 7.81 (d, J = 7.9 Hz, 1H), 7.78 (d, J = 7.3 Hz, 1H), 7.76 (d, J = 7.9 Hz, 1H), 7.64 (d, J = 8.0 Hz, 2H), 7.38 (dd, J = 7.9, 4.7 Hz, 1H), 7.34 (t, J = 7.3 Hz, 1H), 7.30 (d, J = 8.0 Hz, 2H), 7.25 (dd, J = 7.9, 7.9 Hz, 1H), 6.41 (brd, J = 7.1 Hz, 1H), 6.02 (s, 2H), 5.48 (brs, 1H), 4.65 – 4.56 (m, 1H),

4.46 (dt, *J* = 7.1, 7.1 Hz, 1H), 3.65 – 3.60 (m, 1H), 3.55 (s, 3H), 3.44 (dd, *J* = 13.0, 7.1 Hz, 1H), 2.58 (s, 6H), 1.45 (s, 6H), 1.42 (s, 9H), 1.21 (d, *J* = 7.1 Hz, 3H).

^{13}C NMR (100 MHz, $CDCl_3$): δ 172.6 (C_q), 170.7 (C_q), 155.8 (C_q), 155.2 (C_q), 151.1 (C_q), 149.0 (CH), 142.9 (C_q), 140.6 (C_q), 137.8 (CH), 136.7 (C_q), 135.2 (C_q), 131.8 (CH), 131.1 (C_q), 128.3 (CH), 127.7 (C_q), 125.1 (CH), 123.4 (C_q), 121.9 (CH), 121.8 (CH), 121.4 (CH), 120.5 (C_q), 120.0 (CH), 119.6 (CH), 119.3 (C_q), 112.1 (CH), 98.2 (C_q), 81.9 (C_q), 80.0 (C_q), 55.2 (CH), 52.2 (CH), 48.1 (CH_3), 28.7 (CH_2), 28.2 (CH_3), 18.5 (CH_3), 14.7 (CH_3), 14.6 (CH_3).

^{19}F NMR (377 MHz, $CDCl_3$): δ –146.2 (q, $^{1}J_{B\text{-}F}$ = 32.3 Hz).

IR (ATR): 3410, 3326, 3298, 2974, 2927, 1743, 1713, 1674, 1543, 1510 cm^{-1}.

MS (ESI) m/z (relative intensity): 835 (100) $[M+Na]^+$, 813 (30) $[M+H]^+$.

HR-MS (ESI) m/z calcd for $C_{46}H_{47}BF_2N_6O_5Na$ $[M+Na]^+$:835.3569, found: 835.3564.

Methyl (*S*)-2-{(2*S*,3*S*)-2-[(*tert*-butoxycarbonyl)amino]-3-methylpentanamido}-3-(2-{[4-(5,5-difluoro-1,3,7,9-tetramethyl-5*H*-4λ_4,5λ_4-dipyrrolo[1,2-*c*:2',1'-*f*][1,3,2]diazaborinin-10-yl)phenyl]ethynyl}-1-(pyridin-2-yl)-1*H*-indol-3-yl)propanoate (12)

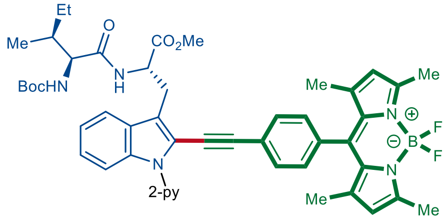

The general procedure **C** was followed using methyl *N*$_\alpha$-[(*tert*-butoxycarbonyl)-*L*-isoleucyl]-1-(pyridin-2-yl)-*L*-tryptophanate (**4f**) (50.9 mg, 0.10 mmol), 10-[4-(bromoethynyl)phenyl]-5,5-difluoro-1,3,7,9-tetramethyl-5*H*-4λ_4,5λ_4-dipyrrolo[1,2-*c*:2',1'-*f*][1,3,2]diazaborinine (**B1**) (47.0 mg, 0.11 mmol), $MnBr(CO)_5$ (5.4 mg, 20 mol %), KOAc (19.6 mg, 2.0 equiv) and BPh_3 (50 μL, 0.01 M in 1,4-dioxane, 0.5 mol %) in 1,4-dioxane (1.0 mL). Purification by column chromatography on silica gel (CH_2Cl_2/MeOH: 100/0.5) yielded **12** (44.4 mg, 52%) as an orange solid.

M. p.: 131 – 134 ºC.

^{1}H NMR (300 MHz, $CDCl_3$): δ 8.73 (d, *J* = 4.9 Hz, 1H), 7.98 (dd, *J* = 7.6, 7.6 Hz, 1H), 7.83 (d, *J* = 7.6 Hz, 1H), 7.78 (d, *J* = 8.1 Hz, 1H), 7.69 (d, *J* = 7.9 Hz, 1H), 7.62 (d, *J* = 7.6 Hz, 2H), 7.44 – 7.34 (m, 2H), 7.32 (d, *J* = 7.6 Hz, 2H), 7.28 – 7.27 (m, 1H), 6.55 (brd, *J* = 5.9 Hz, 1H), 6.01 (s, 2H), 5.10 – 4.89 (m, 2H), 4.08 – 3.93 (m, 1H), 3.67 (s, 3H), 3.55 (d, *J* = 6.6 Hz, 2H), 2.58 (s, 6H),

1.94 – 1.77 (m, 1H), 1.71 – 1.55 (m, 1H), 1.44 (s, 6H), 1.42 (s, 9H), 1.22 – 0.95 (m, 1H), 0.91 – 0.77 (m, 6H).

^{13}C NMR (125 MHz, $CDCl_3$): δ 172.0 (C_q), 171.1 (C_q), 155.8 (C_q), 155.5 (C_q), 151.0 (C_q), 149.0 (CH), 142.8 (C_q), 140.5 (C_q), 137.9 (CH), 136.7 (C_q), 135.3 (C_q), 131.7 (CH), 131.1 (C_q), 128.4 (CH), 127.6 (C_q), 125.2 (CH), 123.3 (C_q), 122.0 (CH), 121.9 (CH), 121.3 (CH), 120.0 (CH), 119.8 (C_q), 119.2 (C_q), 119.1 (CH), 112.2 (CH), 98.0 (C_q), 81.8 (C_q), 79.7 (C_q), 59.1 (CH), 52.9 (CH), 52.5 (CH_3), 37.6 (CH), 28.3 (CH_2), 28.2 (CH_3), 24.5 (CH_2), 15.2 (CH_3), 14.7 (CH_3), 14.6 (CH_3), 11.5 (CH_3).

^{19}F NMR (282 MHz, $CDCl_3$): δ –146.3 (q, $^{1}J_{B\text{-}F}$ = 32.3 Hz).

IR (ATR): 3413, 3320, 3049, 2963, 2928, 1745, 1716, 1677, 1589, 1544 cm^{-1}.

MS (ESI) m/z (relative intensity): 878 (100) $[M+Na]^+$, 855 (70) $[M+H]^+$.

HR-MS (ESI) m/z calcd for $C_{49}H_{53}BF_2N_6O_5Na$ $[M+Na]^+$:877.4039, found: 877.4037.

Methyl (*S*)-2-[(*S*)-2-acetamido-5-amino-5-oxopentanamido]-3-(2-{[4-(5,5-difluoro-1,3,7,9-tetramethyl-5*H*-4λ_4,5λ_4-dipyrrolo[1,2-*c*:2',1'-*f*][1,3,2]diazaborinin-10-yl)phenyl]ethynyl}-1-(pyridin-2-yl)-1*H*-indol-3-yl)propanoate (13)

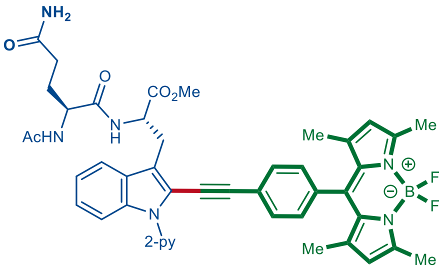

The general procedure **C** was followed using methyl *N*$_a$-(acetyl-*L*-glutaminyl)-1-(pyridin-2-yl)-*L*-tryptophanate (**4g**) (46.5 mg, 0.10 mmol), 10-[4-(bromoethynyl)phenyl]-5,5-difluoro-1,3,7,9-tetramethyl-5*H*-4λ_4,5λ_4-dipyrrolo[1,2-*c*:2',1'-*f*][1,3,2]diazaborinine (**B1**) (47.0 mg, 0.11 mmol), $MnBr(CO)_5$ (5.4 mg, 20 mol %), KOAc (19.6 mg, 2.0 equiv) and BPh_3 (50 µL, 0.01 m in 1,4-dioxane, 0.5 mol %) in 1,4-dioxane (1.0 mL). Purification by column chromatography on silica gel (CH_2Cl_2/MeOH: 100/2) yielded **13** (41.3 mg, 51%) as a brown oil.

^{1}H NMR (400 MHz, DMSO-*d*$_6$, 120 °C): δ 8.70 (dd, *J* = 4.0, 2.0 Hz, 1H), 8.10 (ddd, *J* = 8.1, 7.5, 2.0 Hz, 1H), 7.92 (dd, *J* = 6.9, 6.9 Hz, 1H), 7.80 (dd, *J* = 8.1, 2.0 Hz, 1H), 7.72 (d, *J* = 7.9 Hz, 1H), 7.67 (d, *J* = 8.3 Hz, 1H), 7.65 – 7.61 (m, 2H), 7.55 – 7.46 (m, 2H), 7.45 – 7.41 (m, 2H), 7.30 (dd, *J* = 8.3, 7.0 Hz, 1H), 7.23 (dd, *J* = 8.1, 7.0 Hz, 1H), 6.49 (brs, 2H), 6.16 (s, 2H), 4.94 – 4.74 (m,

1H), 4.39 – 4.24 (m, 1H), 3.61 (s, 3H), 3.51 (dd, J = 14.3, 7.1 Hz, 1H), 3.41 (dd, J = 14.3, 7.0 Hz, 1H), 2.49 (s, 6H), 2.13 (t, J = 7.6 Hz, 1H), 2.08 (t, J = 7.9 Hz, 1H), 1.99 – 1.86 (m, 1H), 1.83 (s, 3H), 1.80 – 1.70 (m, 1H), 1.44 (s, 6H).

^{13}C NMR (101 MHz, DMSO-d_6)(2:1 mixture of rotamers): δ_{major} 174.0 (C_q), 172.2 (C_q), 172.1 (C_q), 169.5 (C_q), 155.6 (C_q), 150.6 (C_q), 149.4 (CH), 143.1 (C_q), 141.3 (C_q), 139.3 (CH), 136.5 (C_q), 134.9 (C_q), 132.0 (CH), 130.9 (C_q), 129.0 (CH), 127.6 (C_q), 125.2 (CH), 123.1 (C_q), 123.0 (CH), 121.9 (CH), 121.0 (C_q), 120.6 (CH), 120.5 (CH), 119.8 (CH), 119.1 (C_q), 112.6 (CH), 98.2 (C_q), 81.9 (C_q), 53.1 (CH), 52.5 (CH_3), 52.4 (CH), 31.8 (CH_2), 28.5 (CH_2), 27.9 (CH_2), 22.9 (CH_3), 14.7 (CH_3), 14.6 (CH_3).

^{19}F NMR (377 MHz, $CDCl_3$): δ –143.6 (q, $^1J_{B-F}$ = 29.7 Hz).

IR (ATR): 3415, 3051, 2982, 2927, 1656, 1545, 1510, 1470, 1266, 1196 cm^{-1}.

MS (ESI) m/z (relative intensity): 834 (100) $[M+Na]^+$, 812 (40) $[M+H]^+$.

HR-MS (ESI) m/z calcd for $C_{45}H_{44}BF_2N_7O_5Na$ $[M+Na]^+$:834.3365; found: 834.3357.

Dimethyl {(*S*)-2-[(*tert*-butoxycarbonyl)amino]-3-(2-{[4-(5,5-difluoro-1,3,7,9-tetramethyl-5*H*-4λ_4,5λ_4-dipyrrolo[1,2-*c*:2',1'-*f*][1,3,2]diazaborinin-10-yl)phenyl]ethynyl}-1-(pyridin-2-yl)-1*H*-indol-3-yl)propanoyl}-*L*-aspartate (14)

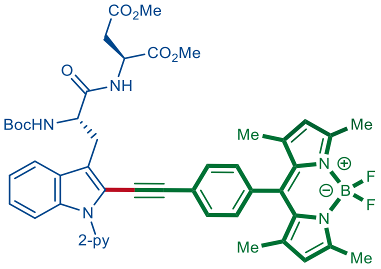

The general procedure **C** was followed using dimethyl N_a-(*tert*-butoxycarbonyl)-1-(pyridin-2-yl)-*L*-tryptophyl-*L*-aspartate (**4h**) (52.4 mg, 0.10 mmol), 10-[4-(bromoethynyl)phenyl]-5,5-difluoro-1,3,7,9-tetramethyl-5*H*-4λ_4,5λ_4-dipyrrolo[1,2-*c*:2',1'-*f*][1,3,2]diazaborinine (**B1**) (47.0 mg, 0.11 mmol), $MnBr(CO)_5$ (2.7 mg, 10 mol %), KOAc (19.6 mg, 2.0 equiv) and BPh_3 (50 µL, 0.01 M in 1,4-dioxane, 0.5 mol %) in 1,4-dioxane (1.0 mL). Purification by column chromatography on silica gel (CH_2Cl_2/MeOH: 100/0.4) yielded **14** (46.2 mg, 53%) as an orange solid.

M. p.: 150 – 155 ºC.

^{1}H NMR (300 MHz, $CDCl_3$): δ 8.72 (d, J = 4.8 Hz, 1H), 7.97 (dd, J = 7.2, 7.2 Hz, 1H), 7.81 (d, J = 7.2 Hz, 1H), 7.79 (d, J = 7.9 Hz, 1H), 7.72 (d, J = 7.8 Hz, 1H), 7.65 (d, J = 7.9 Hz, 2H), 7.38

(dd, J = 7.2, 4.8 Hz, 1H), 7.37 – 7.25 (m, 2H), 7.25 (t, J = 7.8 Hz, 1H), 6.81 (brd, J = 6.6 Hz, 1H), 6.01 (s, 2H), 5.48 (brs, 1H), 4.72 – 4.61 (m, 1H), 4.61 (q, J = 6.6 Hz, 1H), 3.70 – 3.60 (m, 1H), 3.60 (s, 3H), 3.56 (s, 3H), 3.46 (dd, J = 13.9, 6.6 Hz, 1H), 2.84 (dd, J = 17.3, 4.1 Hz, 1H), 2.64 – 2.60 (m, 1H), 2.58 (s, 6H), 1.45 (s, 6H), 1.41 (s, 9H).

^{13}C NMR (101 MHz, $CDCl_3$): δ 171.2 (C_q), 171.0 (C_q), 170.4 (C_q), 155.8 (C_q), 155.1 (C_q), 151.0 (C_q), 149.0 (CH), 142.9 (C_q), 140.6 (C_q), 137.8 (CH), 136.7 (C_q), 135.3 (C_q), 131.8 (CH), 131.1 (C_q), 128.3 (CH), 127.7 (C_q), 125.1 (CH), 123.3 (C_q), 121.9 (CH), 121.8 (CH), 121.3 (CH), 120.3 (C_q), 120.1 (CH), 119.4 (CH), 119.2 (C_q), 112.1 (CH), 98.3 (C_q), 81.9 (C_q), 80.1 (C_q), 55.5 (CH), 52.7 (CH_3), 51.8 (CH_3), 48.6 (CH), 35.8 (CH_2), 28.4 (CH_2), 28.2 (CH_3), 14.7 (CH_3), 14.6 (CH_3).

^{19}F NMR (377 MHz, $CDCl_3$): δ –146.2 (q, $^{1}J_{B\text{-}F}$ = 32.4 Hz).

IR (ATR): 3406, 3057, 2974, 2953, 2927, 1736, 1675, 1542, 1508, 1451 cm^{-1}.

MS (ESI) m/z (relative intensity): 893 (100) $[M+Na]^+$, 871 (40) $[M+H]^+$.

HR-MS (ESI) m/z calcd for $C_{48}H_{49}BF_2N_6O_7Na$ $[M+Na]^+$:893.3624, found: 893.3629.

Methyl {(*S*)-2-[(*tert*-butoxycarbonyl)amino]-3-(2-{[4-(5,5-difluoro-1,3,7,9-tetramethyl-5*H*-4λ_4,5λ_4-dipyrrolo[1,2-*c*:2',1'-*f*][1,3,2]diazaborinin-10-yl)phenyl]ethynyl}-1-(pyridin-2-yl)-1*H*-indol-3-yl)propanoyl}-*L*-phenylalaninate (15)

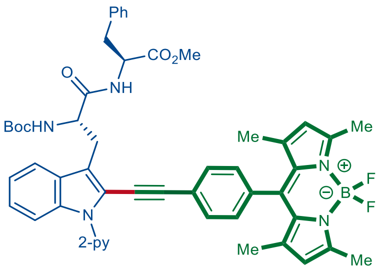

The general procedure **C** was followed using methyl N_a-(*tert*-butoxycarbonyl)-1-(pyridin-2-yl)-*L*-tryptophyl-*L*-phenylalaninate (**4i**) (54.3 mg, 0.10 mmol), 10-[4-(bromoethynyl)phenyl]-5,5-difluoro-1,3,7,9-tetramethyl-5*H*-4λ_4,5λ_4-dipyrrolo[1,2-*c*:2',1'-*f*][1,3,2]diazaborinine (**B1**) (47.0 mg, 0.11 mmol), $MnBr(CO)_5$ (2.7 mg, 10 mol %), KOAc (19.6 mg, 2.0 equiv) and BPh_3 (50 µL, 0.01 M in 1,4-dioxane, 0.5 mol %) in 1,4-dioxane (1.0 mL). Purification by column chromatography on silica gel (CH_2Cl_2/MeOH: 100/0.3) yielded **15** (56.9 mg, 64%) as an orange solid.

M. p.: 124 – 128 ºC.

^{1}H NMR (300 MHz, $CDCl_3$): δ 8.73 (dd, J = 5.0, 1.7 Hz, 1H), 7.97 (ddd, J = 8.0, 7.8, 1.7 Hz, 1H), 7.81 (d, J = 8.0 Hz, 1H), 7.76 (d, J = 8.0 Hz, 2H), 7.62 (d, J = 8.0 Hz, 2H), 7.38 (dd, J = 7.8, 5.0 Hz, 1H), 7.35 – 7.31 (m, 2H), 7.28 – 7.26 (m, 2H), 7.23 – 7.17 (m, 3H), 6.87 – 6.85 (m, 2H), 6.29 (brd, J = 5.6 Hz, 1H), 6.02 (s, 2H), 5.45 (brs, 1H), 4.72 (q, J = 5.0 Hz, 1H), 4.56 (dd, J = 5.6, 4.9 Hz, 1H), 3.64 – 3.57 (m, 1H), 3.49 (s, 3H), 3.43 – 3.39 (m, 1H), 2.99 (dd, J = 12.3, 4.9 Hz, 1H), 2.87 (dd, J = 12.3, 2.8 Hz, 1H), 2.59 (s, 6H), 1.45 (s, 6H), 1.40 (s, 9H).

^{13}C NMR (125 MHz, $CDCl_3$): δ 170.9 (C_q), 170.8 (C_q), 155.7 (C_q), 155.1 (C_q), 150.8 (C_q), 148.8 (CH), 142.9 (C_q), 140.5 (C_q), 137.9 (CH), 136.6 (C_q), 135.5 (C_q), 135.2 (C_q), 131.7 (CH), 131.1 (C_q), 129.1 (CH), 129.0 (CH), 128.3 (CH), 128.2 (CH), 127.7 (C_q), 126.8 (CH), 125.2 (CH), 123.3 (C_q), 121.9 (CH), 121.3 (CH), 120.4 (C_q), 120.1 (CH), 119.5 (CH), 119.2 (C_q), 112.0 (CH), 98.3 (C_q), 81.7 (C_q), 80.0 (C_q), 55.3 (CH), 53.3 (CH), 52.0 (CH_3), 38.0 (CH_2), 28.6 (CH_2), 28.2 (CH_3), 14.6 (CH_3), 14.5 (CH_3).

^{19}F NMR (377 MHz, $CDCl_3$): δ –146.2 (q, $^{1}J_{B\text{-}F}$ = 32.2 Hz).

IR (ATR): 3412, 3059, 3029, 2973, 2928, 1742, 1714, 1677, 1543, 1510 cm^{-1}.

MS (ESI) m/z (relative intensity): 911 (100) $[M+Na]^+$, 889 (65) $[M+H]^+$.

HR-MS (ESI) m/z calcd for $C_{52}H_{51}BF_2N_6O_5Na$ $[M+Na]^+$:911.3883, found: 911.3869.

Methyl (*S*)-2-{(*S*)-2-[(*tert*-butoxycarbonyl)amino]-3-(1*H*-indol-3-yl)propanamido}-3-(2-{[4-(5,5-difluoro-1,3,7,9-tetramethyl-5*H*-4λ_4,5λ_4-dipyrrolo[1,2-*c*:2',1'-*f*][1,3,2]diazaborinin-10-yl)phenyl]ethynyl}-1-(pyridin-2-yl)-1*H*-indol-3-yl)propanoate (16)

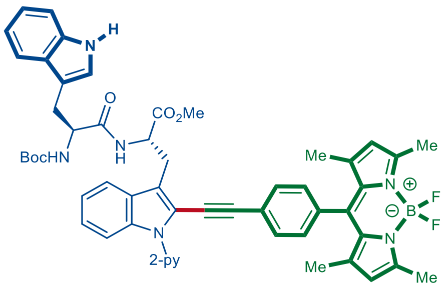

The general procedure **C** was followed using methyl N_a-((*tert*-butoxycarbonyl)-*L*-tryptophyl)-1-(pyridin-2-yl)-*L*-tryptophanate (**4j**) (58.1 mg, 0.10 mmol), 10-[4-(bromoethynyl)phenyl]-5,5-difluoro-1,3,7,9-tetramethyl-5*H*-4λ_4,5λ_4-dipyrrolo[1,2-*c*:2',1'-*f*][1,3,2]diazaborinine (**B1**) (47.0 mg, 0.11 mmol), $MnBr(CO)_5$ (2.7 mg, 10 mol %), KOAc (19.6 mg, 2.0 equiv) and BPh_3 (50 µL, 0.01 M in 1,4-dioxane, 0.5 mol %) in 1,4-dioxane (1.0 mL). Purification by column

chromatography on silica gel (CH_2Cl_2/MeOH: 100/0.4) yielded **16** (47.3 mg, 51%) as an orange solid.

M. p.: 146 – 150 ºC.

^{1}H NMR (300 MHz, $CDCl_3$): δ 8.89 (brs, 1H), 8.83 (d, J = 4.7 Hz, 1H), 8.02 (dd, J = 7.8, 7.8 Hz, 1H), 7.78 (d, J = 8.4 Hz, 1H), 7.68 (d, J = 8.4 Hz, 1H), 7.59 (d, J = 7.8 Hz, 1H), 7.50 – 7.47 (m, 2H), 7.44 (d, J = 7.9 Hz, 2H), 7.29 – 7.28 (m, 1H), 7.26 (d, J = 7.9 Hz, 2H), 7.15 – 7.09 (m, 3H), 7.02 (dd, J = 7.3, 7.3 Hz, 1H), 6.59 (s, 1H), 6.49 (brd, J = 8.2 Hz, 1H), 6.00 (s, 2H), 5.10 (brs, 1H), 5.02 (ddd, J = 6.6, 6.6, 6.6 Hz, 1H), 4.49 – 4.34 (m, 1H), 3.61 (s, 3H), 3.52 – 3.40 (m, 2H), 3.29 (dd, J = 14.6, 6.6 Hz, 1H), 3.02 (dd, J = 14.6, 6.6 Hz, 1H), 2.58 (s, 6H), 1.43 (s, 9H), 1.42 (s, 6H).

^{13}C NMR (100 MHz, $CDCl_3$): δ 171.69 (C_q), 171.61 (C_q), 155.8 (C_q), 155.2 (C_q), 151.0 (C_q), 148.9 (CH), 142.9 (C_q), 140.5 (C_q), 138.4 (CH), 136.6 (C_q), 136.0 (C_q), 135.3 (C_q), 131.6 (CH), 131.1 (C_q), 128.3 (CH), 127.6 (C_q), 127.5 (C_q), 125.2 (CH), 122.9 (C_q), 122.7 (CH), 122.4 (CH), 121.9 (CH), 121.7 (CH), 121.3 (CH), 120.6 (CH), 119.6 (C_q), 119.4 (CH), 119.3 (C_q), 118.8 (CH), 118.6 (CH), 112.1 (CH), 110.9 (CH), 110.1 (C_q), 98.2 (C_q), 81.0 (C_q), 79.7 (C_q), 54.9 (CH), 52.9 (CH), 52.4 (CH_3), 28.2 (CH_3), 28.0 (CH_2), 27.7 (CH_2), 14.6 (CH_3), 14.5 (CH_3).

^{19}F NMR (283 MHz, $CDCl_3$): δ –146.23 (q, $^1J_{B\text{-}F}$ = 32.2 Hz).

IR (ATR): 3410, 3357, 3056, 2973, 2926, 1740, 1712, 1675, 1543, 1509 cm^{-1}.

MS (ESI) m/z (relative intensity): 950 (100) $[M+Na]^+$, 928 (65) $[M+H]^+$.

HR-MS (ESI) m/z calcd for $C_{54}H_{52}BF_2N_7O_5Na$ $[M+Na]^+$:950.3992; found: 950.3981.

Methyl {(*S*)-2-[(*tert*-butoxycarbonyl)amino]-3-(2-{[4-(5,5-difluoro-1,3,7,9-tetramethyl-5*H*-4λ_4,5λ_4-dipyrrolo[1,2-*c*:2',1'-*f*][1,3,2]diazaborinin-10-yl)phenyl]ethynyl}-1-(pyridin-2-yl)-1*H*-indol-3-yl)propanoyl}-*L*-tyrosinate (17)

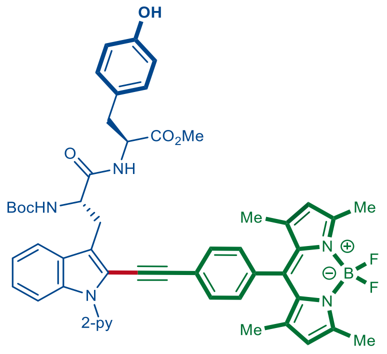

The general procedure **C** was followed using methyl N_a-(*tert*-butoxycarbonyl)-1-(pyridin-2-yl)-*L*-tryptophyl-*L*-tyrosinate (**4k**) (55.8 mg, 0.10 mmol), 10-[4-(bromoethynyl)phenyl]-5,5-difluoro-1,3,7,9-tetramethyl-5*H*-4λ_4,5λ_4-dipyrrolo[1,2-*c*:2',1'-*f*][1,3,2]diazaborinine (**B1**) (47.0 mg, 0.11 mmol), $MnBr(CO)_5$ (2.7 mg, 10 mol %), KOAc (19.6 mg, 2.0 equiv) and BPh_3 (50 µL, 0.01 M in 1,4-dioxane, 0.5 mol %) in 1,4-dioxane (1.0 mL). Purification by column chromatography on silica gel (CH_2Cl_2/MeOH: 100/0.8) yielded **17** (49.5 mg, 55%) as an orange solid.

M. p.: 160 – 162 ºC.

^{1}H NMR (400 MHz, $CDCl_3$): δ 8.74 (dd, *J* = 5.4, 1.8 Hz, 1H), 7.98 (ddd, *J* = 7.8, 7.8, 1.8 Hz, 1H), 7.77 – 7.72 (m, 3H), 7.62 (d, *J* = 7.9 Hz, 2H), 7.40 (dd, *J* = 7.8, 5.4 Hz, 1H), 7.33 – 7.26 (m, 3H), 7.23 (t, *J* = 7.5 Hz, 1H), 6.64 (d, *J* = 8.1 Hz, 2H), 6.58 (d, *J* = 8.1 Hz, 2H), 6.39 (br, 1H), 6.34 (brd, *J* = 6.0 Hz, 1H), 6.01 (s, 2H), 5.42 (brs, 1H), 4.64 (q, *J* = 6.0 Hz, 1H), 4.60 – 4.56 (m, 1H), 3.65 – 3.58 (m, 1H), 3.52 (s, 3H), 3.39 (dd, *J* = 14.2, 7.2 Hz, 1H), 2.92 – 2.76 (m, 2H), 2.58 (s, 6H), 1.43 (s, 6H), 1.40 (s, 9H).

^{13}C NMR (100 MHz, $CDCl_3$): δ 171.2 (C_q), 171.0 (C_q), 155.8 (C_q), 155.2 (C_q), 155.0 (C_q), 150.9 (C_q), 148.9 (CH), 142.9 (C_q), 140.6 (C_q), 138.1 (CH), 136.7 (C_q), 135.3 (C_q), 131.8 (CH), 131.1 (C_q), 130.1 (CH), 128.3 (CH), 127.7 (C_q), 127.0 (C_q), 125.2 (CH), 123.3 (C_q), 122.1 (CH), 121.9 (CH), 121.4 (CH), 120.4 (CH), 120.2 (C_q), 119.6 (CH), 119.3 (C_q), 115.5 (CH), 111.9 (CH), 98.3 (C_q), 81.7 (C_q), 80.2 (C_q), 55.2 (CH), 53.5 (CH), 52.1 (CH_3), 37.1 (CH_2), 28.4 (CH_2), 28.2 (CH_3), 14.7 (CH_3), 14.6 (CH_3).

^{19}F NMR (377 MHz, $CDCl_3$): δ – 146.1 (q, $^1J_{B\text{-}F}$ = 31.7 Hz).

IR (ATR): 3406, 3058, 2975, 2927, 1740, 1713, 1674, 1589, 1542, 1509 cm^{-1}.

MS (ESI) m/z (relative intensity): 927 (50) $[M+Na]^+$, 905 (100) $[M+H]^+$.

HR-MS (ESI) m/z calcd for $C_{52}H_{51}BF_2N_6O_6Na$ $[M+Na]^+$:905.4013; found: 905.4021.

Methyl {(*S*)-2-[(*tert*-butoxycarbonyl)amino]-3-(2-{[4-(5,5-difluoro-1,3,7,9-tetramethyl-5*H*-4*λ*4,5*λ*4-dipyrrolo[1,2-*c*:2',1'-*f*][1,3,2]diazaborinin-10-yl)phenyl]ethynyl}-1-(pyridin-2-yl)-1*H*-indol-3-yl)propanoyl}-*L*-phenylalanyl-*L*-alaninate (18)

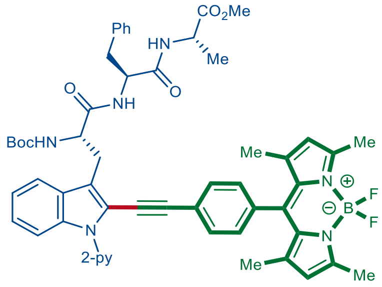

The general procedure **C** was followed using methyl N_a-(*tert*-butoxycarbonyl)-1-(pyridin-2-yl)-*L*-tryptophyl-*L*-phenylalanyl-*L*-alaninate (**4l**) (61.4 mg, 0.10 mmol), 10-[4-(bromoethynyl)phenyl]-5,5-difluoro-1,3,7,9-tetramethyl-5*H*-4*λ*4,5*λ*4-dipyrrolo[1,2-*c*:2',1'-*f*][1,3,2]diazaborinine (**B1**) (47.0 mg, 0.11 mmol), $MnBr(CO)_5$ (5.5 mg, 20 mol %), KOAc (19.6 mg, 2.0 equiv) and BPh_3 (50 µL, 0.01 M in 1,4-dioxane, 0.5 mol %) in 1,4-dioxane (1.0 mL). Purification by column chromatography on silica gel (CH_2Cl_2/MeOH: 100/0.5) yielded **18** (47.7 mg, 50%) as an orange solid.

M. p.: 127 – 132 ºC.

^{1}H NMR (300 MHz, $CDCl_3$): δ 8.76 (d, J = 3.5 Hz, 1H), 8.00 (ddd, J = 8.0, 7.8, 1.5 Hz, 1H), 7.80 (d, J = 8.0 Hz, 1H), 7.78 (d, J = 8.2 Hz, 1H), 7.75 (d, J = 7.8 Hz, 1H), 7.64 (d, J = 8.0 Hz, 2H), 7.41 (dd, J = 7.8, 3.5 Hz, 1H), 7.39 – 7.37 (m, 1H), 7.34 – 7.31 (m, 3H), 7.17 – 7.15 (m, 3H), 6.88 – 6.72 (m, 2H), 6.53 (brs, 1H), 6.24 (brd, J = 7.2 Hz, 1H), 6.02 (s, 2H), 5.55 (brs, 1H), 4.61 – 4.46 (m, 2H), 4.39 (p, J = 7.2 Hz, 1H), 3.71 (s, 3H), 3.62 (d, J = 14.3 Hz, 1H), 3.47 (dd, J = 14.3, 5.3 Hz, 1H), 3.13 – 3.08 (m, 1H), 2.58 (s, 6H), 2.24 – 2.05 (m, 1H), 1.44 (s, 6H), 1.24 (d, J = 7.2 Hz, 3H), 1.23 (s, 9H).

^{13}C NMR (100 MHz, $CDCl_3$): δ 172.4 (C_q), 171.3 (C_q), 170.8 (C_q), 169.9 (C_q), 155.9 (C_q), 150.9 (C_q), 149.2 (CH), 142.8 (C_q) 140.3 (C_q), 138.0 (CH), 136.6 (C_q), 135.9 (C_q), 135.6 (C_q), 131.8 (CH), 131.1 (C_q), 129.3 (CH), 128.5 (CH), 128.4 (CH), 127.8 (C_q), 126.9 (CH), 125.5 (CH), 123.0 (C_q), 122.2 (CH), 122.1 (CH), 121.4 (CH), 120.07 (C_q), 120.05 (CH), 119.7 (CH), 119.1 (C_q), 112.1 (CH), 98.3 (C_q), 81.8 (C_q), 80.3 (C_q), 56.2 (CH), 53.1 (CH), 52.3 (CH_3), 48.3 (CH), 36.7 (CH_2), 28.0 (CH_3), 27.5 (CH_2), 17.6 (CH_3), 14.7 (CH_3), 14.6 (CH_3).

^{19}F NMR (377 MHz, $CDCl_3$): δ –146.3 (q, $^1J_{B\text{-}F}$ = 32.2 Hz).

IR (ATR): 3401, 3297, 3060, 2976, 2925, 2853, 1742, 1650, 1542, 1509 cm^{-1}.

MS (ESI) m/z (relative intensity): 982 (100) [M+Na]$^+$, 960 (45) [M+H]$^+$.

HR-MS (ESI) m/z calcd for $C_{55}H_{56}BF_2N_7O_6Na$ [M+Na]$^+$:982.4255, found: 982.4251.

Methyl (*S*,*E*)-2-acetamido-3-{2-[4-(5,5-difluoro-1,3,7,9-tetramethyl-5*H*-4*λ*4,5*λ*4-dipyrrolo[1,2-*c*:2',1'-*f*][1,3,2]diazaborinin-10-yl)styryl]-1-(pyridin-2-yl)-1*H*-indol-3-yl}propanoate (20)

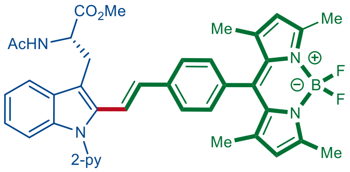

The general procedure **D** was followed using methyl N_α-acetyl-1-(pyridin-2-yl)-*L*-tryptophanate (**4a**) (34.8 mg, 0.10 mmol), 10-(4-ethynylphenyl)-5,5-difluoro-1,3,7,9-tetramethyl-5*H*-4*λ*4,5*λ*4-dipyrrolo[1,2-*c*:2',1'-*f*][1,3,2]diazaborinine (**B2**) (38.3 mg, 0.11 mmol), $MnBr(CO)_5$ (5.5 mg, 20 mol %) and (1-Ad)CO_2H (7.2 mg, 40 mol %) in 1,4-dioxane (1.0 mL). Purification by column chromatography on silica gel (CH_2Cl_2/MeOH: 100/0.2) yielded **20** (51.4 mg, 75%) as an orange solid.

M. p.: 162 – 167 ºC.

^{1}H NMR (300 MHz, $CDCl_3$): δ 8.77 (dd, J = 4.9, 1.2, 1H), 7.94 (ddd, J = 7.7, 7.7, 1.2 Hz, 1H), 7.57 (d, J = 7.7 Hz, 1H), 7.47 (d, J = 8.2 Hz, 2H), 7.46 – 7.42 (m, 2H), 7.41 (dd, J = 7.7, 4.9 Hz, 1H), 7.26 – 7.18 (m, 5H), 6.48 (d, J = 16.8 Hz, 1H), 6.14 (d, J = 7.0 Hz, 1H), 5.99 (s, 2H), 5.03 (ddd, J = 7.0, 6.7, 6.7 Hz, 1H), 3.67 – 3.60 (m, 2H), 3.55 (s, 3H), 2.57 (s, 6H), 1.88 (s, 3H), 1.42 (s, 6H).

^{13}C NMR (101 MHz, $CDCl_3$): δ 172.2 (C_q), 169.6 (C_q), 155.5 (C_q), 152.0 (C_q), 149.7 (CH), 142.9 (C_q), 141.3 (C_q), 138.5 (CH), 138.3 (C_q), 137.8 (C_q), 134.4 (C_q), 134.3 (C_q), 131.3 (C_q), 130.4 (CH), 128.9 (C_q), 128.4 (CH), 126.8 (CH), 124.0 (CH), 122.6 (CH), 122.4 (CH), 121.3 (CH), 121.2 (CH), 118.7 (CH), 117.7 (CH), 113.2 (C_q), 110.8 (CH), 52.9 (CH), 52.5 (CH_3), 27.6 (CH_2), 23.2 (CH_3), 14.6 (CH_3), 14.6 (CH_3).

^{19}F NMR (377 MHz, $CDCl_3$): δ –146.2 (q, $^1J_{B\text{-}F}$ = 32.4 Hz).

IR (ATR): 3409, 3287, 3051, 2952, 2926, 2863, 1741, 1664, 1541, 1508 cm^{-1}.

MS (ESI) m/z (relative intensity): 708 (90) [M+Na]$^+$, 686 (100) [M+H]$^+$.

HR-MS (ESI) m/z calcd for $C_{40}H_{38}BF_2N_5O_3Na$ [M+Na]$^+$:708.2935, found: 708.2924.

Benzyl (*S*,*E*)-2-[(*tert*-butoxycarbonyl)amino]-3-{2-[4-(5,5-difluoro-1,3,7,9-tetramethyl-5*H*-4λ_4,5λ_4-dipyrrolo[1,2-*c*:2',1'-*f*][1,3,2]diazaborinin-10-yl)styryl]-1-[pyridin-2-yl]-1*H*-indol-3-yl}propanoate (21)

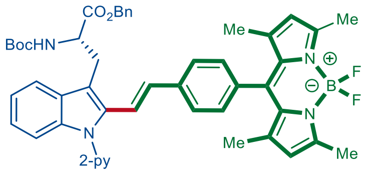

The general procedure **D** was followed using benzyl N_a-acetyl-1-(pyridin-2-yl)-*L*-tryptophanate (**4b**) (47.1 mg, 0.10 mmol), 10-(4-ethynylphenyl)-5,5-difluoro-1,3,7,9-tetramethyl-5*H*-4λ_4,5λ_4-dipyrrolo[1,2-*c*:2',1'-*f*][1,3,2]diazaborinine (**B2**) (38.3 mg, 0.11 mmol), $MnBr(CO)_5$ (5.5 mg, 20 mol %) and (1-Ad)CO_2H (7.2 mg, 40 mol %) in 1,4-dioxane (1.0 mL). Purification by column chromatography on silica gel (CH_2Cl_2/MeOH: 100/0.2) yielded **21** (57.4 mg, 70%) as an orange solid.

M. p.: 120 – 122 ºC.

^{1}H NMR (300 MHz, $CDCl_3$): δ 8.77 (d, *J* = 3.7 Hz, 1H), 7.92 (td, *J* = 7.7, 1.6 Hz, 1H), 7.64 (d, *J* = 7.7 Hz, 1H), 7.45 – 7.39 (m, 4H), 7.37 (d, *J* = 17.3 Hz, 1H), 7.32 – 7.28 (m, 1H), 7.24 – 7.17 (m, 7H), 7.10 – 7.09 (m, 2H), 6.61 (d, *J* = 17.3 Hz, 1H), 6.00 (s, 2H), 5.28 (brd, *J* = 7.3 Hz, 1H), 5.08 (d, *J* = 11.6 Hz, 1H), 4.90 (d, *J* = 11.6 Hz, 1H), 4.86 – 4.79 (m, 1H), 3.68 – 3.44 (m, 2H), 2.58 (s, 6H), 1.43 (s, 6H), 1.41 (s, 9H).

^{13}C NMR (75 MHz, $CDCl_3$): δ 172.3 (C_q), 155.4 (C_q), 155.0 (C_q), 151.9 (C_q), 149.6 (CH), 143.0 (C_q), 141.5 (C_q), 138.3 (CH), 138.1 (C_q), 137.9 (C_q), 135.0 (C_q), 134.2 (C_q), 134.1 (C_q), 131.3 (C_q), 130.5 (CH), 129.1 (C_q), 128.4 (CH), 128.3 (CH), 128.3 (CH), 128.2 (CH), 126.9 (CH), 123.9 (CH), 122.4 (CH), 122.2 (CH), 121.3 (CH), 121.1 (CH), 118.9 (CH), 118.0 (CH), 113.3 (C_q), 110.8 (CH), 79.9 (C_q), 67.4 (CH_2), 54.4 (CH), 28.9 (CH_2), 28.2 (CH_3), 14.7 (CH_3), 14.6 (CH_3).

^{19}F NMR (377 MHz, $CDCl_3$): δ –146.2 (q, $^1J_{B\text{-}F}$ = 32.4 Hz).

IR (ATR): 3421, 3054, 3033, 2972, 2927, 2859, 1711, 1542, 1509, 1469 cm^{-1}.

MS (ESI) m/z (relative intensity): 842 (100) $[M+Na]^+$, 820 (90) $[M+H]^+$.

HR-MS (ESI) m/z calcd for $C_{49}H_{48}BF_2N_5O_4Na$ $[M+Na]^+$:842.3668, found: 842.3658.

(*S*,*E*)-2-[(*tert*-butoxycarbonyl)amino]-3-{2-[4-(5,5-difluoro-1,3,7,9-tetramethyl-5*H*-4λ_4,5λ_4-dipyrrolo[1,2-*c*:2',1'-*f*][1,3,2]diazaborinin-10-yl)styryl]-1-[pyridin-2-yl]-1*H*-indol-3-yl}propanoic acid (22)

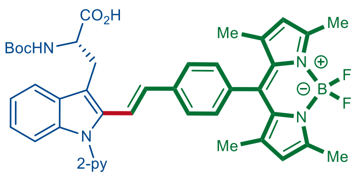

The general procedure **D** was followed using N_a-(*tert*-butoxycarbonyl)-1-(pyridin-2-yl)-*L*-tryptophan (**4m**) (38.1 mg, 0.10 mmol), 10-(4-ethynylphenyl)-5,5-difluoro-1,3,7,9-tetramethyl-5*H*-4λ_4,5λ_4-dipyrrolo[1,2-*c*:2',1'-*f*][1,3,2]diazaborinine (**B2**) (69.7 mg, 0.20 mmol), $MnBr(CO)_5$ (5.5 mg, 20 mol %) and $(1\text{-Ad})CO_2H$ (7.2 mg, 40 mol %) in 1,4-dioxane (1.0 mL). Purification by column chromatography on silica gel (Hex/EtOAc: 5/5 +1% AcOH) yielded **22** (47.4 mg, 65%) as an orange solid.

M. p.: 94 – 97 ºC.

^{1}H NMR (400 MHz, DMSO-d_6): δ 12.81 (brs, 1H), 8.74 (dd, *J* = 5.0, 1.9 Hz, 1H), 8.10 (ddd, *J* = 7.7, 7.7, 2.0 Hz, 1H), 7.82 – 7.69 (m, 1H), 7.61 – 7.53 (m, 3H), 7.50 (d, *J* = 8.0 Hz, 1H), 7.43 (d, *J* = 16.7 Hz, 1H), 7.36 – 7.29 (m, 3H), 7.28 – 7.13 (m, 3H), 6.35 (d, *J* = 16.7 Hz, 1H), 6.18 (s, 2H), 4.32 (ddd, *J* = 8.9, 8.0, 4.7 Hz, 1H), 3.46 (dd, *J* = 14.4, 4.7 Hz, 1H), 3.32 – 3.28 (m, 1H), 2.45 (s, 6H), 1.39 (s, 6H), 1.27 (s, 9H).

^{13}C NMR (101 MHz, DMSO-d_6): δ 174.1 (C_q), 155.9 (C_q), 155.3 (C_q), 151.8 (C_q), 150.1 (CH), 143.1 (C_q), 142.2 (C_q), 139.7 (CH), 138.5 (C_q), 138.2 (C_q), 134.3 (C_q), 133.6 (C_q), 131.1 (C_q), 129.9 (CH), 128.9 (C_q), 128.7 (CH), 127.3 (CH), 124.1 (CH), 123.7 (CH), 122.8 (CH), 121.8 (CH), 121.2 (CH), 119.7 (CH), 118.8 (CH), 115.8 (C_q), 110.9 (CH), 78.5 (C_q), 55.1 (CH), 28.6 (CH_3), 27.4 (CH_2), 14.7 (CH_3), 14.7 (CH_3).

^{19}F NMR (377 MHz, DMSO-d_6): δ –143.7 (q, $^1J_{B\text{-}F}$ = 31.1 Hz).

IR (ATR): 2963, 2925, 1707, 1542, 1469, 1306, 1195, 982 cm^{-1}.

MS (ESI) m/z (relative intensity): 752 (40) $[M+Na]^+$, 730 (100) $[M+H]^+$.

HR-MS (ESI) m/z calcd for $C_{42}H_{43}BF_2N_5O_4$ $[M+H]^+$:730.3378, found: 730.3356.

Methyl (*S*,*E*)-2-[(*tert*-butoxycarbonyl)amino]-3-{2-[3-(5,5-difluoro-1,3,7,9-tetramethyl-5*H*-4λ_4,5λ_4-dipyrrolo[1,2-*c*:2',1'-*f*][1,3,2]diazaborinin-10-yl)styryl]-1-[pyridin-2-yl]-1*H*-indol-3-yl}propanoate (23)

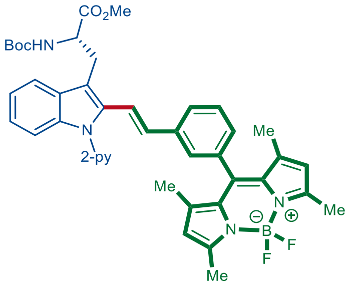

The general procedure **D** was followed using methyl N_a-(*tert*-butoxycarbonyl)-1-(pyridin-2-yl)-*L*-tryptophanate (**1**) (39.5 mg, 0.10 mmol), 10-(3-ethynylphenyl)-5,5-difluoro-1,3,7,9-tetramethyl-5*H*-4λ_4,5λ_4-dipyrrolo[1,2-*c*:2',1'-*f*][1,3,2]diazaborinine (**B4**) (38.3 mg, 0.11 mmol), $MnBr(CO)_5$ (5.5 mg, 20 mol %) and (1-Ad)CO_2H (7.2 mg, 40 mol %) in 1,4-dioxane (1.0 mL). Purification by column chromatography on silica gel (CH_2Cl_2/MeOH: 100/0.3) yielded **23** (57.2 mg, 77%) as an orange solid.

M. p.: 116 – 118 ºC.

^{1}H NMR (400 MHz, $CDCl_3$): δ 8.71 (d, J = 3.4 Hz, 1H), 7.88 (ddd, J = 7.8, 7.8, 1.5 Hz, 1H), 7.66 – 7.51 (m, 2H), 7.50 – 7.32 (m, 4H), 7.25 – 7.10 (m, 5H), 6.46 (d, J = 16.7 Hz, 1H), 6.00 (s, 2H), 5.24 (d, J = 8.3 Hz, 1H), 4.73 (ddd, J = 8.3, 7.0, 7.0 Hz, 1H), 3.70 – 3.35 (m, 5H), 2.58 (s, 6H), 1.45 – 1.34 (m, 15H).

^{13}C NMR (101 MHz, $CDCl_3$): δ 172.7 (C_q), 155.5 (C_q), 155.0 (C_q), 152.0 (C_q), 149.7 (CH), 143.0 (C_q), 141.3 (C_q), 138.3 (CH), 138.3 (C_q), 135.4 (C_q), 135.4 (C_q), 134.2 (C_q), 131.3 (C_q), 130.7 (CH), 129.4 (CH), 129.0 (C_q), 127.1 (CH), 126.3 (CH), 123.9 (CH), 122.5 (CH), 122.2 (CH), 121.2 (CH), 119.0 (CH), 117.8 (CH), 113.6 (C_q), 110.7 (CH), 79.9 (C_q), 54.4 (CH), 52.4 (CH_3), 28.8 (CH_2), 28.2 (CH_3), 14.6 (CH_3), 14.6 (CH_3). (Two aromatic CH are missing due to overlap, the overlap was verified by HSQC analysis, showing that the peaks at 126.3 and 121.2 ppm correspond to two carbons).

^{19}F NMR (282 MHz, $CDCl_3$): δ –146.30 (q, $^1J_{B\text{-}F}$ = 32.0 Hz).

IR (ATR): 2971, 2921, 1738, 1706, 1541, 1505, 1468, 1435, 1190, 1153, 973, 728 cm^{-1}.

MS (ESI) m/z (relative intensity): 766 (100) $[M+Na]^+$, 744 (5) $[M+H]^+$.

HR-MS (ESI): m/z calcd for $C_{43}H_{46}BF_2N_5O_4$ $[M+H]^+$: 744.3535, found: 744.3525.

Methyl (*S*,*E*)-2-[(*tert*-butoxycarbonyl)amino]-3-{2-[4-(5,5-difluoro-3,7-diphenyl-5*H*-4λ4,5λ4-dipyrrolo[1,2-*c*:2',1'-*f*][1,3,2]diazaborinin-10-yl)styryl]-1-[pyridin-2-yl]-1*H*-indol-3-yl}propanoate (24)

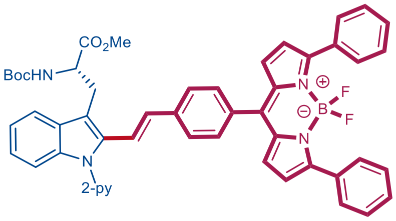

The general procedure **D** was followed using methyl N_a-(te*rt*-butoxycarbonyl)-1-(pyridin-2-yl)-*L*-tryptophanate (**1**) (39.5 mg, 0.10 mmol), 10-(4-ethynylphenyl)-5,5-difluoro-3,7-diphenyl-5*H*-4*λ4*,5*λ4*-dipyrrolo[1,2-*c*:2',1'-*f*][1,3,2]diazaborinine (**B5**) (48.9 mg, 0.11 mmol), $MnBr(CO)_5$ (5.5 mg, 20 mol %) and (1-Ad)CO_2H (7.2 mg, 40 mol %) in 1,4-dioxane (1.0 mL). Purification by column chromatography on silica gel (CH_2Cl_2/MeOH: 99/1) yielded **24**(45.3 mg, 54%) as a purple solid.

M. p.: 128 – 130 ºC.

^{1}H NMR (300 MHz, $CDCl_3$): δ 8.78 (d, *J* = 4.8 Hz, 1H), 7.99 – 7.85 (m, 5H), 7.64 (d, *J* = 6.7 Hz, 1H), 7.60 – 7.52 (m, 4H), 7.51 – 7.35 (m, 9H), 7.33 – 7.29 (m, 1H), 7.28 – 7.17 (m, 2H), 6.94 (d, *J* = 4.3 Hz, 2H), 6.66 (d, *J* = 4.3 Hz, 2H), 6.57 (d, *J* = 16.7 Hz, 1H), 5.27 (d, *J* = 8.4 Hz, 1H), 4.80 (ddd, *J* = 8.4, 6.9, 6.9 Hz, 1H), 3.69 – 3.40 (m, 5H), 1.42 (s, 9H).

^{13}C NMR (75 MHz, $CDCl_3$): δ 172.7 (C_q), 158.8 (C_q), 155.1 (C_q), 152.1 (C_q), 149.7 (CH), 143.8 (C_q), 139.4 (C_q), 138.5 (CH), 138.4 (C_q), 136.2 (C_q), 134.4 (C_q), 133.6 (C_q), 132.6 (C_q), 131.2 (CH), 130.7 (CH), 129.5 (CH), 129.5 (CH), 129.4 (CH), 129.0 (C_q), 128.2 (CH), 126.2 (CH), 124.2 (CH), 122.4 (CH), 122.3 (CH), 121.4 (CH), 120.9 (CH), 119.1 (CH), 118.9 (CH), 114.1 (C_q), 110.9 (CH), 79.9 (C_q), 54.3 (CH), 52.5 (CH_3), 28.7 (CH_3), 28.3 (CH_2).

^{19}F NMR (282 MHz, $CDCl_3$): δ –132.45 (q, $^1J_{B\text{-}F}$ = 31.7 Hz).

IR (ATR): 3439, 2923, 1739, 1707, 1561, 1537, 1466, 1435, 1135, 1066, 728 cm^{-1}.

MS (ESI) m/z (relative intensity): 862 (100) $[M+Na]^+$, 840 (71) $[M+H]^+$.

HR-MS (ESI): m/z calcd for $C_{51}H_{45}BF_2N_5O_4$ $[M+H]^+$: 840.3536, found: 840.3517.

Methyl (*S*,*E*)-2-[(*tert*-butoxycarbonyl)amino]-3-{2-[4-(5,5-difluoro-3,7-di-*p*-tolyl-5*H*-4λ4,5λ4-dipyrrolo[1,2-*c*:2',1'-*f*][1,3,2]diazaborinin-10-yl)styryl]-1-[pyridin-2-yl]-1*H*-indol-3-yl}propanoate (25)

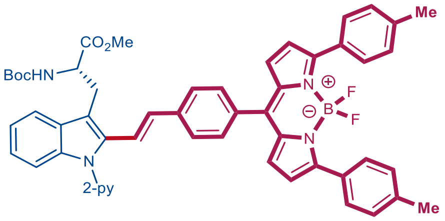

The general procedure **D** was followed using methyl N_a-(te*rt*-butoxycarbonyl)-1-(pyridin-2-yl)-*L*-tryptophanate (**1**) (39.5 mg, 0.10 mmol), 10-(4-ethynylphenyl)-5,5-difluoro-3,7-di-*p*-tolyl-5*H*-4λ_4,5λ_4-dipyrrolo[1,2-*c*:2',1'-*f*][1,3,2]diazaborinine (**B6**) (51.9 mg, 0.11 mmol), $MnBr(CO)_5$ (5.5 mg, 20 mol %) and (1-Ad)CO_2H (7.2 mg, 40 mol %) in 1,4-dioxane (1.0 mL). Purification by column chromatography on silica gel (CH_2Cl_2/MeOH: 99/1) yielded **25** (63.3 mg, 73%) as a purple solid.

M. p.: 158 – 160 °C.

^{1}H NMR (400 MHz, $CDCl_3$): δ 8.79 (d, J = 4.7 Hz, 1H), 7.93 (dd, J = 7.8, 7.8Hz, 1H), 7.83 (d, J = 7.7 Hz, 4H), 7.65 (d, J = 7.5 Hz, 1H), 7.59 – 7.48 (m, 5H), 7.44 – 7.39 (m, 2H), 7.34 (d, J = 16.6 Hz, 1H), 7.29 – 7.24 (m, 6H), 6.91 (d, J = 4.3 Hz, 2H), 6.65 (d, J = 4.3 Hz, 2H), 6.58 (d, J = 16.6 Hz, 1H), 5.28 (d, J = 8.4 Hz, 1H), 4.81 (ddd, J = 8.4, 7.3, 7.3 Hz, 1H), 3.81 – 3.45 (m, 5H), 2.42 (s, 6H), 1.43 (s, 9H).

^{13}C NMR (101 MHz, $CDCl_3$): δ 172.6 (C_q), 158.7 (C_q), 155.0 (C_q), 152.0 (C_q), 149.6 (CH), 142.9 (C_q), 139.6 (C_q), 139.1 (C_q), 138.4 (CH), 138.3 (C_q), 136.1 (C_q), 134.3 (C_q), 133.7 (C_q), 131.1 (CH), 130.6 (CH), 130.3 (CH), 129.8 (C_q), 129.3 (CH), 129.0 (CH), 129.0 (C_q), 126.1 (CH), 124.1 (CH), 122.3 (CH), 122.2 (CH), 121.3 (CH), 120.6 (CH), 119.0 (CH), 118.7 (CH), 113.9 (C_q), 110.9 (CH), 79.8 (C_q), 54.3 (CH), 52.4 (CH_3), 28.6 (CH_2), 28.2 (CH_3), 21.4 (CH_3).

^{19}F NMR (377 MHz, $CDCl_3$): δ –132.59 (q, $^1J_{B\text{-}F}$ = 32.1 Hz).

IR (ATR): 2920, 1740, 1707, 1561, 1537, 1465, 1432, 1279, 1137, 1055, 727 cm^{-1}.

MS (ESI) m/z (relative intensity): 890 (100) $[M+Na]^+$, 868 (58) $[M+H]^+$.

HR-MS (ESI): m/z calcd for $C_{53}H_{49}BF_2N_5O_4$ $[M+H]^+$: 868.3849, found: 868.3834.

Methyl (*S*,*E*)-(2-{[*tert*-butoxycarbonyl]amino}-3-{2-[4-(5,5-difluoro-1,3,7,9-tetramethyl-5*H*-4λ_4,5λ_4-dipyrrolo[1,2-*c*:2',1'-*f*][1,3,2]diazaborinin-10-yl)styryl]-1-[pyridin-2-yl]-1*H*-indol-3-yl}propanoy)glycinate (26)

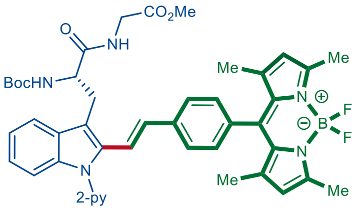

The general procedure **D** was followed using methyl N_a-(*tert*-butoxycarbonyl)-1-(pyridin-2-yl)-*L*-tryptophylglycinate (**4d**) (45.2 mg, 0.10 mmol), 10-(4-ethynylphenyl)-5,5-difluoro-1,3,7,9-tetramethyl-5*H*-4λ_4,5λ_4-dipyrrolo[1,2-*c*:2',1'-*f*][1,3,2]diazaborinine (**B2**) (38.3 mg, 0.11 mmol), $MnBr(CO)_5$ (5.5 mg, 20 mol %) and (1-Ad)CO_2H (7.2 mg, 40 mol %) in 1,4-dioxane (1.0 mL). Purification by column chromatography on silica gel (CH_2Cl_2/MeOH: 100/0.2) yielded **26** (54.4 mg, 68%) as an orange solid.

M. p.: 160 – 163 ºC.

^{1}H NMR (300 MHz, $CDCl_3$): δ 8.76 (d, *J* = 3.6 Hz, 1H), 7.95 (ddd, *J* = 7.7, 7.7, 2.0 Hz, 1H), 7.75 – 7.64 (m, 1H), 7.55 (d, *J* = 7.8 Hz, 2H), 7.49 – 7.39 (m, 3H), 7.33 (d, *J* = 16.3 Hz, 1H), 7.24 – 7.21 (m, 4H), 6.66 (d, *J* = 16.3 Hz, 1H), 6.09 (brs, 1H), 6.00 (s, 2H), 5.47 (brs, 1H), 4.59 (ddd, *J* = 7.7, 7.7, 7.6 Hz, 1H), 3.94 (dd, *J* = 18.2, 5.4 Hz, 1H), 3.74 – 3.65 (m, 1H), 3.61 (s, 3H), 3.59 – 3.55 (m, 1H), 3.48 – 3.41 (m, 1H), 2.57 (s, 6H), 1.46 – 1.43 (m, 15H)

^{13}C NMR (75 MHz, $CDCl_3$): δ 171.4 (C_q), 169.3 (C_q), 155.4 (C_q), 155.2 (C_q), 151.8 (C_q), 149.7 (CH), 143.1 (C_q), 141.5 (C_q), 138.4 (CH), 138.1 (C_q), 138.0 (C_q), 134.3 (C_q), 134.1 (C_q), 131.4 (C_q), 130.7 (CH), 128.9 (C_q), 128.3 (CH), 127.1 (CH), 124.0 (CH), 122.5 (CH), 122.3 (CH), 121.4 (CH), 121.1 (CH), 118.9 (CH), 117.9 (CH), 113.4 (C_q), 110.7 (CH), 80.1 (C_q), 55.5 (CH), 52.2 (CH_3), 41.3 (CH_2), 29.0 (CH_2), 28.3 (CH_3), 14.6 (CH_3), 14.5 (CH_3).

^{19}F NMR (282 MHz, $CDCl_3$): δ –146.2 (q, $^1J_{B-F}$ = 32.2 Hz).

IR (ATR): 3420, 3343, 3052, 2976, 2928, 2857, 1748, 1705, 1670, 1541 cm^{-1}.

MS (ESI): m/z (relative intensity): 823 (100) $[M+Na]^+$, 801 (50) $[M+H]^+$.

HR-MS (ESI) m/z calcd for $C_{45}H_{47}BF_2N_6O_5Na$ $[M+Na]^+$:823.3569; found: 823.3563.

Methyl ({*S*}-2-{[*tert*-butoxycarbonyl]amino}-3-{2-[(*E*)-4-(5,5-difluoro-1,3,7,9-tetramethyl-5*H*-4λ_4,5λ_4-dipyrrolo[1,2-*c*:2',1'-*f*][1,3,2]diazaborinin-10-yl)styryl]-1-[pyridin-2-yl]-1*H*-indol-3-yl}propanoyl)-*L*-alaninate (27)

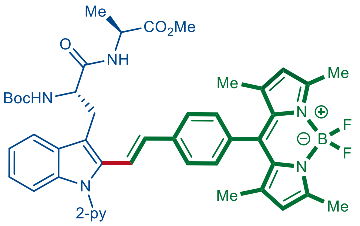

The general procedure **D** was followed using methyl N_a-(*tert*-butoxycarbonyl)-1-(pyridin-2-yl)-*L*-tryptophyl-*L*-alaninate (**4e**) (46.6 mg, 0.10 mmol), 10-(4-ethynylphenyl)-5,5-difluoro-1,3,7,9-tetramethyl-5*H*-4λ_4,5λ_4-dipyrrolo[1,2-*c*:2',1'-*f*][1,3,2]diazaborinine (**B2**) (38.3 mg, 0.11 mmol), MnBr(CO)$_5$ (5.5 mg, 20 mol %) and (1-Ad)CO_2H (7.2 mg, 40 mol %) in 1,4-dioxane (1.0 mL). Purification by column chromatography on silica gel (CH_2Cl_2/MeOH: 100/0.7) yielded **27** (59.2 mg, 73%) as an orange solid.

M. p.: 153 – 155 ºC.

^{1}H NMR (300 MHz, $CDCl_3$): δ 8.86 – 8.56 (dd, *J* = 5.0, 2.0, 1H), 7.95 (ddd, *J* = 7.7, 7.7, 2.0 Hz, 1H), 7.79 – 7.64 (m, 1H), 7.57 (d, *J* = 7.8 Hz, 2H), 7.50 (d, *J* = 8.0 Hz, 1H), 7.42 (dd, *J* = 7.7, 5.0 Hz, 1H), 7.41 – 7.37 (m, 1H), 7.34 (d, *J* = 18.2 Hz, 1H), 7.23 (d, *J* = 7.8 Hz, 2H), 7.19 – 7.16 (m, 2H), 6.71 (d, *J* = 18.2 Hz, 1H), 6.16 (d, *J* = 6.7 Hz, 1H), 6.00 (s, 2H), 5.49 (brs, 1H), 4.54 (ddd, *J* = 7.8, 7.8, 6.7 Hz, 1H), 4.42 – 4.17 (m, 1H), 3.67 – 3.59 (m, 1H), 3.50 (s, 3H), 3.40 (dd, *J* = 14.7, 7.8 Hz, 1H), 2.57 (s, 6H), 1.47 (s, 9H), 1.45 (s, 6H), 1.24 (d, *J* = 7.8 Hz, 3H).

^{13}C NMR (101 MHz, $CDCl_3$): δ 172.1 (C_q), 170.6 (C_q), 155.3 (C_q), 155.1 (C_q), 151.8 (C_q), 149.6 (CH), 143.0 (C_q), 141.5 (C_q), 138.3 (CH), 138.1 (C_q), 138.0 (C_q), 134.1 (C_q), 134.0 (C_q), 131.3 (C_q), 130.5 (CH), 128.8 (C_q), 128.2 (CH), 127.0 (CH), 123.7 (CH), 122.4 (CH), 122.3 (CH), 121.2 (CH), 121.1 (CH), 118.8 (CH), 117.9 (CH), 113.2 (C_q), 110.5 (CH), 79.9 (C_q), 55.3 (CH), 52.2 (CH_3), 48.2 (CH), 29.0 (CH_2), 28.2 (CH_3), 18.5 (CH_3), 14.6 (CH_3), 14.5 (CH_3).

^{19}F NMR (282 MHz, $CDCl_3$): δ –146.3 (q, $^1J_{B\text{-}F}$ = 32.4 Hz).

IR (ATR): 3402, 3313, 3053, 2977, 2929, 1741, 1705, 1669, 1541, 1508 cm^{-1}.

MS (ESI) m/z (relative intensity): 837 (100) $[M+Na]^+$, 815 (50) $[M+H]^+$.

HR-MS (ESI) m/z calcd for $C_{46}H_{49}BF_2N_6O_5Na$ $[M+Na]^+$:837.3726, found: 837.3715.

Methyl ({*S*)-2-{[*tert*-butoxycarbonyl]amino}-3-{2-[(*E*)-4-(5,5-difluoro-1,3,7,9-tetramethyl-5*H*-4λ_4,5λ_4-dipyrrolo[1,2-*c*:2',1'-*f*][1,3,2]diazaborinin-10-yl)styryl]-1-[pyridin-2-yl]-1*H*-indol-3-yl}propanoyl)-*L*-phenylalaninate (28)

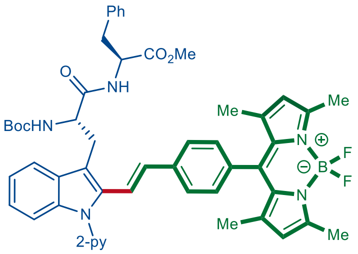

The general procedure **D** was followed using methyl N_a-(*tert*-butoxycarbonyl)-1-(pyridin-2-yl)-*L*-tryptophyl-*L*-phenylalaninate (**4i**) (54.3 mg, 0.10 mmol), 10-(4-ethynylphenyl)-5,5-difluoro-1,3,7,9-tetramethyl-5*H*-4λ_4,5λ_4-dipyrrolo[1,2-*c*:2',1'-*f*][1,3,2]diazaborinine (**B2**) (38.3 mg, 0.11 mmol), $MnBr(CO)_5$ (5.5 mg, 20 mol %) and (1-Ad)CO_2H (7.2 mg, 40 mol %) in 1,4-dioxane (1.0 mL). Purification by column chromatography on silica gel (CH_2Cl_2/MeOH: 100/0.2) yielded **28** (48.3 mg, 54%) as an orange solid. The reaction was also performed at 60 ˚C, using **B2** (69.6 mg, 0.11 mmol), and yielded **28** (46.6 mg, 52%).

M. p.: 129 – 133 ºC.

^{1}H NMR (400 MHz, $CDCl_3$): δ 8.72 (d, *J* = 3.6 Hz, 1H), 7.89 (dd, *J* = 7.7, 7.7, 1.8 Hz, 1H), 7.66 – 7.60 (m, 1H), 7.52 (d, *J* = 7.8 Hz, 2H), 7.43 (d, *J* = 7.7 Hz, 1H), 7.38 (dd, *J* = 7.7, 3.6 Hz, 1H), 7.35 – 7.33 (m, 1H), 7.30 (d, *J* = 16.7 Hz, 1H), 7.18 (d, *J* = 7.8 Hz, 2H), 7.17 – 7.14 (m, 5H), 6.87 – 6.85 (m, 2H), 6.67 (d, *J* = 16.7 Hz, 1H), 5.98 (brs, 1H), 5.96 (s, 2H), 5.45 (brs, 1H), 4.60 – 4.40 (m, 2H), 3.57 – 3.50 (m, 1H), 3.38 (s, 3H), 3.36 – 3.32 (m, 1H), 2.95 (dd, *J* = 13.7, 5.9 Hz, 1H), 2.87 (dd, *J* = 13.7, 5.0 Hz, 1H), 2.54 (s, 6H), 1.43 (s, 6H), 1.41 (s, 9H).

^{13}C NMR (100 MHz, $CDCl_3$): δ 170.7 (C_q), 170.4 (C_q), 155.3 (C_q), 155.1 (C_q), 151.7 (C_q), 149.6 (CH), 143.0 (C_q), 141.5 (C_q), 138.3 (CH), 138.0 (C_q), 135.4 (C_q), 134.1 (C_q), 134.0 (C_q), 131.3 (C_q), 130.5 (CH), 129.1 (CH), 128.9 (C_q), 128.8 (C_q), 128.3 (CH), 128.2 (CH), 127.0 (CH), 126.9 (CH), 123.8 (CH), 122.4 (CH), 122.3 (CH), 121.3 (CH), 121.1 (CH), 118.8 (CH), 117.9 (CH), 113.1 (C_q), 110.6 (CH), 79.9 (C_q), 55.4 (CH), 53.5 (CH), 52.0 (CH_3), 37.9 (CH_2), 29.1 (CH_2), 28.2 (CH_3), 14.6 (CH_3), 14.5 (CH_3).

^{19}F NMR (377 MHz, $CDCl_3$): δ –146.3 (q, $^1J_{B\text{-}F}$ = 32.1 Hz).

IR (ATR): 3397, 3316, 3057, 2975, 2928, 1741, 1707, 1672, 1541, 1508 cm^{-1}.

MS (ESI) m/z (relative intensity): 913 (100) $[M+Na]^+$, 891 (85) $[M+H]^+$.

HR-MS (ESI) m/z calcd for $C_{52}H_{53}BF_2N_6O_5Na$ $[M+Na]^+$:913.4036, found: 913.4037.

Dimethyl ({*S*}-2-{[*tert*-butoxycarbonyl]amino}-3-{2-[(*E*)-4-(5,5-difluoro-1,3,7,9-tetramethyl-5*H*-4λ_4,5λ_4-dipyrrolo[1,2-*c*:2',1'-*f*][1,3,2]diazaborinin-10-yl)styryl]-1-[pyridin-2-yl]-1*H*-indol-3-yl}propanoyl)-*L*-aspartate (29)

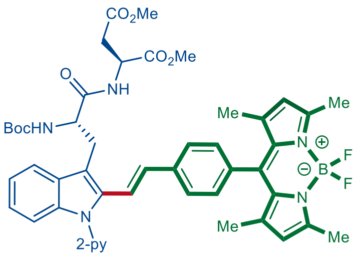

The general procedure **D** was followed using dimethyl N_a-(*tert*-butoxycarbonyl)-1-(pyridin-2-yl)-*L*-tryptophyl-*L*-aspartate (**4h**) (52.5 mg, 0.10 mmol), 10-(4-ethynylphenyl)-5,5-difluoro-1,3,7,9-tetramethyl-5*H*-4λ_4,5λ_4-dipyrrolo[1,2-*c*:2',1'-*f*][1,3,2]diazaborinine (**B2**) (38.3 mg, 0.11 mmol), $MnBr(CO)_5$ (5.5 mg, 20 mol %) and (1-Ad)CO_2H (7.2 mg, 40 mol %) in 1,4-dioxane (1.0 mL). Purification by column chromatography on silica gel (CH_2Cl_2/MeOH: 100/0.5) yielded **29** (60.1 mg, 69%) as an orange solid.

M. p.: 157 – 159 ºC.

^{1}H NMR (400 MHz, $CDCl_3$): δ 8.77 (d, J = 4.8 Hz, 1H), 7.95 (dd, J = 7.6, 7.2 Hz, 1H), 7.65 (d, J = 7.6 Hz, 1H), 7.56 (d, J = 7.1 Hz, 2H), 7.52 (d, J = 8.1 Hz, 1H), 7.44 (dd, J = 7.2, 4.8 Hz, 1H), 7.41 – 7.38 (m, 1H), 7.32 (d, J = 16.6 Hz, 1H), 7.23 (d, J = 7.1 Hz, 2H), 7.20 – 7.18 (m, 2H), 6.75 (d, J = 16.6 Hz, 1H), 6.50 (brd, J = 6.7 Hz, 1H), 6.00 (s, 2H), 5.42 (brd, J = 7.7 Hz, 1H), 4.55 (ddd, J = 7.7, 7.7, 7.6 Hz, 1H), 4.48 – 4.40 (m, 1H), 3.64 – 3.61 (m, 1H). 3.60 (s, 3H). 3.51 (s, 3H), 3.43 (dd, J = 14.4, 8.8 Hz, 1H), 2.91 – 2.76 (m, 2H), 2.58 (s, 6H), 1.47 – 1.44 (m, 15H).

^{13}C NMR (101 MHz, $CDCl_3$): δ 171.2 (C_q), 170.8 (C_q), 169.9 (C_q), 155.4 (C_q), 155.0 (C_q), 151.9 (C_q), 149.6 (CH), 143.1 (C_q), 141.5 (C_q), 138.4 (CH), 138.1 (C_q), 138.0 (C_q), 134.2 (C_q), 134.1 (C_q), 131.4 (C_q), 130.6 (CH), 129.0 (C_q), 128.3 (CH), 127.0 (CH), 123.8 (CH), 122.5 (CH), 122.4 (CH), 121.3 (CH), 121.1 (CH), 118.8 (CH), 117.9 (CH), 113.1 (C_q), 110.7 (CH), 80.0 (C_q), 55.4 (CH), 52.6 (CH_3), 51.9 (CH_3), 48.9 (CH), 36.0 (CH_2), 29.0 (CH_2), 28.3 (CH_3), 14.6 (CH_3), 14.5 (CH_3).

^{19}F NMR (283 MHz, $CDCl_3$): δ –146.31 (q, $^1J_{B\text{-}F}$ = 32.3 Hz).

IR (ATR): 3376, 2996, 2954, 2929, 1739, 1673, 1542, 1509, 1469, 1437 cm^{-1}.

MS (ESI) m/z (relative intensity): 895 (100) $[M+Na]^+$, 873 (55) $[M+H]^+$.

HR-MS (ESI): m/z calcd for $C_{48}H_{51}BF_2N_6O_7Na$ $[M+Na]^+$:895.3781, found: 895.3781.

Methyl ({*S*}-2-{[*tert*-butoxycarbonyl]amino}-3-{2-[(*E*)-4-(5,5-difluoro-1,3,7,9-tetramethyl-5H-4λ_4,5λ_4-dipyrrolo[1,2-*c*:2',1'-*f*][1,3,2]diazaborinin-10-yl)styryl]-1-[pyridin-2-yl]-1*H*-indol-3-yl}propanoyl)-*L*-tryptophanate (30)

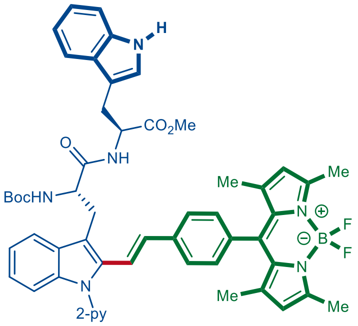

The general procedure **D** was followed using methyl N_a-(te*rt*-butoxycarbonyl)-1-(pyridin-2-yl)-*L*-tryptophyl-*L*-tryptophanate (**4j**) (52.5 mg, 0.10 mmol), 10-(4-ethynylphenyl)-5,5-difluoro-1,3,7,9-tetramethyl-5*H*-4λ_4,5λ_4-dipyrrolo[1,2-*c*:2',1'-*f*][1,3,2]diazaborinine (**B2**) (38.3 mg, 0.11 mmol), $MnBr(CO)_5$ (5.5 mg, 20 mol %) and (1-Ad)CO_2H (7.2 mg, 40 mol %) in 1,4-dioxane (1.0 mL). Purification by column chromatography on silica gel (*n*-hexane/CH_2C: 10/1) yielded **30** (67.8 mg, 73%) as an orange solid.

M. p.: 160 – 162 ºC.

^{1}H NMR (400 MHz, $CDCl_3$): δ 8.79 (dd, J = 4.9, 1.9 Hz, 1H), 8.56 (d, J = 2.4 Hz, 1H), 7.90 (ddd, J = 7.7, 7.7, 2.0 Hz, 1H), 7.74 – 7.65 (m, 1H), 7.51 – 7.40 (m, 3H), 7.39 – 7.28 (m, 4H), 7.24 (d, J = 8.2 Hz, 1H), 7.22 – 7.15 (m, 4H), 7.08 (dd, J = 7.7, 7.7 Hz, 1H), 6.94 (dd, J = 7.5, 7.5 Hz, 1H), 6.49 (d, J = 2.4 Hz, 1H), 6.41 (d, J = 16.8 Hz, 1H), 6.09 (d, J = 6.9 Hz, 1H), 5.97 (s, 2H), 5.36 (d, J = 7.5 Hz, 1H), 4.71 – 4.46 (m, 2H), 3.71 – 3.56 (m, 1H), 3.43 (s, 3H), 3.36 (dd, J = 14.4, 9.1 Hz, 1H), 3.11 (dd, J = 14.8, 5.4 Hz, 1H), 3.04 (dd, J = 14.8, 5.6 Hz, 1H), 2.55 (s, 6H), 1.48 – 1.36 (m, 15H).

^{13}C NMR (101 MHz, $CDCl_3$): δ 171.2 (C_q), 170.8 (C_q), 155.4 (C_q), 155.2 (C_q), 152.0 (C_q), 149.5 (CH), 143.1 (C_q), 141.6 (C_q), 138.7 (CH), 138.4 (C_q), 137.9 (C_q), 135.9 (C_q), 134.3 (C_q), 134.1 (C_q), 131.4 (C_q), 130.6 (CH), 128.9 (C_q), 128.2 (CH), 127.3 (C_q), 127.1 (CH), 124.0 (CH), 123.0 (CH), 122.8 (CH), 121.9 (CH), 121.4 (CH), 121.1 (CH), 119.4 (CH), 119.3 (CH), 118.3 (CH), 117.9 (CH), 113.4 (C_q), 111.1 (CH), 110.6 (CH), 109.3 (C_q), 80.0 (C_q), 55.3 (CH), 53.0 (CH), 52.1

(CH_3), 28.5 (CH_2), 28.3 (CH_3), 27.3 (CH_2), 14.6 (CH_3), 14.6 (CH_3). (One aromatic CH is missing due to overlap, the overlap was verified by HSQC analysis, showing that the peak at 111.1 corresponds to two carbons).

^{19}F NMR (377 MHz, $CDCl_3$): δ –146.26 (q, $^1J_{B\text{-}F}$ = 32.0 Hz).

IR (ATR): 1670, 1509, 1455, 1195, 1156, 904, 723, 647 cm^{-1}.

MS (ESI) m/z (relative intensity): 952 (100) $[M+Na]^+$, 930 (45) $[M+H]^+$.

HR-MS (ESI) m/z calcd for $C_{54}H_{55}BF_2N_7O_5$ $[M+H]^+$:930.4329, found: 930.4330.

Methyl ({*S*}-2-{[*tert*-butoxycarbonyl]amino}-3-{2-[(*E*)-4-(5,5-difluoro-1,3,7,9-tetramethyl-5*H*-4λ_4,5λ_4-dipyrrolo[1,2-*c*:2',1'-*f*][1,3,2]diazaborinin-10-yl)styryl]-1-[pyridin-2-yl]-1*H*-indol-3-yl}propanoyl0-*L*-tyrosinate (31)

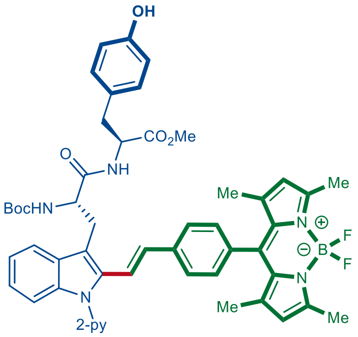

The general procedure **D** was followed using methyl N_a-(*tert*-butoxycarbonyl)-1-(pyridin-2-yl)-*L*-tryptophyl-*L*-tyrosinate (**4k**) (55.7 mg, 0.10 mmol), 10-(4-ethynylphenyl)-5,5-difluoro-1,3,7,9-tetramethyl-5*H*-4λ_4,5λ_4-dipyrrolo[1,2-*c*:2',1'-*f*][1,3,2]diazaborinine (**B2**) (38.3 mg, 0.11 mmol), $MnBr(CO)_5$ (5.5 mg, 20 mol %) and (1-Ad)CO_2H (7.2 mg, 40 mol %) in 1,4-dioxane (1.0 mL). Purification by column chromatography on silica gel (CH_2Cl_2/MeOH: 100/0.7) yielded **31** (61.1 mg, 67%) as an orange solid.

M. p.: 158 – 162 ºC.

^{1}H NMR (400 MHz, $CDCl_3$): δ 8.77 (dd, J = 4.8, 1.9 Hz, 1H), 7.94 (ddd, J = 7.8, 7.7, 1.9 Hz, 1H), 7.64 (dd, J = 7.7, 1.9 Hz, 1H), 7.53 (d, J = 7.7 Hz, 2H), 7.48 – 7.41 (m, 2H), 7.35 (dd, J = 7.7, 4.8 Hz, 1H), 7.34 (d, J = 18.2 Hz, 1H), 7.21 (d, J = 7.7 Hz, 2H), 7.19 – 7.15 (m, 2H), 6.65 (d, J = 8.4 Hz, 2H), 6.56 (d, J = 8.4 Hz, 2H), 6.52 (d, J = 18.2 Hz, 1H), 6.45 (brs, 1H), 6.06 (brs, 1H), 5.99 (s, 2H), 5.39 (brs, 1H), 4.60 – 4.53 (m, 1H), 4.49 (ddd, J = 5.8, 5.8, 5.7 Hz, 1H), 3.62 (d, J = 12.4

Hz, 1H), 3.47 (s, 3H), 3.35 (dd, J = 12.4, 9.3 Hz, 1H), 2.90 (dd, J = 13.9, 5.7 Hz, 1H), 2.80 (dd, J = 13.9, 5.8 Hz, 1H), 2.57 (s, 6H), 1.46 (s, 9H), 1.44 (s, 6H).

^{13}C NMR (101 MHz, $CDCl_3$): δ 170.9 (C_q), 170.7 (C_q), 155.4 (C_q), 155.2 (C_q), 155.0 (C_q), 151.9 (C_q), 149.4 (CH), 143.1 (C_q), 141.5 (C_q), 138.7 (CH), 138.3 (C_q), 137.9 (C_q), 134.3 (C_q), 134.1 (C_q), 131.3 (C_q), 130.5 (CH), 130.1 (CH), 128.8 (C_q), 128.2 (CH), 127.1 (CH), 127.0 (C_q), 123.9 (CH), 122.8 (CH), 122.7 (CH), 121.4 (CH), 121.1 (CH), 119.1 (CH), 117.8 (CH), 115.6 (CH), 113.4 (C_q), 110.5 (CH), 80.1 (C_q), 55.3 (CH), 53.5 (CH), 52.1 (CH_3), 36.9 (CH_2), 28.5 (CH_2), 28.3 (CH_3), 14.7 (CH_3), 14.6 (CH_3).

^{19}F NMR (377 MHz, $CDCl_3$): δ – 146.24 (q, $^{1}J_{B\text{-}F}$ = 31.7 Hz).

IR (ATR): 3388, 3300, 2971, 2926, 2856, 1741, 1709, 1670, 1542, 1509 cm^{-1}.

MS (ESI) m/z (relative intensity): 929 (100) $[M+Na]^+$, 907 (90) $[M+H]^+$.

HR-MS (ESI) m/z calcd for $C_{52}H_{53}BF_2N_6O_6Na$ $[M+Na]^+$:929.3989, found: 929.3982.

Methyl ({*S*}-2-{[*tert*-butoxycarbonyl]amino}-3-{2-[(*E*)-4-(5,5-difluoro-1,3,7,9-tetramethyl-5*H*-4λ_4,5λ_4-dipyrrolo[1,2-*c*:2',1'-*f*][1,3,2]diazaborinin-10-yl)styryl]-1-[pyridin-2-yl]-1*H*-indol-3-yl}propanoyl)-*L*-serinate (32)

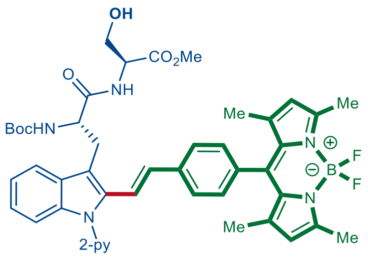

The general procedure **D** was followed using methyl N_a-(*tert*-butoxycarbonyl)-1-(pyridin-2-yl)-*L*-tryptophyl-*L*-serinate (**4n**) (48.2 mg, 0.10 mmol), 10-(4-ethynylphenyl)-5,5-difluoro-1,3,7,9-tetramethyl-5*H*-4λ_4,5λ_4-dipyrrolo[1,2-*c*:2',1'-*f*][1,3,2]diazaborinine (**B2**) (69.7 mg, 0.20 mmol), $MnBr(CO)_5$ (5.5 mg, 20 mol %) and (1-Ad)CO_2H (7.2 mg, 40 mol %) in 1,4-dioxane (1.0 mL). Purification by column chromatography on silica gel (CH_2Cl_2/EtOAc: 7/3) yielded **32** (65.6 mg, 79%) as an orange solid.

M. p.: 158 – 160 ºC.

^{1}H NMR (400 MHz, $CDCl_3$): δ 8.68 (dd, J = 4.8, 2.0 Hz, 1H), 8.05 (ddd, J = 7.7, 7.7, 1.9 Hz, 1H), 7.72 – 7.66 (m, 1H), 7.63 (d, J = 7.9 Hz, 1H), 7.52 – 7.36 (m, 4H), 7.34 – 7.29 (m, 1H), 7.27 – 7.18 (m, 4H), 6.75 (d, J = 6.6 Hz, 1H), 6.27 (d, J = 16.7 Hz, 1H), 6.00 (s, 2H), 5.36 (d, J = 7.7 Hz,

1H), 4.66 (ddd, J = 7.7, 7.4, 4.2 Hz, 1H), 4.51 – 4.41 (m, 1H), 3.91 – 3.82 (m, 2H), 3.81 – 3.71 (m, 2H), 3.64 (s, 3H), 3.42 (dd, J = 14.6, 7.4 Hz, 1H), 2.57 (s, 6H), 1.47 (s, 9H), 1.44 (s, 6H).

^{13}C NMR (101 MHz, $CDCl_3$): δ 171.6 (C_q), 170.1 (C_q), 155.5 (C_q), 155.3 (C_q), 151.7 (C_q), 149.8 (CH), 143.1 (C_q), 141.5 (C_q), 139.1 (CH), 138.6 (C_q), 137.9 (C_q), 134.4 (C_q), 134.3 (C_q), 131.4 (C_q), 130.4 (CH), 129.0 (C_q), 128.4 (CH), 127.0 (CH), 124.3 (CH), 123.0 (CH), 122.4 (CH), 121.7 (CH), 121.2 (CH), 118.8 (CH), 117.9 (CH), 113.9 (C_q), 110.4 (CH), 80.4 (C_q), 62.3 (CH_2), 55.8 (CH), 55.2 (CH), 52.6 (CH_3), 28.3 (CH_3), 27.5 (CH_2), 14.6 (CH_3), 14.6 (CH_3).

^{19}F NMR (377 MHz, $CDCl_3$): δ –146.3 (q, $^{1}J_{B\text{-}F}$ = 32.1 Hz).

IR (ATR): 3410, 2955, 2925, 1744, 1705, 1674, 1542, 1470, 1195, 1157, 982 cm^{-1}.

MS (ESI) m/z (relative intensity): 853 (65) $[M+Na]^+$, 831 (100) $[M+H]^+$.

HR-MS (ESI) m/z calcd for $C_{46}H_{50}BF_2N_6O_6$ $[M+H]^+$:831.3855, found: 831.3842.

Methyl ({*S*}-2-{[*tert*-butoxycarbonyl]amino}-3-{2-[(*E*)-4-(5,5-difluoro-1,3,7,9-tetramethyl-5*H*-4λ_4,5λ_4-dipyrrolo[1,2-*c*:2',1'-*f*][1,3,2]diazaborinin-10-yl)styryl]-1-[pyridin-2-yl]-1*H*-indol-3-yl}propanoyl)-*L*-threoninate (33)

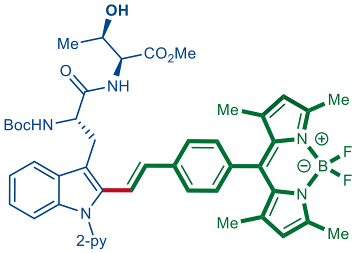

The general procedure **D** was followed using methyl N_a-(*tert*-butoxycarbonyl)-1-(pyridin-2-yl)-*L*-tryptophyl-*L*-threoninate (**4o**) (49.6 mg, 0.10 mmol), 10-(4-ethynylphenyl)-5,5-difluoro-1,3,7,9-tetramethyl-5*H*-4λ_4,5λ_4-dipyrrolo[1,2-*c*:2',1'-*f*][1,3,2]diazaborinine (**B2**) (69.7 mg, 0.20 mmol), $MnBr(CO)_5$ (5.5 mg, 20 mol %) and $(1\text{-Ad})CO_2H$ (7.2 mg, 40 mol %) in 1,4-dioxane (1.0 mL). Purification by column chromatography on silica gel (CH_2Cl_2/EtOAc: 7/3) yielded **33** (70.0 mg, 83%) as an orange solid.

M. p.: 157 – 160 ºC.

^{1}H NMR (400 MHz, $CDCl_3$): δ 8.71 (dd, J = 5.0, 1.9 Hz, 1H), 7.96 (ddd, J = 7.7, 7.7, 2.0 Hz, 1H), 7.76 – 7.63 (m, 1H), 7.56 – 7.48 (m, 3H), 7.45 – 7.39 (m, 1H), 7.36 – 7.28 (m, 2H), 7.23 – 7.15 (m, 4H), 6.57 (d, J = 16.7 Hz, 1H), 6.42 (d, J = 7.6 Hz, 1H), 5.97 (s, 2H), 5.48 (d, J = 7.0 Hz, 1H), 4.58 (ddd, J = 8.0, 7.0, 5.3 Hz, 1H), 4.30 (dd, J = 7.6, 3.5 Hz, 1H), 4.12 – 3.99 (m, 1H), 3.59 (dd,

J = 14.3, 5.3 Hz, 1H), 3.52 – 3.37 (m, 4H), 2.79 (d, J = 6.0 Hz, 1H), 2.55 (s, 6H), 1.44 (s, 9H), 1.42 (s, 6H), 1.06 (d, J = 6.4 Hz, 3H).

^{13}C NMR (101 MHz, $CDCl_3$): δ 171.8 (C_q), 170.2 (C_q), 155.5 (C_q), 155.3 (C_q), 151.9 (C_q), 149.7 (CH), 143.1 (C_q), 141.6 (C_q), 138.7 (CH), 138.3 (C_q), 138.0 (C_q), 134.3 (C_q), 134.2 (C_q), 131.4 (C_q), 130.7 (CH), 128.8 (C_q), 128.3 (CH), 127.1 (CH), 124.0 (CH), 122.7 (CH), 122.5 (CH), 121.4 (CH), 121.2 (CH), 119.1 (CH), 118.0 (CH), 113.6 (C_q), 110.5 (CH), 80.2 (C_q), 68.3 (CH), 57.8 (CH), 55.5(CH), 52.4 (CH_3), 28.7 (CH_2), 28.3 (CH_3), 19.9 (CH_3), 14.7 (CH_3), 14.6 (CH_3).

^{19}F NMR (377 MHz, $CDCl_3$): δ – 146.3 (q, $^1J_{B\text{-}F}$ = 32.3 Hz).

IR (ATR): 3423, 2976, 2926, 1742, 1706, 1671, 1542, 1469, 1155, 906, 727 cm^{-1}.

MS (ESI) m/z (relative intensity): 867 (55) $[M+Na]^+$, 845 (100) $[M+H]^+$.

HR-MS (ESI) m/z calcd for $C_{47}H_{52}BF_2N_6O_6$ $[M+H]^+$:845.4012, found: 845.3995.

(*S*)-2-{[*S*]-2-[(*tert*-butoxycarbonyl)amino]-3-methylbutanamido}-3-{2-[(*E*)-4-(5,5-difluoro-1,3,7,9-tetramethyl-5*H*-4λ_4,5λ_4-dipyrrolo[1,2-*c*:2',1'-*f*][1,3,2]diazaborinin-10-yl)styryl]-1-[pyridin-2-yl]-1*H*-indol-3-yl}propanoic acid (34)

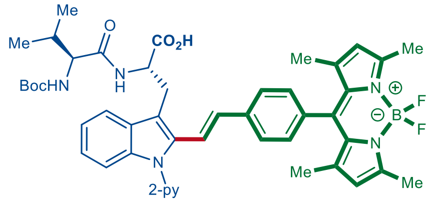

The general procedure **D** was followed using N_a-[(*tert*-butoxycarbonyl)-*L*-valyl]-1-(pyridin-2-yl)-*L*-tryptophan (**4p**) (48.0 mg, 0.10 mmol), 10-(4-ethynylphenyl)-5,5-difluoro-1,3,7,9-tetramethyl-5*H*-4λ_4,5λ_4-dipyrrolo[1,2-*c*:2',1'-*f*][1,3,2]diazaborinine (**B2**) (69.7 mg, 0.20 mmol), $MnBr(CO)_5$ (5.5 mg, 20 mol %) and (1-Ad)CO_2H (7.2 mg, 40 mol %) in 1,4-dioxane (1.0 mL). Purification by column chromatography on silica gel (CH_2Cl_2/EtOAc: 9/1→7/3 +1% AcOH) yielded **34** (42.1 mg, 51%) as an orange solid.

M. p.: 105 – 107 °C.

^{1}H NMR (400 MHz, DMSO-d_6): δ 12.88 (brs, 1H), 8.73 (dd, J = 5.0, 1.8 Hz, 1H), 8.36 (d, J = 8.3 Hz, 1H), 8.08 (ddd, J = 7.7, 7.7, 1.9 Hz, 1H), 7.78 – 7.69 (m, 1H), 7.60 – 7.53 (m, 3H), 7.50 (d, J = 8.0 Hz, 1H), 7.37 (d, J = 16.7 Hz, 1H), 7.34 – 7.27 (m, 3H), 7.23 – 7.12 (m, 2H), 6.56 (d, J = 9.2 Hz, 1H), 6.48 (d, J = 16.7 Hz, 1H), 6.18 (s, 2H), 4.72 (ddd, J = 8.0, 7.3, 6.8 Hz, 1H), 3.83 (dd,

J = 9.2, 7.0 Hz, 1H), 3.48 (dd, J = 14.3, 6.8 Hz, 1H), 3.28 (dd, J = 14.3, 7.3 Hz, 1H), 2.45 (s, 6H), 1.90 (h, J = 6.9 Hz, 1H), 1.40 (s, 6H), 1.35 (s, 9H), 0.82 – 0.65 (m, 6H).

^{13}C NMR (101 MHz, DMSO-d_6): δ 173.6 (C_q), 171.8 (C_q), 155.7 (C_q), 155.3 (C_q), 151.6 (C_q), 150.1 (CH), 143.1 (C_q), 142.2 (C_q), 139.7 (CH), 138.3 (C_q), 138.2 (C_q), 134.5 (C_q), 133.6 (C_q), 131.1 (C_q), 130.3 (CH), 128.8 (C_q), 128.7 (CH), 127.4 (CH), 124.0 (CH), 123.6 (CH), 122.7 (CH), 121.8 (CH), 121.3 (CH), 119.6 (CH), 118.8 (CH), 114.7 (C_q), 111.0 (CH), 78.5 (C_q), 60.3 (CH), 53.2 (CH), 31.1 (CH), 28.6 (CH_3), 28.1 (CH_2), 19.6 (CH_3), 18.5 (CH_3), 14.7 (CH_3), 14.7 (CH_3).

^{19}F NMR (377 MHz, DMSO-d_6): δ –143.7 (q, $^{1}J_{B\text{-}F}$ = 30.6 Hz).

IR (ATR): 2960, 2925, 1713, 1672, 1543, 1470, 1195, 1157, 984 cm^{-1}.

MS (ESI) m/z (relative intensity): 851 (60) $[M+Na]^+$, 829 (100) $[M+H]^+$.

HR-MS (ESI) m/z calcd for $C_{47}H_{52}BF_2N_6O_5$ $[M+H]^+$:829.4063, found: 829.4044.

Benzyl (*S*)-2-({*S*}-6-{[(benzyloxy)carbonyl}amino}-2-{[*tert*-butoxycarbonyl]amino}hexanamido)-3-{2-[(*E*)-4-(5,5-difluoro-1,3,7,9-tetramethyl-5*H*-4λ_4,5λ_4-dipyrrolo[1,2-*c*:2',1'-*f*][1,3,2]diazaborinin-10-yl)styryl]-1-[pyridin-2-yl]-1*H*-indol-3-yl}propanoate (35)

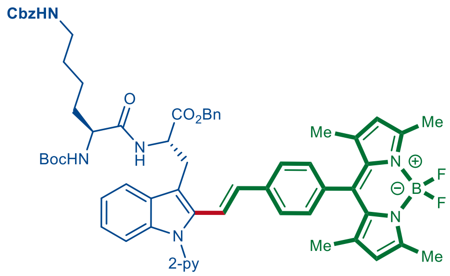

The general procedure **D** was followed using benzyl N_a-(N_6-((benzyloxy)carbonyl)-N_2-(*tert*-butoxycarbonyl)-*L*-lysyl)-1-(pyridin-2-yl)-*L*-tryptophanate (**4q**) (73.4 mg, 0.10 mmol), 10-(4-ethynylphenyl)-5,5-difluoro-1,3,7,9-tetramethyl-5*H*-4λ_4,5λ_4-dipyrrolo[1,2-*c*:2',1'-*f*][1,3,2]diazaborinine (**B2**) (69.6 mg, 0.20 mmol), $MnBr(CO)_5$ (5.5 mg, 20 mol %) and (1-Ad)CO_2H (7.2 mg, 40 mol %) in 1,4-dioxane (1.0 mL). Purification by column chromatography on silica gel (CH_2Cl_2/EtOAc: 10/1→10/2) yielded **35** (63.8 mg, 59%) as an orange solid.

M. p.: 132 – 134 ºC.

^{1}H NMR (400 MHz, $CDCl_3$): δ 8.75 (dd, J = 5.0, 1.9 Hz, 1H), 7.91 (ddd, J = 7.7, 7.7, 2.0 Hz, 1H), 7.68 – 7.57 (m, 1H), 7.46 – 7.38 (m, 4H), 7.38 – 7.29 (m, 6H), 7.27 – 7.21 (m, 5H), 7.21 – 7.16 (m, 3H), 7.11 – 6.99 (m, 2H), 6.80 (d, J = 7.7 Hz, 1H), 6.66 (d, J = 16.7 Hz, 1H), 5.99 (s, 2H),

5.19 – 5.01 (m, 5H), 5.02 – 4.94 (m, 1H), 4.87 (d, J = 12.2 Hz, 1H), 4.19 – 3.99 (m, 1H), 3.66 – 3.43 (m, 2H), 3.19 – 3.01 (m, 2H), 2.57 (s, 6H), 1.76 – 1.68 (m, 1H), 1.55 – 1.35 (m, 18H), 1.33 – 1.16 (m, 2H).

^{13}C NMR (101 MHz, $CDCl_3$): δ 171.8 (C_q), 171.7 (C_q), 156.5 (C_q), 155.6 (C_q), 155.4 (C_q), 151.7 (C_q), 149.7 (CH), 143.0 (C_q), 141.4 (C_q), 138.4 (CH), 137.9 (C_q), 137.8 (C_q), 136.6 (C_q), 134.7 (C_q), 134.2 (C_q), 134.2 (C_q), 131.3 (C_q), 130.7 (CH), 129.0 (C_q), 128.5 (CH), 128.4 (CH), 128.3 (CH), 128.3 (CH), 128.2 (CH), 128.1 (CH), 128.0 (CH), 126.9 (CH), 124.0 (CH), 122.5 (CH), 122.1 (CH), 121.4 (CH), 121.2 (CH), 118.6 (CH), 117.9 (CH), 112.8 (C_q), 110.9 (CH), 80.1 (C_q), 67.6 (CH_2), 66.6 (CH_2), 54.3 (CH), 53.0 (CH), 40.3 (CH_2), 31.7 (CH_2), 29.3 (CH_2), 28.4 (CH_2), 28.2 (CH_3), 22.1 (CH_2), 14.6 (CH_3), 14.6 (CH_3).

^{19}F NMR (377 MHz, $CDCl_3$): δ –146.26 (q, $^{1}J_{B\text{-}F}$ = 31.7 Hz).

IR (ATR): 3334, 2929, 1699, 1540, 1507, 1468, 1191, 1154, 972, 740 cm^{-1}.

MS (ESI) m/z (relative intensity): 1104 (100) $[M+Na]^+$, 1082 (24) $[M+H]^+$.

HR-MS (ESI): m/z calcd for $C_{63}H_{67}BF_2N_7O_7$ $[M+H]^+$: 1082.5168, found: 1082.5149.

Methyl (*S*)-2-({*S*}-6-amino-2-{[(benzyloxy)carbonyl]amino}hexanamido)-3-{2-[(*E*)-4-(5,5-difluoro-1,3,7,9-tetramethyl-5*H*-4λ_4,5λ_4-dipyrrolo[1,2-*c*:2',1'-*f*][1,3,2]diazaborinin-10-yl)styryl]-1-[pyridin-2-yl]-1*H*-indol-3-yl}propanoate (36)

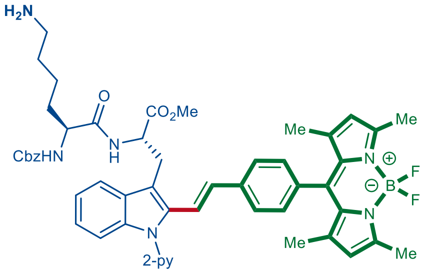

The general procedure **D** was followed using methyl N_a-{[(benzyloxy)carbonyl]-*L*-lysyl}-1-(pyridin-2-yl)-*L*-tryptophanate (**4r**) (55.7 mg, 0.10 mmol), 10-(4-ethynylphenyl)-5,5-difluoro-1,3,7,9-tetramethyl-5*H*-4λ_4,5λ_4-dipyrrolo[1,2-*c*:2',1'-*f*][1,3,2]diazaborinine (**B2**) (69.7 mg, 0.20 mmol), $MnBr(CO)_5$ (27.4 mg, 100 mol %) and (1-Ad)CO_2H (25.3 mg, 140 mol %) in 1,4-dioxane (2.0 mL). Purification by column chromatography on silica gel (CH_2Cl_2/MeOH: 98/2→95/5 + 3% NEt_3) yielded **36** (59.9 mg, 66%) as an orange solid.

M. p.: 92 – 94 ºC.

^{1}**H NMR** (400 MHz, DMSO-d_6): δ 8.73 (d, J = 5.1 Hz, 1H), 8.60 (d, J = 7.9 Hz, 1H), 8.05 (ddd, J = 7.8, 7.8, 2.0 Hz, 1H), 7.70 – 7.61 (m, 1H), 7.61 – 7.48 (m, 4H), 7.40 – 7.22 (m, 10H), 7.20 – 7.13 (m, 2H), 6.42 (d, J = 16.7 Hz, 1H), 6.17 (s, 2H), 5.02 (d, J = 12.6 Hz, 1H), 4.95 (d, J = 12.6 Hz, 1H), 4.69 (dt, J = 7.9, 7.4 Hz, 1H), 4.06 (ddd, J = 8.6, 8.6, 5.0 Hz, 1H), 3.56 – 3.38 (m, 9H), 2.45 (s, 6H), 1.68 – 1.42 (m, 3H), 1.37 (s, 6H), 1.34 – 1.17 (m, 3H).

^{13}**C NMR** (101 MHz, DMSO-d_6): δ 172.2 (C_q), 172.0 (C_q), 155.8 (C_q), 154.9 (C_q), 151.1 (C_q), 149.6 (CH), 142.6 (C_q), 141.7 (C_q), 139.2 (CH), 137.7 (C_q), 137.6 (C_q), 136.9 (C_q), 134.0 (C_q), 133.2 (C_q), 130.6 (C_q), 130.0 (CH), 128.2 (CH), 128.2 (C_q), 127.7 (CH), 127.6 (CH), 126.9 (CH), 123.6 (CH), 123.1 (CH), 122.2 (CH), 121.3 (CH), 120.9 (CH), 118.9 (CH), 118.3 (CH), 113.7 (C_q), 110.6 (CH), 69.8 (CH_2), 65.4 (CH_2), 54.6 (CH), 52.9 (CH), 51.9 (CH_3), 40.7 (CH_2), 31.7 (CH_2), 31.4 (CH_2), 27.2 (CH_2), 14.2 (CH_3), 14.2 (CH_3). (One aromatic CH is missing due to overlap, the overlap was verified by HSQC analysis, showing that the peak at 128.2 corresponds to two CH carbons)

^{19}**F NMR** (377 MHz, $CDCl_3$): δ –143.7 (q, $^1J_{B\text{-}F}$ = 30.8 Hz).

IR (ATR): 3260, 3248, 3032, 2927, 2859, 1716, 1669, 1540, 1468, 1435, 1193, 1155, 1024, 982, 741 cm^{-1}.

MS (ESI) m/z (relative intensity): 906 (60) $[M+H]^+$.

HR-MS (ESI) m/z calcd for $C_{52}H_{55}BF_2N_7O_5$ $[M+H]^+$:906.4329, found: 906.4352.

Ethyl ({*S*}-2-{[*tert*-butoxycarbonyl]amino}-3-{2-[(*E*)-4-(5,5-difluoro-1,3,7,9-tetramethyl-5*H*-4λ_4,5λ_4-dipyrrolo[1,2-*c*:2',1'-*f*][1,3,2]diazaborinin-10-yl)styryl]-1-[pyridin-2-yl]-1*H*-indol-3-yl}propanoyl)-*L*-leucylglycinate (37)

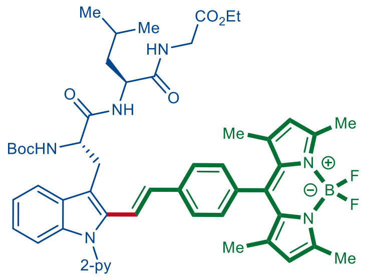

The general procedure **D** was followed using ethyl N_a-(*tert*-butoxycarbonyl)-1-(pyridin-2-yl)-*L*-tryptophyl-*L*-leucylglycinate (**4s**) (58.0 mg, 0.10 mmol), 10-(4-ethynylphenyl)-5,5-difluoro-1,3,7,9-tetramethyl-5*H*-4λ_4,5λ_4-dipyrrolo[1,2-*c*:2',1'-*f*][1,3,2]diazaborinine (**B2**) (69.6 mg, 0.20 mmol), $MnBr(CO)_5$ (5.5 mg, 20 mol %) and (1-Ad)CO_2H (7.2 mg, 40 mol %) in 1,4-dioxane (1.0

mL). Purification by column chromatography on silica gel (CH_2Cl_2/EtOAc: 20/1→10/1) yielded **37** (65.9 mg, 71%) as an orange solid.

M. p.: 156 – 158 ºC.

^{1}H NMR (400 MHz, $CDCl_3$): δ 8.76 (dd, J = 5.0, 1.9 Hz, 1H), 7.96 (ddd, J = 7.7, 7.7, 1.9 Hz, 1H), 7.72 – 7.67 (m, 1H), 7.54 (d, J = 7.9 Hz, 2H), 7.50 (d, J = 8.0 Hz, 1H), 7.44 (dd, J = 7.5, 4.9 Hz, 1H), 7.40 – 7.36 (m, 1H), 7.31 (d, J = 16.9 Hz, 1H), 7.26 – 7.16 (m, 4H), 6.70 (d, J = 16.7 Hz, 1H), 6.31 – 6.14 (m, 2H), 5.99 (s, 2H), 5.45 – 5.29 (m, 1H), 4.57 (ddd, J = 7.2, 7.2, 7.2 Hz, 1H), 4.39 (ddd, J = 8.6, 8.6, 5.5 Hz, 1H), 4.14 (q, J = 7.2 Hz, 2H), 3.81 – 3.65 (m, 2H), 3.65 – 3.42 (m, 2H), 2.57 (s, 6H), 1.69 – 1.50 (m, 2H), 1.49 – 1.36 (m, 16H), 1.25 (t, J = 7.2 Hz, 3H), 0.87 (d, J = 6.4 Hz, 6H).

^{13}C NMR (101 MHz, $CDCl_3$): δ 171.4 (C_q), 171.3 (C_q), 169.4 (C_q), 155.6 (C_q), 155.4 (C_q), 151.7 (C_q), 149.7 (CH), 143.0 (C_q), 141.4 (C_q), 138.5 (CH), 138.0 (C_q), 137.9 (C_q), 134.3 (C_q), 134.2 (C_q), 131.3 (C_q), 130.9 (CH), 128.7 (C_q), 128.3 (CH), 127.1 (CH), 123.9 (CH), 122.6 (CH), 122.4 (CH), 121.4 (CH), 121.1 (CH), 118.9 (CH), 117.9 (CH), 112.8 (C_q), 110.7 (CH), 80.4 (C_q), 61.3 (CH_2), 55.8 (CH), 51.7 (CH), 41.2 (CH_2), 41.1 (CH_2), 28.2 (CH_3), 24.6 (CH), 22.8 (CH_3), 22.0 (CH_3), 14.6 (CH_3), 14.5 (CH_3), 14.1 (CH_3). (One aliphatic CH_2 is missing due to overlap, the overlap was verified by HSQC analysis, showing that the peak at 28.2 corresponds to two carbons, the CH_2 of the Trp side chain and the CH_3 of the Boc group).

^{19}F NMR (377 MHz, $CDCl_3$): δ –146.28 (q, $^1J_{B\text{-}F}$ = 31.8 Hz).

IR (ATR): 3302, 2959, 2930, 1740, 1656, 1507, 1468, 1436, 1191, 1154, 974, 741 cm^{-1}.

MS (ESI) m/z (relative intensity): 950 (100) $[M+Na]^+$, 928 (29) $[M+H]^+$.

HR-MS (ESI) m/z calcd for $C_{52}H_{61}BF_2N_7O_6$ $[M+H]^+$: 928.4748, found: 928.4739.

Methyl ({*S*}-2-{[*tert*-butoxycarbonyl]amino}-3-{2-[(*E*)-4-(5,5-difluoro-1,3,7,9-tetramethyl-5H-4λ_4,5λ_4-dipyrrolo[1,2-*c*:2',1'-*f*][1,3,2]diazaborinin-10-yl)styryl]-1-[pyridin-2-yl]-1*H*-indol-3-yl}propanoyl)-*L*-valyl-*L*-alaninate (38)

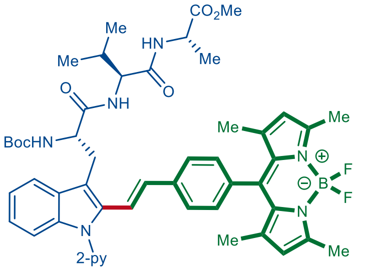

The general procedure **D** was followed using methyl N_a-(*tert*-butoxycarbonyl)-1-(pyridin-2-yl)-*L*-tryptophyl-*L*-valyl-*L*-alaninate (**4t**) (56.5 mg, 0.10 mmol), 10-(4-ethynylphenyl)-5,5-difluoro-1,3,7,9-tetramethyl-5*H*-4λ_4,5λ_4-dipyrrolo[1,2-*c*:2',1'-*f*][1,3,2]diazaborinine (**B2**) (69.6 mg, 0.11 mmol), $MnBr(CO)_5$ (5.5 mg, 20 mol %) and (1-Ad)CO_2H (7.2 mg, 40 mol %) in 1,4-dioxane (1.0 mL). Purification by column chromatography on silica gel (CH_2Cl_2/EtOAc: 20/1→10/1) yielded **38** (57.5 mg, 63%) as an orange solid. The reaction was also performed at 60 ˚C, and yielded **38** (53.9 mg, 59%).

M. p.: 192 – 194 ºC.

^{1}H NMR (400 MHz, $CDCl_3$): δ 8.78 – 8.70 (m, 1H), 7.93 (ddd, *J* = 7.7, 7.7, 2.1 Hz, 1H), 7.72 – 7.62 (m, 1H), 7.55 – 7.47 (m, 3H), 7.45 – 7.26 (m, 3H), 7.24 – 7.11 (m, 4H), 6.68 (d, *J* = 16.7 Hz, 1H), 6.41 (d, *J* = 8.1 Hz, 1H), 6.17 (d, *J* = 7.1 Hz, 1H), 5.97 (s, 2H), 5.40 (d, *J* = 7.4 Hz, 1H), 4.56 (dd, *J* = 7.1, 7.1 Hz, 1H), 4.29 (ddd, *J* = 8.2, 7.4, 5.6 Hz, 1H), 4.12 (dd, *J* = 8.1, 7.1 Hz, 1H), 3.69 (s, 3H), 3.57 (dd, *J* = 14.6, 5.6 Hz, 1H), 3.46 (dd, *J* = 14.6, 8.2 Hz, 1H), 2.54 (s, 6H), 2.08 – 1.96 (m, 1H), 1.51 – 1.34 (m, 15H), 1.25 (d, *J* = 7.1 Hz, 3H), 0.87 – 0.77 (m, 6H).

^{13}C NMR (101 MHz, $CDCl_3$): δ 172.8 (C_q), 171.3 (C_q), 169.6 (C_q), 155.4 (C_q), 151.8 (C_q), 149.6 (CH), 143.0 (C_q), 141.5 (C_q), 138.4 (CH), 138.1 (C_q), 138.0 (C_q), 134.2 (C_q), 134.1 (C_q), 131.3 (C_q), 130.7 (CH), 128.8 (C_q), 128.3 (CH), 127.0 (CH), 123.8 (CH), 122.6 (CH), 122.5 (CH), 121.2 (CH), 121.1 (CH), 118.9 (CH), 118.0 (CH), 112.9 (C_q), 110.7 (CH), 80.2 (C_q), 58.1 (CH), 55.6 (CH), 52.3 (CH_3), 48.0 (CH), 31.2 (CH), 28.2 (CH_3), 18.7 (CH_3), 17.8 (CH_3), 17.7 (CH_3), 14.6 (CH_3), 14.5 (CH_3). (One aromatic C_q and one aliphatic CH_2 is missing due to overlap, the overlap was verified by HSQC and HMBC analysis, showing that the peak at 155.4 correspond to two C_q carbons and the peak at 28.2 corresponds to two carbons, the CH_2 of the Trp side chain and the CH_3 of the Boc group).

^{19}F NMR (377 MHz, $CDCl_3$): δ –146.27 (q, $^1J_{B\text{-}F}$ = 31.8 Hz).

IR (ATR): 1670, 1542, 1509, 1456, 1195, 1157, 904, 723, 647 cm^{-1}.

MS (ESI) m/z (relative intensity): 936 (25) $[M+Na]^+$, 914 (100) $[M+H]^+$.

HR-MS (ESI) m/z calcd for $C_{51}H_{59}BF_2N_7O_6$ $[M+H]^+$: 914.4591, found: 914.4606.

Methyl *O*-benzyl-*N*-({*S*}-2-{[*tert*-butoxycarbonyl]amino}-3-{2-[(*E*)-4-(5,5-difluoro-1,3,7,9-tetramethyl-5*H*-4λ_4,5λ_4-dipyrrolo[1,2-*c*:2',1'-*f*][1,3,2]diazaborinin-10-yl)styryl]-1-[pyridin-2-yl]-1H-indol-3-yl}propanoyl)-*L*-threonyl-*L*-phenylalaninate (39)

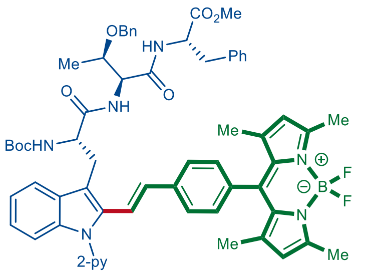

The general procedure **D** was followed using ethyl N_a-(*tert*-butoxycarbonyl)-1-(pyridin-2-yl)-*L*-tryptophyl-*L*-leucylglycinate (**4u**) (73.4 mg, 0.10 mmol), 10-(4-ethynylphenyl)-5,5-difluoro-1,3,7,9-tetramethyl-5*H*-4λ_4,5λ_4-dipyrrolo[1,2-*c*:2',1'-*f*][1,3,2]diazaborinine (**B2**) (69.6 mg, 0.20 mmol), $MnBr(CO)_5$ (5.5 mg, 20 mol %) and (1-Ad)CO_2H (7.2 mg, 40 mol %) in 1,4-dioxane (1.0 mL). Purification by column chromatography on silica gel (CH_2Cl_2/EtOAc: 10/1→10/2) yielded **39** (59.5 mg, 55%) as an orange solid.

M. p.: 147 – 149 ºC.

^{1}H NMR (400 MHz, $CDCl_3$): δ 8.77 (d, J = 5.0 Hz, 1H), 7.95 (ddd, J = 7.7, 7.7, 2.0 Hz, 1H), 7.62 – 7.53 (m, 4H), 7.44 (dd, J = 7.7, 5.0 Hz, 1H), 7.37 – 7.26 (m, 8H), 7.24 – 7.20 (m, 4H), 7.14 – 7.02 (m, 2H), 7.00 – 6.90 (m, 3H), 6.82 (d, J = 16.8 Hz, 1H), 6.62 (d, J = 5.4 Hz, 1H), 5.99 (s, 2H), 5.44 (d, J = 7.4 Hz, 1H), 4.72 – 4.35 (m, 4H), 4.25 – 4.09 (m, 1H), 4.03 (qd, J = 6.3, 3.4 Hz, 1H), 3.71 – 3.64 (m, 1H), 3.62 (s, 3H), 3.42 (dd, J = 14.3, 8.4 Hz, 1H), 2.96 (dd, J = 13.9, 5.8 Hz, 1H), 2.85 (dd, J = 13.9, 7.1 Hz, 1H), 2.58 (s, 6H), 1.47 – 1.38 (m, 15H), 0.92 (d, J = 6.3 Hz, 3H).

^{13}C NMR (101 MHz, $CDCl_3$): δ 171.4 (C_q), 171.4 (C_q), 168.0 (C_q), 155.4 (C_q), 155.2 (C_q), 151.8 (C_q), 149.6 (CH), 143.1 (C_q), 141.6 (C_q), 138.4 (CH), 138.2 (C_q), 138.1 (C_q), 137.8 (C_q), 135.7 (C_q), 134.1 (C_q), 134.0 (C_q), 131.4 (C_q), 130.6 (CH), 128.9 (CH), 128.8 (C_q),, 128.5 (CH), 128.4 (CH), 128.2 (CH), 127.8 (CH), 127.7 (CH), 127.1 (CH), 127.0 (CH), 123.8 (CH), 122.7 (CH), 122.5 (CH), 121.1 (CH), 121.1 (CH), 118.8 (CH), 118.0 (CH), 112.8 (C_q), 110.5 (CH), 80.1 (C_q), 73.8 (CH), 71.2 (CH_2), 55.5 (CH), 55.4 (CH), 53.6 (CH), 52.0 (CH_3), 37.7 (CH_2), 28.7 (CH_2), 28.3 (CH_3), 14.6 (CH_3), 14.5 (CH_3), 13.9 (CH_3).

^{19}F NMR (377 MHz, $CDCl_3$): δ –146.29 (q, $^1J_{B-F}$ = 31.8 Hz).

IR (ATR): 3343, 2973, 2928, 1744, 1667, 1541, 1507, 1454, 1192, 1154, 973, 733 cm^{-1}.

MS (ESI) m/z (relative intensity): 1104 (100) [M+Na]$^+$, 1083 (41) [M+H]$^+$.

HR-MS (ESI) m/z calcd for $C_{63}H_{67}BF_2N_7O_7$ $[M+H]^+$: 1082.5168, found: 1082.5147.

Benzyl (*S*)-3-({*S*}-2-acetamido-3-{2-[(*E*)-4-(5,5-difluoro-1,3,7,9-tetramethyl-5*H*-4λ_4,5λ_4-dipyrrolo[1,2-*c*:2',1'-*f*][1,3,2]diazaborinin-10-yl)styryl]-1-[pyridin-2-yl]-1*H*-indol-3-yl}propanamido)-4-{[(*S*)-1-methoxy-1-oxo-3-phenylpropan-2-yl]amino}-4-oxobutanoate (40)

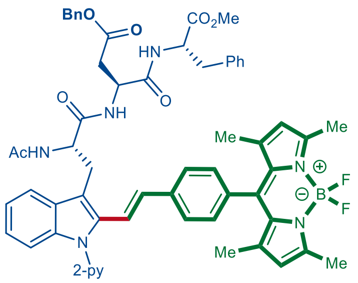

The general procedure **D** was followed using benzyl (*S*)-3-{[*S*]-2-acetamido-3-[1-(pyridin-2-yl)-1*H*-indol-3-yl]propanamido}-4-{[(*S*)-1-methoxy-1-oxo-3-phenylpropan-2-yl]amino}-4-oxobutanoate (**4v**) (68.9 mg, 0.10 mmol), 10-(4-ethynylphenyl)-5,5-difluoro-1,3,7,9-tetramethyl-5*H*-4λ_4,5λ_4-dipyrrolo[1,2-*c*:2',1'-*f*][1,3,2]diazaborinine (**B2**) (69.6 mg, 0.20 mmol), $MnBr(CO)_5$ (5.5 mg, 20 mol %) and (1-Ad)CO_2H (7.2 mg, 40 mol %) in 1,4-dioxane (1.0 mL). Purification by column chromatography on silica gel (CH_2Cl_2/Acetone: 10/1) yielded **40** (70.5 mg, 68%) as an orange solid.

M. p.: 151 – 153 ºC.

^{1}H NMR (400 MHz, $CDCl_3$): δ 8.75 (d, J = 4.8 Hz, 1H), 7.94 (ddd, J = 7.8, 7.8, 1.9 Hz, 1H), 7.59 (d, J = 5.0 Hz, 1H), 7.57 – 7.49 (m, 3H), 7.44 (dd, J = 7.4, 4.9 Hz, 1H), 7.37 – 7.20 (m, 12H), 7.17 – 7.05 (m, 4H), 6.85 (d, J = 7.4 Hz, 1H), 6.82 – 6.71 (m, 2H), 6.37 (d, J = 6.9 Hz, 1H), 5.99 (s, 2H), 5.03 (s, 2H), 4.77 (ddd, J = 8.4, 6.9, 5.4 Hz, 1H), 4.60 – 4.33 (m, 2H), 3.70 (dd, J = 14.4, 5.4 Hz, 1H), 3.61 (s, 3H), 3.39 (dd, J = 14.4, 8.4 Hz, 1H), 3.00 (dd, J = 13.9, 5.9 Hz, 1H), 2.93 (dd, J = 13.9, 7.2 Hz, 1H), 2.84 (dd, J = 17.4, 3.6 Hz, 1H), 2.57 (s, 6H), 2.42 (dd, J = 17.4, 8.1 Hz, 1H), 1.94 (s, 3H), 1.43 (s, 6H).

^{13}C NMR (101 MHz, $CDCl_3$): δ 171.8 (C_q), 171.2 (C_q), 170.6 (C_q), 170.2 (C_q), 169.1 (C_q), 155.4 (C_q), 151.7 (C_q), 149.7 (CH), 143.0 (C_q), 141.4 (C_q), 138.5 (CH), 138.1 (C_q), 137.8 (C_q), 135.8 (C_q), 135.2 (C_q), 134.3 (C_q), 134.1 (C_q), 131.3 (C_q), 130.8 (CH), 129.1 (CH), 128.8 (C_q), 128.6 (CH), 128.5 (CH), 128.4 (CH), 128.3 (CH), 128.2 (CH), 127.1 (CH), 127.0 (CH), 124.0 (CH), 122.7 (CH), 122.6 (CH), 121.4 (CH), 121.2 (CH), 118.5 (CH), 117.7 (CH), 112.6 (C_q), 110.7 (CH),

66.8 (CH_2), 54.3 (CH), 53.8 (CH), 52.2 (CH_3), 48.8 (CH), 37.4 (CH_2), 35.9 (CH_2), 27.7 (CH_2), 23.1 (CH_3), 14.6 (CH_3), 14.6 (CH_3).

^{19}F NMR (377 MHz, $CDCl_3$): δ –146.26 (q, $^{1}J_{B\text{-}F}$ = 31.4 Hz).

IR (ATR): 3286, 3032, 1732, 1632, 1540, 1507, 1453, 1304, 1191, 972, 711 cm^{-1}.

MS (ESI) m/z (relative intensity): 1060 (100) $[M+Na]^+$, 1038 (55) $[M+H]^+$.

HR-MS (ESI): m/z calcd for $C_{60}H_{59}BF_2N_7O_7$ $[M+H]^+$: 1038.4541, found: 1038.4527.

Methyl (*S*,*E*)-3-{2-[4-(5,5-difluoro-1,3,7,9-tetramethyl-5*H*-4λ_4,5λ_4-dipyrrolo[1,2-*c*:2',1'-*f*][1,3,2]diazaborinin-10-yl)styryl]-1-[pyridin-2-yl]-1*H*-indol-3-yl}-2-dodecanamidopropanoate (41)

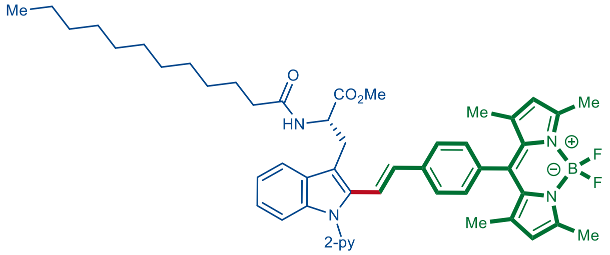

The general procedure **D** was followed using methyl *N*$_{\alpha}$-dodecanoyl-1-(pyridin-2-yl)-*L*-tryptophanate (**4w**) (47.8 mg, 0.10 mmol), 10-(4-ethynylphenyl)-5,5-difluoro-1,3,7,9-tetramethyl-5*H*-4λ_4,5λ_4-dipyrrolo[1,2-*c*:2',1'-*f*][1,3,2]diazaborinine (**B2**) (38.3 mg, 0.11 mmol), $MnBr(CO)_5$ (5.5 mg, 20 mol %) and $(1\text{-}Ad)CO_2H$ (7.2 mg, 40 mol %) in 1,4-dioxane (1.0 mL). Purification by column chromatography on silica gel (*n*-hexane/EtOAc: 8/2) yielded **41** (54.6 mg, 66%) as an orange solid.

M. p.: 114 – 117 ºC.

^{1}H NMR (600 MHz, $CDCl_3$): δ 8.72 (ddd, *J* = 4.9, 2.0, 0.9 Hz, 1H), 7.89 (ddd, *J* = 7.7, 7.7, 2.0 Hz, 1H), 7.55 (ddd, *J* = 7.7, 0.9, 0.9 Hz, 1H), 7.43 (d, *J* = 8.2 Hz, 2H), 7.40 – 7.35 (m, 3H), 7.21 – 7.13 (m, 5H), 6.46 (d, *J* = 16.8 Hz, 1H), 6.08 (d, *J* = 7.9 Hz, 1H), 5.95 (s, 2H), 5.00 (ddd, *J* = 7.9, 7.1, 5.1 Hz, 1H), 3.58 (dd, *J* = 14.6, 5.1 Hz, 1H), 3.55 – 3.52 (dd, *J* = 14.6, 7.1 Hz, 1H), 3.51 (s, 3H), 2.53 (s, 6H), 2.10 – 2.04 (m, 2H), 1.56 – 1.46 (m, 2H), 1.39 (s, 6H), 1.28 – 1.16 (m, 16H), 0.85 (t, *J* = 7.1 Hz, 3H).

^{13}C NMR (151 MHz, $CDCl_3$): δ 172.7 (C_q), 172.4 (C_q), 155.5 (C_q), 151.9 (C_q), 149.7 (CH), 142.9 (C_q), 141.3 (C_q), 138.4 (CH), 138.3 (C_q), 137.8 (C_q), 134.3 (C_q), 134.2 (C_q), 131.3 (C_q), 130.4 (CH), 129.0 (C_q), 128.4 (CH), 126.8 (CH), 124.0 (CH), 122.5 (CH), 122.3 (CH), 121.2 (CH), 121.2

(CH), 118.8 (CH), 117.8 (CH), 113.3 (C_q), 110.8 (CH), 52.7 (CH), 52.4 (CH_3), 36.5 (CH_2), 31.9 (CH_2), 29.6 (CH_2), 29.5 (CH_2), 29.4 (CH_2), 29.3 (CH_2), 29.3 (CH_2), 29.2 (CH_2), 27.8 (CH_2), 25.3 (CH_2), 22.6 (CH_2), 14.6 (CH_3), 14.5 (CH_3), 14.1 (CH_3).

^{19}F NMR (282 MHz, $CDCl_3$): δ –146.28 (q, $^{1}J_{B\text{-}F}$ = 32.2 Hz).

IR (ATR): 2921, 2851, 1740, 1653, 1540, 1508, 1466, 1191, 1154, 1081, 973, 741 cm^{-1}.

MS (ESI) m/z (relative intensity): 848 (41) $[M+Na]^+$, 826 (100) $[M+H]^+$.

HR-MS (ESI) m/z calcd for $C_{50}H_{59}BF_2N_5O_3$ $[M+H]^+$: 826.4682, found: 826.4660.

Methyl (*S*,*E*)-3-{2-[4-(5,5-difluoro-1,3,7,9-tetramethyl-5*H*-4λ_4,5λ_4-dipyrrolo[1,2-*c*:2',1'-*f*][1,3,2]diazaborinin-10-yl)styryl]-1-[pyridin-2-yl]-1*H*-indol-3-yl}-2-palmitamidopropanoate (42)

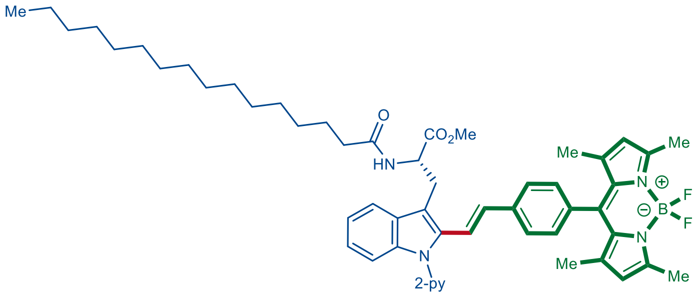

The general procedure **D** was followed using methyl N_α-palmitoyl-1-(pyridin-2-yl)-*L*-tryptophanate (**4x**) (53.3 mg, 0.10 mmol), 10-(4-ethynylphenyl)-5,5-difluoro-1,3,7,9-tetramethyl-5*H*-4λ_4,5λ_4-dipyrrolo[1,2-*c*:2',1'-*f*][1,3,2]diazaborinine (**B2**) (38.3 mg, 0.11 mmol), $MnBr(CO)_5$ (5.5 mg, 20 mol %) and $(1\text{-Ad})CO_2H$ (7.2 mg, 40 mol %) in 1,4-dioxane (1.0 mL). Purification by column chromatography on silica gel (*n*-hexane/EtOAc: 8/2) yielded **42** (59.9 mg, 68%) as an orange solid.

M. p.: 122 – 124 °C.

^{1}H NMR (400 MHz, $CDCl_3$): δ 8.77 (dd, *J* = 5.0, 1.8 Hz, 1H), 7.94 (ddd, *J* = 7.8, 7.8, 2.0 Hz, 1H), 7.59 (dd, *J* = 7.0, 1.7 Hz, 1H), 7.47 (d, *J* = 8.0 Hz, 2H), 7.45 – 7.39 (m, 3H), 7.27 – 7.16 (m, 5H), 6.50 (d, *J* = 16.8 Hz, 1H), 6.11 (d, *J* = 7.8 Hz, 1H), 5.99 (s, 2H), 5.05 (ddd, *J* = 7.8, 7.1, 5.2 Hz, 1H), 3.63 (dd, *J* = 14.6, 5.2 Hz, 1H), 3.59 (dd, *J* = 14.6, 7.1 Hz, 1H), 3.55 (s, 3H), 2.57 (s, 6H), 2.11 (t, *J* = 7.7 Hz, 2H), 1.63 – 1.49 (m, 2H), 1.43 (s, 6H), 1.31 – 1.17 (m, 24H), 0.90 (t, *J* = 6.7 Hz, 3H).

^{13}C NMR (101 MHz, $CDCl_3$): δ 172.7 (C_q), 172.4 (C_q), 155.5 (C_q), 152.0 (C_q), 149.7 (CH), 142.9 (C_q), 141.3 (C_q), 138.4 (CH), 138.3 (C_q), 137.8 (C_q), 134.3 (C_q), 134.3 (C_q), 131.3 (C_q), 130.4 (CH), 129.0 (C_q), 128.4 (CH), 126.8 (CH), 124.0 (CH), 122.5 (CH), 122.4 (CH), 121.3 (CH), 121.2 (CH), 118.8 (CH), 117.8 (CH), 113.3 (C_q), 110.8 (CH), 52.7 (CH), 52.4 (CH_3), 36.6 (CH_2), 31.9 (CH_2), 29.7 (CH_2), 29.7 (CH_2), 29.6 (CH_2), 29.6 (CH_2), 29.6 (CH_2), 29.4 (CH_2), 29.3 (CH_2), 29.3 (CH_2), 29.2 (CH_2), 27.8 (CH_2), 25.4 (CH_2), 22.7 (CH_2), 14.6 (CH_3), 14.6 (CH_3), 14.1 (CH_3). (One aliphatic CH_2 is missing due to overlap).
^{19}F NMR (377 MHz, $CDCl_3$): δ –146.29 (q, $^{1}J_{B\text{-}F}$ = 32.1 Hz).
IR (ATR): 2924, 1739, 1541, 1509, 1468, 1435, 1193, 982, 741 cm^{-1}.
MS (ESI) m/z (relative intensity): 904 (38) $[M+Na]^+$, 882 (100) $[M+H]^+$.
HR-MS (ESI) m/z calcd for $C_{54}H_{67}BF_2N_5O_3$ $[M+H]^+$: 882.5309, found: 882.5273.

Methyl (*S*,*E*)-3-{2-[4-(5,5-difluoro-1,3,7,9-tetramethyl-5*H*-4λ_4,5λ_4-dipyrrolo[1,2-*c*:2',1'-*f*][1,3,2]diazaborinin-10-yl)styryl)-1-[pyridin-2-yl]-1*H*-indol-3-yl}-2-stearamidopropanoate (43)

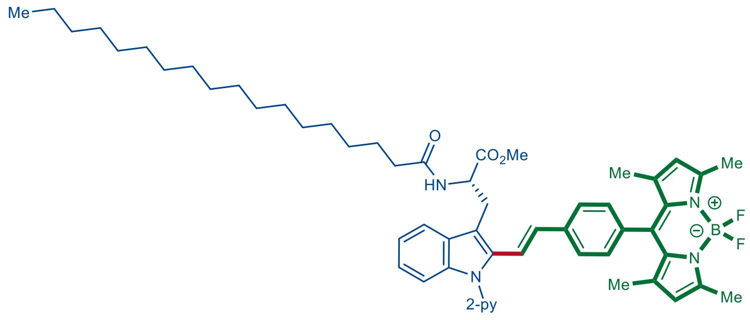

The general procedure **D** was followed using methyl 1-(pyridin-2-yl)-N_a-stearoyl-*L*-tryptophanate (**4y**) (56.1 mg, 0.10 mmol), 10-(4-ethynylphenyl)-5,5-difluoro-1,3,7,9-tetramethyl-5*H*-4λ_4,5λ_4-dipyrrolo[1,2-*c*:2',1'-*f*][1,3,2]diazaborinine (**B2**) (38.3 mg, 0.11 mmol), $MnBr(CO)_5$ (5.5 mg, 20 mol %) and (1-Ad)CO_2H (7.2 mg, 40 mol %) in 1,4-dioxane (1.0 mL). Purification by column chromatography on silica gel (*n*-hexane/EtOAc: 8/2) yielded **43** (60.0 mg, 66%) as an orange solid.
M. p.: 125 – 127 ºC.
^{1}H NMR (400 MHz, $CDCl_3$): δ 8.77 (dd, *J* = 5.0, 1.8 Hz, 1H), 7.94 (td, *J* = 7.7, 1.9 Hz, 1H), 7.62 – 7.55 (m, 1H), 7.50 – 7.45 (m, 2H), 7.45 – 7.38 (m, 3H), 7.26 – 7.17 (m, 5H), 6.50 (d, *J* = 16.7 Hz, 1H), 6.12 (d, *J* = 7.8 Hz, 1H), 5.99 (s, 2H), 5.05 (ddd, *J* = 7.2, 7.2 , 5.1 Hz, 1H), 3.63 (dd, *J* =

14.6, 5.2 Hz, 1H), 3.60 – 3.57 (m, 1H), 3.55 (s, 3H), 2.57 (s, 6H), 2.11 (dd, *J* = 8.5, 6.9 Hz, 2H), 1.55 (t, *J* = 7.3 Hz, 2H), 1.43 (s, 6H), 1.33 – 1.18 (m, 28H), 0.89 (t, *J* = 7.1 Hz, 3H).

^{13}C NMR (101 MHz, $CDCl_3$): δ 172.7 (C_q), 172.4 (C_q), 155.5 (C_q), 152.0 (C_q), 149.7 (CH), 142.9 (C_q), 141.3 (C_q), 138.4 (CH), 138.3 (C_q), 137.8 (C_q), 134.3 (C_q), 134.3 (C_q), 131.3 (C_q), 130.5 (CH), 129.0 (C_q), 128.4 (CH), 126.8 (CH), 124.0 (CH), 122.5 (CH), 122.4 (CH), 121.3 (CH), 121.2 (CH), 118.8 (CH), 117.8 (CH), 113.3 (C_q), 110.8 (CH), 52.7 (CH), 52.4 (CH_3), 38.7 (CH_2), 36.6 (CH_2), 36.4 (CH_2), 31.9 (CH_2), 29.7 (CH_2), 29.7 (CH_2), 29.6 (CH_2), 29.6 (CH_2), 29.6 (CH_2), 29.4 (CH_2), 29.3 (CH_2), 29.3 (CH_2), 29.2 (CH_2), 27.8 (CH_2), 25.4 (CH_2), 22.7 (CH_2), 14.6 (CH_3), 14.6 (CH_3), 14.1 (CH_3). (One aliphatic CH_2 is missing due to overlap).

^{19}F NMR (377 MHz, $CDCl_3$): δ –146.30 (q, $^{1}J_{B\text{-}F}$ = 32.2 Hz).

IR (ATR): 2921, 2850, 1740, 1712, 1541, 1508, 1456, 1435, 1192, 975, 742 cm^{-1}.

MS (ESI) m/z (relative intensity): 932 (100) $[M+Na]^+$, 910 (57) $[M+H]^+$.

HR-MS (ESI) m/z calcd for $C_{56}H_{71}BF_2N_5O_3$ $[M+H]^+$: 910.5622, found: 910.5592.

Methyl (*S*)-3-{2-[(*E*)-4-(5,5-difluoro-1,3,7,9-tetramethyl-5*H*-4λ_4,5λ_4-dipyrrolo[1,2-*c*:2',1'-*f*][1,3,2]diazaborinin-10-yl)styryl]-1-[pyridin-2-yl]-1*H*-indol-3-yl}-2-oleamidopropanoate (44)

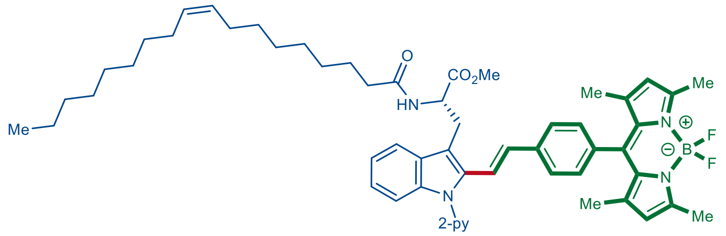

The general procedure **D** was followed using methyl N_a-oleoyl-1-(pyridin-2-yl)-*L*-tryptophanate (**4z**) (56.0 mg, 0.10 mmol), 10-(4-ethynylphenyl)-5,5-difluoro-1,3,7,9-tetramethyl-5*H*-4λ_4,5λ_4-dipyrrolo[1,2-*c*:2',1'-*f*][1,3,2]diazaborinine (**B2**) (38.3 mg, 0.11 mmol), $MnBr(CO)_5$ (5.5 mg, 20 mol %) and (1-Ad)CO_2H (7.2 mg, 40 mol %) in 1,4-dioxane (1.0 mL). Purification by column chromatography on silica gel (*n*-hexane/EtOAc: 8/2) yielded **44** (48.1 mg, 53%) as an orange solid.

M. p.: 116 – 118 °C.

^{1}H NMR (400 MHz, $CDCl_3$): *δ* 8.77 (d, *J* = 4.7 Hz, 1H), 7.94 (ddd, *J* = 7.9, 7.9 1.6 Hz, 1H), 7.60 (d, *J* = 7.4 Hz, 1H), 7.48 (d, *J* = 7.7 Hz, 2H), 7.46 – 7.38 (m, 3H), 7.28 – 7.16 (m, 5H), 6.50 (d, *J* = 16.7 Hz, 1H), 6.11 (d, *J* = 7.8 Hz, 1H), 6.00 (s, 2H), 5.43 – 5.28 (m, 2H), 5.05 (ddd, *J* = 7.8, 7.1,

5.2 Hz, 1H), 3.64 (dd, J = 14.7, 5.2 Hz, 1H), 3.59 (dd, J = 14.7, 7.1 Hz, 1H), 3.56 (s, 3H), 2.58 (s, 6H), 2.17 – 1.90 (m, 6H), 1.62 – 1.49 (m, 2H), 1.43 (s, 6H), 1.36 – 1.21 (m, 20H), 0.90 (t, J = 6.6 Hz, 3H).

^{13}C NMR (101 MHz, $CDCl_3$): δ 172.7 (C_q), 172.4 (C_q), 155.5 (C_q), 152.0 (C_q), 149.7 (CH), 142.9 (C_q), 141.3 (C_q), 138.4 (CH), 138.3 (C_q), 137.8 (C_q), 134.3 (C_q), 134.3 (C_q), 131.3 (C_q), 130.5 (CH), 130.0 (CH), 129.7 (CH), 129.0 (C_q), 128.4 (CH), 126.8 (CH), 124.0 (CH), 122.5 (CH), 122.4 (CH), 121.3 (CH), 121.2 (CH), 118.8 (CH), 117.8 (CH), 113.3 (C_q), 110.8 (CH), 52.7 (CH), 52.4 (CH_3), 36.6 (CH_2), 31.9 (CH_2), 29.7 (CH_2), 29.7 (CH_2), 29.5 (CH_2), 29.3 (CH_2), 29.3 (CH_2), 29.2 (CH_2), 29.2 (CH_2), 29.1 (CH_2), 27.8 (CH_2), 27.2 (CH_2), 27.2 (CH_2), 25.4 (CH_2), 22.7 (CH_2), 14.6 (CH_3), 14.6 (CH_3), 14.1 (CH_3).

^{19}F NMR (377 MHz, $CDCl_3$): δ –146.30 (q, $^{1}J_{B\text{-}F}$ = 32.2 Hz).

IR (ATR): 2922, 2850, 1736, 1666, 1650, 1541, 1509, 1305, 1193, 951, 705 cm^{-1}.

MS (ESI) m/z (relative intensity): 931 (100) $[M+Na]^+$, 909 (56) $[M+H]^+$.

HR-MS (ESI): m/z calcd for $C_{56}H_{68}BF_2N_5O_3Na$ $[M+Na]^+$: 930.5285, found: 930.5263.

Methyl (*S*,*E*)-2-[(*tert*-butoxycarbonyl)amino]-3-{2-[4-(5,5-difluoro-1,3,7,9-tetramethyl-5*H*-4λ_4,5λ_4-dipyrrolo[1,2-*c*:2',1'-*f*][1,3,2]diazaborinin-10-yl)styryl]-1*H*-indol-3-yl}propanoate (45)

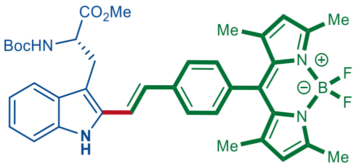

To a stirred solution of **3** (111.5 mg, 0.15 mmol) in CH_2Cl_2 (1.0 mL) methyl trifluoromethanesulfonate (73.8 mg, 0.45 mmol) was added at 0 °C. After 30 min, the mixture was allowed to warm up to 25 °C and stirred for 18 h. The crude mixture was concentrated under reduced pressure to afford an orange solid. In a sealed-tube, the crude product, $Pd(OH)_2/C$ (20 wt%, containing 50% water) (9.2 mg, 5.0 mol %), and ammonium formate (141.9 mg, 15.0 equiv) were dissolved in methanol (2.0 mL), and stirred at 60 ˚C for 16 hours. The mixture was filtered through a short plug of celite, concentrated under reduced pressure and purified by column chromatography on silica gel (Hexane/EtOAc: 8/2 → 7/3), yielding **45** (46.9 mg, 47%) as an orange solid.

M. p.: 168 – 170 ºC.

^{1}**H NMR** (400 MHz, $CDCl_3$): δ 8.36 (brs, 1H), 7.66 (d, J = 7.8 Hz, 2H), 7.52 (d, J = 7.4 Hz, 1H), 7.35 – 7.26 (m, 4H), 7.21 (dd, J = 7.4 Hz, 1H), 7.10 (dd, J = 7.4 Hz, 1H), 6.89 (d, J = 16.5 Hz, 1H), 6.00 (s, 2H), 5.18 (d, J = 8.2 Hz, 1H), 4.71 (ddd, J = 8.2, 6.9, 4.4 Hz, 1H), 3.57 (s, 3H), 3.46 (dd, J = 14.9, 4.4 Hz, 1H), 3.41 (dd, J = 14.9, 6.9 Hz, 1H), 2.57 (s, 6H), 1.46 (s, 6H), 1.41 (s, 9H).
^{13}**C NMR** (101 MHz, $CDCl_3$): δ 172.5 (C_q), 155.7 (C_q), 155.2 (C_q), 143.1 (C_q), 141.5 (C_q), 137.8 (C_q), 136.7 (C_q), 134.3 (C_q), 133.7 (C_q), 131.5 (C_q), 129.3 (C_q), 128.7 (CH), 127.1 (CH), 125.7 (CH), 123.8 (CH), 121.4 (CH), 120.2 (CH), 119.3 (CH), 118.0 (CH), 111.8 (C_q), 110.8 (CH), 80.0 (C_q), 54.6 (CH), 52.4 (CH_3), 28.5 (CH_3), 27.7 (CH_2), 14.8 (CH_3), 14.7 (CH_3).
^{19}**F NMR** (377 MHz, $CDCl_3$): δ –146.1 (q, $^{1}J_{B\text{-}F}$ = 32.2 Hz).
IR (ATR): 3401, 2970, 2952, 1707, 1541, 1506, 1191, 1153, 973, 710 cm^{-1}.
MS (ESI) m/z (relative intensity): 689 (100) $[M+Na]^+$, 667 (60) $[M+H]^+$, 647 (45) $[M-F]^+$.
HR-MS (ESI) m/z calcd for $C_{38}H_{42}BF_2N_4O_4$ $[M+H]^+$:667.3268, found: 667.3248.

Ethyl *O*-benzyl-*N*-({*S*}-2-{[*tert*-butoxycarbonyl]amino}-3-{2-[(*E*)-4-(5,5-difluoro-1,3,7,9-tetramethyl-5H-4λ_4,5λ_4-dipyrrolo[1,2-*c*:2',1'-*f*][1,3,2]diazaborinin-10-yl)styryl]-1-[pyridin-2-yl]-1*H*-indol-3-yl}propanoyl)-*L*-threonyl-*L*-isoleucylglycinate (47)

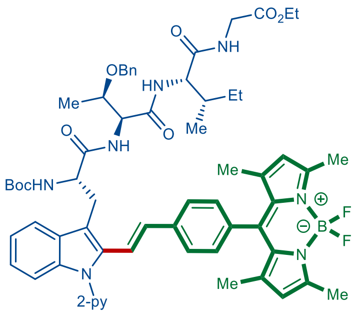

The general procedure **D** was followed using ethyl *O*-benzyl-*N*-(N_a-(*tert*-butoxycarbonyl)-1-(pyridin-2-yl)-*L*-tryptophyl)-*L*-threonyl-*L*-isoleucylglycinate (**4aa**) (77.1 mg, 0.10 mmol), 10-(4-ethynylphenyl)-5,5-difluoro-1,3,7,9-tetramethyl-5*H*-4λ_4,5λ_4-dipyrrolo[1,2-*c*:2',1'-*f*][1,3,2]diazaborinine (**B2**) (69.6 mg, 0.20 mmol), $MnBr(CO)_5$ (5.5 mg, 20 mol %) and (1-Ad)CO_2H (7.2 mg, 40 mol %) in 1,4-dioxane (1.0 mL). Purification by column chromatography on silica gel (CH_2Cl_2/EtOAc: 10/2→10/4) yielded **47** (97.3 mg, 87%) as an orange solid. The reaction was also performed at 60 ˚C and yielded **47** (69.6 mg, 62%).
M. p.: 152 – 154 ºC.

^{1}**H NMR** (400 MHz, $CDCl_3$): δ 8.78 (dd, J = 5.3, 2.0 Hz, 1H), 7.97 (ddd, J = 7.7, 7.7, 1.9 Hz, 1H), 7.73 – 7.61 (m, 1H), 7.52 – 7.43 (m, 4H), 7.40 (dd, J = 7.6, 1.4 Hz, 1H), 7.34 – 7.27 (m, 4H), 7.26 – 7.18 (m, 6H), 7.11 (brs, 1H), 7.04 (d, J = 8.3 Hz, 1H), 6.83 (s, 1H), 6.66 (d, J = 16.7 Hz, 1H), 5.99 (s, 2H), 5.29 (d, J = 4.7 Hz, 1H), 4.61 – 4.46 (m, 3H), 4.39 (d, J = 11.4 Hz, 1H), 4.24 – 4.13 (m, 4H), 4.07 (dd, J = 17.9, 5.8 Hz, 1H), 3.92 (dd, J = 17.9, 5.3 Hz, 1H), 3.66 (dd, J = 14.8, 5.8 Hz, 1H), 3.56 (dd, J = 14.8, 6.4 Hz, 1H), 2.57 (s, 6H), 1.84 – 1.70 (m, 1H), 1.62 – 1.46 (m, 2H), 1.43 (s, 6H), 1.32 – 1.21 (m, 12H), 0.92 – 0.80 (m, 9H).

^{13}**C NMR** (101 MHz, $CDCl_3$): δ 173.0 (C_q), 172.4 (C_q), 169.6 (C_q), 169.6 (C_q), 156.1 (C_q), 155.6 (C_q), 151.7 (C_q), 149.7 (CH), 143.0 (C_q), 141.2 (C_q), 138.6 (CH), 138.2 (C_q), 137.6 (C_q), 137.6 (C_q), 134.6 (C_q), 134.4 (C_q), 131.5 (CH), 131.3 (C_q), 128.7 (C_q), 128.5 (CH), 128.4 (CH), 127.9 (CH), 127.8 (CH), 127.0 (CH), 124.3 (CH), 122.8 (CH), 122.5 (CH), 121.7 (CH), 121.3 (CH), 118.8 (CH), 117.6 (CH), 112.5 (C_q), 110.9 (CH), 80.9 (C_q), 73.6 (CH), 71.7 (CH_2), 61.1 (CH_2), 58.3 (CH), 56.4 (CH), 51.9 (CH), 41.3 (CH_2), 40.0 (CH_2), 28.0 (CH_3), 27.1 (CH_2), 24.5 (CH), 23.1 (CH_3), 21.2 (CH_3), 15.9 (CH_3), 14.6 (CH_3), 14.6 (CH_3), 14.1 (CH_3).

^{19}**F NMR** (377 MHz, $CDCl_3$): δ –146.30 (q, $^{1}J_{B\text{-}F}$ = 31.8 Hz).

IR (ATR): 3311, 3272, 2969, 2924, 1749, 1669, 1640, 1541, 1507, 1191, 1154, 974, 739 cm^{-1}.

MS (ESI) m/z (relative intensity): 1142 (100) $[M+Na]^+$, 1120 (11) $[M+H]^+$.

HR-MS (ESI): m/z calcd for $C_{63}H_{74}BF_2N_8O_8$ $[M+H]^+$: 1119.5695, found: 1119.5684.

Ethyl ({*S*}-2-{[*tert*-butoxycarbonyl]amino}-3-{2-[(*E*)-4-(5,5-difluoro-1,3,7,9-tetramethyl-5*H*-4λ_4,5λ_4-dipyrrolo[1,2-*c*:2',1'-*f*][1,3,2]diazaborinin-10-yl)styryl]-1-[pyridin-2-yl]-1*H*-indol-3-yl}propanoyl)-*L*-leucyl-*L*-phenylalanylglycinate (48)

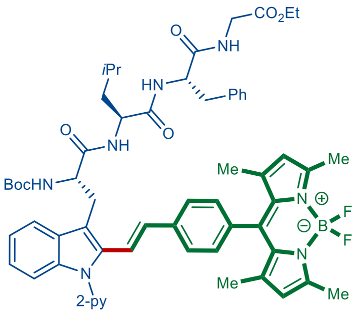

The general procedure **D** was followed using ethyl N_a-(*tert*-butoxycarbonyl)-1-(pyridin-2-yl)-*L*-tryptophyl-*L*-leucyl-*L*-phenylalanylglycinate (**4ab**) (72.7 mg, 0.10 mmol), 10-(4-ethynylphenyl)-5,5-difluoro-1,3,7,9-tetramethyl-5*H*-4λ_4,5λ_4-dipyrrolo[1,2-*c*:2',1'-*f*][1,3,2]diazaborinine (**b2**) (69.6

mg, 0.20 mmol), $MnBr(CO)_5$ (5.5 mg, 20 mol %) and (1-Ad)CO_2H (7.2 mg, 40 mol %) in 1,4-dioxane (1.0 mL). Purification by column chromatography on silica gel (CH_2Cl_2/EtOAc: 10/1→10/2) yielded **48** (81.7 mg, 76%) as an orange solid.

M. p.: 168 – 170 ºC.

^{1}H NMR (400 MHz, $CDCl_3$): δ 8.78 – 8.69 (m, 1H), 7.94 (ddd, J = 7.7, 7.7, 1.9 Hz, 1H), 7.74 – 7.61 (m, 1H), 7.50 (d, J = 8.0 Hz, 2H), 7.46 – 7.37 (m, 3H), 7.32 (d, J = 16.8 Hz, 1H), 7.27 – 7.10 (m, 9H), 6.75 (brs, 1H), 6.65 (d, J = 8.3 Hz, 1H), 6.60 (d, J = 16.8 Hz, 1H), 6.39 (d, J = 6.3 Hz, 1H), 5.99 (s, 2H), 5.28 (d, J = 6.1 Hz, 1H), 4.69 – 4.53 (m, 2H), 4.30 – 4.04 (m, 4H), 3.85 (dd, J = 18.0, 4.8 Hz, 1H), 3.70 – 3.47 (m, 2H), 3.25 (dd, J = 14.4, 5.9 Hz, 1H), 2.89 (dd, J = 14.4, 8.6 Hz, 1H), 2.57 (s, 6H), 1.43 (s, 6H), 1.41 – 1.34 (m, 11H), 1.26 (t, J = 7.1 Hz, 3H), 1.23 – 1.17 (m, 1H), 0.82 – 0.69 (m, 6H).

^{13}C NMR (101 MHz, $CDCl_3$): δ 172.4 (C_q), 171.3 (C_q), 170.9 (C_q), 169.5 (C_q), 155.9 (C_q), 155.5 (C_q), 151.8 (C_q), 149.8 (CH), 143.0 (C_q), 141.3 (C_q), 138.6 (CH), 138.2 (C_q), 137.7 (C_q), 137.1 (C_q), 134.5 (C_q), 131.3 (C_q), 131.3 (CH), 129.0 (CH), 128.7 (C_q), 128.5 (CH), 128.5 (CH), 127.1 (CH), 126.7 (CH), 124.2 (CH), 122.7 (CH), 122.3 (CH), 121.6 (CH), 121.2 (CH), 118.9 (CH), 117.8 (CH), 112.9 (C_q), 110.9 (CH), 80.7 (C_q), 61.4 (CH_2), 55.8 (CH), 54.1 (CH), 52.9 (CH), 41.3 (CH_2), 40.6 (CH_2), 37.2 (CH_2), 28.2 (CH_3), 27.3 (CH_2), 24.6 (CH), 22.7 (CH_3), 22.1 (CH_3), 14.7 (CH_3), 14.6 (CH_3), 14.1 (CH_3). (One aromatic C_q is missing due to overlap, the overlap was verified by HSQC and HMBC analysis, showing that the peak at 134.5 corresponds to two carbons).

^{19}F NMR (377 MHz, $CDCl_3$): δ –146.29 (q, $^1J_{B\text{-}F}$ = 31.9 Hz).

IR (ATR): 3305, 2959, 2930, 1749, 1667, 1641, 1541, 1508, 1468, 1191, 975, 739 cm^{-1}.

MS (ESI) m/z (relative intensity): 1098 (100) $[M+Na]^+$, 1076 (18) $[M+H]^+$.

HR-MS (ESI): m/z calcd for $C_{61}H_{70}BF_2N_8O_7$ $[M+H]^+$: 1075.5433, found: 1075.5410.

Ethyl ({*S*}-2-{[*tert*-butoxycarbonyl]amino}-3-{2-[(*E*)-4-(5,5-difluoro-1,3,7,9-tetramethyl-5*H*-4*λ*4,5*λ*4-dipyrrolo[1,2-*c*:2',1'-*f*][1,3,2]diazaborinin-10-yl)styryl]-1-[pyridin-2-yl]-1*H*-indol-3-yl}propanoyl)-*L*-alanyl-*L*-leucylglycinate (49)

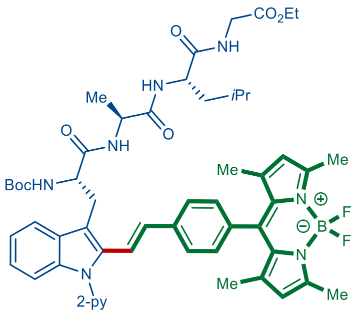

The general procedure **D** was followed using ethyl N_a-(*tert*-butoxycarbonyl)-1-(pyridin-2-yl)-*L*-tryptophyl-*L*-alanyl-*L*-leucylglycinate (**4ac**) (65.0 mg, 0.10 mmol), 10-(4-ethynylphenyl)-5,5-difluoro-1,3,7,9-tetramethyl-5*H*-4λ_4,5λ_4-dipyrrolo[1,2-*c*:2',1'-*f*][1,3,2]diazaborinine (**B2**) (69.6 mg, 0.20 mmol), $MnBr(CO)_5$ (5.5 mg, 20 mol %) and (1-Ad)CO_2H (7.2 mg, 40 mol %) in 1,4-dioxane (1.0 mL). Purification by column chromatography on silica gel (CH_2Cl_2/EtOAc: 6/4) yielded **49** (73.9 mg, 74%) as an orange solid.

M. p.: 166 – 169 ºC.

^{1}H NMR (400 MHz, $CDCl_3$): δ 8.78 (d, J = 4.8 Hz, 1H), 7.95 (dd, J = 6.8, 6.8 Hz, 1H), 7.65 (d, J = 7.4 Hz, 1H), 7.53 – 7.37 (m, 5H), 7.31 – 7.27 (m, 1H), 7.27 – 7.19 (m, 4H), 7.03 (brs, 1H), 6.84 (d, J = 8.4 Hz, 1H), 6.60 (d, J = 16.7 Hz, 1H), 6.44 (brs, 1H), 5.99 (s, 2H), 5.29 (d, J = 5.0 Hz, 1H), 4.55 (ddd, J = 5.8, 5.5, 4.8 Hz, 1H), 4.42 (ddd, J = 7.6, 7.6, 6.4 Hz, 1H), 4.25 – 4.14 (m, 3H), 4.08 (dd, J = 18.1, 5.5 Hz, 1H), 3.94 (dd, J = 18.1, 4.8 Hz, 1H), 3.66 – 3.49 (m, 2H), 2.56 (s, 6H), 1.86 – 1.75 (m, 1H), 1.67 – 1.50 (m, 2H), 1.43 (s, 6H), 1.37 (s, 9H), 1.26 (t, J = 7.1 Hz, 3H), 1.19 (d, J = 6.9 Hz, 3H), 0.90 (d, J = 6.3 Hz, 3H), 0.87 (d, J = 6.3 Hz, 3H).

^{13}C NMR (101 MHz, $CDCl_3$): δ 172.4 (C_q), 172.2 (C_q), 171.7 (C_q), 169.6 (C_q), 155.8 (C_q), 155.5 (C_q), 151.7 (C_q), 149.7 (CH), 142.9 (C_q), 141.2 (C_q), 138.6 (CH), 138.1 (C_q), 137.5 (C_q), 134.5 (C_q), 134.4 (C_q), 131.6 (CH), 131.3 (C_q), 128.7 (C_q), 128.4 (CH), 127.0 (CH), 124.3 (CH), 122.7 (CH), 122.4 (CH), 121.6 (CH), 121.2 (CH), 118.7 (CH), 117.5 (CH), 112.7 (C_q), 110.9 (CH), 80.8 (C_q), 61.2 (CH_2), 56.1 (CH), 51.7 (CH), 50.0 (CH), 41.3 (CH_2), 40.2 (CH_2), 28.1 (CH_3), 27.1 (CH_2), 24.8 (CH), 22.9 (CH_3), 21.5 (CH_3), 17.6 (CH_3), 14.6 (CH_3), 14.5 (CH_3), 14.1 (CH_3).

^{19}F NMR (377 MHz, $CDCl_3$): δ –146.27 (q, $^1J_{B\text{-}F}$ = 31.7 Hz).

IR (ATR): 3275, 2966, 1700, 1633, 1541, 1507, 1453, 1191, 975, 709 cm^{-1}.

MS (ESI) m/z (relative intensity): 1021 (100) $[M+Na]^+$, 999 (25) $[M+H]^+$.

HR-MS (ESI): m/z calcd for $C_{55}H_{66}BF_2N_8O_7$ $[M+H]^+$: 999.5119, found: 999.5100.

Methyl (6*S*,9*S*,15*S*)-9-benzyl-15-({2-[(*E*)-4-(5,5-difluoro-1,3,7,9-tetramethyl-5*H*-4λ_4,5λ_4-dipyrrolo[1,2-*c*:2',1'-*f*][1,3,2]diazaborinin-10-yl)styryl]-1-[pyridin-2-yl]-1*H*-indol-3-yl}methyl)-2,2,6-trimethyl-4,7,10,13-tetraoxo-3-oxa-5,8,11,14-tetraazahexadecan-16-oate (50)

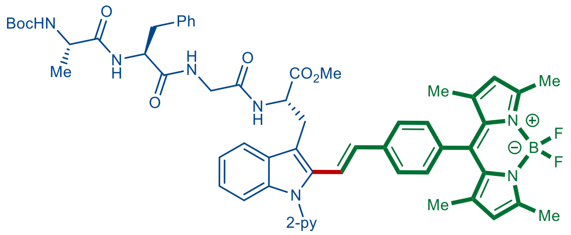

The general procedure **D** was followed using methyl N_a-(*tert*-butoxycarbonyl)-*L*-alanyl-*L*-phenylalanylglycyl-1-(pyridin-2-yl)-*L*-tryptophanate (**4ad**) (67.1 mg, 0.10 mmol), 10-(4-ethynylphenyl)-5,5-difluoro-1,3,7,9-tetramethyl-5*H*-4λ_4,5λ_4-dipyrrolo[1,2-*c*:2',1'-*f*][1,3,2]diazaborinine (**B2**) (69.6 mg, 0.20 mmol), $MnBr(CO)_5$ (5.5 mg, 20 mol %) and (1-Ad)CO_2H (7.2 mg, 40 mol %) in 1,4-dioxane (1.0 mL). Purification by column chromatography on silica gel (CH_2Cl_2/EtOAc: 1/1) yielded **50** (57.1 mg, 56%) as an orange solid.

M. p.: 167 – 169 °C.

^{1}H NMR (400 MHz, $CDCl_3$): δ 8.66 (dd, J = 5.0, 2.0 Hz, 1H), 7.95 (ddd, J = 7.7, 7.7, 2.0 Hz, 1H), 7.62 – 7.56 (m, 1H), 7.51 (d, J = 8.2 Hz, 2H), 7.46 (d, J = 8.0 Hz, 1H), 7.43 – 7.36 (m, 2H), 7.28 – 7.18 (m, 8H), 7.16 – 7.10 (m, 2H), 7.09 – 6.97 (m, 2H), 6.91 (d, J = 7.5 Hz, 1H), 6.55 (d, J = 16.7 Hz, 1H), 5.99 (s, 2H), 5.12 – 4.88 (m, 2H), 4.61 (ddd, J = 7.0, 7.0, 7.0 Hz, 1H), 4.03 (dq, J = 7.5, 6.9 Hz, 1H), 3.96 – 3.77 (m, 2H), 3.58 (d, J = 6.6 Hz, 2H), 3.55 (s, 3H), 3.09 (d, J = 6.7 Hz, 2H), 2.57 (s, 6H), 1.43 (s, 6H), 1.39 (s, 9H), 1.19 (d, J = 6.9 Hz, 3H).

^{13}C NMR (101 MHz, $CDCl_3$): δ 173.0 (C_q), 172.0 (C_q), 171.2 (C_q), 168.5 (C_q), 155.7 (C_q), 155.4 (C_q), 151.8 (C_q), 149.7 (CH), 143.0 (C_q), 141.4 (C_q), 138.6 (CH), 138.1 (C_q), 137.8 (C_q), 136.4 (C_q), 134.3 (C_q), 134.3 (C_q), 131.3 (C_q), 130.7 (CH), 129.1 (CH), 128.9 (C_q), 128.7 (CH), 128.4 (CH), 127.0 (CH), 126.9 (CH), 124.0 (CH), 122.6 (CH), 122.3 (CH), 121.3 (CH), 121.2 (CH), 118.9 (CH), 117.9 (CH), 113.2 (C_q), 110.7 (CH), 80.5 (C_q), 54.3 (CH), 52.9 (CH), 52.5 (CH_3), 50.7 (CH), 43.2 (CH_2), 37.1 (CH_2), 28.2 (CH_3), 27.9 (CH_2), 17.8 (CH_3), 14.6 (CH_3), 14.6 (CH_3).

^{19}**F NMR** (377 MHz, $CDCl_3$): δ –146.26 (q, $^{1}J_{B\text{-}F}$ = 31.8 Hz).

IR (ATR): 3297, 3279, 2952, 1738, 1667, 1639, 1540, 1507, 1436, 1192, 975 cm^{-1}.

MS (ESI) m/z (relative intensity): 1041 (100) $[M+Na]^+$, 1019 (18) $[M+H]^+$.

HR-MS (ESI): m/z calcd for $C_{57}H_{62}BF_2N_8O_7$ $[M+H]^+$: 1019.4806, found: 1019.4789.

Methyl *N*-[*N*$_a$-(*tert*-butoxycarbonyl)-1-(pyridin-2-yl)-*L*-tryptophyl]-*S*-{[(*R*)-2-({*S*}-2-{[*tert*-butoxycarbonyl]amino}-3-{2-[(*E*)-4-(5,5-difluoro-1,3,7,9-tetramethyl-5*H*-4λ_4,5λ_4-dipyrrolo[1,2-*c*:2',1'-*f*][1,3,2]diazaborinin-10-yl)styryl]-1-[pyridin-2-yl]-1*H*-indol-3-yl}propanamido)-3-methoxy-3-oxopropyl]thio}-*L*-cysteinate (51)

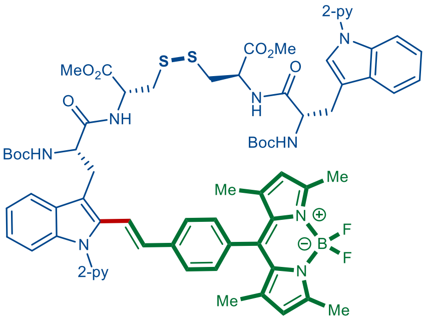

The general procedure **D** was followed using methyl (6*S*,9*R*,14*R*)-14-{[*S*]-2-[(*tert*-butoxycarbonyl)amino]-3-[1-(pyridin-2-yl)-1*H*-indol-3-yl]propanamido}-9-(methoxycarbonyl)-2,2-dimethyl-4,7-dioxo-6-{[1-(pyridin-2-yl)-1*H*-indol-3-yl]methyl}-3-oxa-11,12-dithia-5,8-diazapentadecan-15-oate (**4ae**) (99.5 mg, 0.10 mmol), 10-(4-ethynylphenyl)-5,5-difluoro-1,3,7,9-tetramethyl-5*H*-4λ_4,5λ_4-dipyrrolo[1,2-*c*:2',1'-*f*][1,3,2]diazaborinine (**B2**) (139.2 mg, 0.40 mmol), $MnBr(CO)_5$ (5.5 mg, 20 mol %) and (1-Ad)CO_2H (7.2 mg, 40 mol %) in 1,4-dioxane (1.0 mL). Purification by column chromatography on silica gel (CH_2Cl_2/EtOAc: 6/4) yielded **51** (67.2 mg, 50%) as an orange solid.

M. p.: 150 – 152 ºC.

^{1}**H NMR** (400 MHz, $CDCl_3$): δ 8.73 (d, *J* = 5.2 Hz, 1H), 8.50 (d, *J* = 5.3 Hz, 1H), 8.24 (d, *J* = 8.3 Hz, 1H), 7.88 (ddd, *J* = 7.7, 7.7, 2.0 Hz, 1H), 7.75 (dd, *J* = 7.9, 7.9 Hz, 1H), 7.69 – 7.60 (m, 3H), 7.55 (d, *J* = 7.7 Hz, 2H), 7.47 – 7.33 (m, 4H), 7.33 – 7.29 (m, 1H), 7.28 – 7.25 (m, 1H), 7.25 – 6.99 (m, 7H), 6.80 (d, *J* = 7.1 Hz, 1H), 6.69 (d, *J* = 16.8 Hz, 1H), 5.99 (s, 2H), 5.77 (d, *J* = 8.0 Hz, 1H), 5.48 (brs, 1H), 4.76 (ddd, *J* = 8.0, 6.0, 5.2 Hz, 1H), 4.73 – 4.56 (m, 3H), 3.68 – 3.46 (m, 8H), 3.40 – 3.21 (m, 2H), 3.08 (dd, *J* = 14.3, 5.2 Hz, 1H), 3.00 – 2.86 (m, 3H), 2.57 (s, 6H), 1.50 – 1.32 (m, 24H).

^{13}C NMR (101 MHz, $CDCl_3$): δ 171.7 (C_q), 171.6 (C_q), 170.2 (C_q), 169.8 (C_q), 155.6 (C_q), 155.5 (C_q), 155.4 (C_q), 152.3 (C_q), 151.9 (C_q), 149.6 (CH), 148.7 (CH), 143.1 (C_q), 141.6 (C_q), 138.4 (CH), 138.3 (CH), 138.1 (C_q), 138.1 (C_q), 135.4 (C_q), 134.2 (C_q), 134.1 (C_q), 131.4 (C_q), 130.6 (CH), 130.1 (C_q), 129.1 (C_q), 128.3 (CH), 127.1 (CH), 124.9 (CH), 123.8 (CH), 123.4 (CH), 122.4 (CH), 122.4 (CH), 121.3 (CH), 121.2 (CH), 121.2 (CH), 119.7 (CH), 119.1 (CH), 119.0 (CH), 118.1 (CH), 114.2 (CH), 113.6 (C_q), 113.6 (C_q), 113.4 (CH), 110.7 (CH), 80.1 (C_q), 80.0 (C_q), 55.5 (CH), 54.8 (CH), 52.7 (CH_3), 52.6 (CH_3), 52.0 (CH), 51.8 (CH), 40.5 (CH_2), 40.3 (CH_2), 29.0 (CH_2), 28.3 (CH_3), 28.3 (CH_3), 28.1 (CH_2), 14.7 (CH_3), 14.6 (CH_3).
^{19}F NMR (377 MHz, $CDCl_3$): δ – 146.28 (q, $^{1}J_{B\text{-}F}$ = 31.2 Hz).
IR (ATR): 1746, 1680, 1507, 1470, 1435, 1192, 1154, 974, 740 cm^{-1}.
MS (ESI) m/z (relative intensity): 1366 (75) $[M+Na]^+$, 1344 (100) $[M+H]^+$.
HR-MS (ESI): m/z calcd for $C_{71}H_{78}BF_2N_{10}O_{10}S$ $[M+H]^+$: 1343.5411, found: 1343.5395.

Methyl ({*S*}-2-{[*tert*-butoxycarbonyl)amino}-3-{2-[(*E*)-4-(5,5-difluoro-1,3,7,9-tetramethyl-5*H*-4λ_4,5λ_4-dipyrrolo[1,2-*c*:2',1'-*f*][1,3,2]diazaborinin-10-yl)styryl]-1-[pyridin-2-yl]-1*H*-indol-3-yl}propanoyl)-*L*-phenylalanyl-*L*-isoleucylglycyl-*L*-serinate (52)

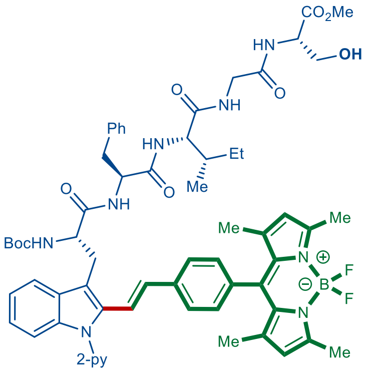

The general procedure **D** was followed using methyl N_a-(*tert*-butoxycarbonyl)-1-(pyridin-2-yl)-*L*-tryptophyl-*L*-phenylalanyl-*L*-isoleucylglycyl-*L*-serinate (**4af**) (80.0 mg, 0.10 mmol), 10-(4-ethynylphenyl)-5,5-difluoro-1,3,7,9-tetramethyl-5*H*-4λ_4,5λ_4-dipyrrolo[1,2-*c*:2',1'-*f*][1,3,2]diazaborinine (**B2**) (69.7 mg, 0.20 mmol), $MnBr(CO)_5$ (5.5 mg, 20 mol %) and (1-Ad)CO_2H (7.2 mg, 40 mol %) in 1,4-dioxane (1.0 mL). Purification by column chromatography on silica gel (CH_2Cl_2/Acetone: 8/2→6/4) yielded **52** (79.0 mg, 69%) as an orange solid.
M. p.: 97 °C (decomposition).

^{1}H NMR (400 MHz, DMSO-d_6): δ 8.73 (dd, J = 5.0, 1.7 Hz, 1H), 8.24 – 8.14 (m, 3H), 8.09 (dd, J = 7.9, 7.9 Hz, 1H), 7.92 (d, J = 8.3 Hz, 1H), 7.79 – 7.71 (m, 1H), 7.62 – 7.53 (m, 3H), 7.48 (d, J = 8.0 Hz, 1H), 7.40 – 7.31 (m, 3H), 7.31 – 7.09 (m, 9H), 6.47 (d, J = 16.7 Hz, 1H), 6.18 (s, 2H), 4.79 (td, J = 8.3, 4.8 Hz, 1H), 4.50 – 4.35 (m, 2H), 4.30 (td, J = 9.3, 4.0 Hz, 1H), 4.22 (t, J = 7.9 Hz, 1H), 3.91 – 3.67 (m, 3H), 3.66 – 3.55 (m, 4H), 3.22 – 3.00 (m, 3H), 2.87 (dd, J = 13.8, 8.7 Hz, 1H), 2.45 (s, 6H), 1.81 – 1.67 (m, 1H), 1.50 – 1.43 (m, 1H), 1.40 (s, 6H), 1.18 (s, 9H), 1.12 – 1.02 (m, 1H), 0.85 (d, J = 6.6 Hz, 3H), 0.79 (t, J = 7.4 Hz, 3H).

^{13}C NMR (101 MHz, DMSO-d_6): δ 171.7 (C_q), 171.4 (C_q), 171.4 (C_q), 171.1 (C_q), 169.3 (C_q), 155.4 (C_q), 155.3 (C_q), 151.7 (C_q), 150.1 (CH), 143.1 (C_q), 142.2 (C_q), 139.7 (CH), 138.3 (C_q), 138.2 (C_q), 137.9 (C_q), 134.1 (C_q), 133.6 (C_q), 131.1 (C_q), 130.0 (CH), 129.9 (CH), 129.0 (C_q), 128.7 (CH), 128.3 (CH), 127.3 (CH), 126.6 (CH), 124.0 (CH), 123.6 (CH), 122.8 (CH), 121.8 (CH), 121.1 (CH), 119.9 (CH), 118.8 (CH), 115.7 (C_q), 110.8 (CH), 78.7 (C_q), 61.7 (CH_2), 57.5 (CH), 56.6 (CH), 55.1 (CH), 53.7 (CH), 52.3 (CH_3), 42.1 (CH_2), 38.3 (CH_2), 37.1 (CH), 28.5 (CH_2), 28.4 (CH_3), 24.8 (CH_2), 15.7 (CH_3), 14.7 (CH_3), 14.7 (CH_3), 11.5 (CH_3).

^{19}F NMR (377 MHz, DMSO-d_6): δ –143.7 (q, $^1J_{B\text{-}F}$ = 31.3 Hz).

IR (ATR): 3280, 2962, 2926, 1745, 1656, 1542, 1509, 1195, 1025, 1007, 983 cm^{-1}.

MS (ESI) m/z (relative intensity): 1171 (100) $[M+Na]^+$, 1149 (10) $[M+H]^+$.

HR-MS (ESI) m/z calcd for $C_{63}H_{72}BF_2N_9O_9Na$ $[M+Na]^+$:1170.5417, found: 1170.5374.

Methyl ({*S*}-2-{[*tert*-butoxycarbonyl]amino}-3-{2-[(*E*)-4-(5,5-difluoro-1,3,7,9-tetramethyl-5*H*-4λ_4,5λ_4-dipyrrolo[1,2-*c*:2',1'-*f*][1,3,2]diazaborinin-10-yl)styryl]-1-[pyridin-2-yl]-1*H*-indol-3-yl}propanoyl)-*L*-alanyl-*L*-isoleucylglycyl-*L*-tryptophanate (53)

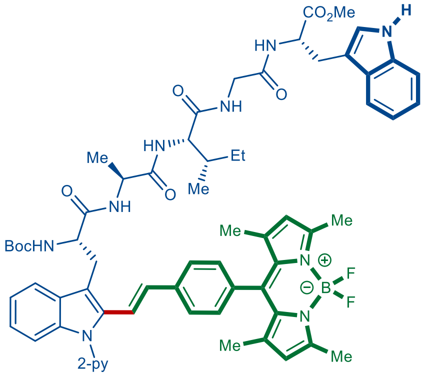

The general procedure **D** was followed using methyl N_a-(*tert*-butoxycarbonyl)-1-(pyridin-2-yl)-*L*-tryptophyl-*L*-alanyl-*L*-isoleucylglycyl-*L*-tryptophanate (**4ag**) (82.6 mg, 0.10 mmol), 10-(4-

ethynylphenyl)-5,5-difluoro-1,3,7,9-tetramethyl-5*H*-4λ_4,5λ_4-dipyrrolo[1,2-*c*:2',1'-*f*][1,3,2]diazaborinine (**B2**) (69.2 mg, 0.40 mmol), $MnBr(CO)_5$ (5.5 mg, 20 mol %) and (1-Ad)CO_2H (7.2 mg, 40 mol %) in 1,4-dioxane (1.0 mL). Purification by column chromatography on silica gel (CH_2Cl_2/EtOAc: 6/4) yielded **53** (66.7 mg, 57%) as an orange solid.

M. p.:179 – 181 ºC.

^{1}H NMR (400 MHz, CD_3OD): δ 8.69 (d, *J* = 5.5 Hz, 1H), 8.06 (dd, *J* = 8.0, 8.0 Hz, 1H), 7.73 (d, *J* = 7.4 Hz, 1H), 7.58 – 7.41 (m, 5H), 7.39 – 7.26 (m, 2H), 7.25 – 7.11 (m, 5H), 7.09 – 6.92 (m, 3H), 6.39 (d, *J* = 16.7 Hz, 1H), 6.03 (s, 2H), 4.71 (dd, *J* = 6.5, 6.5 Hz, 1H), 4.58 (dd, *J* = 6.9, 6.9 Hz, 1H), 4.32 (d, *J* = 7.8 Hz, 1H), 4.11 (d, *J* = 7.1 Hz, 1H), 3.93 (d, *J* = 16.6 Hz, 1H), 3.79 (d, *J* = 16.9 Hz, 1H), 3.65 – 3.46 (m, 4H), 3.44 – 3.35 (m, 1H), 3.29 – 3.10 (m, 2H), 2.49 (s, 6H), 1.87 – 1.75 (m, 1H), 1.60 – 1.46 (m, 1H), 1.40 (s, 6H), 1.31 (s, 9H), 1.24 – 1.08 (m, 4H), 0.90 (d, *J* = 7.8 Hz, 3H), 0.86 (d, *J* = 7.3 Hz, 3H).

^{13}C NMR (101 MHz, CD_3OD): δ 174.7 (C_q), 174.1 (C_q), 174.0 (C_q), 173.6 (C_q), 171.2 (C_q), 157.4 (C_q), 156.7 (C_q), 153.4 (C_q), 150.5 (CH), 144.4 (C_q), 143.2 (C_q), 140.9 (CH), 140.2 (C_q), 139.5 (C_q), 137.9 (C_q), 135.8 (C_q), 135.4 (C_q), 132.5 (C_q), 131.7 (CH), 130.3 (C_q), 129.7 (CH), 128.7 (C_q), 128.2 (CH), 125.1 (CH), 124.5 (CH), 124.5 (CH), 122.4 (CH), 122.3 (CH), 122.2 (CH), 120.4 (CH), 119.9 (CH), 119.2 (CH), 119.1 (CH), 115.8 (C_q), 112.3 (CH), 111.4 (CH), 111.4 (CH), 110.5 (C_q), 80.8 (C_q), 59.8 (CH), 57.0 (CH), 54.8 (CH), 52.7 (CH_3), 50.5 (CH), 43.3 (CH_2), 37.7 (CH), 28.8 (CH_2), 28.6 (CH_3), 28.4 (CH_2), 26.2 (CH_2), 17.9 (CH_3), 15.9 (CH_3), 14.9 (CH_3), 14.6 (CH_3), 11.6 (CH_3).

^{19}F NMR (377 MHz, CD_3OD): δ –146.95 (q, $^1J_{B\text{-}F}$ = 31.2 Hz).

IR (ATR): 3292, 2962, 2929, 1742, 1712, 1628, 1541, 1454, 1409, 1192, 1154, 973, 739 cm^{-1}.

MS (ESI) m/z (relative intensity): 1194 (100) $[M+Na]^+$, 1172 (8) $[M+H]^+$.

HR-MS (ESI): m/z calcd for $C_{65}H_{74}BF_2N_{10}O_8$ $[M+H]^+$: 1171.5757, found: 1171.5739.

(3*S*,8$_a$*S*)-3-({2-[(*E*)-4-(5,5-difluoro-1,3,7,9-tetramethyl-5*H*-4λ_4,5λ_4-dipyrrolo[1,2-*c*:2',1'-*f*][1,3,2]diazaborinin-10-yl)styryl]-1-]pyridin-2-yl]-1*H*-indol-3-yl}methyl)hexahydropyrrolo[1,2-*a*]pyrazine-1,4-dione (54)

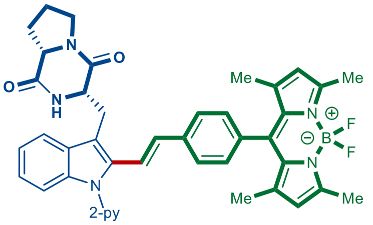

The general procedure **D** was followed using (3*S*,8*aS*)-3-{[1-(pyridin-2-yl)-1*H*-indol-3-yl]methyl}hexahydropyrrolo[1,2-*a*]pyrazine-1,4-dione (**4ah**) (36.1 mg, 0.10 mmol), 10-(4-ethynylphenyl)-5,5-difluoro-1,3,7,9-tetramethyl-5*H*-4λ_4,5λ_4-dipyrrolo[1,2-*c*:2',1'-*f*][1,3,2]diazaborinine (**B2**) (38.3 mg, 0.11 mmol), $MnBr(CO)_5$ (5.5 mg, 20 mol %) and (1-Ad)CO_2H (7.2 mg, 40 mol %) in 1,4-dioxane (1.0 mL). Purification by column chromatography on silica gel (CH_2Cl_2/EtOAc: 10/1) yielded **54** (52.4 mg, 74%) as an orange solid.

M. p.: 178 ºC (decomposition).

^{1}H NMR (400 MHz, $CDCl_3$): δ 8.74 (dd, *J* = 5.1, 1.8 Hz, 1H), 7.95 (ddd, *J* = 7.7, 7.7, 2.0 Hz, 1H), 7.68 – 7.59 (m, 1H), 7.47 (d, *J* = 8.0 Hz, 1H), 7.44 – 7.38 (m, 4H), 7.28 (dd, *J* = 7.2, 1.3 Hz, 1H), 7.25 (dd, *J* = 1.7, 1.7 Hz, 1H), 7.23 – 7.16 (m, 3H), 6.58 (d, *J* = 16.7 Hz, 1H), 5.98 (s, 2H), 5.72 (s, 1H), 4.56 (dd, *J* = 11.5, 2.1 Hz, 1H), 4.10 (ddd, *J* = 7.4, 7.4, 3.6 Hz, 1H), 4.01 (dd, *J* = 15.2, 3.6 Hz, 1H), 3.78 – 3.58 (m, 2H), 3.34 (dd, *J* = 15.2, 11.5 Hz, 1H), 2.55 (s, 6H), 2.35 (ddd, *J* = 9.9, 8.4, 5.8 Hz, 1H), 2.16 – 2.02 (m, 2H), 1.99 – 1.85 (m, 1H), 1.42 (s, 6H).

^{13}C NMR (101 MHz, $CDCl_3$): δ 169.4 (C_q), 165.5 (C_q), 155.5 (C_q), 151.4 (C_q), 149.8 (CH), 143.0 (C_q), 141.2 (C_q), 138.5 (CH), 138.1 (C_q), 137.4 (C_q), 134.6 (C_q), 134.6 (C_q), 131.9 (CH), 131.3 (C_q), 128.4 (CH), 128.2 (C_q), 127.1 (CH), 124.4 (CH), 122.7 (CH), 122.1 (CH), 121.7 (CH), 121.2 (CH), 118.5 (CH), 117.7 (CH), 111.8 (C_q), 111.1 (CH), 59.3 (CH), 54.9 (CH), 45.5 (CH_2), 28.3 (CH_2), 26.2 (CH_2), 22.7 (CH_2), 14.7 (CH_3), 14.6 (CH_3).

^{19}F NMR (283 MHz, $CDCl_3$): δ –146.33 (q, $^1J_{B\text{-}F}$ = 32.7 Hz).

IR (ATR): 3259, 2923, 1701, 1666, 1542, 1468, 1436, 1144, 1070, 760 cm^{-1}.

MS (ESI) m/z (relative intensity): 731 (100) $[M+Na]^+$, 709 (63) $[M+H]^+$.

HR-MS (ESI): m/z calcd for $C_{42}H_{40}BF_2N_6O_3$ $[M+H]^+$: 709.3276, found: 709.3260.

(3*S*,8a*S*)-3-({2-[(*E*)-4-(5,5-Difluoro-3,7-di-*p*-tolyl-5*H*-4λ_4,5λ_4-dipyrrolo[1,2-*c*:2',1'-*f*][1,3,2]diazaborinin-10-yl)styryl]-1-[pyridin-2-yl]-1*H*-indol-3-yl}methyl)hexahydropyrrolo[1,2-*a*]pyrazine-1,4-dione (55)

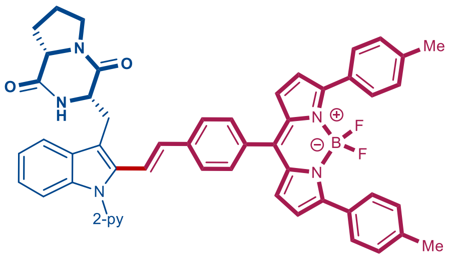

The general procedure **D** was followed using (3*S*,8*aS*)-3-{[1-(pyridin-2-yl)-1*H*-indol-3-yl]methyl}hexahydropyrrolo[1,2-*a*]pyrazine-1,4-dione (**4ai**) (36.1 mg, 0.10 mmol), 10-(4-ethynylphenyl)-5,5-difluoro-3,7-di-*p*-tolyl-5*H*-4λ_4,5λ_4-dipyrrolo[1,2-*c*:2',1'-*f*][1,3,2]diazaborinine (**B6**) (51.9 mg, 0.11 mmol), $MnBr(CO)_5$ (5.5 mg, 20 mol %) and (1-Ad)CO_2H (7.2 mg, 40 mol %) in 1,4-dioxane (1.0 mL). Purification by column chromatography on silica gel (CH_2Cl_2/MeOH: 98/2) yielded **55** (55.7 mg, 67%) as a purple solid.

M. p.: 191 – 194 ºC.

^{1}H NMR (400 MHz, $CDCl_3$): δ 8.78 (d, J = 5.0 Hz, 1H), 7.97 (ddd, J = 7.7, 7.7, 2.0 Hz, 1H), 7.81 (d, J = 8.0 Hz, 4H), 7.72 – 7.65 (m, 1H), 7.56 (d, J = 8.0 Hz, 2H), 7.52 – 7.46 (m, 4H), 7.44 (dd, J = 7.5, 5.0 Hz, 1H), 7.34 – 7.29 (m, 2H), 7.27 – 7.20 (m, 5H), 6.90 (d, J = 4.3 Hz, 2H), 6.68 (d, J = 16.8 Hz, 1H), 6.64 (d, J = 4.3 Hz, 2H), 5.73 (s, 1H), 4.64 – 4.47 (m, 1H), 4.17 – 3.99 (m, 2H), 3.73 (dt, J = 11.6, 7.9 Hz, 1H), 3.64 (ddd, J = 11.6, 8.8, 2.9 Hz, 1H), 3.38 (dd, J = 15.2, 11.6 Hz, 1H), 2.45 – 2.32 (m, 7H), 2.18 – 2.01 (m, 2H), 2.01 – 1.86 (m, 1H).

^{13}C NMR (101 MHz, $CDCl_3$): δ 169.3 (C_q), 165.4 (C_q), 158.8 (C_q), 151.4 (C_q), 149.8 (CH), 142.7 (C_q), 139.7 (C_q), 138.6 (C_q), 138.5 (CH), 138.1 (C_q), 136.1 (C_q), 134.6 (C_q), 134.1 (C_q), 132.0 (CH), 131.1 (CH), 130.4 (CH), 129.8 (C_q), 129.4 (CH), 129.0 (CH), 128.2 (C_q), 126.2 (CH), 124.5 (CH), 122.6 (CH), 122.0 (CH), 121.8 (CH), 120.7 (CH), 118.5 (CH), 118.4 (CH), 112.2 (C_q), 111.2 (CH), 59.2 (CH), 55.0 (CH), 45.5 (CH_2), 28.3 (CH_2), 26.1 (CH_2), 22.6 (CH_2), 21.4 (CH_3).

^{19}F NMR (377 MHz, $CDCl_3$): δ –132.63 (q, $^1J_{B\text{-}F}$ = 31.8 Hz).

IR (ATR): 3372, 1660, 1561, 1537, 1464, 1429, 1278, 1137, 1068, 1055, 738 cm^{-1}.

MS (ESI) m/z (relative intensity): 855 (66) $[M+Na]^+$, 833 (100) $[M+H]^+$.

HR-MS (ESI): m/z calcd for $C_{52}H_{44}BF_2N_6O_2$ $[M+H]^+$: 833.3590, found: 833.3573.

(3*S*,6*S*)-3-({1*H*-Indol-3-yl}methyl)-6-({2-[(*E*)-4-(5,5-difluoro-1,3,7,9-tetramethyl-5*H*-4λ_4,5λ_4-dipyrrolo[1,2-*c*:2',1'-*f*][1,3,2]diazaborinin-10-yl)styryl]-1-[pyridin-2-yl]-1*H*-indol-3-yl}methyl)piperazine-2,5-dione (56)

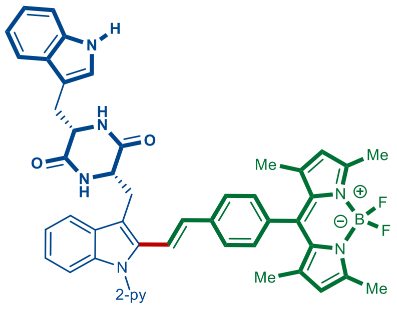

The general procedure **D** was followed using (3*S*,6*S*)-3-[(1*H*-indol-3-yl)methyl]-6-{[1-(pyridin-2-yl)-1*H*-indol-3-yl]methyl}piperazine-2,5-dione (**4aj**) (44.9 mg, 0.10 mmol), 10-(4-ethynylphenyl)-5,5-difluoro-1,3,7,9-tetramethyl-5*H*-4λ_4,5λ_4-dipyrrolo[1,2-*c*:2',1'-*f*][1,3,2]diazaborinine (**B2**) (38.3 mg, 0.11 mmol), $MnBr(CO)_5$ (5.5 mg, 20 mol %) and (1-Ad)CO_2H (7.2 mg, 40 mol %) in 1,4-dioxane (1.0 mL). Purification by column chromatography on silica gel (CH_2Cl_2/MeOH: 96/4) yielded **56** (44.7 mg, 56%) as an orange solid.

M. p.:202 ºC (decomposition).

^{1}H NMR (400 MHz, $CDCl_3$): δ 8.73 (dd, *J* = 4.9, 1.8 Hz, 1H), 8.28 (s, 1H), 7.94 (ddd, *J* = 7.7, 7.7, 1.9 Hz, 1H), 7.69 – 7.60 (m, 1H), 7.49 (d, *J* = 7.9 Hz, 2H), 7.46 – 7.38 (m, 3H), 7.38 – 7.34 (m, 1H), 7.27 – 7.14 (m, 8H), 6.88 – 6.82 (m, 1H), 6.78 (d, *J* = 16.7 Hz, 1H), 6.07 (s, 1H), 5.98 (s, 2H), 5.92 (s, 1H), 4.36 (d, *J* = 9.1 Hz, 1H), 4.29 (d, *J* = 7.4 Hz, 1H), 3.69 (dd, *J* = 14.8, 3.0 Hz, 1H), 3.41 (dd, *J* = 14.8, 3.4 Hz, 1H), 2.98 – 2.81 (m, 2H), 2.57 (s, 6H), 1.41 (s, 6H).

^{13}C NMR (101 MHz, $CDCl_3$): δ 167.2 (C_q), 166.7 (C_q), 155.5 (C_q), 151.4 (C_q), 149.8 (CH), 143.0 (C_q), 141.3 (C_q), 138.6 (CH), 137.7 (C_q), 137.6 (C_q), 136.3 (C_q), 134.5 (C_q), 134.4 (C_q), 131.7 (CH), 131.3 (C_q), 128.6 (C_q), 128.4 (CH), 127.1 (CH), 126.8 (C_q), 124.1 (CH), 123.9 (CH), 122.7 (CH), 122.7 (CH), 122.1 (CH), 121.9 (CH), 121.2 (CH), 120.1 (CH), 118.9 (CH), 118.9 (CH), 118.1 (CH), 112.0 (C_q), 111.4 (CH), 110.8 (CH), 109.2 (C_q), 56.1 (CH), 55.3 (CH), 30.4 (CH_2), 30.0 (CH_2), 14.6 (CH_3), 14.6 (CH_3).

^{19}F NMR (377 MHz, $CDCl_3$): δ –146.17 (q, $^1J_{B-F}$ = 31.9 Hz).

IR (ATR): 3229, 1666, 1541, 1506, 1454, 1434, 1153, 973, 739 cm^{-1}.

MS (ESI) m/z (relative intensity): 820 (100) $[M+Na]^+$, 798 (65) $[M+H]^+$.

HR-MS (ESI): m/z calcd for $C_{48}H_{43}BF_2N_7O_2$ $[M+H]^+$: 798.3542, found: 798.3527.

(3*S*,6*S*)-3-[(1*H*-indol-3-yl)methyl]-6-({2-[(*E*)-4-(5,5-difluoro-1,3,7,9-tetramethyl-5*H*-4λ_4,5λ_4-dipyrrolo[1,2-*c*:2',1'-*f*][1,3,2]diazaborinin-10-yl)styryl]-1-[pyridin-2-yl]-1*H*-indol-3-yl}methyl)piperazine-2,5-dione (57)

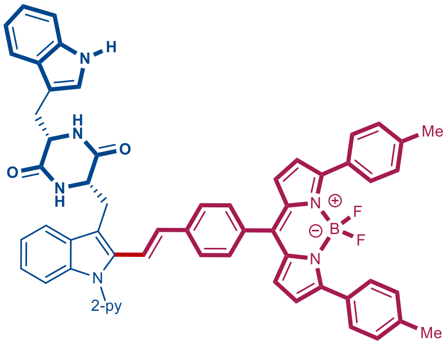

The general procedure **D** was followed using (3*S*,6*S*)-3-[(1*H*-indol-3-yl)methyl]-6-{[1-(pyridin-2-yl)-1*H*-indol-3-yl]methyl}piperazine-2,5-dione (**4ak**) (44.9 mg, 0.10 mmol), 10-(4-ethynylphenyl)-5,5-difluoro-3,7-di-*p*-tolyl-5*H*-4λ_4,5λ_4-dipyrrolo[1,2-*c*:2',1'-*f*][1,3,2]diazaborinine (**B6**) (51.9 mg, 0.11 mmol), $MnBr(CO)_5$ (5.5 mg, 20 mol %) and $(1\text{-Ad})CO_2H$ (7.2 mg, 40 mol %) in 1,4-dioxane (1.0 mL). Purification by column chromatography on silica gel (CH_2Cl_2/MeOH: 95/5) yielded **57** (58.9 mg, 64%) as a purple solid.

M. p.: 189 – 191 ºC.

^{1}H NMR (400 MHz, $CDCl_3$): δ 8.72 (d, J = 3.9 Hz, 1H), 8.27 (brs, 1H), 7.90 (ddd, J = 7.9, 5.8, 2.0 Hz, 1H), 7.83 – 7.75 (m, 4H), 7.66 – 7.56 (m, 1H), 7.52 – 7.41 (m, 5H), 7.39 (dd, J = 7.8, 3.8 Hz, 2H), 7.36 – 7.12 (m, 11H), 6.83 (d, J = 4.0 Hz, 2H), 6.74 – 6.61 (m, 2H), 6.57 (d, J = 4.0 Hz, 2H), 6.09 (d, J = 9.1 Hz, 2H), 4.28 (d, J = 7.3 Hz, 1H), 4.20 (d, J = 8.5 Hz, 1H), 3.61 – 3.48 (m, 1H), 3.44 – 3.23 (m, 1H), 2.98 (ddd, J = 19.0, 5.8, 5.8 Hz, 1H), 2.73 – 2.54 (m, 1H), 2.38 (s, 6H).

^{13}C NMR (101 MHz, $CDCl_3$): δ 167.2 (C_q), 166.7 (C_q), 158.8 (C_q), 151.5 (C_q), 149.7 (CH), 142.8 (C_q), 139.7 (C_q), 138.9 (C_q), 138.6 (CH), 137.8 (C_q), 136.3 (C_q), 136.0 (C_q), 134.7 (C_q), 133.9 (C_q), 131.7 (CH), 131.1 (CH), 130.5 (CH), 129.8 (C_q), 129.4 (CH), 129.4 (CH), 129.0 (CH), 128.6 (C_q), 126.7 (C_q), 126.2 (CH), 124.2 (CH), 123.9 (CH), 122.6 (CH), 122.0 (CH), 121.9 (CH), 120.7 (CH), 120.0 (CH), 119.2 (CH), 118.9 (CH), 118.8 (CH), 112.5 (CH), 111.4 (C_q), 110.9 (C_q), 109.2 (CH), 56.1 (CH), 55.2 (CH), 30.5 (CH_2), 29.8 (CH_2), 21.4 (CH_3).

^{19}F NMR (377 MHz, $CDCl_3$): δ –132.26 (q, $^1J_{B\text{-}F}$ = 31.9 Hz).

IR (ATR): 2919, 1669, 1562, 1539, 1465, 1433, 1279, 1139, 1056, 1018, 739 cm^{-1}.

MS (ESI) m/z (relative intensity): 944 (60) $[M+Na]^+$, 922 (100) $[M+H]^+$.

HR-MS (ESI): m/z calcd for $C_{58}H_{46}BF_2N_7O_2Na$ $[M+Na]^+$: 944.3676, found: 944.3650.

(3S,6S,9S,12S)-6-Benzyl-12-({2-[(E)-4-(5,5-difluoro-1,3,7,9-tetramethyl-5H-4λ_4,5λ_4-dipyrrolo[1,2-*c*:2',1'-*f*][1,3,2]diazaborinin-10-yl)styryl]-1-[pyridin-2-yl]-1H-indol-3-yl}methyl)-3-isobutyl-9-methyl-1,4,7,10,13-pentaazacyclopentadecane-2,5,8,11,14-pentaone (58)

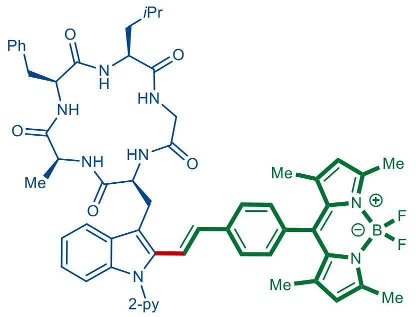

The general procedure **D** was followed using (3S,6S,9S,12S)-6-benzyl-3-isobutyl-9-methyl-12-{[1-(pyridin-2-yl)-1H-indol-3-yl]methyl}-1,4,7,10,13-pentaazacyclopentadecane-2,5,8,11,14-pentaone (**4al**) (65.2 mg, 0.10 mmol), 10-(4-ethynylphenyl)-5,5-difluoro-1,3,7,9-tetramethyl-5H-4λ_4,5λ_4-dipyrrolo[1,2-*c*:2',1'-*f*][1,3,2]diazaborinine (**B2**) (69.2 mg, 0.40 mmol), $MnBr(CO)_5$ (5.5 mg, 20 mol %) and (1-Ad)CO_2H (7.2 mg, 40 mol %) in 1,4-dioxane (1.0 mL). Purification by column chromatography on silica gel (CH_2Cl_2/MeOH: 96/4) yielded **58** (66.9 mg, 67%) as an orange solid.

M. p.: 217 – 219 ºC.

^{1}H NMR (400 MHz, DMSO-d_6): δ 8.84 (dd, J = 5.9, 5.9 Hz, 1H), 8.73 (dd, J = 4.9, 1.9 Hz, 1H), 8.51 (d, J = 8.6 Hz, 1H), 8.20 (d, J = 7.6 Hz, 1H), 8.08 (ddd, J = 7.7, 7.7, 2.0 Hz, 1H), 8.02 (d, J = 8.1 Hz, 1H), 7.95 (d, J = 8.4 Hz, 1H), 7.87 – 7.79 (m, 1H), 7.62 – 7.49 (m, 4H), 7.42 (d, J = 16.7 Hz, 1H), 7.36 – 7.30 (m, 2H), 7.29 – 7.23 (m, 3H), 7.23 – 7.13 (m, 5H), 6.51 (d, J = 16.7 Hz, 1H), 6.17 (s, 2H), 4.53 (ddd, J = 8.4, 8.4, 5.5 Hz, 1H), 4.32 – 4.19 (m, 1H), 4.17 – 3.98 (m, 2H), 3.84 (dd, J = 14.2, 5.9 Hz, 1H), 3.57 (dd, J = 14.2, 5.3 Hz, 1H), 3.31 – 3.27 (m, 1H), 3.25 – 3.12 (m, 2H), 3.07 (dd, J = 13.6, 6.2 Hz, 1H), 2.45 (s, 6H), 1.53 – 1.46 (m, 2H), 1.38 (s, 6H), 1.26 – 1.17 (m, 4H), 0.89 (d, J = 5.9 Hz, 3H), 0.85 (d, J = 5.8 Hz, 3H).

^{13}C NMR (101 MHz, DMSO-d_6): δ 173.5 (C_q), 172.1 (C_q), 171.6 (C_q), 171.0 (C_q), 169.8 (C_q), 155.4 (C_q), 151.7 (C_q), 150.1 (CH), 143.1 (C_q), 142.2 (C_q), 139.7 (CH), 138.3 (C_q), 138.2 (C_q), 138.2 (C_q), 134.2 (C_q), 133.6 (C_q), 131.1 (C_q), 130.0 (CH), 129.5 (CH), 128.8 (C_q), 128.7 (CH), 128.6 (CH), 127.3 (CH), 126.9 (CH), 124.1 (CH), 123.6 (CH), 122.9 (CH), 121.8 (CH), 121.3 (CH), 119.8 (CH), 118.8 (CH), 115.5 (C_q), 111.0 (CH), 58.3 (CH), 55.6 (CH), 51.5 (CH), 50.1

(CH), 44.2 (CH_2), 41.2 (CH_2), 36.3 (CH_2), 27.3 (CH_2), 24.9 (CH), 23.4 (CH_3), 22.2 (CH_3), 17.3 (CH_3), 14.7 (CH_3), 14.6 (CH_3).

^{19}F NMR (377 MHz, DMSO-*d*$_6$): δ –143.67 (q, $^1J_{B\text{-}F}$ = 28.7 Hz).

IR (ATR): 3306, 2954, 2900, 1655, 1514, 1454, 1437, 1289, 1148, 1080, 740 cm^{-1}.

MS (ESI) m/z (relative intensity): 1022 (100) [M+Na]$^+$, 1000 (75) [M+H]$^+$.

HR-MS (ESI): m/z calcd for $C_{57}H_{60}BF_2N_9O_5Na$ [M+Na]$^+$: 1022.4680, found: 1022.4659.

(3*S*,6*S*,9*S*,12*S*)-6-Benzyl-3-[(*S*)-*sec*-butyl]-12-({2-[(*E*)-4-(5,5-difluoro-1,3,7,9-tetramethyl-5*H*-4λ_4,5λ_4-dipyrrolo[1,2-*c*:2',1'-*f*][1,3,2]diazaborinin-10-yl)styryl]-1-[pyridin-2-yl]-1*H*-indol-3-yl}methyl)-9-methyl-1,4,7,10,13-pentaazacyclopentadecane-2,5,8,11,14-pentaone (59)

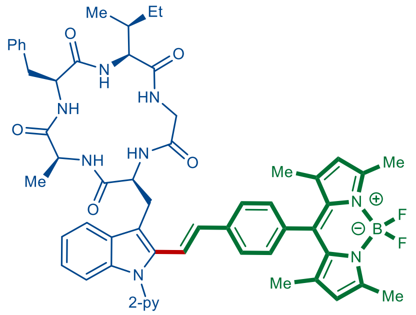

The general procedure **D** was followed using (3*S*,6*S*,9*S*,12*S*)-6-benzyl-3-[(*S*)-*sec*-butyl]-9-methyl-12-{[1-(pyridin-2-yl)-1*H*-indol-3-yl]methyl}-1,4,7,10,13-pentaazacyclopentadecane-2,5,8,11,14-pentaonee (**4am**) (65.2 mg, 0.10 mmol), 10-(4-ethynylphenyl)-5,5-difluoro-1,3,7,9-tetramethyl-5*H*-4λ_4,5λ_4-dipyrrolo[1,2-*c*:2',1'-*f*][1,3,2]diazaborinine (**B2**) (69.2 mg, 0.40 mmol), $MnBr(CO)_5$ (5.5 mg, 20 mol %) and (1-Ad)CO_2H (7.2 mg, 40 mol %) in 1,4-dioxane (1.0 mL). Purification by column chromatography on silica gel (CH_2Cl_2/MeOH: 95/5) yielded **52** (57.9 mg, 58%) as an orange solid.

M. p.: 207 –209 ºC.

^{1}H NMR (400 MHz, DMSO-*d*$_6$): δ 9.05 (t, *J* = 5.9 Hz, 1H), 8.83 (d, *J* = 8.9 Hz, 1H), 8.73 (dd, *J* = 5.0, 1.9 Hz, 1H), 8.37 (d, *J* = 7.3 Hz, 1H), 8.08 (ddd, *J* = 7.7, 7.7, 2.0 Hz, 1H), 7.90 – 7.77 (m, 3H), 7.59 (d, *J* = 8.1 Hz, 2H), 7.58 – 7.50 (m, 2H), 7.44 (d, *J* = 16.8 Hz, 1H), 7.33 (d, *J* = 8.1 Hz, 2H), 7.30 – 7.13 (m, 8H), 6.55 (d, *J* = 16.8 Hz, 1H), 6.16 (s, 2H), 4.52 (ddd, *J* = 9.2, 8.9, 4.2 Hz, 1H), 4.26 (dq, *J* = 7.0, 6.8 Hz, 1H), 4.12 (dd, *J* = 9.1, 7.4 Hz, 1H), 3.95 (ddd, *J* = 9.6, 6.4, 4.1 Hz, 1H), 3.80 (dd, *J* = 13.7, 5.7 Hz, 1H), 3.61 (dd, *J* = 14.4, 4.1 Hz, 1H), 3.31 – 3.21 (m, 2H), 3.17 –

3.06 (m, 2H), 2.45 (s, 6H), 1.75 – 1.59 (m, 1H), 1.47 – 1.40 (m, 1H), 1.38 (s, 6H), 1.25 (d, J = 6.8 Hz, 3H), 1.11 – 0.97 (m, 1H), 0.91 – 0.78 (m, 6H).

^{13}C NMR (101 MHz, DMSO-d_6): δ 172.9 (C_q), 171.6 (C_q), 171.4 (C_q), 170.4 (C_q), 169.3 (C_q), 154.9 (C_q), 151.2 (C_q), 149.6 (CH), 142.6 (C_q), 141.7 (C_q), 139.2 (CH), 137.8 (C_q), 137.8 (C_q), 133.6 (C_q), 133.1 (C_q), 130.6 (C_q), 129.6 (CH), 128.9 (CH), 128.4 (C_q), 128.2 (CH), 128.2 (CH), 126.8 (CH), 126.4 (CH), 123.6 (CH), 123.1 (CH), 122.4 (CH), 121.3 (CH), 120.8 (CH), 119.3 (CH), 118.3 (CH), 115.5 (C_q), 110.5 (CH), 59.1 (CH), 56.5 (CH), 55.1 (CH), 49.2 (CH), 44.0 (CH_2), 37.1 (CH), 35.6 (CH_2), 26.8 (CH_2), 24.4 (CH_2), 16.8 (CH_3), 15.5 (CH_3), 14.2 (CH_3), 11.0 (CH_3).(One aromatic C_q and one CH_3 are missing due to overlap, the overlap was verified by HSQC and HMBC analysis, showing that the peak at 137.8 corresponds to two carbons and the peak at 14.2 corresponds to the two CH_3).

^{19}F NMR (377 MHz, DMSO-d_6) δ –143.67 (q, $^{1}J_{B\text{-}F}$ = 30.1 Hz).

IR (ATR): 3313, 3288, 2914, 1658, 1513, 1468, 1150, 1081, 975 cm^{-1}.

MS (ESI) m/z (relative intensity): 1022 (100) [M+Na]$^+$, 1000 (80) [M+H]$^+$.

HR-MS (ESI): m/z calcd for $C_{57}H_{61}BF_2N_9O_5$ [M+H]$^+$: 1000.4861, found: 1000.4836.

Methyl (*S*)-2-[(*tert*-butoxycarbonyl)amino]-3-{2-[4-(5,5-difluoro-1,3,7,9-tetramethyl-5*H*-4λ_4,5λ_4-dipyrrolo[1,2-*c*:2',1'-*f*][1,3,2]diazaborinin-10-yl)phenyl]-1-[pyridin-2-yl]-1*H*-indol-3-yl}propanoate (60)

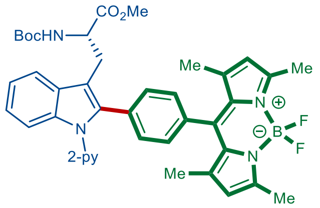

A suspension of peptide methyl N_a-(*tert*-butoxycarbonyl)-1-(pyridin-2-yl)-*L*-tryptophanate (**1**) (79.1 mg, 0.20 mmol), 5,5-difluoro-10-(4-iodophenyl)-1,3,7,9-tetramethyl-5*H*-4λ_4,5λ_4-dipyrrolo[1,2-*c*:2',1'-*f*][1,3,2]diazaborinine (**BS1**)(99.1 mg, 0.22 mmol), [RuCl$_2$(*p*-cymene)]$_2$ (6.1 mg, 5.0 mol %), PPh_3 (15.7 mg, 30 mol %) and K_3PO_4 (127.3 mg, 3 equiv) in *m*-xylene (2.0 mL) was stirred at 120 °C for 20 h. After cooling to ambient temperature, CH_2Cl_2 (10 mL) was added and the mixture was concentrated *in vacuo*. Purification by column chromatography on silica gel (CH_2Cl_2/EtOAc: 10/1) yielded **60** (63.4 mg, 44%) as an orange solid.

M. p.: 136 – 138 ºC.

^{1}**H NMR** (400 MHz, $CDCl_3$): δ 8.54 (d, J = 5.0 Hz, 1H), 7.83 – 7.65 (m, 2H), 7.55 (ddd, J = 7.6, 7.6, 1.9 Hz, 1H), 7.45 (d, J = 7.7 Hz, 2H), 7.35 – 7.29 (m, 4H), 7.19 (dd, J = 7.5, 4.9 Hz, 1H), 6.94 (d, J = 7.7 Hz, 1H), 6.02 (s, 2H), 4.79 (d, J = 8.1 Hz, 1H), 4.49 (ddd, J = 8.1, 8.0 ,6.1 Hz, 1H), 3.62 (s, 3H), 3.35 (dd, J = 14.3, 6.1 Hz, 1H), 3.26 (dd, J = 14.3, 8.0 Hz, 1H), 2.58 (s, 6H), 1.44 (s, 6H), 1.33 (s, 9H).

^{13}**C NMR** (101 MHz, $CDCl_3$): δ 172.9 (C_q), 155.7 (C_q), 154.9 (C_q), 151.4 (C_q), 149.2 (CH), 142.8 (C_q), 140.8 (C_q), 137.5 (CH), 137.1 (C_q), 136.7 (C_q), 134.8 (C_q), 132.9 (C_q), 131.4 (CH), 131.3 (C_q), 128.4 (C_q), 128.3 (CH), 123.8 (CH), 121.5 (CH), 121.5 (CH), 121.4 (CH), 119.2 (CH), 111.9 (C_q), 111.5 (CH), 79.7 (C_q), 54.2 (CH), 52.3 (CH_3), 28.2 (CH_3), 28.0 (CH_2), 14.7 (CH_3), 14.6 (CH_3).). (One aromatic CH is missing due to overlap, the overlap was verified by HSQC analysis, showing that the peak at 121.5 corresponds to two carbons).

^{19}**F NMR** (377 MHz, $CDCl_3$) δ –146.3 (q, $^{1}J_{B\text{-}F}$ = 31.6 Hz).

IR (ATR): 2975, 1742, 1712, 1542, 1507, 1454, 1435, 1364, 1190, 1153, 1048, 973, 740 cm^{-1}.

MS (ESI) m/z (relative intensity): 740 (45) $[M+Na]^+$, 718 (100) $[M+H]^+$, (27).

HR-MS (ESI): m/z calcd for $C_{41}H_{43}BF_2N_5O_4$ $[M+H]^+$: 718.3378, found: 718.3376.

Studies on Potential Racemization

Racemic tryptophan **4b** was employed in the manganese-catalyzed C–H alkynylation and analyzed by HPLC, showing that no racemization occurs during the reaction. The *ee* % of the **7** was determined >99%.

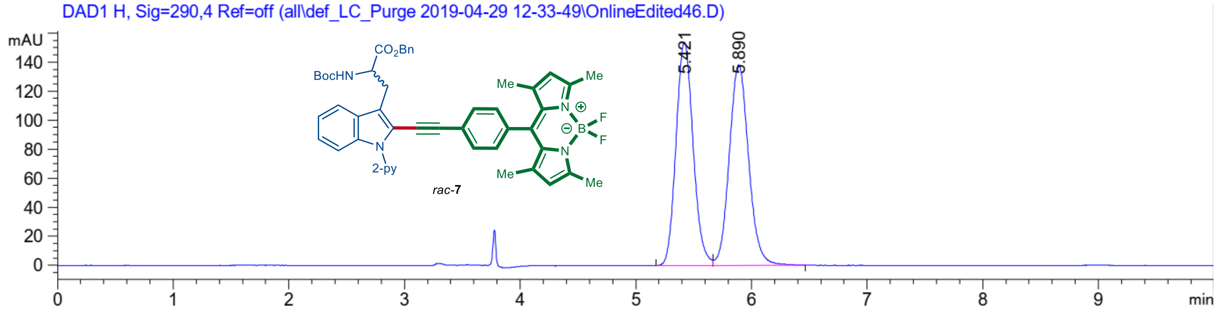

Signal 3: DAD1 H, Sig=290,4 Ref=off

Peak #	RetTime [min]	Type	Width [min]	Area [mAU*s]	Height [mAU]	Area %
1	5.421	BV	0.1610	1582.57568	153.89273	49.5599
2	5.890	VB	0.1799	1610.68066	138.28320	50.4401
Totals :				3193.25635	292.17593	

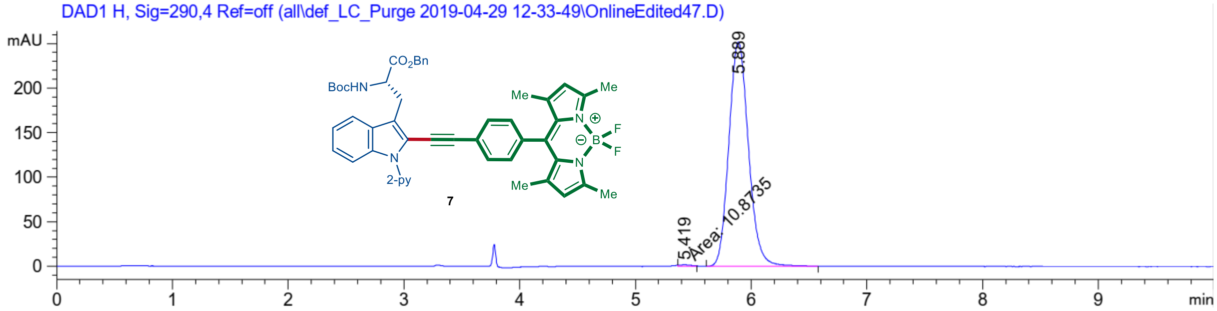

Signal 3: DAD1 H, Sig=290,4 Ref=off

Peak #	RetTime [min]	Type	Width [min]	Area [mAU*s]	Height [mAU]	Area %
1	5.419	MM	0.1200	10.87350	1.51054	0.3653
2	5.889	VV R	0.1835	2965.77881	251.66124	99.6347
Totals :				2976.65231	253.17178	

Supplementary Figure 2. HPLC-Chromatograms of the *rac*-**7** and **7**. HPLC chromatograms were recorded on an Agilent 1290 Infinity using the column CHIRALPAK® ID and *n*-hexane/EtOAc (60:40, 1 mL/min, detection at 280 nm).

Partially racemized tryptophan **4a**[5] was employed in the manganese-catalyzed C–H alkenylation and analyzed by HPLC, showing that no racemization occurs during the reaction. The *ee* % of the **20** was determined >99%.

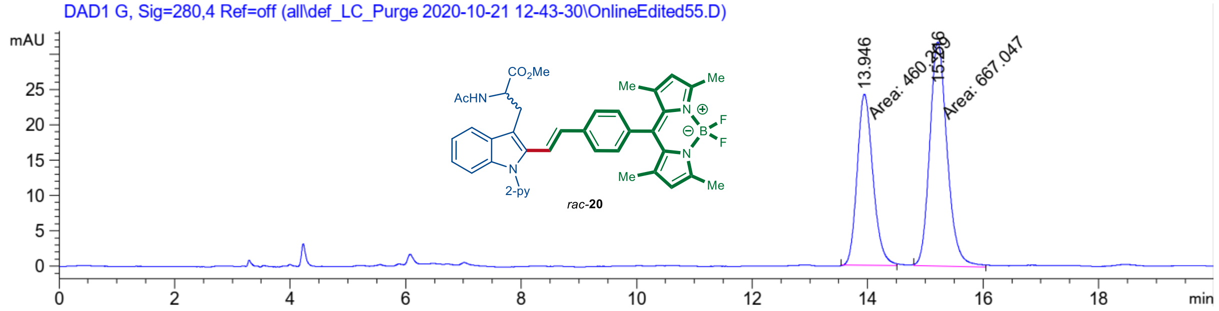

Peak #	RetTime [min]	Type	Width [min]	Area [mAU*s]	Height [mAU]	Area %
1	13.946	MM	0.3168	460.26932	24.21520	40.8288
2	15.216	MM	0.3505	667.04712	31.71710	59.1712
Totals :				1127.31644	55.93230	

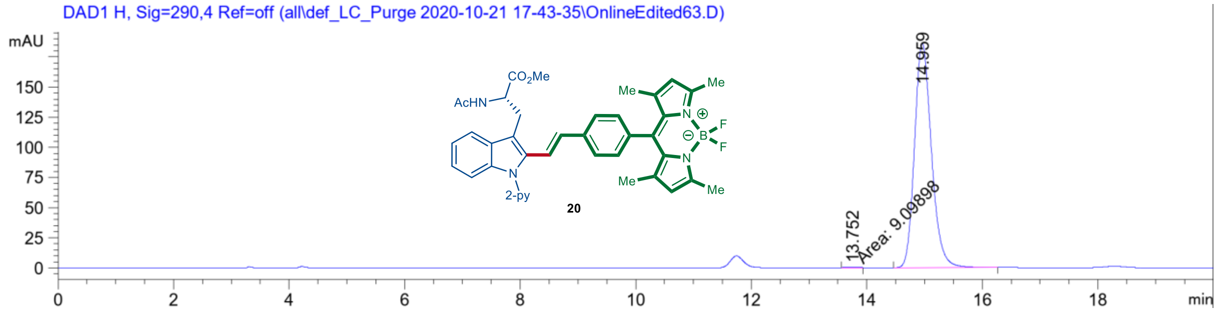

Peak #	RetTime [min]	Type	Width [min]	Area [mAU*s]	Height [mAU]	Area %
1	13.752	MM	0.2731	9.09898	5.55365e-1	0.2394
2	14.959	BB	0.3131	3790.91089	186.50409	99.7606
Totals :				3800.00987	187.05945	

Supplementary Figure 3. HPLC-Chromatograms of the *rac*-**20** and **20**. HPLC chromatograms were recorded on an Agilent 1290 Infinity using the column CHIRALPAK® ID and *n*-hexane/EtOAc (60:40, 1 mL/min, detection at 280 nm).

Supplementary Figures and Table

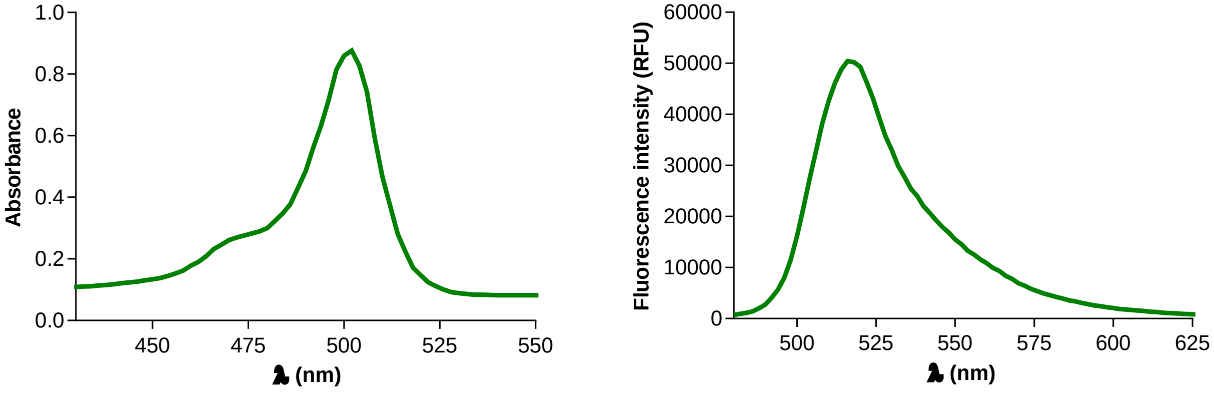

Supplementary Figure 4. Spectral characterization of the amino acid 2. Absorbance (left) and emission (right) spectra of compound **2** (25 μM) in EtOH (λ_{exc}: 450 nm).

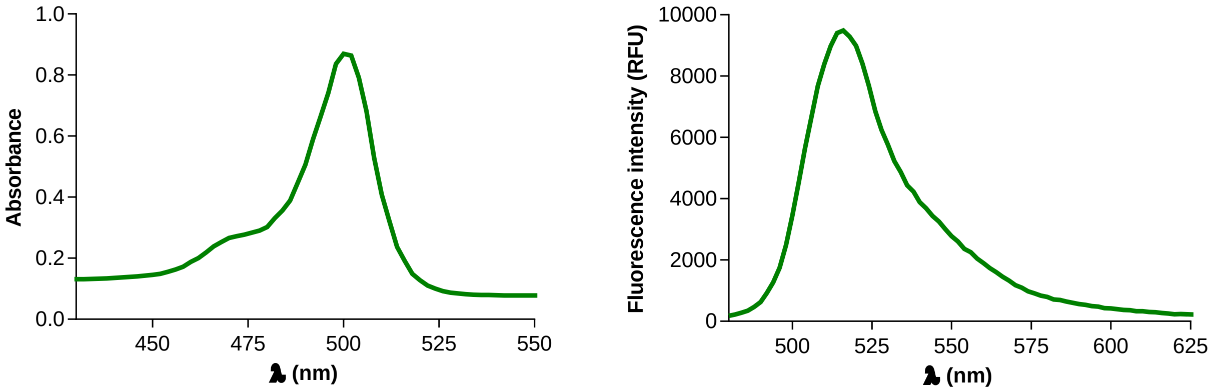

Supplementary Figure 5. Spectral characterization of the amino acid 3. Absorbance (left) and emission (right) spectra of compound **3** (25 μM) in EtOH (λ_{exc}: 450 nm).

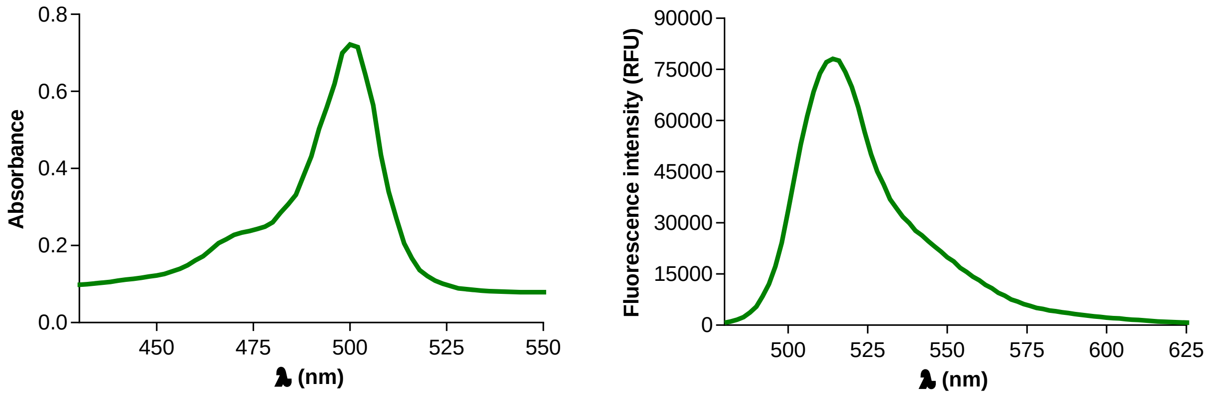

Supplementary Figure 6. Spectral characterization of the amino acid 60. Absorbance (left) and emission (right) spectra of compound **60** (25 µM) in EtOH (λ_{exc}: 450 nm).

Supplementary Table 3. List of screened small molecule drugs and working concentrations.

**Compound**	**concentration (µM)**
lovastatin	30
avasimibe	10
U18666A	30
spermine	30
AR-C155858	10
E-64	10

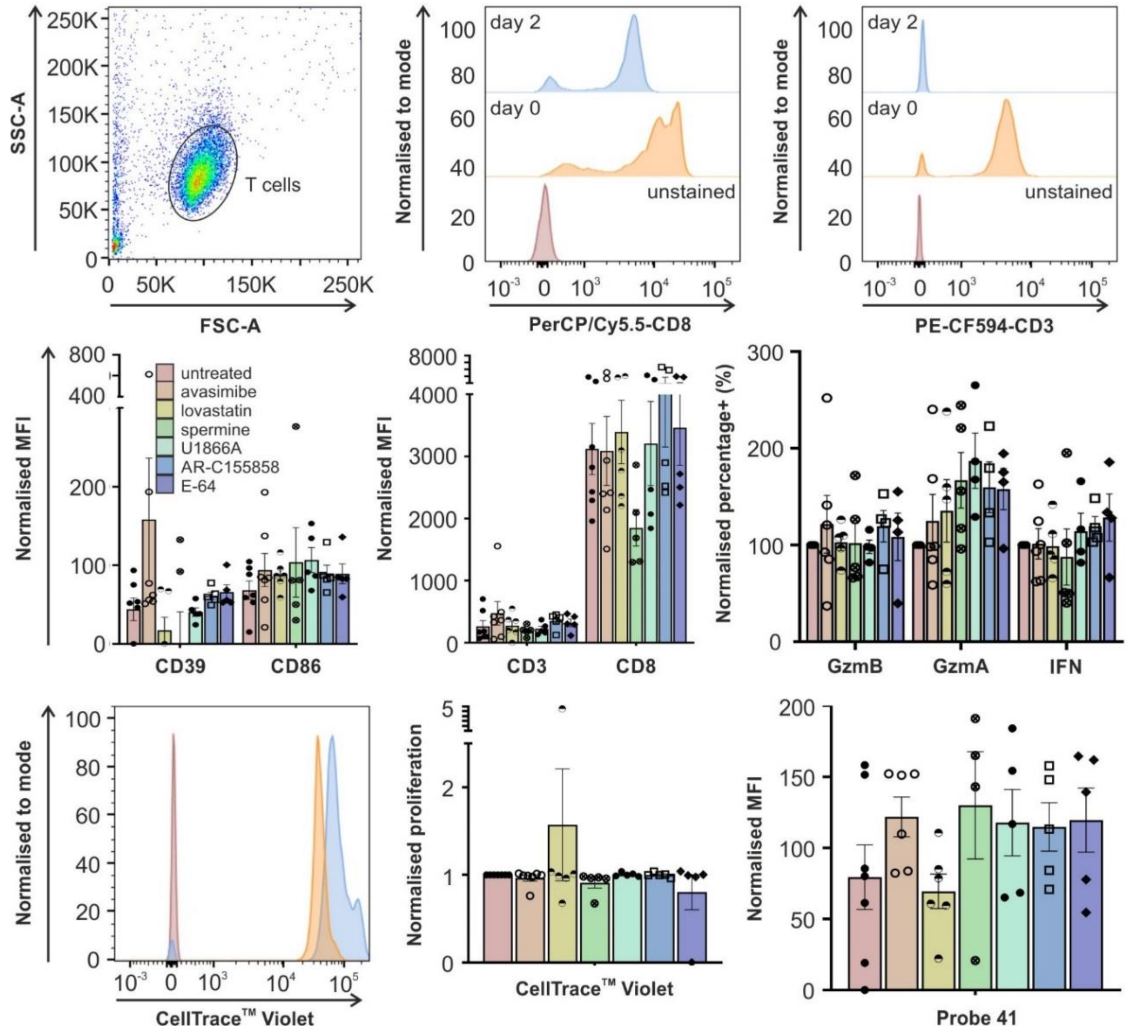

Supplementary Figure 7. **Flow cytometry characterisation of cell surface and intracellular markers of activated CD8+ T cells after incubation with selected drugs.** Upper panel: left) gating of CD8+ T cells, center) histograms of the comparison between CD8 expression on naïve (day 0) and activated (day 2) CD8+ T cells, right) histograms of CD3 downregulation on activated (day 2) and naïve (day 0) CD8+ T cells. Middle panel: normalised mean fluorescence intensity (MFI) of cell surface and intracellular markers in CD8+ T cells after incubation with selected drugs for 24 h. Bottom panel: histogram of Celltrace™ Violet of naïve (blue) and activated CD8+ T cells upon stimulation with 2 µg mL^{-1} αCD3, 5 µg mL^{-1} αCD28 and 80 U mL^{-1} IL-2 followed by 8 h with 1000 U mL^{-1} IL-2. Celltrace™ Violet (λ_{exc}: 405 nm, λ_{em}: 450/50 nm), ef450-CD86 (λ_{exc}: 405 nm, λ_{em}: 450/50 nm), probe **41** (λ_{exc}: 488 nm, λ_{em}: 525/50 nm), AF488-GzmA (λ_{exc}: 488 nm, λ_{em}: 525/50 nm), PE/CF594-CD3 (λ_{exc}: 561 nm, λ_{em}: 610/20 nm), PerCP/Cy5.5-CD8 (λ_{exc}: 488 nm, λ_{em}: 710/50 nm), APC-GzmB (λ_{exc}: 647 nm, λ_{em}: 670/14 nm), APC/Cy7-IFNγ (λ_{exc}:

647 nm, λ_{em}: 780/60 nm). All data was acquired on a 5L LSR flow cytometer and processed with FlowJo with values as means and error bars as SD (n=4-7). Each n represents an independent experiment with CD8 T cells from different subjects. Middle panel: Untreated, avasimibe and lovastatin: extracellular surface markers (n=7), intracellular cytokines (n=6); and spermine, U1866A, AR-C155858 and E-64: extracellular surface markers (n=5) and intracellular cytokines (n=4). Bottom panel: Untreated (n=7); avasimibe, lovastatin (n=6); U18668, AR-C155858 and E-64 (n=5) for Celltrace Violet and Probe 41. Spermine (n=5) for Celltrace Violet and (n=4) for Probe **41**.

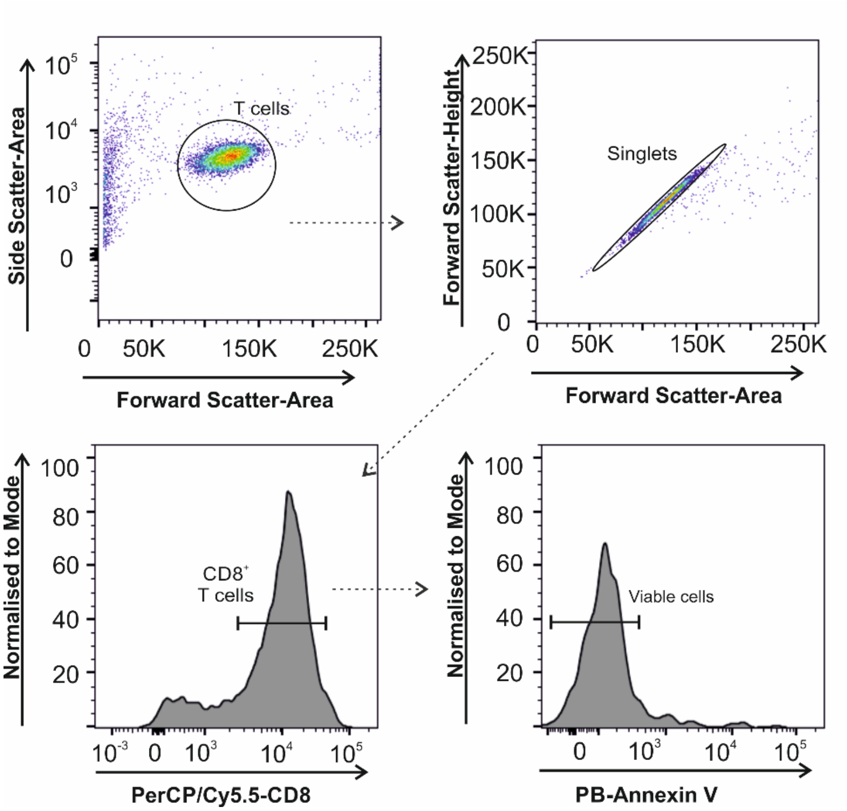

Supplementary Figure 8. Gating strategy of CD8 T cells prior to flow cytometric analysis of CD62L, PD-1 and Probe **41** with or without avasimibe treatment. Upper left: cell gating on FSC-A and SSC-a properties, upper right: single cell gating, lower left: selection of CD8 T cells, lower right: gating on Annexin V negative cells viable population.

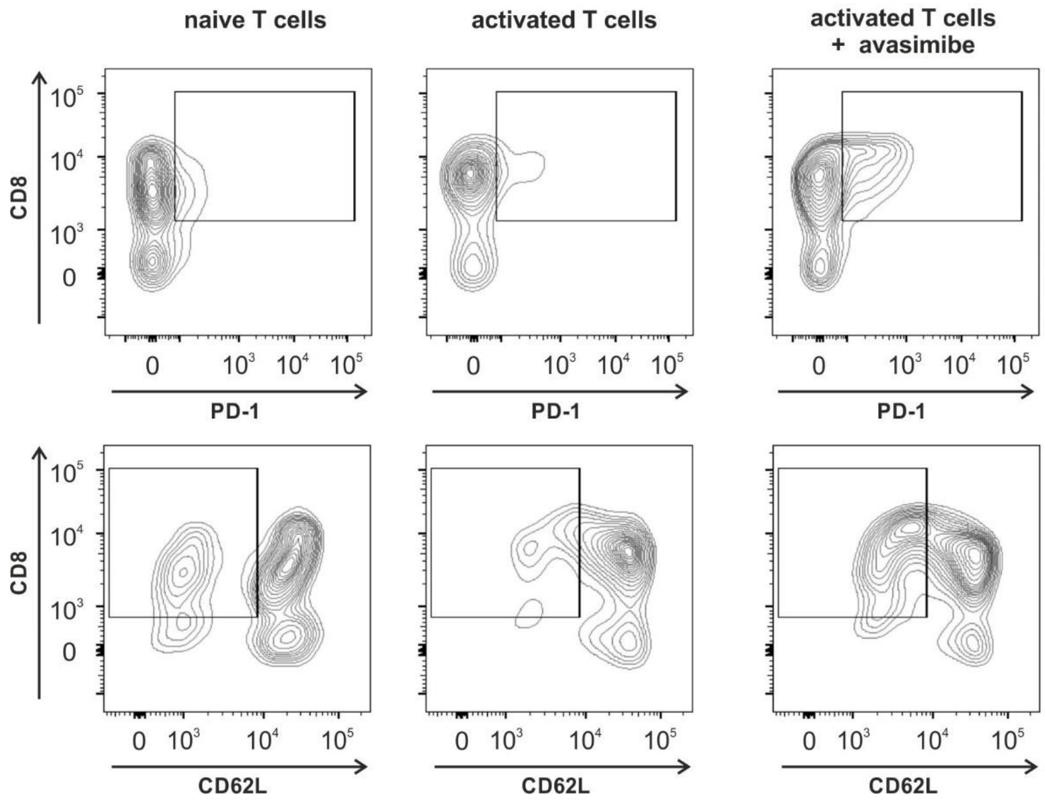

Supplementary Figure 9. Flow cytometry plots of cell surface expression of markers PD-1 and CD62L on naïve, activated CD8+ T cells and activated CD8+ T cells after incubation with avasimibe.

NMR Spectra

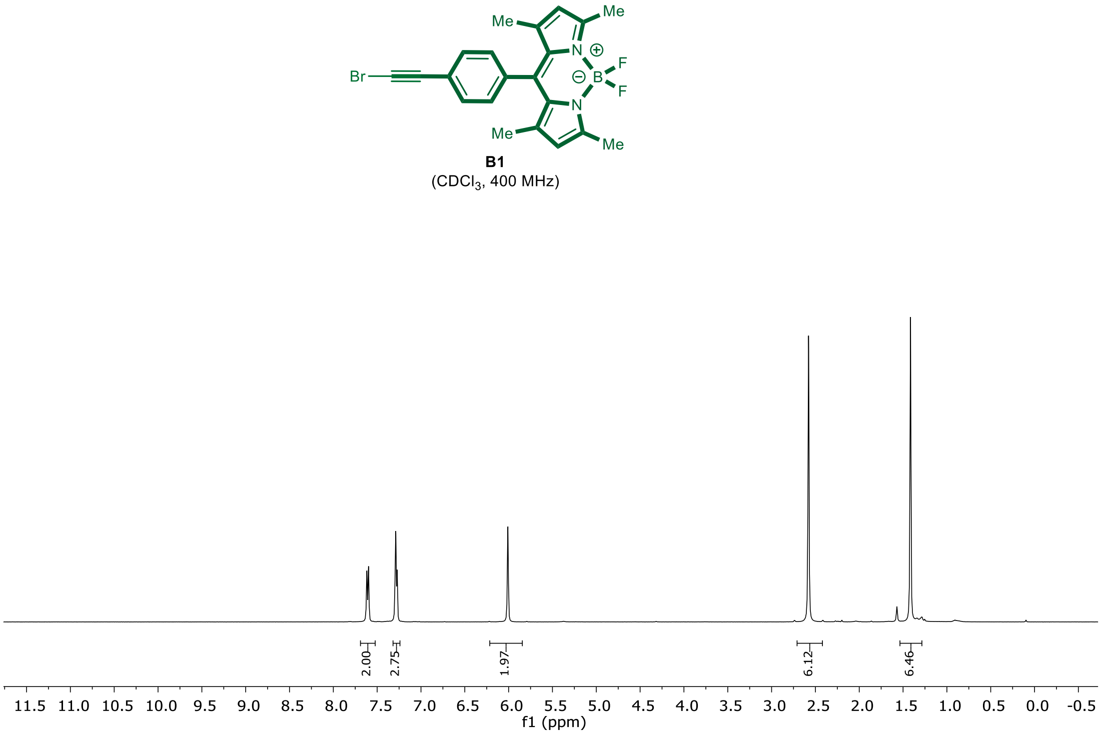

Supplementary Figure 10. ^{1}H-NMR spectrum of **B1**.

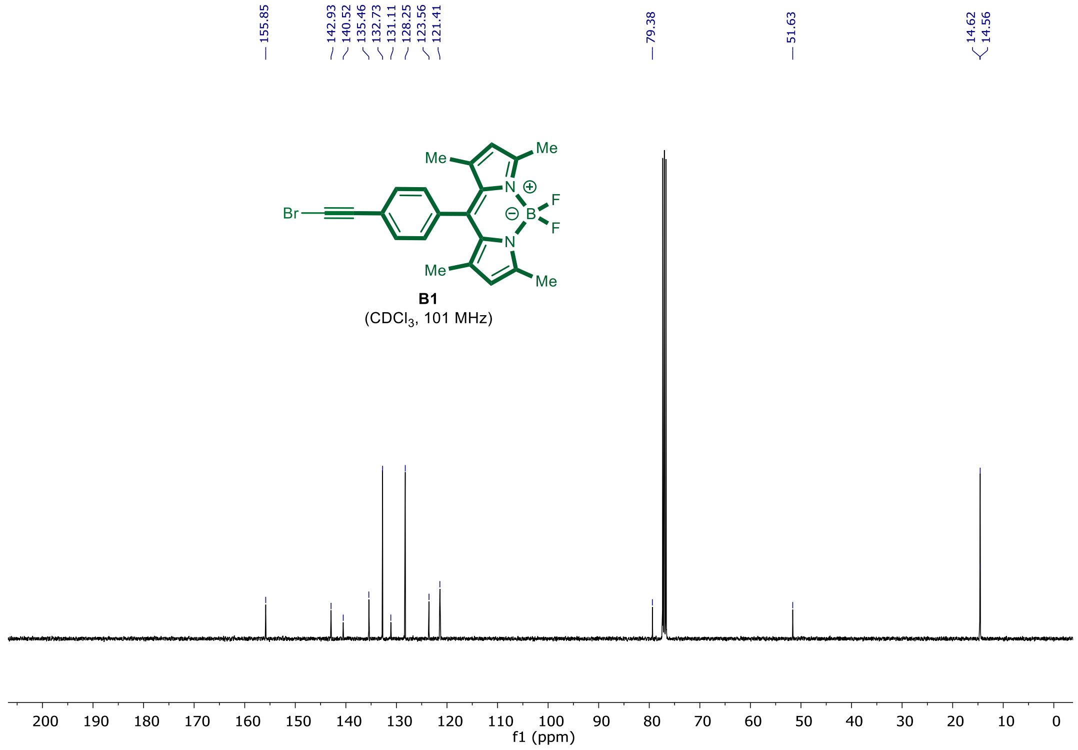

Supplementary Figure 11. ^{13}C-NMR spectrum of **B1**.

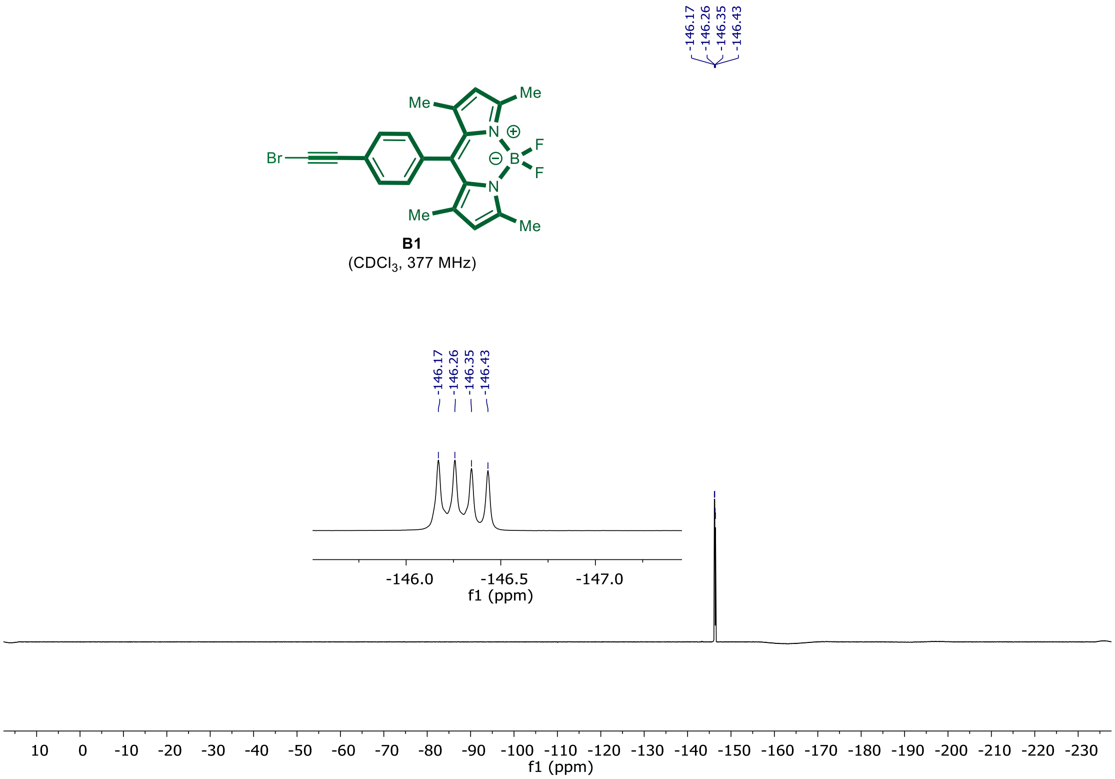

Supplementary Figure 12. ^{19}F-NMR spectrum of **B1**.

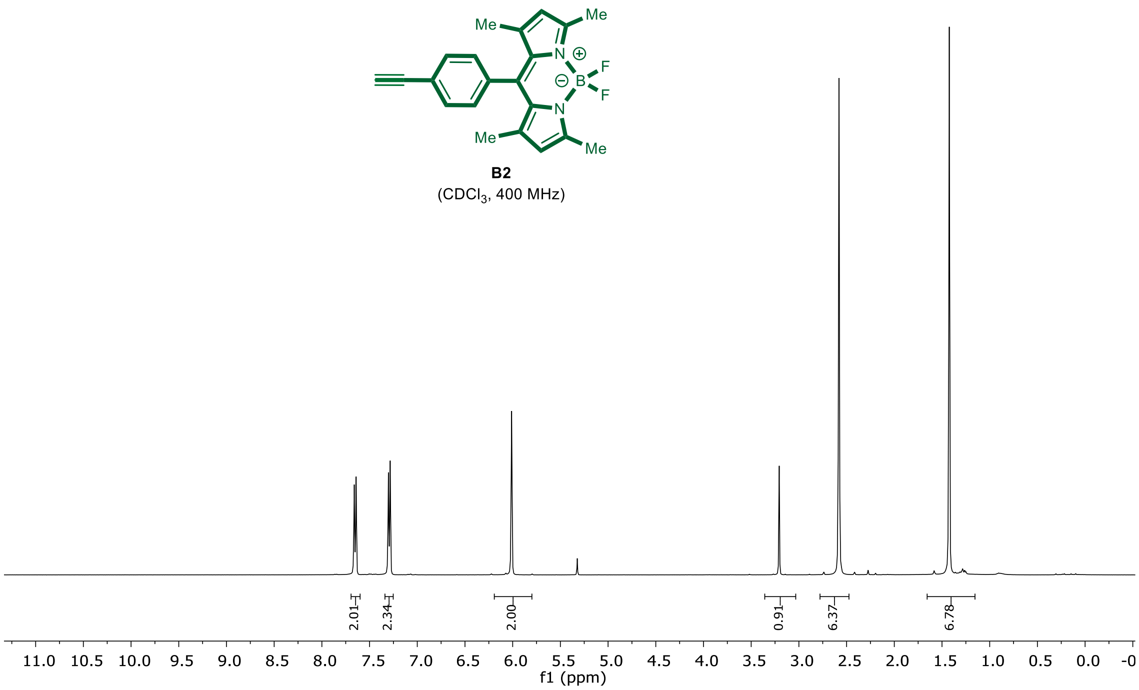

Supplementary Figure 13. ^{1}H-NMR spectrum of **B2**.

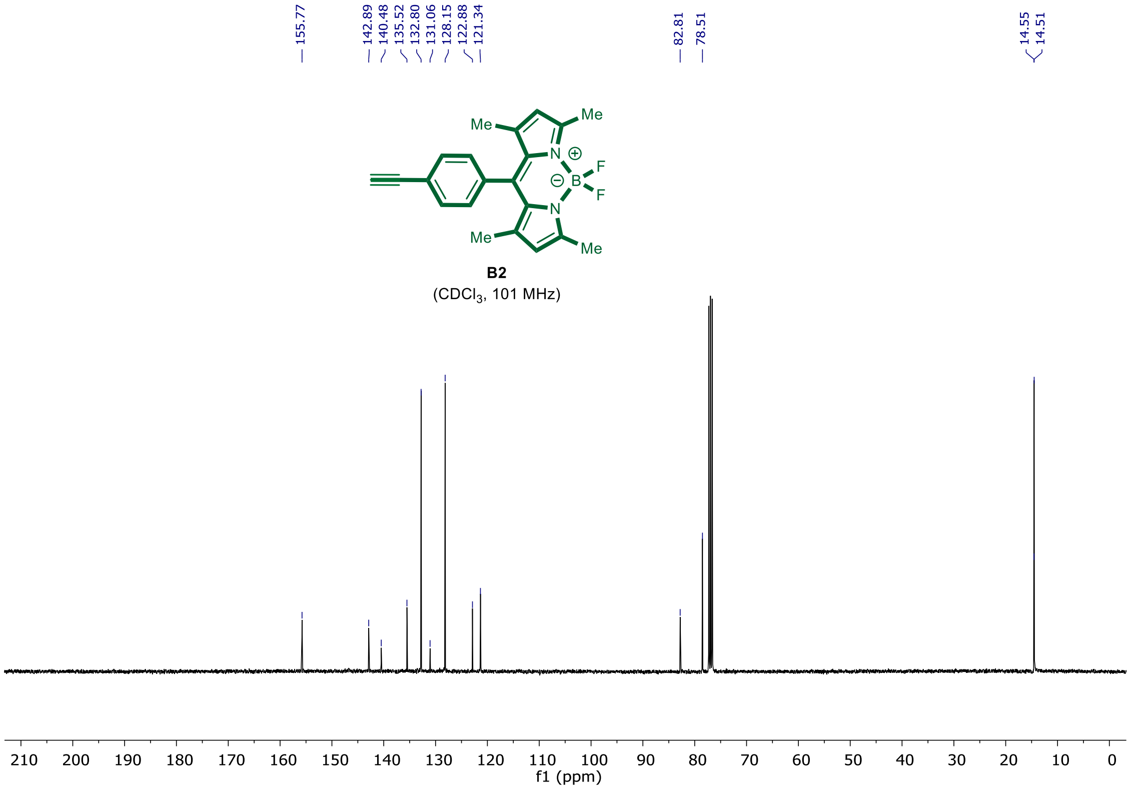

Supplementary Figure 14. ^{13}C-NMR spectrum of **B2**.

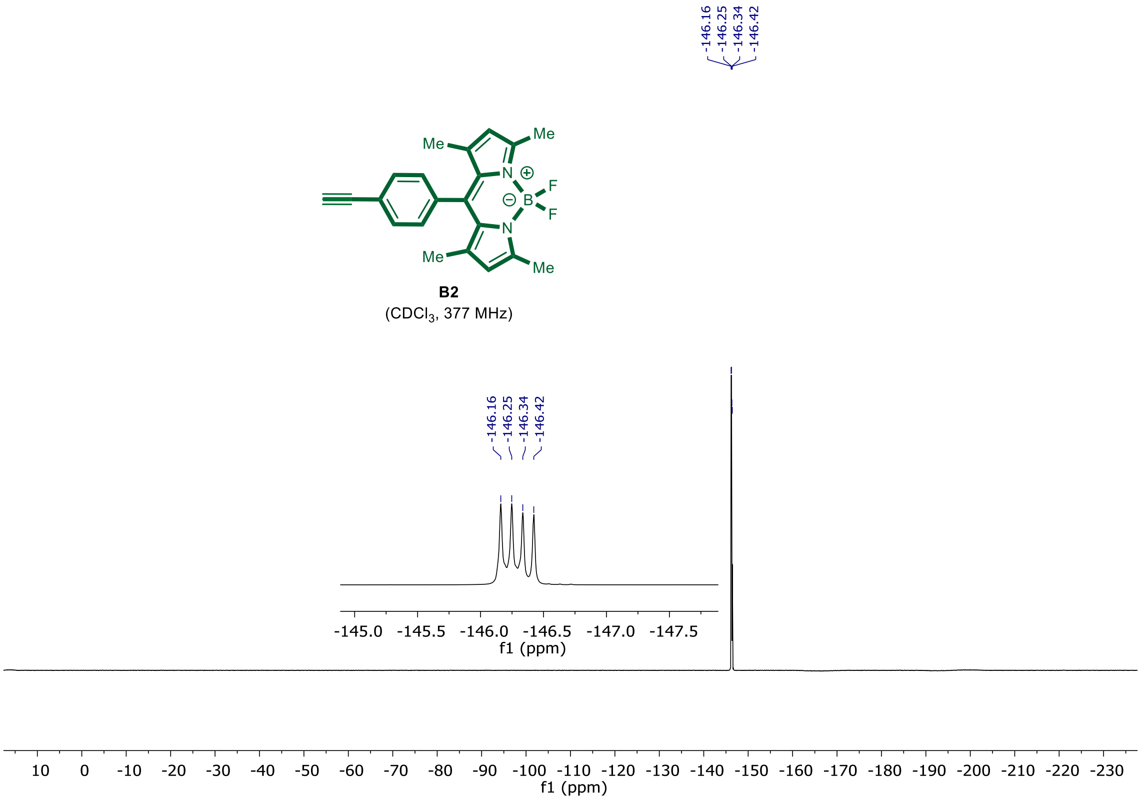

Supplementary Figure 15. ^{19}F-NMR spectrum of **B2**.

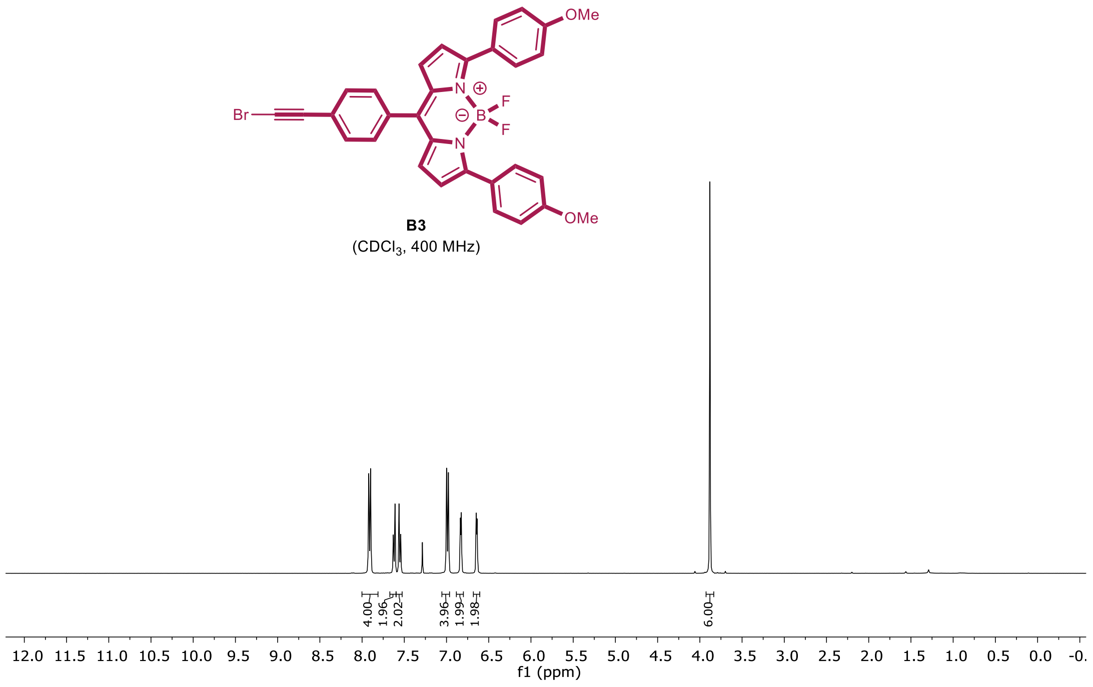

Supplementary Figure 16. ^{1}H-NMR spectrum of **B3**.

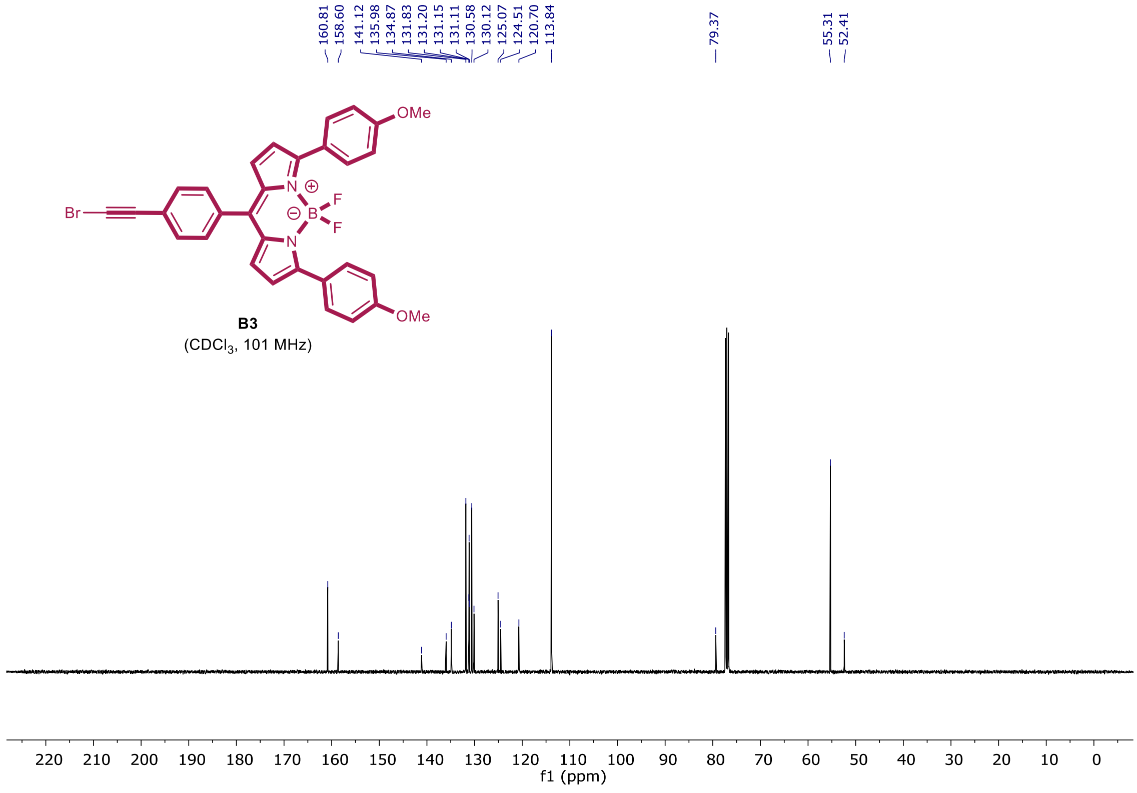

Supplementary Figure 17. ^{13}C-NMR spectrum of **B3**.

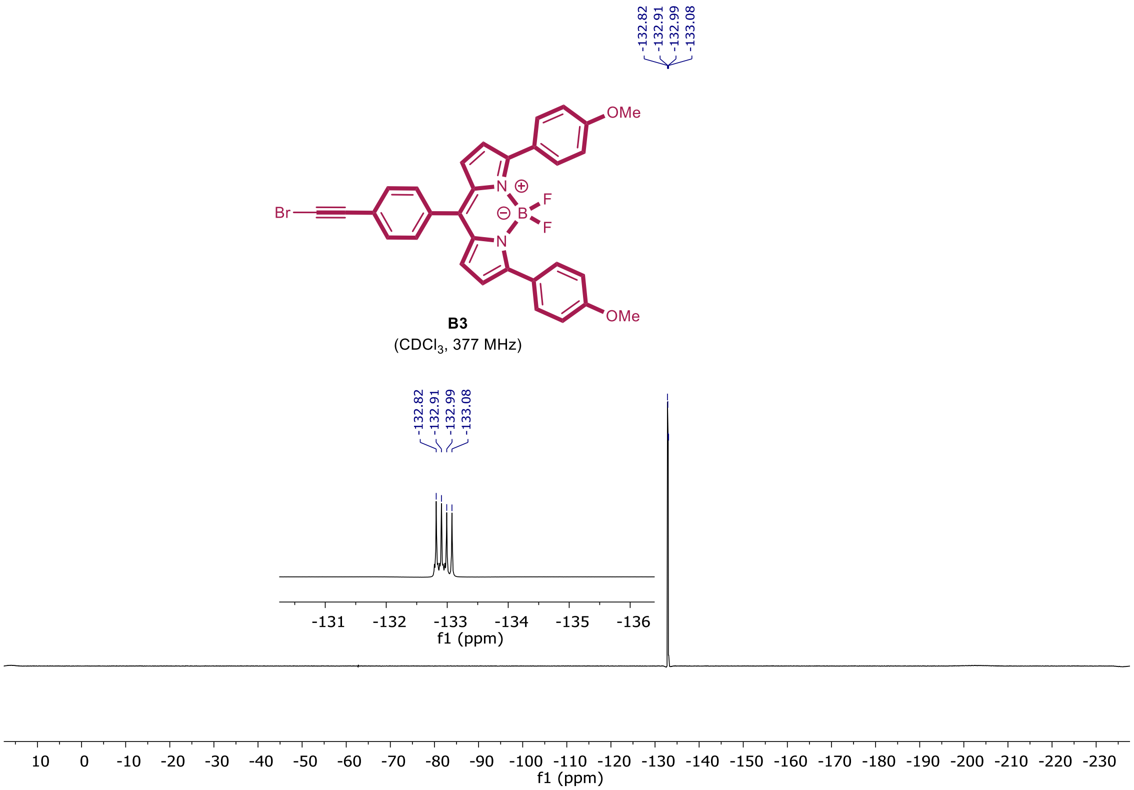

Supplementary Figure 18. ^{19}F-NMR spectrum of **B3**.

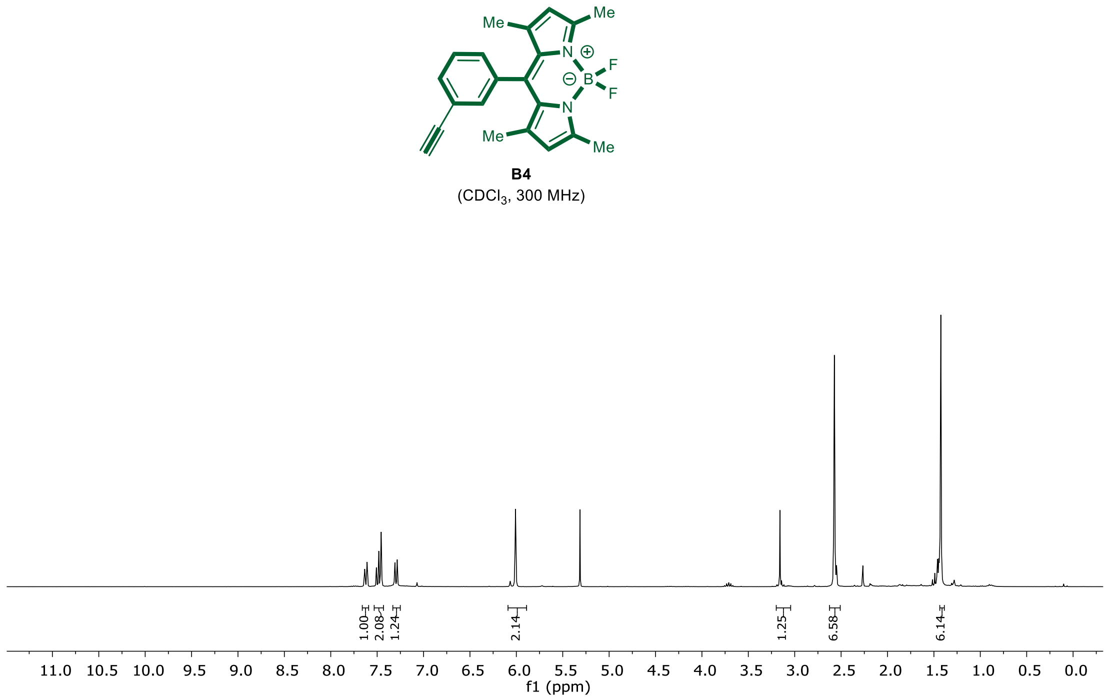

Supplementary Figure 19. ^{1}H-NMR spectrum of **B4**.

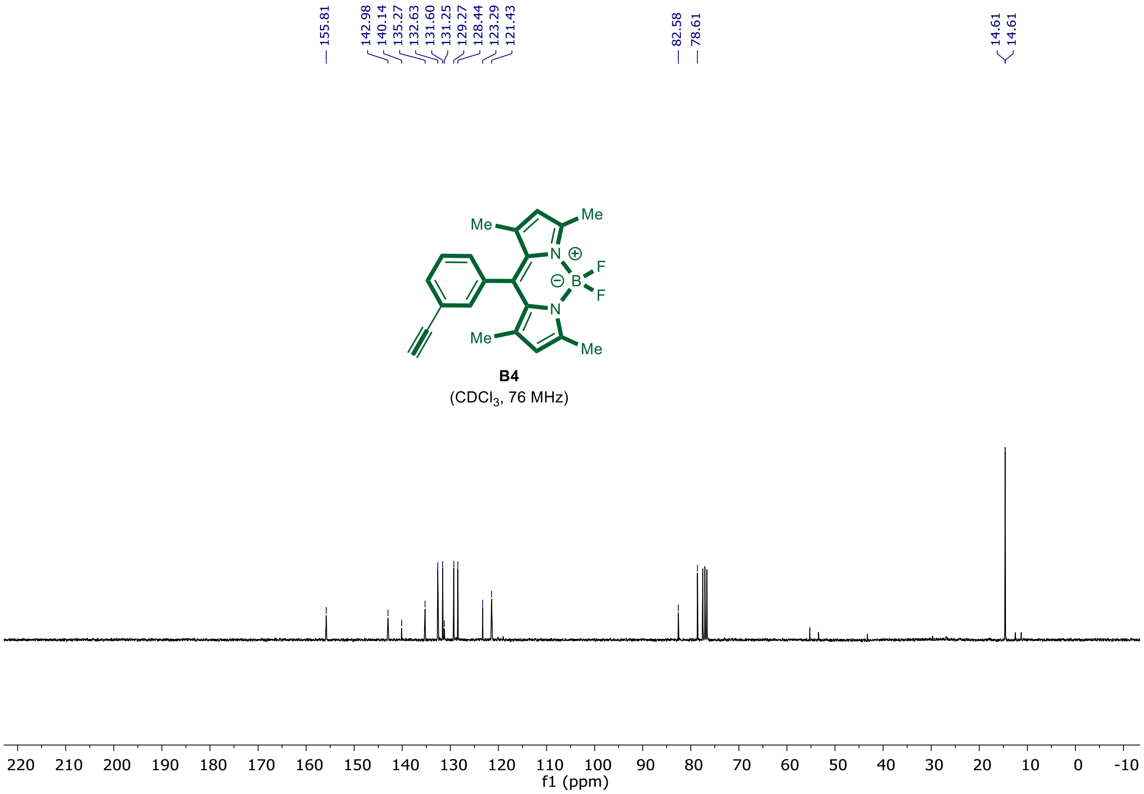

Supplementary Figure 20. ^{13}C-NMR spectrum of **B4**.

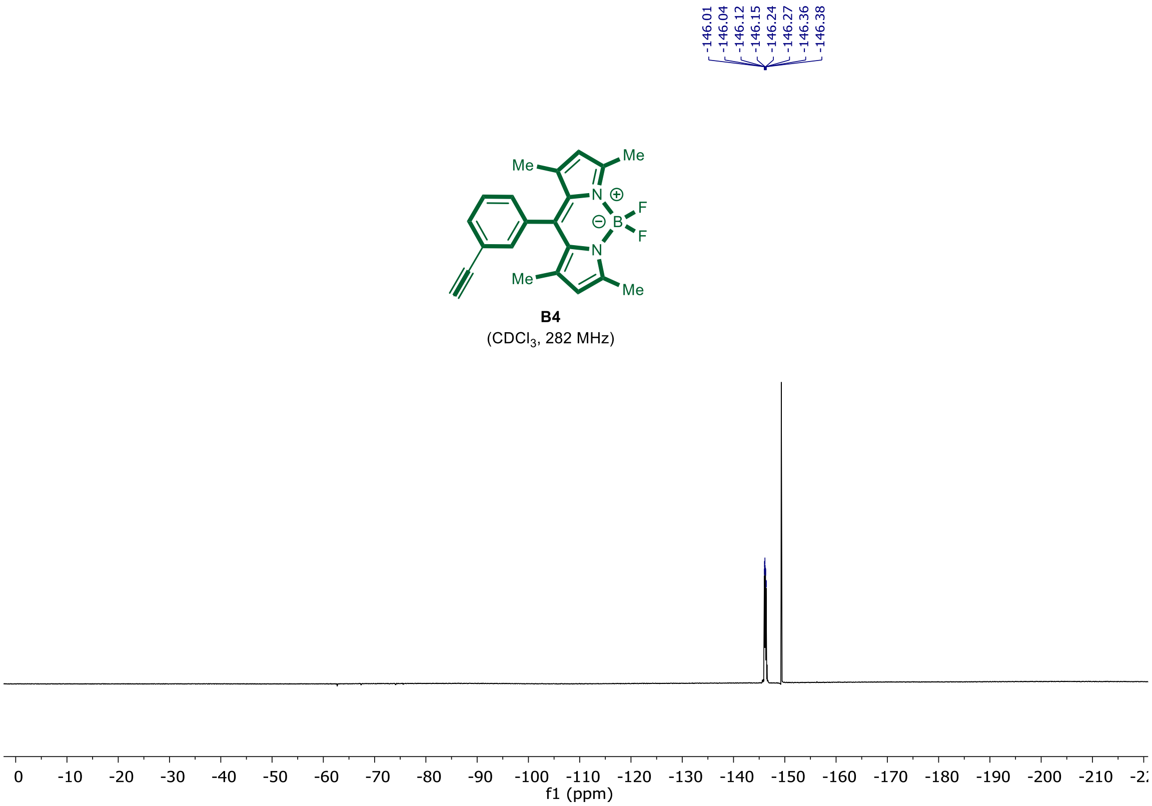

Supplementary Figure 21. ^{19}F-NMR spectrum of **B4**.

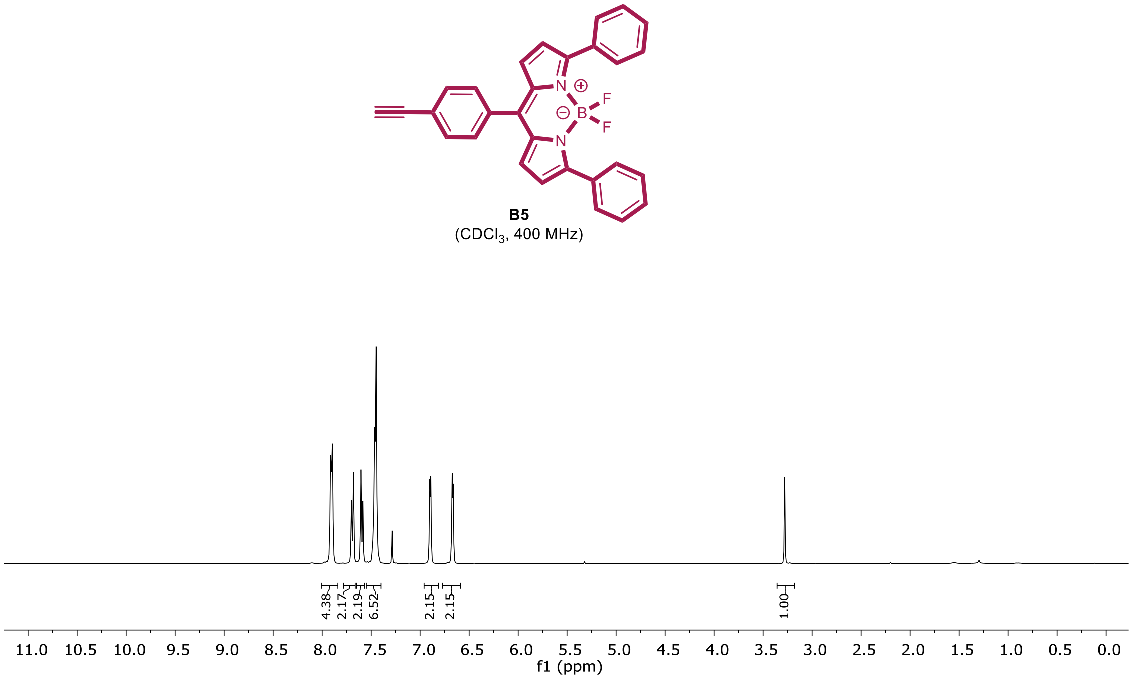

Supplementary Figure 22. ^{1}H-NMR spectrum of **B5**.

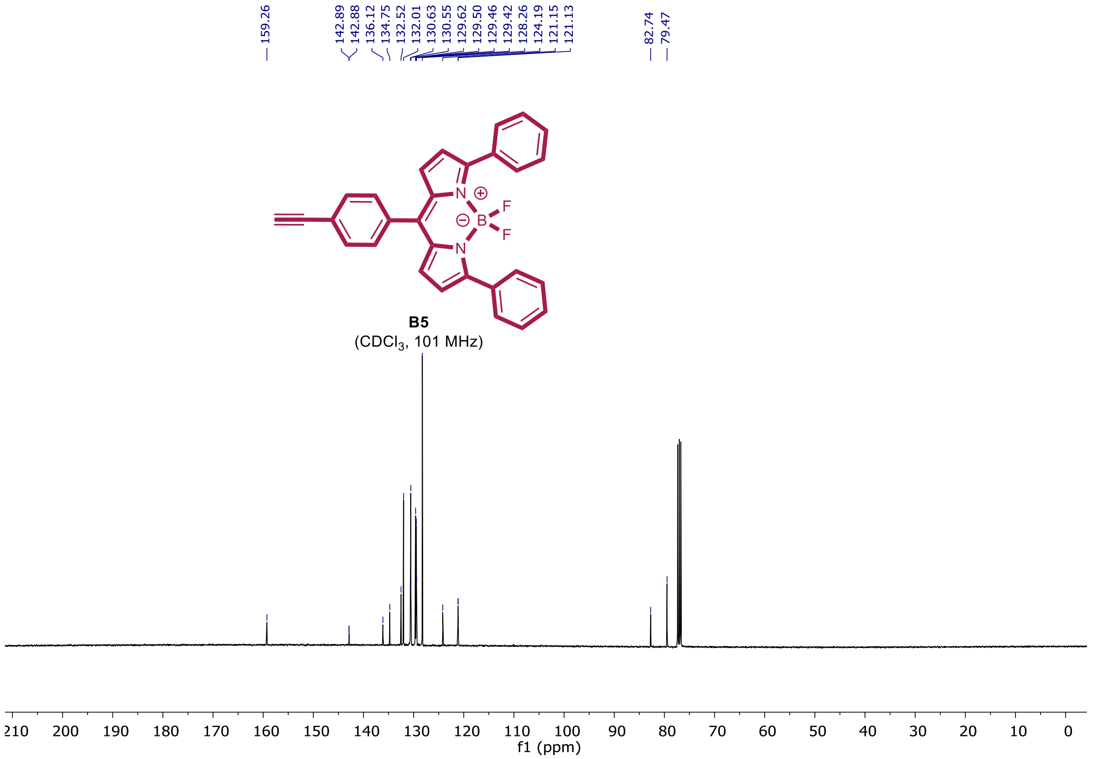

Supplementary Figure 23. ^{13}C-NMR spectrum of **B5**.

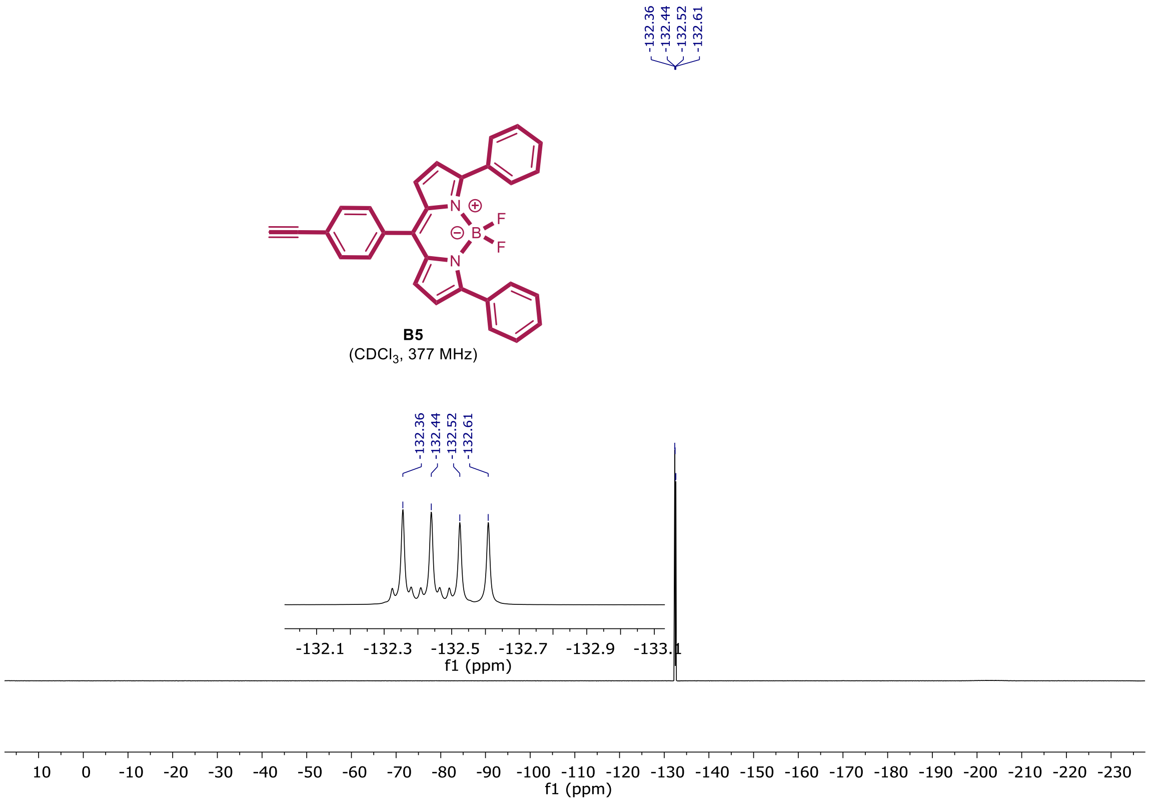

Supplementary Figure 24. ^{19}F-NMR spectrum of **B5**.

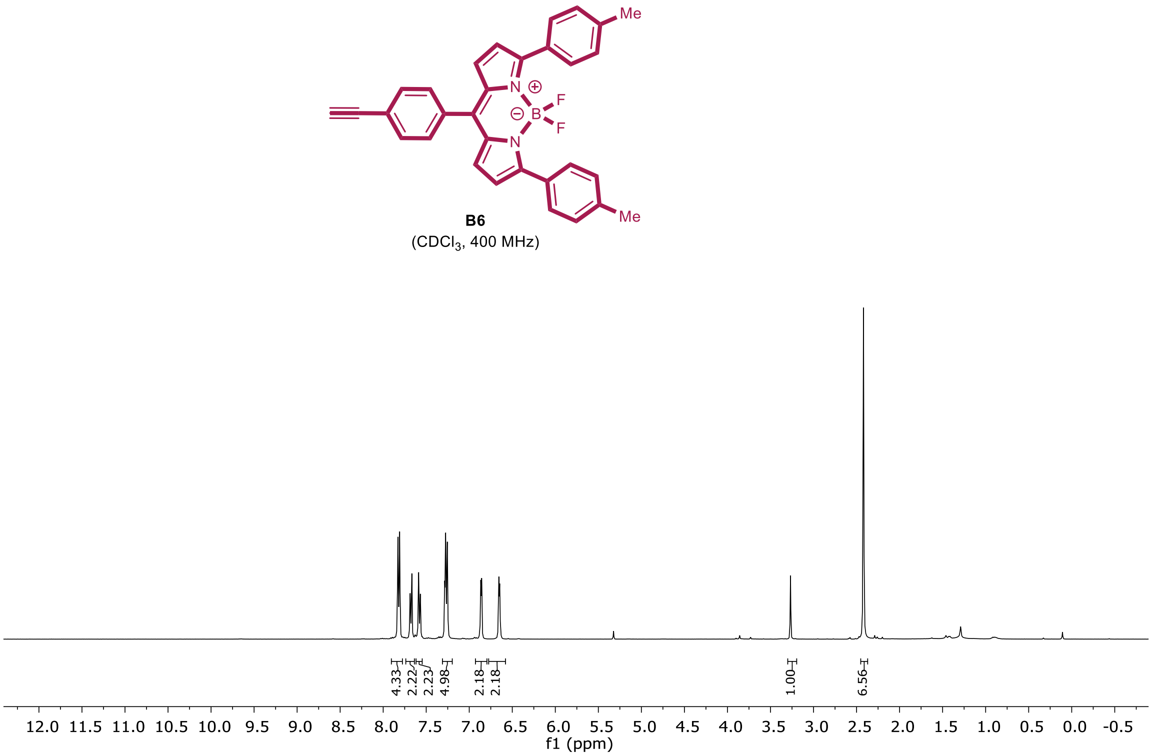

Supplementary Figure 25. ^{1}H-NMR spectrum of **B6**.

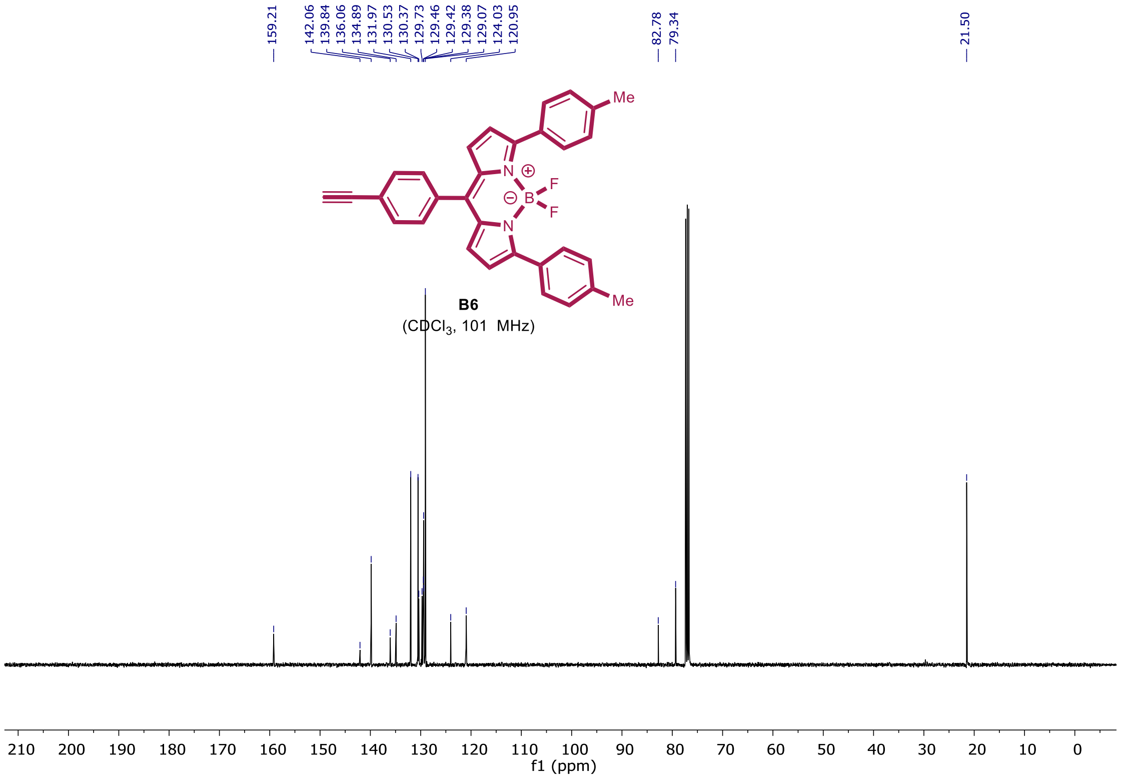

Supplementary Figure 26. ^{13}C-NMR spectrum of **B6**.

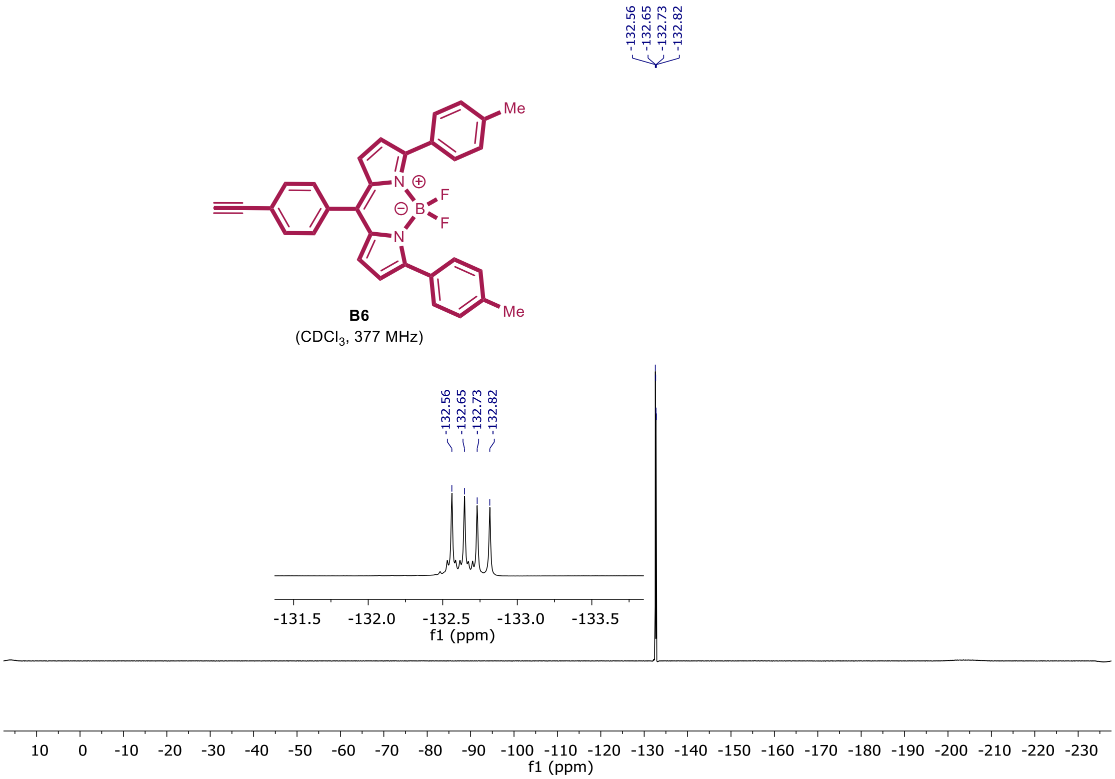

Supplementary Figure 27. ^{19}F-NMR spectrum of **B6**.

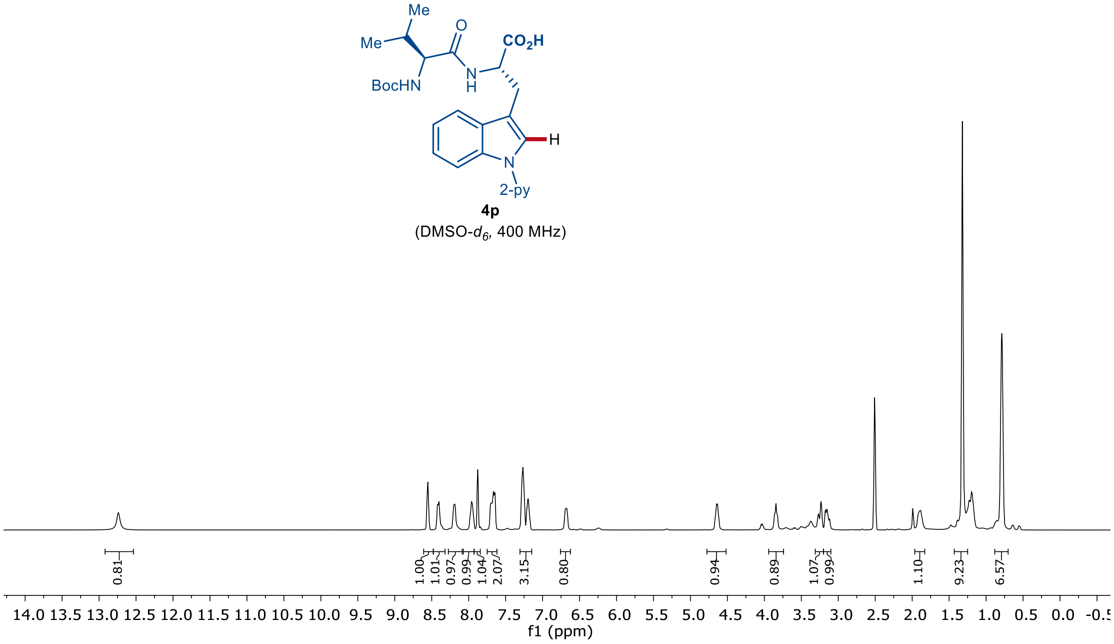

Supplementary Figure 28. ^{1}H-NMR spectrum of **4p**.

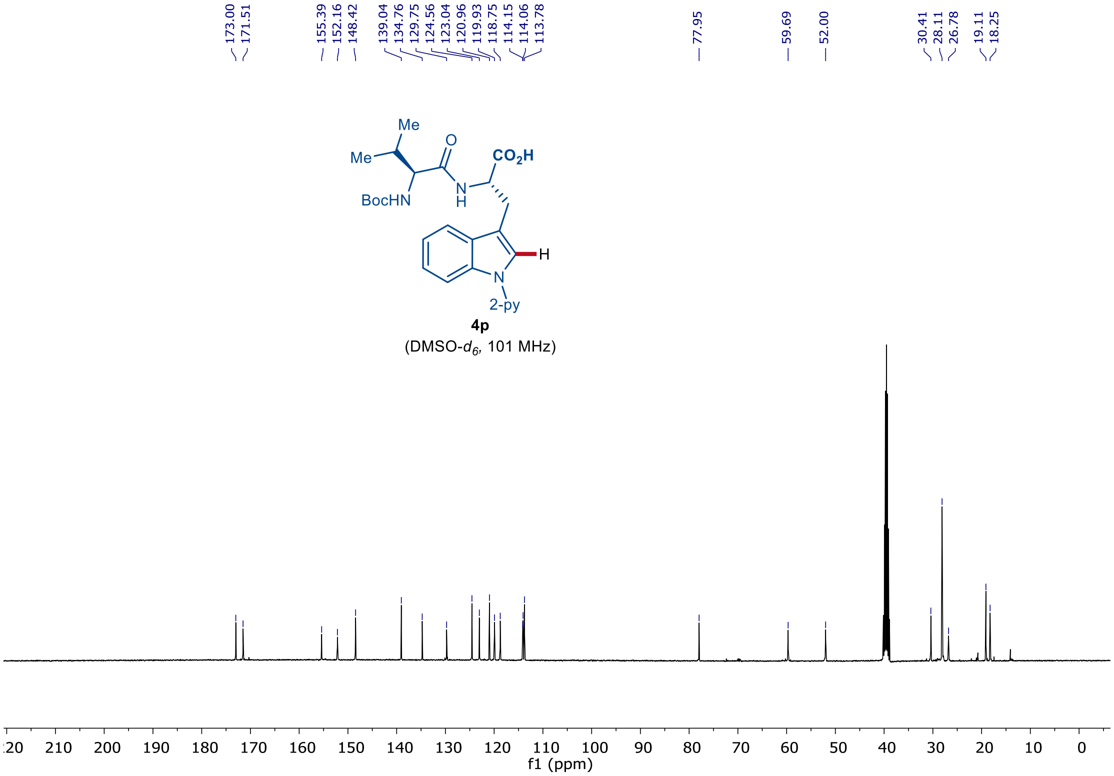

Supplementary Figure 29. ^{13}C-NMR spectrum of **4p**.

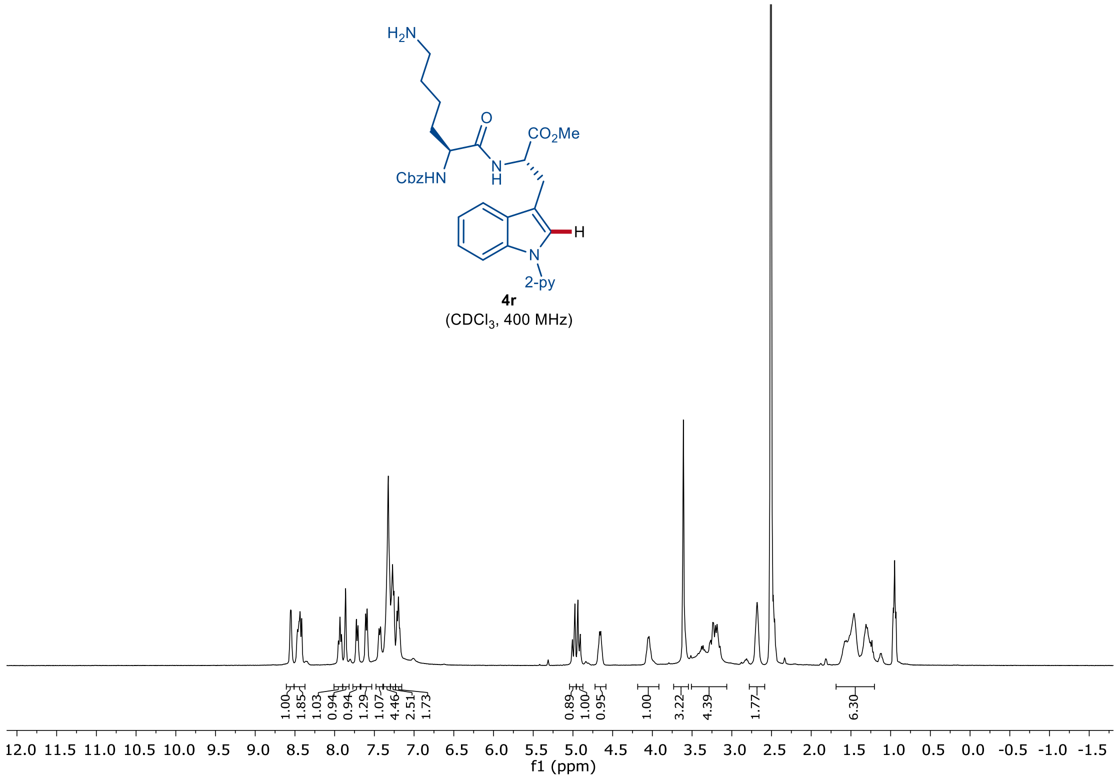

Supplementary Figure 30. ^{1}H-NMR spectrum of **4r**.

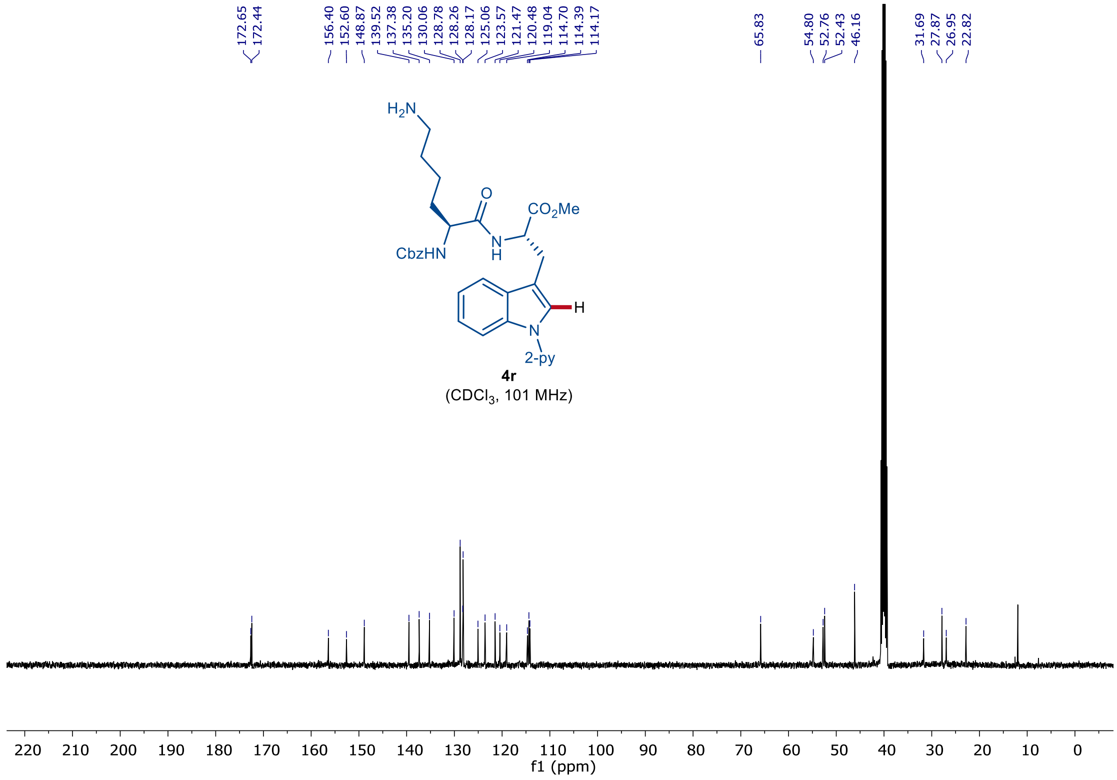

Supplementary Figure 31. ^{13}C-NMR spectrum of **4r**.

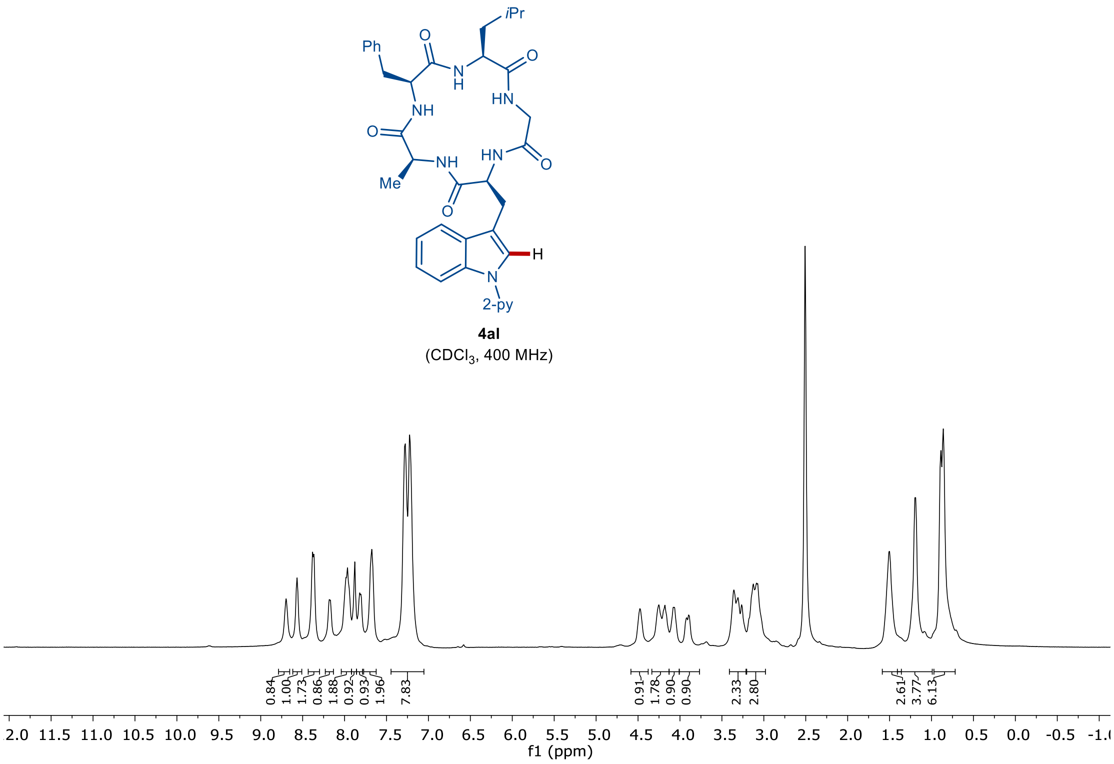

Supplementary Figure 32. ^{1}H-NMR spectrum of **4al**.

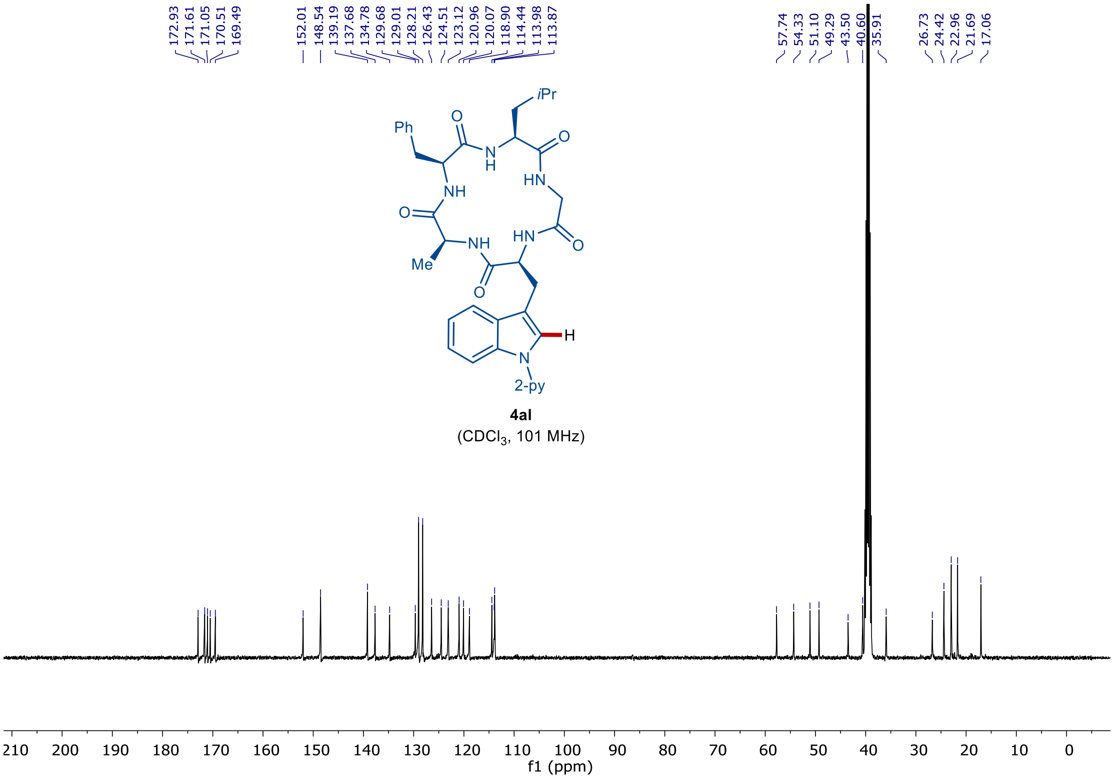

Supplementary Figure 33. ^{13}C-NMR spectrum of **4al**

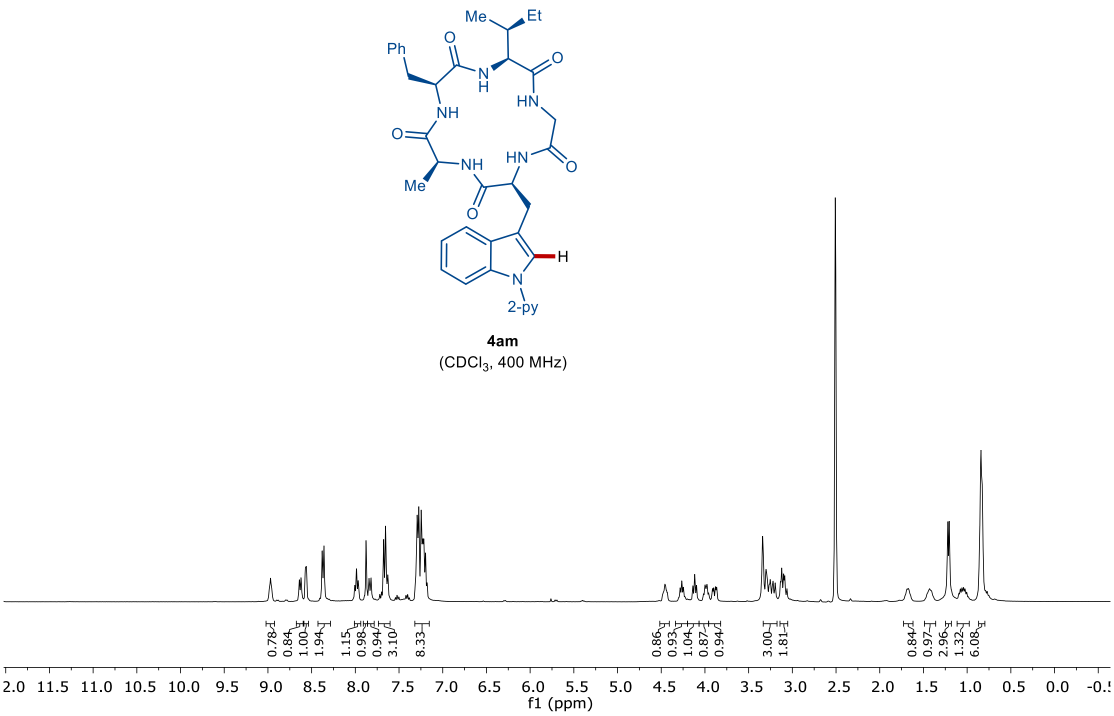

Supplementary Figure 34. ^{1}H-NMR spectrum of **4am**.

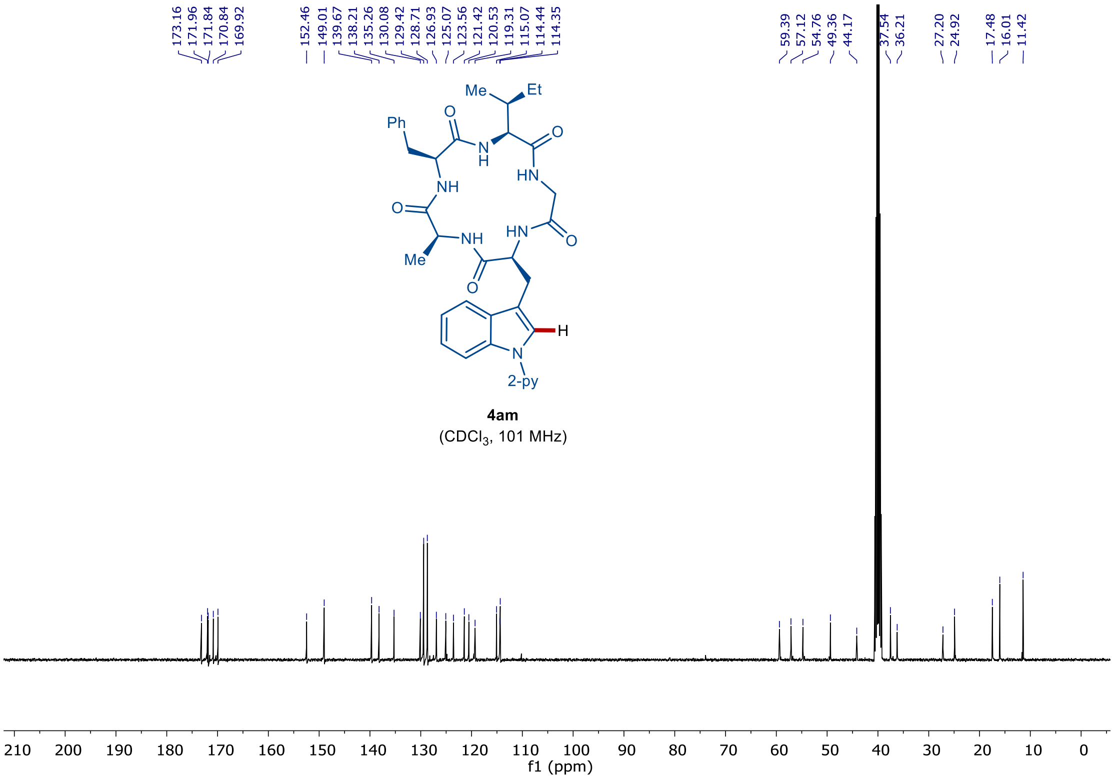

Supplementary Figure 35. ^{13}C-NMR spectrum of **4am**.

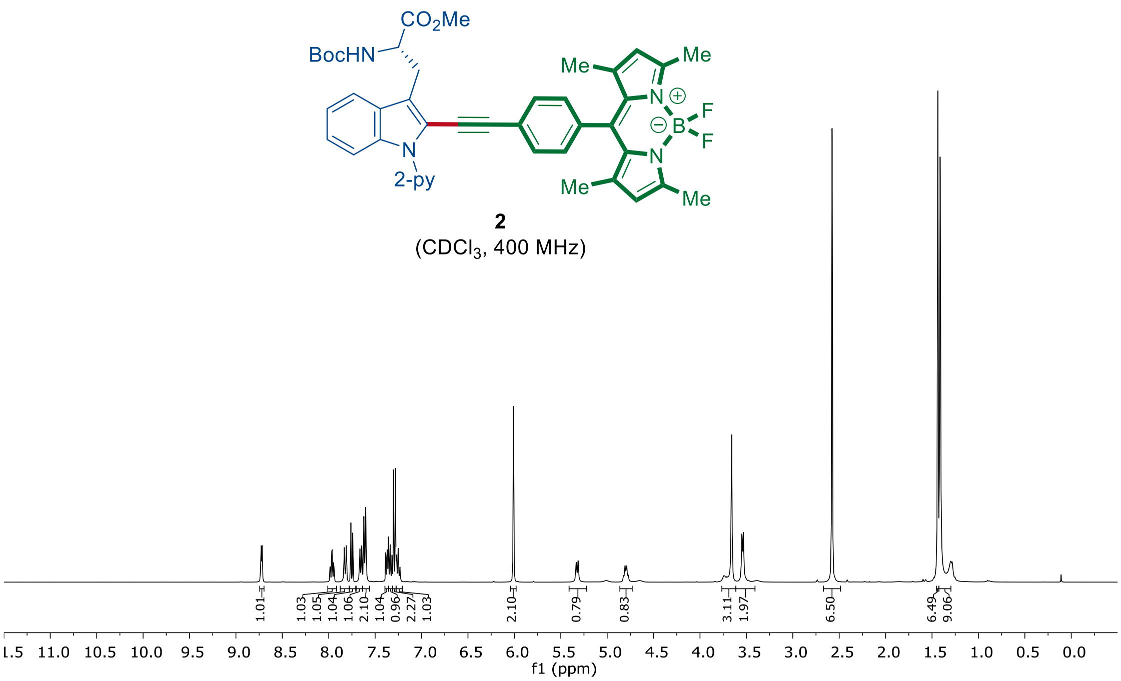

Supplementary Figure 36. ^{1}H-NMR spectrum of **2**.

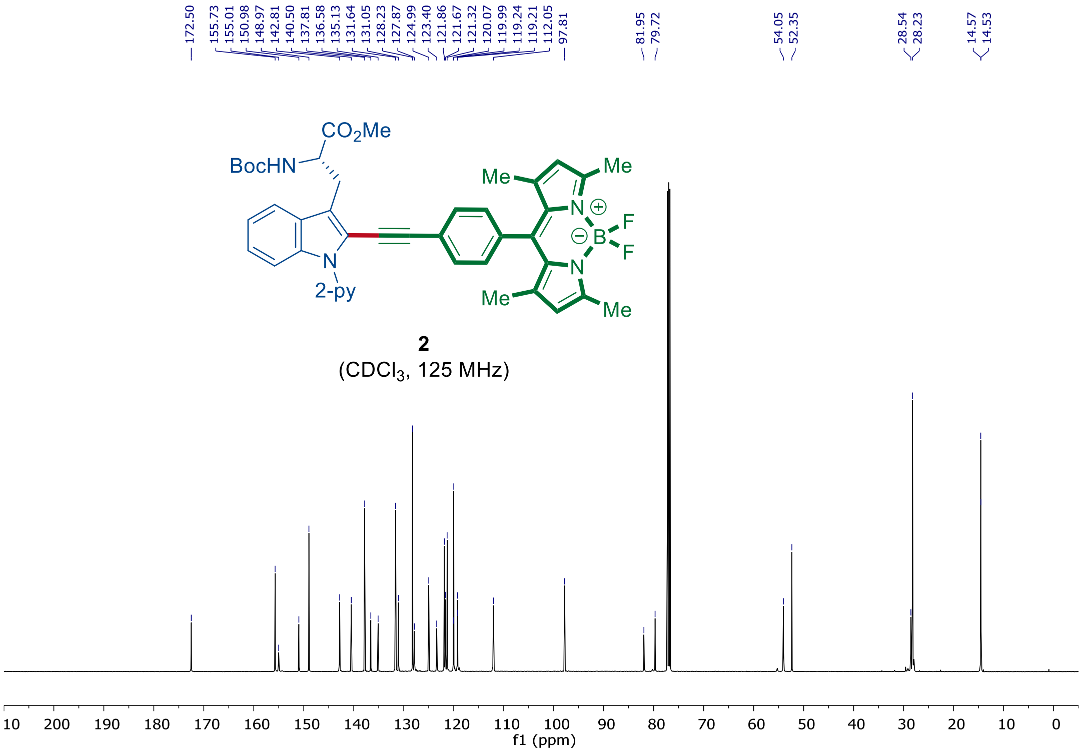

Supplementary Figure 37. ^{13}C-NMR spectrum of **2**.

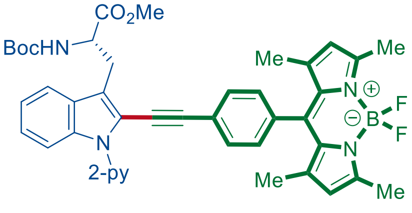

2
($CDCl_3$, 283 MHz)

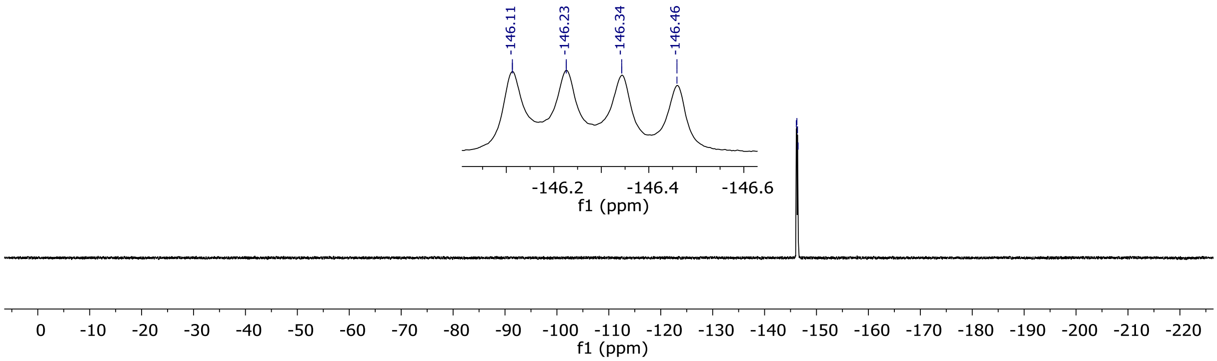

Supplementary Figure 38. ^{19}F-NMR spectrum of **2**.

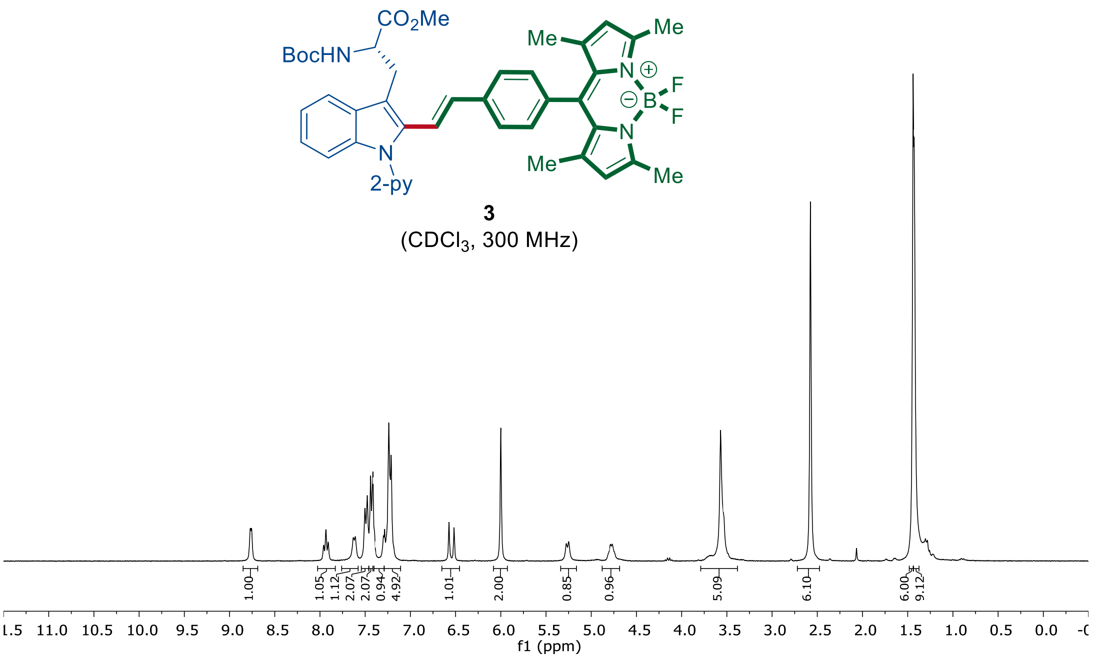

Supplementary Figure 39. ^{1}H-NMR spectrum of **3**.

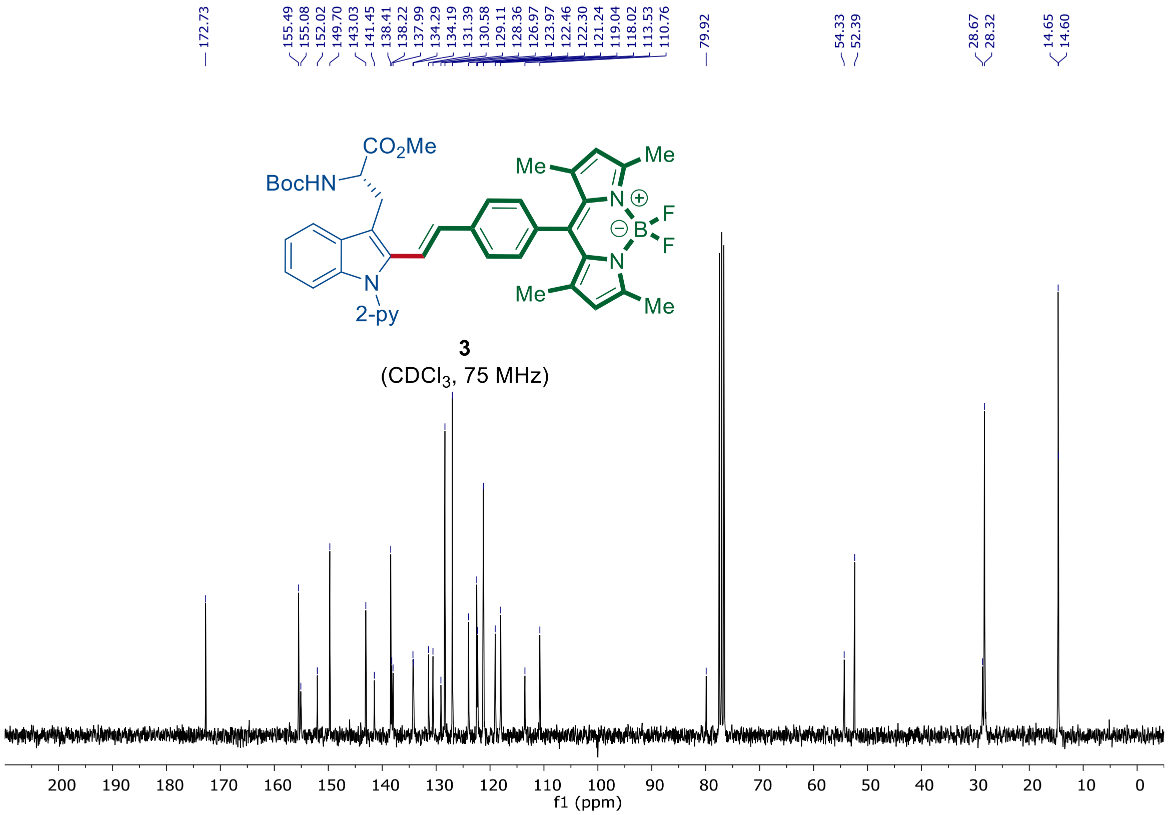

Supplementary Figure 40. ^{13}C-NMR spectrum of **3**.

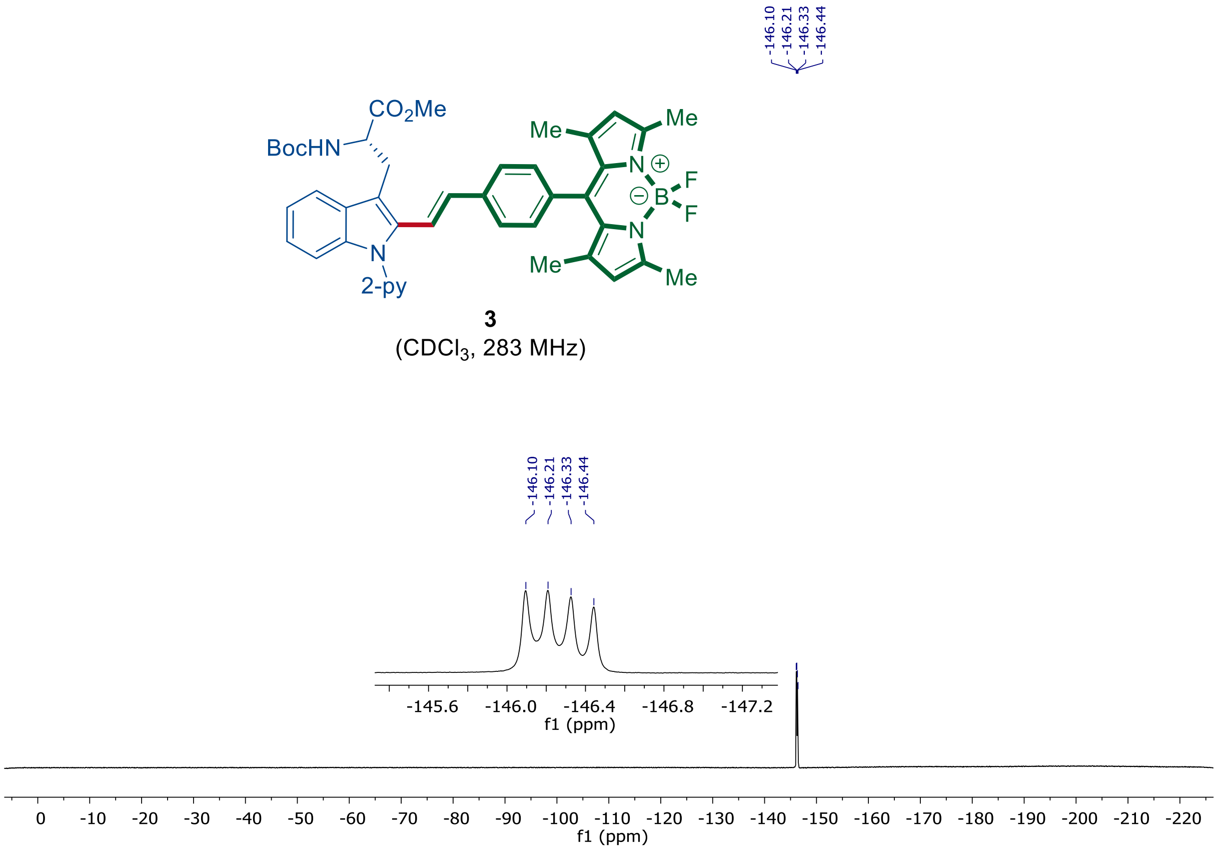

Supplementary Figure 41. ^{19}F-NMR spectrum of **3**.

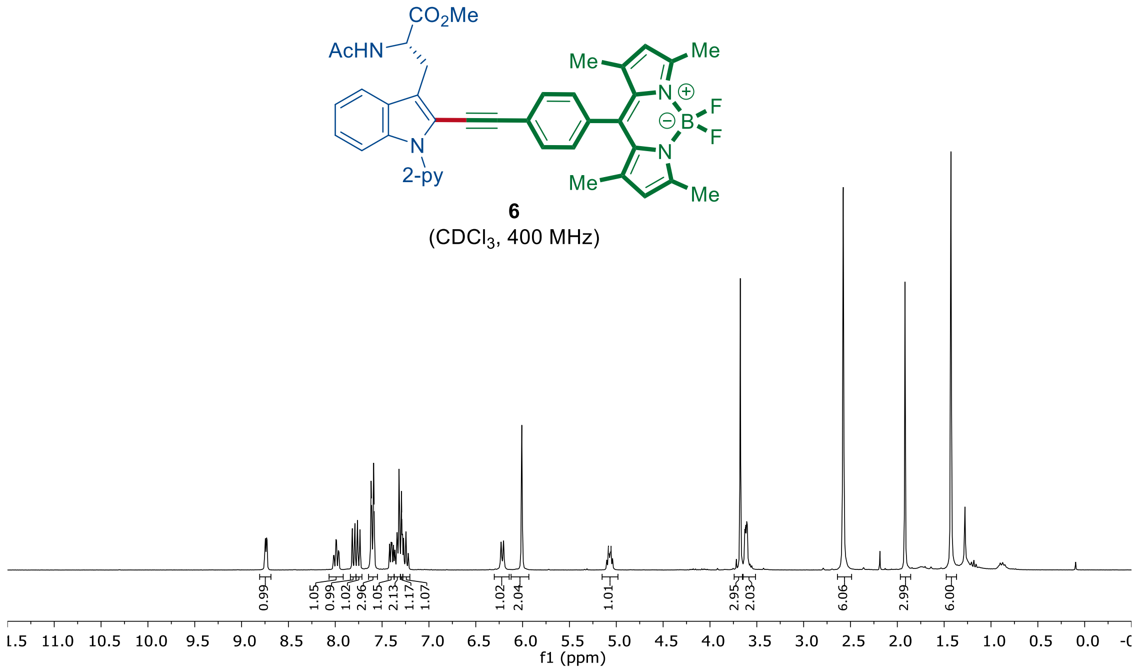

Supplementary Figure 42. ^{1}H-NMR spectrum of **6**.

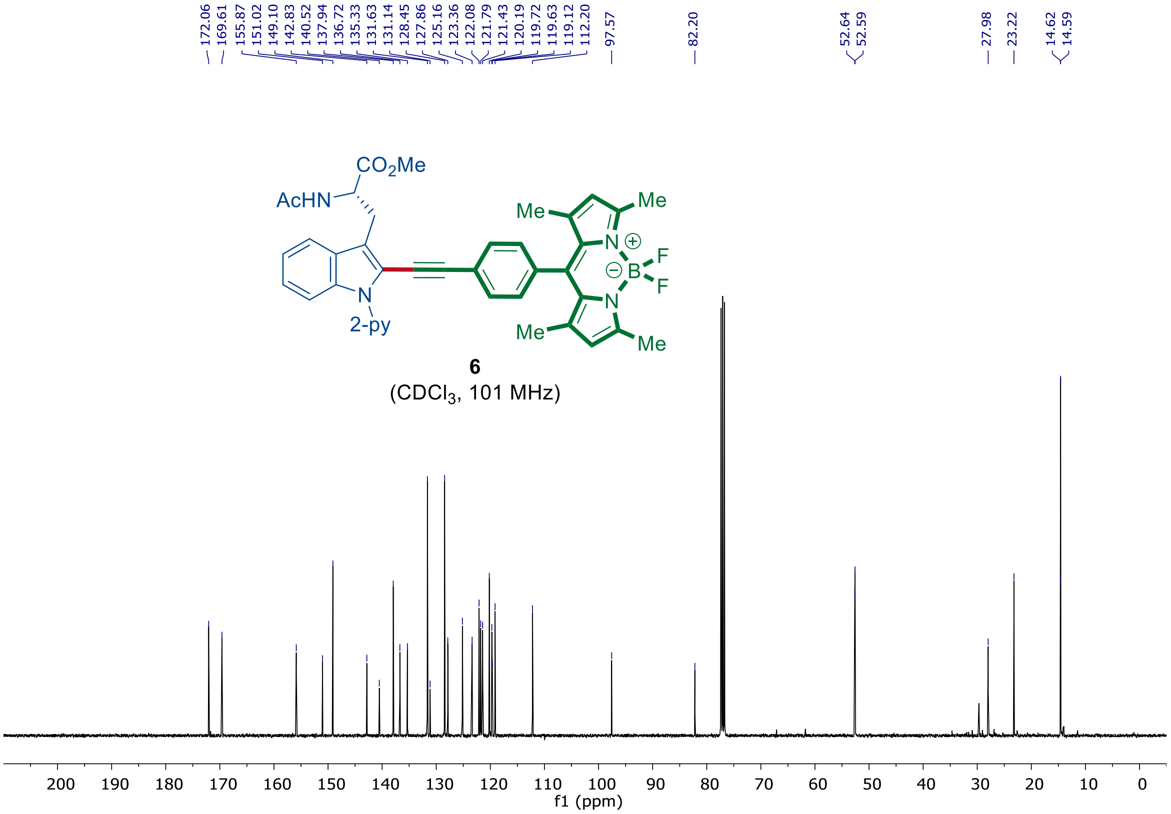

Supplementary Figure 43. ^{13}C-NMR spectrum of **6**.

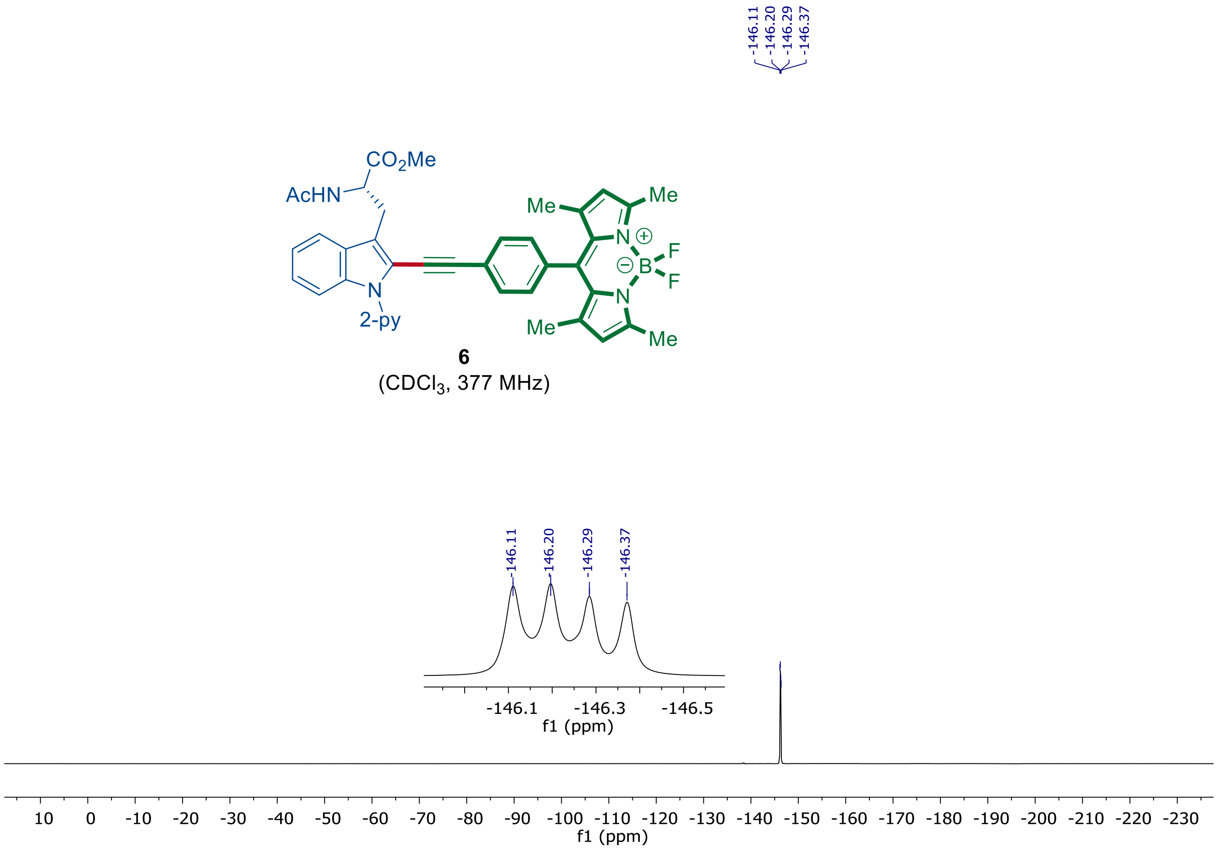

Supplementary Figure 44. ^{19}F-NMR spectrum of **6**.

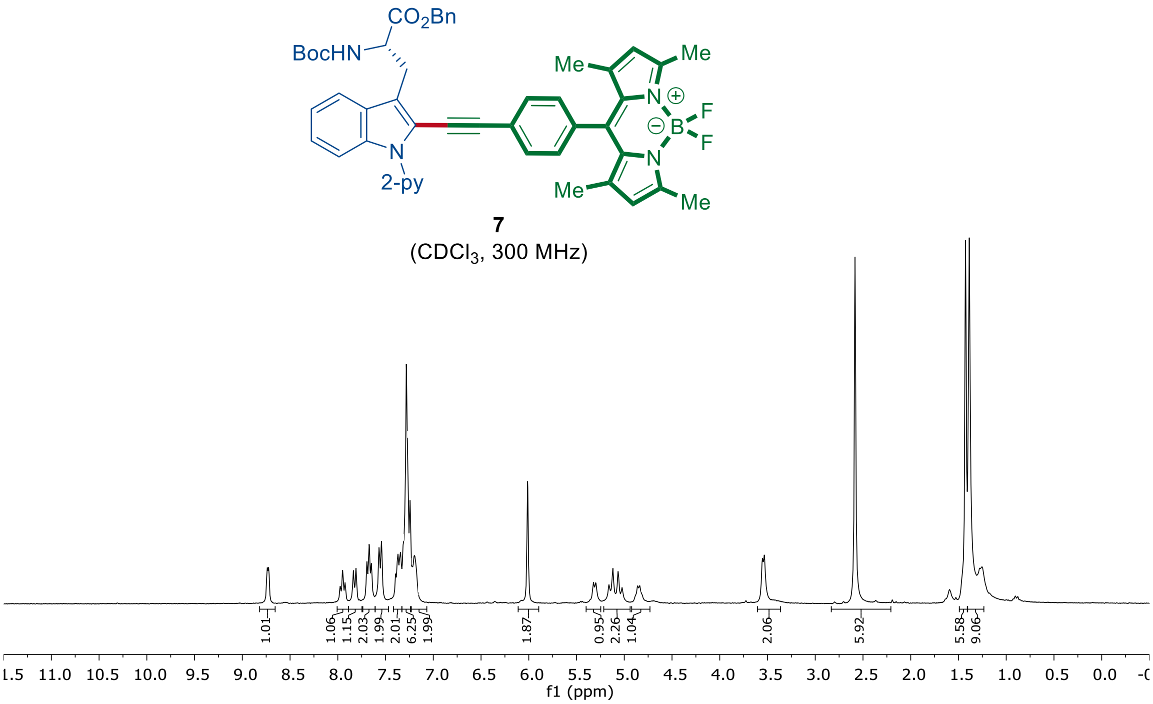

Supplementary Figure 45. ^{1}H-NMR spectrum of **7**.

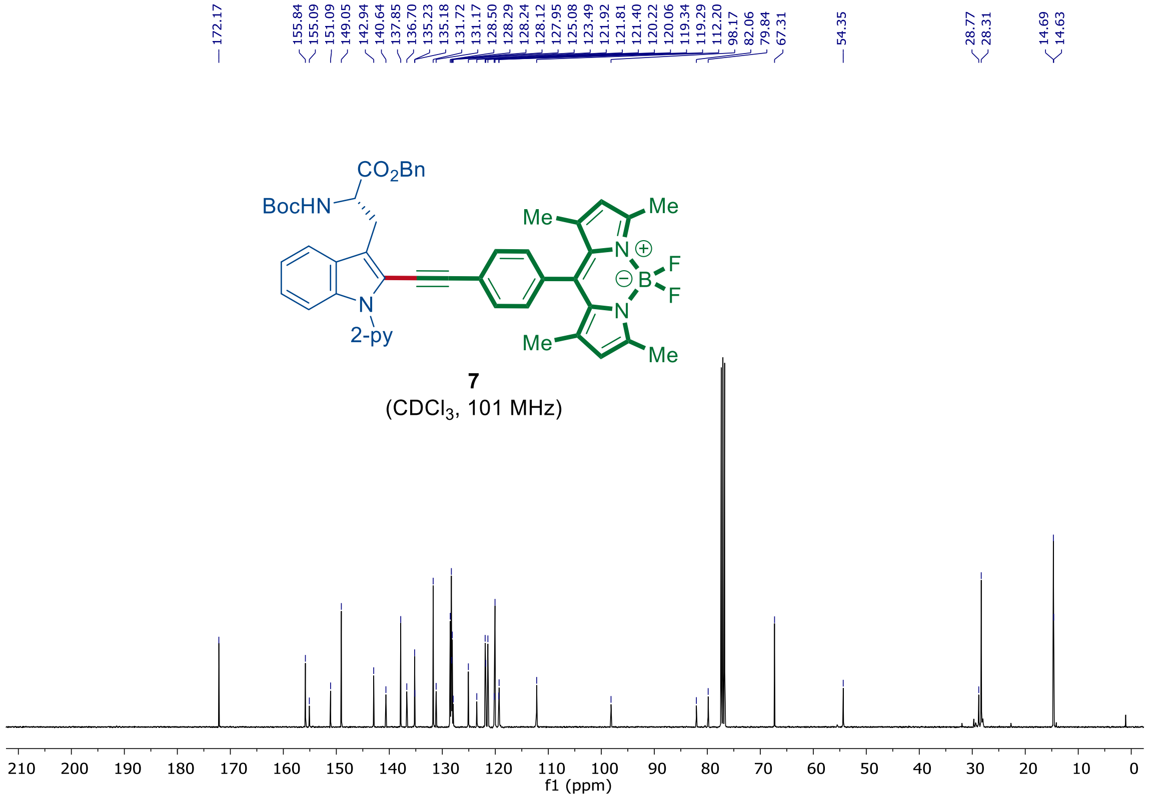

Supplementary Figure 46. ^{13}C-NMR spectrum of **7**.

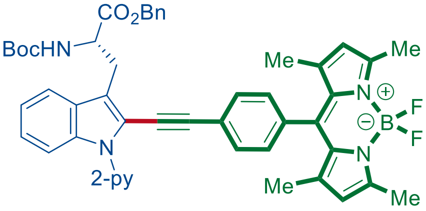

7
($CDCl_3$, 377 MHz)

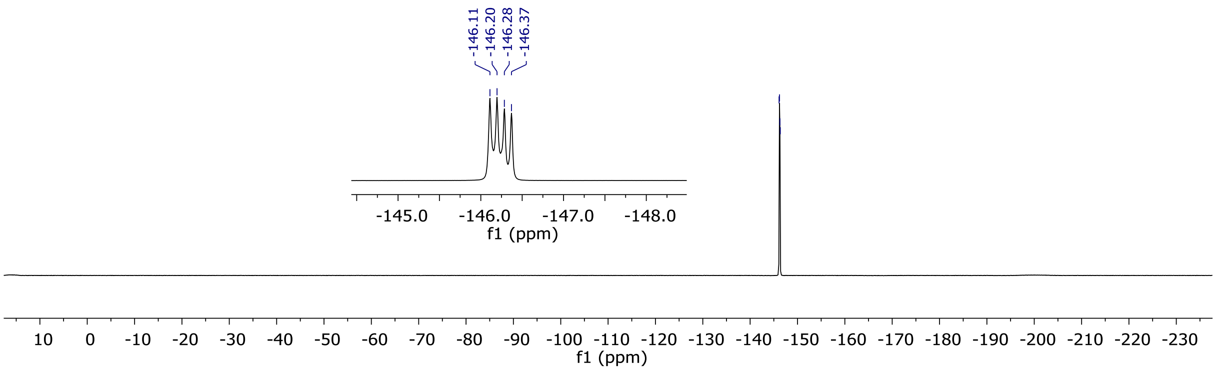

Supplementary Figure 47. ^{19}F-NMR spectrum of **7**.

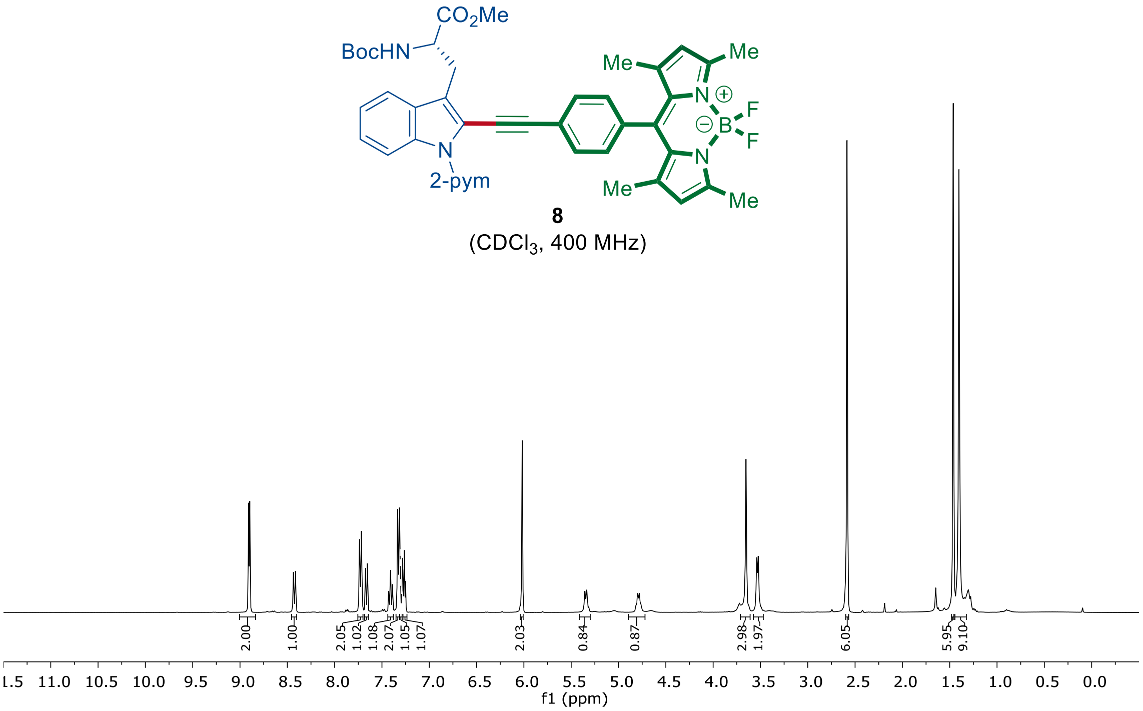

Supplementary Figure 48. ^{1}H-NMR spectrum of **8**.

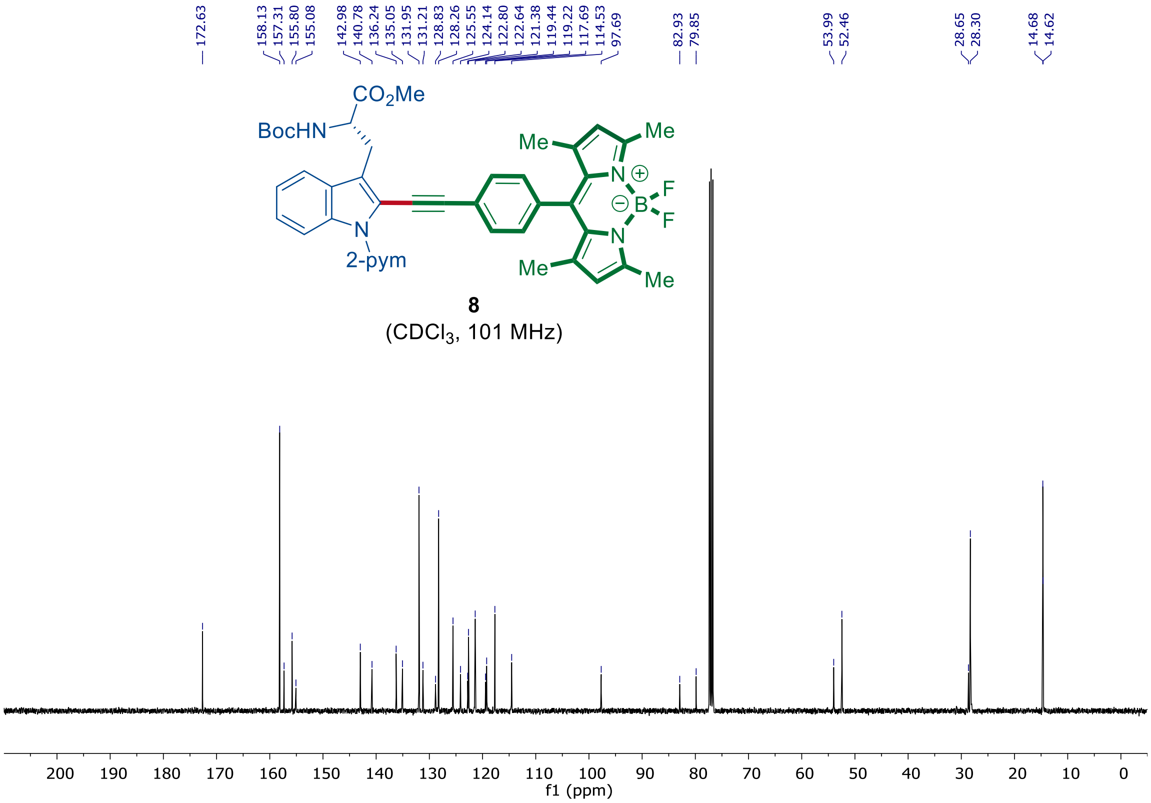

Supplementary Figure 49. ^{13}C-NMR spectrum of **8**.

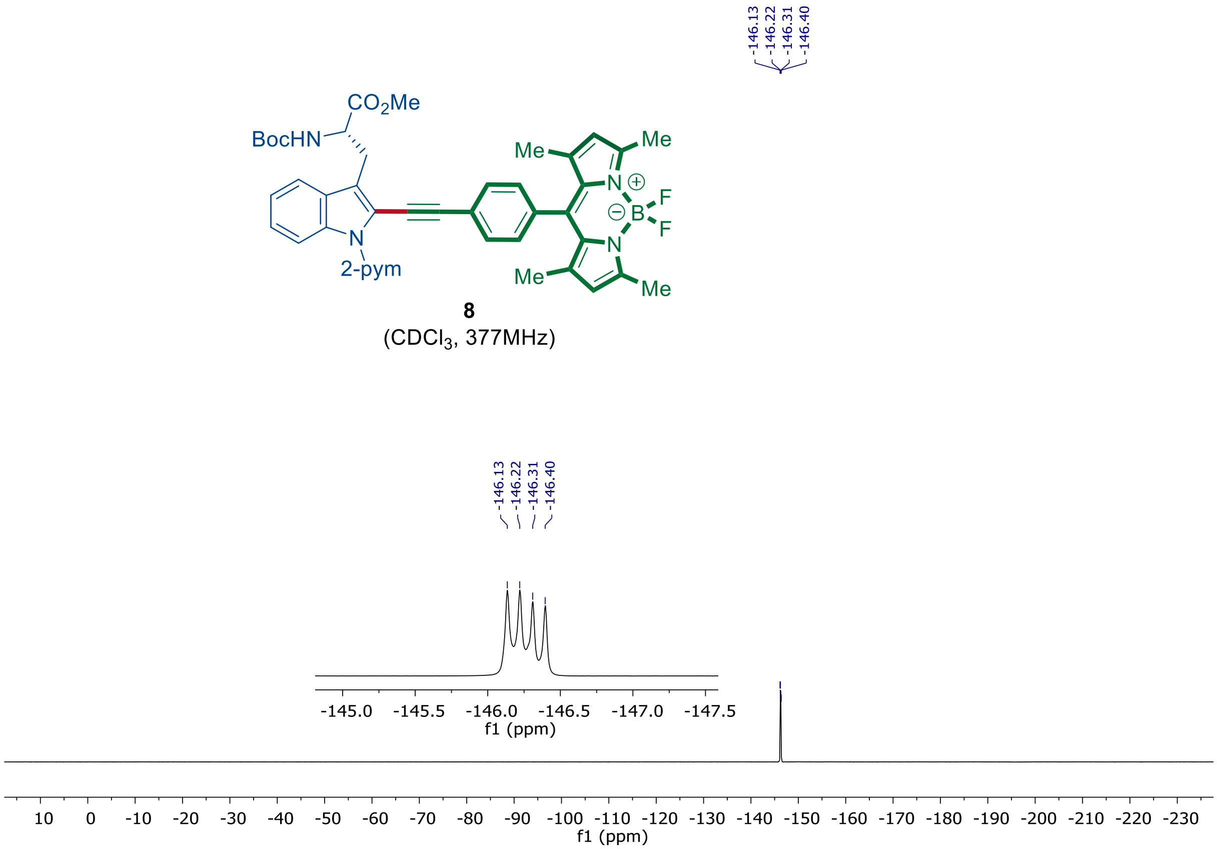

Supplementary Figure 50. ^{19}F-NMR spectrum of **8**.

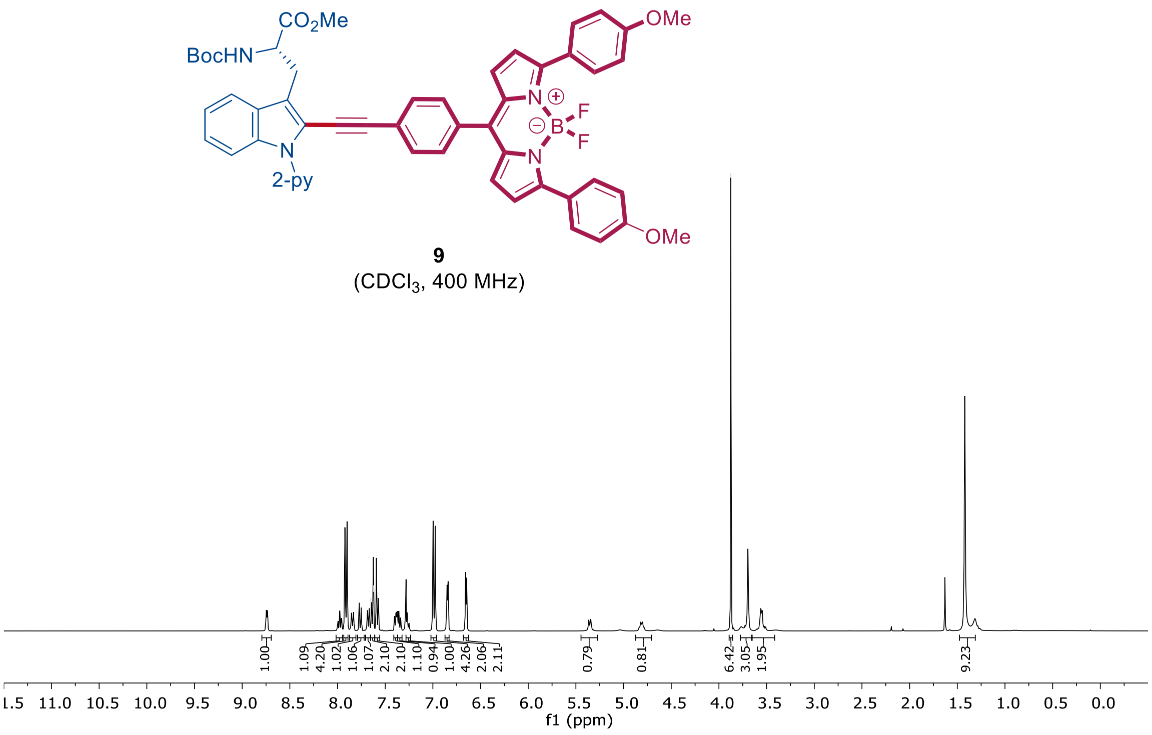

Supplementary Figure 51. ^{1}H-NMR spectrum of **9**.

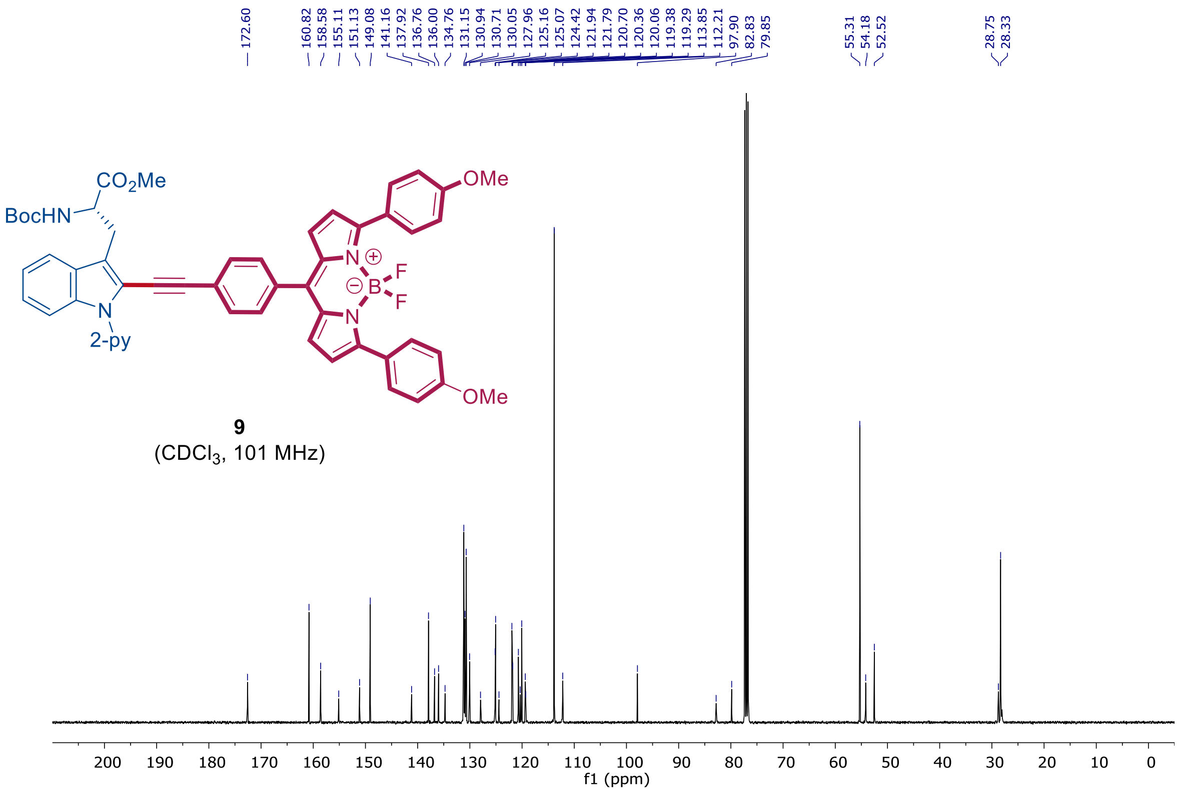

Supplementary Figure 52. ^{13}C-NMR spectrum of **9**.

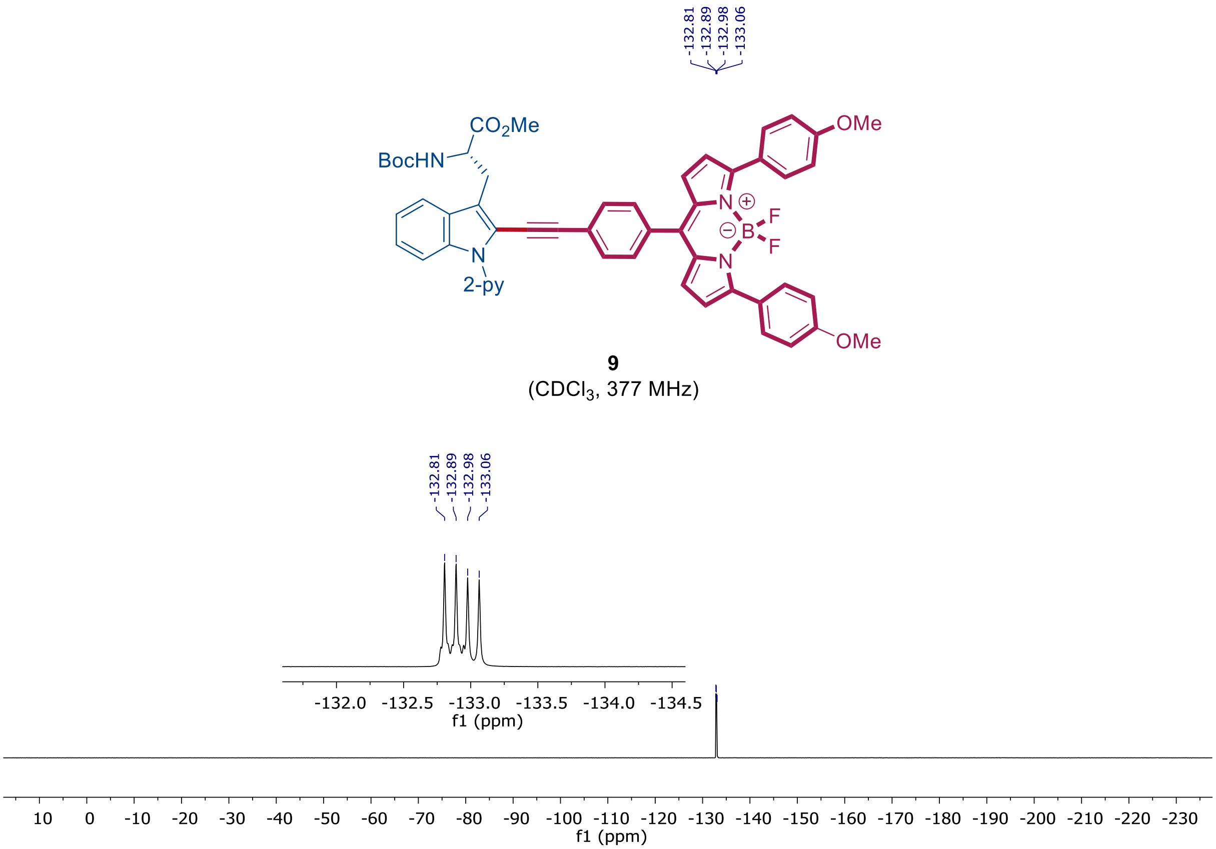

Supplementary Figure 53. ^{19}F-NMR spectrum of **9**.

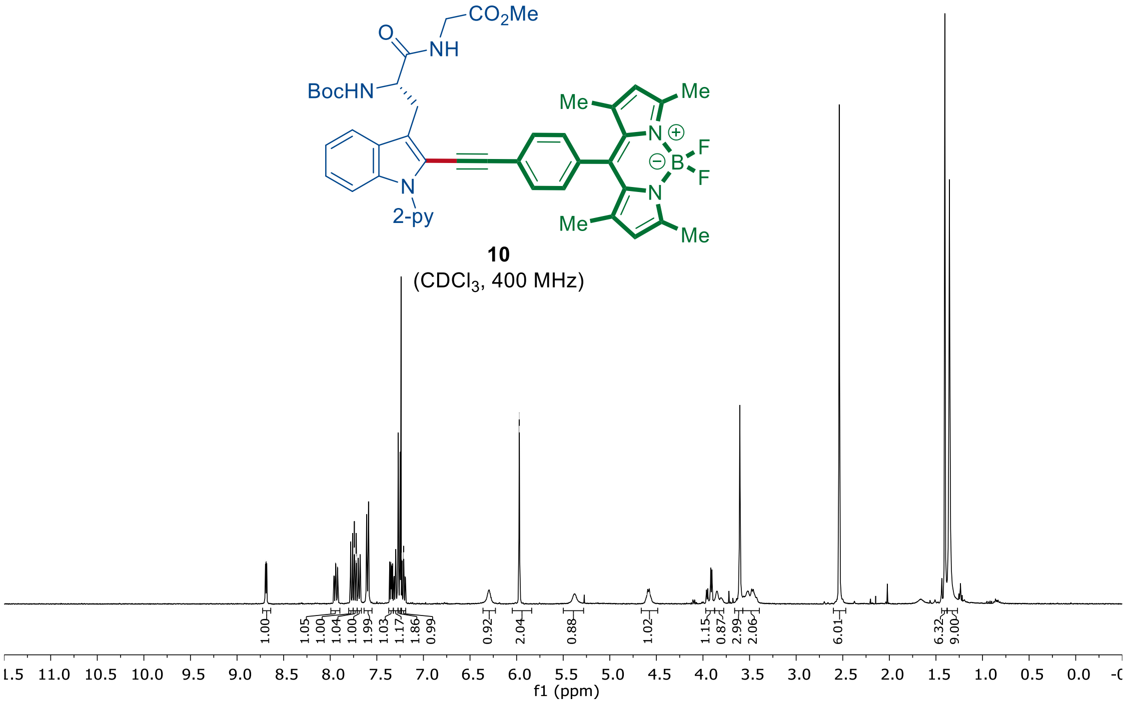

Supplementary Figure 54. ^{1}H-NMR spectrum of **10**.

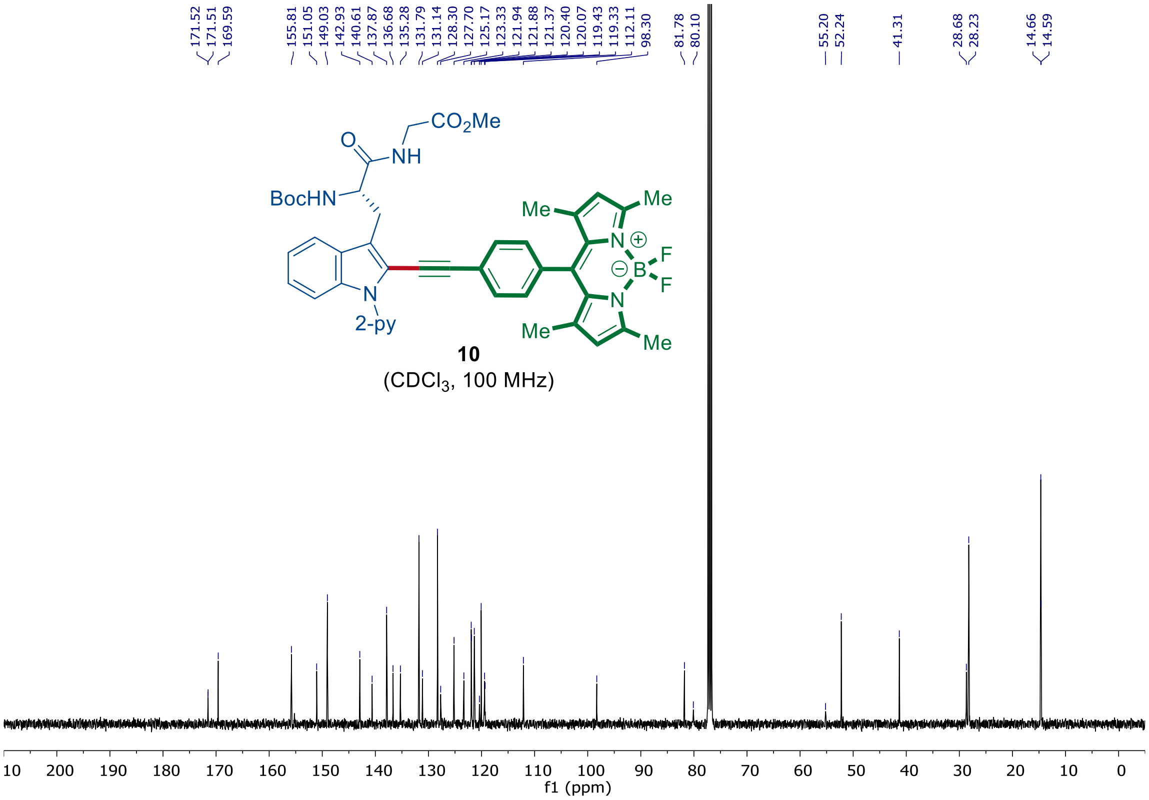

Supplementary Figure 55. ^{13}C-NMR spectrum of **10**.

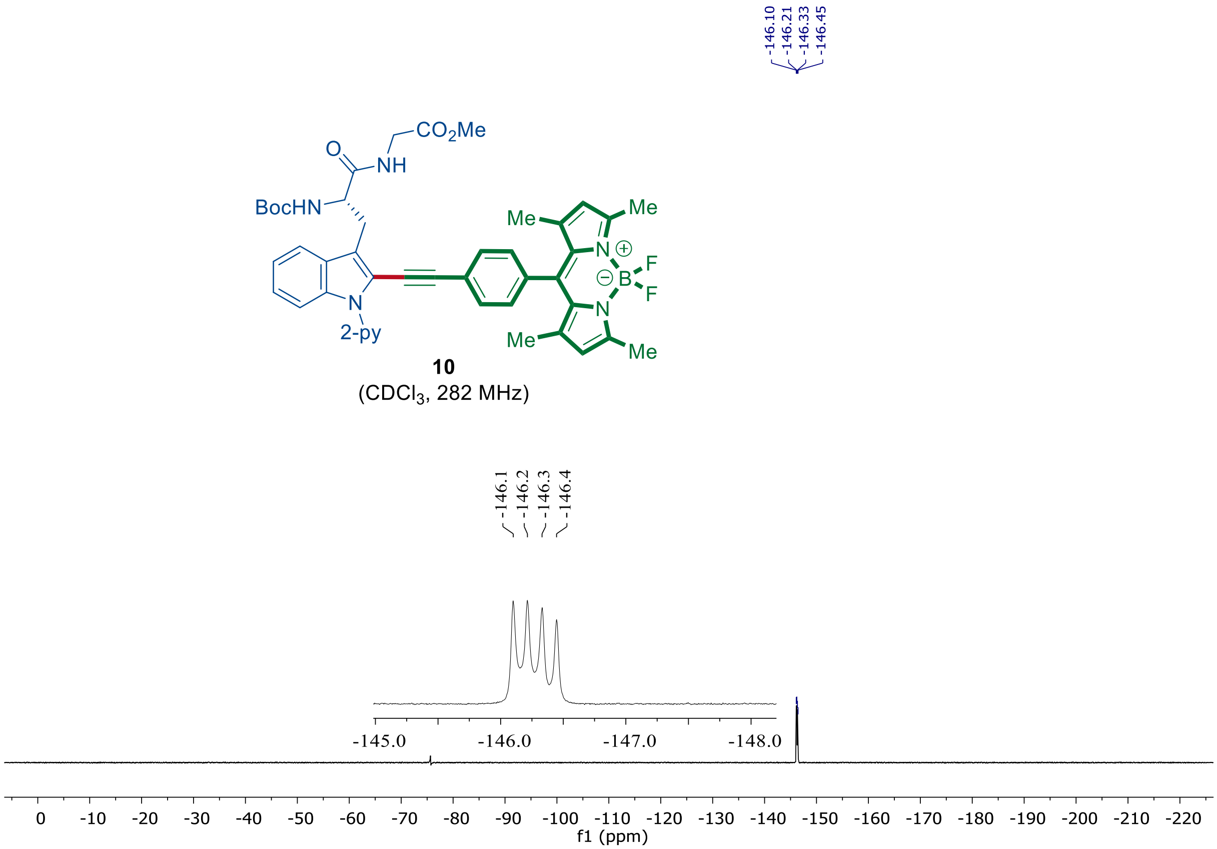

Supplementary Figure 56. ^{19}F-NMR spectrum of **10**.

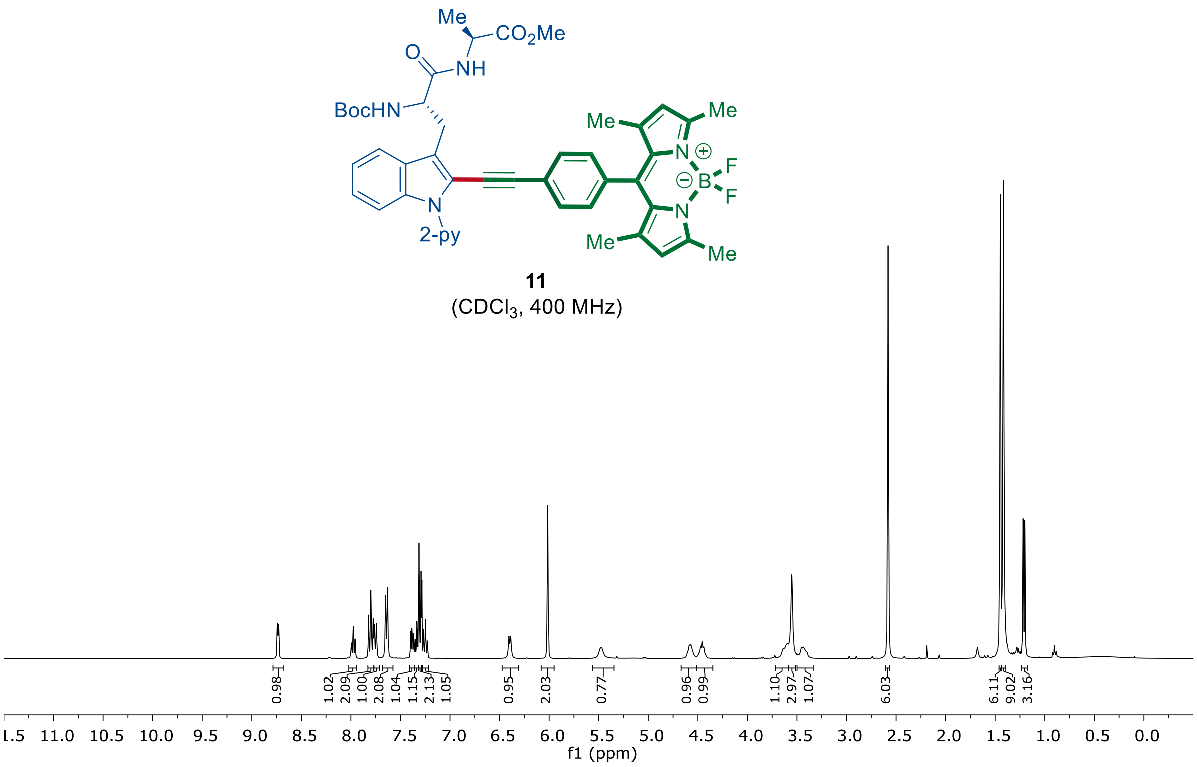

Supplementary Figure 57. ^{1}H-NMR spectrum of **11**.

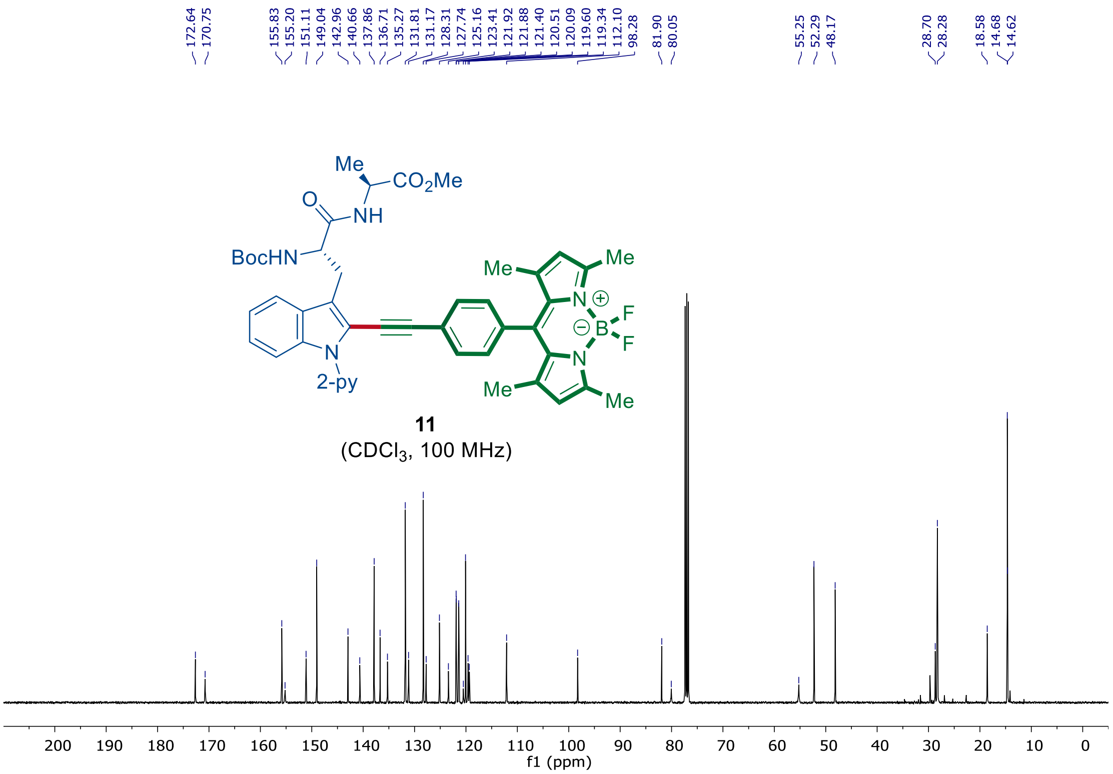

Supplementary Figure 58. ^{13}C-NMR spectrum of **11**.

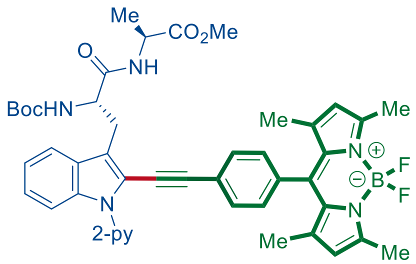

11
(CDCl$_3$, 377 MHz)

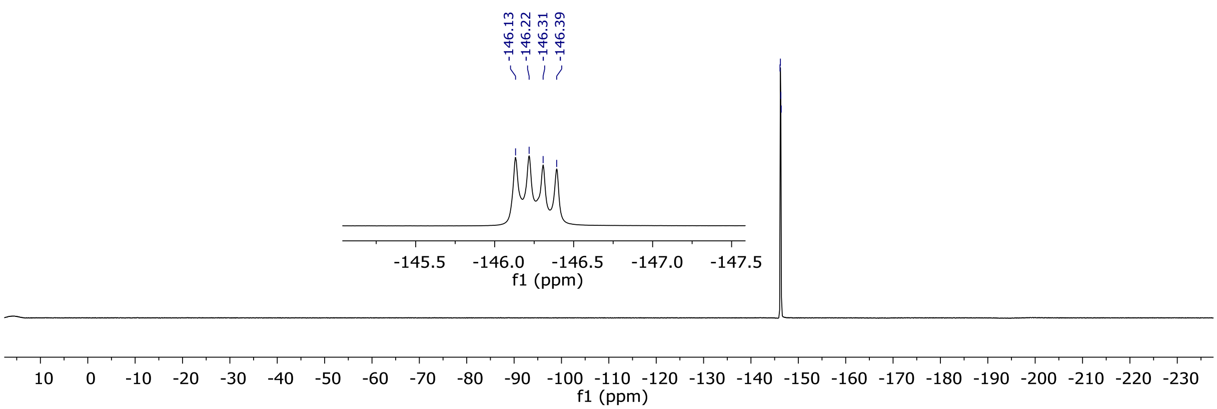

Supplementary Figure 59. ^{19}F-NMR spectrum of **11**.

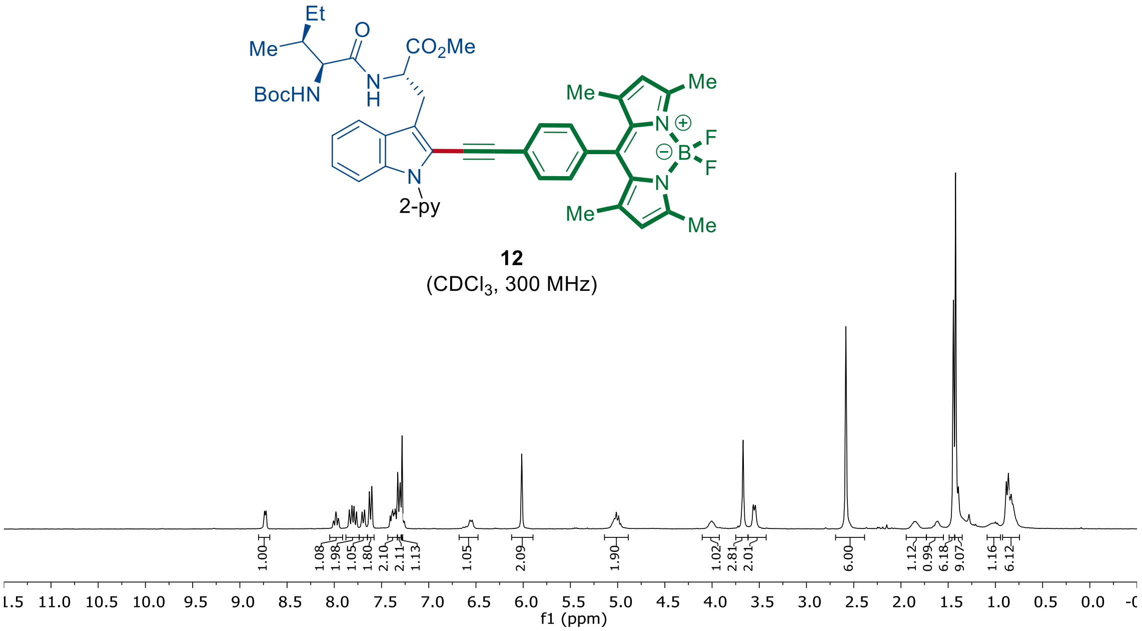

Supplementary Figure 60. ^{1}H-NMR spectrum of **12**.

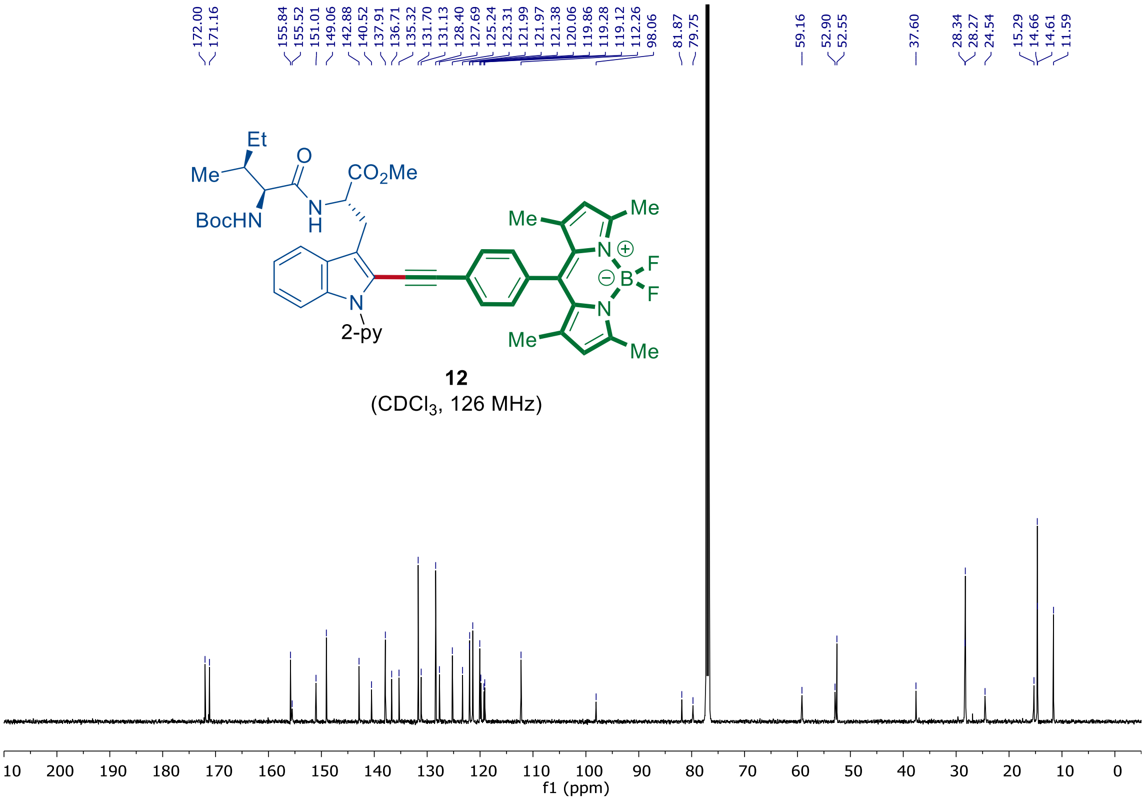

Supplementary Figure 61. ^{13}C-NMR spectrum of **12**.

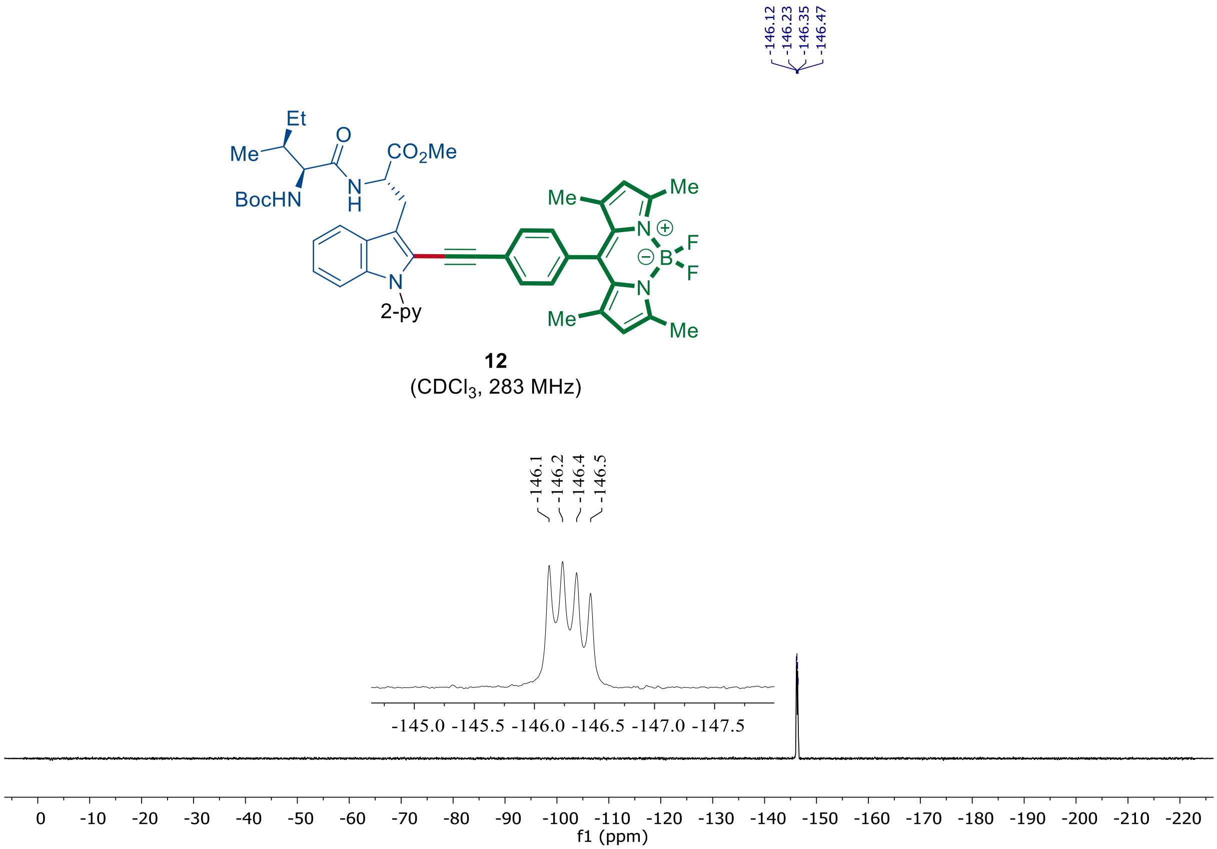

Supplementary Figure 62. ^{19}F-NMR spectrum of **12**.

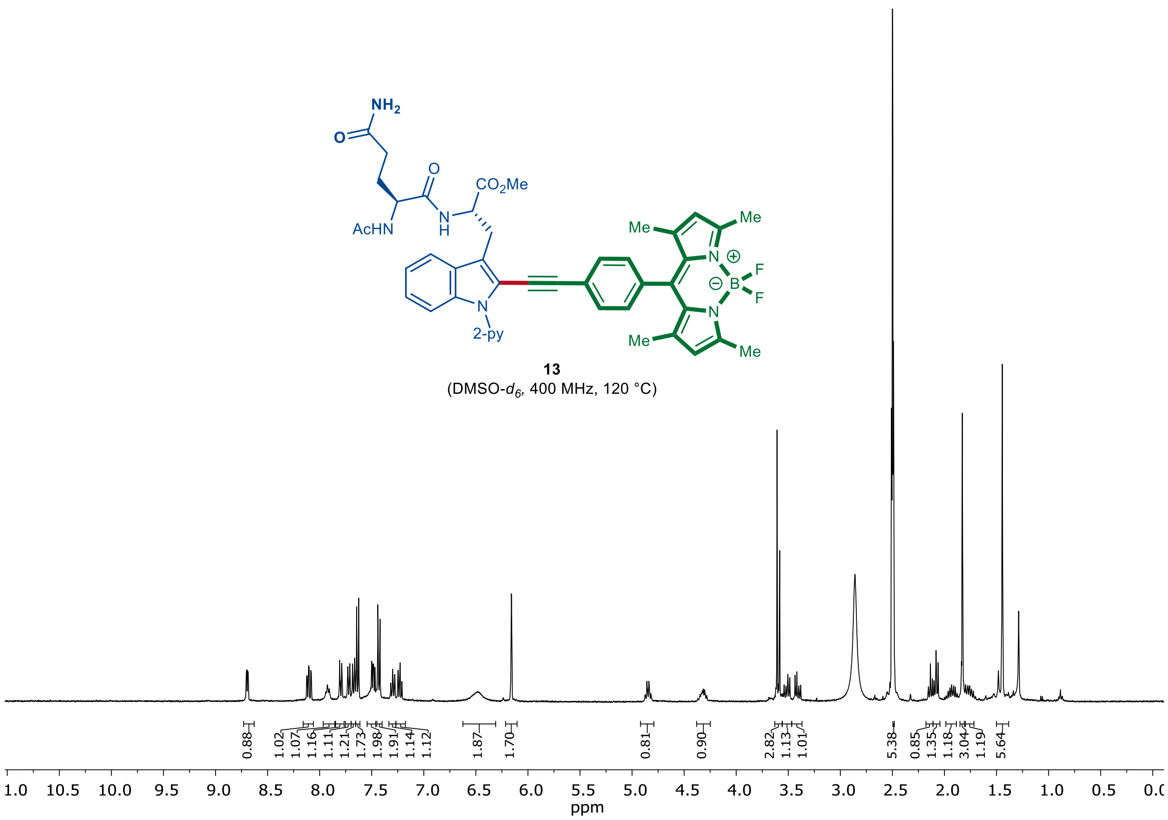

Supplementary Figure 63. ^{1}H-NMR spectrum of **13**.

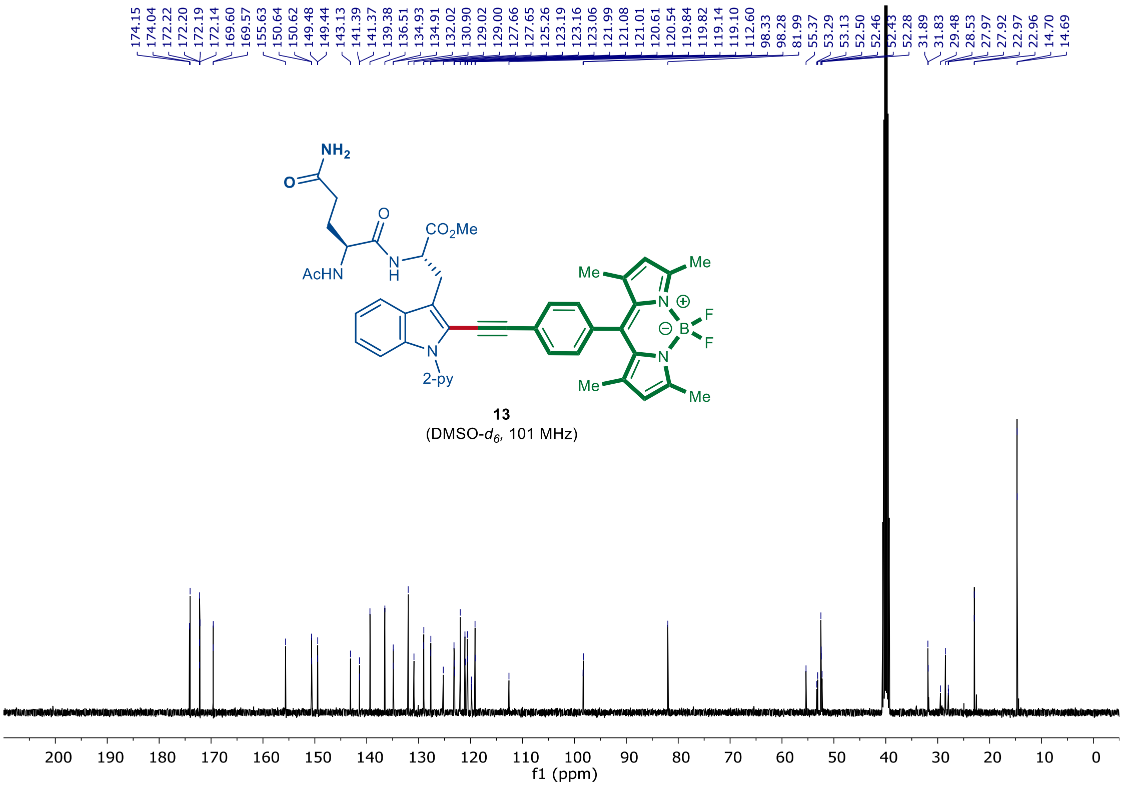

Supplementary Figure 64. ^{13}C-NMR spectrum of **13**.

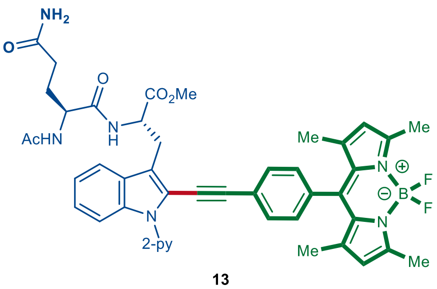

13
(DMSO-d_6, 377 MHz)

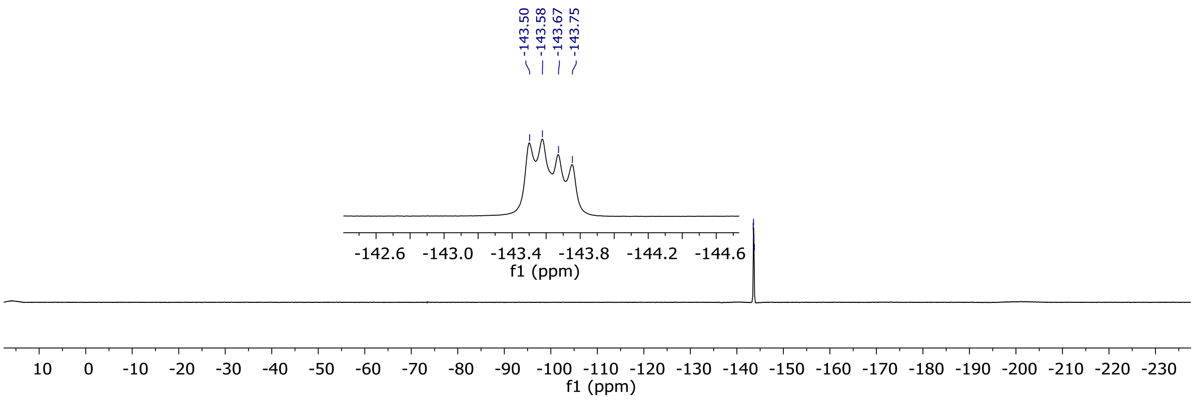

Supplementary Figure 65. ^{19}F-NMR spectrum of **13**.

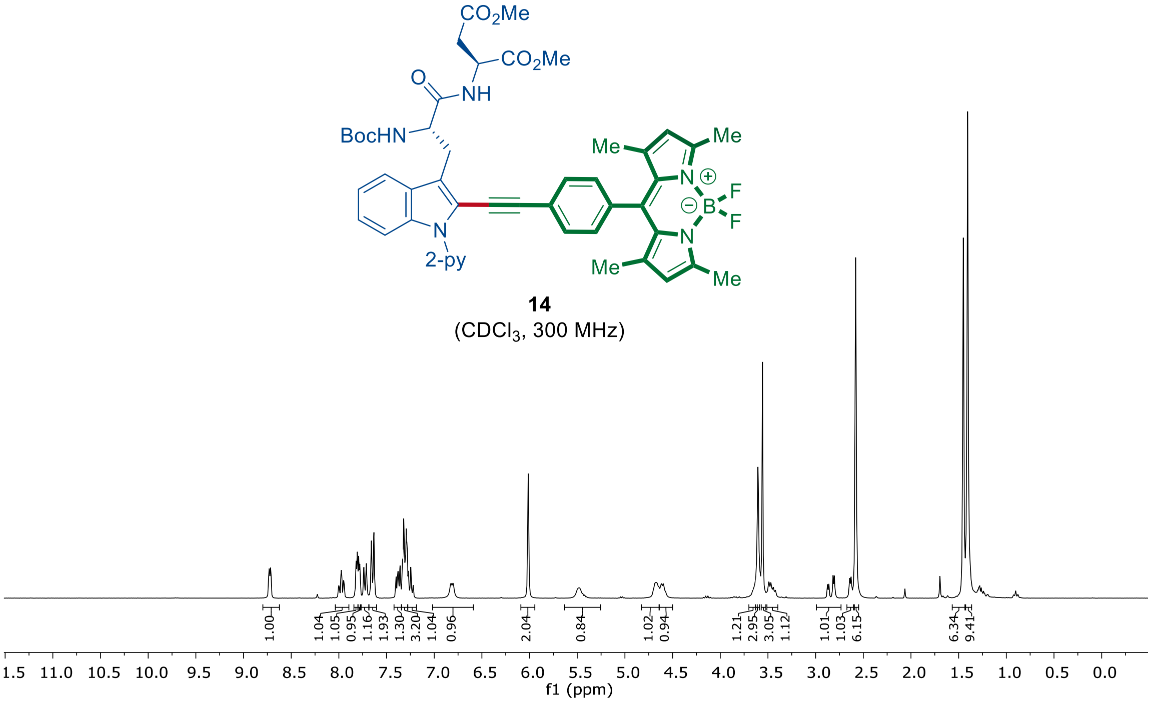

Supplementary Figure 66. ^{1}H-NMR spectrum of **14**.

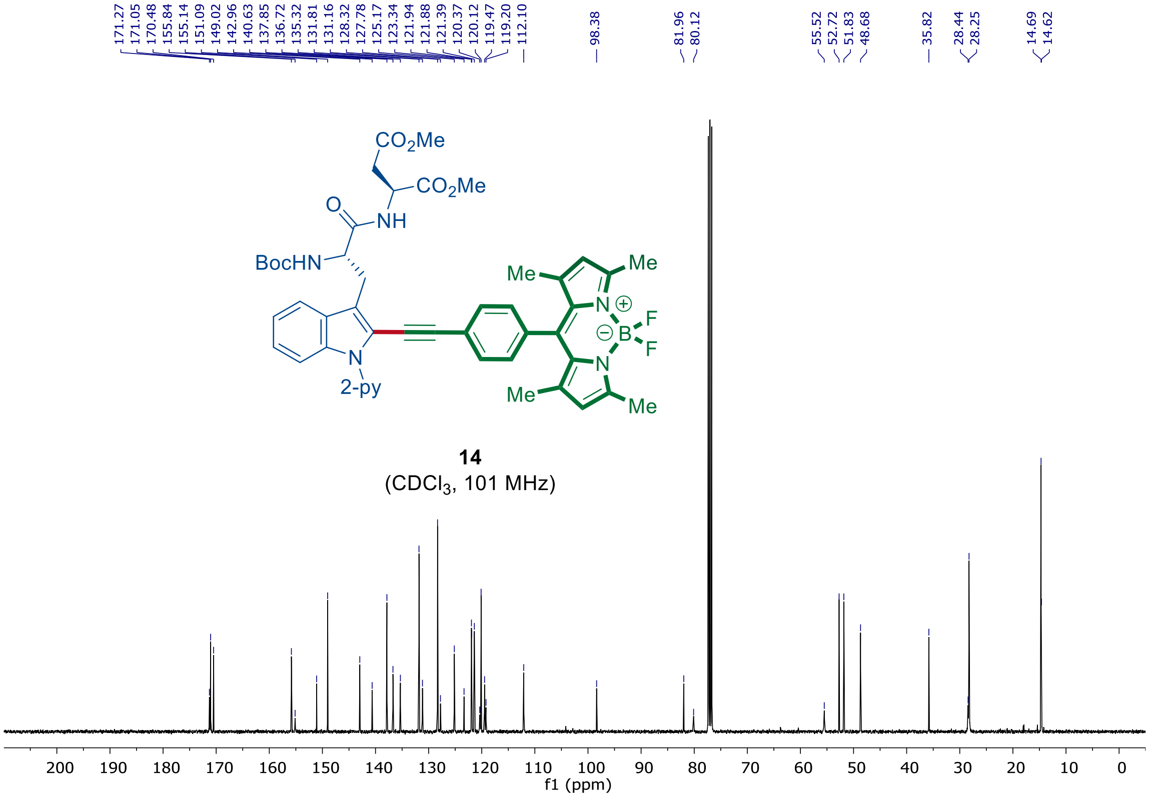

Supplementary Figure 67. ^{13}C-NMR spectrum of **14**.

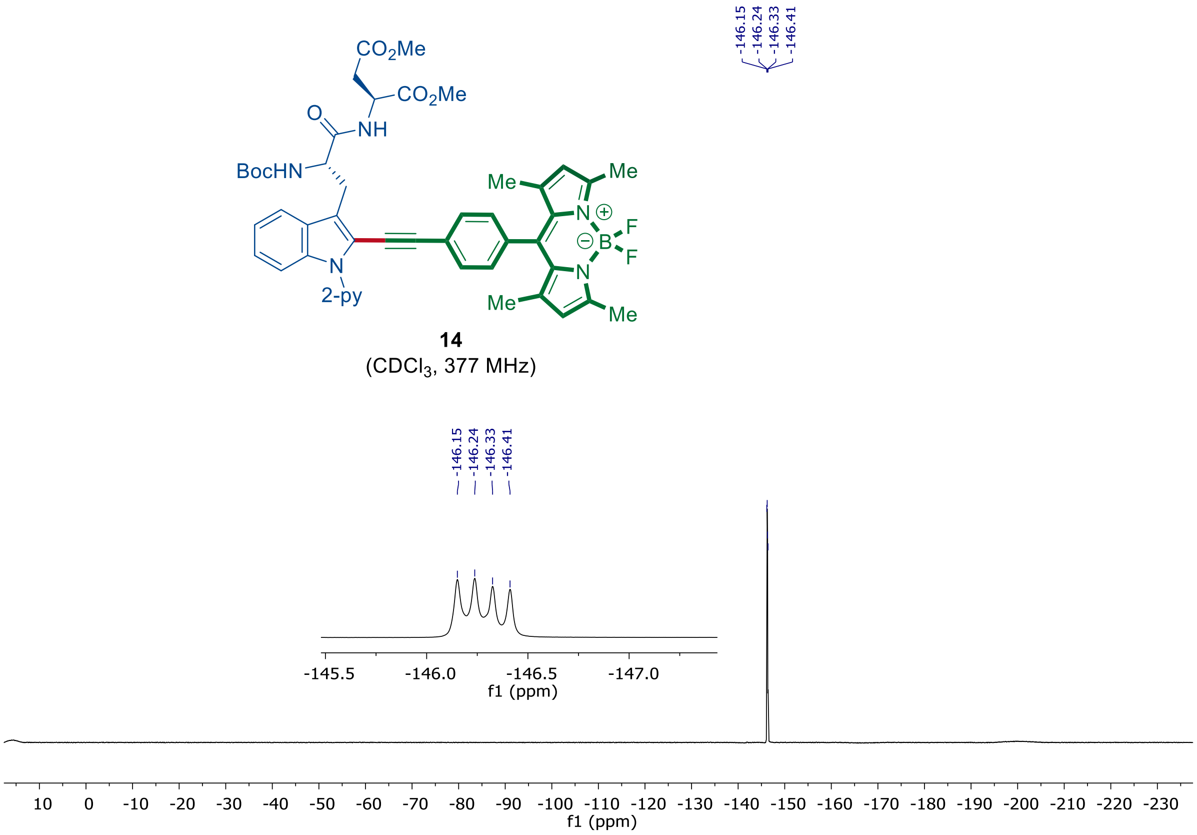

Supplementary Figure 68. ^{19}F-NMR spectrum of **14**.

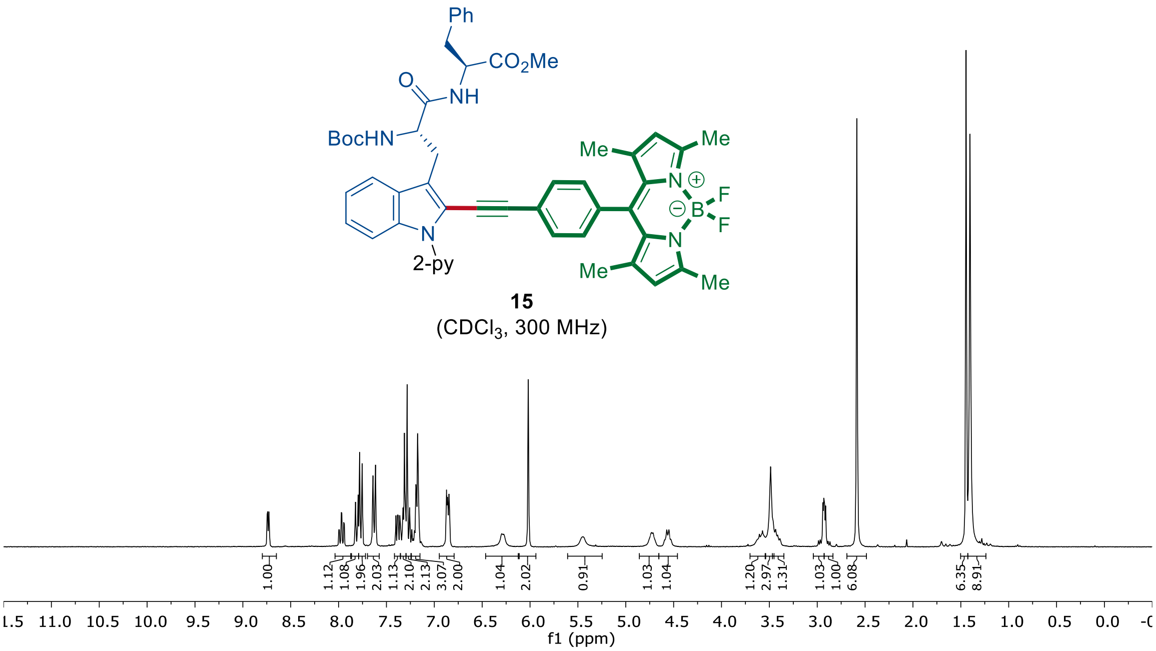

Supplementary Figure 69. ^{1}H-NMR spectrum of **15**.

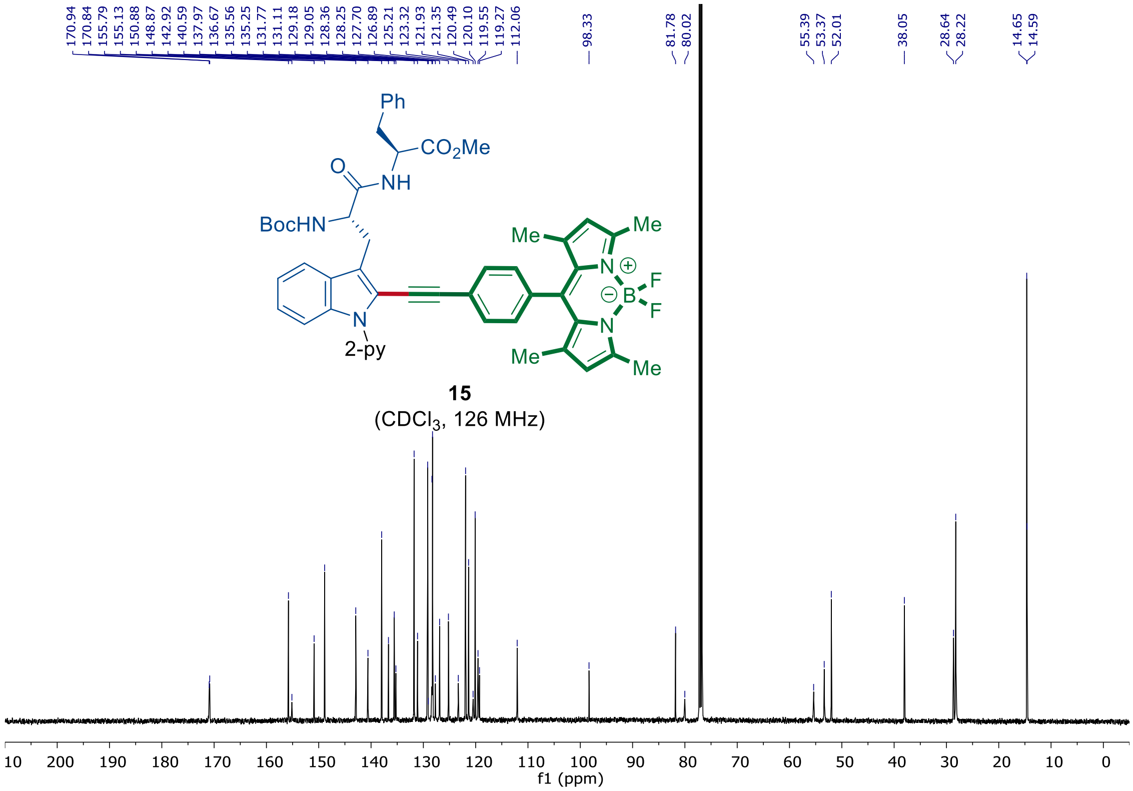

Supplementary Figure 70. ^{13}C-NMR spectrum of **15**.

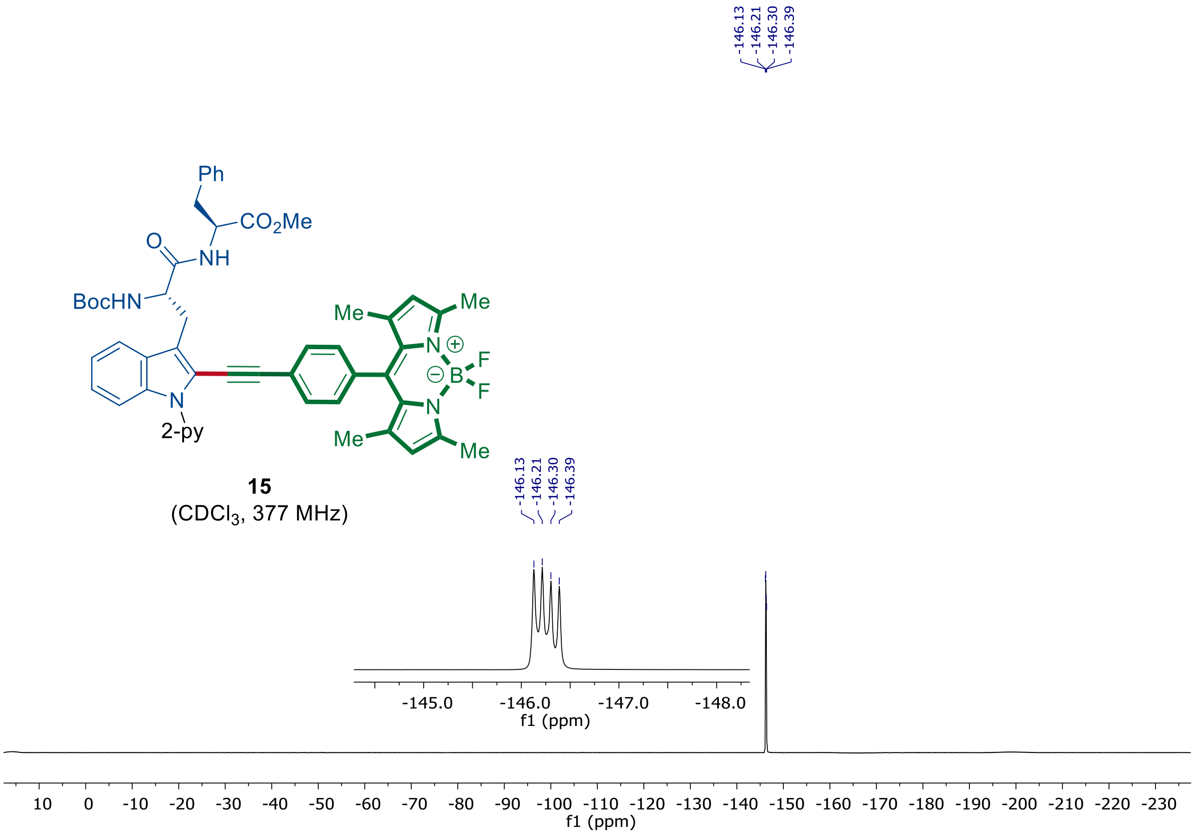

Supplementary Figure 71. ^{19}F-NMR spectrum of **15**.

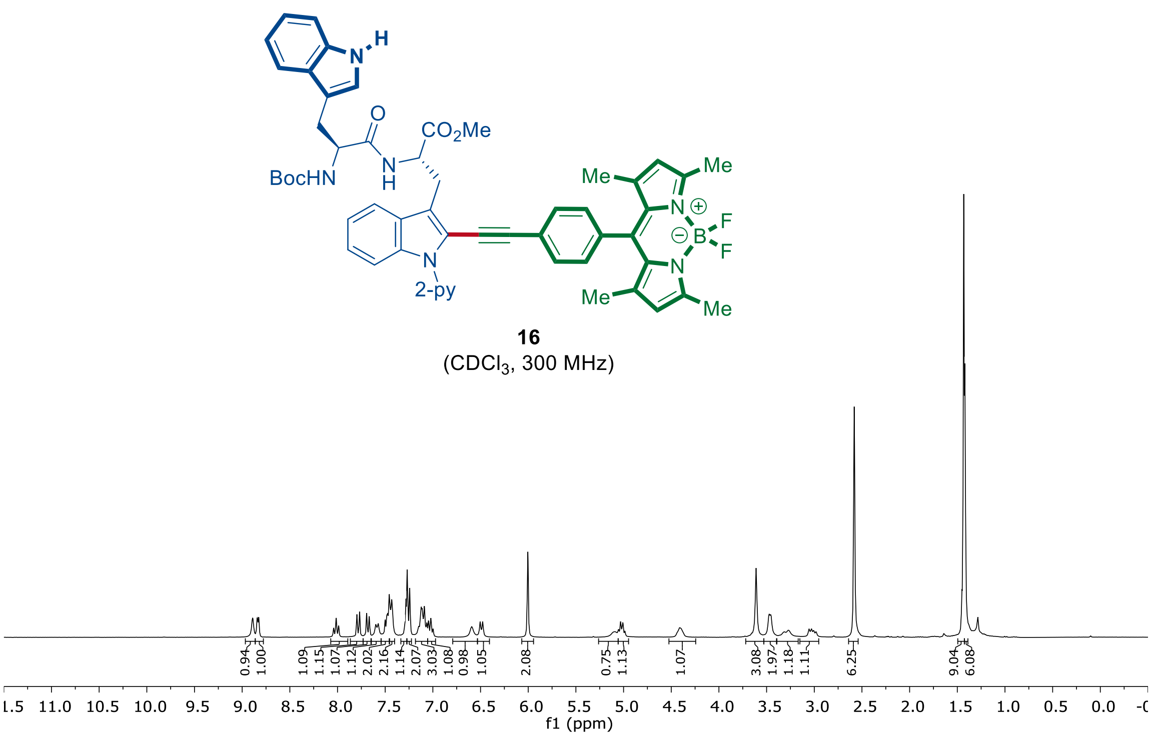

Supplementary Figure 72. ^{1}H-NMR spectrum of **16**.

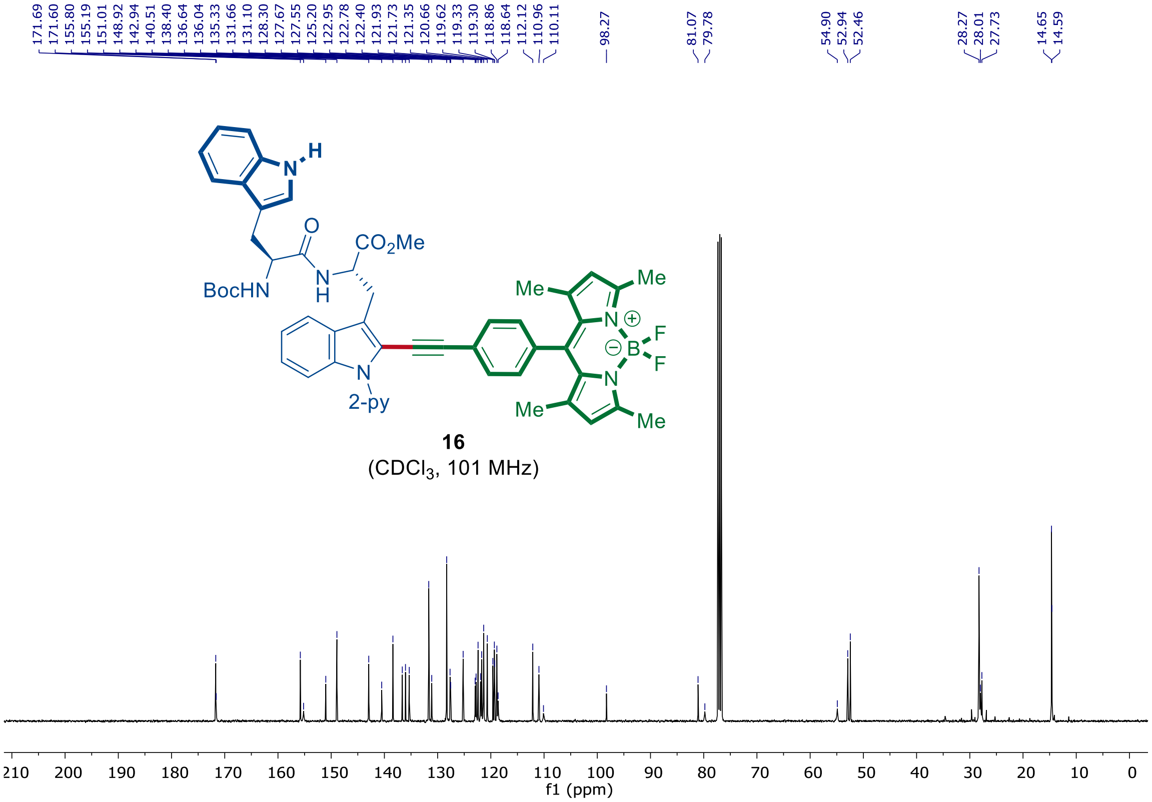

Supplementary Figure 73. ^{13}C-NMR spectrum of **16**.

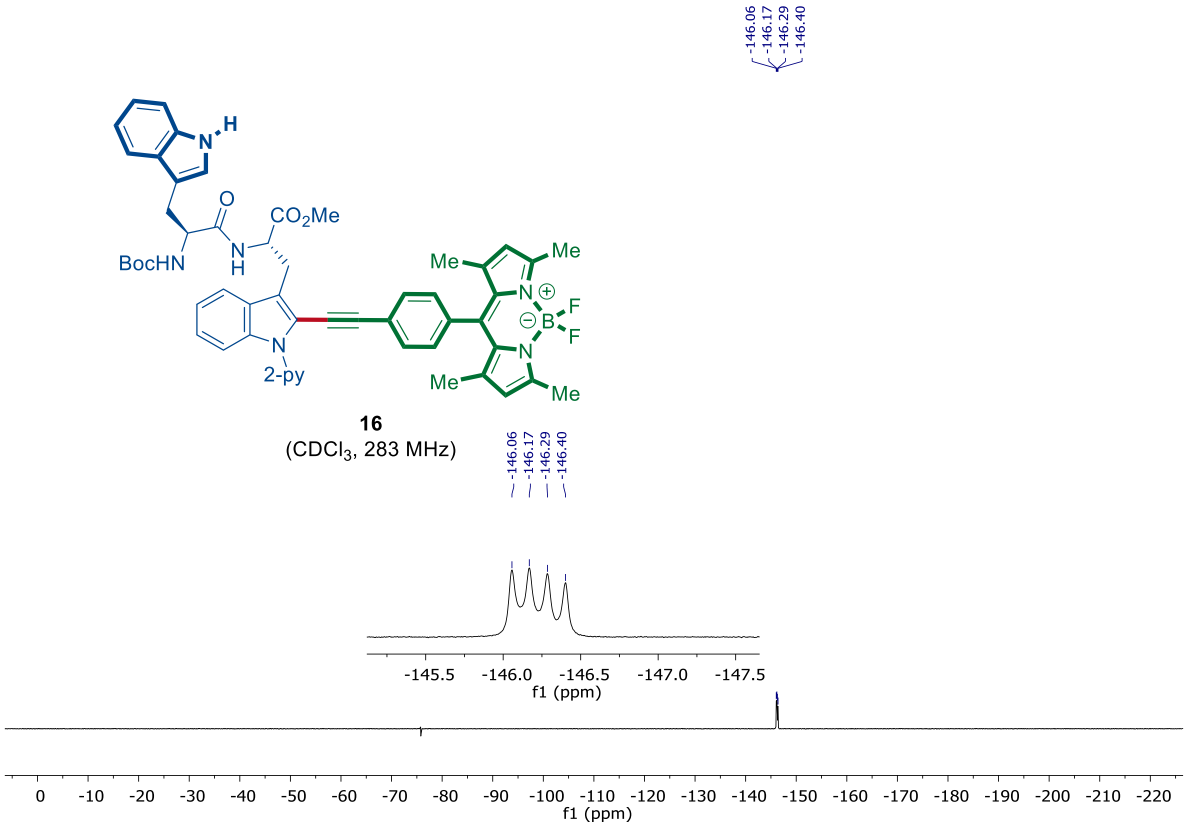

Supplementary Figure 74. ^{19}F-NMR spectrum of **16**.

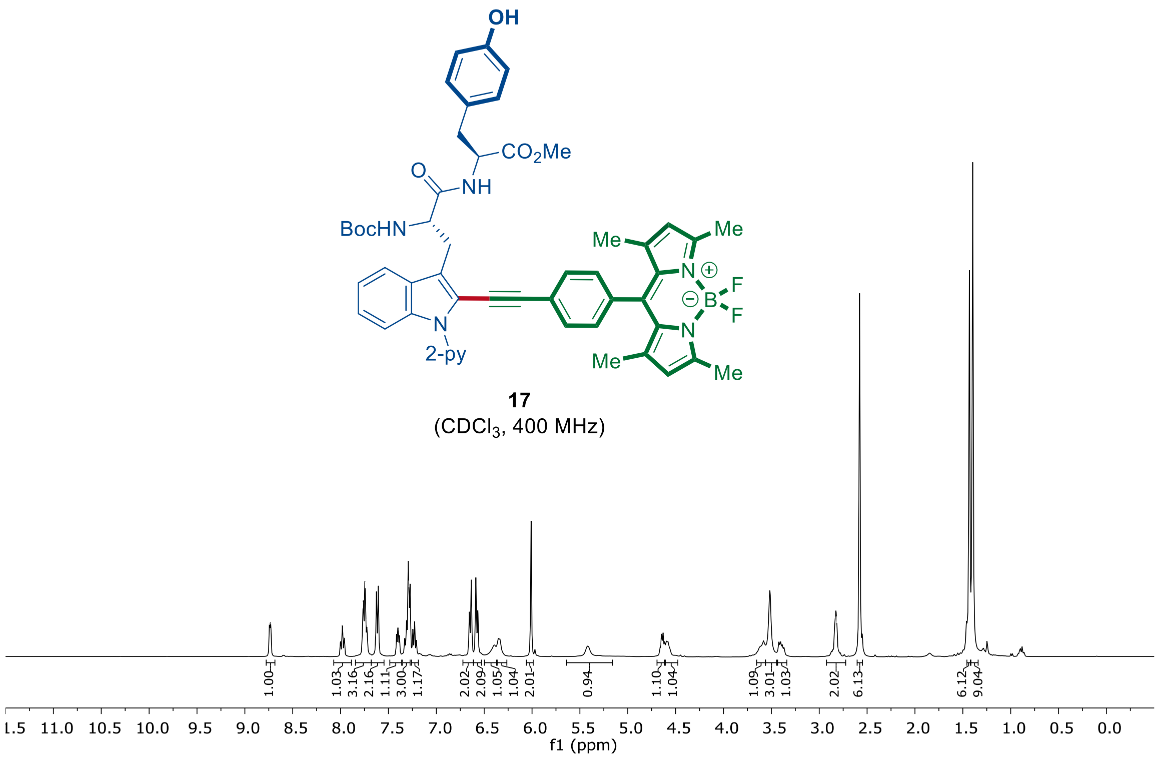

Supplementary Figure 75. ^{1}H-NMR spectrum of **17**.

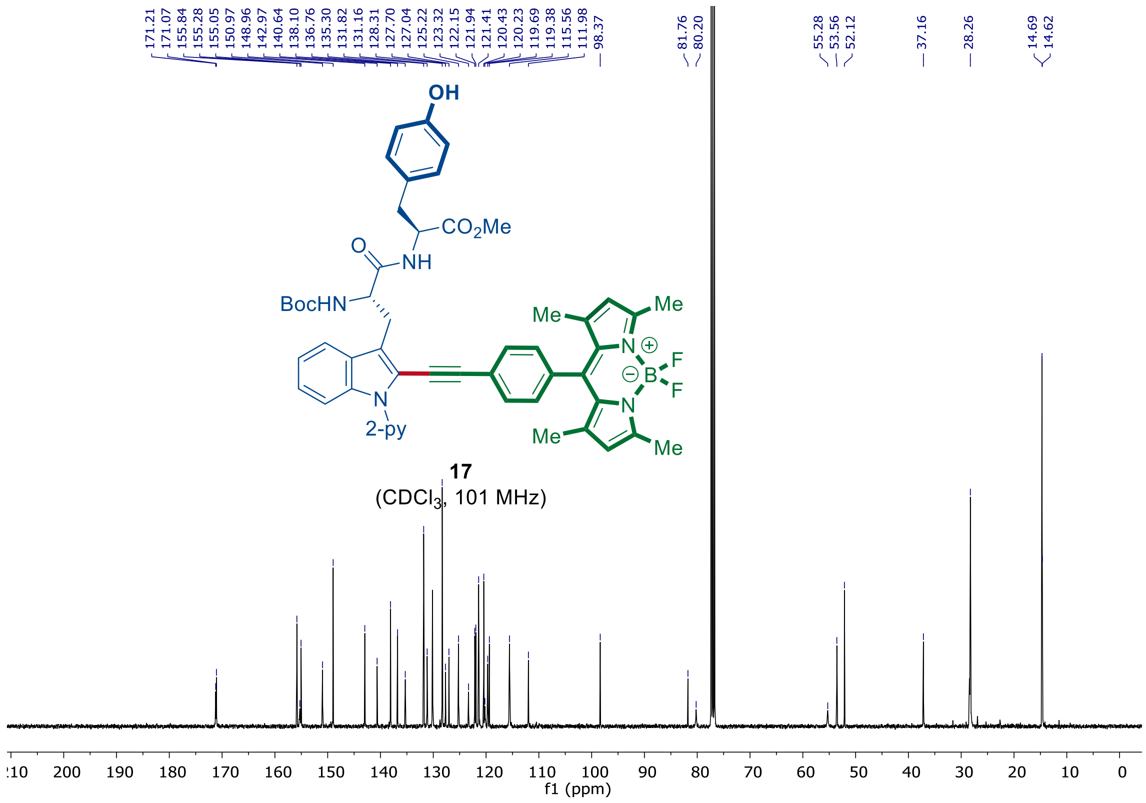

Supplementary Figure 76. ^{13}C-NMR spectrum of **17**.

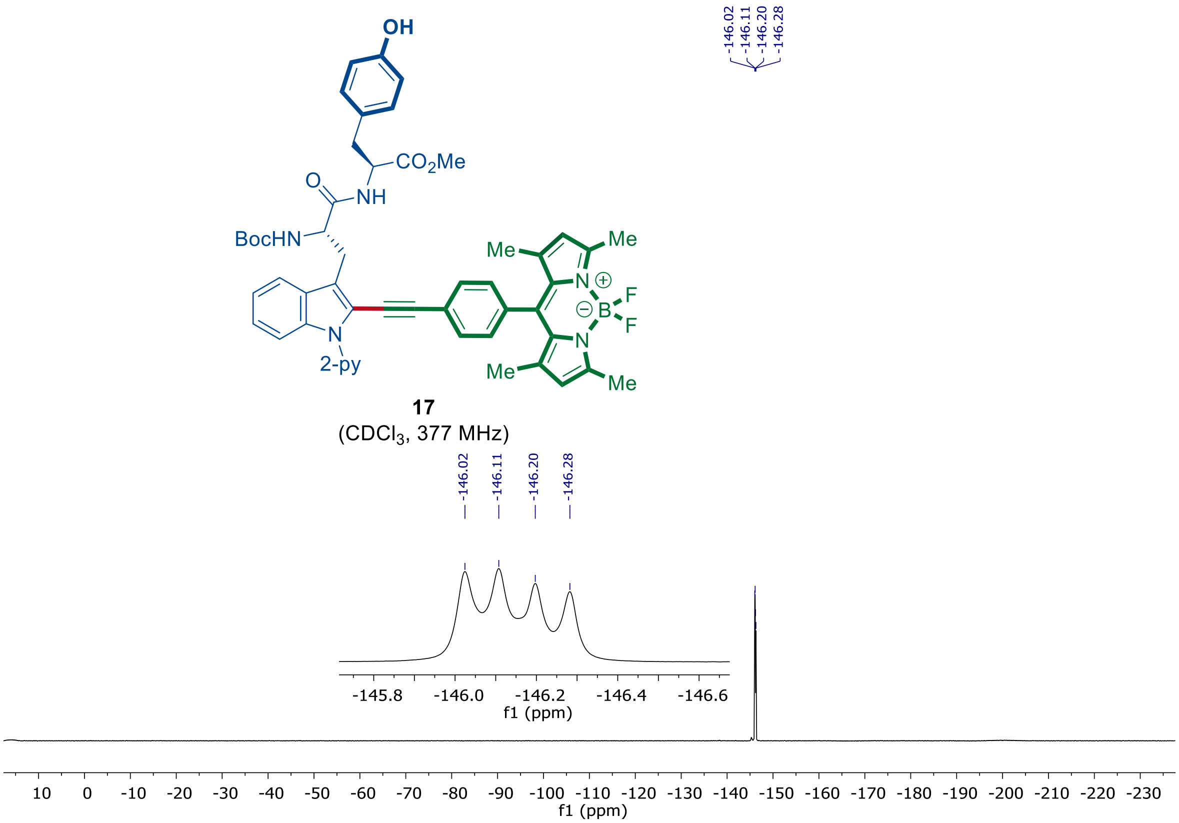

Supplementary Figure 77. ^{19}F-NMR spectrum of **17**.

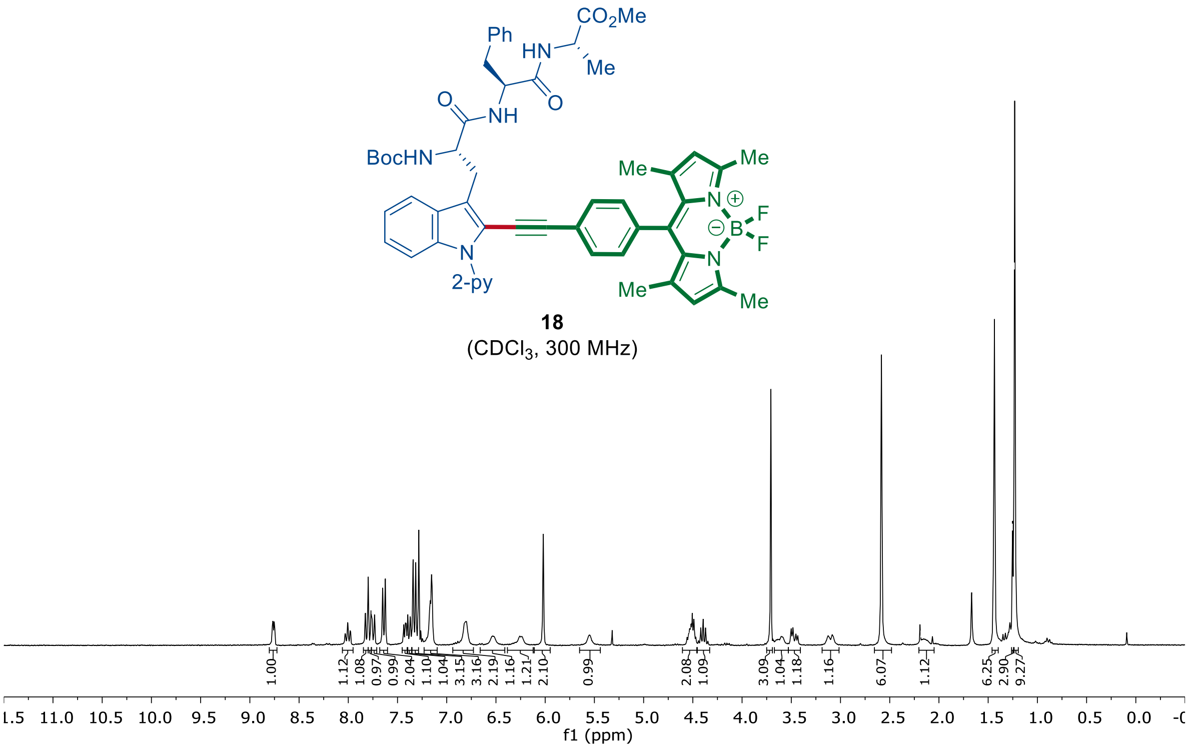

Supplementary Figure 78. ^{1}H-NMR spectrum of **18**.

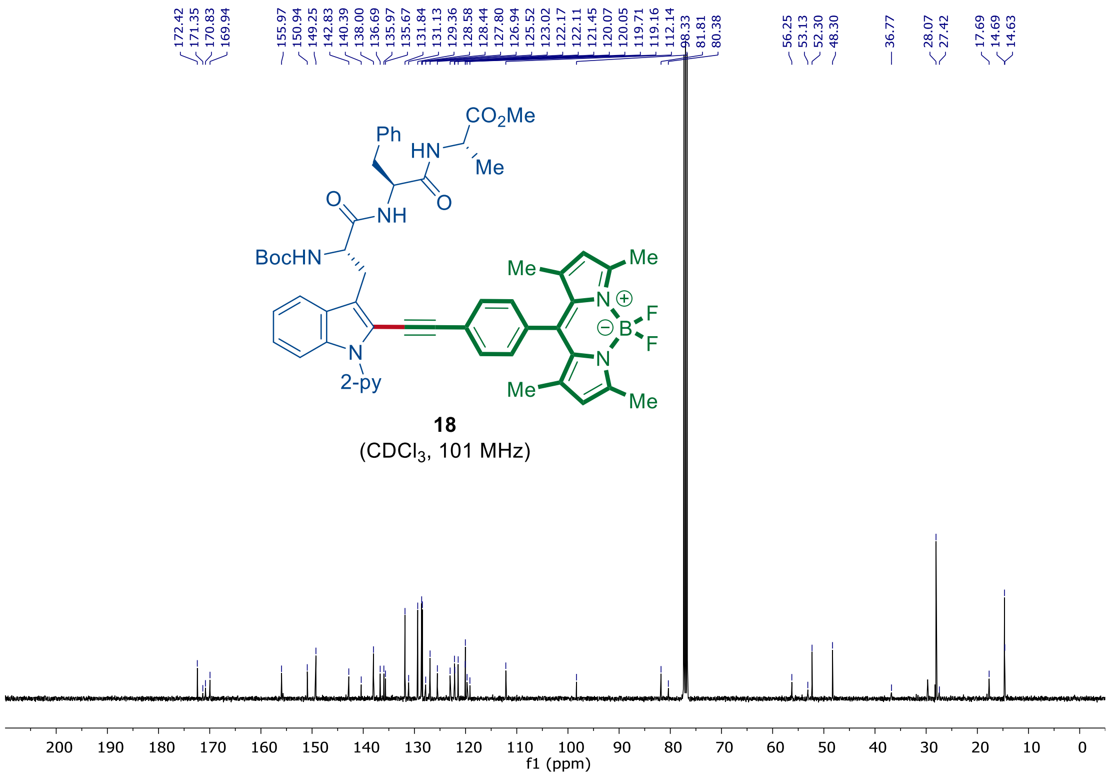

Supplementary Figure 79. ^{13}C-NMR spectrum of **18**.

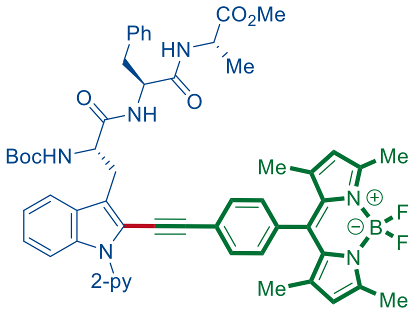

18
(CDCl$_3$, 377 MHz)

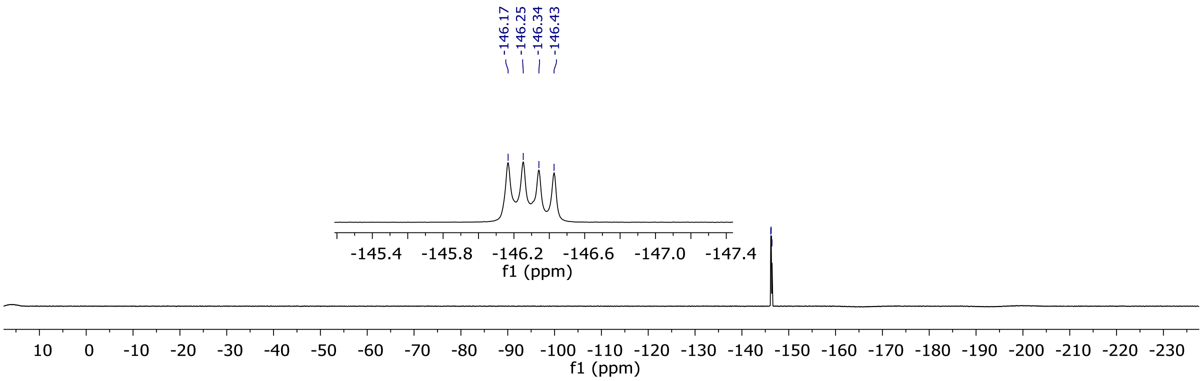

Supplementary Figure 80. ^{19}F-NMR spectrum of **18**.

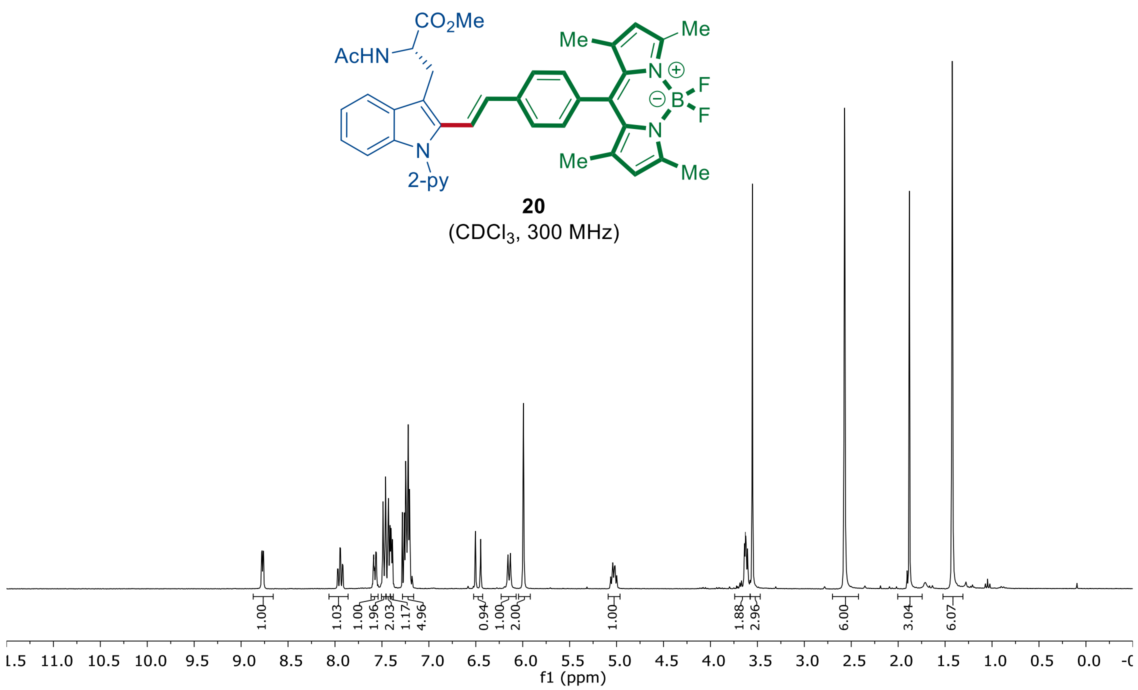

Supplementary Figure 81. ^{1}H-NMR spectrum of **20**.

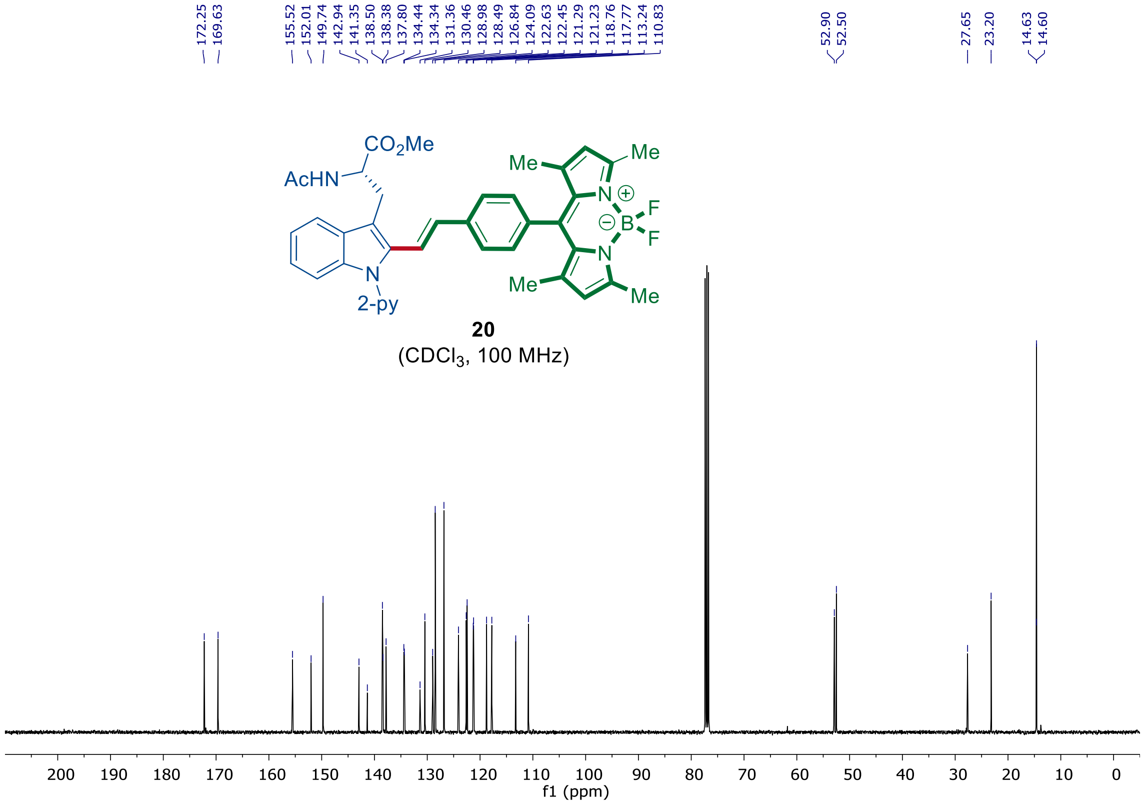

Supplementary Figure 82. ^{13}C-NMR spectrum of **20**.

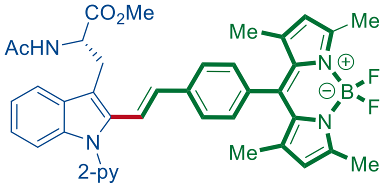

20
($CDCl_3$, 377 MHz)

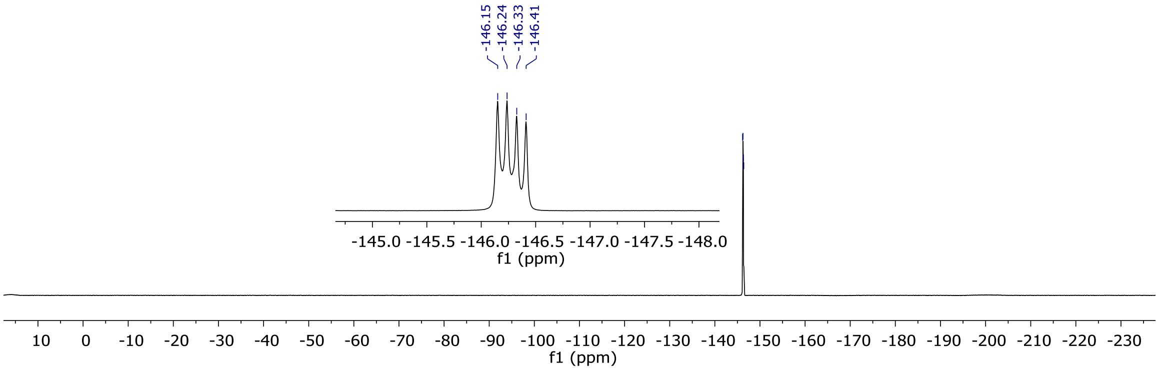

Supplementary Figure 83. ^{19}F-NMR spectrum of **20**.

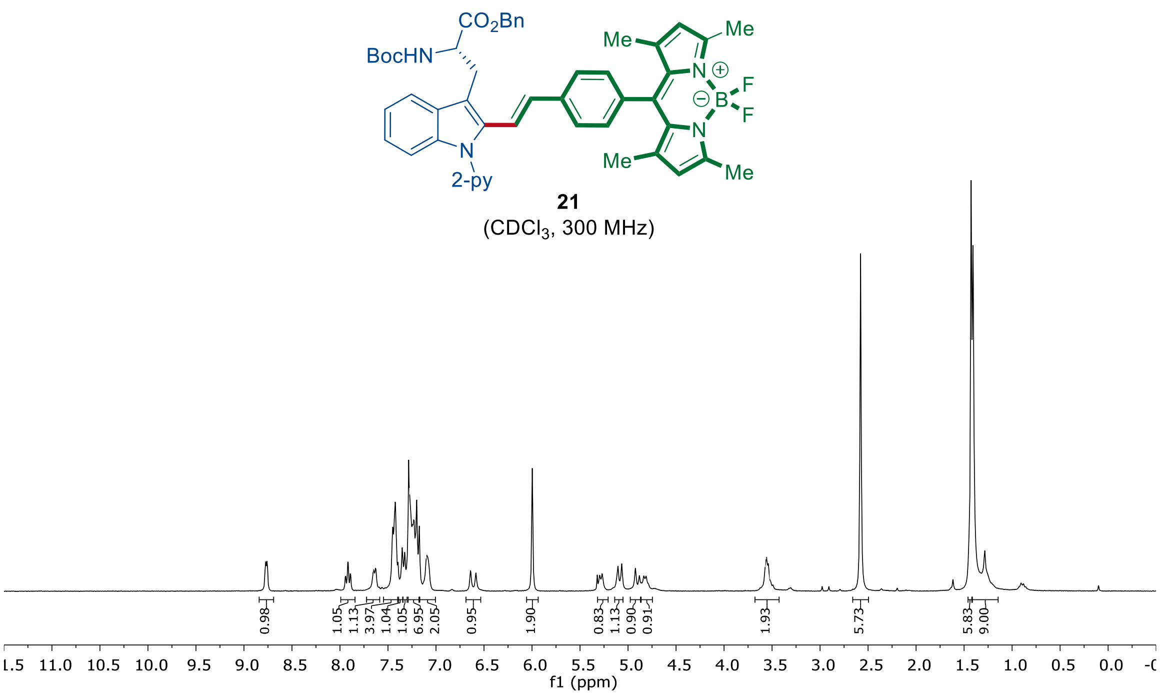

Supplementary Figure 84. ^{1}H-NMR spectrum of **21**.

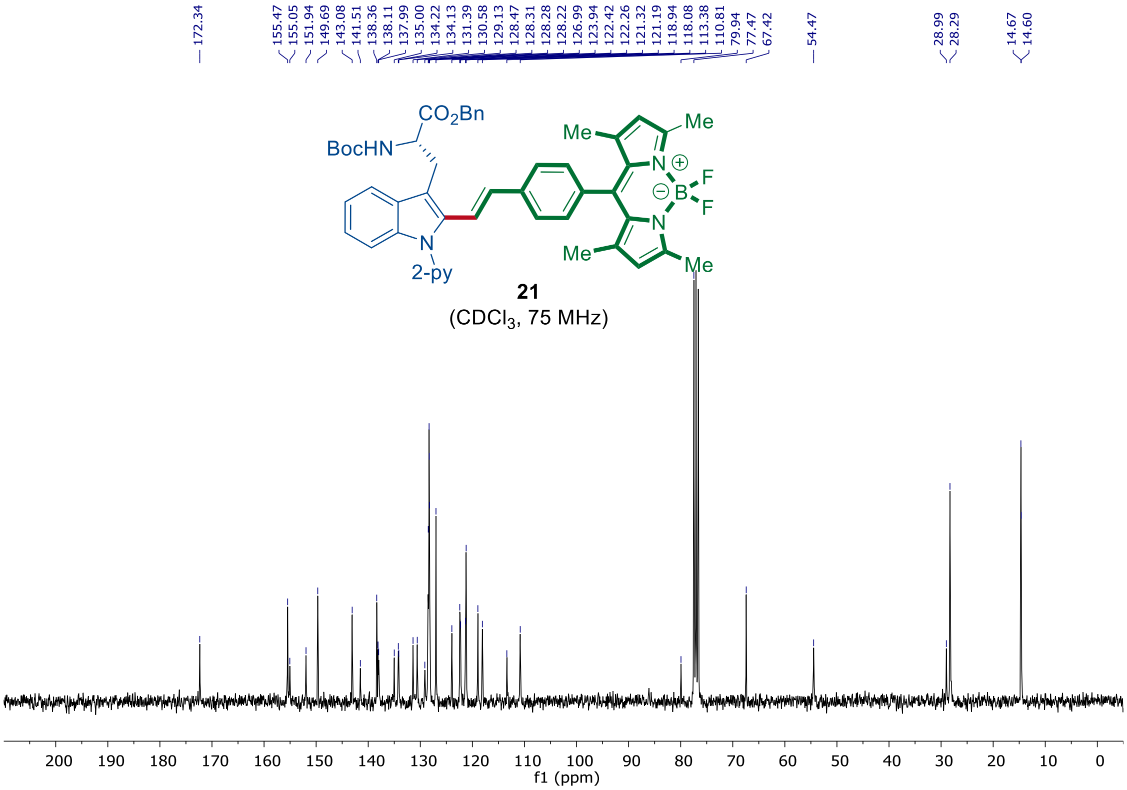

Supplementary Figure 85. ^{13}C-NMR spectrum of **21**.

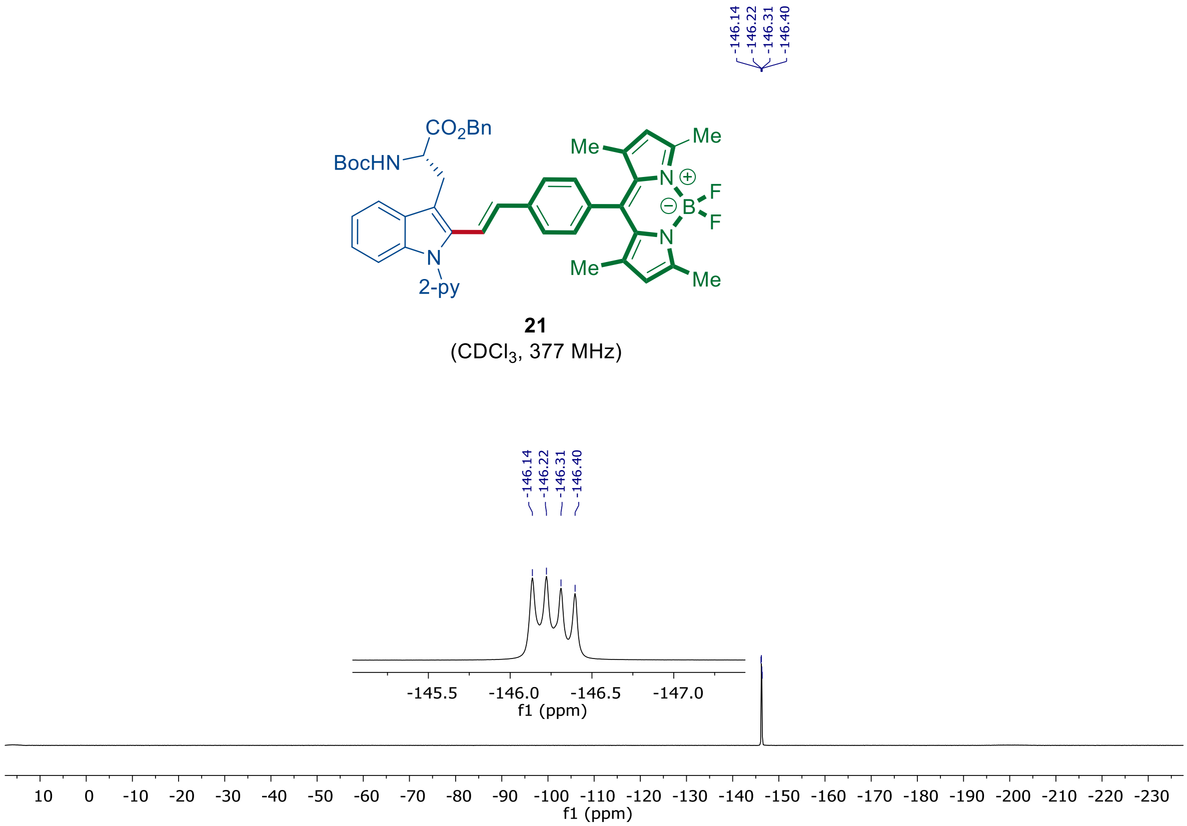

Supplementary Figure 86. ^{19}F-NMR spectrum of **21**.

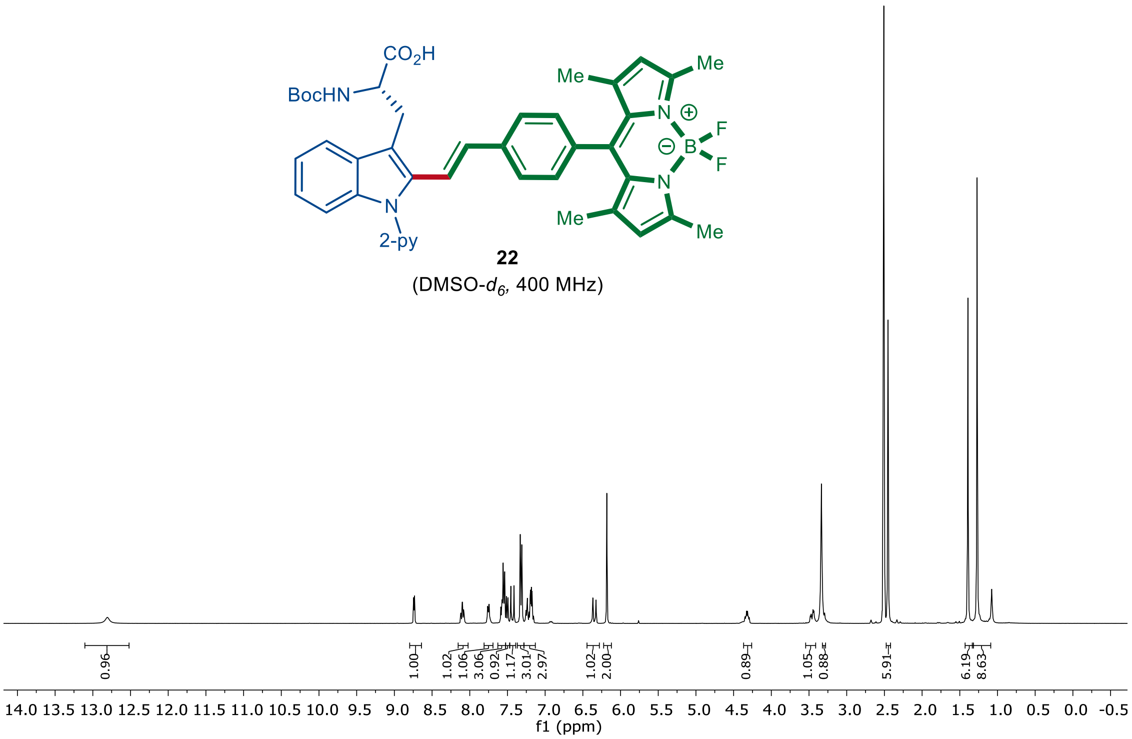

Supplementary Figure 87. ^{1}H-NMR spectrum of **22**.

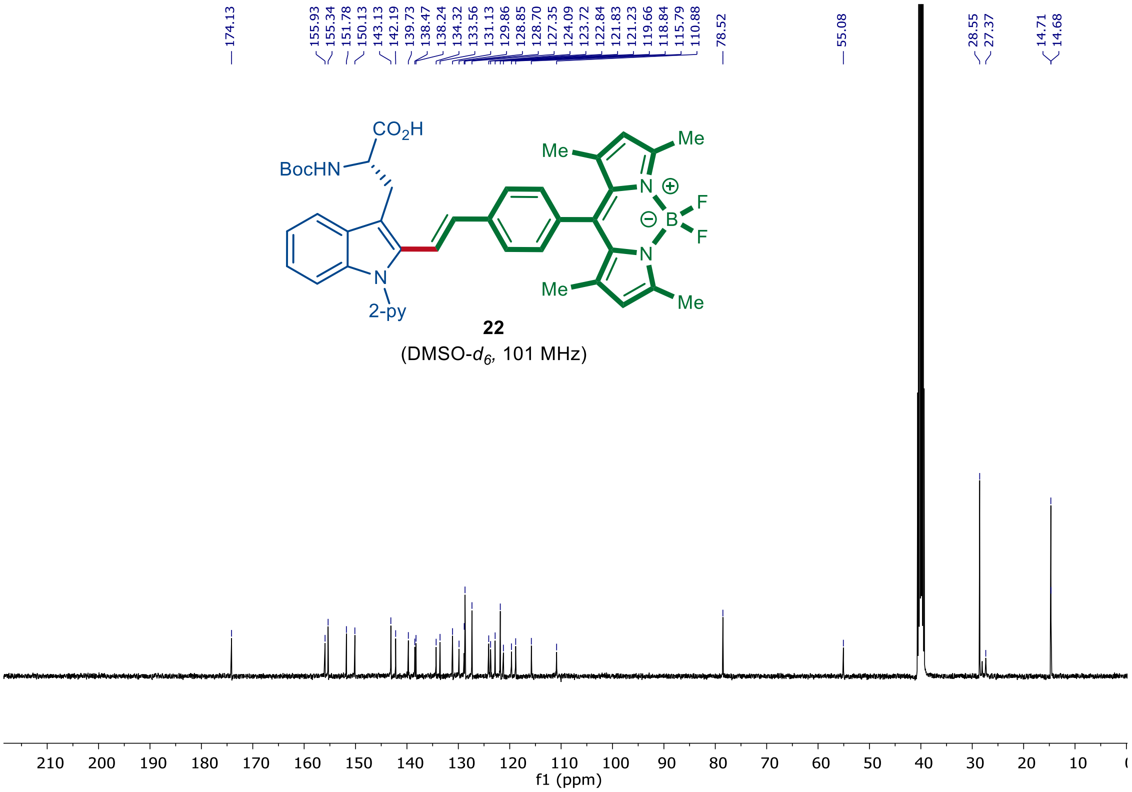

Supplementary Figure 88. ^{13}C-NMR spectrum of **22**.

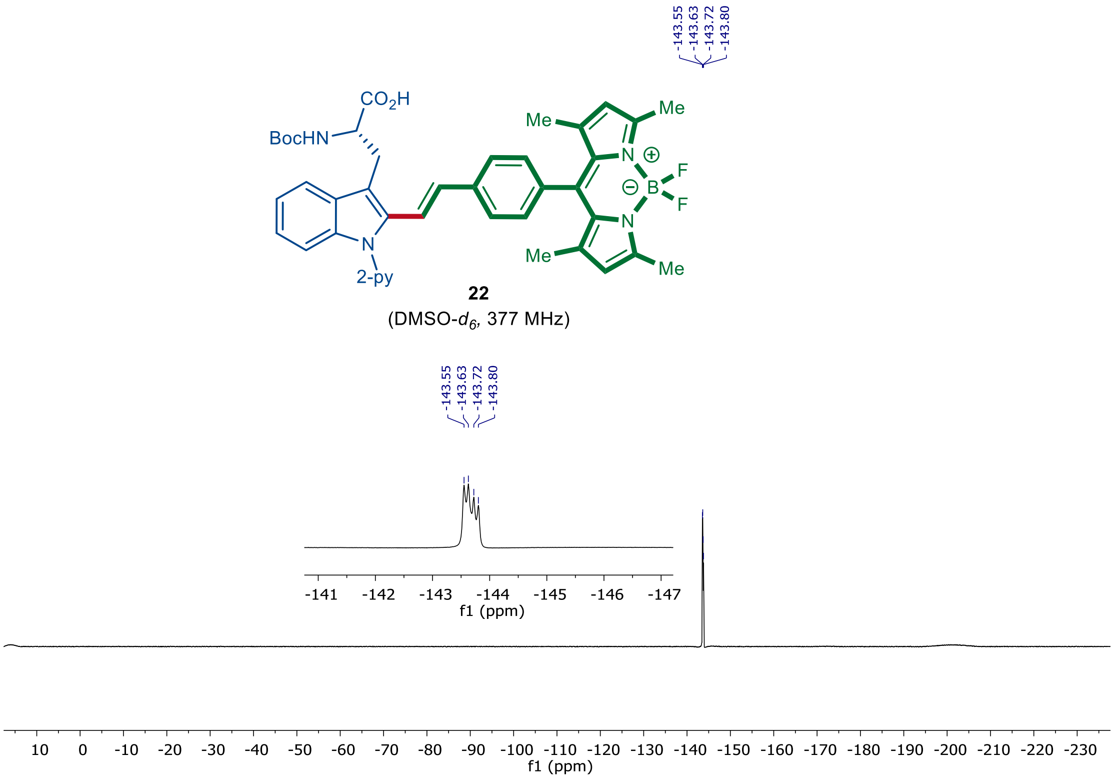

Supplementary Figure 89. ^{19}F-NMR spectrum of **22**.

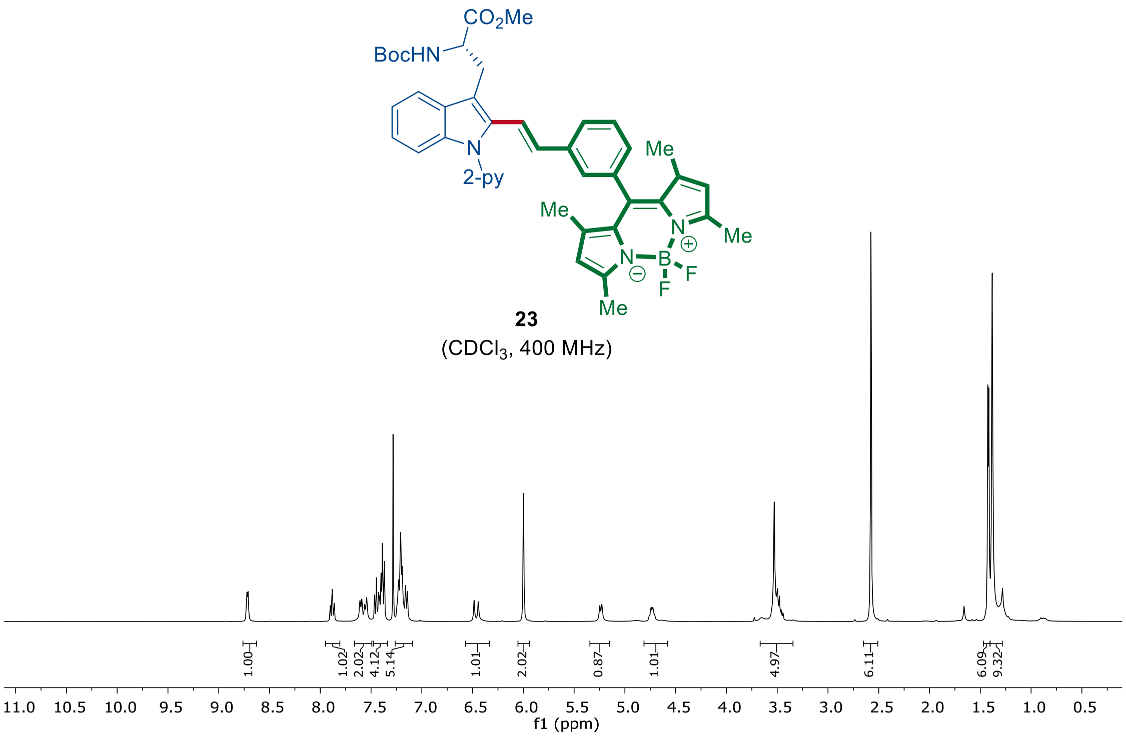

Supplementary Figure 90. ^{1}H-NMR spectrum of **23**.

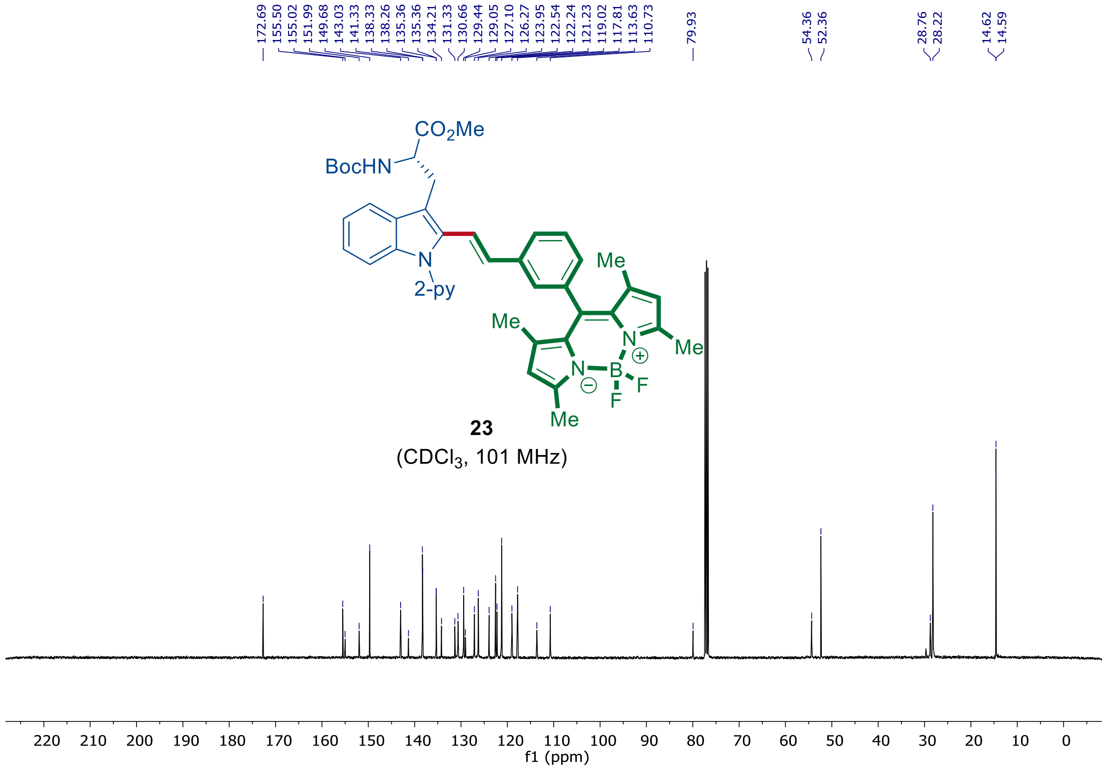

Supplementary Figure 91. ^{13}C-NMR spectrum of **23**.

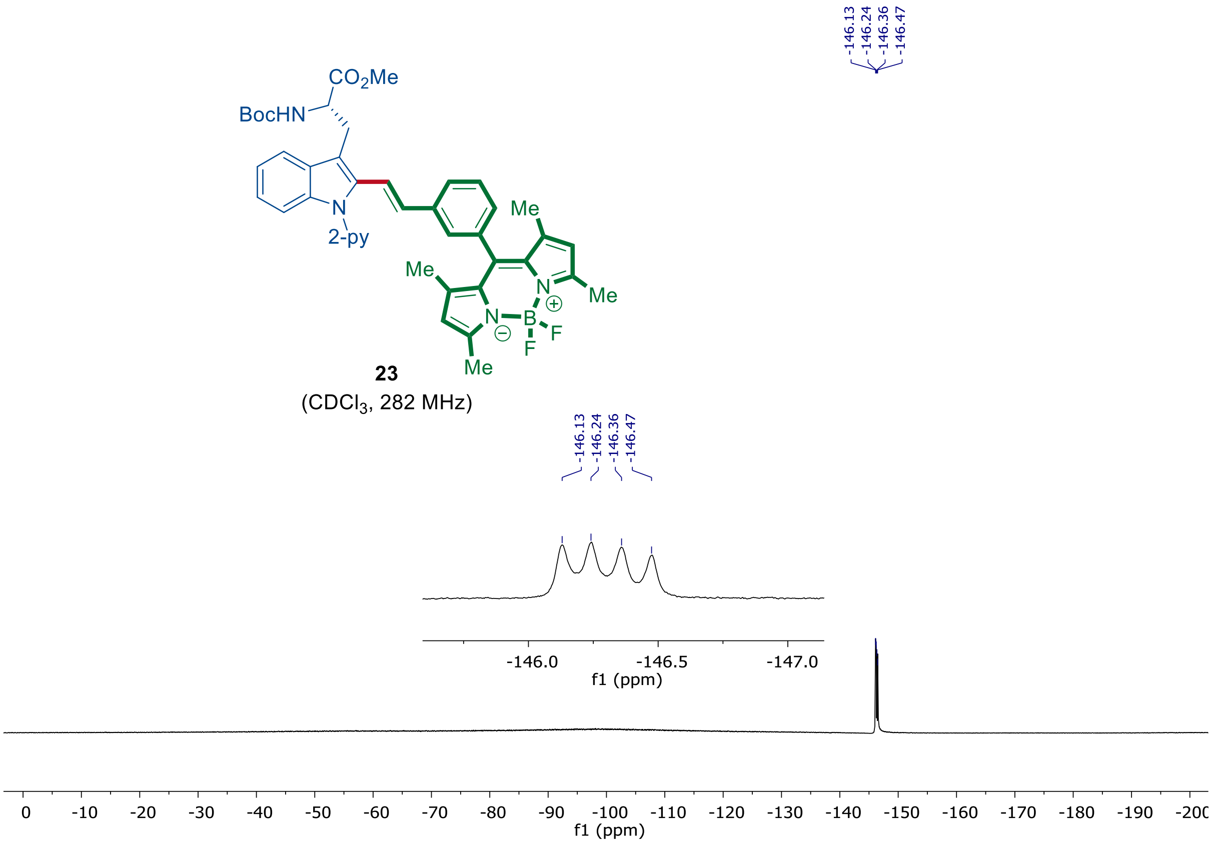

Supplementary Figure 92. ^{19}F-NMR spectrum of **23**.

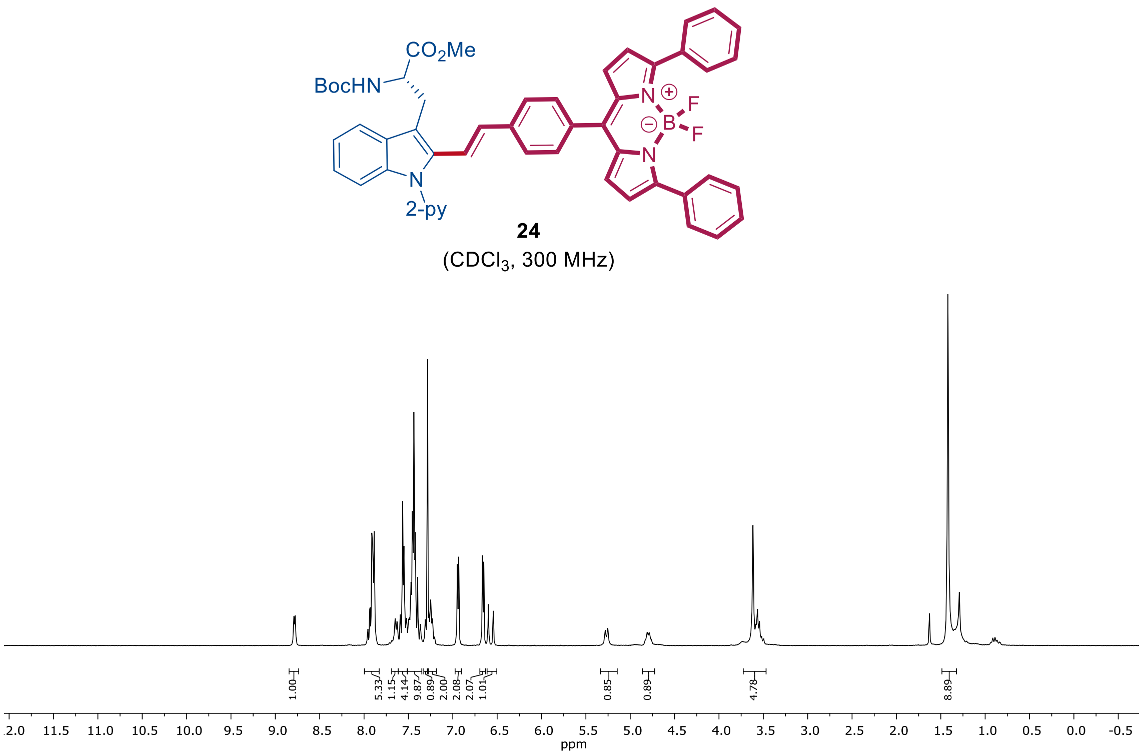

Supplementary Figure 93. ^{1}H-NMR spectrum of **24**.

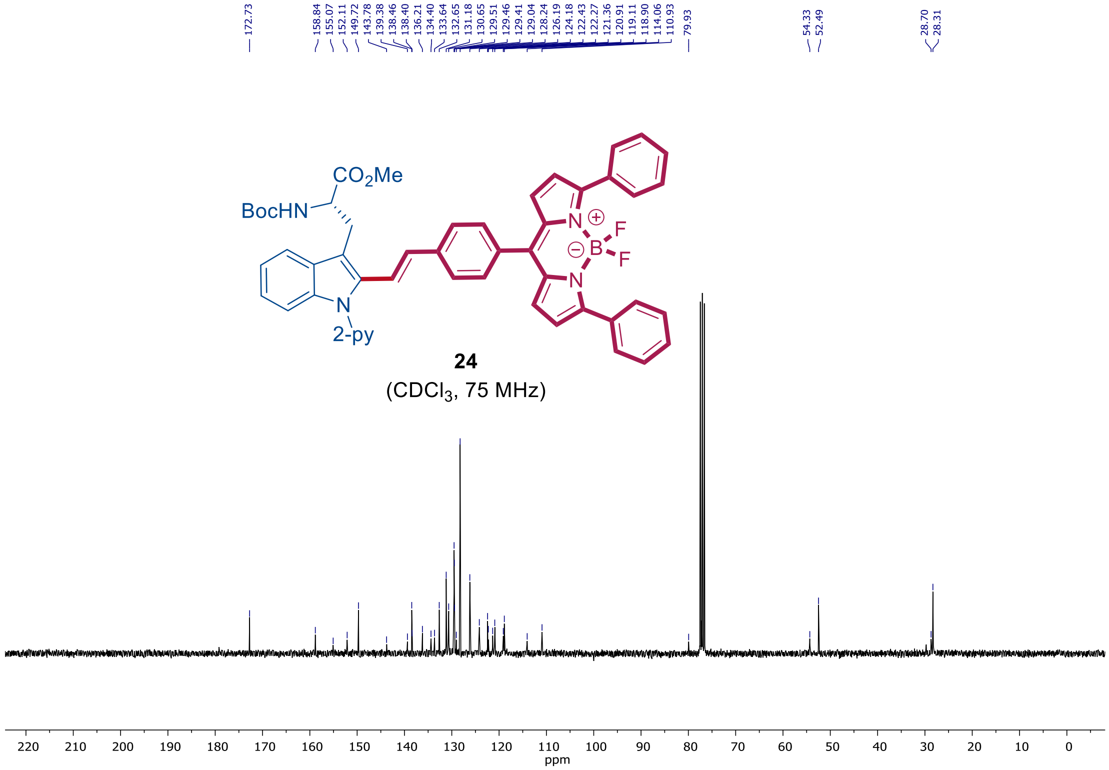

Supplementary Figure 94. ^{13}C-NMR spectrum of **24**.

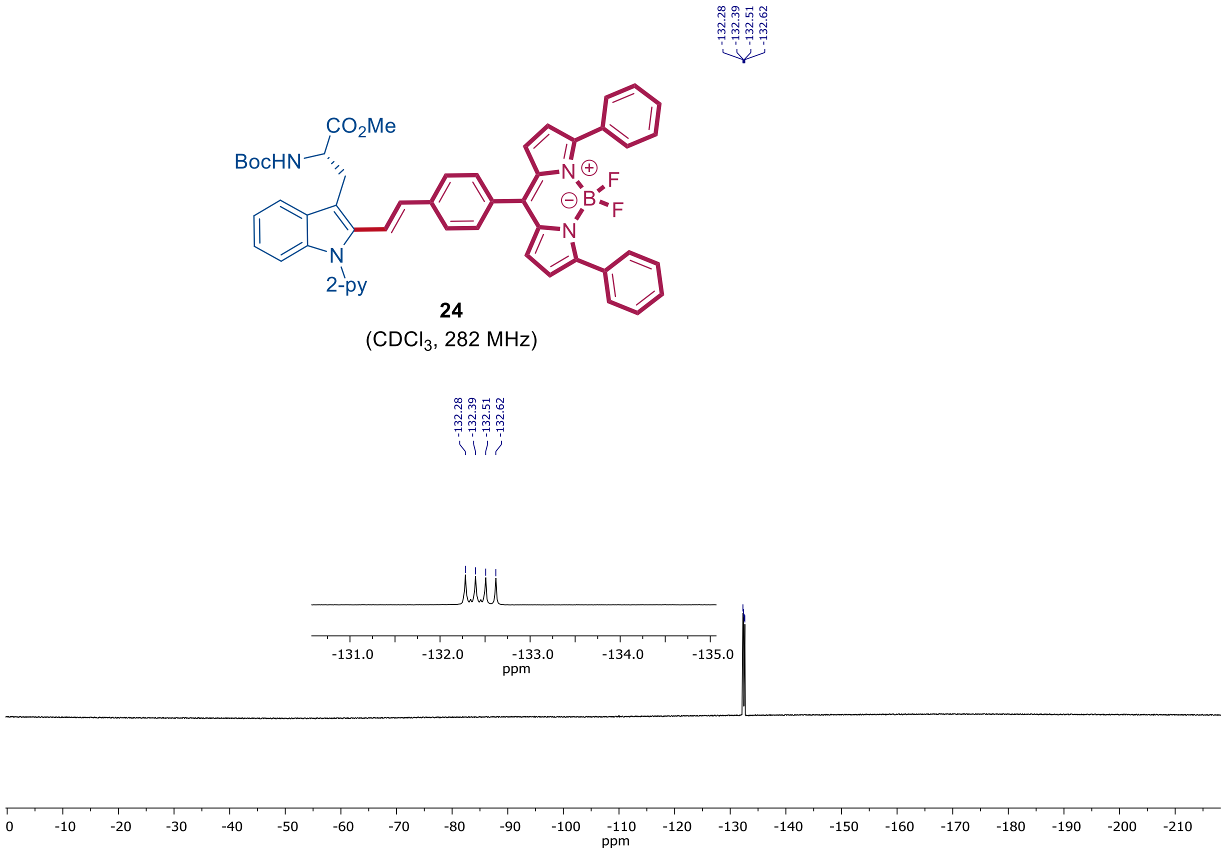

Supplementary Figure 95. ^{19}F-NMR spectrum of **24**.

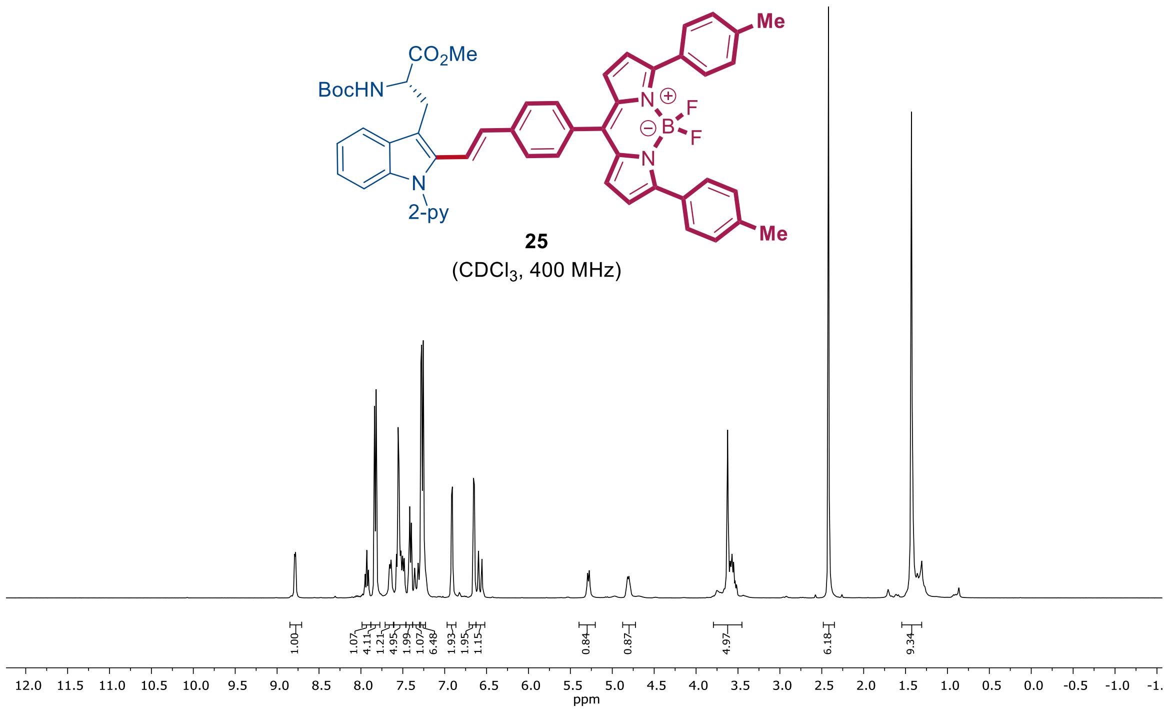

Supplementary Figure 96. ^{1}H-NMR spectrum of **25**.

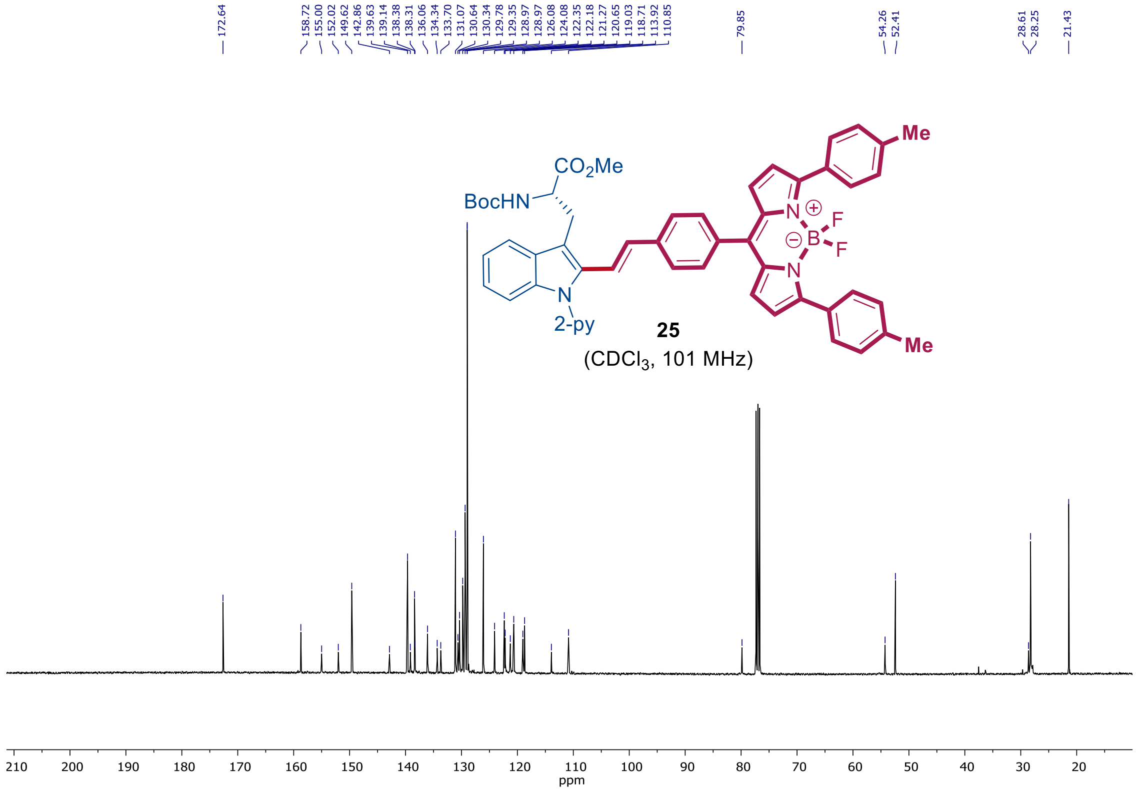

Supplementary Figure 97. ^{13}C-NMR spectrum of **25**.

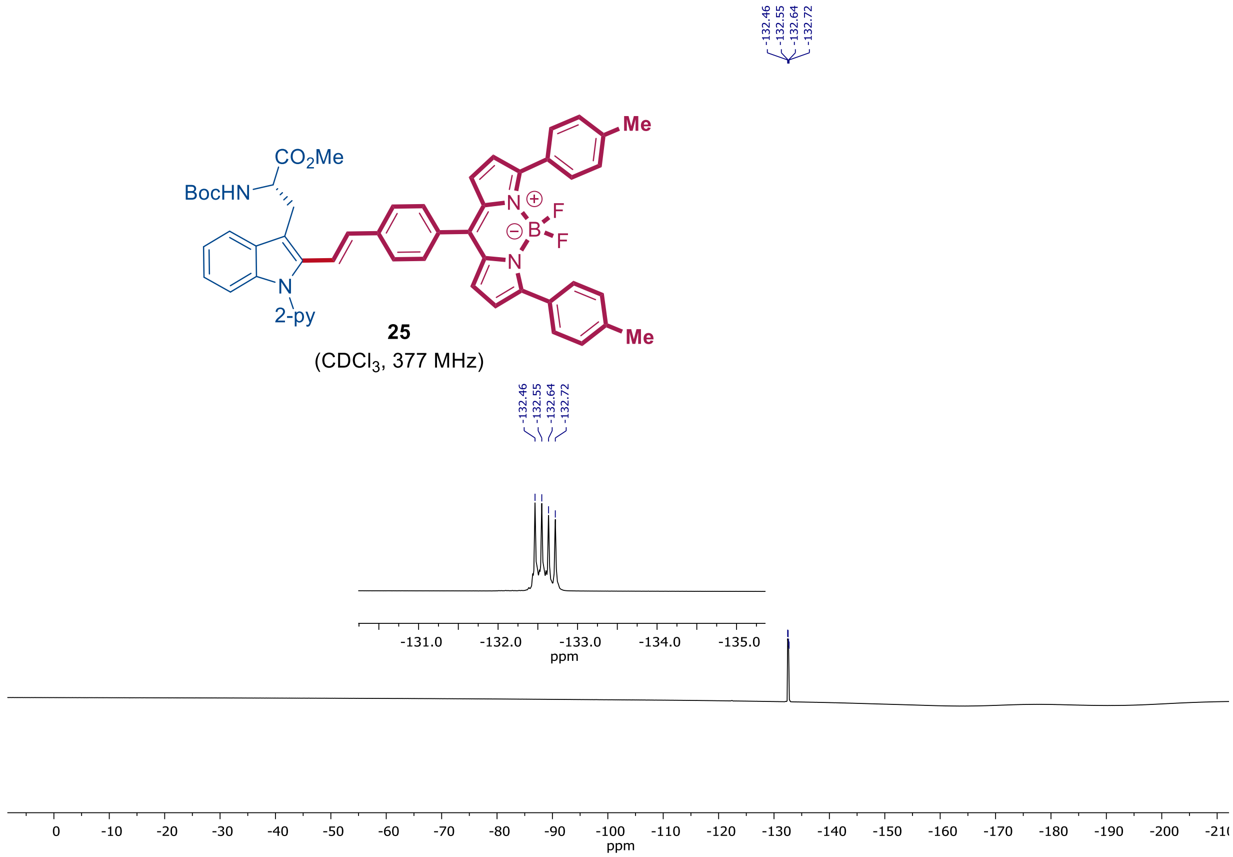

Supplementary Figure 98. ^{19}F-NMR spectrum of **25**.

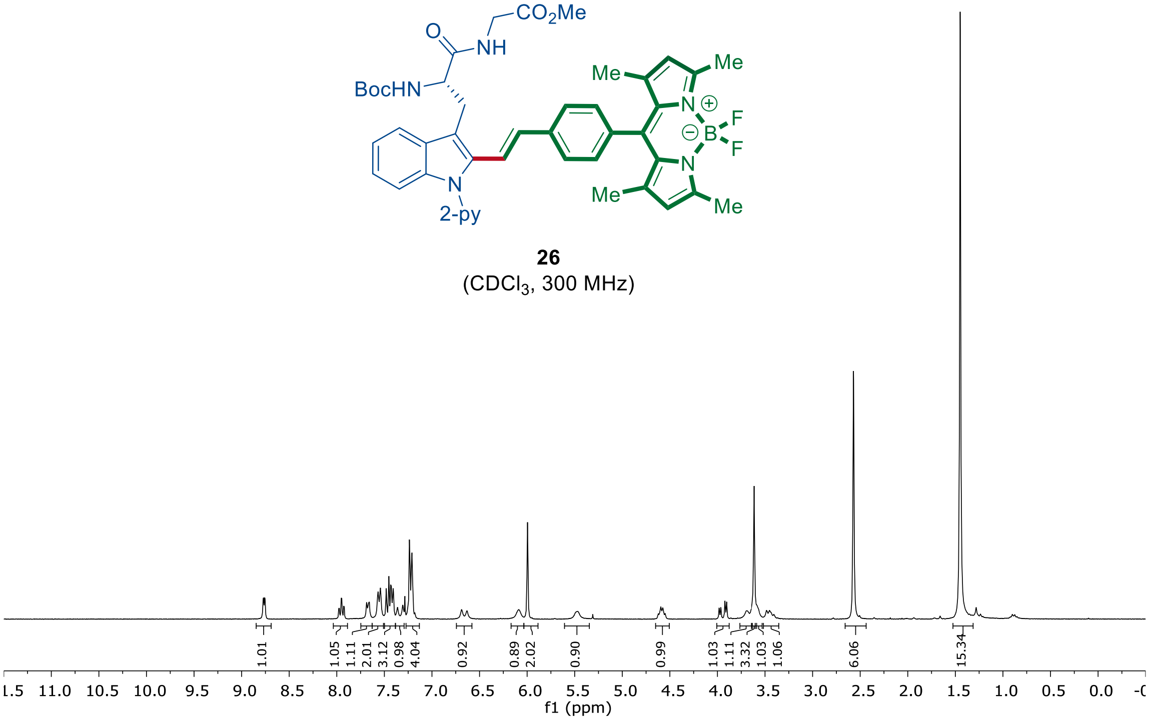

Supplementary Figure 99. ^{1}H-NMR spectrum of **26**.

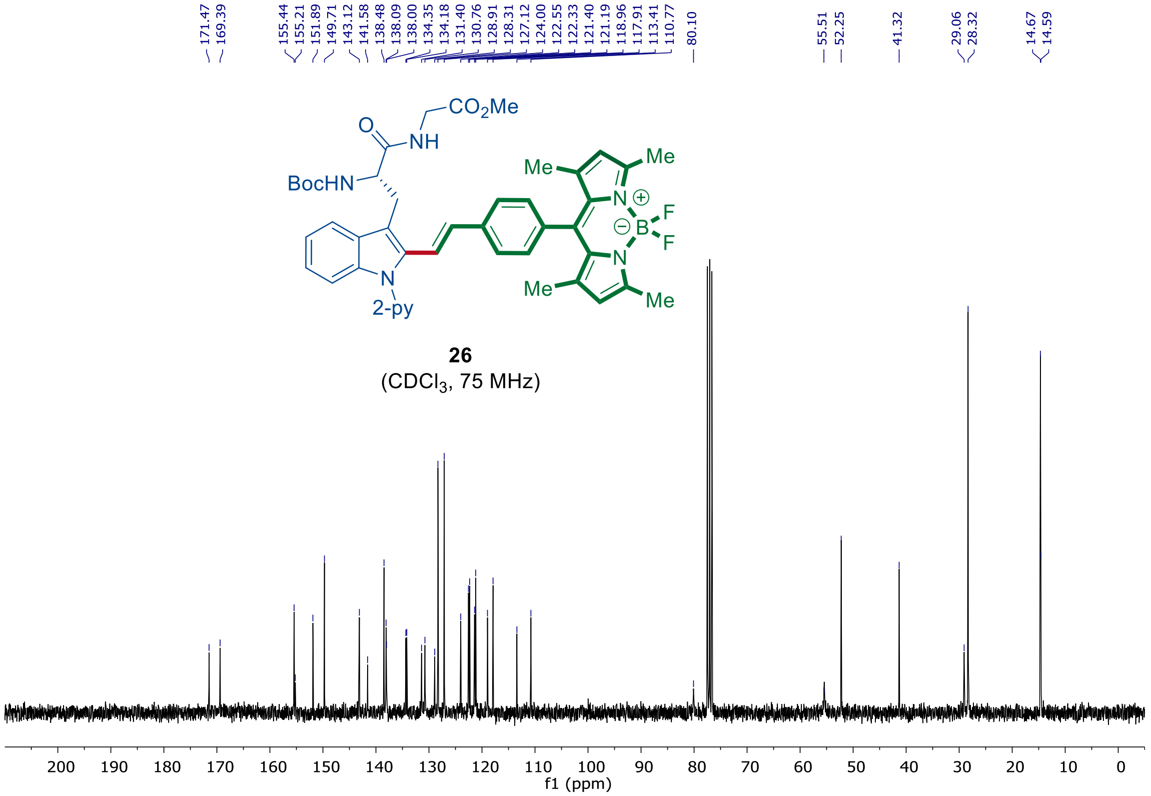

Supplementary Figure 100. ^{13}C-NMR spectrum of **26**.

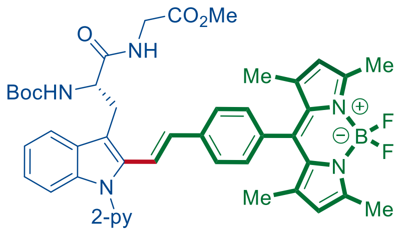

26
($CDCl_3$, 283 MHz)

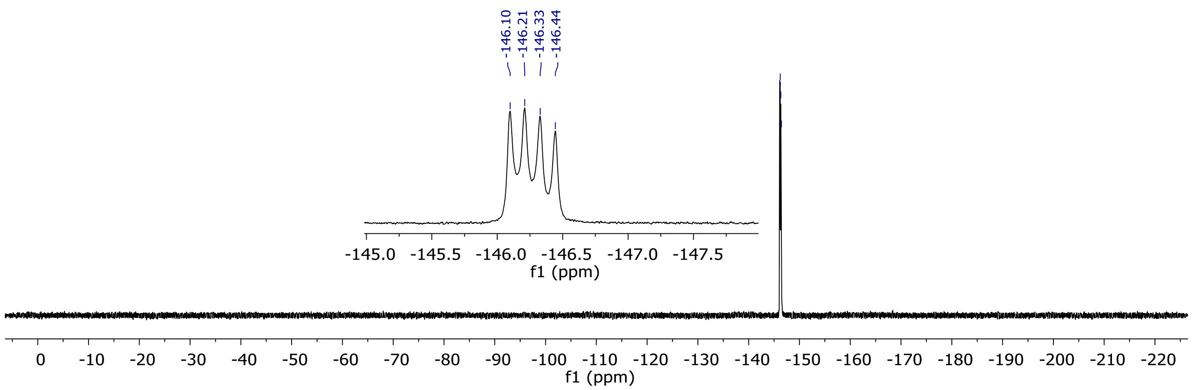

Supplementary Figure 101. ^{19}F-NMR spectrum of **26**.

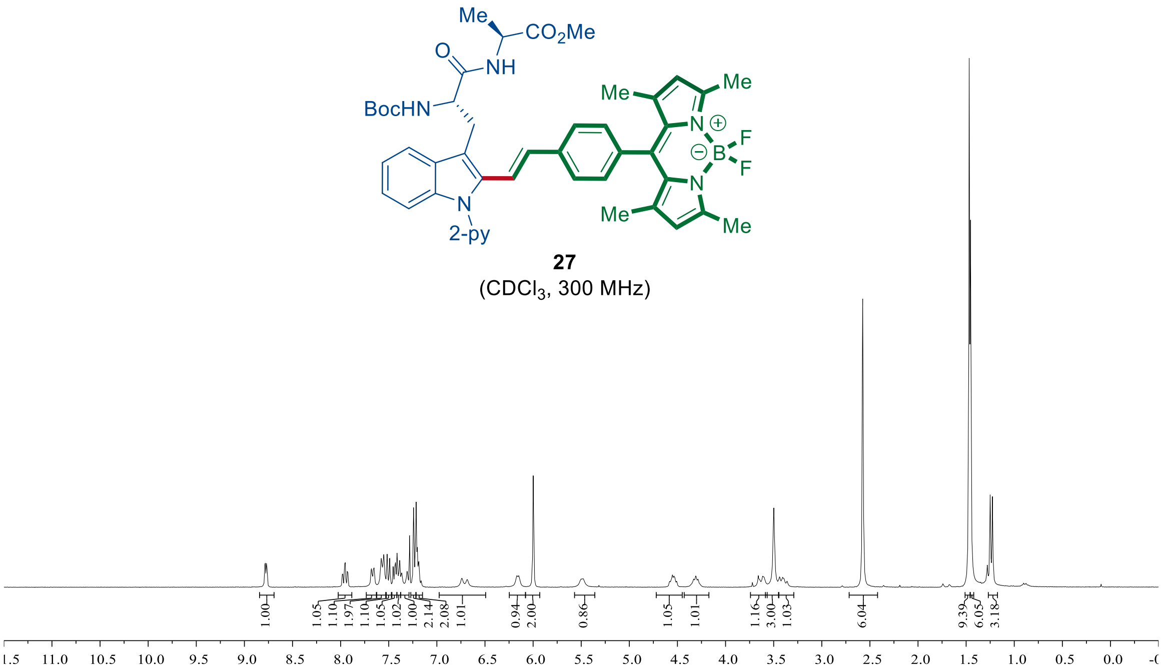

Supplementary Figure 102. ^{1}H-NMR spectrum of **27**.

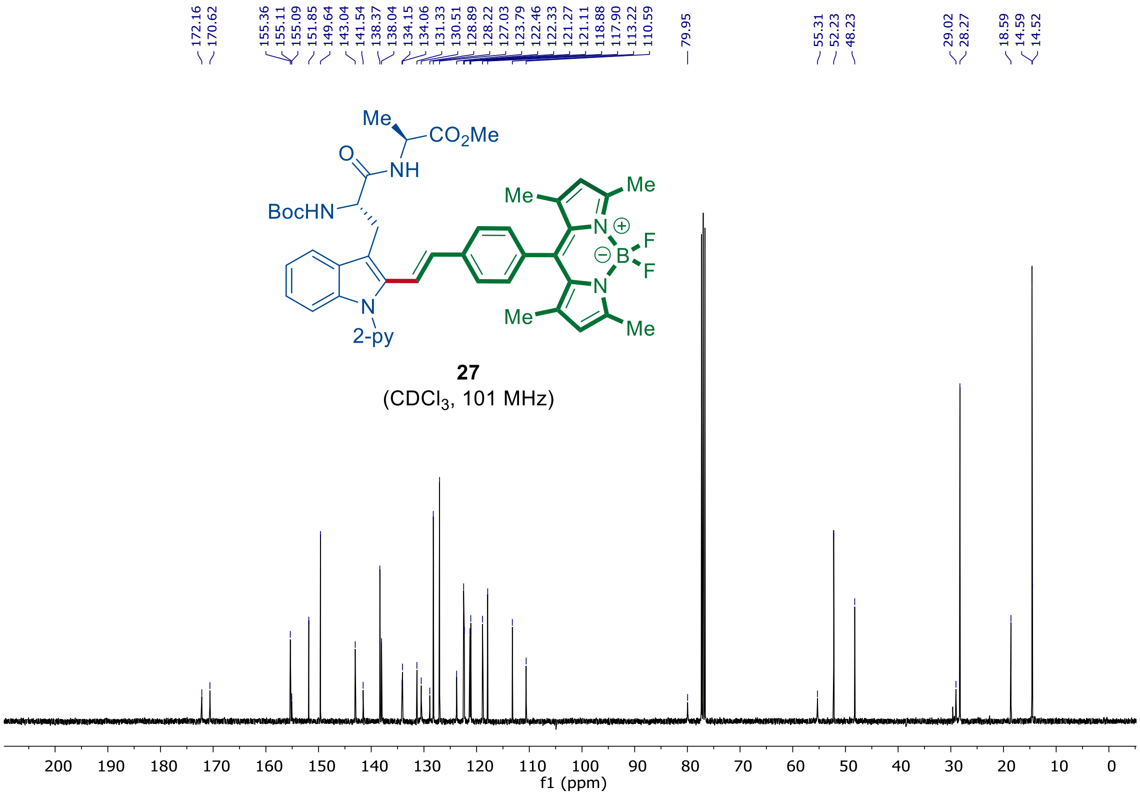

Supplementary Figure 103. ^{13}C-NMR spectrum of **27**.

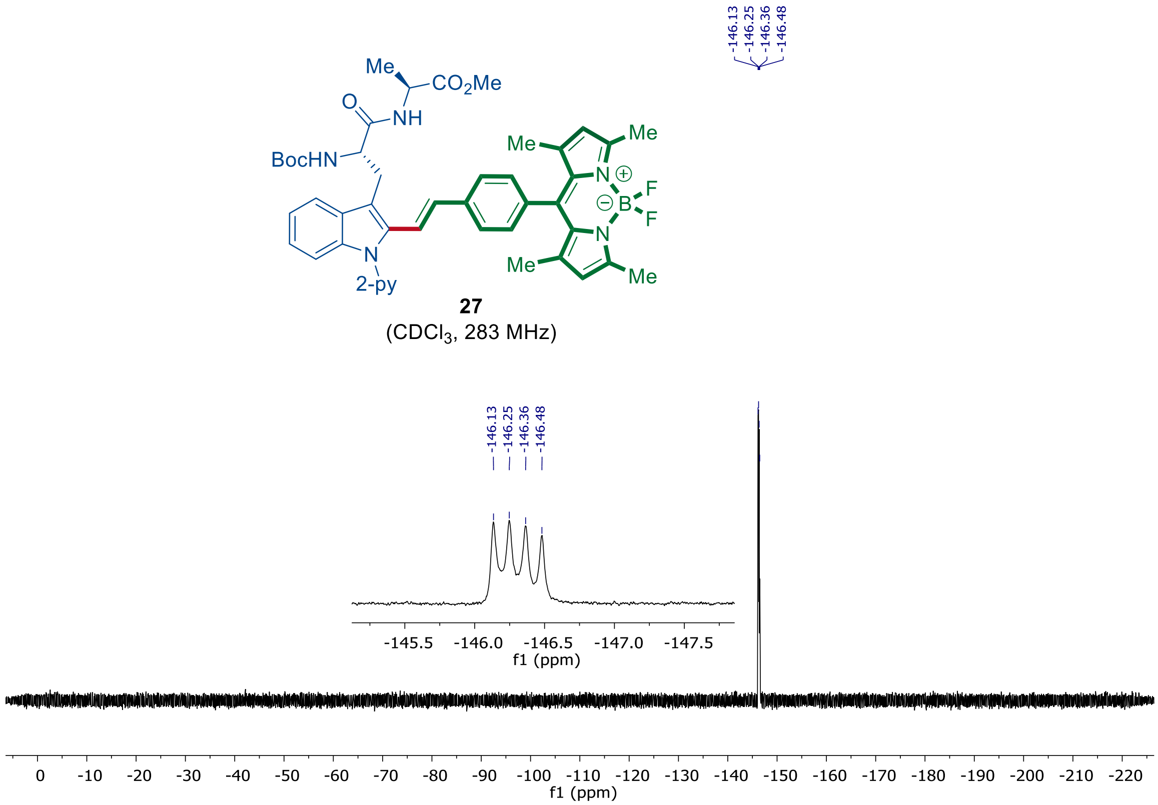

Supplementary Figure 104. ^{19}F-NMR spectrum of **27**.

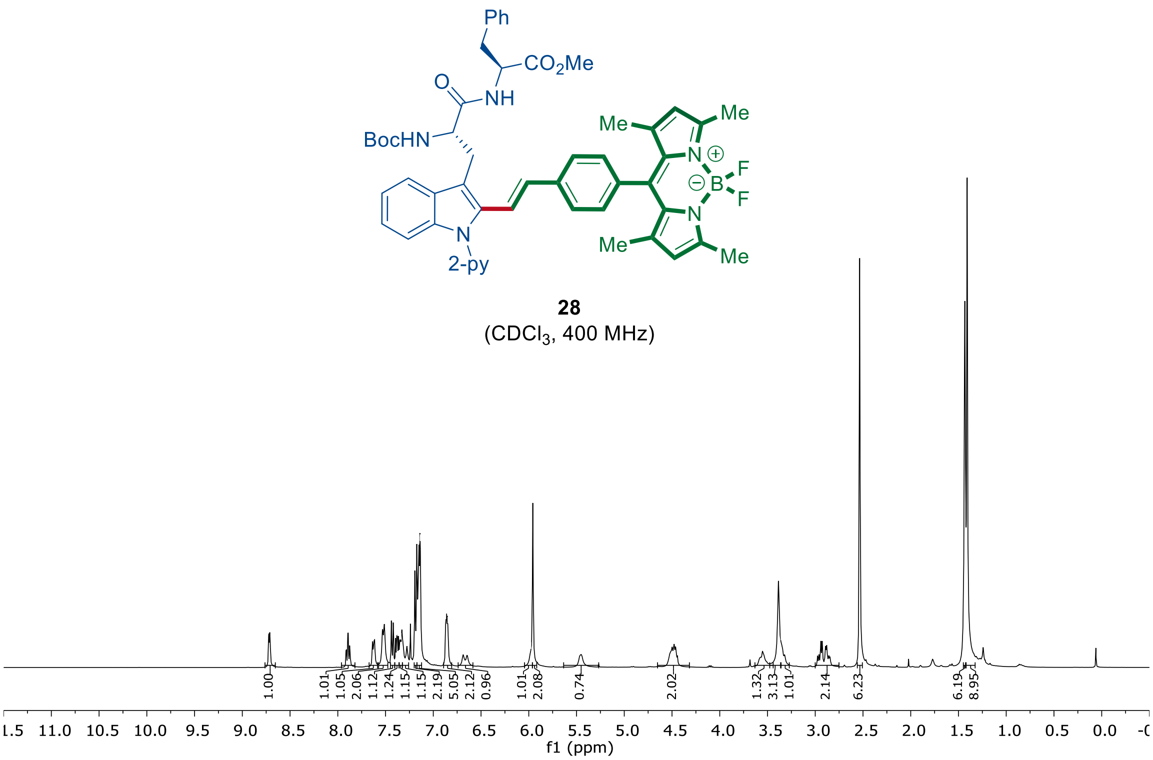

Supplementary Figure 105. ^{1}H-NMR spectrum of **28**.

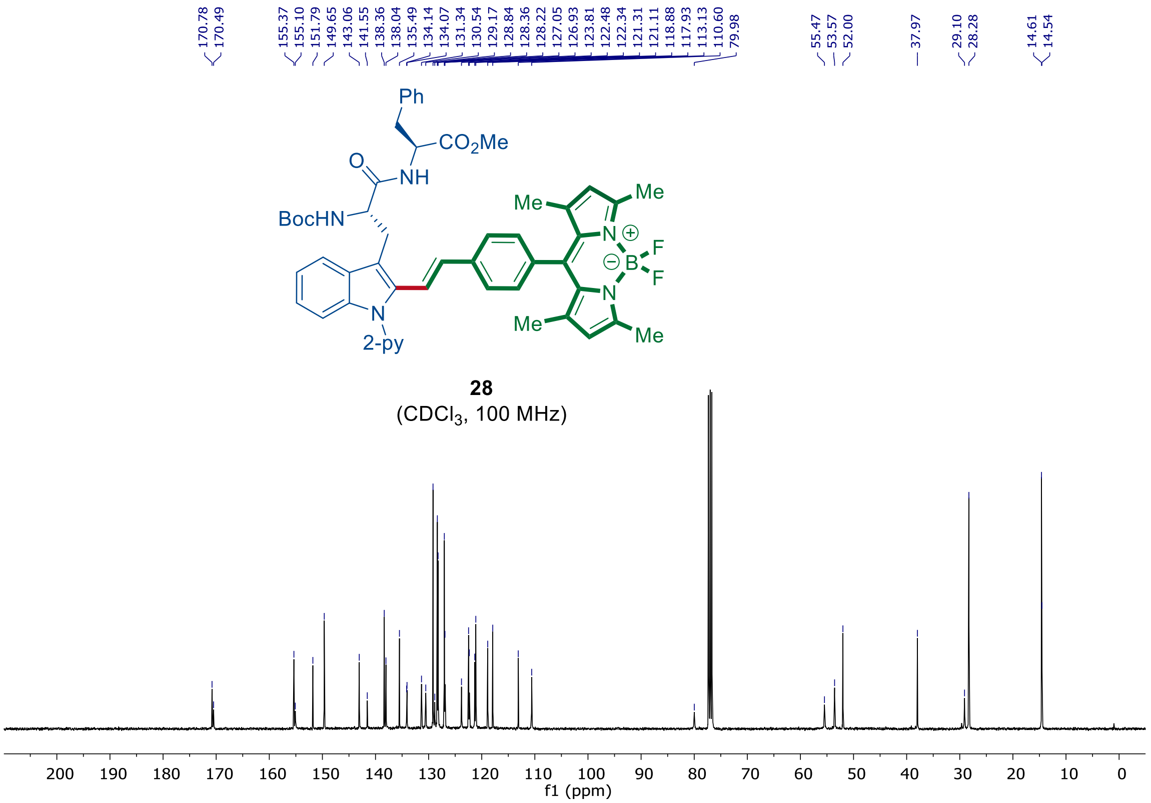

Supplementary Figure 106. ^{13}C-NMR spectrum of **28**.

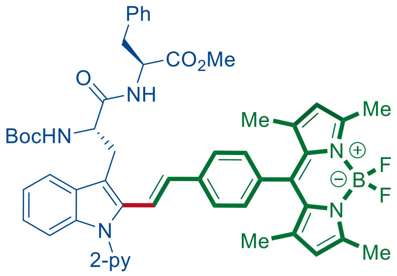

28
($CDCl_3$, 377 MHz)

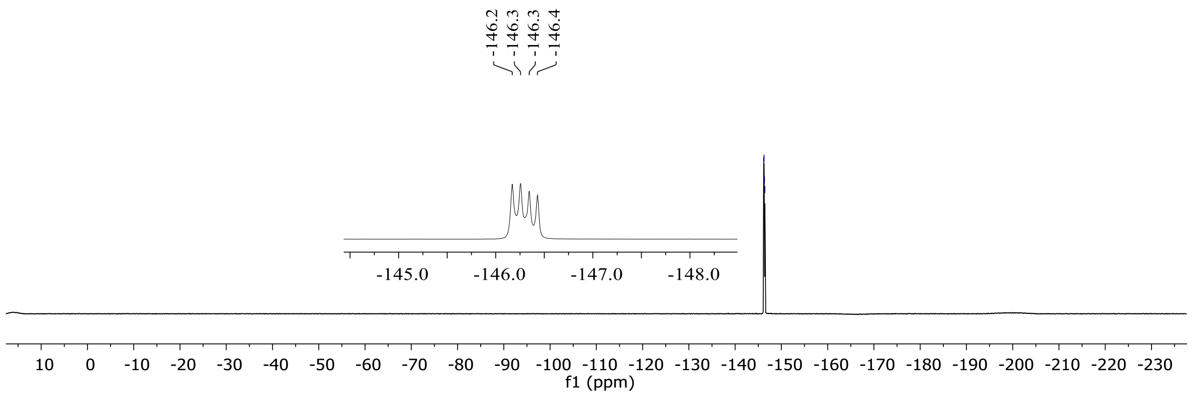

Supplementary Figure 107. ^{19}F-NMR spectrum of **28**.

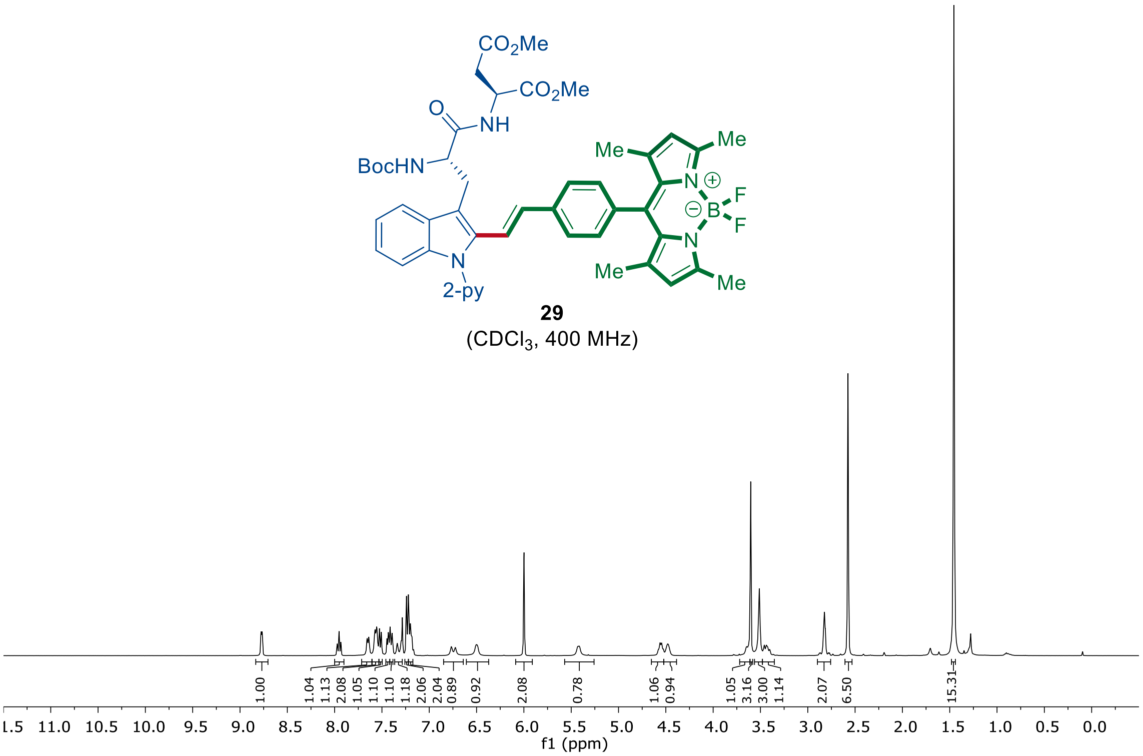

Supplementary Figure 108. ^{1}H-NMR spectrum of **29**.

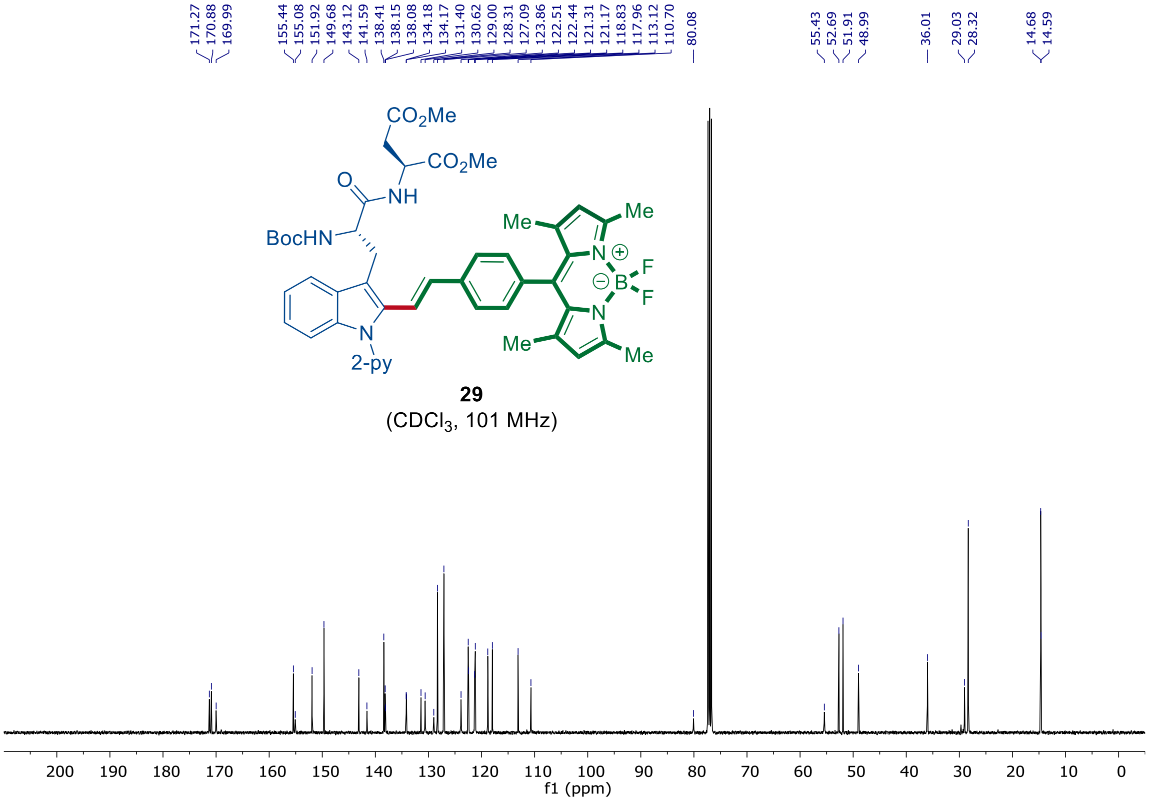

Supplementary Figure 109. ^{13}C-NMR spectrum of **29**.

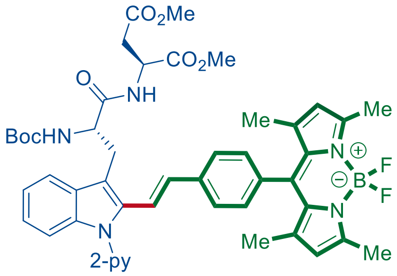

29
($CDCl_3$, 283 MHz)

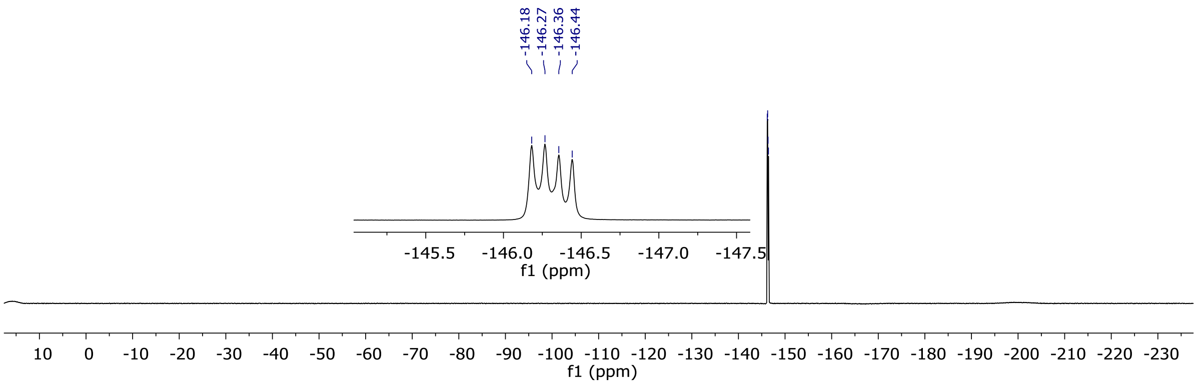

Supplementary Figure 110. ^{19}F-NMR spectrum of **29**.

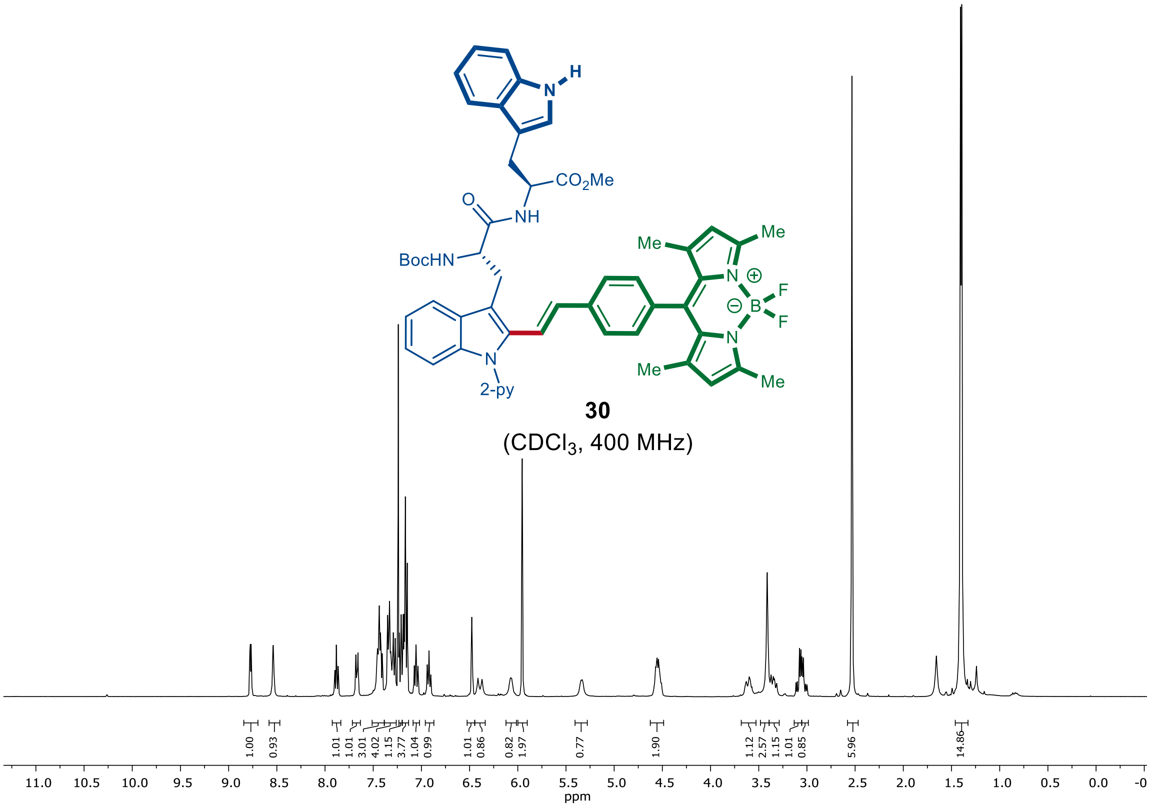

Supplementary Figure 111. ^{1}H-NMR spectrum of **30**.

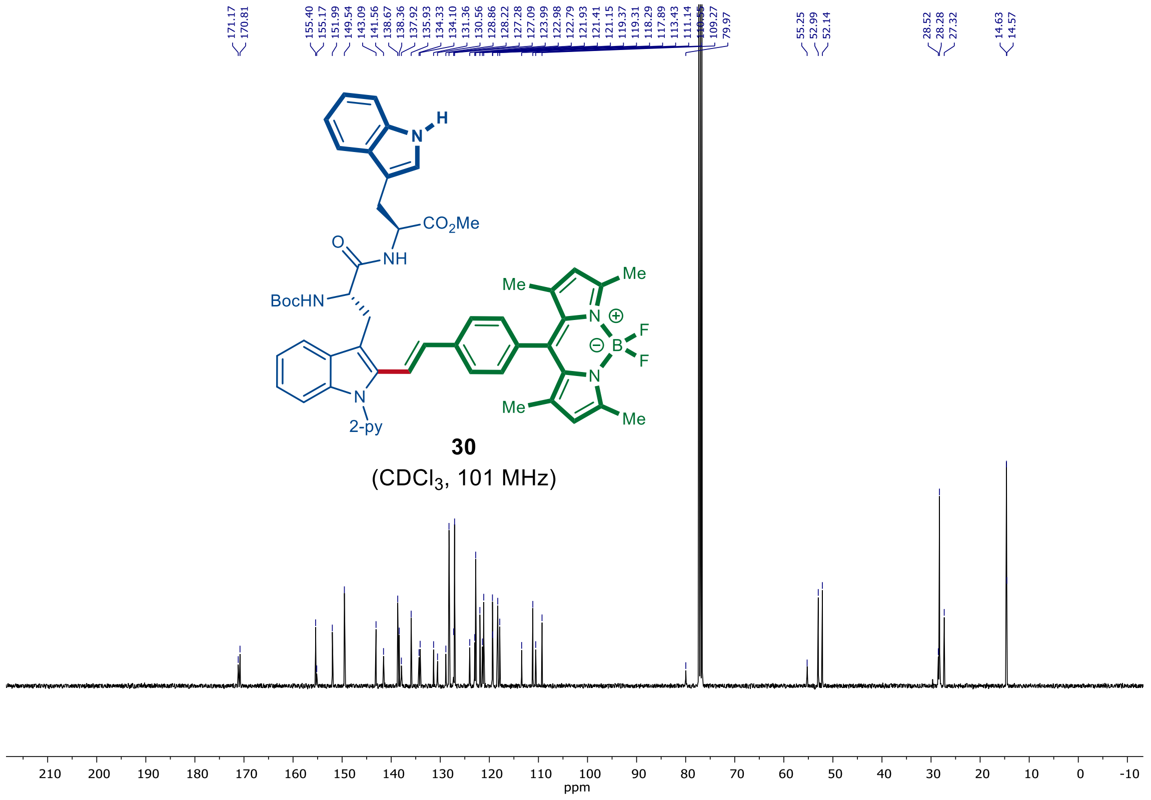

Supplementary Figure 112. ^{13}C-NMR spectrum of **30**.

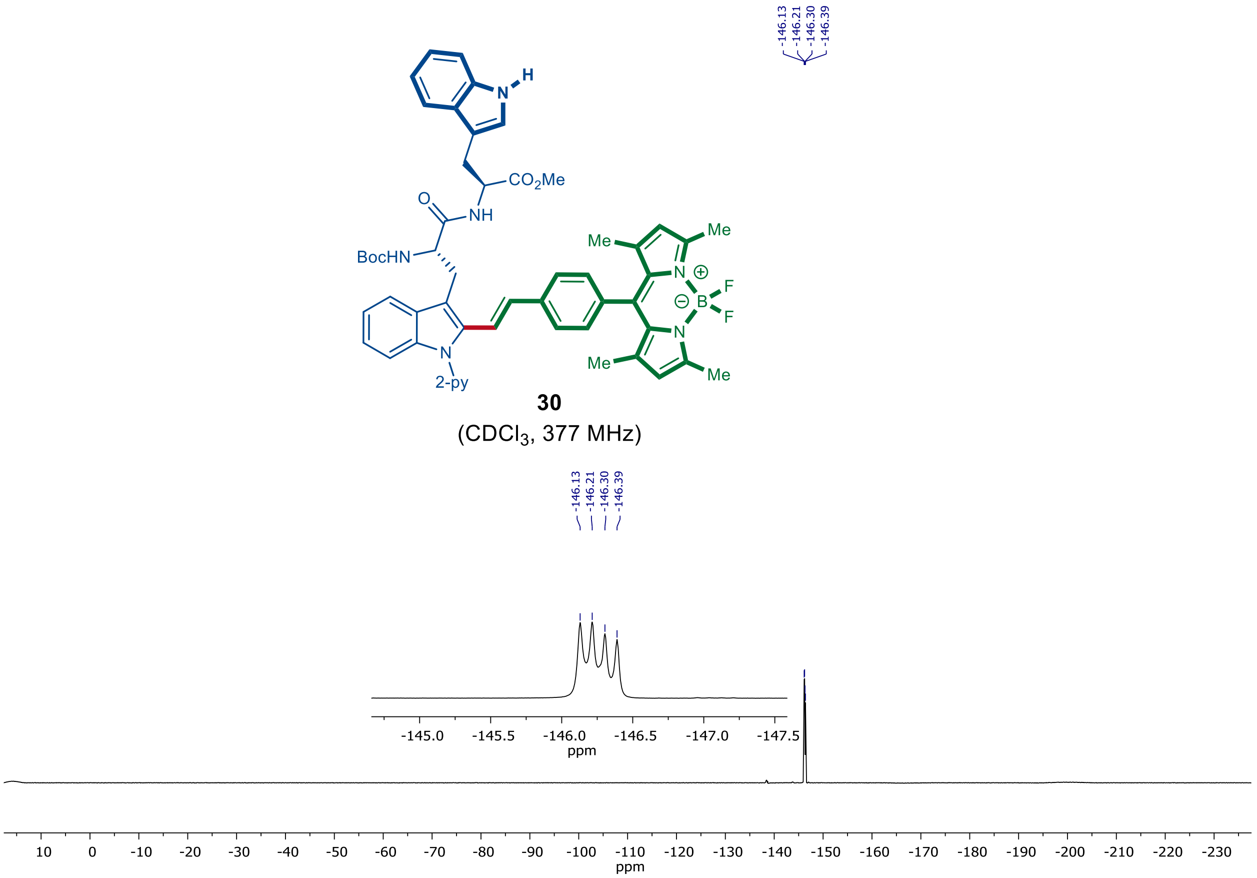

Supplementary Figure 113. ^{19}F-NMR spectrum of **30**.

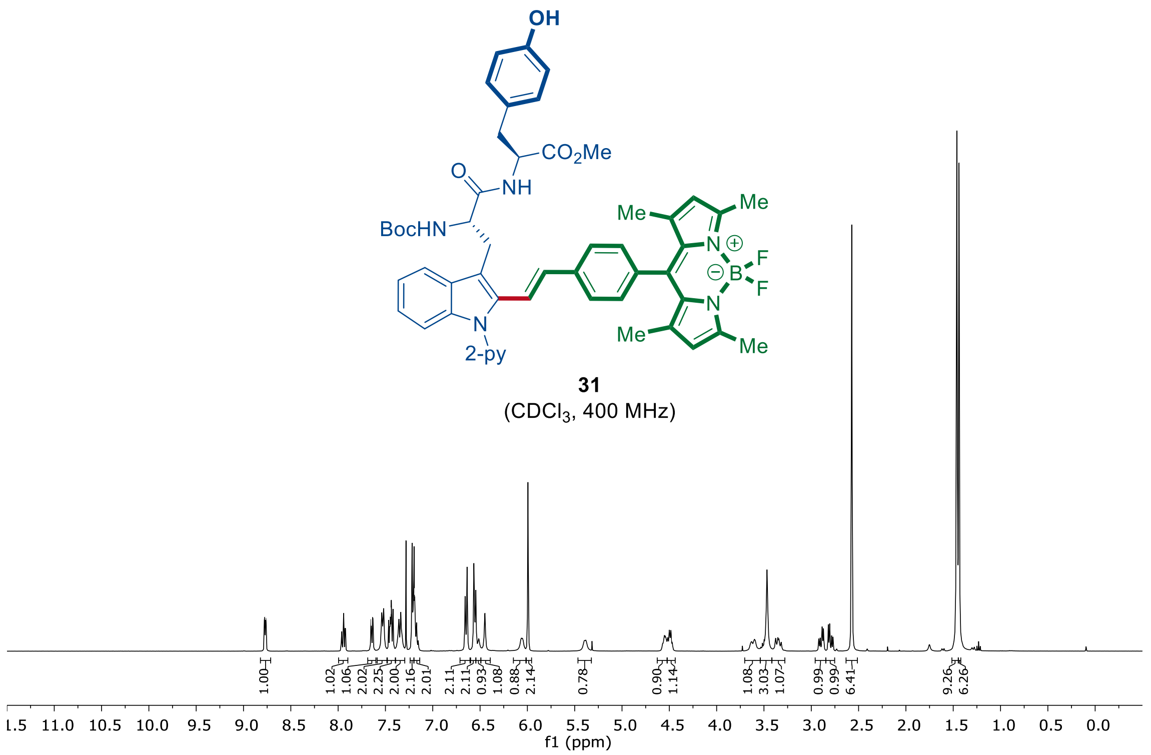

Supplementary Figure 114. ^{1}H-NMR spectrum of **31**.

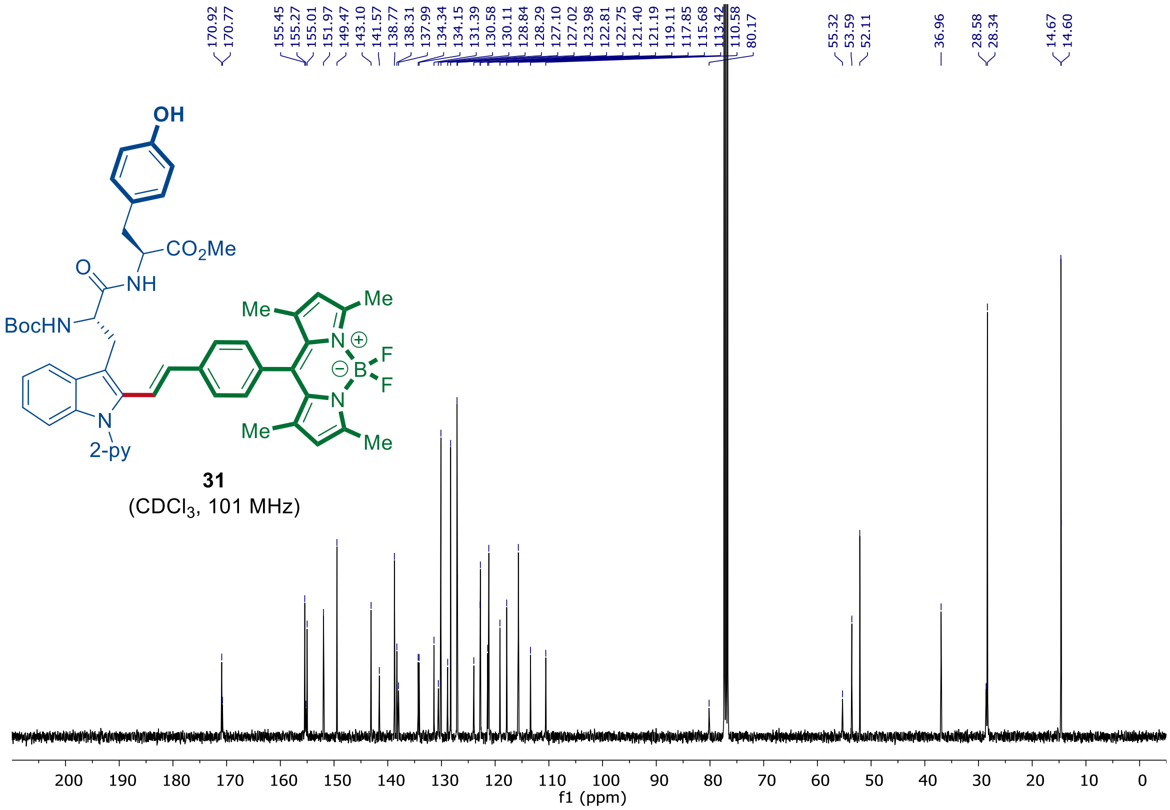

Supplementary Figure 115. ^{13}C-NMR spectrum of **31**.

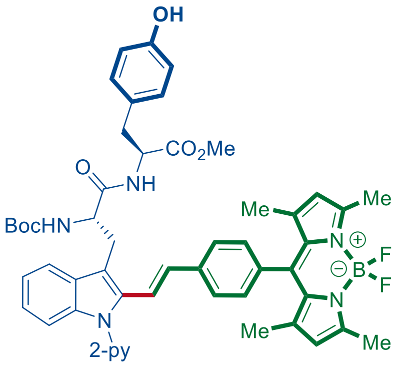

31
($CDCl_3$, 377 MHz)

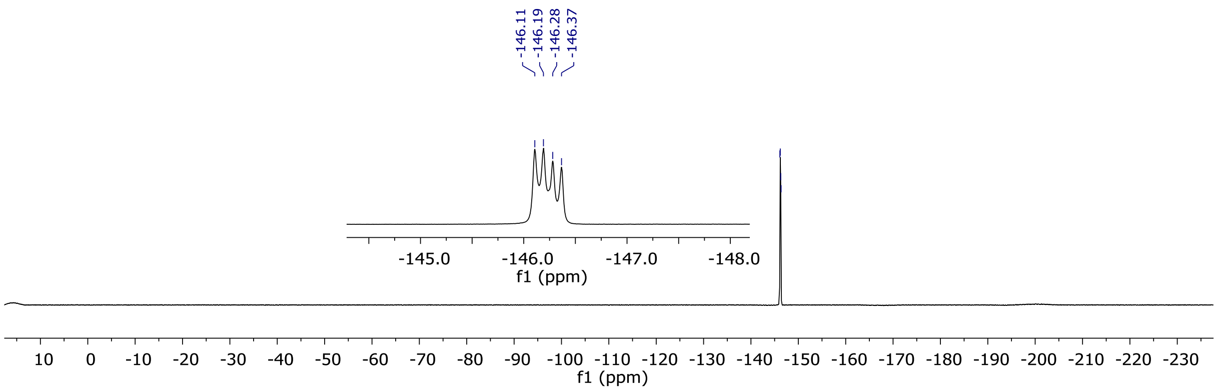

Supplementary Figure 116. ^{19}F-NMR spectrum of **31**.

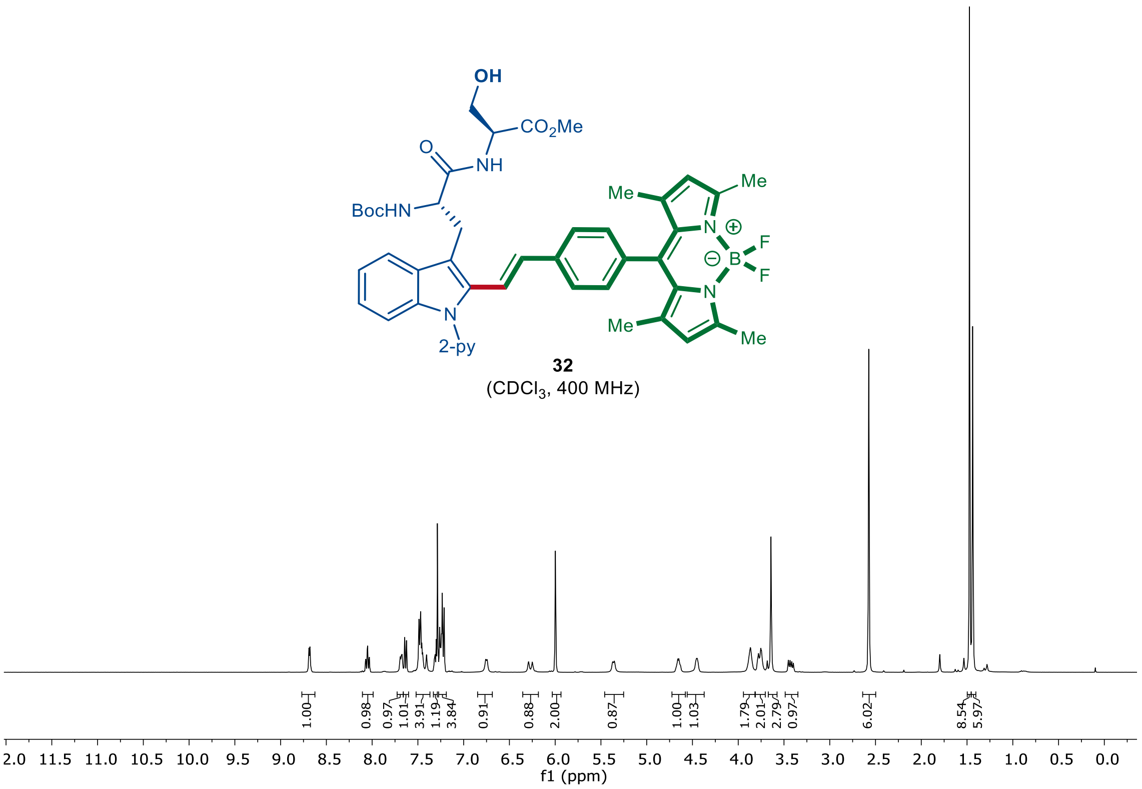

Supplementary Figure 117. ^{1}H-NMR spectrum of **32**.

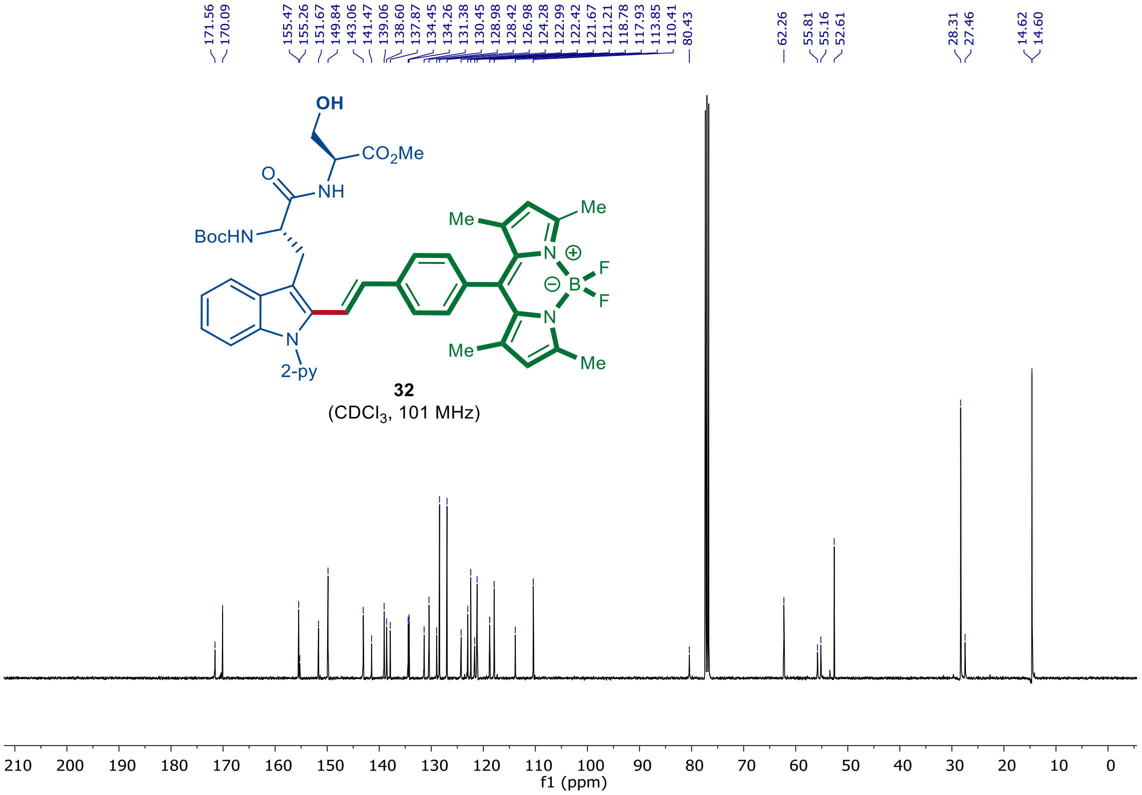

Supplementary Figure 118. ^{13}C-NMR spectrum of **32**.

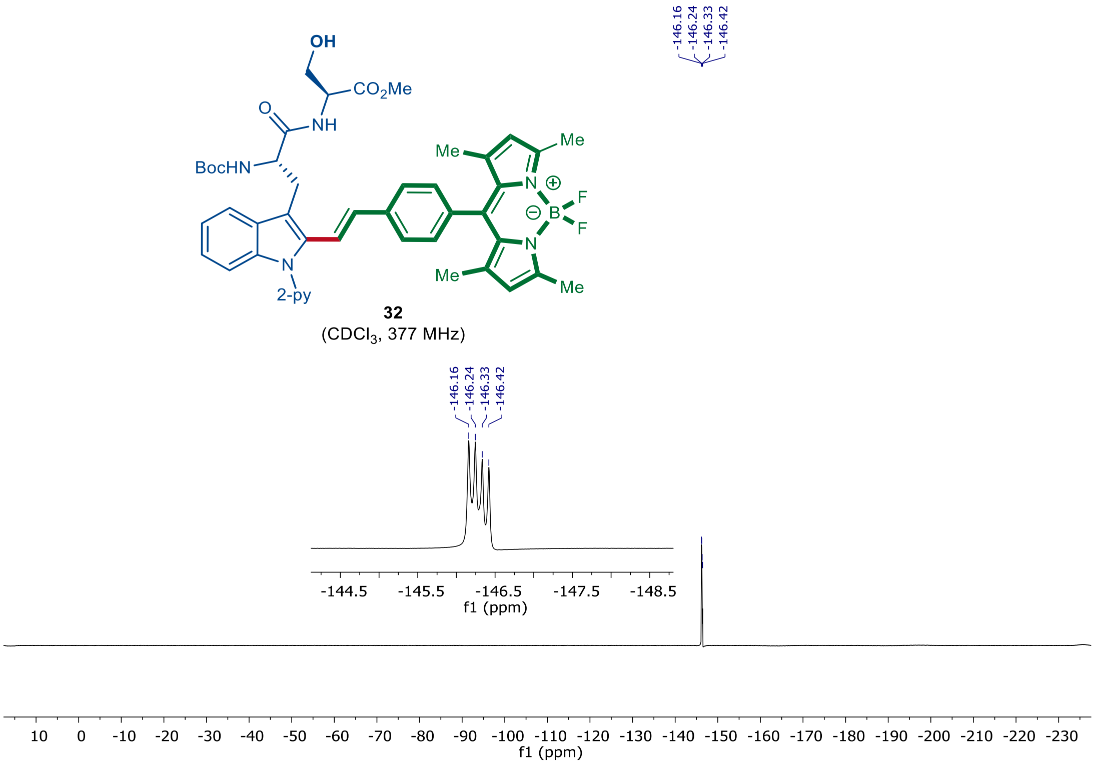

Supplementary Figure 119. ^{19}F-NMR spectrum of **32**.

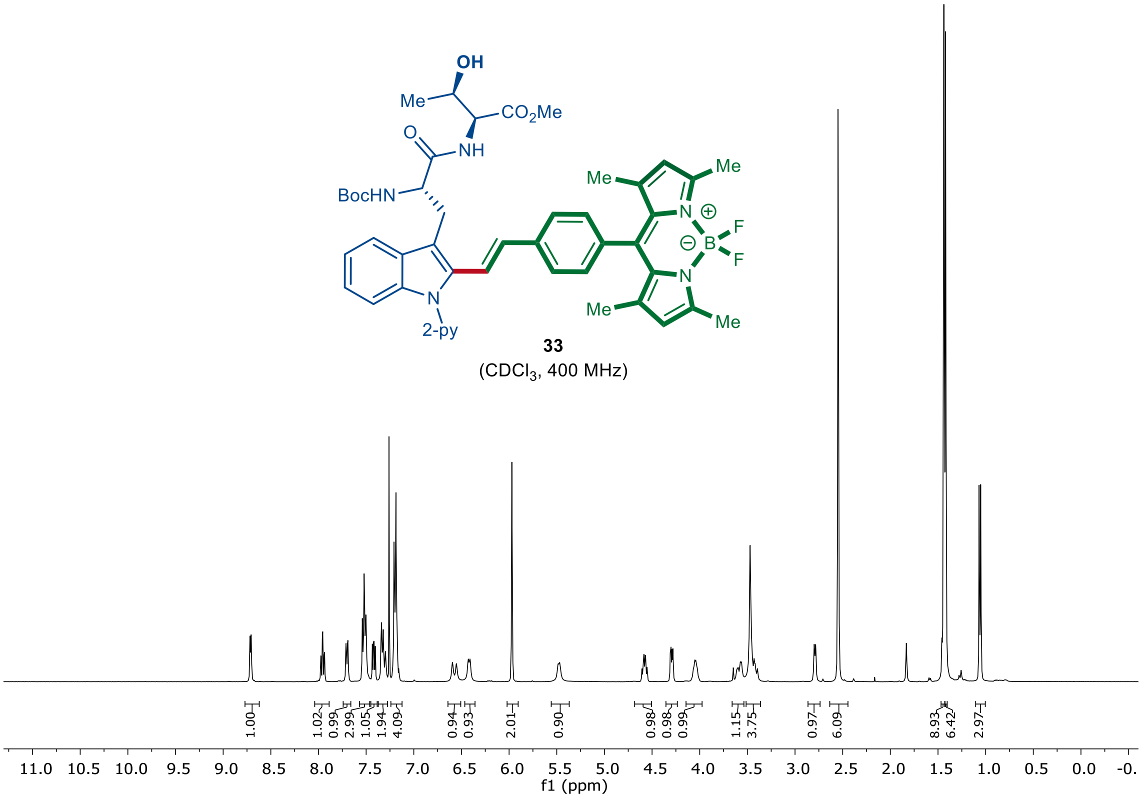

Supplementary Figure 120. ^{1}H-NMR spectrum of **33**.

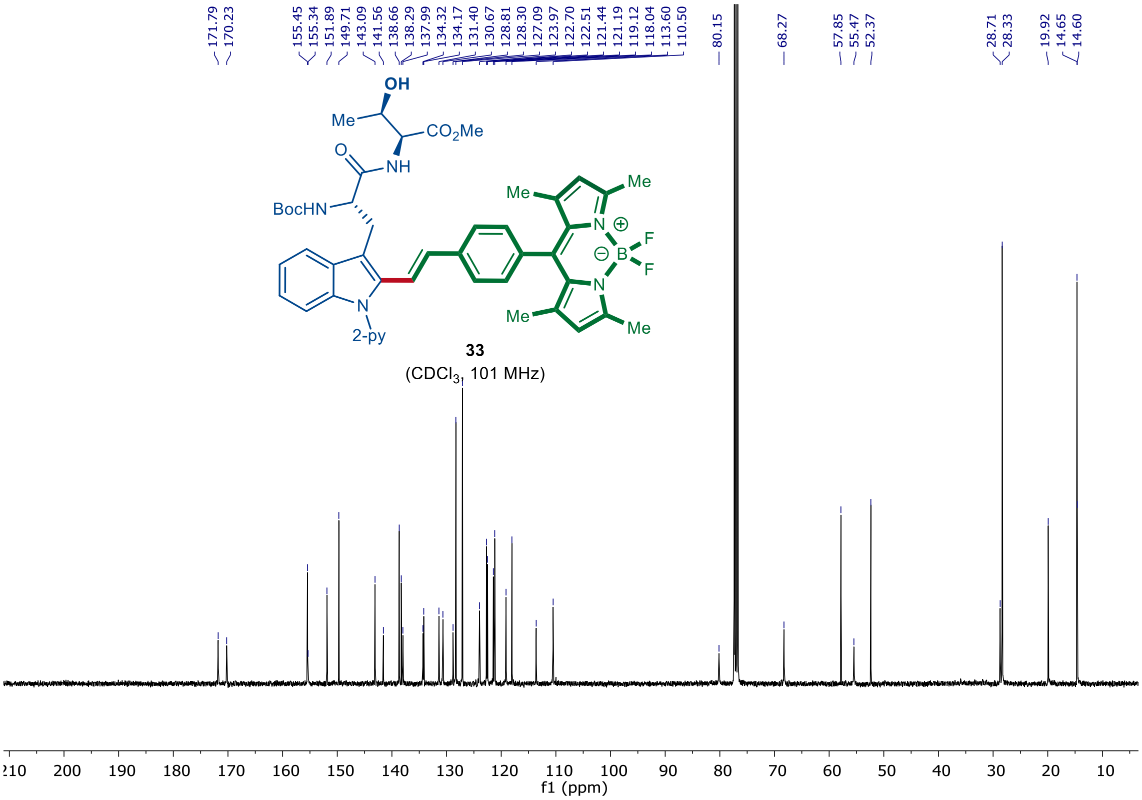

Supplementary Figure 121. ^{13}C-NMR spectrum of **33**.

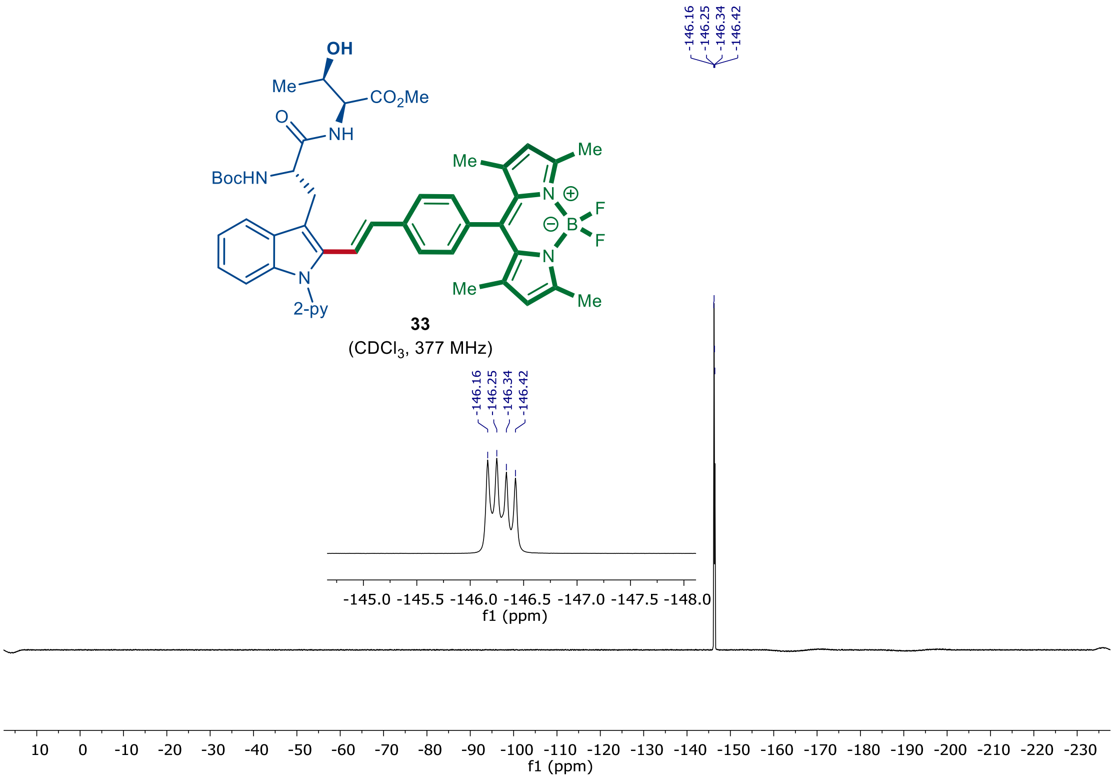

Supplementary Figure 122. ^{19}F-NMR spectrum of **33**.

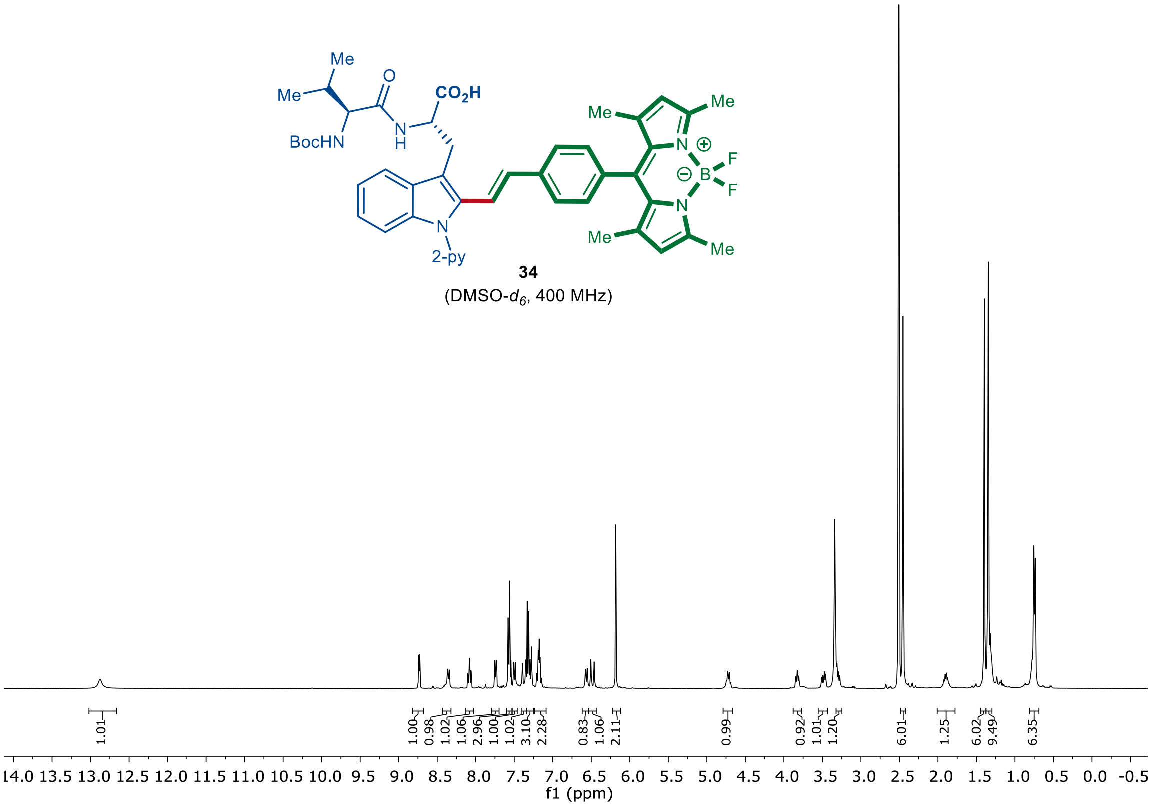

Supplementary Figure 123. ^{1}H-NMR spectrum of **34**.

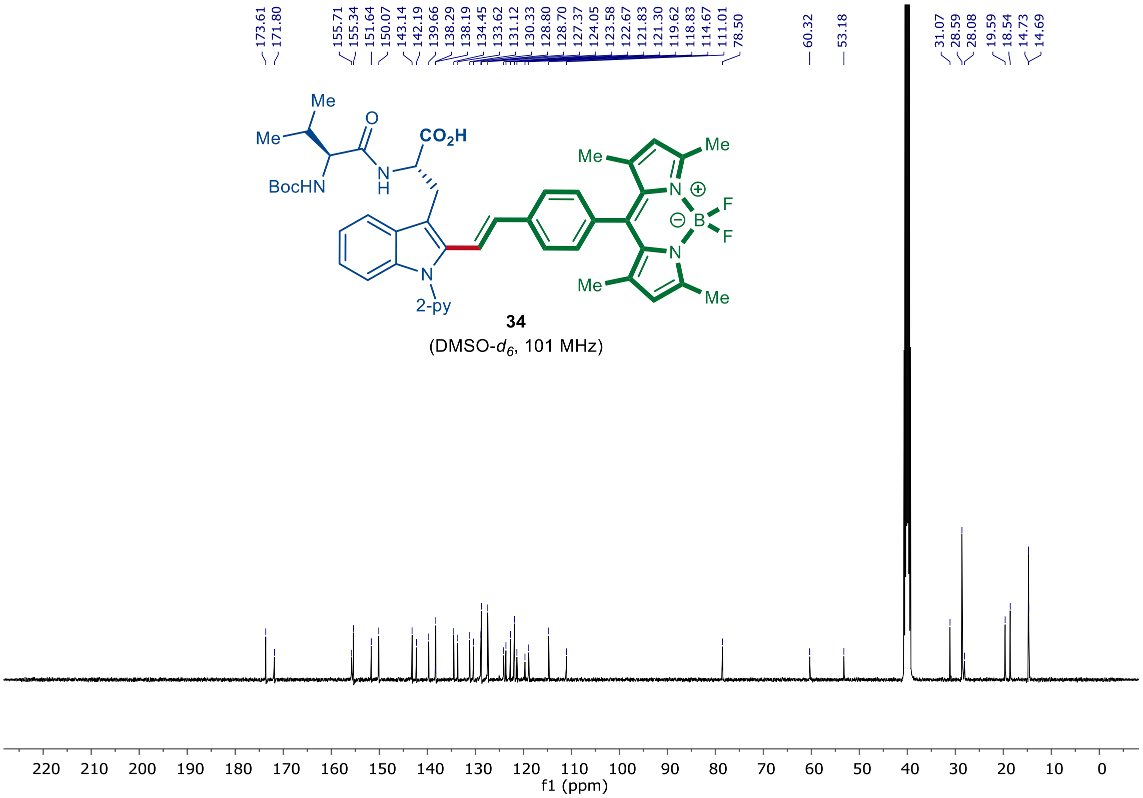

Supplementary Figure 124. ^{13}C-NMR spectrum of **34**.

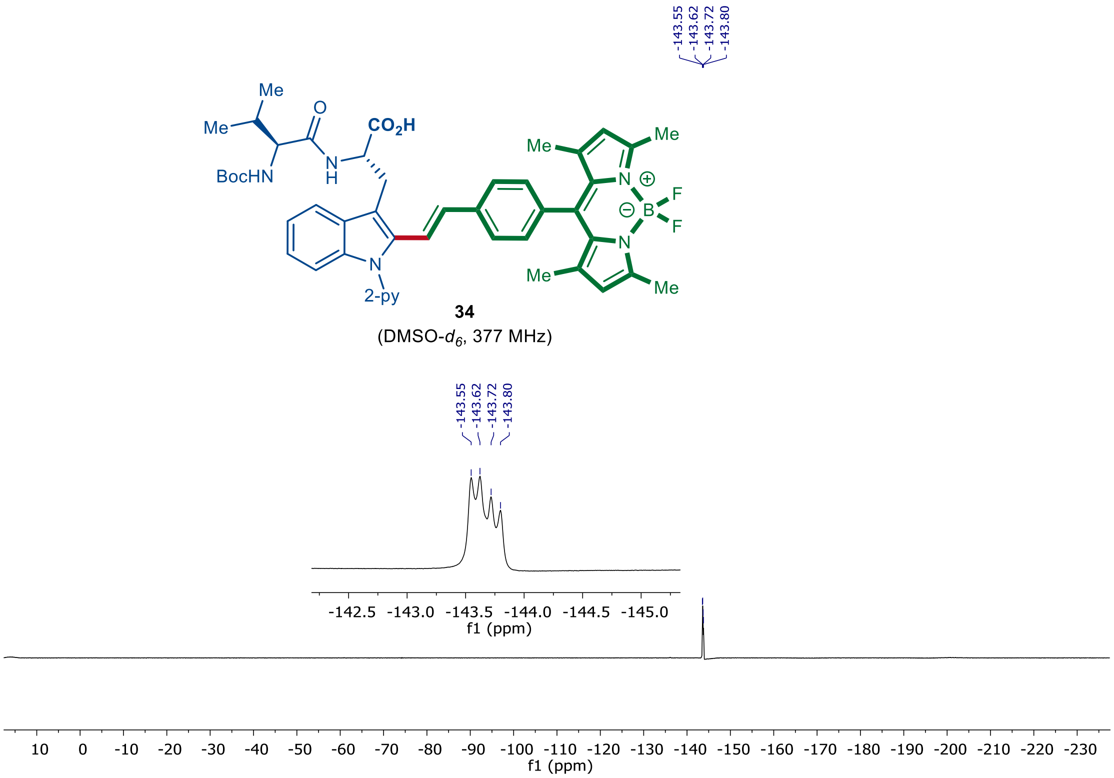

Supplementary Figure 125. ^{19}F-NMR spectrum of **34**.

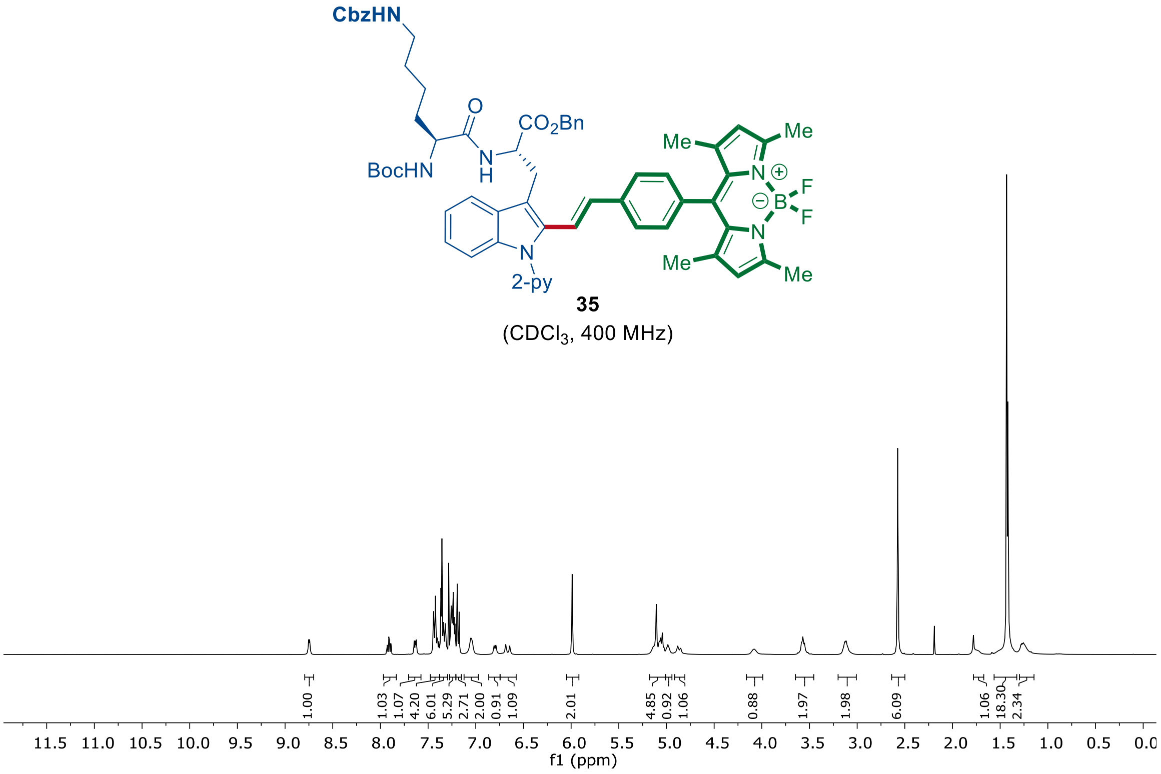

Supplementary Figure 126. ^{1}H-NMR spectrum of **35**.

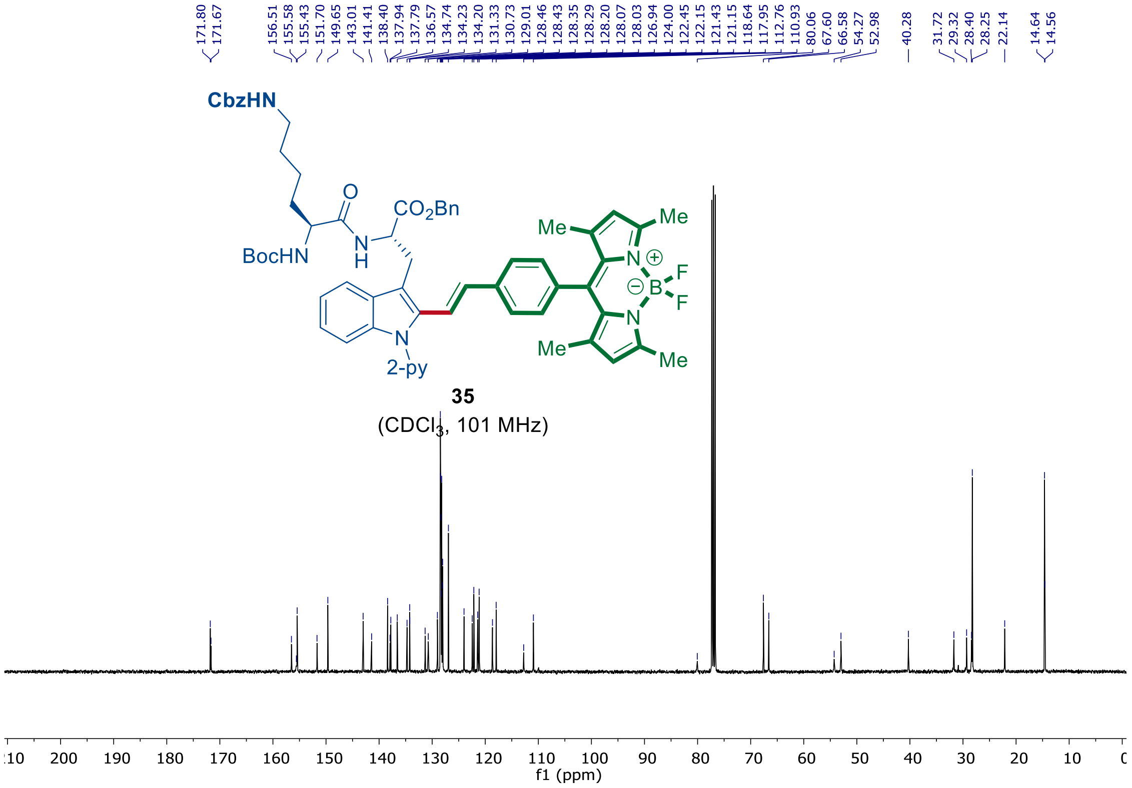

Supplementary Figure 127. ^{13}C-NMR spectrum of **35**.

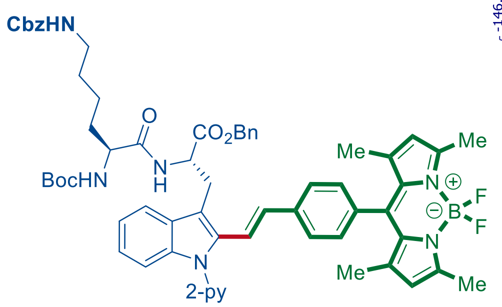

35
($CDCl_3$, 377 MHz)

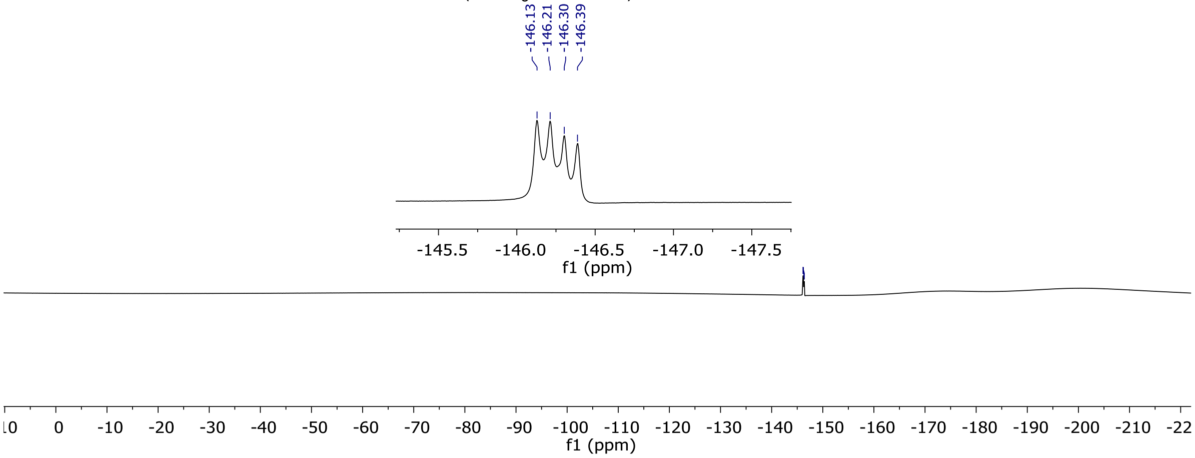

Supplementary Figure 128. ^{19}F-NMR spectrum of **35**.

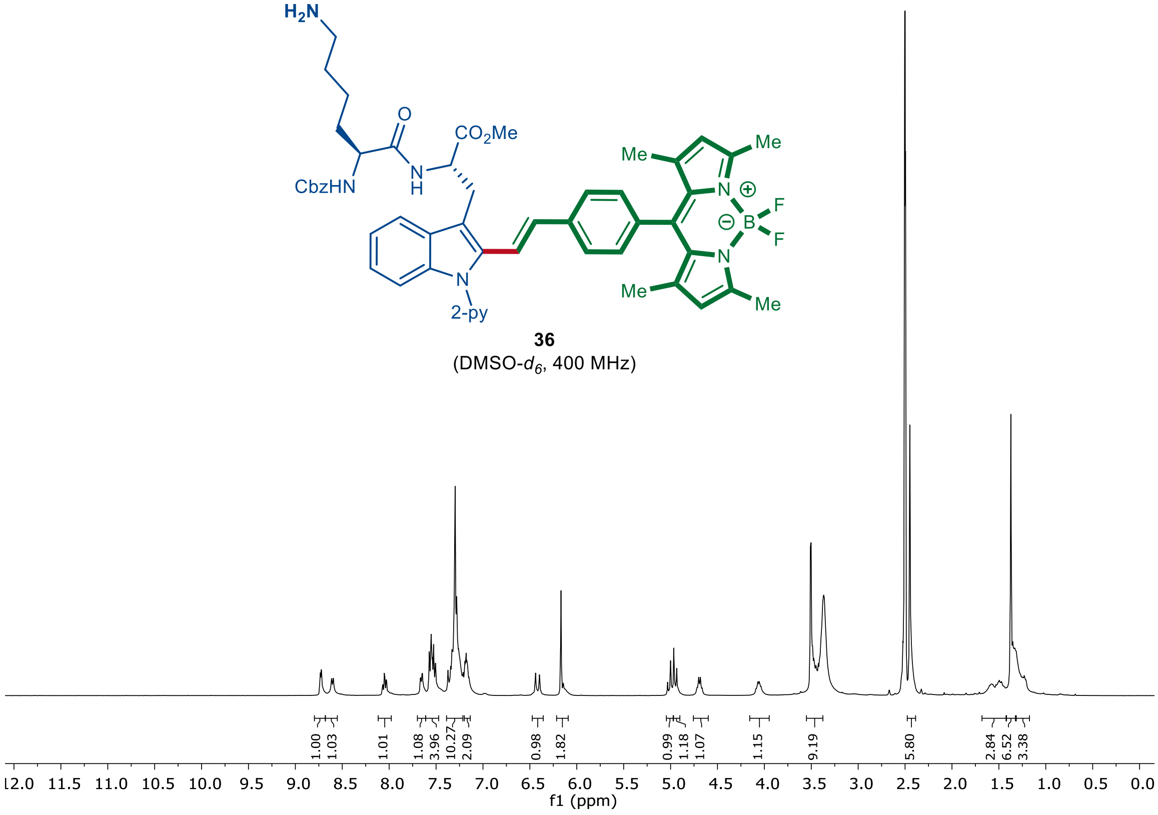

Supplementary Figure 129. ^{1}H-NMR spectrum of **36**.

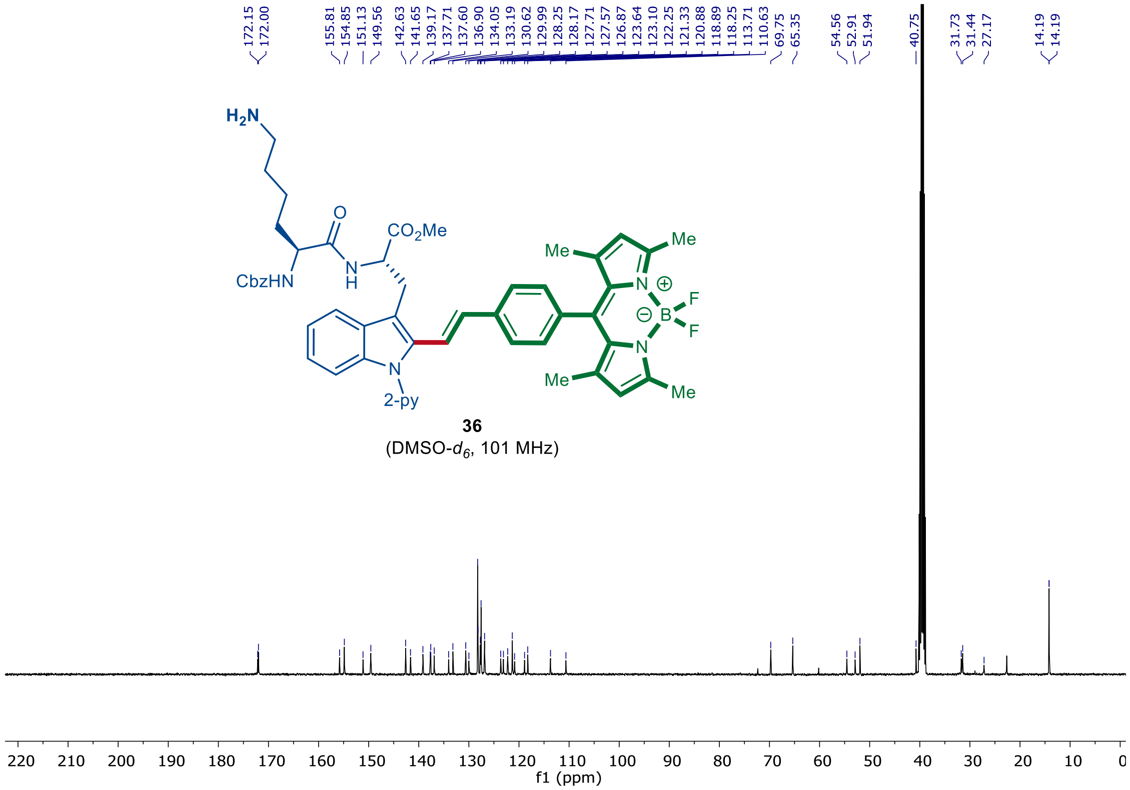

Supplementary Figure 130. ^{13}C-NMR spectrum of **36**.

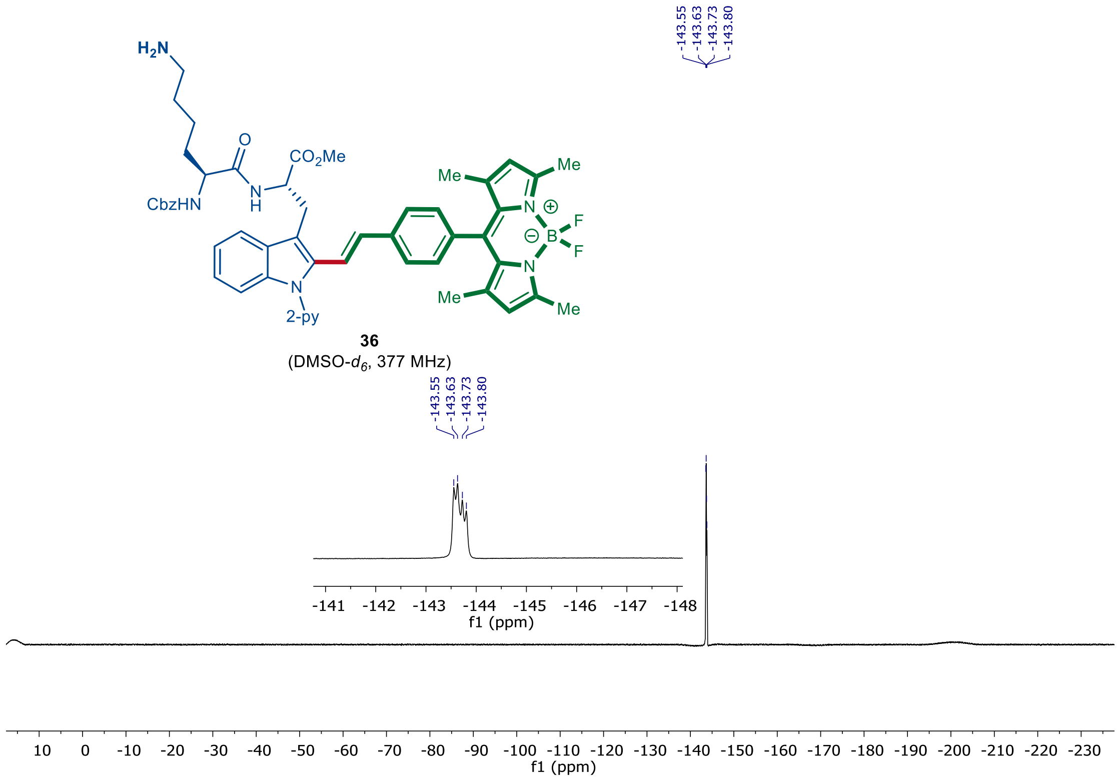

Supplementary Figure 131. ^{19}F-NMR spectrum of **36**.

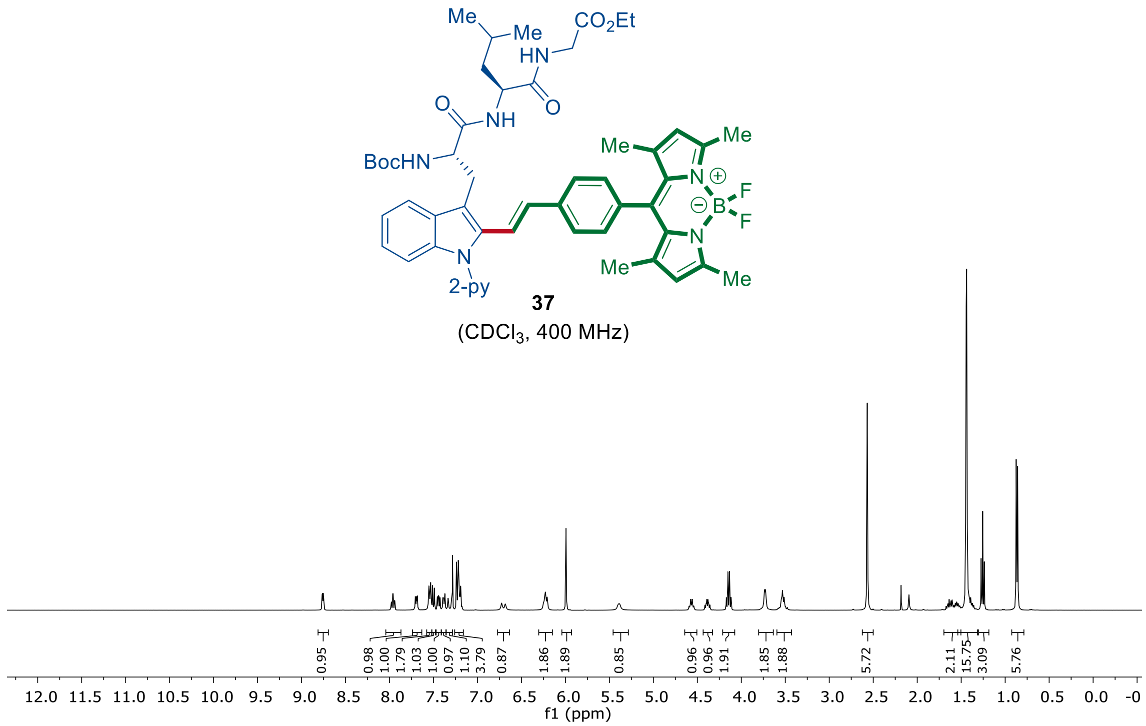

Supplementary Figure 132. ^{1}H-NMR spectrum of **37**.

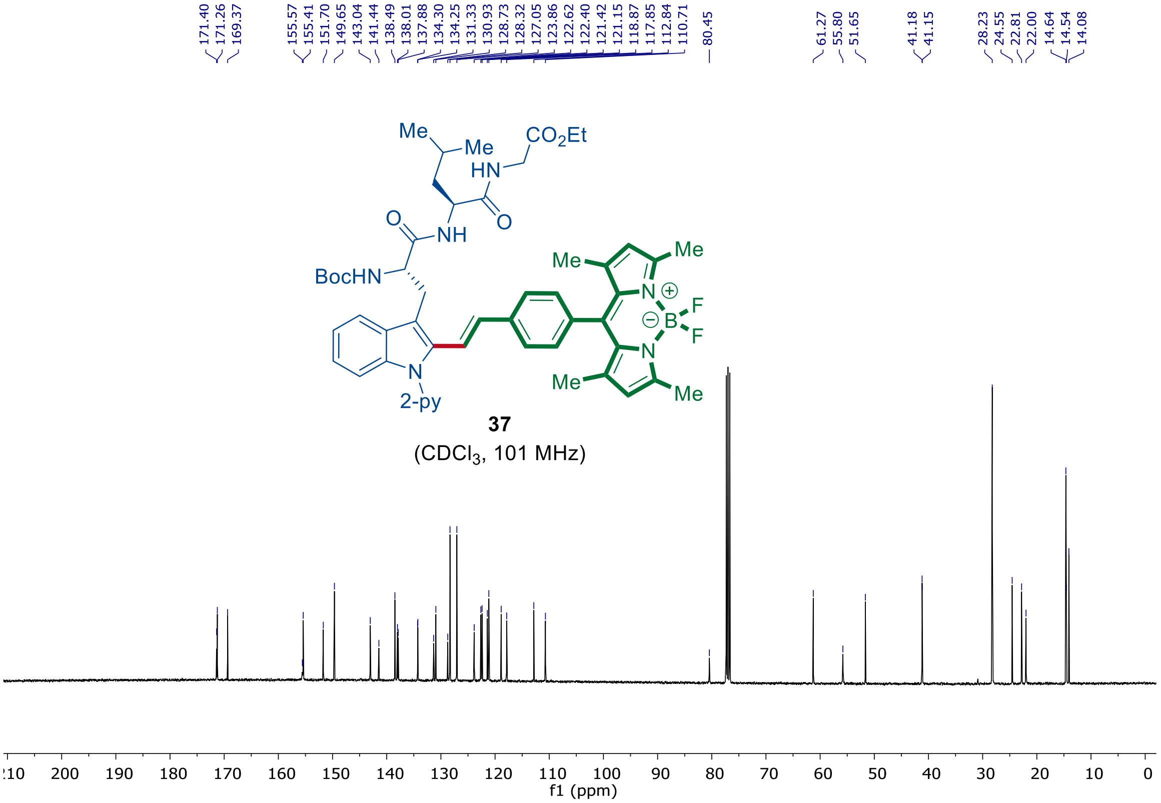

Supplementary Figure 133. ^{13}C-NMR spectrum of **37**.

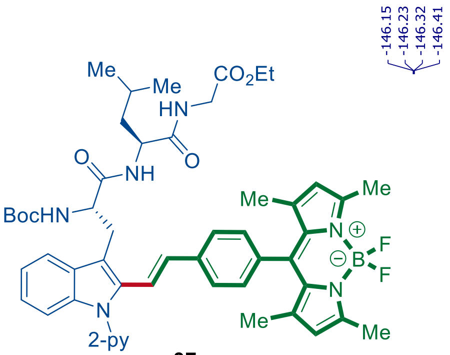

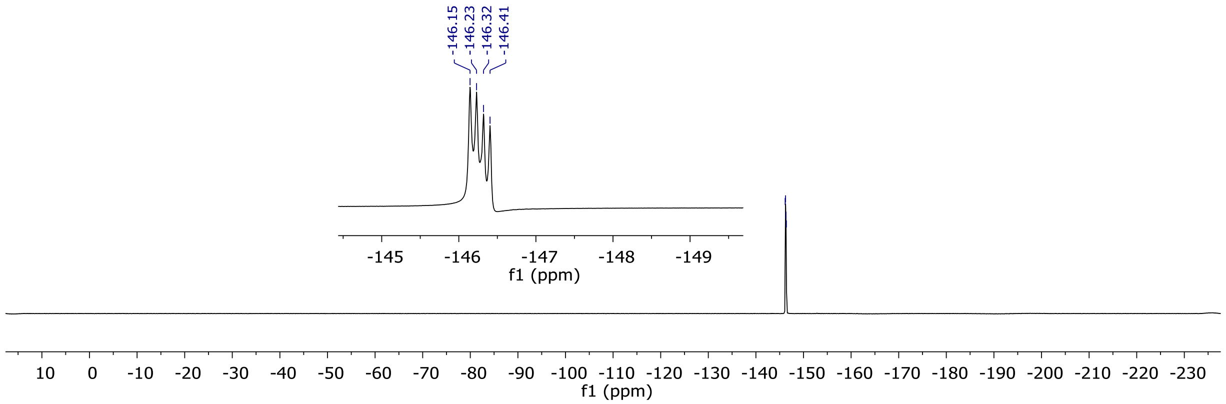

Supplementary Figure 134. ^{19}F-NMR spectrum of **37**.

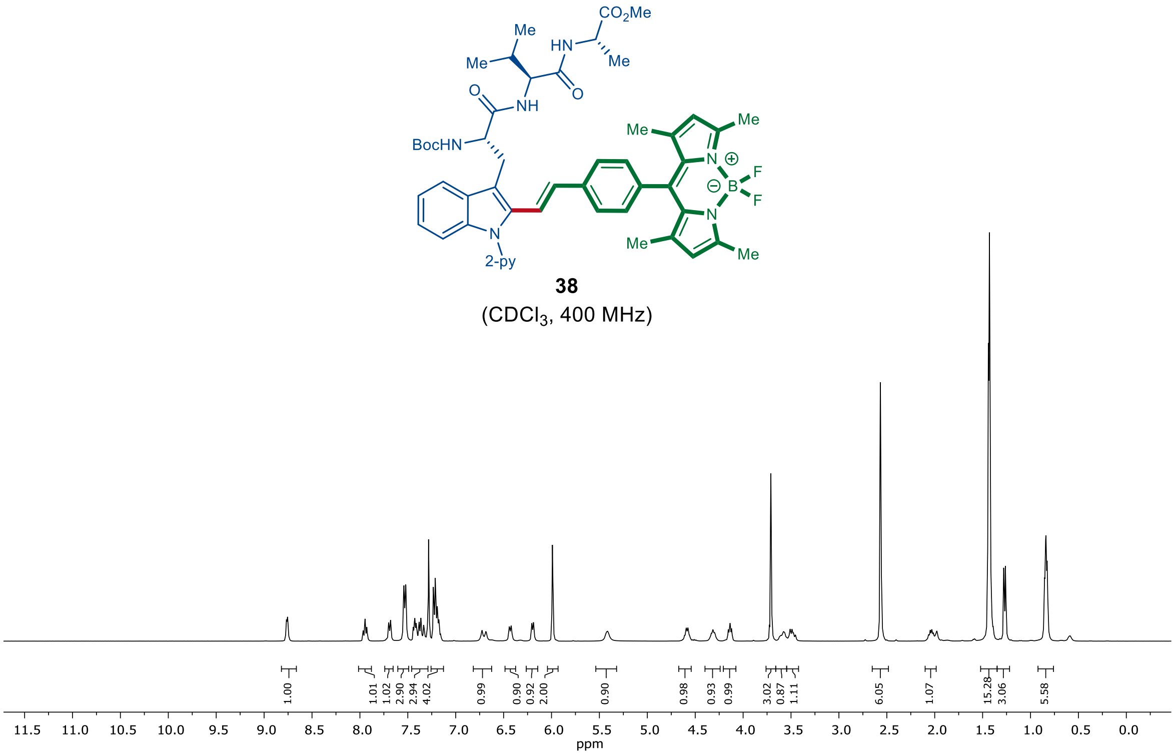

Supplementary Figure 135. ^{1}H-NMR spectrum of **38**.

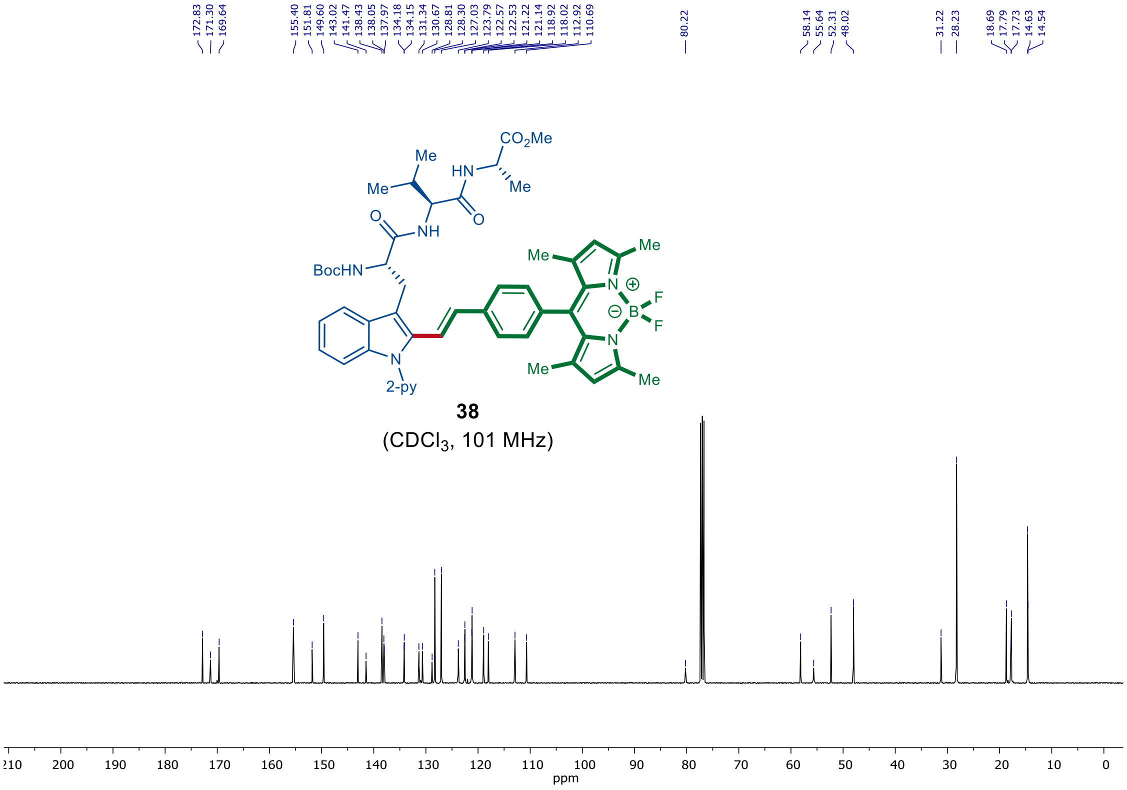

Supplementary Figure 136. ^{13}C-NMR spectrum of **38**.

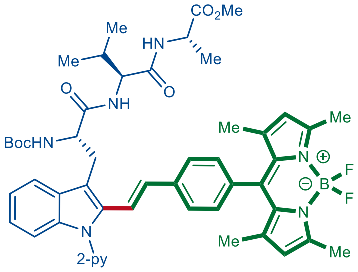

38

($CDCl_3$, 377 MHz)

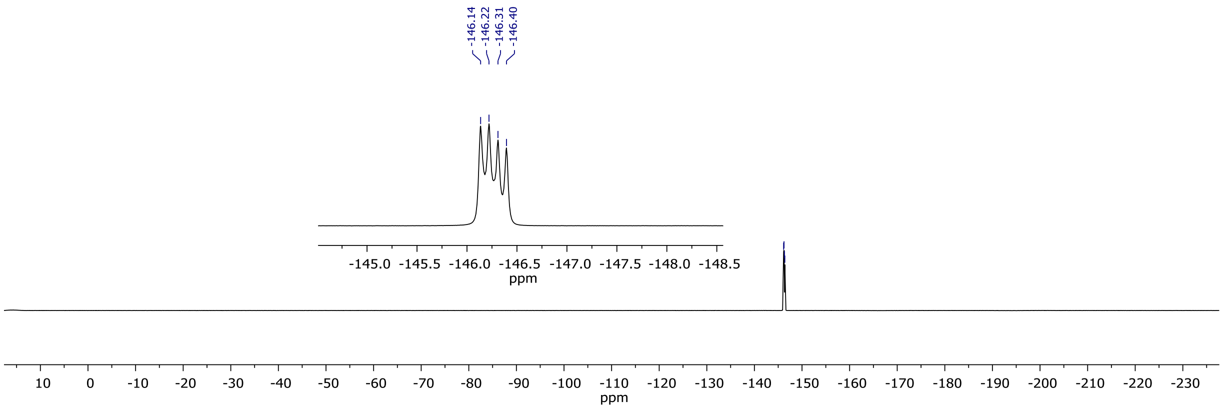

Supplementary Figure 137. ^{19}F-NMR spectrum of **38**.

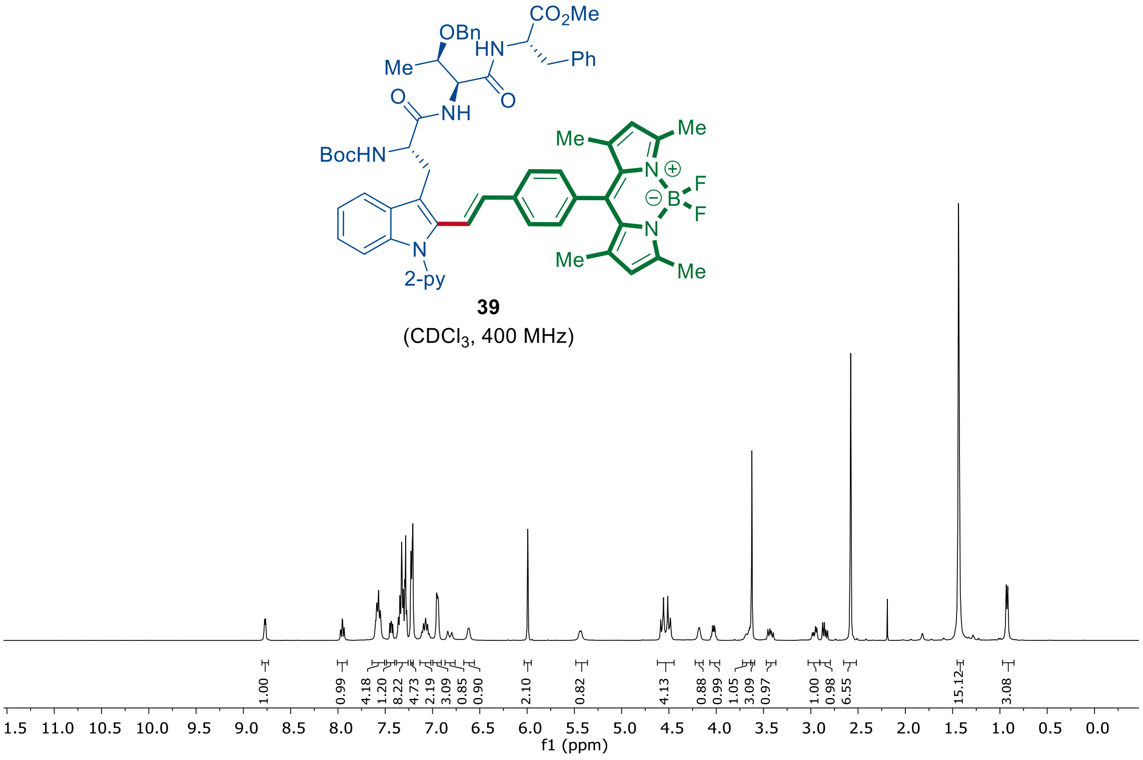

Supplementary Figure 138. ^{1}H-NMR spectrum of **39**.

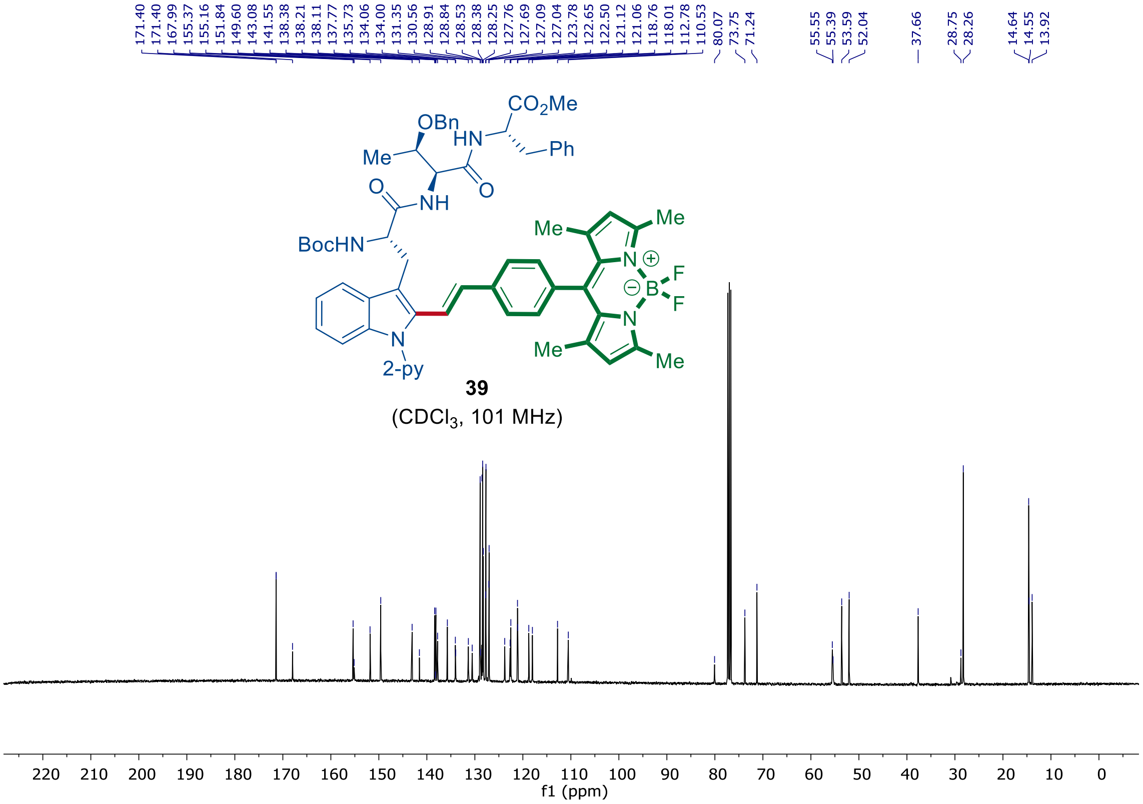

Supplementary Figure 139. ^{13}C-NMR spectrum of **39**.

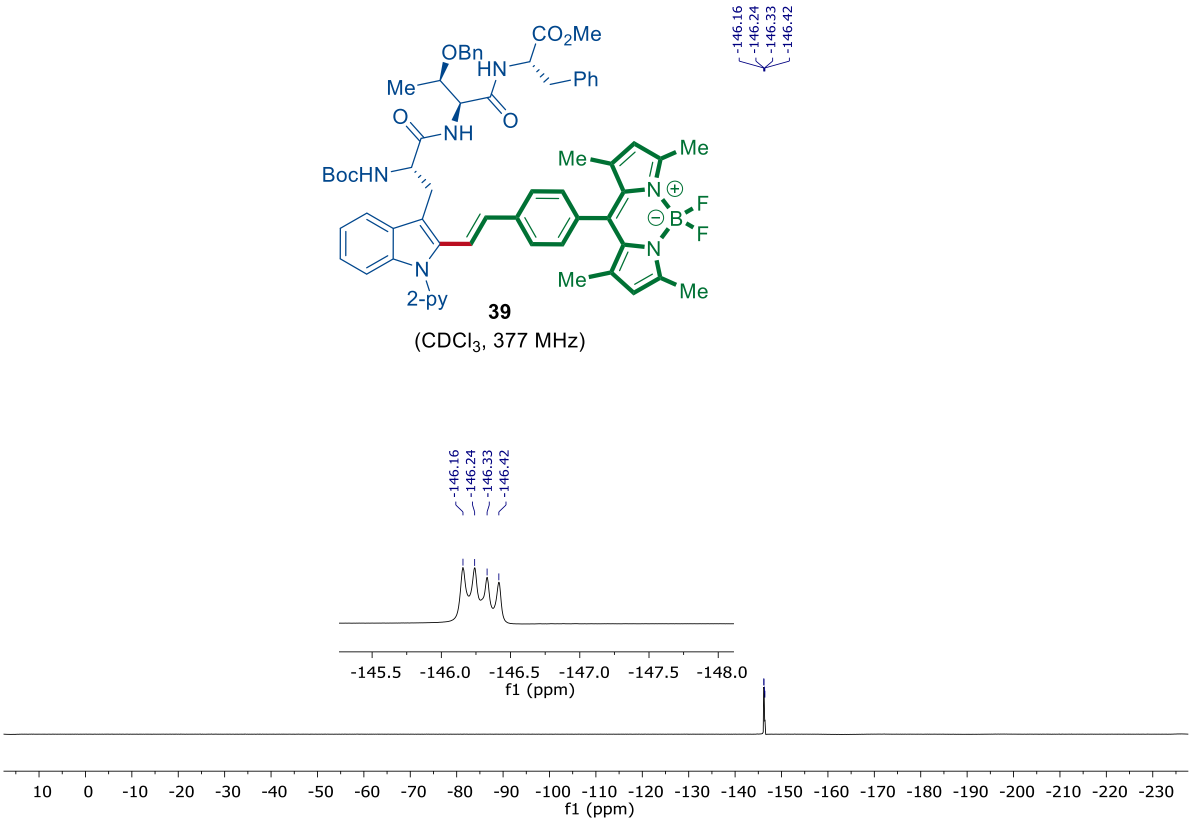

Supplementary Figure 140. ^{19}F-NMR spectrum of **39**.

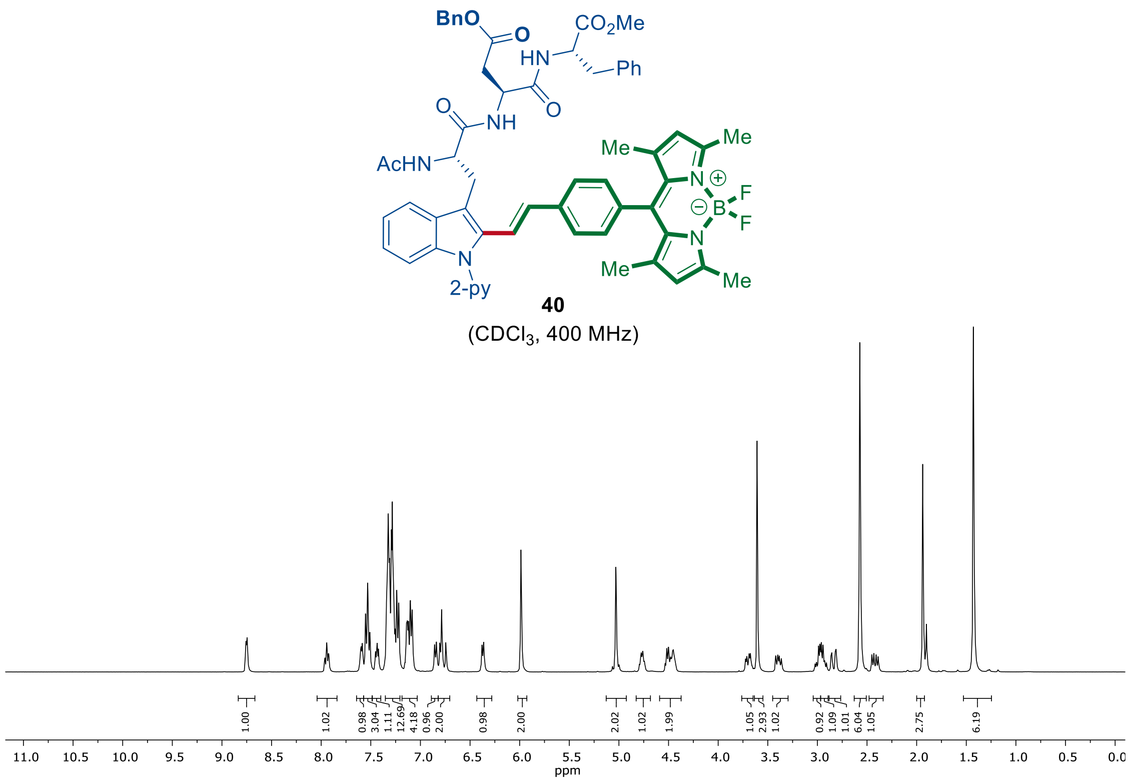

Supplementary Figure 141. ^{1}H-NMR spectrum of **40**.

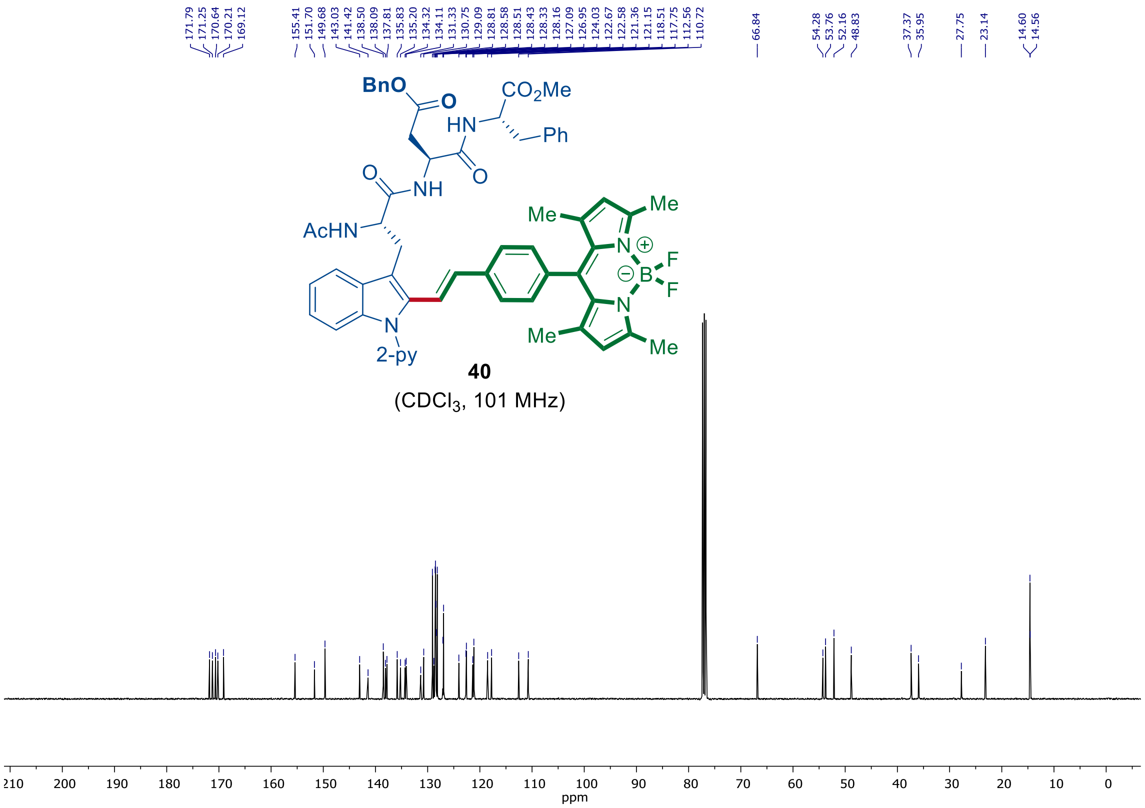

Supplementary Figure 142. ^{13}C-NMR spectrum of **40**.

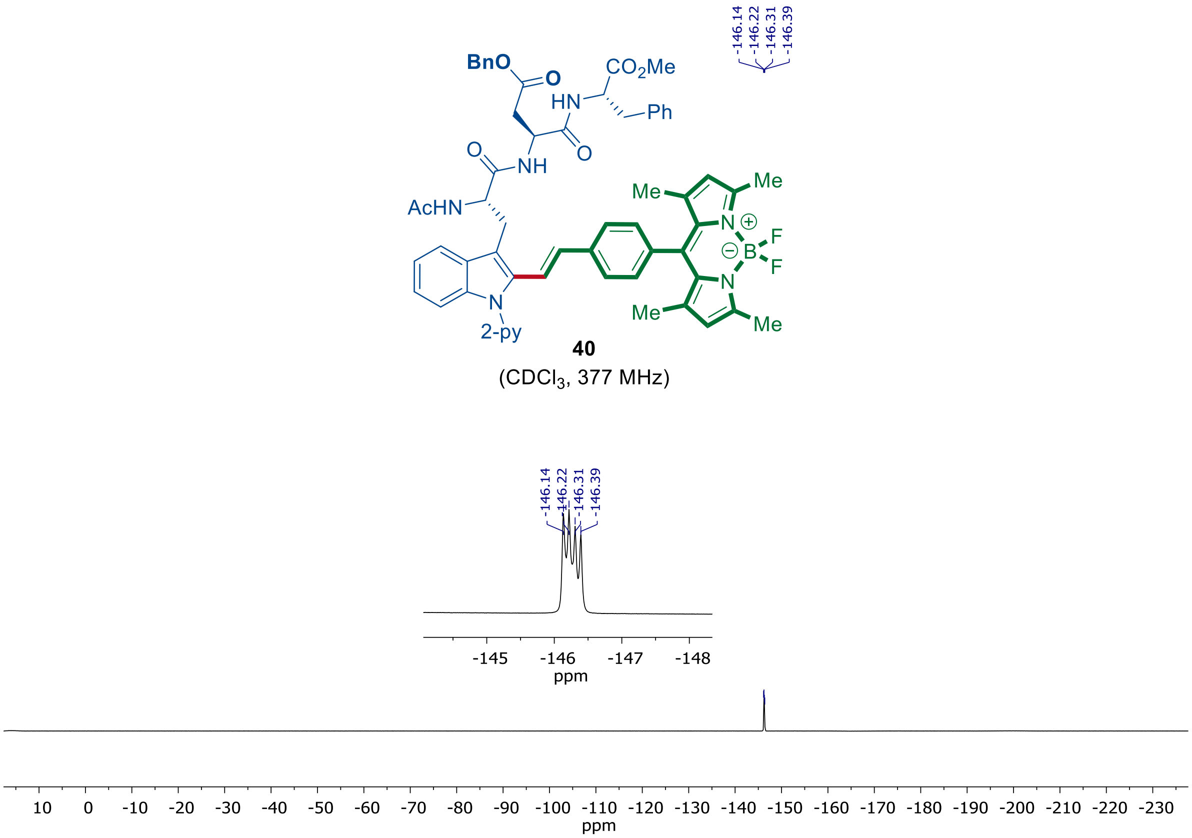

Supplementary Figure 143. ^{19}F-NMR spectrum of **40**.

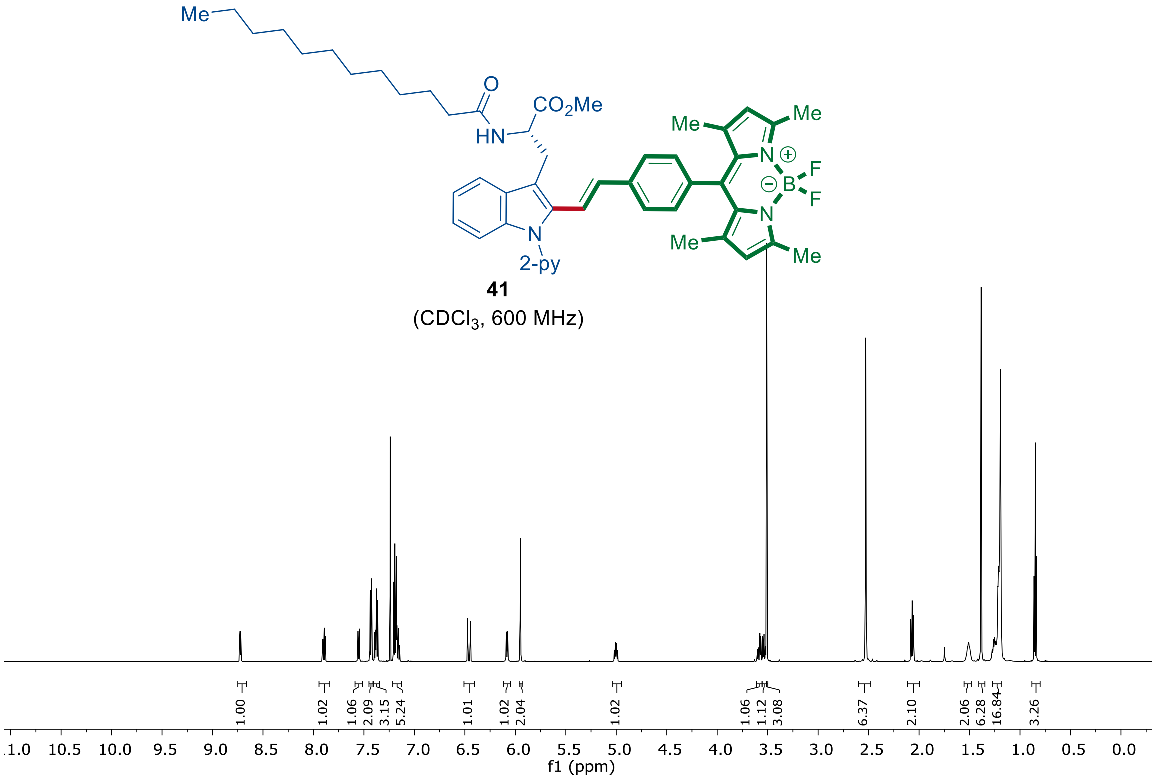

Supplementary Figure 144. ^{1}H-NMR spectrum of **41**.

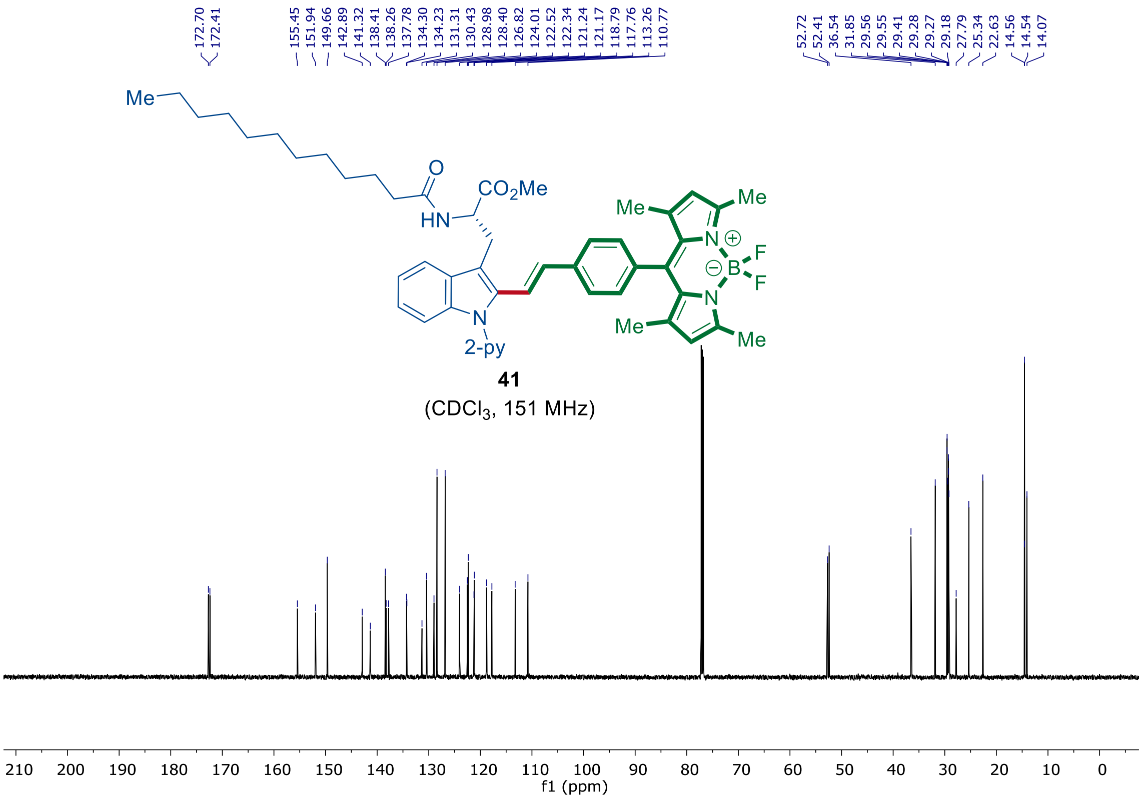

Supplementary Figure 145. ^{13}C-NMR spectrum of **41**.

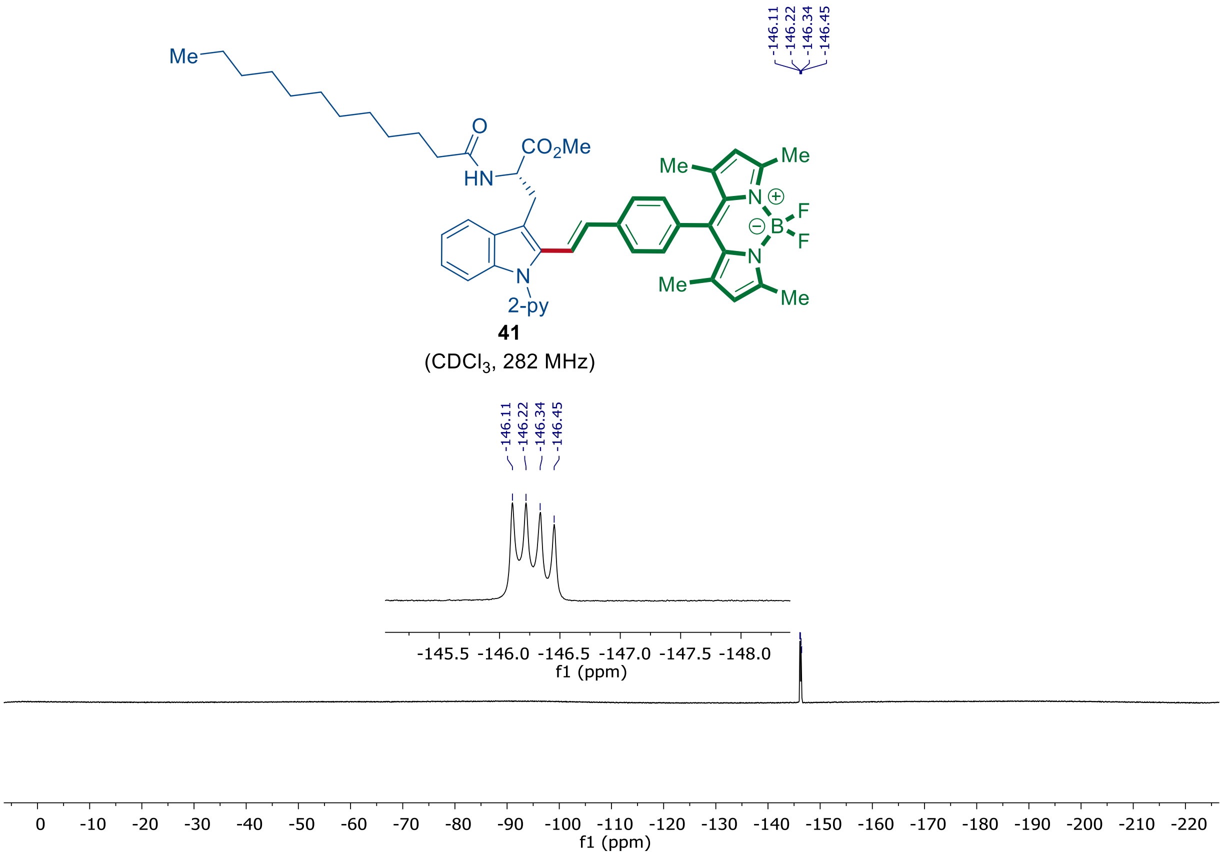

Supplementary Figure 146. ^{19}F-NMR spectrum of **41**.

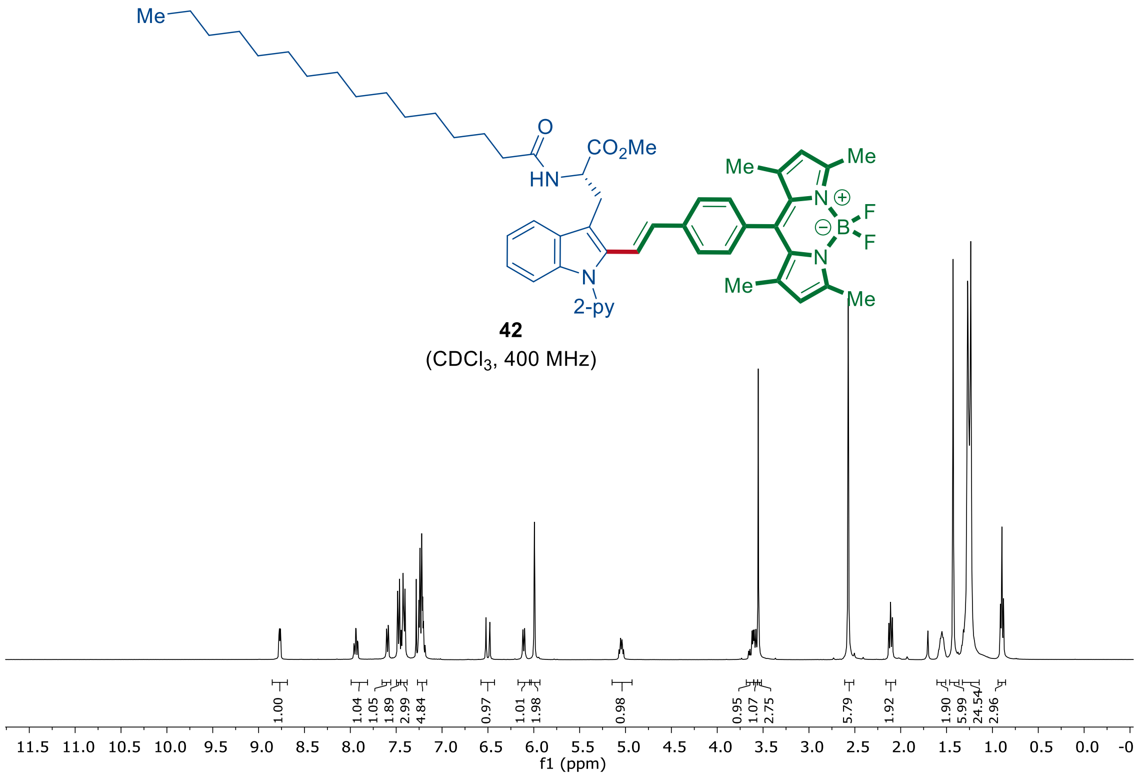

Supplementary Figure 147. ^{1}H-NMR spectrum of **42**.

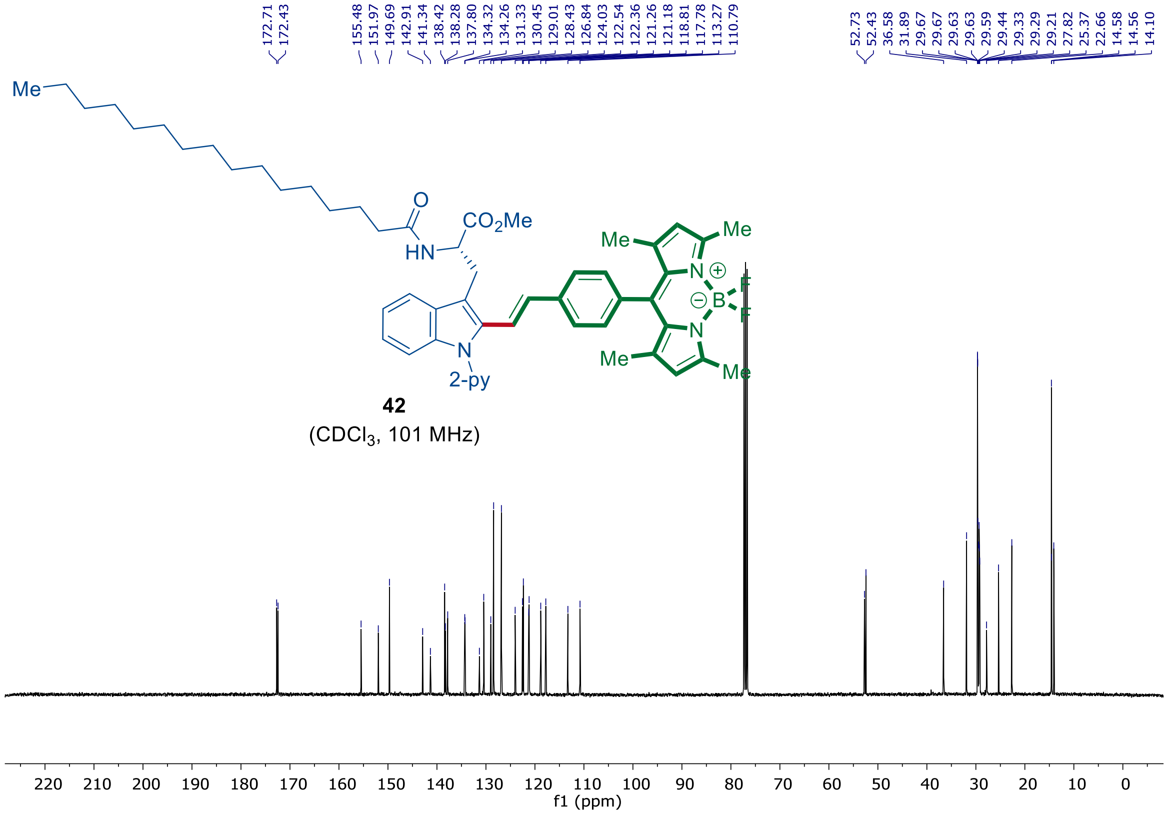

Supplementary Figure 148. ^{13}C-NMR spectrum of **42**.

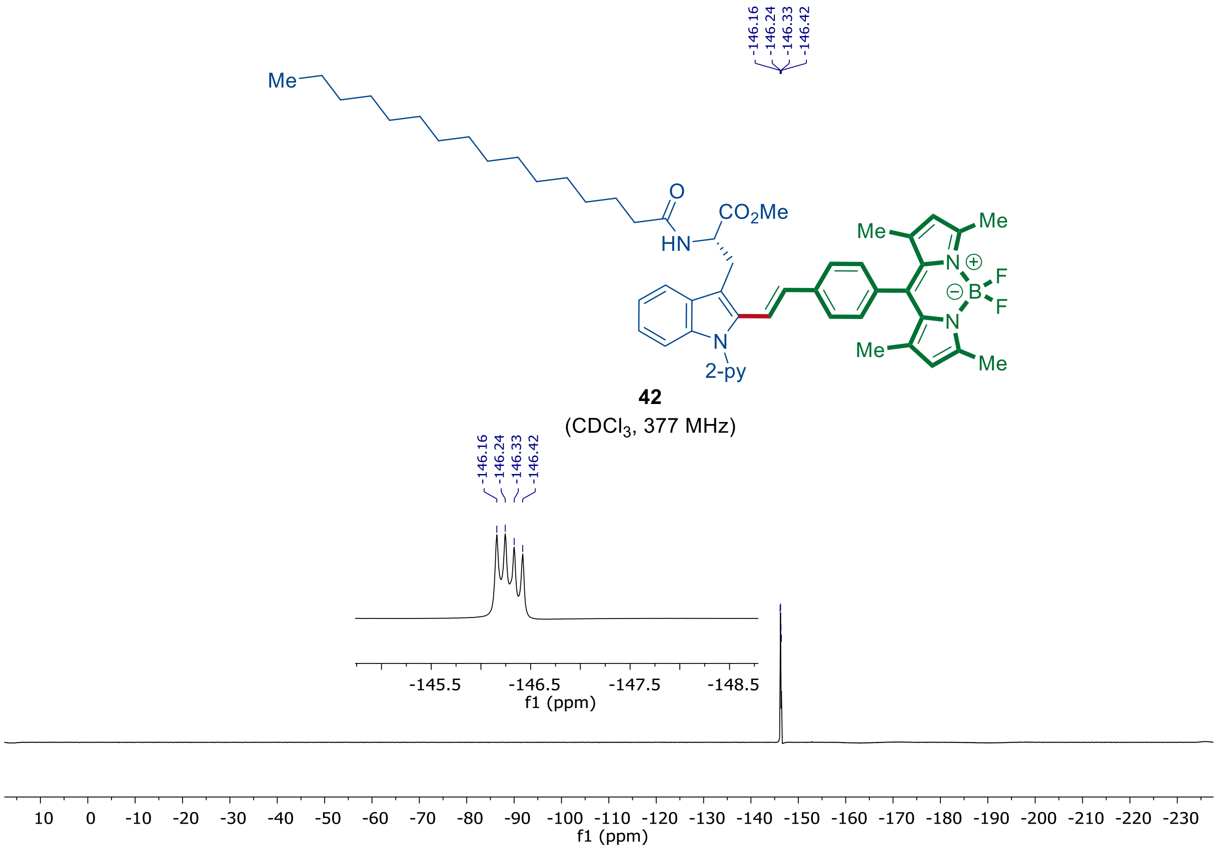

Supplementary Figure 149. ^{19}F-NMR spectrum of **42**.

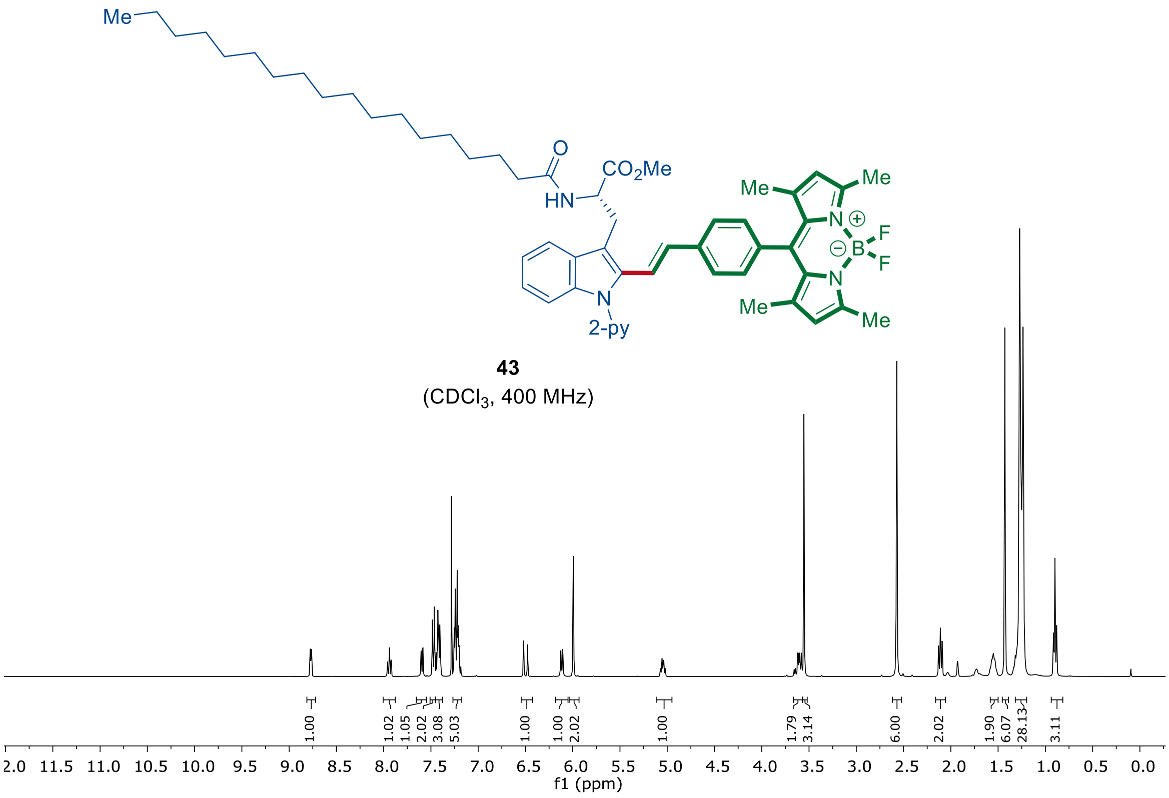

Supplementary Figure 150. ^{1}H-NMR spectrum of **43**.

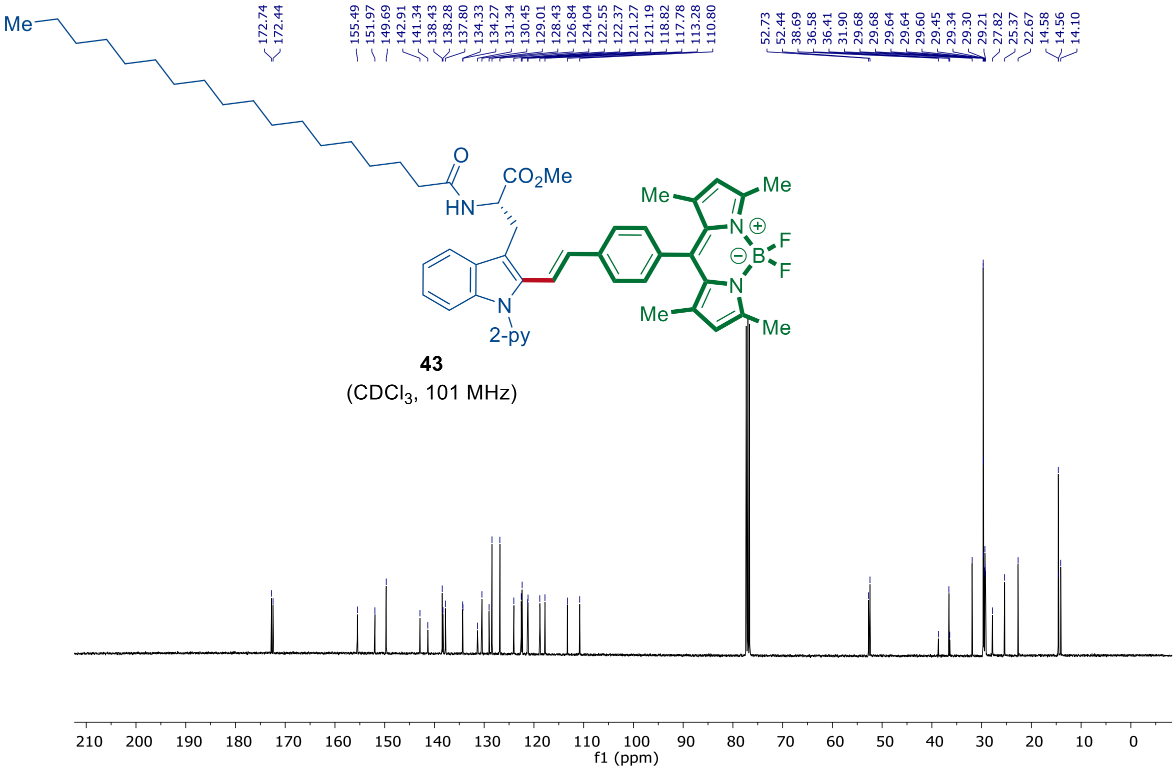

Supplementary Figure 151. ^{13}C-NMR spectrum of **43**.

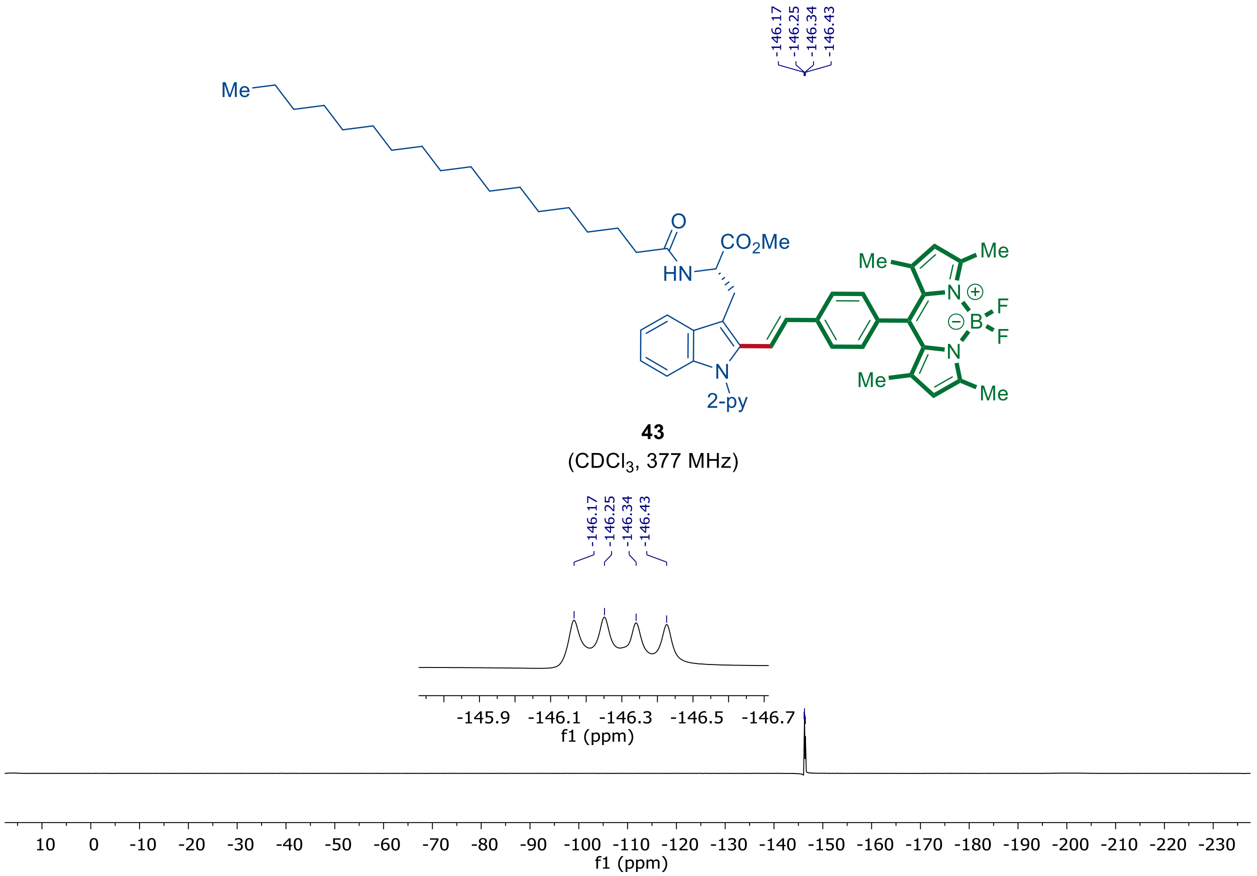

Supplementary Figure 152. ^{19}F-NMR spectrum of **43**.

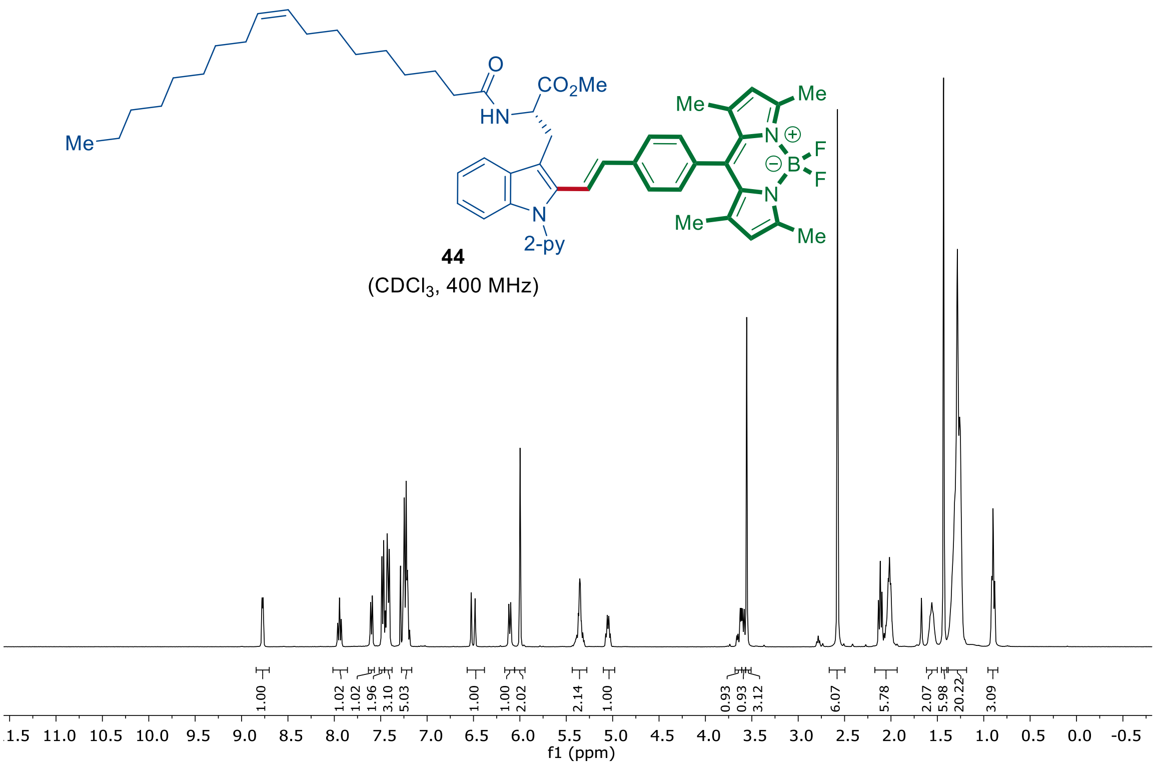

Supplementary Figure 153. ^{1}H-NMR spectrum of **44**.

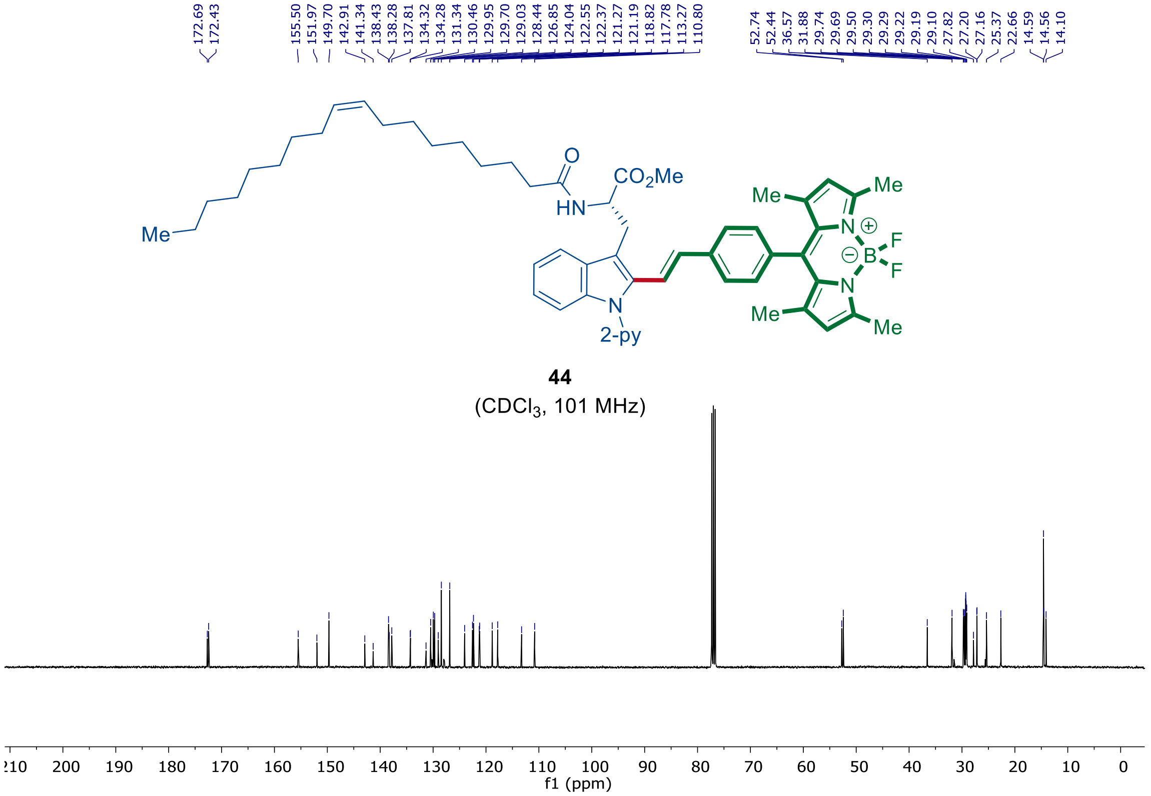

Supplementary Figure 154. ^{13}C-NMR spectrum of **44**.

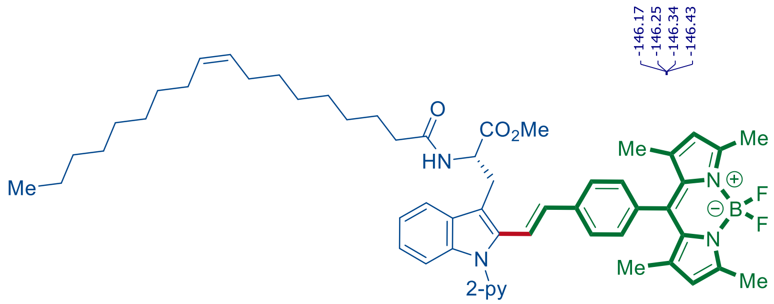

44

($CDCl_3$, 377 MHz)

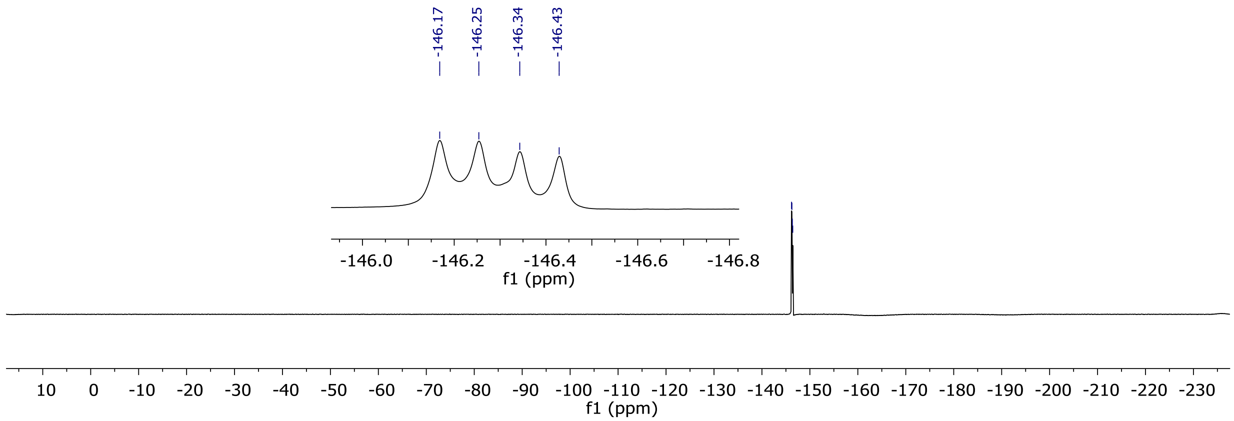

Supplementary Figure 155. ^{19}F-NMR spectrum of **44**.

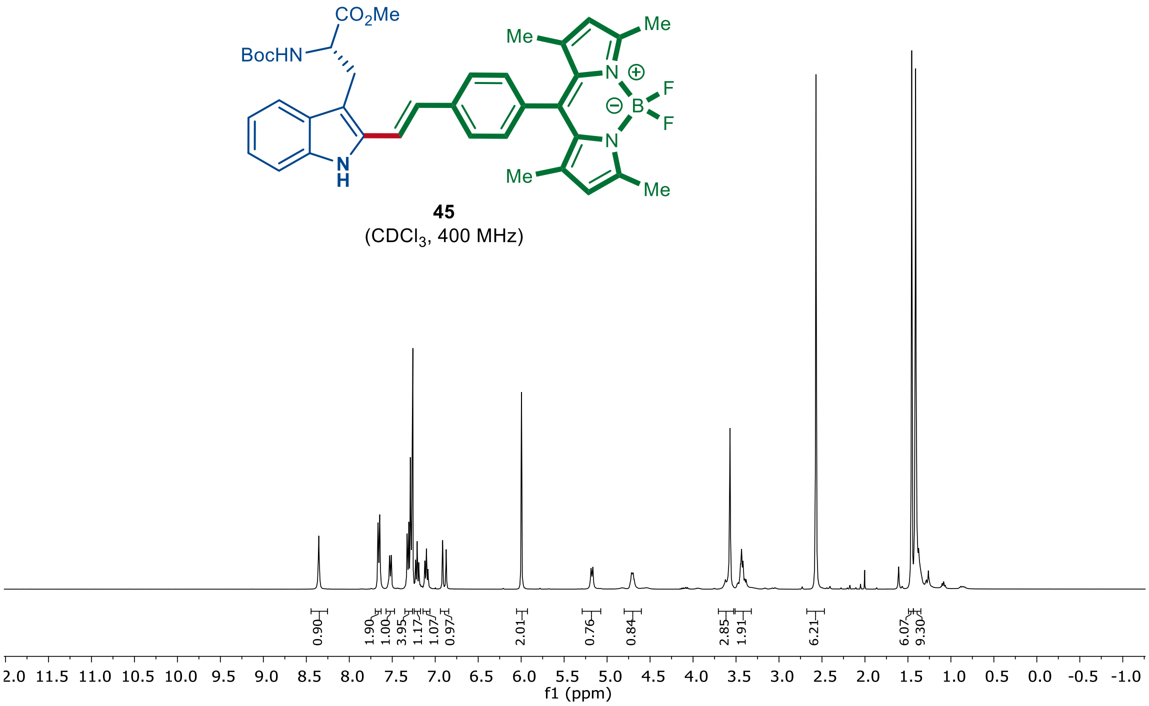

Supplementary Figure 156. ^{1}H-NMR spectrum of **45**.

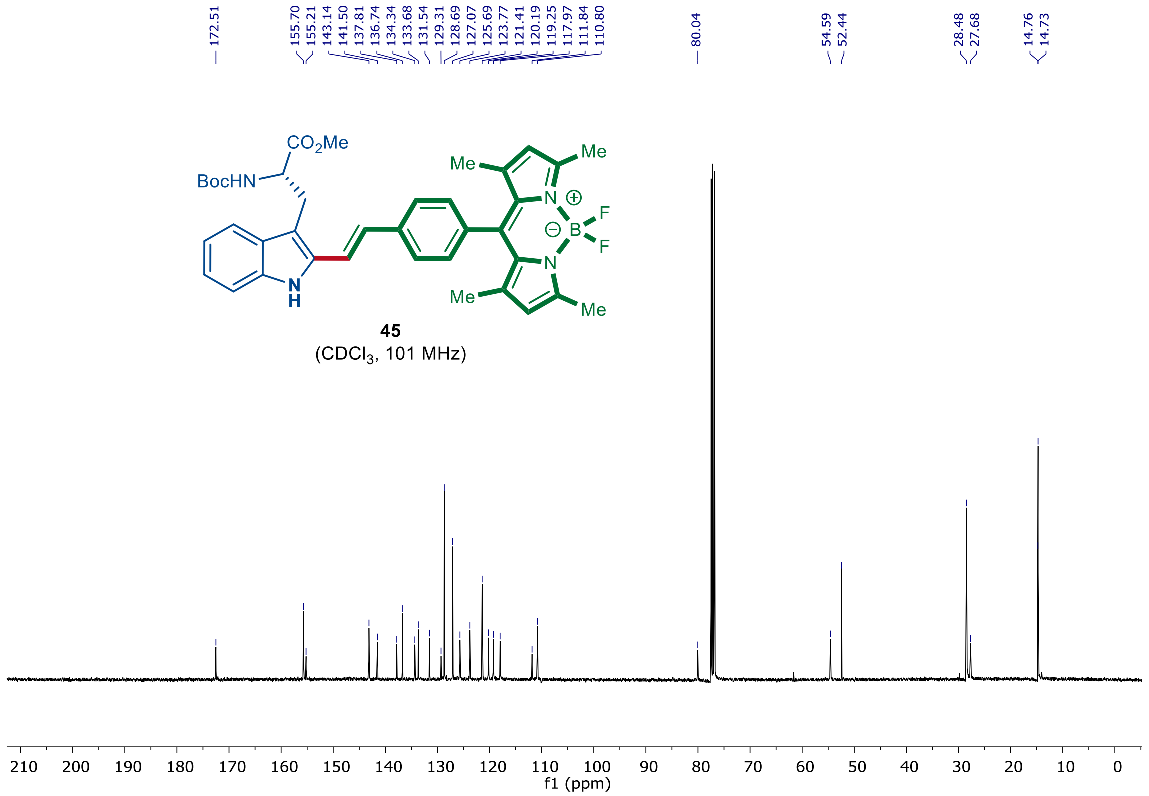

Supplementary Figure 157. ^{13}C-NMR spectrum of **45**.

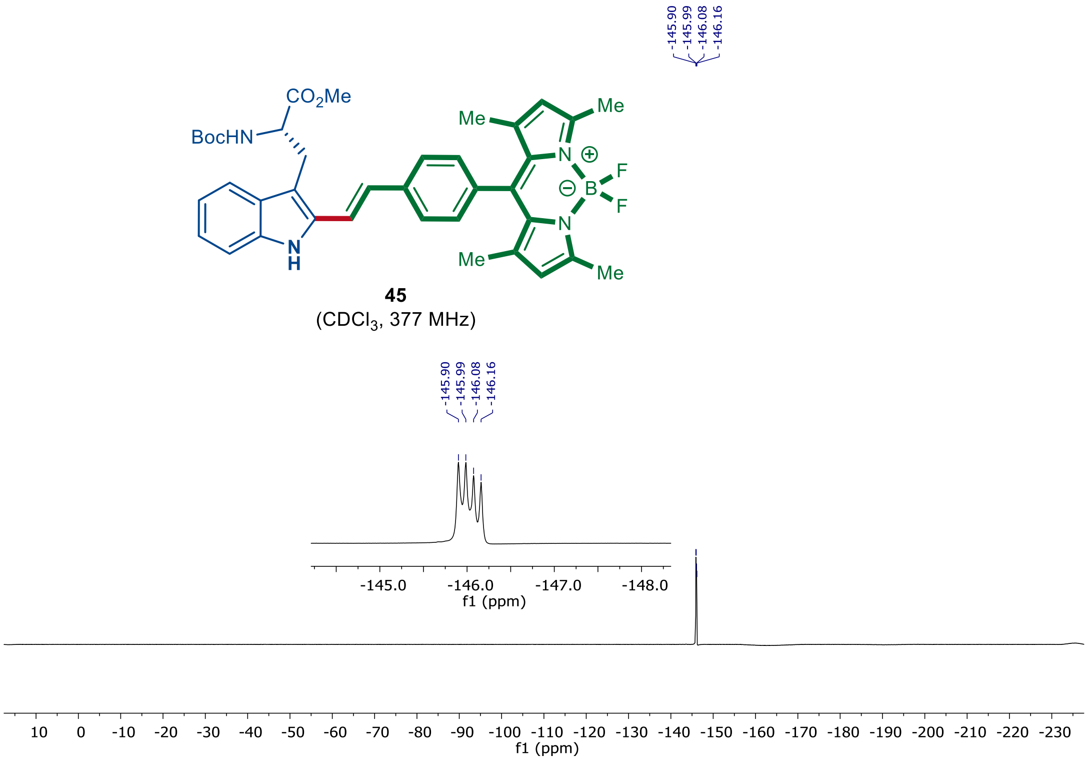

Supplementary Figure 158. ^{19}F-NMR spectrum of **45**.

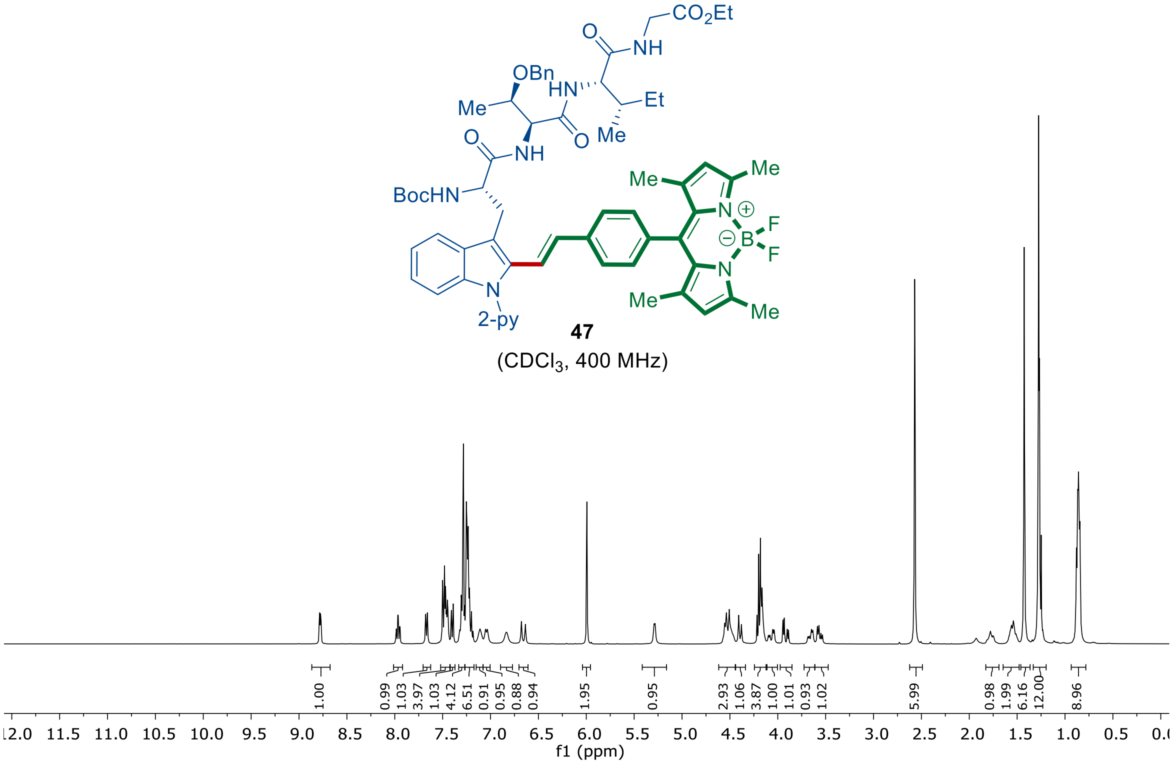

Supplementary Figure 159. ^{1}H-NMR spectrum of **47**.

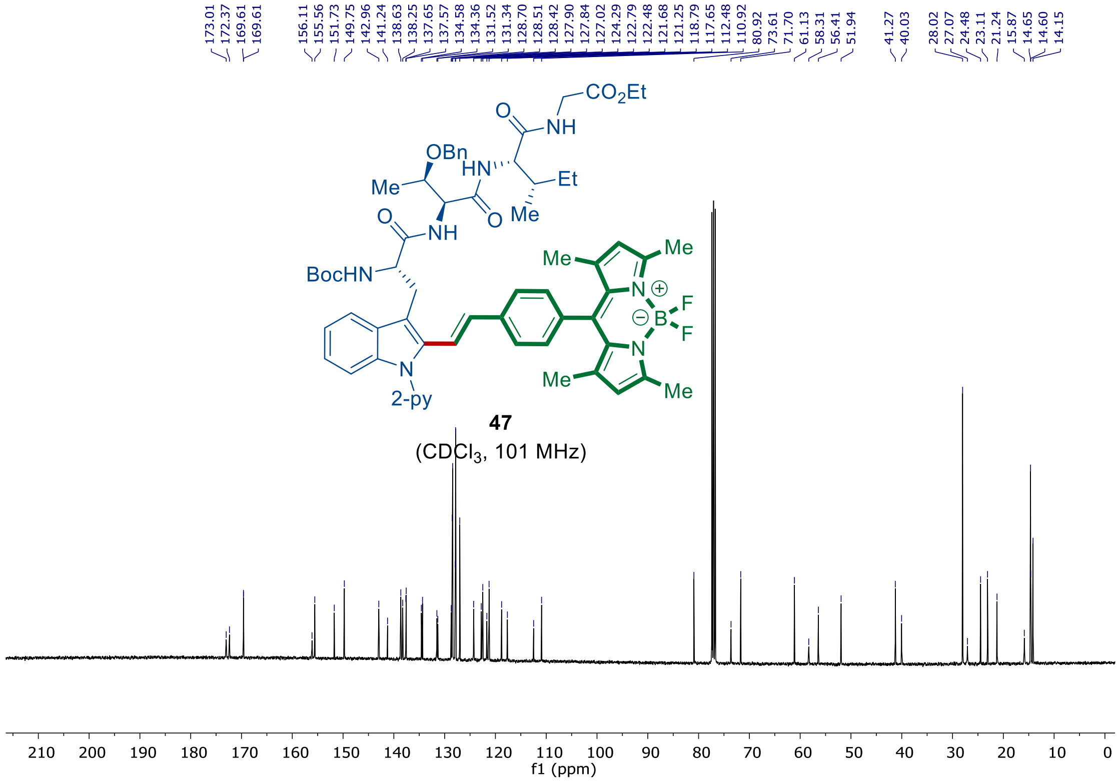

Supplementary Figure 160. ^{13}C-NMR spectrum of **47**.

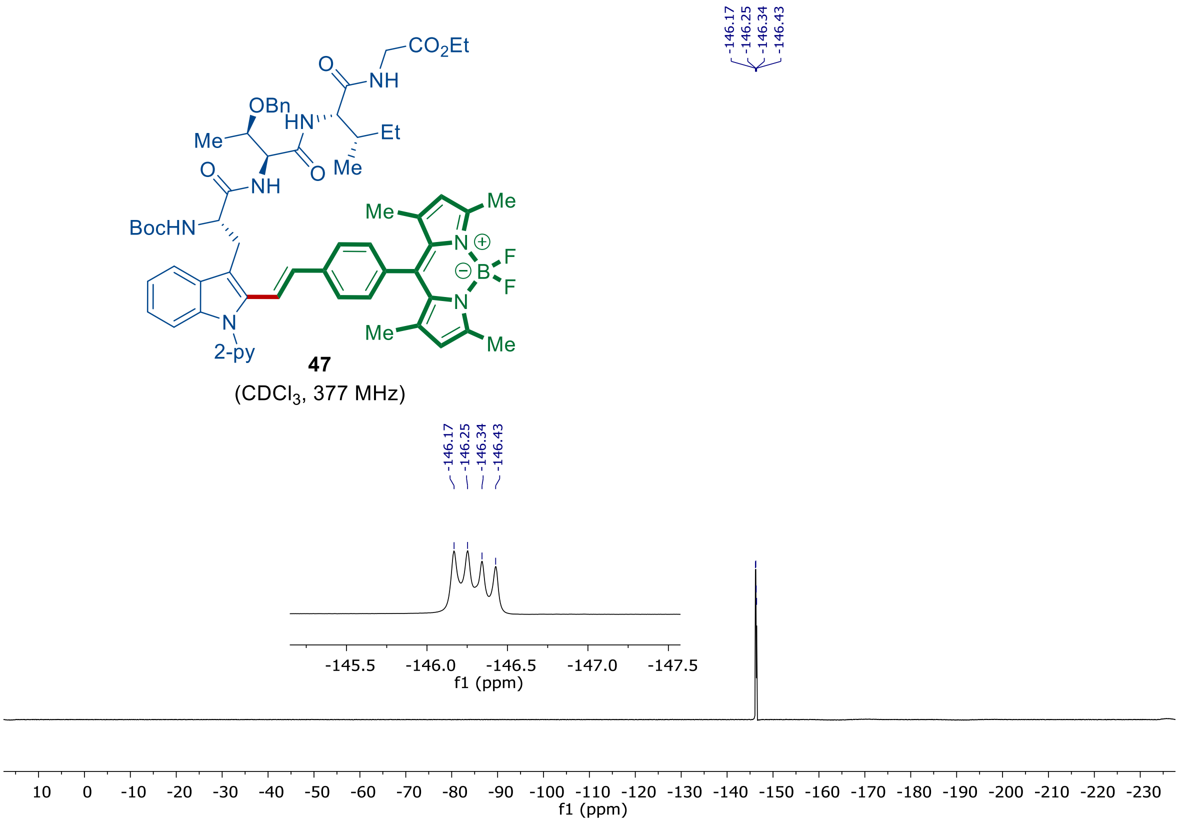

Supplementary Figure 161. ^{19}F-NMR spectrum of **47**.

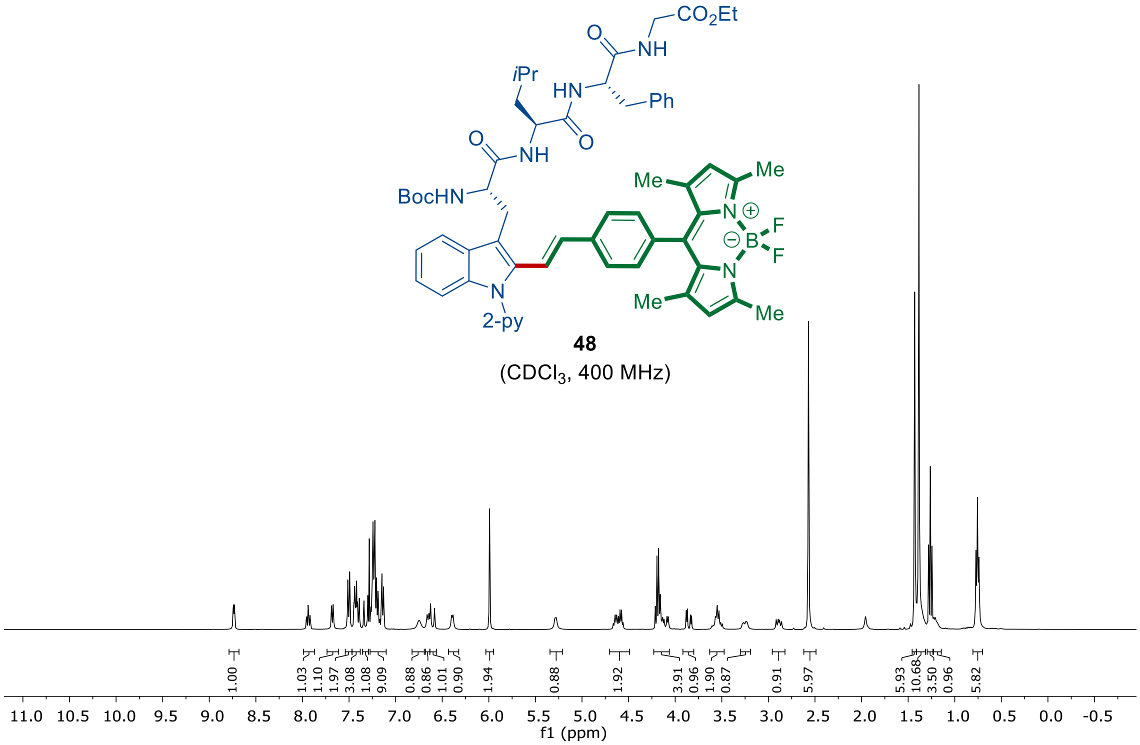

Supplementary Figure 162. ^{1}H-NMR spectrum of **48**.

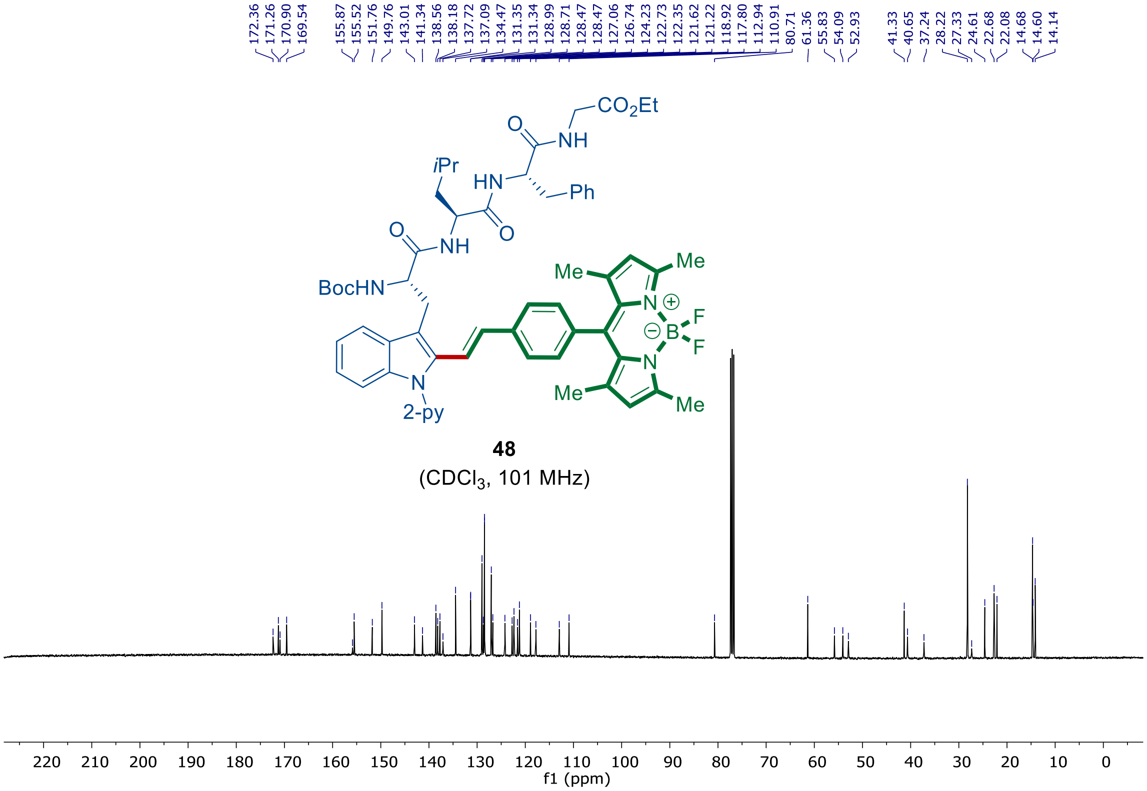

Supplementary Figure 163. ^{13}C-NMR spectrum of **48**.

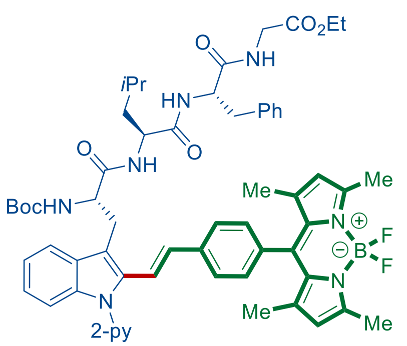

48

($CDCl_3$, 377 MHz)

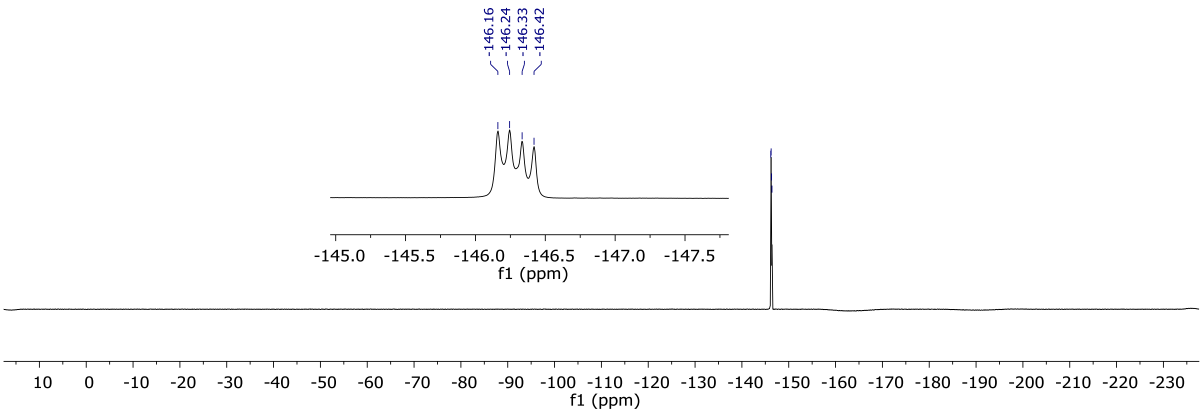

Supplementary Figure 164. ^{19}F-NMR spectrum of **48**.

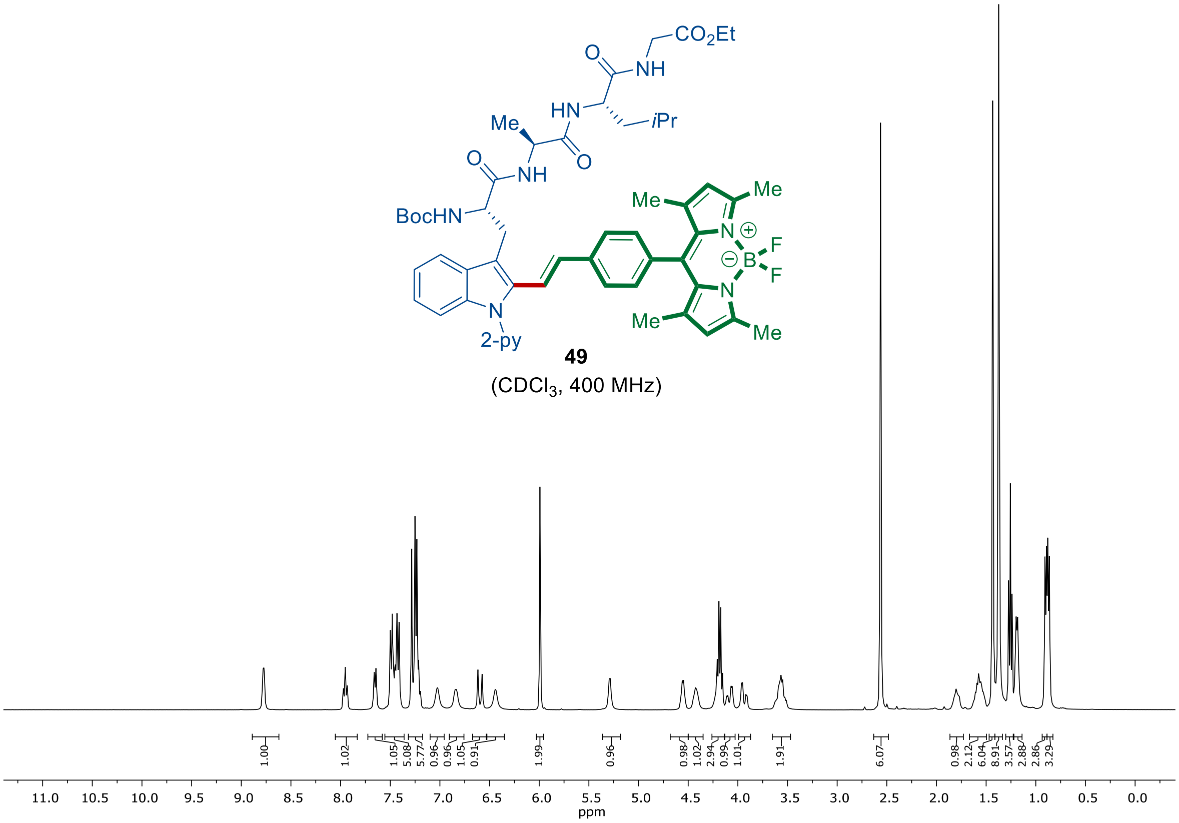

Supplementary Figure 165. ^{1}H-NMR spectrum of **49**.

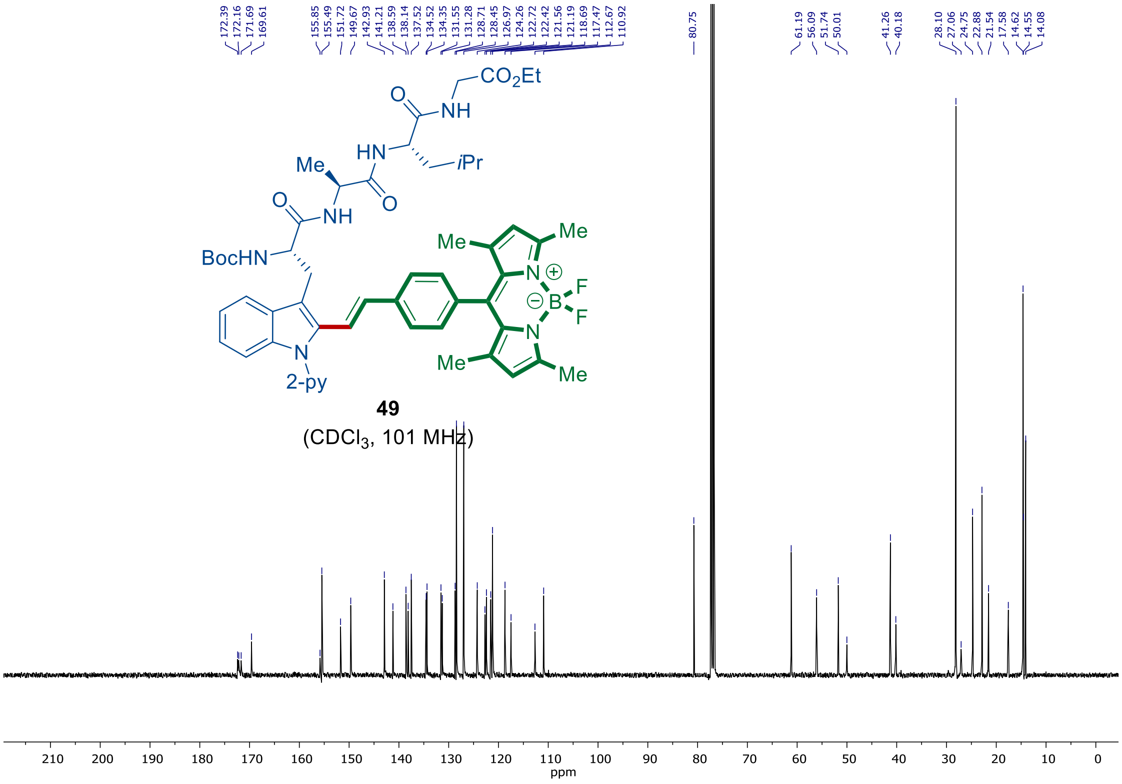

Supplementary Figure 166. ^{13}C-NMR spectrum of **49**.

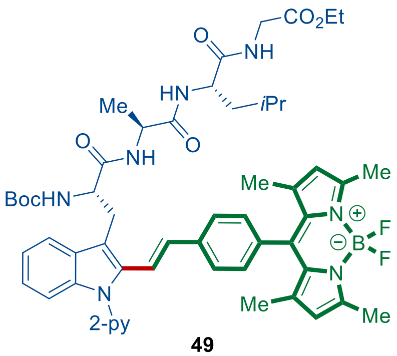

49

($CDCl_3$, 377 MHz)

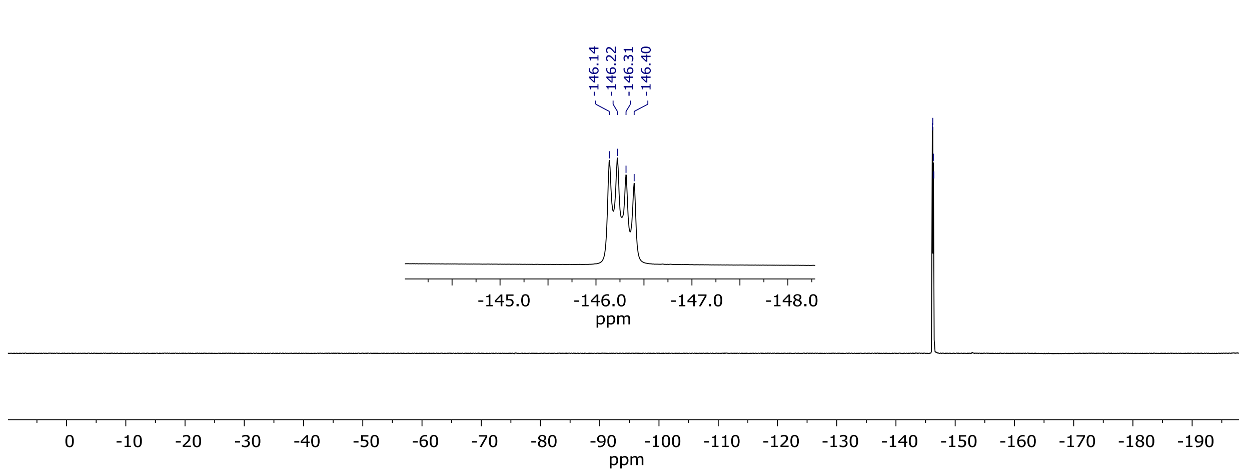

Supplementary Figure 167. ^{19}F-NMR spectrum of **49**.

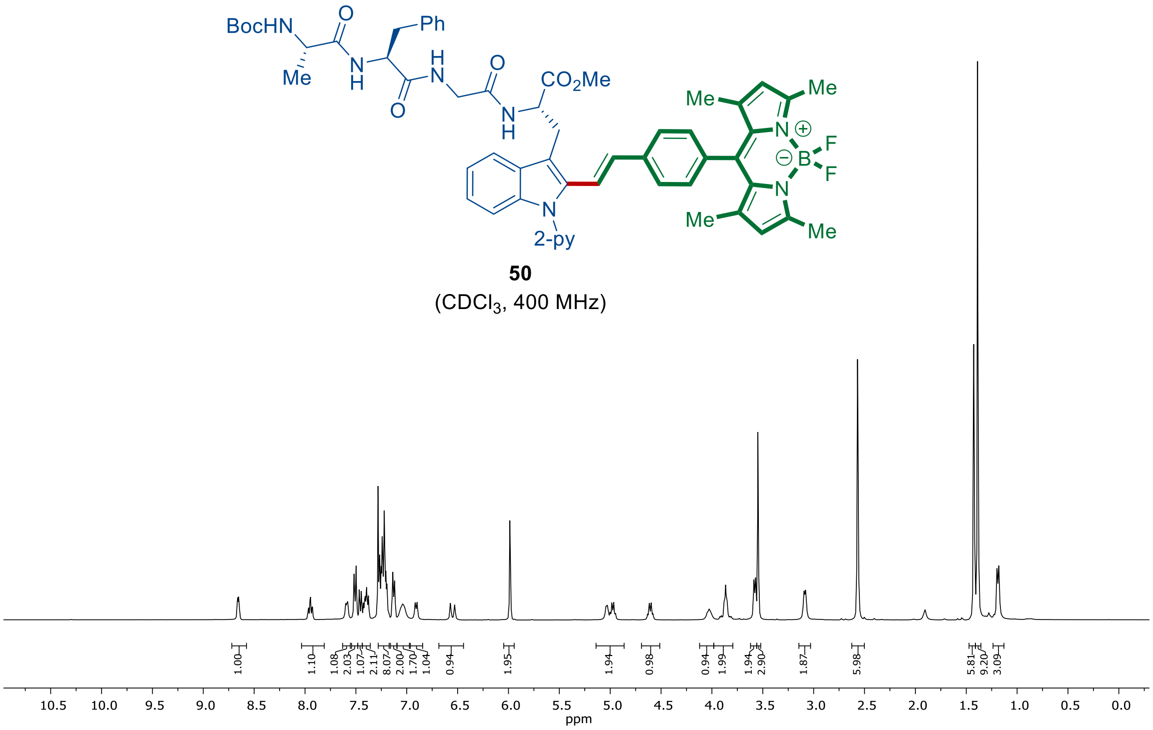

Supplementary Figure 168. ^{1}H-NMR spectrum of **50**.

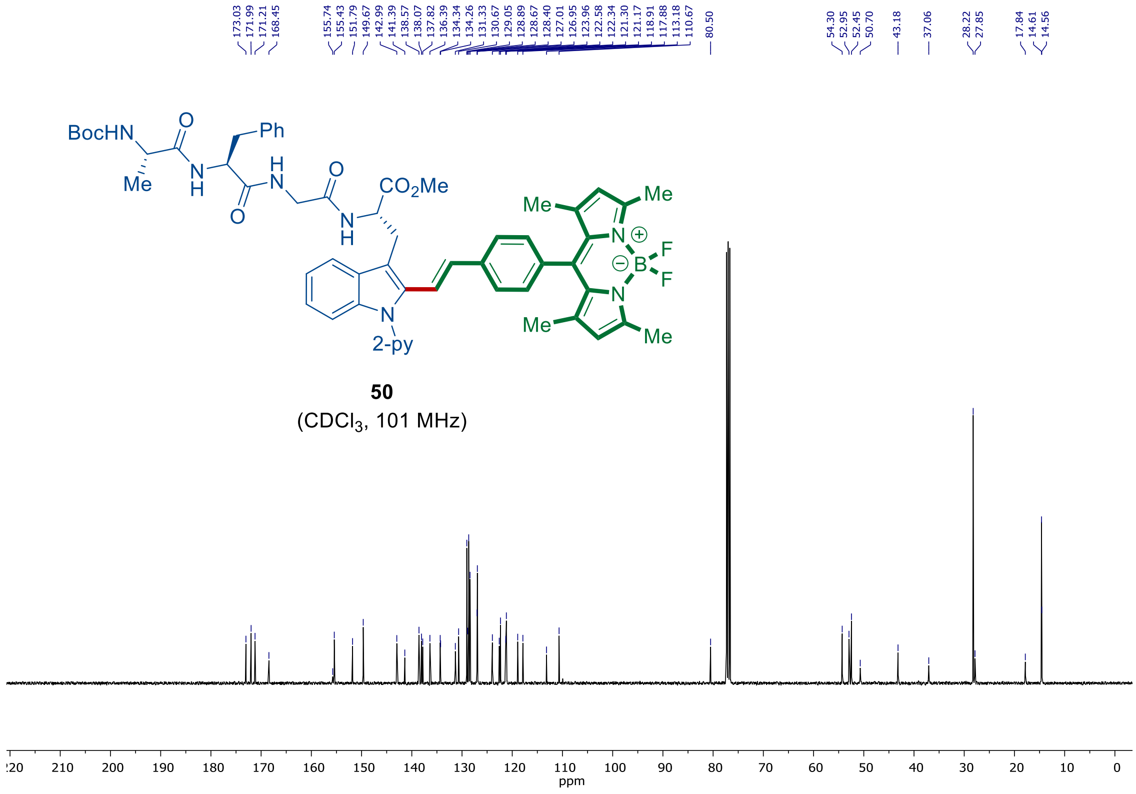

Supplementary Figure 169. ^{13}C-NMR spectrum of **50**.

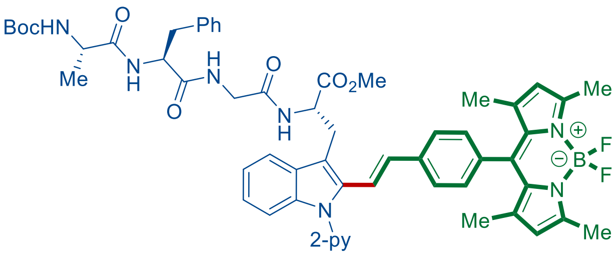

50

($CDCl_3$, 377 MHz)

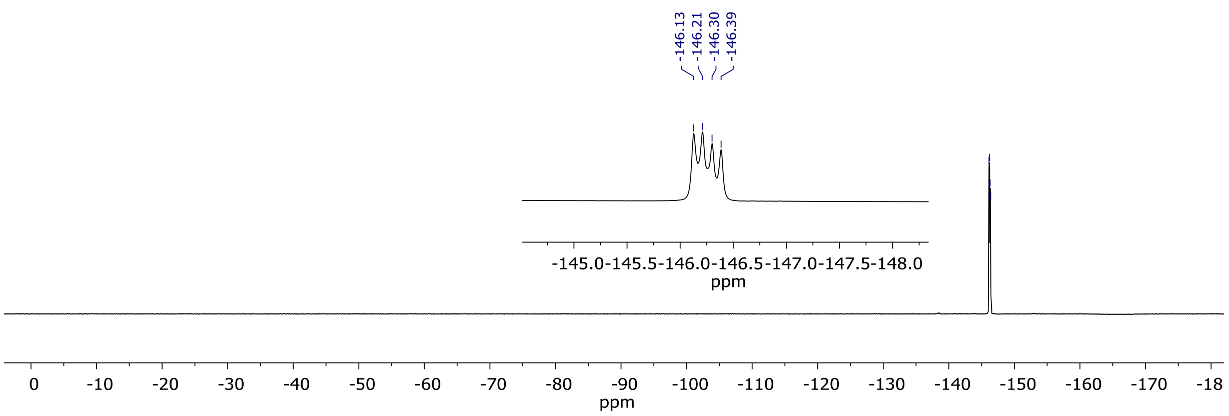

Supplementary Figure 170. ^{19}F-NMR spectrum of **50**.

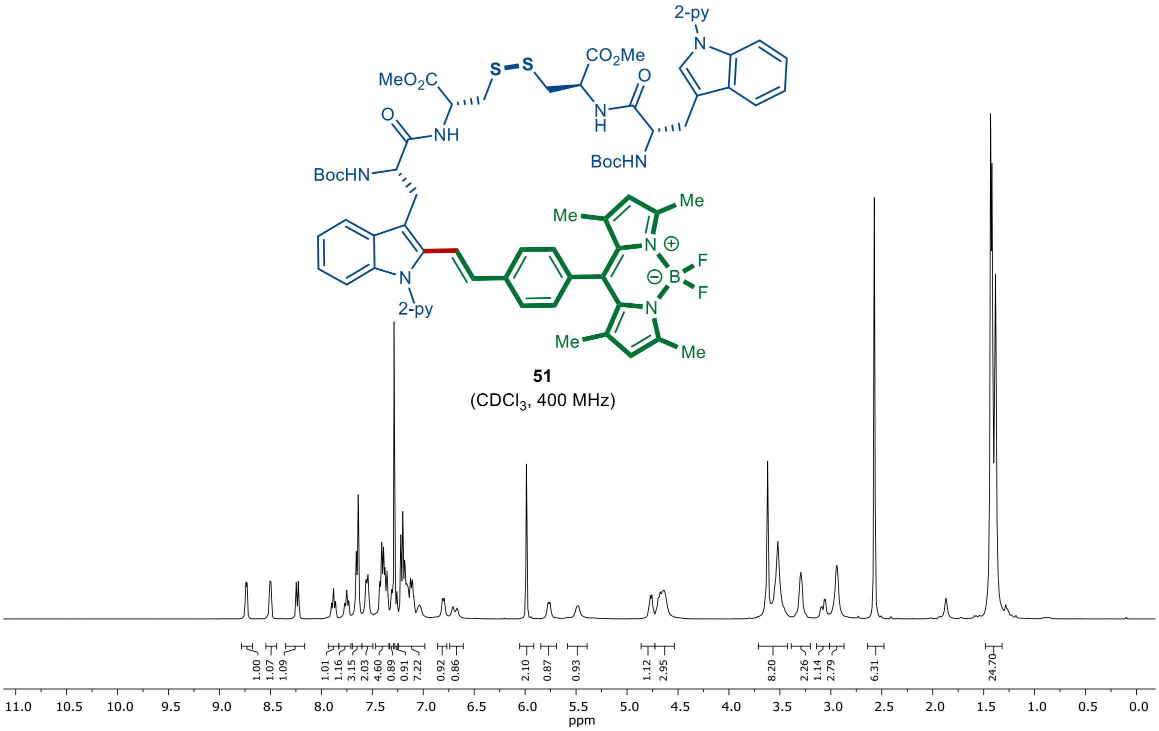

Supplementary Figure 171. ^{1}H-NMR spectrum of **51**.

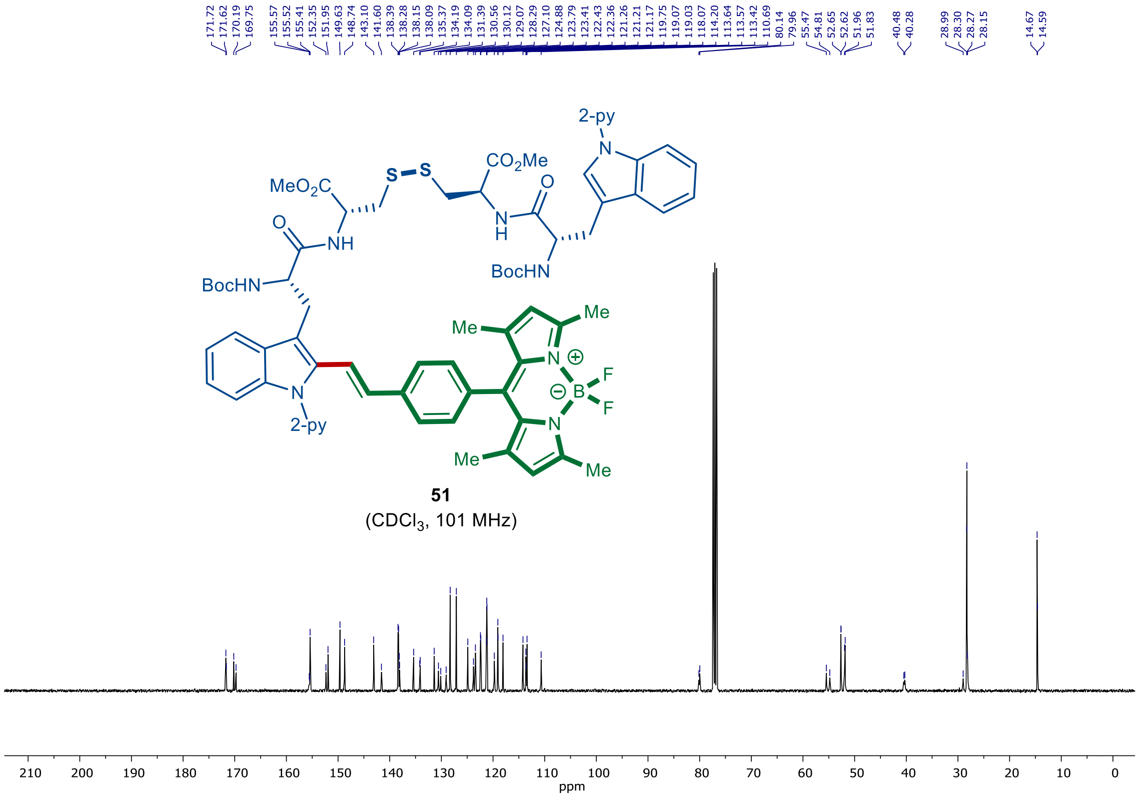

Supplementary Figure 172. ^{13}C-NMR spectrum of **51**.

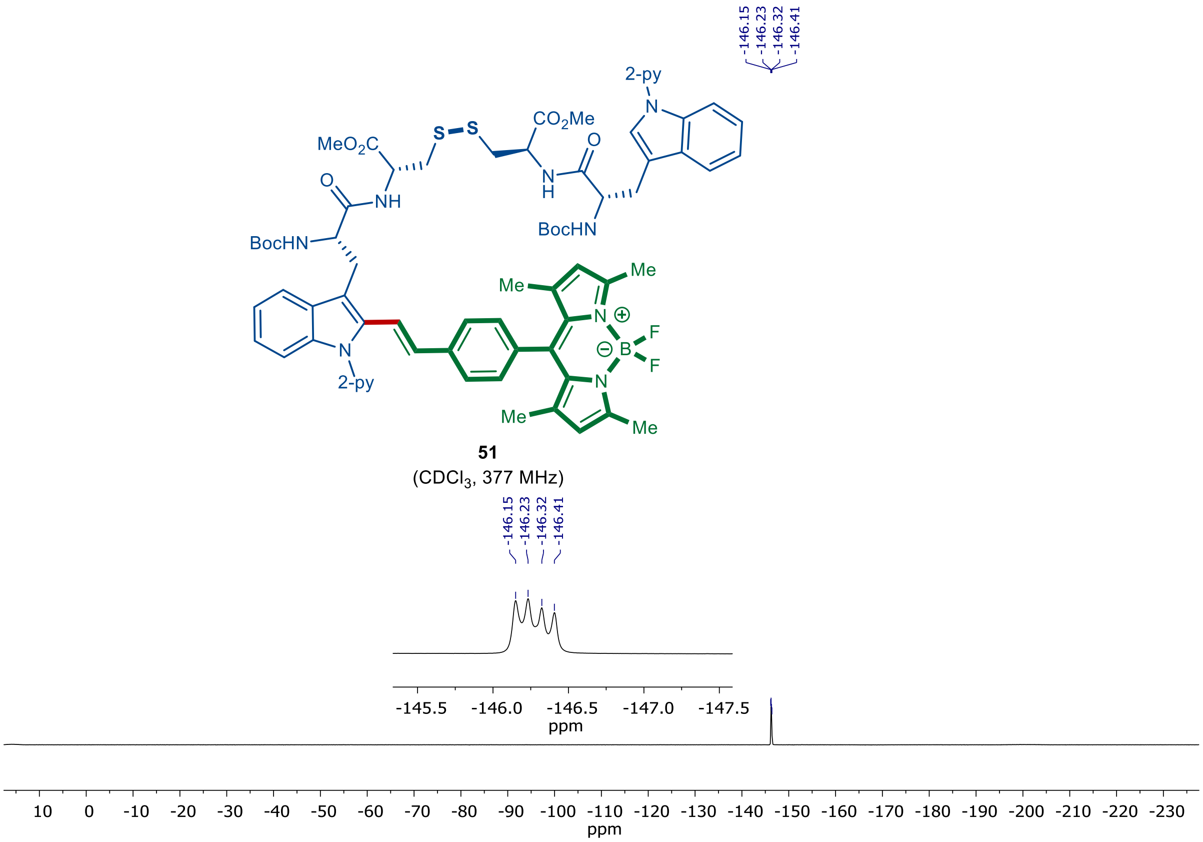

Supplementary Figure 173. ^{19}F-NMR spectrum of **51**.

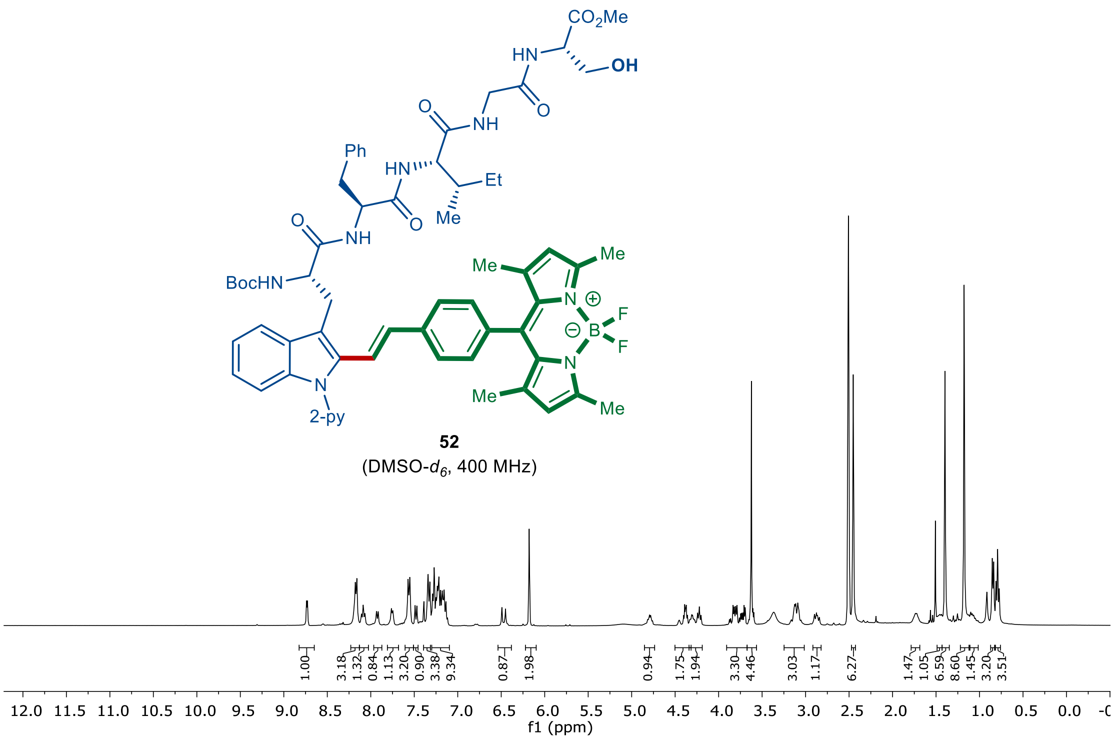

Supplementary Figure 174. ^{1}H-NMR spectrum of **52**.

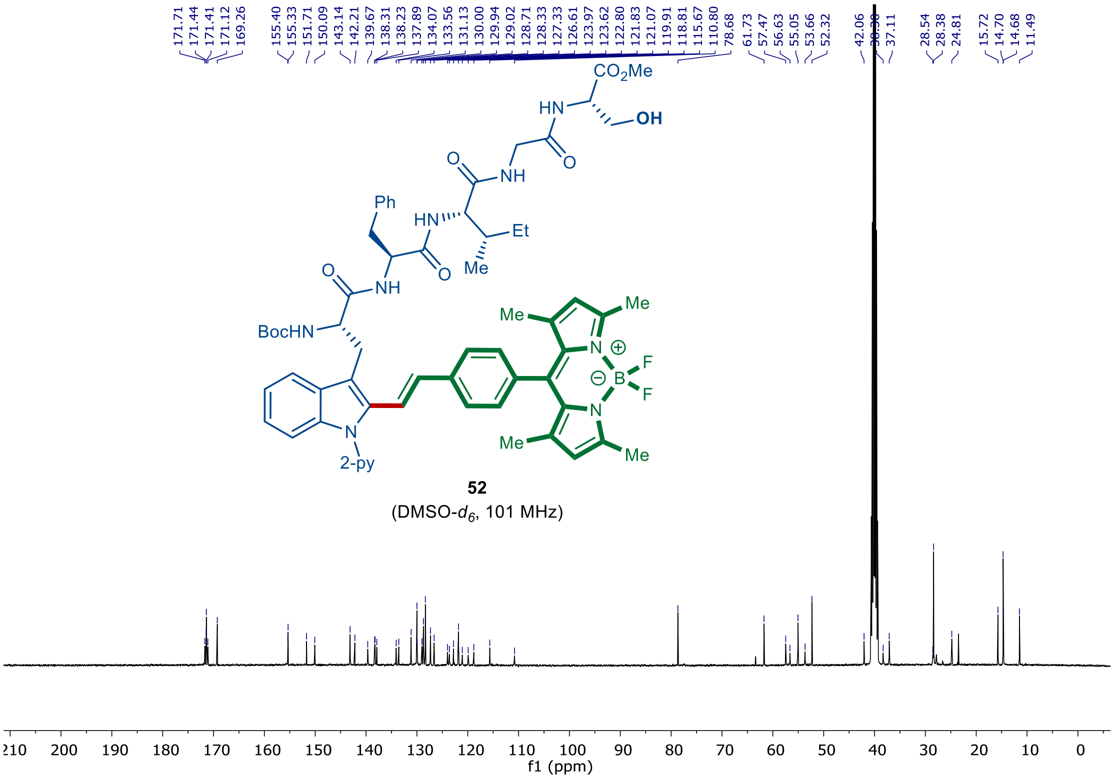

Supplementary Figure 175 ^{13}C-NMR spectrum of **52**.

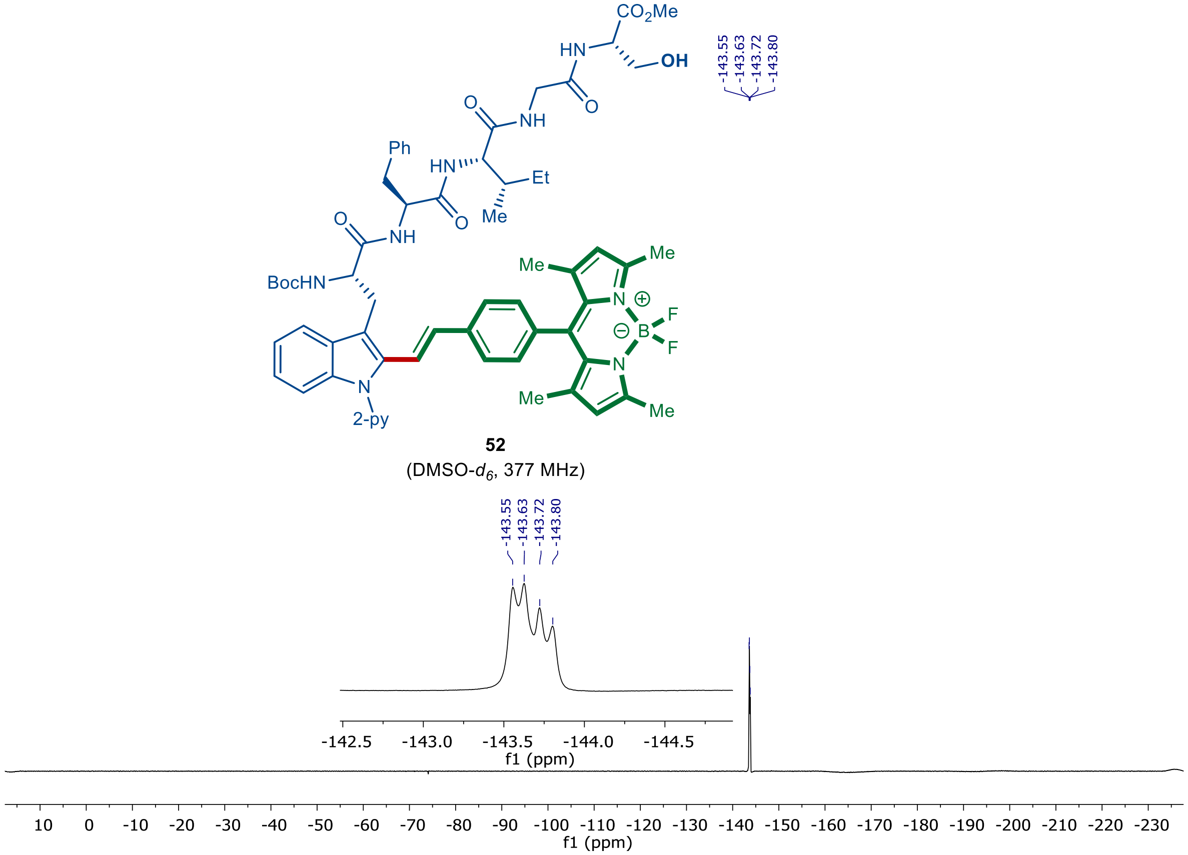

Supplementary Figure 176. ^{19}F-NMR spectrum of **52**.

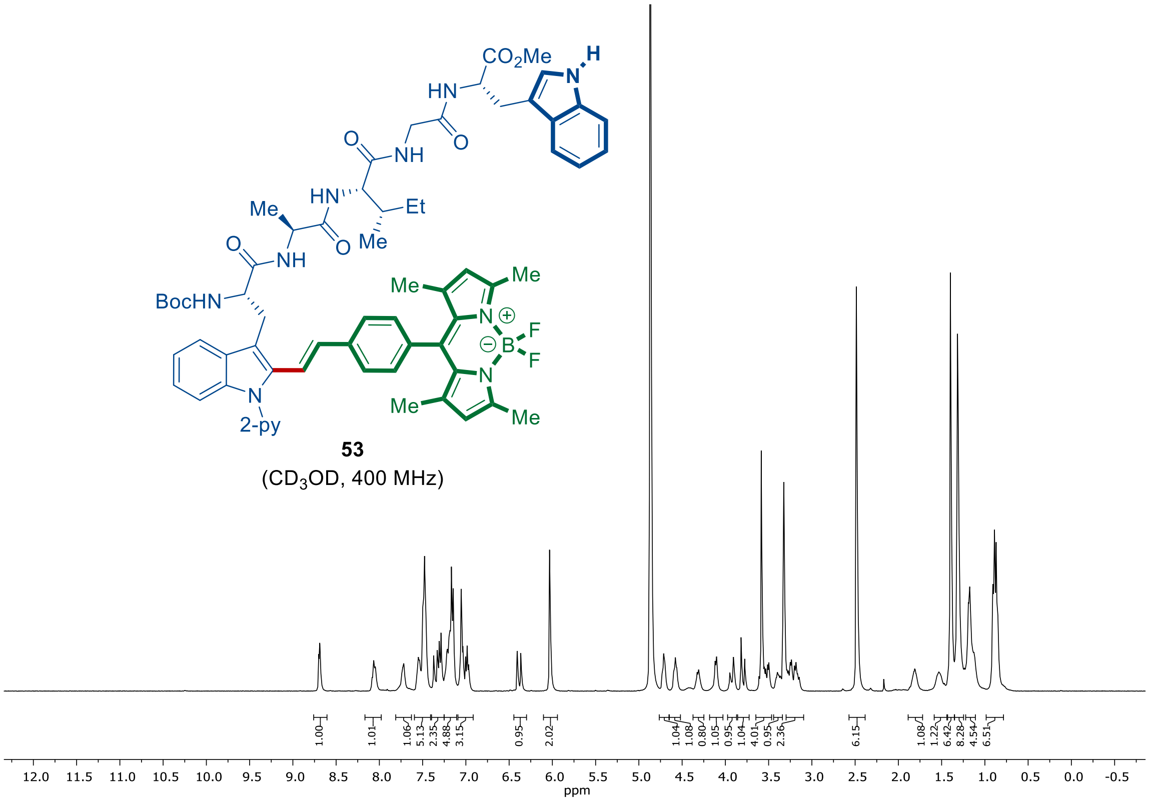

Supplementary Figure 177. ^{1}H-NMR spectrum of **53**.

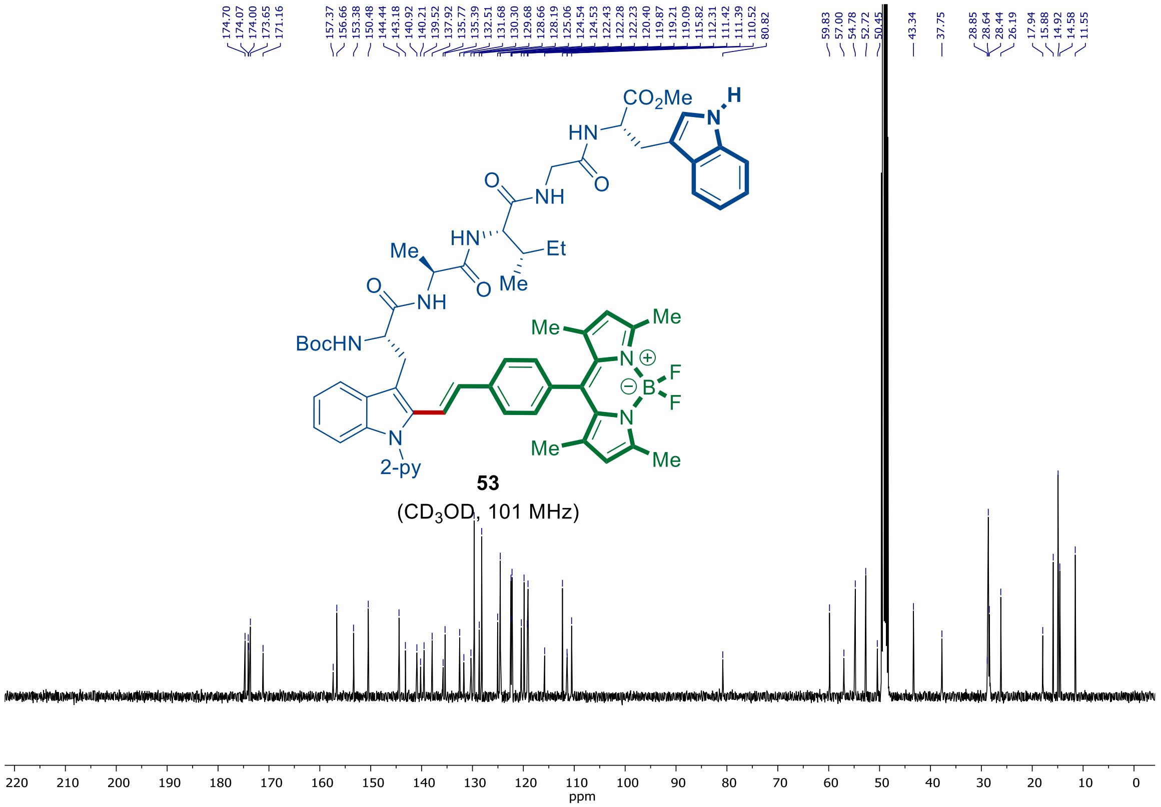

Supplementary Figure 178. ^{13}C-NMR spectrum of **53**.

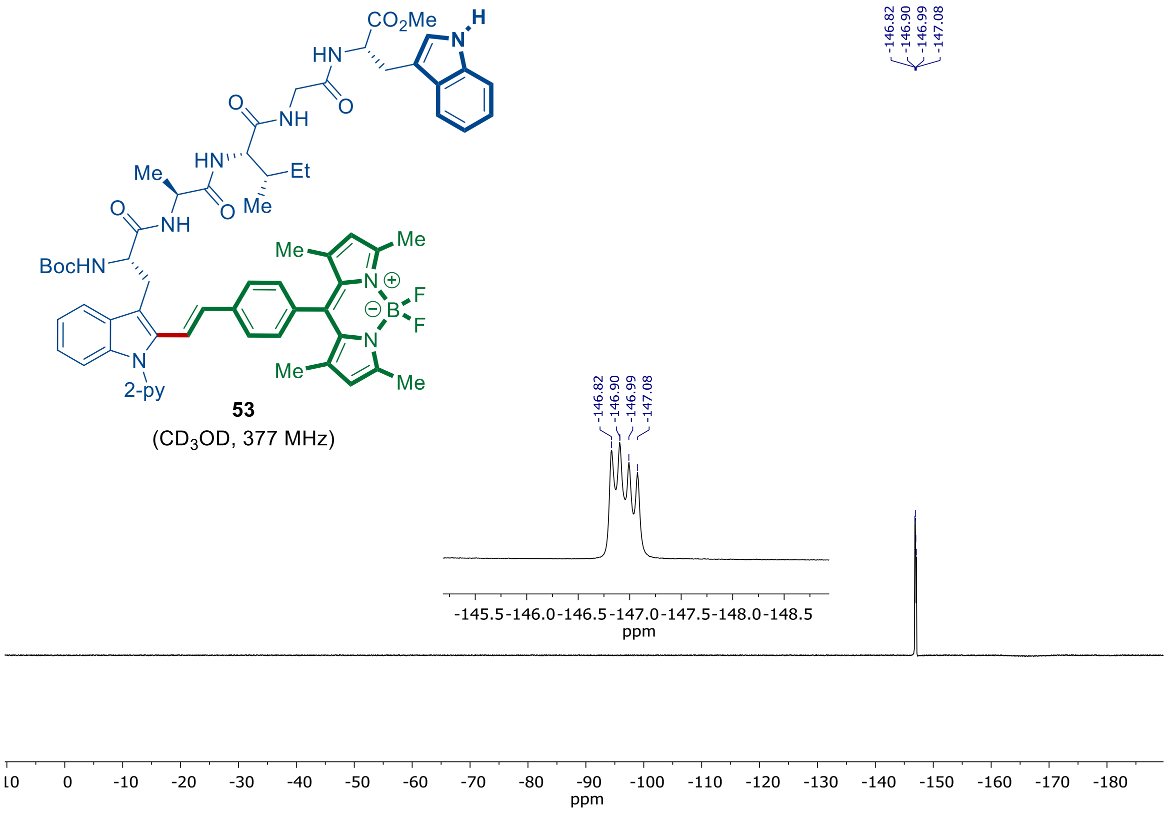

Supplementary Figure 179. ^{19}F-NMR spectrum of **53**.

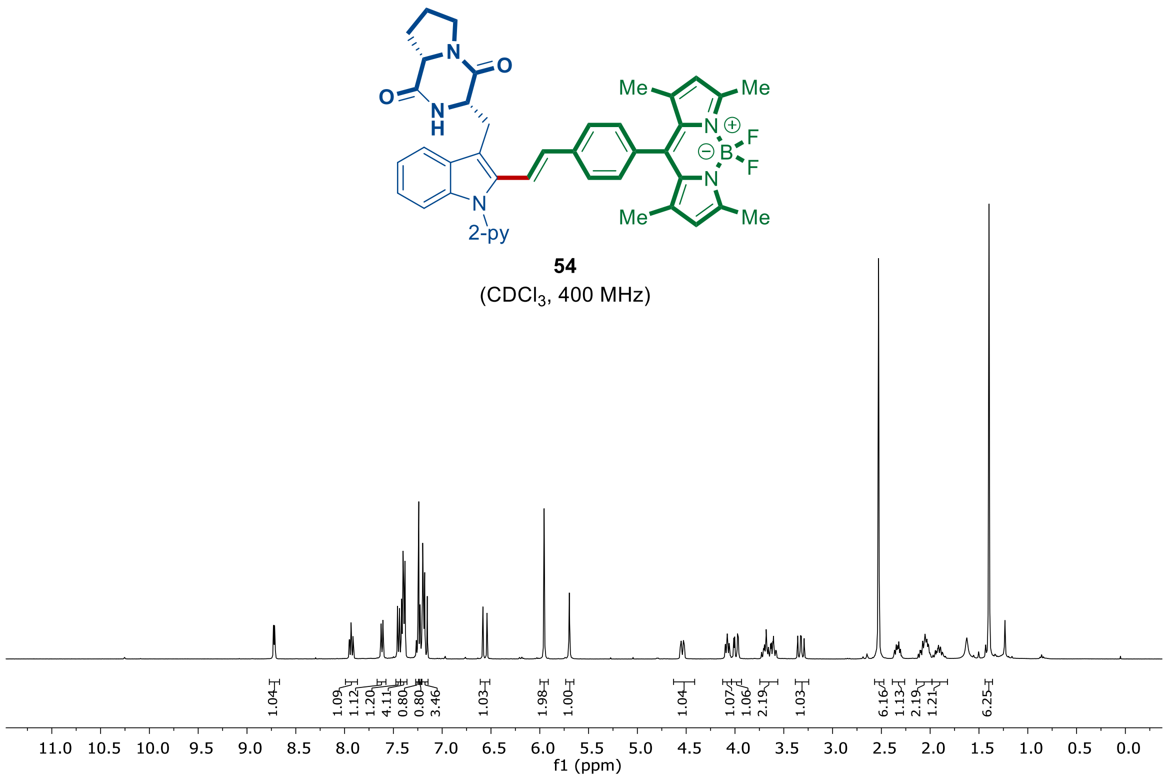

Supplementary Figure 180. ^{1}H-NMR spectrum of **54**.

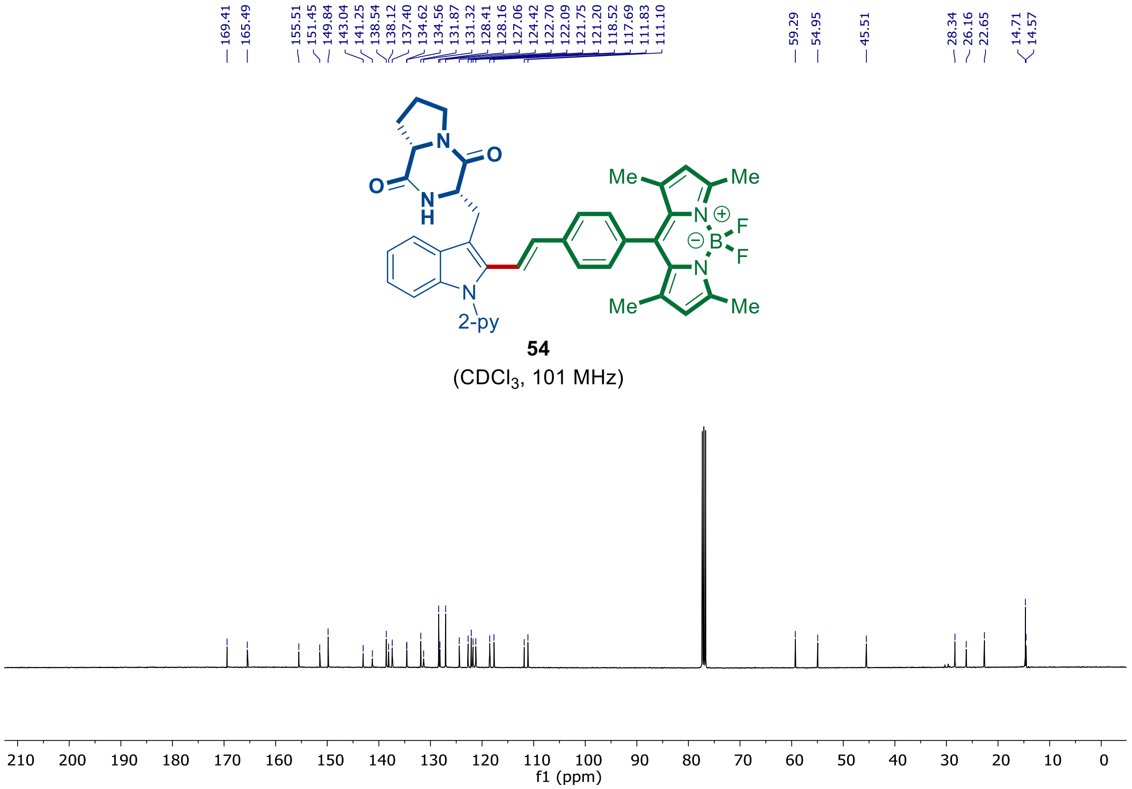

Supplementary Figure 181. ^{13}C-NMR spectrum of **54**.

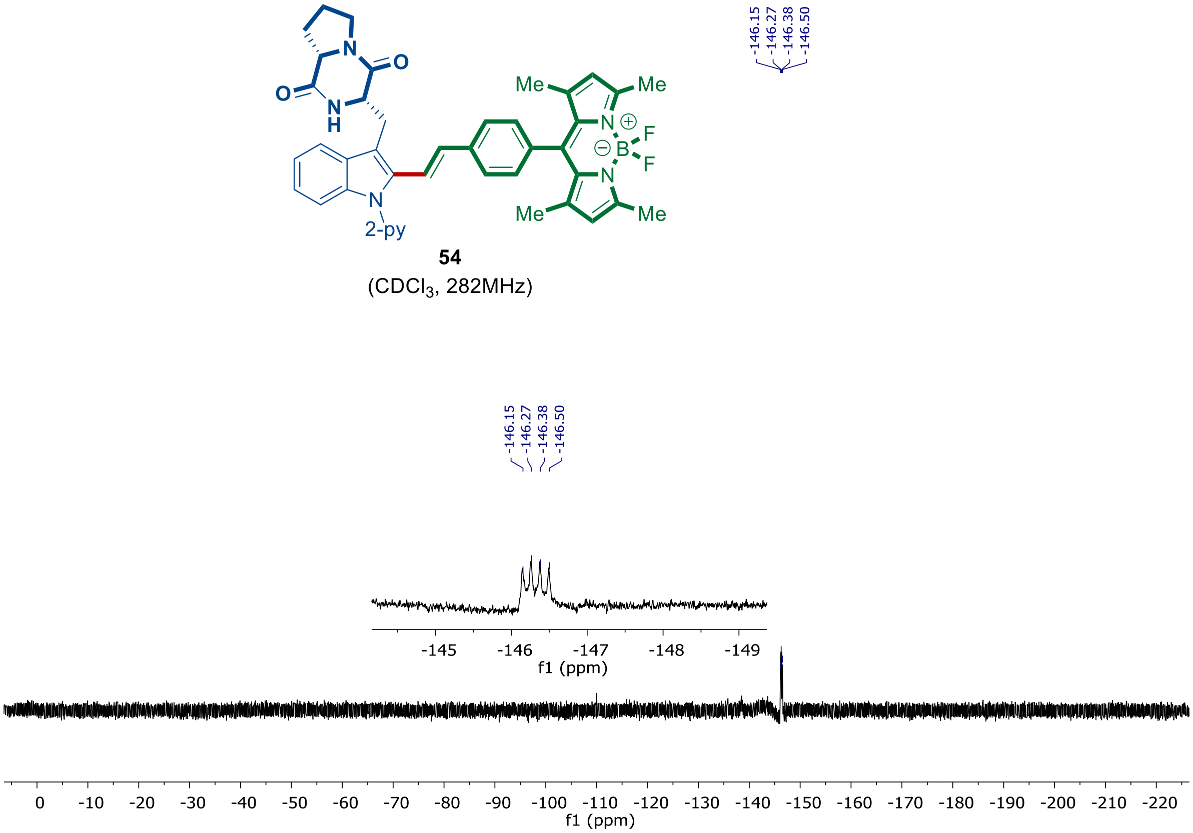

Supplementary Figure 182. ^{19}F-NMR spectrum of **54**.

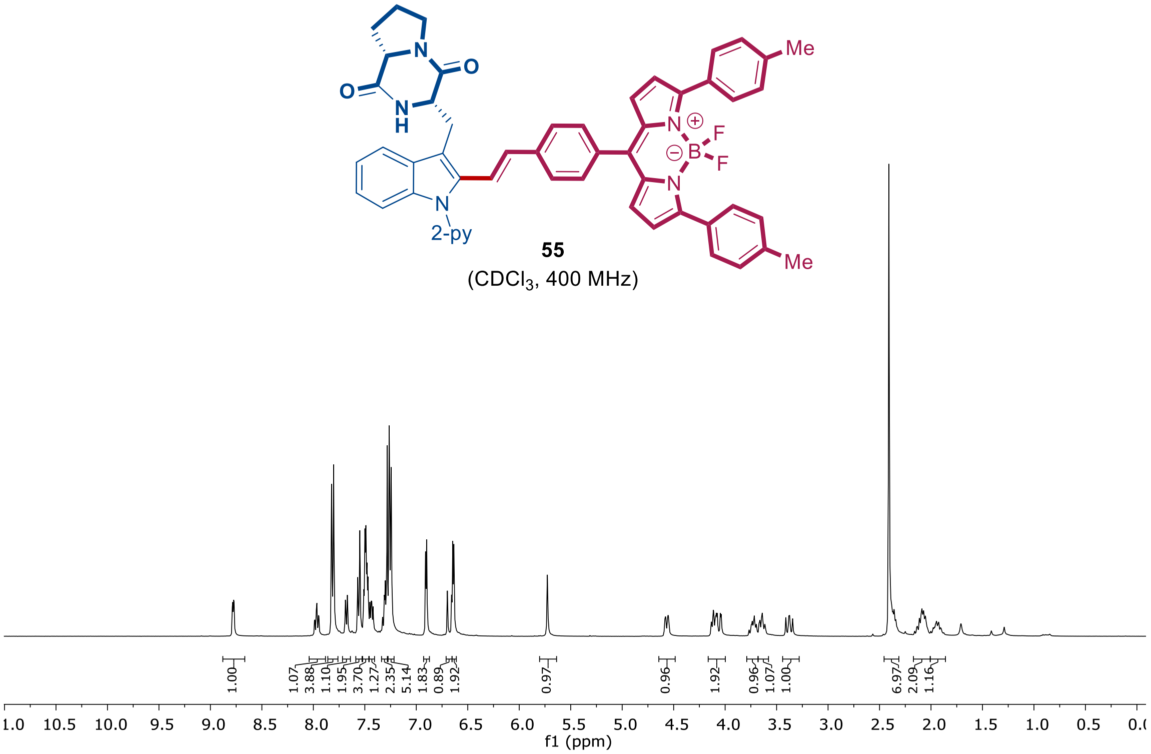

Supplementary Figure 183. ^{1}H-NMR spectrum of **55**.

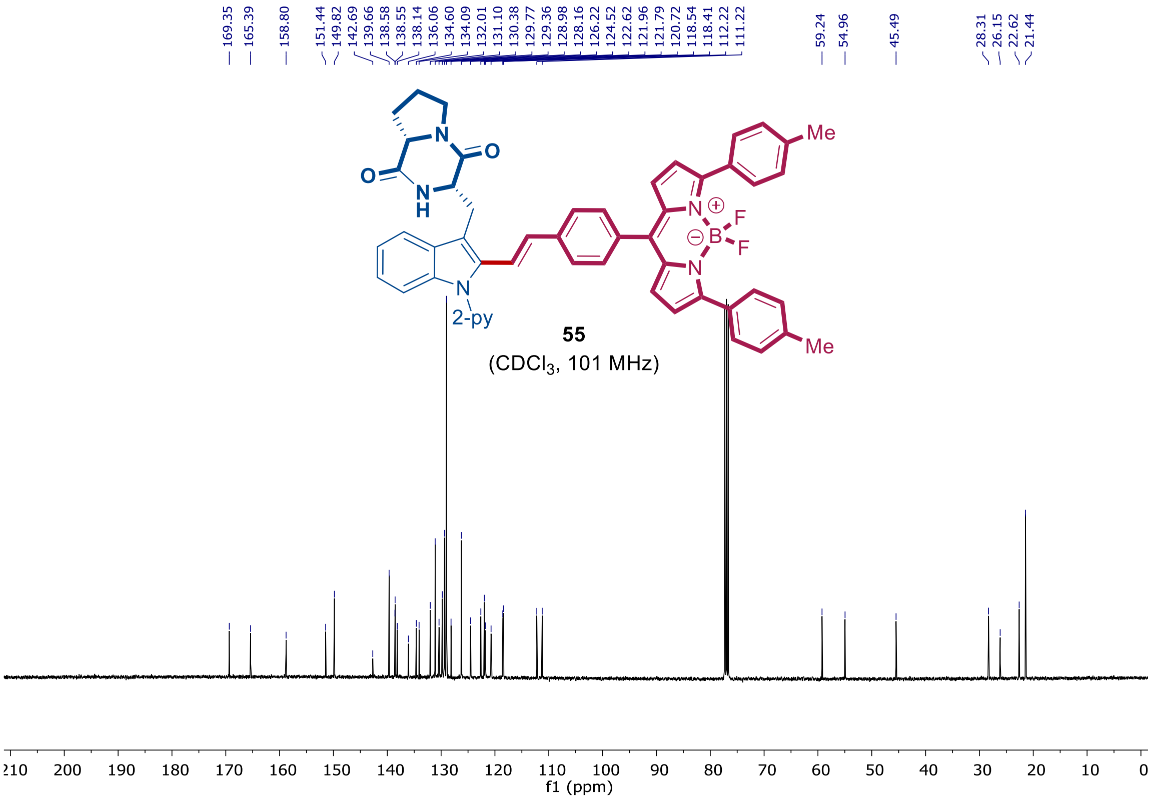

Supplementary Figure 184. ^{13}C-NMR spectrum of **55**.

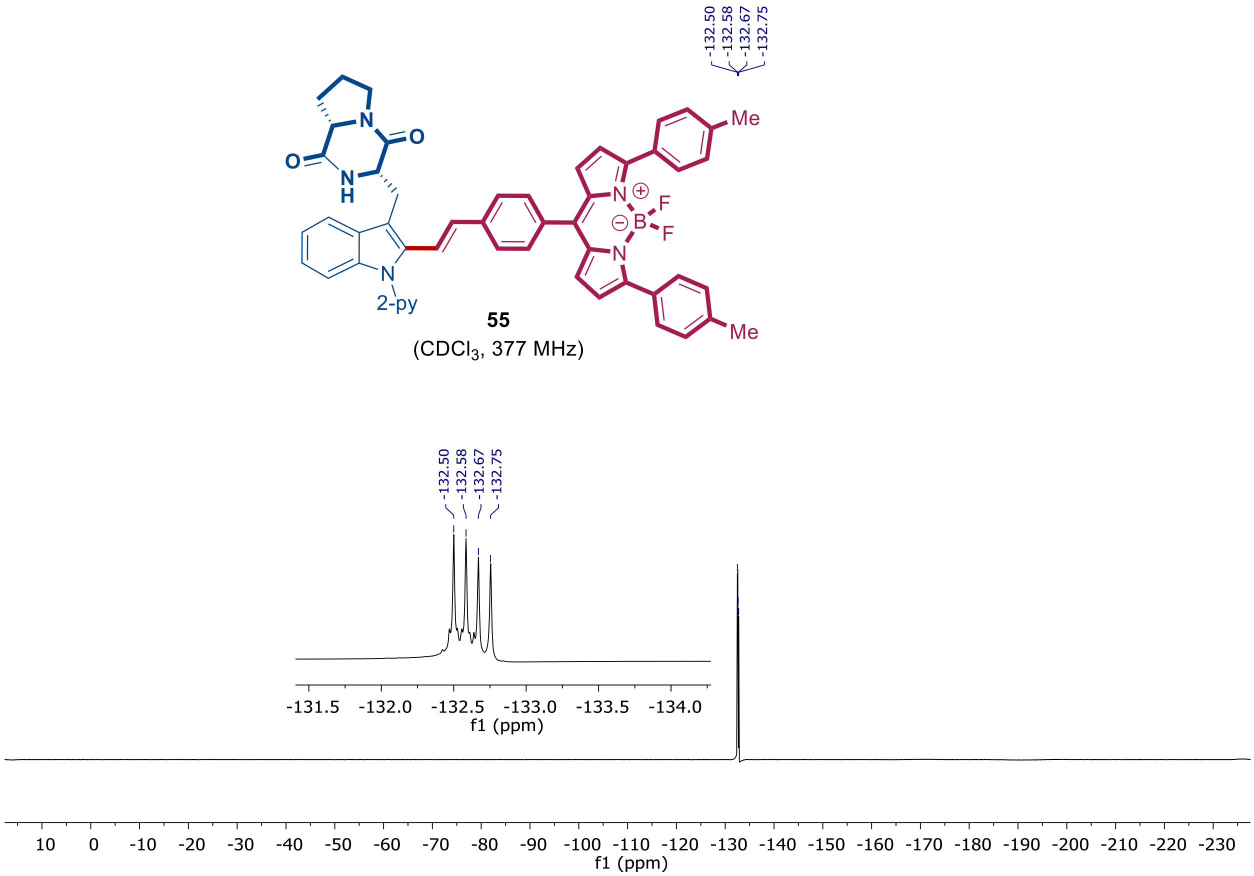

Supplementary Figure 185. ^{19}F-NMR spectrum of **55**.

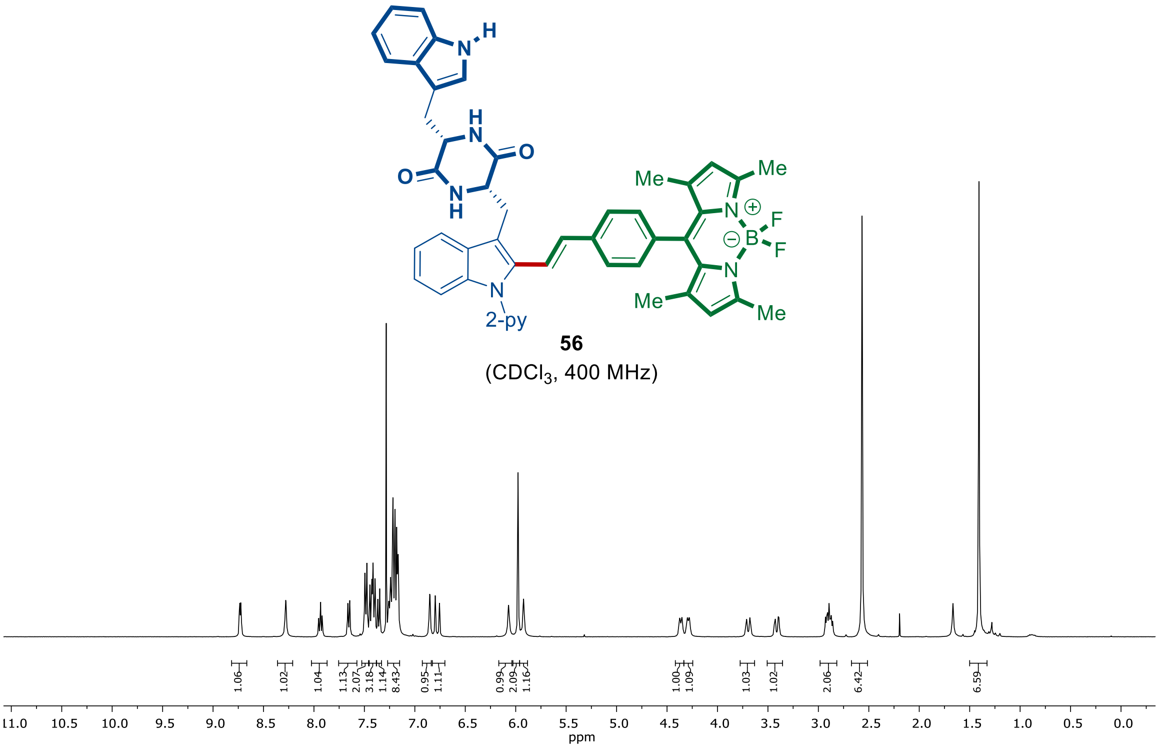

Supplementary Figure 186. ^{1}H-NMR spectrum of **56**.

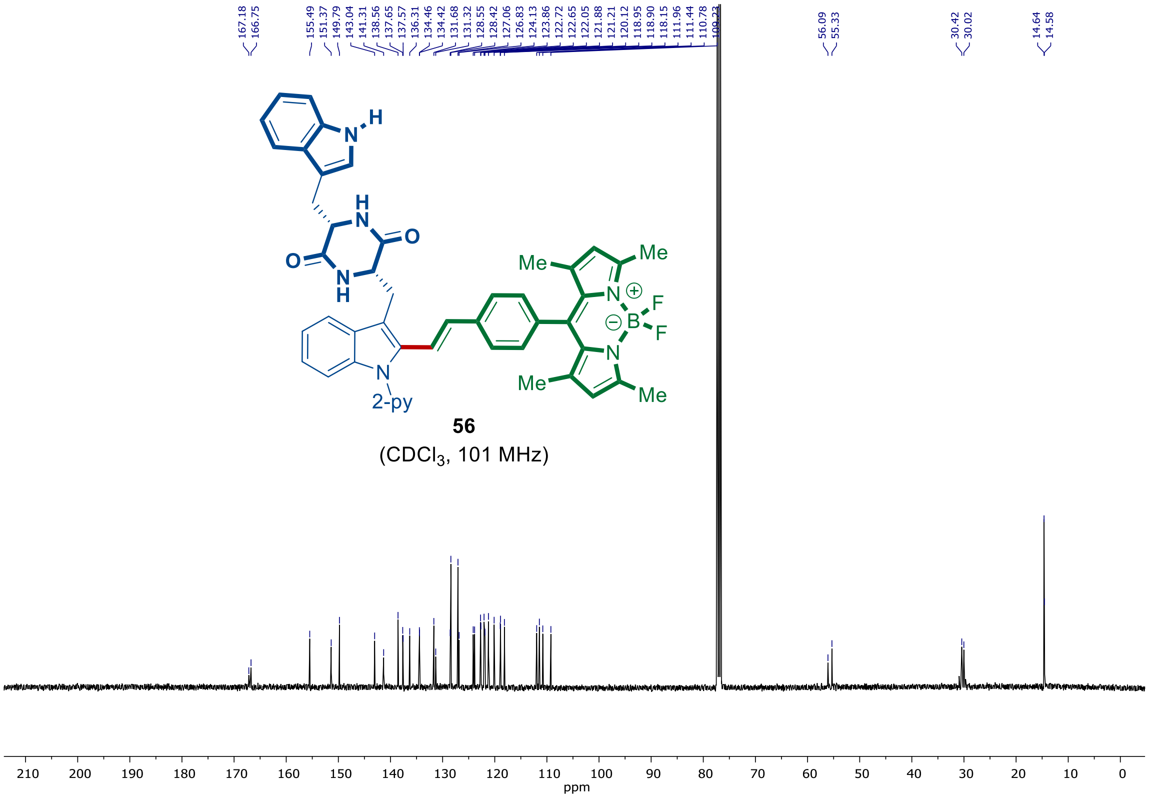

Supplementary Figure 187. ^{13}C-NMR spectrum of **56**.

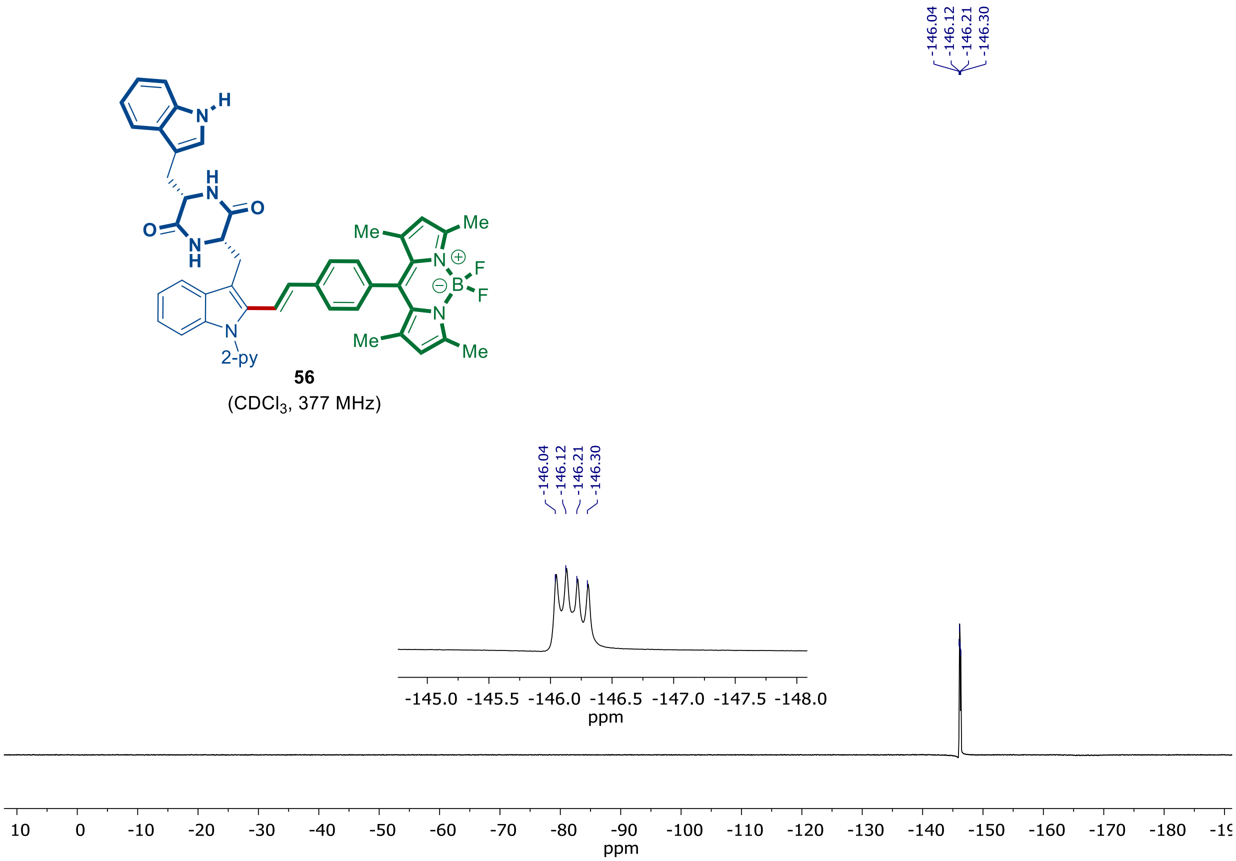

Supplementary Figure 188. ^{19}F-NMR spectrum of **56**.

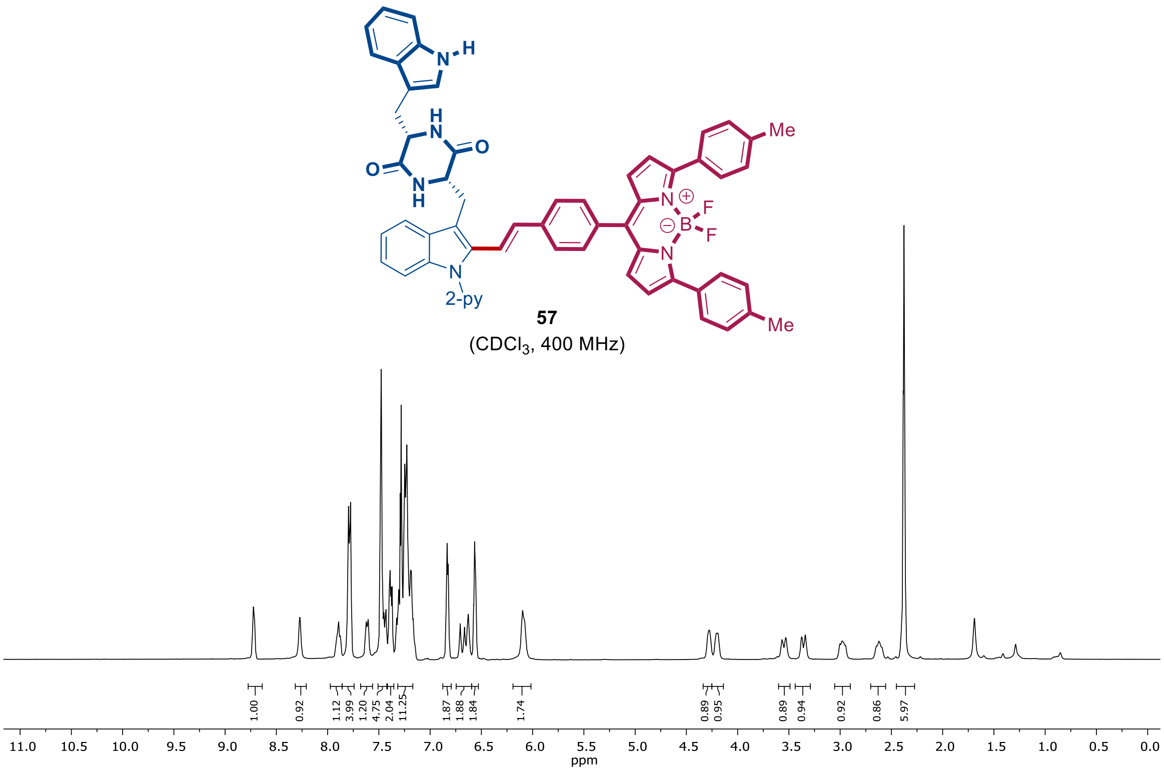

Supplementary Figure 189. ^{1}H-NMR spectrum of **57**.

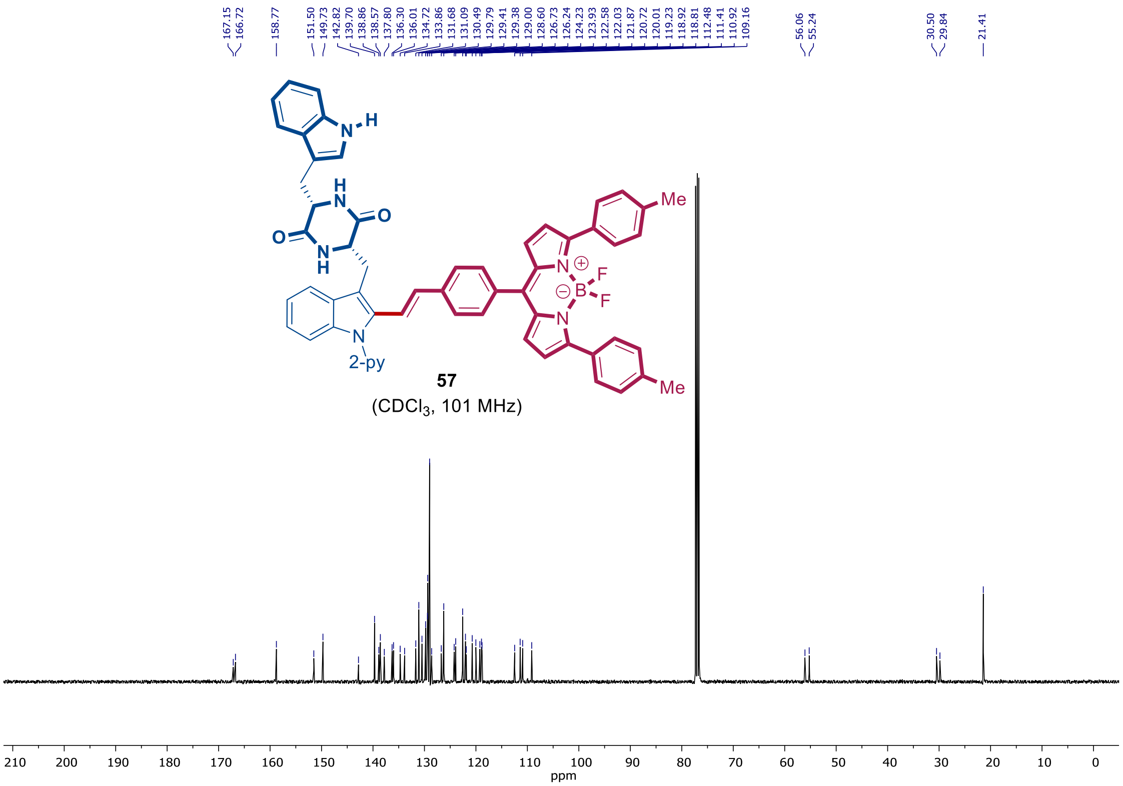

Supplementary Figure 190. ^{13}C-NMR spectrum of **57**.

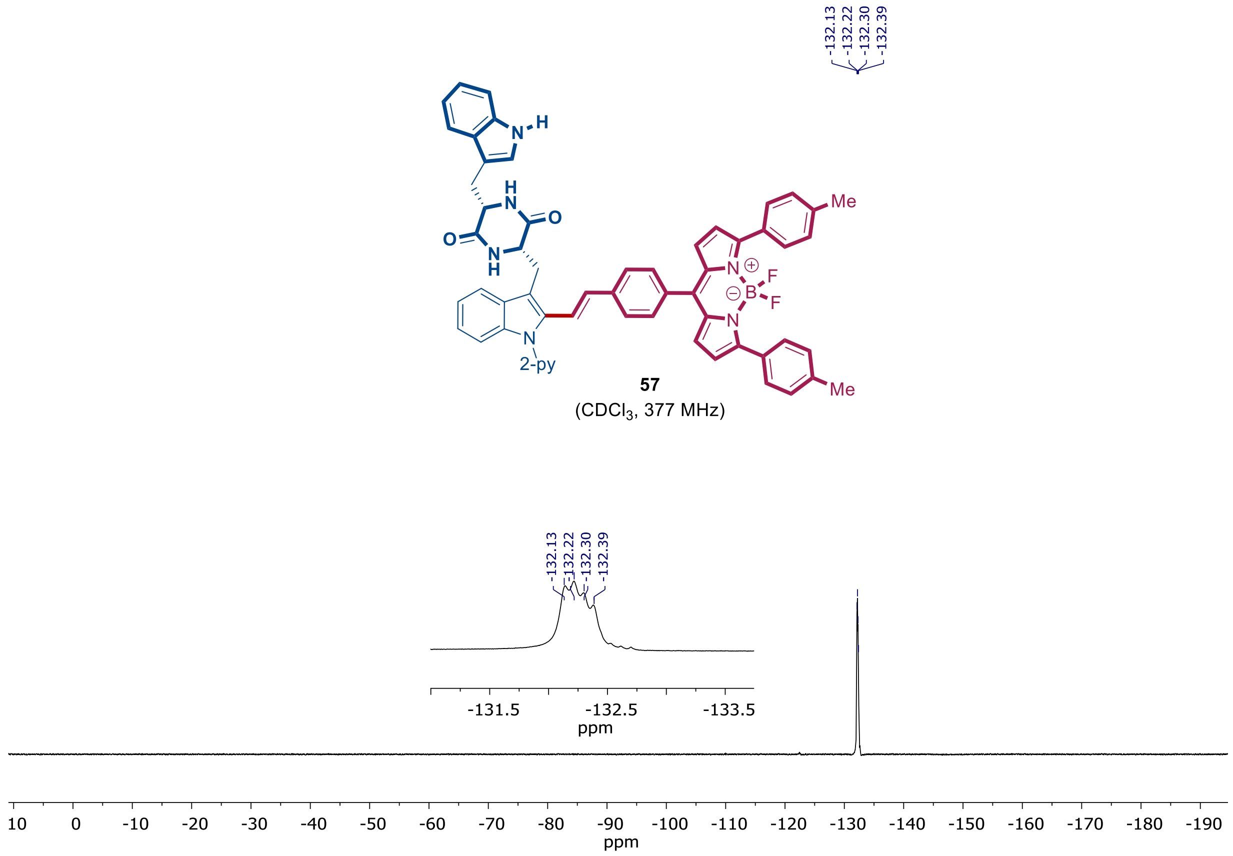

Supplementary Figure 191. ^{19}F-NMR spectrum of **57**.

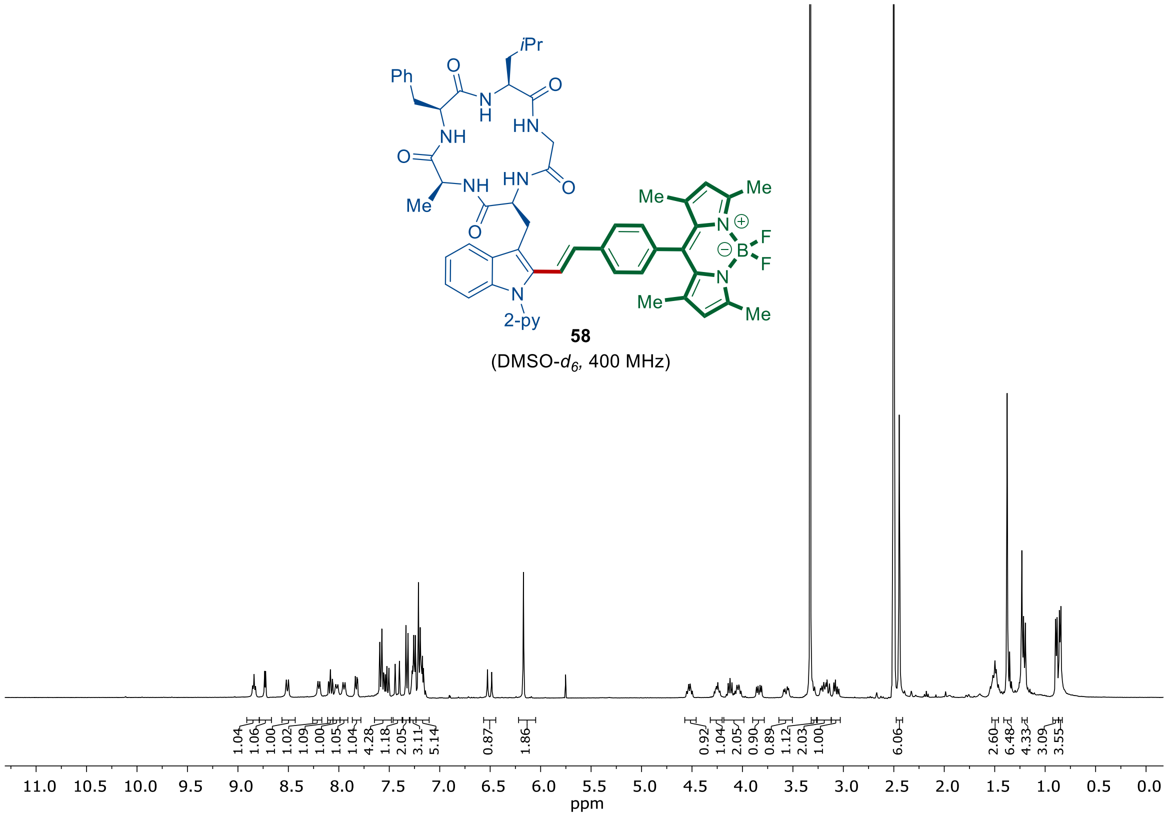

Supplementary Figure 192. ^{1}H-NMR spectrum of **58**.

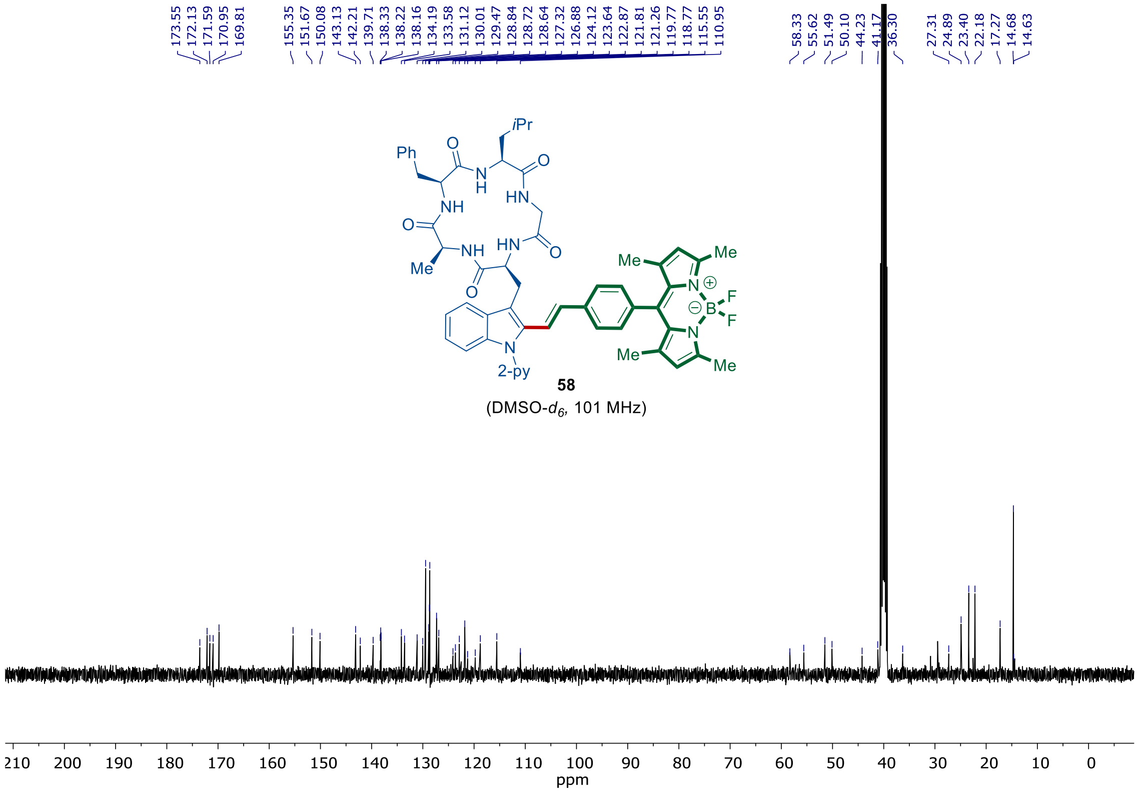

Supplementary Figure 193. ^{13}C-NMR spectrum of **58**.

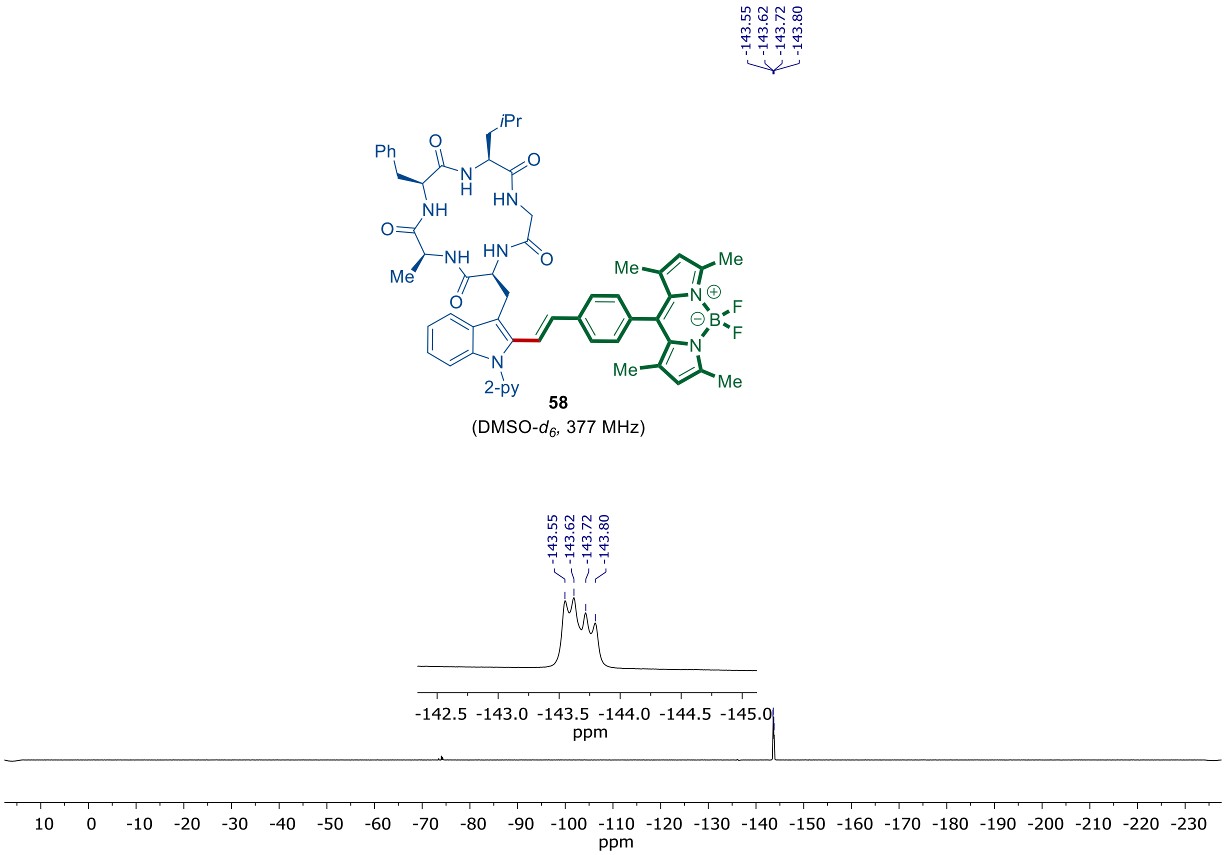

Supplementary Figure 194. ^{19}F-NMR spectrum of **58**.

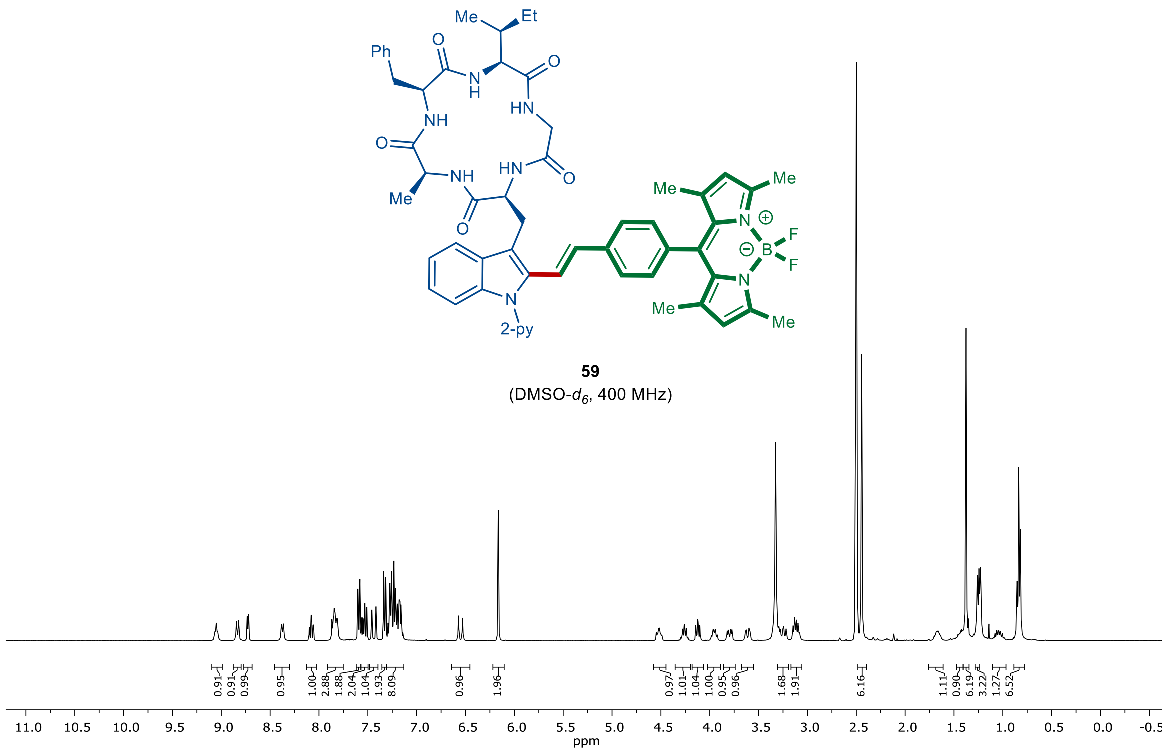

Supplementary Figure 195. ^{1}H-NMR spectrum of **59**.

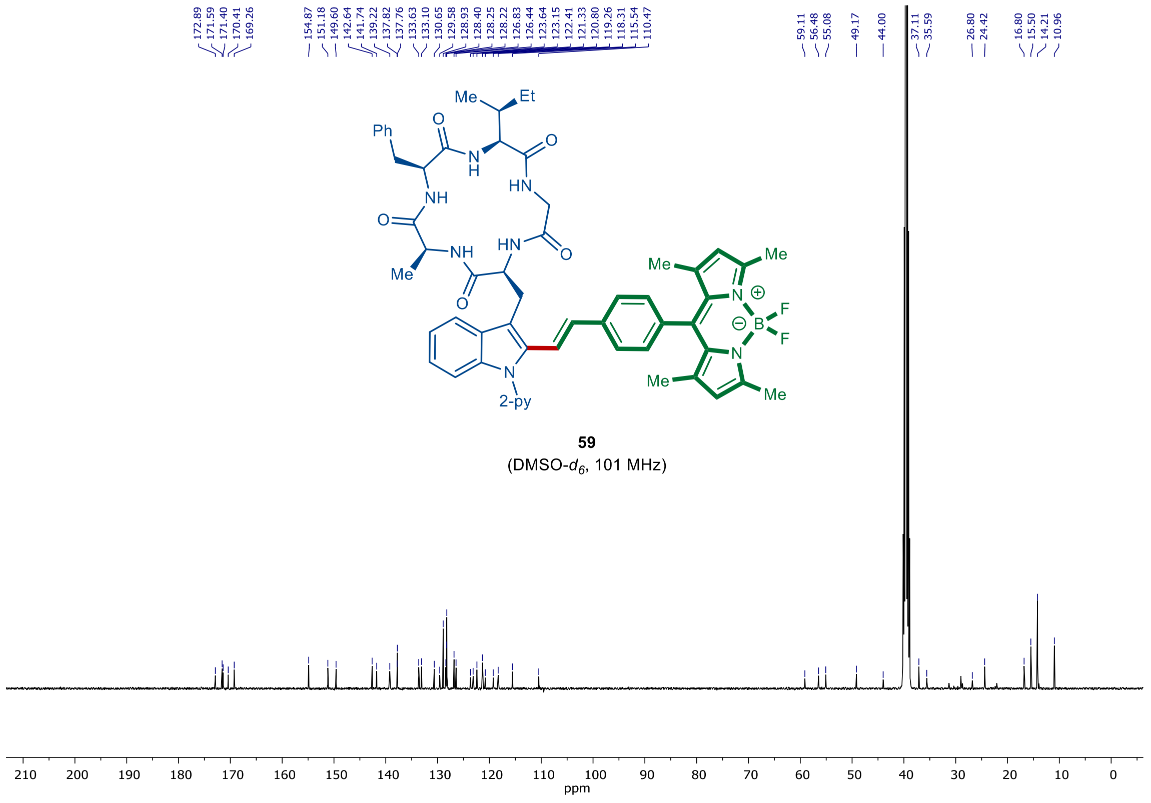

Supplementary Figure 196. ^{13}C-NMR spectrum of **59**.

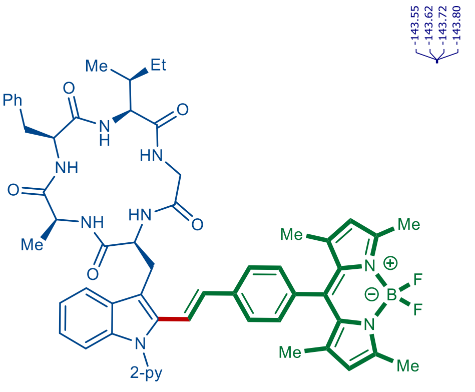

59
(DMSO-d_6, 377 MHz)

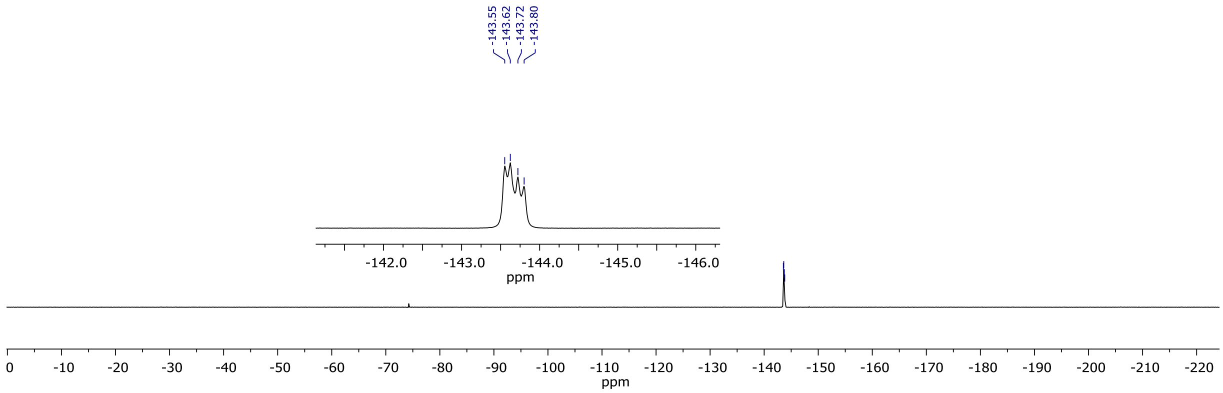

Supplementary Figure 197. ^{19}F-NMR spectrum of **59**.

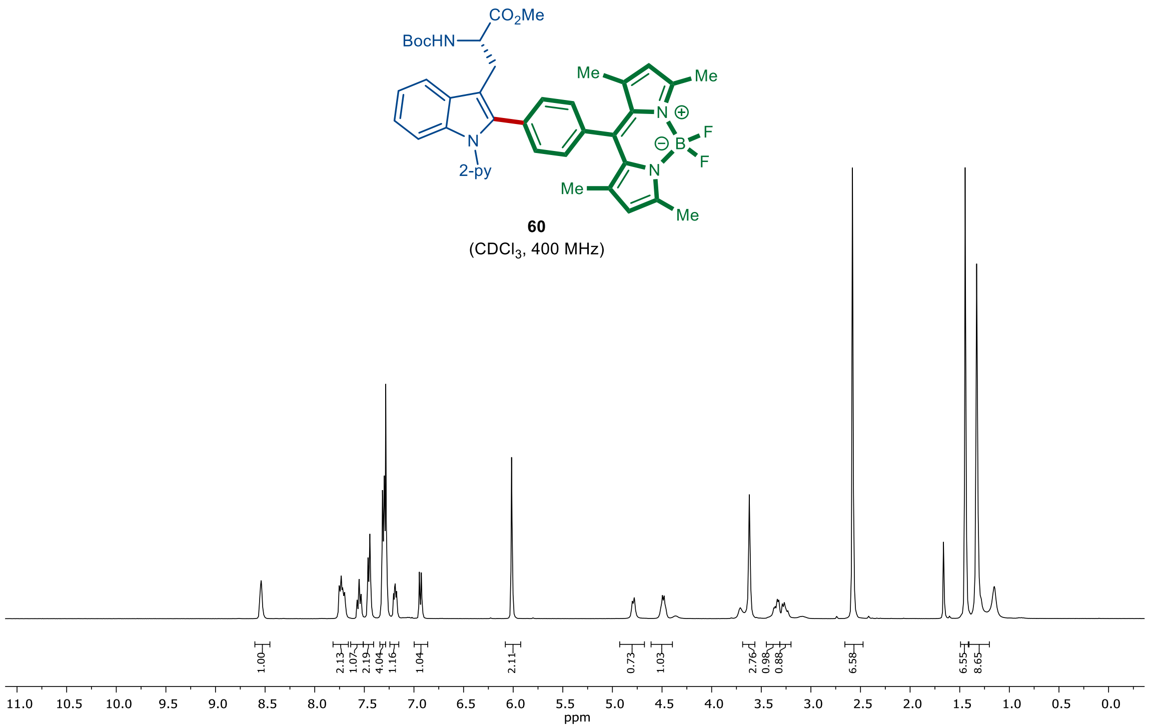

Supplementary Figure 198. ^{1}H-NMR spectrum of **60**.

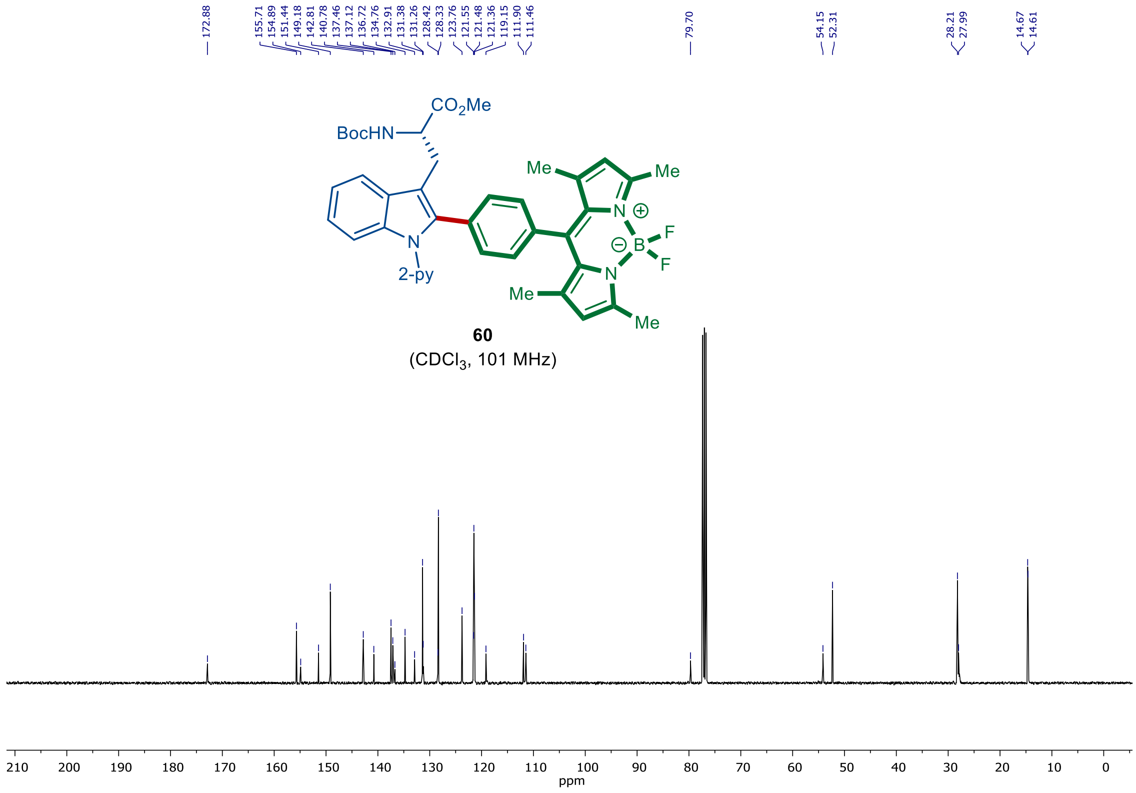

Supplementary Figure 199. ^{13}C-NMR spectrum of **60**.

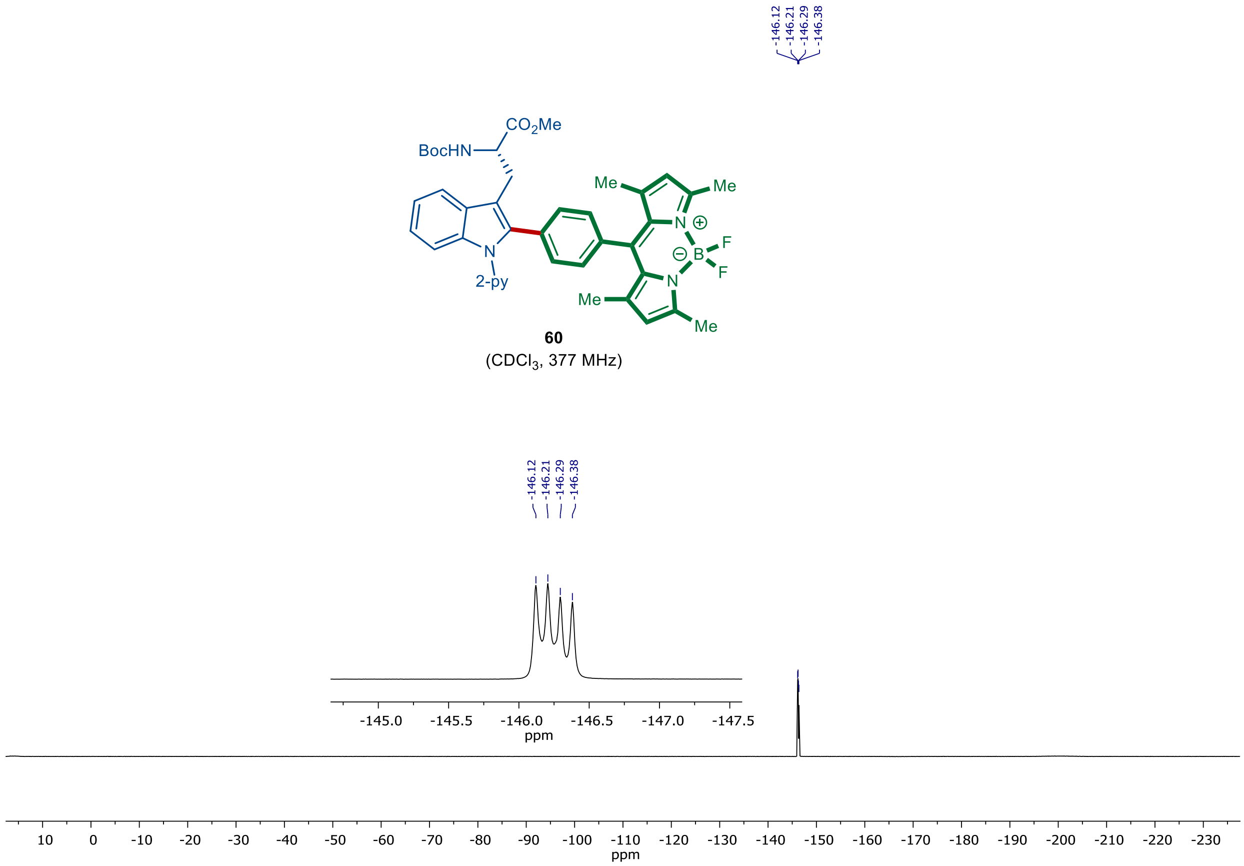

Supplementary Figure 200. ^{19}F-NMR spectrum of **60**.

Supplementary References

1. Schischko, A., Ren, H., Kaplaneris, N. & Ackermann, L. Bioorthogonal Diversification of Peptides through Selective Ruthenium(II)-Catalyzed C–H Activation. *Angew. Chem. Int. Ed.* **56**, 1576-1580 (2017).
2. Cheng, N. S. Formula for the viscosity of a glycerol-water mixture. *Ind. Eng. Chem. Res.* **47**, 3285–3288 (2008).
3. Brouwer, A. M. Standards for photoluminescence quantum yield measurements in solution. *Pure Appl. Chem.* **83,** 2213–2228 (2011).
4. Meares, A., Satraitis, A., Santhanam, N., Yu, Z. & Ptaszek M. Deep-Red Emissive BODIPY–Chlorin Arrays Excitable with Green and Red Wavelengths. *J. Org. Chem.* **80,** 3858–3869 (2015).
5. Meyer, T. H., Liu, W., Feldt, M., Wuttke, A., Mata, R. A. & Ackermann, L. Manganese(I)-Catalyzed Dispersion-Enabled C−H/C−C Activation. *Chem. Eur. J.* **23**, 5443–5447 (2017).
