# Supplementary material for: Chemodivergent manganese-catalyzed C–H activation: modular synthesis of fluorogenic probes
Source: Nat Commun. 2021 Jun 7;12:3389. doi: 10.1038/s41467-021-23462-9 (PMC8185085; doi:10.1038/s41467-021-23462-9)
Supplement: Supplementary file 2 — Reporting Summary [file 41467_2021_23462_MOESM2_ESM.pdf]

nature research

Corresponding author(s): Lutz Ackermann/ Marc Vendrell

Last updated by author(s): Apr 2, 2021

Reporting Summary

Nature Research wishes to improve the reproducibility of the work that we publish. This form provides structure for consistency and transparency in reporting. For further information on Nature Research policies, see our Editorial Policies and the Editorial Policy Checklist.

Statistics

For all statistical analyses, confirm that the following items are present in the figure legend, table legend, main text, or Methods section.

n/a	Confirmed	
☐	☒	The exact sample size (*n*) for each experimental group/condition, given as a discrete number and unit of measurement
☐	☒	A statement on whether measurements were taken from distinct samples or whether the same sample was measured repeatedly
☐	☒	The statistical test(s) used AND whether they are one- or two-sided<br>*Only common tests should be described solely by name; describe more complex techniques in the Methods section.*
☒	☐	A description of all covariates tested
☒	☐	A description of any assumptions or corrections, such as tests of normality and adjustment for multiple comparisons
☐	☒	A full description of the statistical parameters including central tendency (e.g. means) or other basic estimates (e.g. regression coefficient) AND variation (e.g. standard deviation) or associated estimates of uncertainty (e.g. confidence intervals)
☐	☒	For null hypothesis testing, the test statistic (e.g. *F*, *t*, *r*) with confidence intervals, effect sizes, degrees of freedom and *P* value noted<br>*Give P values as exact values whenever suitable.*
☒	☐	For Bayesian analysis, information on the choice of priors and Markov chain Monte Carlo settings
☒	☐	For hierarchical and complex designs, identification of the appropriate level for tests and full reporting of outcomes
☒	☐	Estimates of effect sizes (e.g. Cohen's *d*, Pearson's *r*), indicating how they were calculated

Our web collection on statistics for biologists contains articles on many of the points above.

Software and code

Policy information about availability of computer code

Data collection	Standard commercial softwares were used for data collection. Specifically, BD 5Laser LSR: BD FACSDIVA V8.0; Leica SP8: Leica Application Suite X version 3.5.7.232225; Synergy HT spectrophotometer: Gen 5.
Data analysis	Data analysis, including statistical analysis, was performed using Graphpad Prism 8.0. FlowJo V10 was used to analyze flow cytometry/ FACS data collected using the 5L LSR. Fluorescence image analysis was performed with Fiji ImageJ 1.52b

For manuscripts utilizing custom algorithms or software that are central to the research but not yet described in published literature, software must be made available to editors and reviewers. We strongly encourage code deposition in a community repository (e.g. GitHub). See the Nature Research guidelines for submitting code & software for further information.

Data

Policy information about availability of data

All manuscripts must include a data availability statement. This statement should provide the following information, where applicable:

- Accession codes, unique identifiers, or web links for publicly available datasets
- A list of figures that have associated raw data
- A description of any restrictions on data availability

All data supporting the findings of this study are available in Supplementary Information file or from the authors upon reasonable request.

Field-specific reporting

Please select the one below that is the best fit for your research. If you are not sure, read the appropriate sections before making your selection.

- [x] Life sciences
- [ ] Behavioural & social sciences
- [ ] Ecological, evolutionary & environmental sciences

For a reference copy of the document with all sections, see nature.com/documents/nr-reporting-summary-flat.pdf

Life sciences study design

All studies must disclose on these points even when the disclosure is negative.

Sample size	Sample/replicate size: The minimum number of experimental repeats was n=3, in line with current standards in the field. Numbers for original data of the manuscript were based on previous experience. Sample size was not predetermined using statistical methods. From our own experience, we were able to detect significant alteration in T cells upon activation when sample size was n=3. Further, Yang and colleagues (Yang et al., Nature, 2016, doi:10.1038/nature17412) demonstrated that there is a significant impact of ACAT1 knockout (cholesterol esterification enzyme) on activated T cells as well as their cholesterol levels when n=4. In assumption, that we will be able to use our lead probe to detect similar differences, we assumed a sample size of four would as sufficient.
Data exclusions	No data was exluded from the analyses.
Replication	Several replicates were carried out to verify the reproducibility of the experimental findings. All attempts at replication were successful. Spectral properties were validated from two independent experiments and replicates performed as triplicates (Fig. 5a-b). Fluorescence intensity of compound 41-44 was determined in the presence of liposomes in four independent experiments and replicates performed as triplicates (Fig. 5c). Experiments such as flow cytometry or confocal imaging involving human subjects were performed with at least three independent donors as indicated in sample size. Experiments involving Jurkat T cell line were performed in duplicates in three or more independent experiments (Fig. 5d, Fig. 6).
Randomization	No randomization method was used for experiments. Cells from human peripheral blood have been allocated a specific donor coding system to protect donor anonymity and allow exact recording of experimental use without biases. For experiments other than those involving human subjects, samples were allocated randomly.
Blinding	Blinding was not relevant to the study because investigators knowdledge of the sample allocation did not interference on the data collection, analysis and results.

Reporting for specific materials, systems and methods

We require information from authors about some types of materials, experimental systems and methods used in many studies. Here, indicate whether each material, system or method listed is relevant to your study. If you are not sure if a list item applies to your research, read the appropriate section before selecting a response.

Materials & experimental systems

n/a	Involved in the study	
[ ]	[x]	Antibodies
[ ]	[x]	Eukaryotic cell lines
[x]	[ ]	Palaeontology and archaeology
[x]	[ ]	Animals and other organisms
[ ]	[x]	Human research participants
[x]	[ ]	Clinical data
[x]	[ ]	Dual use research of concern

Methods

n/a	Involved in the study	
[x]	[ ]	ChIP-seq
[ ]	[x]	Flow cytometry
[x]	[ ]	MRI-based neuroimaging

Antibodies

Antibodies used	human anti-CD3 (supplier name: Biolegend; Cat. number: 317301; clone name: OKT3; lot number: B269636); human anti-CD28 (supplier name: Biolegend; Cat. number: 302901; clone name: CD28.2; lot number: B288170); anti-human PD-1-PE (supplier name: Biolegend; Cat. number: 329905; clone name: EH12.2H7; lot number: B252642); anti-human CD62L-PE/Cy7 (supplier name: Biolegend; Cat. number: 304821; clone name: DREG-56; lot number: B242742); anti-human CD8-PerCP/Cy5.5 (supplier name: Biolegend; Cat. number: 300924; clone name: HIT8a; lot number: B285302); Anti-human Granzyme B-APC (supplier name: Biolegend, Cat. number: MHGB05, Clone: GB12, lot number: 20156588); Anti-human CD39-APC (supplier name: Biotec, Cat. number: 120-100-459, Clone: MZ18-23C8, lot number: 5200109640); Anti-human CD3-PE-CF594 (supplier name: BD Biosciences, Cat. number: 562280, Clone: UCHT1, lot number: 9102751); Anti-human IFNg-APC-Cy7 (supplier name: Biolegend, Cat. number: 506524, Clone: B27, lot number: B3173); Anti-human Granzyme A-AF488 (supplier name: Biolegend, Cat. number: 507212, Clone: CB9, lot number: B26-564); Anti-CD86-ef450 (supplier name: Invitrogen, Cat. number: 48-0862-82, Clone: GL1, lot number: 4336369).

Validation	Validation was performed as indicated in the manufacturer's website. Antibodies have been rigorously tested and validated using IHC, Western blotting and Flow cytometry. Human anti-CD3 has been used in 45 publications, see more detail on manufacturer's webpage (https://www.biolegend.com/it-it/products/purified-anti-human-cd3-antibody-3642). Human anti-CD28 has been referenced in 34 papers, please see: https://www.biolegend.com/en-us/products/purified-anti-human-cd28-antibody-632?GroupID=GROUP28. Information of PE-conjugated PD-1 can be found in 35 papers, please see: https://www.biolegend.com/en-us/products/pe-anti-human-cd279-pd-1-antibody-4412?GroupID=GROUP28. PE/Cy7-conjugated anti-human CD62L has been used in eight studies, please see: https://www.biolegend.com/en-us/products/pe-cyanine7-anti-human-cd62l-antibody-3944?GroupID=GROUP28. Flow cytometry has been performed with PerCP-Cy5.5. conjugated anti-human CD8 in the following 12 papers, please see: https://www.biolegend.com/en-us/products/percp-cyanine5-5-anti-human-cd8-antibody-6389. APC conjugated anti-human Granzyme B has been referenced in 43 papers, please see https://www.thermofisher.com/antibody/product/Granzyme-B-Antibody-clone-GB12-Monoclonal/MHGB05. APC-CD39 has been used in one paper, please see: https://www.miltenyibiotec.com/GB-en/products/cd39-antibody-anti-human-mz18-23c8.html#gref. PE-CF594-CD3 has been validated by BD Biosciences and referenced in 6 papers, plase see: https://bdbiosciences.com/us/applications/research/t-cell-immunology/th-1-cells/surface-markers/human/pe-cf594-mouse-anti-human-cd3-ucht1-also-known-as-ucht-1-ucht-1/p/562280. Granzyme A -AF488 has been referenced in two papers, please see: https://www.biolegend.com/en-us/products/alexa-fluor-488-anti-human-granzyme-a-antibody-3498. IFNg-APC/Cy7 has been validated by Biolegend and published in six papers, please see: https://www.biolegend.com/de-de/products/apc-cyanine7-anti-human-ifn-gamma-antibody-12330. CD86-ef450 has been validated by invitrogen and used in 38 publications, please see: https://www.thermofisher.com/antibody/product/CD86-B7-2-Antibody-clone-GL1-Monoclonal/48-0862-82.

Eukaryotic cell lines

Policy information about cell lines

Cell line source(s)	Jurkat T cells were obtained from ATCC.
Authentication	Granzyme B and CD3 staining was performed to confirm identity of cell lines.
Mycoplasma contamination	Mycoplasma was not detected using the MycoAleret Mycoplasma Detetection Kit (Lonza).
Commonly misidentified lines (See ICLAC register)	The cell line used (Jurkat T cells) do not belong to misidentified cell line.

Human research participants

Policy information about studies involving human research participants

Population characteristics	Healthy male and female volunteers were used for this study (age range: 20-60 years). All donor samples were anonymized following blood sample collection. Information (sex, age, etc) was recorded but it was not made available to the researchers.
Recruitment	Participants for the T cell isolation from blood were randomly recruited according to their availability. No potential self-selection bias was expected. Healthy volunteers were randomly selected with availability (based on sample volume and frequency of donation) according to local ethical guidelines of the blood donor register. Information on donors was limited to age and gender and blinded for analysis of experiments. Work complied with all relevant ethical regulations and informed consent was obtained. The study protocol was approved by the Accredited Medical Regional Ethics Committee (AMREC, reference number 20-HV-069) at the University of Edinburgh.
Ethics oversight	Ethical approval was received by the Accredited Medical Regional Ethics Committee (AMREC, reference number 20-HV-069).

Note that full information on the approval of the study protocol must also be provided in the manuscript.

Flow Cytometry

Plots

Confirm that:

- [x] The axis labels state the marker and fluorochrome used (e.g. CD4-FITC).
- [x] The axis scales are clearly visible. Include numbers along axes only for bottom left plot of group (a 'group' is an analysis of identical markers).
- [x] All plots are contour plots with outliers or pseudocolor plots.
- [x] A numerical value for number of cells or percentage (with statistics) is provided.

Methodology

Sample preparation	Sample preparation was performed as described in detail in Online Methods.Briefly, cells were washed once with HEPES-NaCl buffer containing 0.1% BSA before resuspenion in HEPES-NaCl buffer. Antibody stainings were performed for 20 min at 4 C.
Instrument	BD 5L LSR
Software	Data collection was performed with FACS DIVA V8.0 software. Data was analysed with FlowJo Software V10.

Cell population abundance	For flow cytometry experiments, 10,000 events were acquired for every population of interest.
Gating strategy	Firstly, cells were selected based on their FSC/SSC properties followed by gating on singlet cells using FSC-A and FSC-H plots to exclude debris and non-singlet events.

☒ Tick this box to confirm that a figure exemplifying the gating strategy is provided in the Supplementary Information.
